# Sensory genes identification with head transcriptome of the migratory armyworm, *Mythimna separata*

**DOI:** 10.1038/srep46033

**Published:** 2017-04-07

**Authors:** Zhenxing Liu, Xiaoyun Wang, Chaoliang Lei, Fen Zhu

**Affiliations:** 1 Hubei Insect Resources Utilization and Sustainable Pest Management Key Laboratory, Huazhong Agricultural University, Wuhan 430070, China

## Abstract

Sensory system plays important roles in a wide array of insect’s behavior and physiological events, including the host landing and locating, feeding, flying, sex responding, mating and oviposition which happen independently and in sequence. The armyworm *Mythimna separata* (Lepidoptera: Noctuidae) of migratory insect is destructive for alimentarn crop and economic crop throughout the world. Here we present the high throughput sequencing of the head transcriptome and identify members of the major sensory genes which are crucial for armyworm’s success worldwide, including 8 opsins, 22 chemosensory proteins, 50 odorant binding proteins, 60 odorant receptors, 8 gustatory receptors, 24 ionotropic receptors, and 2 sensory neuron membrane proteins. It is worth noting that a duplication of the LW opsin gene exists in this insect. Several genes were clustered with functionally validated genes, such as Co-receptors of OR and IR, PBPs, PRs, CO2 GRs, bitter GRs and sweet GRs, were also identified. The transcriptome gene library provided the basis for further studies that elucidate the fundamental molecular mechanism of biology and control in *M. separata*. Our research exhibits the first comprehensive catalogue of the sensory genes fundamental for success and distribution in *M. separata*, which are potential novel targets for pest control strategies.

Insects are the most successful living creature consideration of the numbers, the biomass, and their distribution, in which the sensory systems play a significant role in their success. The armyworm *Mythimna separata* (Lepidoptera: Noctuidae) is destructive for crop production in Asia and Oceania with wide plant hosts, such as wheat, maize, rice and many other cereal crops. It is a typical pest that migrate seasonally in long distance of 500–1,000 km and the migration happens at night with downwind^1^,^2^. In china, there are at least four times of migration for *M. separata* which always lead to disastrous pest outbreaks cross generations in massive scales^3^,^4^. For example, the control area of armyworm was up to 8,455,000 hm^2^ and the yearly actual loss of main crops by armyworm reached 692,000 tons during 2012–2013^5^. Therefore, many studies were conducted on the biological and ecological habits, population monitoring, prediction and integrated control of *M. separata*.

It has been known that migration of *M. separata* is an interaction between environment and genetic. Olfactory, visuosensory, and gustatory system are important contributors for *M. separata* to feel, handle and respond to environment cues. These may help us understand the intrinsic factor of its migration. However, the fundamental mechanisms are not well understood. Insect head is often equipped with compound eyes, antennae, mouthpart, and related neurons. Antenna structure of *M. separata* was introduced to explain the behavior mechanism in chemecological level^6^. Recent studies of our group, compound eye fine structures of *M. separata*, would help us to understand the visual-based behavior of *M. separata* (unpublished data).

Transcriptome analysis has been applied in a large number of insects for gene mining and different expressed gene identification during various life processes^7^. Taking the importance of heads in *M. separata* and the completeness of sequence information into consideration, heads from 1–6^th^ larvae, female and male pupae, and female and male adults of *M. separata* were sampled in respective stages. The mRNA of mixed heads was applied for transcriptome analysis. The transcriptome gene library obtained in this study provided the basis for further studies that elucidate the fundamental molecular mechanism of biology and control in *M. separata*.

## Results

### Head transcriptome

Illumina sequencing of the cDNA library prepared from the mRNA of the larvae, pupae and Adults *Mythimna separata* head totally present 123,444,756 raw reads. Trimming adaptor sequences and eliminating low quality reads produced 102,202,512 reads (Supplementary Data S1). After assembly, with scaffolding of the head transcriptomes, there were 228,197 transcripts, from which 156,254 unigenes were predicted, and average length of 416 bp and a maximum length of 29,803 bp. (Supplementary Data S1). The assembled transcripts were used as queries in BLASTx against the database Nr (NCBI non-redundant protein sequences), Nt (NCBI non-redundant nucleotide sequences), GO (Gene Ontology), PFAM (Protein family), KOG/COG (Clusters of Orthologous Groups of proteins), Swiss-Prot (A manually annotated and reviewed protein sequence database), and KO (KEGG Orthology), with an e-value cut-off of 10E−5. In general, the sequences had e-values between 1.0E–4 and 1.0E–10 (Fig. S1). Searches against the databases returned 111,124 transcripts showing sequence similarity to known proteins (Supplementary Data S1). The head transcripts of *M. separata* was most similar to the sequences of *Danaus plexippus*, followed by the sequences of *Bombyx mori* (Fig. S2).

**Figure 1:**
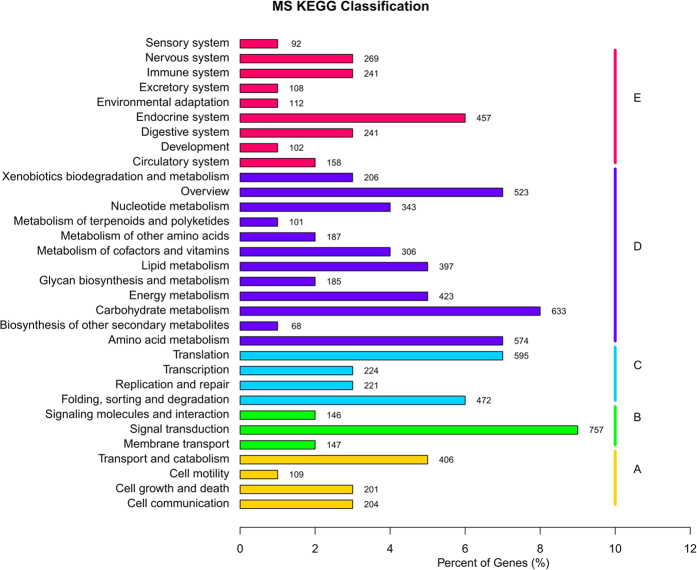
Kyoto Encyclopedia of Genes and Genomes (KEGG) annotation summary. KEGG distribution of the *Mythimna separata* unigenes were annotated by 136 pathways in 5 major groups.

All 7976 unigenes encoded in The KEGG returned cut-off BLAST hits > 1.0E–5. A KEGG metabolic pathway analysis present 5461 transcripts that could generate 136 predicted pathways (Fig. 1). The top 14 KEGG pathways contained over 120 unigenes. For example, 280 sequences belonged to the class “Purine metabolism” (PATH: ko00230), the class “Carbon metabolism” (PATH: ko01200) have 280 sequences too, followed by 260 in the “Biosynthesis of amino acids” (PATH: ko01230) and 212 in “Oxidative phosphorylation” (PATH: ko00190) (Supplementary Data S1).

Of these transcripts, 22,949 (~20.65% of all predicted proteins) were classifies as at least one GO term (Fig. S3). The largest number of GO term associations were related to the fundamental functions of cell; GO terms related to sensory such as binding, stress response, receptor activity and signal transduction as well as enzyme activity were also included in the data sets. A lot of transcripts uncorrelated with GO terms (88,175 transcripts, ~ 79.35%) probably existed as orphan genes.

A summary of the highly-expressed transcripts in the *M. separata* head is present in Supplementary Data S2. The highly-expressed transcripts consisted of cuticular proteins, troponin, OPSINs, CSPs and OBP. Chitin and certain proteins constitute the insect cuticle, of which different properties (flexible or stiff) are manifested according to the developmental stage of insect. Troponin which is composed of three regulatory proteins participates in skeletal muscle and myocardial contraction. However, the contraction mechanism of smooth muscle which is necessary for movement do not require the involvement of troponin. The Opsin (MS|comp149511_c0), OBP (MS|comp141270_c0), and CSPs (MS|comp154133_c0, MS|comp149520_c0, and MS|comp156988_c0) were highly expressed in the head with 5,344.8, 4,073.9, 8,937.6, 7,433.8 and 5,835.5 FPKMs, respectively, which means that they play significant roles in odorant reception (Supplementary Data S2). Major ribosomal protein genes such as ribosomal protein S27a (Rps27a), Rps2, Rps17, Rpl10, Rpl23, and Rpl39 were highly expressed in the head of *M. separata* (Supplementary Data S2). Interestingly, heat shock protein 19.8 was highly expressed in the *M. separata* head, and it showed 77% similarity to *Cydia pomonella* (ADX96000.1).

### Identification of candidate OPSINs

**Figure 2:**
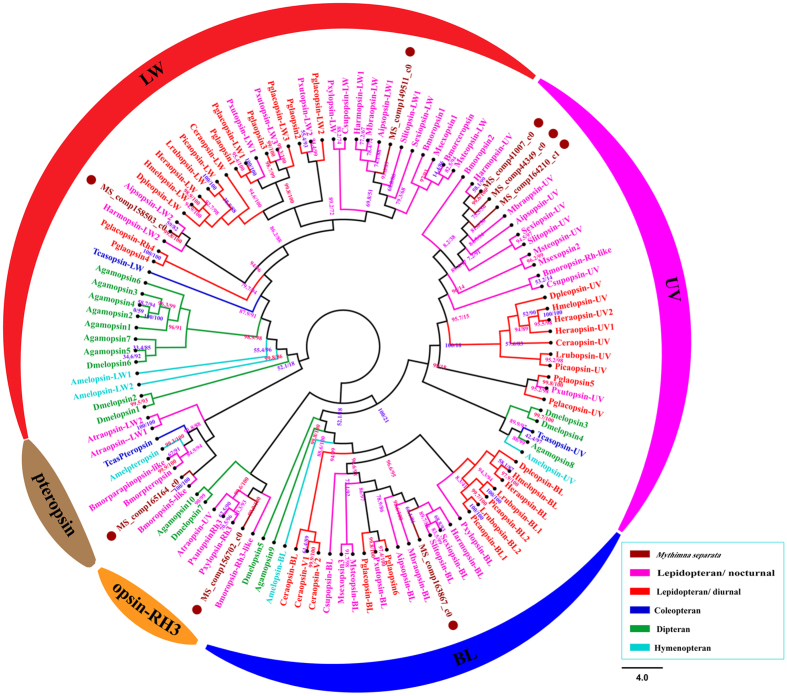
Phylogenetic tree of vision-related opsins (opsins). Included are opsins from Lepidopteran, Coleopteran, Dipteran and Hymenopteran insects of 25 species. *M. separata* opsins are marked with a “•”. The specific clades are marked. Node support was assessed with 1000 bootstrap replicates.

Eight genes were identified as vision-related opsin and four opsins likely represent full-length sequences (Supplementary Data S3). A phylogenetic tree based on the maximum likelihood method was constructed used opsin protein sequences from Lepidopteran, Coleopteran, Dipteran and Hymenopteran insects of 25 species (Fig. 2). Two long wavelength light-sensitive opsins genes were found in several Lepidopterans, which resulted from the early duplication of gene LW1^8^,^9^. Interestingly, we found two LW opsins genes in *M. separata*, LW1 opsin (MS|comp149511_c0) was expressed in higher level (FPKM: 5344.8) than LW2 opsin (MS|comp158503_c0) (FPKM: 30.3). FPKM of blue light-sensitive (BL) opsin (MS|comp163867_c0), ultraviolet light-sensitive (UV) opsin (MS|comp164210_c1), and peropsin (MS|comp165164_c0) were 135.9, 537.4, and 42.1, respectively. Two UV opsin (MS|comp41007_c0, MS|comp44349_c0) and one opsin-RH3-like (MS|comp156702_c0) were also opsin genes with weak expression levels (FPKM < 5) (Supplementary Data S5). Sequence alignment revealed that *M. separata* opsins shared strong homology with opsins identified from other Lepidopteran insects, such as *Helicoverpa armigera, Plutella xylostella, B. mori* and *D. plexippus*. The observed homology was 34.7% ~ 86.0% between opsins in *M. separata* (Supplementary Data S4).

### Identification of candidate chemosensory proteins

Twenty-two transcripts encoding candidate chemosensory proteins (CSPs) were identified (Fig. S4), sixteen of which likely represent full-length proteins. MS|comp154133_c0 and MS|comp149520_c0 are the most highly expressed transcripts (FPKM > 7000) (Supplementary Data S3). A total of the identified full-length proteins included a signal peptide and the highly conserved four-cysteine profile (C1-X6-C2-X18-C3-X2-C4, where X represents any amino acid) (Fig. S4 and Supplementary Data S5). The agreement rate of the deduced CSPs ranged from 2% to 72%, which indicated that they belong to the different CSP protein families (Supplementary Data S4). The protein sequence alignment shows that aromatic residues are highly conserved within the *M. separata* CSP protein family (Fig. S4). hydrophobic residues and alpha helical domains of lepidopteran CSPs were totally retained (Fig. S4).

The CSP sequences of seven lepidopteran species, *Tribolium castaneum* and *Drosophila melanogaster*, were aligned to build an ML tree, which represent three orders (Fig. S4). The lepidopteran specific CSP lineage that diverged from dipterans and coleopterans were formed (Fig. S4). MS|comp149055_c0 and MS|comp158640_c0 are clustered with HarmCSP4. MS|comp154133_c0, MS|comp149520_c0, MS|comp155046_c0 and MS|comp149356_c0 are clustered with BmorCSP1, BmorCSP2, BmorCSP6, HarmCSP6, respectively (Fig. S4).

### Identification of putative odorant binding proteins

**Figure 3:**
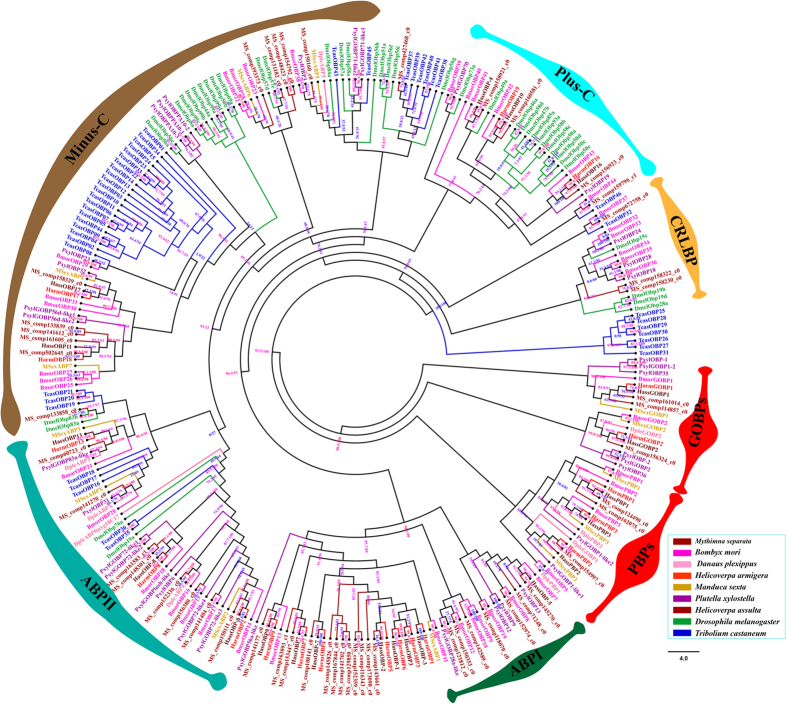
Phylogenetic tree of odorant binding proteins (OBPs). Included are OBPs from *M. separata* (MS), *B. mori* (Bmor), *D. plexippus* (Dple)*, H. armigera* (Harm), *M. sexta* (Msex), *P. xylostella* (Pxyl), *H. assulta* (Hass), *D. melanogaster* (Dmel) and *T. castaneum* (Tcas). The specific clades are marked. Node support was assessed with 1000 bootstrap replicates.

We analyzed 50 odorant binding proteins (OBPs) transcripts. Of the 30 full-length OBPs of *M. separata*, 15 exhibited the classic arrangement of conserved six-cysteines, 12 was the Plus-C gene motif, and 3 were Minus-C (Fig. 3). Table 1 summarized transcript name, length, Signal Peptide, Cysteine Number and FPKM in three biological replicates of putative odorant binding proteins. We also detected low amino acid sequence identity among the full-length OBPs of *M. separata*, which ranged from 5.1% to 68.5%, with an average of 17.9%. Among the 50 OBPs of *M. separata*, MS|comp141270_c0 and MS|comp116343_c0 showed the highest expression level (FPKM > 3000) (Supplementary Data S3).

The 50 OBPs of *M. separata* along with 230 OBPs from 8 other species (including *B. mori, P. xylostella, D. plexippus, H. armigera, Helicoverpa assulta, Manduca sexta, T. castaneum* and *D. melanogaster*) were chosen to construct a phylogenetic tree based on the amino acid sequences. The tree was classified into several distinct branches: the GOBP/PBP family, the CRLBP family, the ABPI family, the ABPII family, the Plus-C OBP family and the Minus-C OBP family (Fig. 3). As expected, the lepidopteran specific GOBP/PBP family were clustered into separate clades away from other OBPs. We also found some OBPs of *M. separata* sharing high homology and closely clustered with DmelOBP49a, DmelOBP57e, DmelOBP49a, BmorPBP1 and HarmPBP1 which have been functionally characterized.

### Identification of candidate odorant receptors

**Figure 4:**
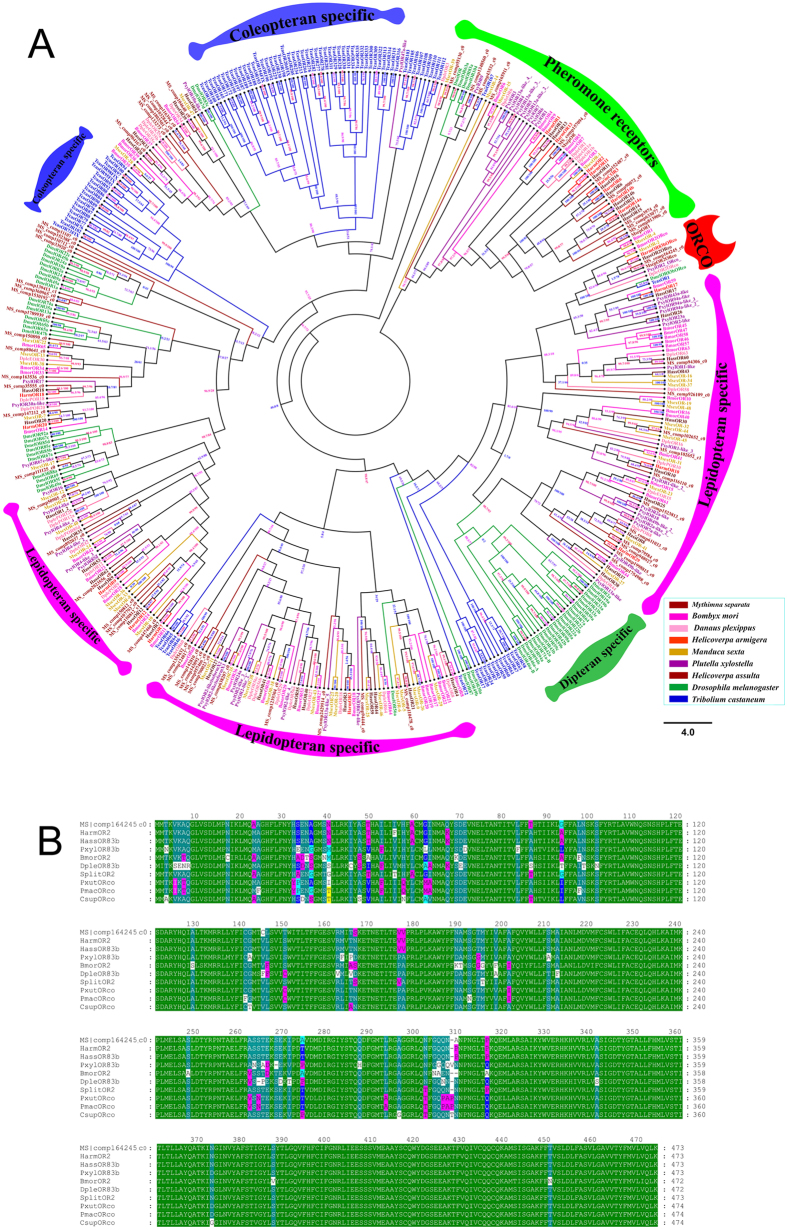
Phylogenetic tree of odorant receptors (ORs) and Alignment of the Odorant receptor co-receptor (Orco). Phylogenetic tree of the ORs (**A**). ORs from Lepidopteran, Coleopteran, Dipteran. The specific clades are marked. Node support was assessed with 1000 bootstrap replicates. Alignment of the Orco (**B**). Orco proteins from *M. separata* (MS), *H. armigera* (Harm), *H. assulta* (Hass), *B. mori* (Bmor), *D. plexippus* (Dple), *P. xylostella* (pxyl), *S. litura* (Split), *P. xuthus* (Pxut), *P. machaon* (Pmac) and *C. suppressalis* (Csup).

Sixty candidate OR proteins were identified from the data sets. Supplementary Data S5 summarized transcript name, length, Signal Peptide, TMD Number and FPKM in three biological replicates of the OR genes. The average sequence length was 327 bp. Four ORs likely represent full-length sequences. The highest FPKM value is 267.0 (MS|comp165911_c0) and the average FPKM value of the ORs was just 7.9. Most of partial length transcripts likely represent separate individual protein, as overlapping regions among them showed low identity of amino acid sequence. The amino acid identity of full length putative *M. separata* ORs ranged from 11.2% to 21.6% (average 17.5%), which was consistent with the diversity of the OR gene family (Supplementary Data S4). According to the analysis of Predictive software, there might be zero to seven transmembrane domains represent in full-length candidate *M. separata* OR transcripts. Consistent with the length of the partial transcripts, the remaining *M. separata* ORs may posse between zero and three transmembrane domains. It was worth noting that the highly-conserved co-receptor Orco which showed 87% to 97% amino acid sequence identity with Orco from *B. mori, P. xylostella, D. plexippus* and other Lepidopterans (Fig. 4 and Supplementary Data S5). A phylogenetic tree of ORs based on the maximum likelihood method was constructed using protein sequences of ORs from *M. separata*, six other lepidopteran species, *T. castaneum* and *D. melanogaster* (Fig. 4). ORs are divided into several subgroups of various size and content in the phylogenetic tree, the odorant co-receptor (Orco) and pheromone receptor (PR) families were highly conserved. Interestingly, MS|comp50905_c0 sharing high homology and closely clustered with MsexOR42, which detects cis-3-hexenyl acetate thereby affect oviposition site location^10^.

### Identification of candidate gustatory receptors

**Figure 5:**
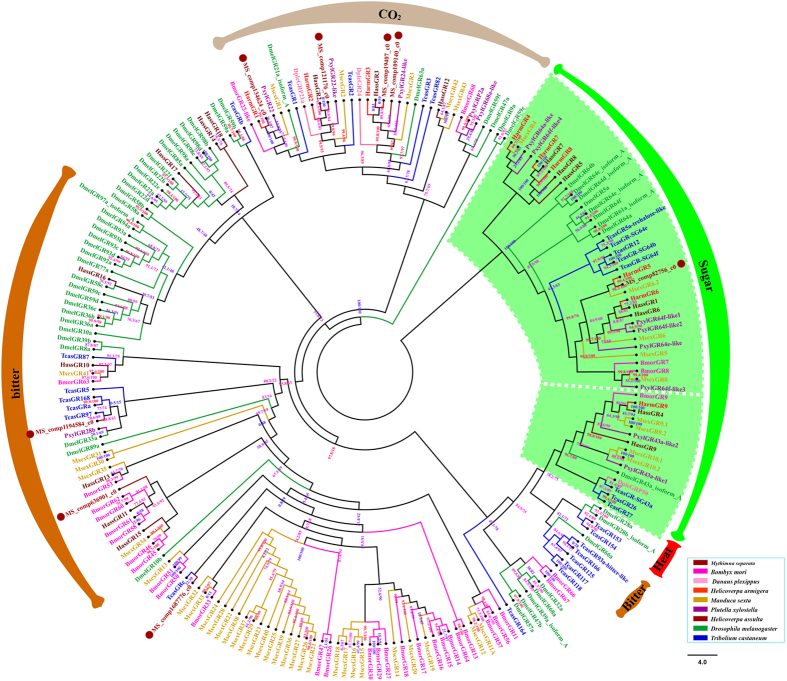
Phylogenetic tree of gustatory receptors (GRs). GRs from Lepidopteran, Coleopteran, Dipteran. The specific clades are marked. Node support was assessed with 1000 bootstrap replicates.

Eight candidate gustatory receptors (GRs) transcripts with very low expression levels (Transcript abundance levels ranged from 0.46 to 1.94 FPKM) were identified (Supplementary Data S3). Only one candidate *M. separata* GRs represent full-length protein and most of them represent partial length fragments. They encoded overlapping but distinct sequences, which established the proteins as components of independent genes. As expected with other insect GRs, transmembrane domain and topology predictions indicated that full-length transcripts most likely possess between zero and two domains with an intracellular N-terminus and extracellular C-terminus (Supplementary Data S5). A phylogenetic tree was constructed using *M. separata* GRs (8), *B. mori* GRs (33), *P. xylostella* GRs (14), *D. plexippus* GRs (3), *H. armigera* GRs (9), *H. assulta* GRs (18), *M. sexta* GRs (45), *T. castaneum* GRs (57) and *D. melanogaster* GRs (27) (Fig. 5). All candidate *M. separata* GRs cluster with CO2, sugar, and putative bitter receptor families.

### Identification of candidate ionotropic receptors

**Figure 6:**
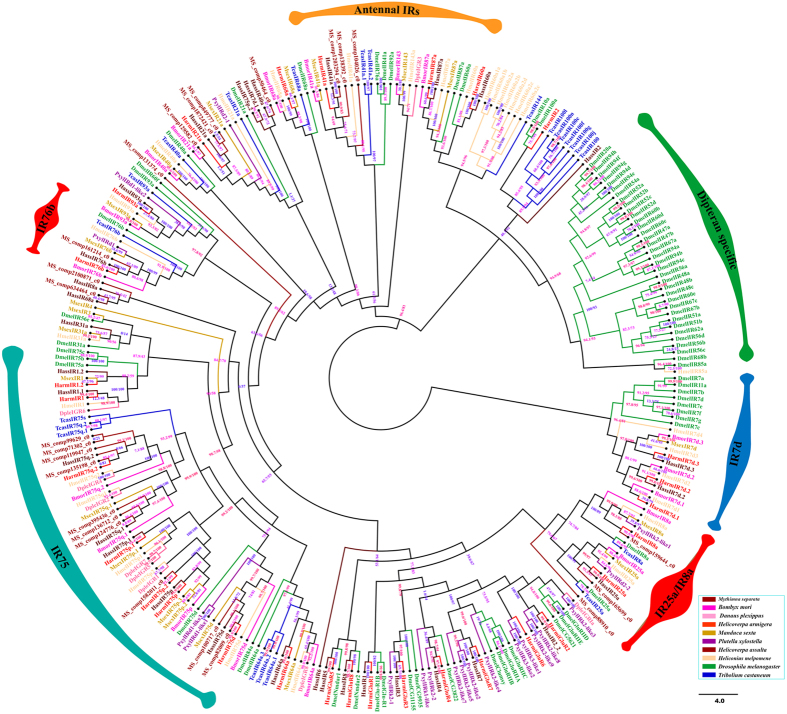
Phylogenetic tree of ionotropic receptors (IRs). IRs from Lepidopteran, Coleopteran, Dipteran. The specific clades are marked. Node support was assessed with 1000 bootstrap replicates.

Twenty-four candidate ionotropic receptors were identified with very low expression levels in the head (Supplementary Data S5). However, Only MS|comp165699_c0, MS|comp135198_c0, MS|comp82009_c0 have relatively highly expressed (FPKM: 21, 27, 15, respectively.) (Supplementary Data S5). Three IRs likely represent full-length sequences and ware longer than 1600 bp in general. The most conserved sequence exhibited in three transmembrane domains and the ion channel pore (Fig. S5)^11^,^12^ and characteristic variability of the glutamate-binding residues in the ligand-binding S1 and S2 domains (Fig. S5). These Twenty-four IRs together with seven lepidopteran species, *T. castaneum* and *D. melanogaster* were used for a phylogenetic analysis (Fig. 6). The conserved “antennal IRs”, three highly conserved co-receptors (IR76b, IR8a and IR25a) and the species-specific “divergent IRs” were present here as well as the large sub-families of IR75 clades.

### Identification of candidate sensory neuron membrane proteins

**Figure 7:**
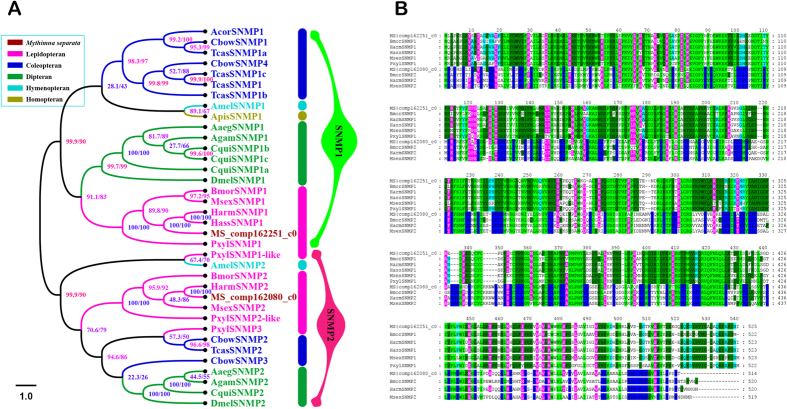
Sensory neuron membrane proteins. Phylogenetic tree of the SNMPs (**A**). SNMPs from Lepidopteran, Coleopteran, Dipteran, Hymenoptern and Homopteran. The specific clades are marked. Node support was assessed with 1000 bootstrap replicates. Alignment of the SNMPs (**B**). SNMPs from *M. separata* (MS), *H. armigera* (Harm), *H. assulta* (Hass), *B. mori* (Bmor), *P. xylostella* (Pxyl), *M. sexta* (Msex).

Two candidate SNMPs (MS|comp162251_c0 and MS|comp162080_c0) were identified in the transcriptomes. Both likely represent full-length genes (exceeded 1,000 bp in size; Supplementary Data S5). The FPKM results showed that MS|comp162080_c0 displayed a higher expression level (FPKM: 74.5) to MS|comp162251_c0(FPKM: 18.8) (Supplementary Data S3). The two candidate SNMPs were very similar to the HarmSNMP1 and HarmSNMP1 published in GenBank, with 89.3% and 85.6% identity, respectively. In addition, which shared more than 60% (73.0% and 625.6%) identity with the corresponding SNMPs in *B. mori* (Supplementary Data S4). We also detected a mean amino acid sequence identity among *M. separata* SNMPs of 28.9%. Phylogenetic analysis showed that MS|comp162251_c0 clustered with the insect SNMP1 group, and MS|comp162080_c0 clustered with the insect SNMP2 group. They belonged to Lepidopteran clade in the phylogenetic tree, respectively (Fig. 7).

## Discussion

Through the analysis of head transcriptome from larvae, pupae and adults of *Mythimna separata*, the major opsins and chemosensory genes involved in sensory mechanism were first reported in the research, which further enriched the molecular biological fundament of armyworm sensory. As sensory systems played significant roles in *M. separata* behavior, the identified genes could be potential novel targets for future pest control methods.

The GO assignment results of classification of predicted functions in *M. Separata* were consistent with that of other invertebrates^13^,^14^,^15^,^16^,^17^,^18^,^19^. As regard to the number of the identified sensory genes, there was no statistical difference among *M. Separata* and other Lepidopteran, Dipteran, and Coleopteran species with identified transcriptome^11^,^17^,^18^. Without considering the potential significance of individual genes to each species studied, the similarity of the different data sets does indicate a certain level of sensory gene conservation with respect to their expression.

Vision deeply involved in the regulatory mechanism of insect behaviors, such as food and mates searching, predators avoiding^12^,^20^,^21^. Thousands of ommatidia composed Compound eyes and the later converted lights into visual images^22^. The compound eyes were one of the most important parts of *M. separata*’s head^23^. Opsins played significant roles in molecular detection of photons and the transduction of visual images deeply depended on amino acid sequences of opsins^24^. The evolution of opsin genes provided a solid molecular basis for color vision adaption^25^. The three major subtypes of opsins were UV, blue and LW opsin group and It has been reported that LW opsin group had the most variety: the nymphalid butterfly *Heliconius erato* uses only one LW opsin to discriminate colors in wide LW ranges with the help of filtering pigments^26^; *Helicoverpa armigera* owns two LW opsins^27^ and *Papilio glaucus* four LW opsins^8^. We also found two LW opsins in *M. Separata.* In accordance with the phylogenetic analysis, the two candidate LWs were very similar to the Harmopsin_LW1 and Harmopsin_LW2 published in GenBank, with 97.9% and 93.1% identity, respectively. And the homology was 85.9% between LW opsins in *M. separata* (Supplementary Data S4). The second LW opsin was classified as a member of the LW2 gene family of lepidopteran species^28^, which indicated that LW duplication also occurred in *M. separata*. In addition, LW1 (MS|comp149511_c0) of *M. Separata* was found to be the most abundant opsin genes (FPKM: 5344.8), and more than 170 times higher expression to LW2 opsin (MS|comp158503_c0) (FPKM: 30.3), this is close to the reported in *H. armigera*^29^. It is possible that LW opsin is important to nocturnal insects because the long-wavelength light is the strongest at night in three different wavelength lights, Thus, the elevated expression of LW opsin might be associated with the nocturnal activities of insects^27^. Amelpteropsin expression in the brain was previously observed in honeybees^30^. These results indicated that opsin genes might mediate not only visual function but also nonvisual function. Interestingly, (MS|comp165164_c0) clustered with the pteropsin group in the phylogenetic tree (Fig. 2). Nonvisual function of opsin has been reported, such as thermosensation, photoperiodic responses, long-distance migration, mechanotransduction of auditory organ and egg-laying^31^,^32^,^33^,^34^,^35^,^36^.

CSPs contain a signal peptide and four conserved cysteines that are capable of forming two disulfide bridges to stabilize the tertiary structure^37^,^38^. which have soluble nature, flexible polypeptide folding, compact structure and a smaller molecular weight (10–16 kDa), permit them to bind a variety of ligands and therefore could undertake several tasks in the biological process^39^. CSPs were highly and almost ubiquitously distributed in chemosensory tissues as well as in non-chemosensory tissues, suggesting that CSPs in insects may also participate in other functions in addition to chemosensation, such as limb regeneration, female survival and reproduction, embryo development, recognition of sex pheromone, sucking and migratory behavioral^40^,^41^,^42^,^43^,^44^,^45^,^46^. Fortunately, MS|comp154133_c0 sharing 66.9% homology and closely clustered with BmorCSP1, and MS|comp149520_c0 sharing 65.8% homology and closely clustered with BmorCSP2. The two most abundant CSPs have different sex-specific expression patterns in antennae of *Bombyx mori*^47^. HarmCSP6, sharing 84.4% homology and closely clustered with MS|comp149356_c0, was reported to be highly transcribed in pheromone glands and display high binding affinity for pheromone components^48^. MS|comp158640_c0 and MS|comp149055_c0 shared 58.9% and 89.7% identity with HarmCSP4 which was detected to be exclusively present in proboscis and could help solubilizing terpenoids present in flower nectar^45^. We identified 22 CSPs in the *M. separata* transcriptome, comparing with the number of CSPs identified from other Lepidoptera: *B. mori* (24)^37^, *Danaus plexippus* (30), *H. armigera* (19)^49^, *Manduca sexta* (21)^50^, *Sesamia inferens* (24)^51^, we may have missed some CSPs in this transcriptome.

The OBP is soluble protein with a molecular weight of 12–20 kDa, ferrying the hydrophobic semiochemicals across the sensilla lymph to olfactory receptors, and has a signal peptide sequence of about 20 amino acids at the N-terminus^52^,^53^,^54^. Insect OBPs can be classified into classical OBPs (six-cysteine conserved signature) and Minus-C (missing C2 and C5) and Plus-C (carry additional conserved cysteine located between C1 and C2 and after C6). In addition, the classical insect OBPs include GOBP/PBP, CRLBP, ABPI and ABPII^53^,^55^. A phylogenetic tree based on the maximum likelihood method and used the amino acid sequences of OBPs from 9 species, show that the 50 OBPs of *M. separata* belong to six insect OBP subfamilies, respectively (Fig. 4). OBPs appear in olfactory and gustatory system and have reported olfactory and gustatory functionally characterized in Recent studies^56^,^57^,^58^,^59^. Interestingly, MS|comp158921_c0 closely clustered with the *Drosophila melanogaster* OBP49a is indispensable for the suppression of sweet taste by bitter chemicals^60^. MS|comp154792_c0, MS|comp148423_c0, MS|comp131182_c0 closely clustered with the DmelOBP57e and DmelOBP57d which are not only involved in taste perception but could also change the behavioral response to the host odors^61^. In Lepidoptera, two subfamilies of GOBPs and PBPs, are responsible for recognizing and transporting host odorants and pheromones, respectively^62^,^63^. GOBPs are a subfamily of OBPs, consisting of two members, GOBP1 and GOBP2 in most Lepidopterans. MS|comp161014_c0 and MS|comp114857_c0 closely clustered with the GOBP1, and MS|comp156324_c0 closely clustered with the GOBP2. PBPs are a subfamily of OBPs and constituted of three members in Lepidopteran, and we found MS|comp124490_c0, MS|comp162075_c0 and MS|comp154997_c0 closely clustered with the lepidopteran specific PBP family in the phylogenetic tree (Fig. 4). While the PBP1 of *B.mori* is capable of enhancing sensitivity and selectively mediating the response to bombykol^64^. The PBP1 of *H. armigera* binds strongly with two principal pheromone components (Z)-11-tetradecenal and (Z)-9-hexadecenal, seems that HarmPBP1 plays a key role in sex pheromone recognition^65^.

Two ORs are required in order to transduce odor-evoked signals, a highly-conserved co-receptor (Orco) and a specific OR, which varies according to ORN type^66^,^67^,^68^,^69^. The number of OR genes varies from 10 to 350^70^,^71^. Insect ORs was determined that ORs display a high degree of divergence, both within and across species due to gene duplications and deletion events^72^. These variations represent the olfaction sensing ability of insects with high level odor detection in insects harboring more odor-specific subunits. The 60 sequences numbers are comparable to the reported numbers in *H. armigera*^49^, *M. sexta*^50^, *Cydia pomonella*^73^ and *Athetis dissimilis*^74^. ORs in moths contain pheromone receptors (PRs) detecting sex pheromone and non-PR ORs, which respond to a variety of volatile chemicals, including plant- and microbe-derived compounds^75^,^76^,^77^.

In the phylogenetic tree of ORs, the specific Orco lineage contained MS|comp164245_c0, which shows that it has high similarity with six lepidopteran Orco, TcasOrco and DmelOrco, and that MS|comp164245_c0 could be the Orco of *M. separata*. The female *M. separata* moths emit the main component of the sex pheromone, Z11–16:OAc^78^. Lepidoptera sex pheromones produced by females may attract males for mating opportunities^79^,^80^,^81^. Based on phylogenetic tree analyzes, six *M. separata* ORs (MS|comp813906_c0, MS|comp533077_c0, MS|comp3974_c0, MS|comp90072_c0, MS|comp152487_c0 and MS|comp157094_c0) clustered in a conserved clade of PRs found in Lepidopteran insects (Fig. 4). We, therefore, hypothesize that some or all of them appear to be dedicated to sex pheromone detection. In addition, MS|comp1239304_c0 clustered with MsexOR18 and BmorOR29 which responds to linalool, citral and linalyl acetate^82^. BmorOR-24, which is broadly tuned and detects^82^, has three orthologues in *M. separata*, which could detect similar ligands. MS|comp892037_c0 clustered with MsexOR24 and BmorOR42 which responds to linalool and linalyl acetate^82^. The heterologous expression system has established to further investigate the functional characteristics of *M. separata* ORs.

Only eight GR-encoding transcripts were identified from the head transcriptome, Due to gustatory sensory neurons are primarily found in chemosensory sensilla on antennal, head tissues, leg tarsi and ovipositors and the low expression level of GR^83^,^84^. GRs play an important role in the detection of taste chemicals and ultimately influence an insect’s decisions about food, mates and egg deposition sites^52^,^85^. The GR family of insect includes receptors for sugars and bitter compounds, as well as cuticular hydrocarbons and odorants (CO_2_). GRs perceive essential nutrients whose chemical structures remain constant such as sugars and CO_2_ receptors. Thus, sugar and CO_2_ receptor genes are relatively highly conserved in most of the insect genomes that have been sequenced to date^11^,^86^,^87^,^88^. Based upon phylogeny, MS_comp121176_c0, MS_comp134624_c0, MS_comp109140_c0, MS_comp19407_c0 in *M. separata* grouped together with TcasGR1, TcasGR2, TcasGR3, DmelGR63a and DmelGR21a, which function as CO_2_ receptors, MS_comp82756_c0 grouped together with DmelGR5a, DmelGR61a, TcasGR64 and DmelGR64, which function as sugar receptors, MS_comp1194584_c0 and MS_comp636901_c0 grouped together with DmelGR10a, DmelGR33a and BmorGR60, which function as bitter receptors, none of “GR43-like” receptors were identified. So far, insect GRs have been identified as sugar receptors in *B. mori*^89^, *H. armigera*^90^ and *D. melanogaster*^85^. As CO_2,_ fructose and bitter receptors in *D. melanogaster*^91^,^92^. However, putative bitter GRs have not been functionally characterized in moths.

In insects, IRs are a conserved family of synaptic ligand-gated ion channels that evolved from ionotropic glutamate receptors (iGluRs) and includes the conserved “antennal IRs” having an olfactory function, and the species-specific “divergent IRs” having gustatory function^93^,^94^. Among “antennal IRs”, one or two of the broadly expressed coreceptors (IR8a, IR25, and IR76b) in one IR-expressing neuron^95^. IRs belong to an ancient chemoreceptor family, and most of the IRs in Drosophila have clear orthologs with genus and Lepidoptera^49^,^50^,^51^,^96^. In this research, 24 candidate IRs with very low expression were identified in the *M. separata* head transcriptome, which is similar to observations in *Plutella xylostella* (19), *H. armigera* (29) and *M. sexta* (21)^97^. Some IRs have been functionally characterized. i.e. IR co-receptors respond to odorant stimulation^94^, IR40a, which detects DEET and is a target of insect repellents^98^, IR64a, which is involved in acid detection^99^, IR94b involved in auditory sense^36^ and IR76b involved in low-salt tasting^100^. But moth IRs have not been functionally investigated. The three candidate co-receptors: MS|comp159644_c0, MS|comp165699_c0 and MS|comp161214_c0. They shared 49%, 67% and 42% amino acid identities with *D. melanogaster* IR8a, IR25a and IR76b, respectively, and showed a higher amino acid identity of over 75% with the co-receptors of other lepidopteran species (*B. mori, P. xylostella, D. plexippus, Heliconius melpomene, H. armigera, Helicoverpa assulta* and *M. sexta*). The phylogenetic analysis proved that the large sub-families of IR75 clades contain ten candidate IRs of *M. separata* while the large sub-families of IR7d contain none (Fig. 6). The relationship between the evolution of these novel receptors and the ecology of the species require further research which will ultimately reveal the manipulation mechanism of this novel receptor family.

SNMPs are members of the CD36 family of proteins and associated with pheromone-sensitive neurons in Lepidoptera and Diptera^101^,^102^. The insect SNMP family consists of two subfamilies, SNMP1 and SNMP2, which were first identified from *Antheraea polyphemus*^103^ and *M. sexta*^63^, respectively. Both are expressed in the sensillum trichodeum, but they differ in location and level of expression. Currently, the general mechanism of insect SNMP function is still poorly understood. In D. melanogaster, SNMP1 is necessary for proper OSN responses to the pheromone compound, cis-vaccenyl acetate^104^. Lepidopteran SNMPs contain two conserved groups of SNMP1 and SNMP2^101^,^105^. While more than two SNMPs has been reported in coleopteran, lepidopteran and dipteran species^11^,^96^,^106^,^107^. In the moth, SNMP1 was primarily expressed in antennae and SNMP2 was abundant expressed in antennae as well as in legs^18^. In the phylogenetic tree, all SNMPs from Lepidoptera, Coleoptera, Diptera, Hymenoptera and Homoptera clustered into two clades, SNMP1 and SNMP2. Two candidate SNMPs of *M. separata* (MS|comp162251_c0 and MS|comp162080_c0) were belong to Lepidopteran sub-clades in each clade, respectively (Fig. 7). The large diversity of SNMP1 and 2 proteins within insect orders suggests that they contribute to the specificity of odour recognition^52^.

## Conclusion

The armyworm *Mythimna separata* is a specialist insect that feeds mainly on maize, sorghum and rice, causing large economic losses. We first obtained abundant biology information on the transcriptome of *M. separata* head using high-throughput sequencing technology with the aim of identifying of the genes potentially involved in the sensory process. A total of 174 transcripts encoding putative sensory proteins from the seven major opsins and olfactory gene families were annotated: 8 opsins, 22 CSPs, 50 OBPs, 60 ORs, 8 GRs, 24 IRs, and 2 SNMPs. Comparative analysis with other Lepidopteran species suggests that near complete information regarding the molecular basis of *M. separata* perception was obtained. As the first step towards understanding gene functions, we conducted a comprehensive and comparative phylogenetic analysis. Several genes were clustered with functionally validated genes from other insects, such as Co-receptors of OR and IR, PBPs, PRs, CO2 GRs, bitter GRs and sweet GRs. Our findings made it possible for future research on the molecular level of olfactory system of *M. separata*, and in particular, the discovery of receptor genes will also contribute to the identification of novel volatile host compounds, which would gain novel targets for the pest management with semiochemicals.

## Methods

### Insects and sampling

Lab species of *Mythimna separata* was reared under controlled conditions: 25 ± 1 °C, 70~80% RH, and photoperiod of 14 L: 10D. Heads of *M. separata* were harvested from 1–6th instar larvae, 1–10 day-old pupae (half male and female), and 0–5 day-old adults (half male and female), respectively. Three repetitions were conducted. Detailed composition of mixed heads was provided in the Table S1.

### RNA preparation and quality control

Total RNA was extracted from mixed heads. NanoPhotometer^®^ spectro-photometer (IMPLEN, CA, USA) and Nano6000 Assay Kit of the Agilent Bioanalyzer 2100 system (Agilent Technologies, CA, USA) were applied for checking the purity and integrity of total RNA, respectively. Qualified mRNA was purified from total RNA using poly-Toligo-attached magnetic beads.

### Library preparation and sequencing

Sequencing libraries were generated using NEBNext^®^ Ultra ™ RNA Library Prep Kit for Illumina^®^ (NEB, USA) following manufacturer’s recommendations and index codes were added to attribute sequences to each sample. Library quality was assessed on the Agilent Bioanalyzer 2100 system. The clustering of the index-coded samples was performed on a cBot Cluster Generation System using TruSeq PE Cluster Kit v3-cBot-HS (Illumia) according to the manufacturer’s instructions. After cluster generation, the library preparations were sequenced on an Illumina Hiseq2000 platform and 100 paired-end reads were generated.

### Transcriptome assembly

Transcriptome assembly was accomplished based on the left.fq and right.fq using Trinity with min_kmer_cov set to 2 by default and all other parameters set default^108^,^109^.

### Gene functional annotation

Gene function was annotated based on the following databases: Nr (NCBI non-redundant protein sequences); Nt (NCBI non-redundant nucleotide sequences); Pfam (Protein family); KOG/COG (Clusters of Orthologous Groups of proteins); Swiss-Prot (A manually annotated and reviewed protein sequence database); KO (KEGG Ortholog database); GO (Gene Ontology).

### Sequence alignment and phylogenetic analysis

The signal peptides were predicted using SignalP 4.1 (http://www.cbs.dtu.dk/services/SignalP/)^110^. The Trans-Membrane Domains(TMDs) were predicted using TMHMM 2.0 (http://www.cbs.dtu.dk/services/TMHMM)^111^. Amino acid sequence alignment was performed using the ClustalW method implemented in the Mega v7.0 and visualized with genedoc^112^,^113^.

The M. separata OPSIN, OBP, CSP, OR, SNMP, GR and IR nucleotide sequences were used as queries (BLASTx) to the GenBank database, and sequences from different insect species and their amino acids were retrieved and used to construct a phylogenetic tree. Amino acid sequences were aligned using the Muscle method implemented in the Mega v7.0^113^. The resulting alignment was manually curated to remove gap-rich regions. Maximum-likelihood trees (for OPSIN, OBP, CSP, OR, SNMP, GR and IR) were constructed using IQ-TREE with the best-fitting substitution-model^114^ and subsequently viewed and graphically edited in FigTree v1.4.3^13^ and Adobe Illustrator. Branch support was assessed using the bootstrap method based on 1000 replicates.

## Additional Information

**How to cite this article**: Liu, Z. *et al*. Sensory genes identification with head transcriptome of the migratory armyworm, *Mythimna separata. Sci. Rep.* **7**, 46033; doi: 10.1038/srep46033 (2017).

**Publisher's note:** Springer Nature remains neutral with regard to jurisdictional claims in published maps and institutional affiliations.

## Supplementary Material

Supplementary Information

Supplementary Data S1

Supplementary Data S2

Supplementary Data S3

Supplementary Data S4

Supplementary Data S5

## Figures and Tables

**Table 1:** Detailed information on the OBP ungenes of *Mythimna separata*.

Unigene ID	Unigene Length (bp)	ORF Length (aa)	Complete ORF	5′ or 3′ Terminus Lost	Signal Peptide	Signal Peptide (aa)	Cysteine Number	FPKM (mean)	FPKM1	FPKM2	FPKM3
MS|comp116343_c0	605	146	YES	—	YES	21	8	3092.00	3070.29	1416.29	4789.41
MS|comp118070_c0	500	148	YES	—	YES	19	6	1.86	1.35	0.00	4.22
MS|comp129850_c0	759	145	YES	—	YES	21	7	12.64	15.43	11.03	11.45
MS|comp141177_c0	651	144	YES	—	YES	23	8	425.24	482.35	341.87	451.49
MS|comp141270_c0	638	137	YES	—	YES	20	6	4073.95	3613.61	2183.50	6424.75
MS|comp141612_c0	743	153	YES	—	YES	17	5	191.25	219.91	105.38	248.47
MS|comp141702_c0	628	139	YES	—	YES	21	9	42.02	58.06	29.15	38.85
MS|comp142589_c0	645	154	YES	—	YES	23	10	10.64	13.69	4.42	13.82
MS|comp148301_c0	565	139	YES	—	YES	18	7	127.82	118.57	113.56	151.34
MS|comp150111_c0	556	142	YES	—	YES	21	7	278.95	313.43	270.81	252.62
MS|comp150160_c0	711	168	YES	—	YES	20	7	348.76	332.15	247.67	466.46
MS|comp150353_c0	673	153	YES	—	YES	21	6	4.09	9.30	1.86	1.10
MS|comp152336_c0	630	164	YES	—	NO	—	9	25.05	27.53	21.22	26.40
MS|comp152359_c0	1482	149	YES	—	YES	21	7	119.11	113.53	84.42	159.38
MS|comp152974_c0	646	147	YES	—	YES	15	8	13.53	16.61	11.15	12.83
MS|comp153447_c0	662	148	YES	—	YES	21	7	760.80	798.02	762.58	721.81
MS|comp156324_c0	669	162	YES	—	YES	21	7	1929.52	1859.69	1742.96	2185.91
MS|comp156380_c0	827	136	YES	—	NO	—	7	10.89	10.72	12.70	9.25
MS|comp156923_c0	675	146	YES	—	YES	16	10	47.20	49.32	17.95	74.33
MS|comp158230_c0	1253	252	YES	—	YES	19	3	78.99	115.66	66.80	54.51
MS|comp158329_c0	815	133	YES	—	YES	16	5	587.29	704.26	393.22	664.38
MS|comp158921_c0	677	183	YES	—	YES	17	16	248.53	294.46	143.34	307.79
MS|comp159796_c1	613	165	YES	—	YES	16	12	437.41	400.40	328.19	583.65
MS|comp160581_c0	794	197	YES	—	YES	17	13	46.84	59.44	38.85	42.24
MS|comp161605_c0	953	154	YES	—	NO	—	6	2638.01	2474.12	1167.94	4271.96
MS|comp162075_c0	1055	170	YES	—	YES	27	7	1919.57	1612.91	1276.67	2869.14
MS|comp163093_c1	3862	515	YES	—	NO	—	10	268.11	172.50	439.16	192.67
MS|comp167844_c0	609	146	YES	—	YES	23	8	299.55	314.18	148.05	436.42
MS|comp173090_c0	648	149	YES	—	YES	22	8	68.25	74.26	29.43	101.07
MS|comp60723_c0	587	141	YES	—	YES	18	8	1436.46	1557.41	1258.45	1493.51
MS|comp108141_c0	440	138	NO	3′	YES	22	7	5.16	4.32	0.00	11.16
MS|comp114857_c0	245	68	NO	3′	YES	17	2	694.08	649.36	839.41	593.47
MS|comp121812_c0	275	90	NO	3′	NO	—	3	2.48	5.45	0.00	1.99
MS|comp124490_c0	219	72	NO	5′and 3′	YES	17	3	3.69	5.95	2.84	2.29
MS|comp131182_c0	345	96	NO	5′and 3′	NO	—	3	4.58	0.00	10.38	3.36
MS|comp133839_c0	470	128	NO	5′	NO	—	5	2.65	3.87	2.51	1.56
MS|comp133858_c0	254	82	NO	5′	NO	—	5	1.71	0.00	0.00	5.13
MS|comp141404_c0	436	137	NO	3′	YES	25	8	119.67	122.50	121.69	114.82
MS|comp143661_c0	563	145	NO	5′	YES	19	8	1.49	1.57	2.21	0.70
MS|comp145270_c0	370	117	NO	3′	YES	17	7	3.31	5.92	0.64	3.38
MS|comp145826_c0	410	129	NO	3′	YES	22	6	1.91	1.88	2.65	1.19
MS|comp154997_c0	532	165	NO	3′	YES	23	8	1115.64	1056.35	1259.04	1031.54
MS|comp158322_c0	1517	20	NO	5′	NO	—	0	454.46	514.82	529.24	319.32
MS|comp161014_c0	1133	91	NO	5′′	NO	—	4	348.36	381.04	382.50	281.53
MS|comp161583_c1	487	60	NO	5′	NO	—	2	3.01	2.53	4.75	1.76
MS|comp393373_c0	444	77	NO	5′	NO	—	3	1.26	0.33	1.38	2.06
MS|comp427468_c0	210	64	NO	3′	YES	21	2	10.98	9.54	20.62	2.79
MS|comp502645_c0	284	92	NO	3′	YES	16	3	1.22	2.49	1.18	0.00
MS|comp672758_c0	297	75	NO	5′	NO	—	3	2.04	3.70	0.00	2.41
MS|comp71248_c0	233	43	NO	5′	NO	—	3	4.84	4.65	9.86	0.00
