# Supplementary material for: Sensory genes identification with head transcriptome of the migratory armyworm, Mythimna separata
Source: Sci Rep. 2017 Apr 7;7:46033. doi: 10.1038/srep46033 (PMC5384095; doi:10.1038/srep46033)
Supplement: Supplementary Information [file srep46033-s1.pdf]

Sensory genes identification with head transcriptome of the migratory armyworm, *Mythimna separata*

Zhenxing Liu, Xiaoyun Wang, Chaoliang Lei, Fen Zhu

Supplementary of Contents

**Title Page**	pg. 1
**Figure S1**	pg. 2
**Figure S2**	pg. 3
**Figure S3**	pg. 3
**Figure S4**	pg. 4
**Figure S5**	pg. 5
**Table S1**	pg. 6
**Supplementary Data S6**	pg. 7-27
**Supplementary Data S7**	pg. 28-275
**Supplementary Data S1**	*
**Supplementary Data S2**	*
**Supplementary Data S3**	*
**Supplementary Data S4**	*
**Supplementary Data S5**	*

* submitted as separate excel file.

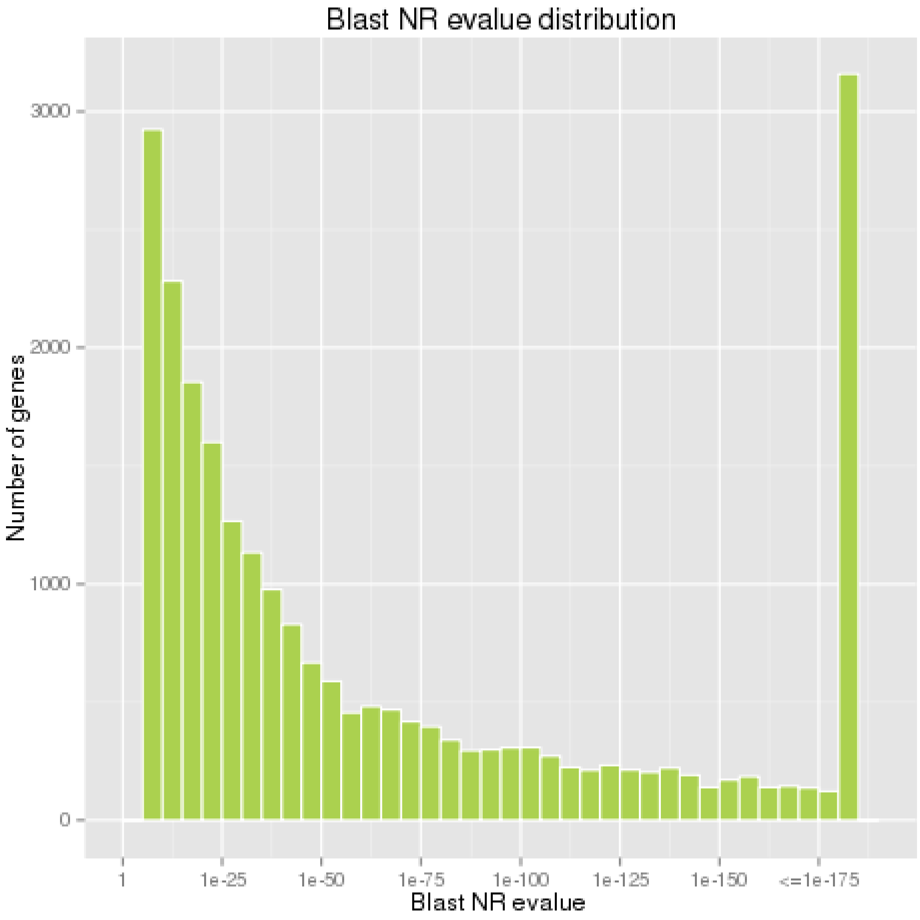

Figure S1. Blast NR evalue distribution of the *M. separata* transcriptome.

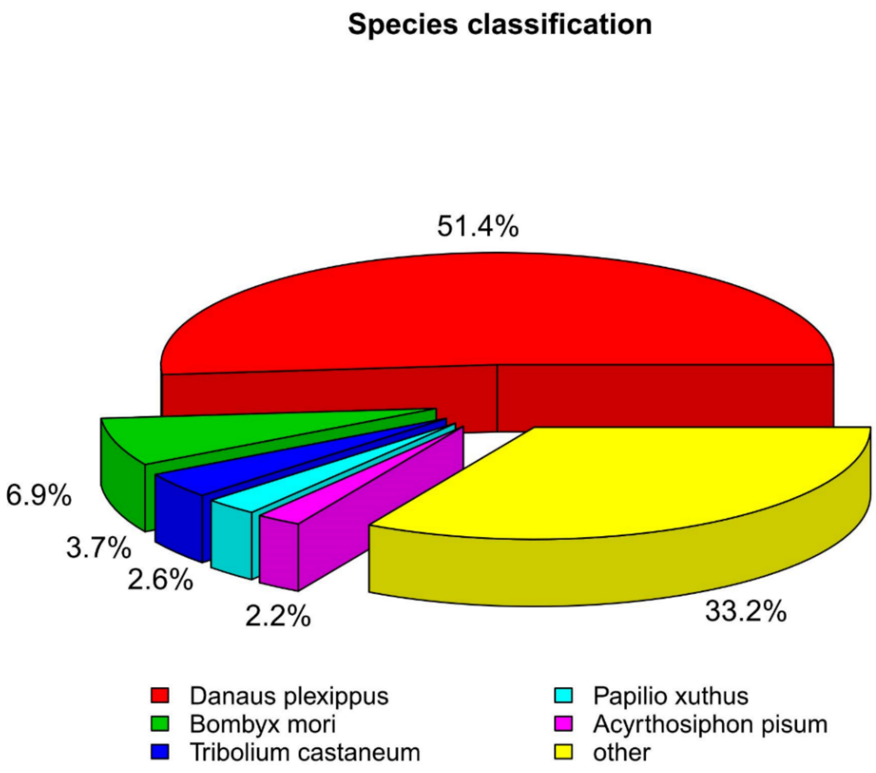

Figure S2. The BLASTx annotations of *M. separata* transcriptome species distribution.

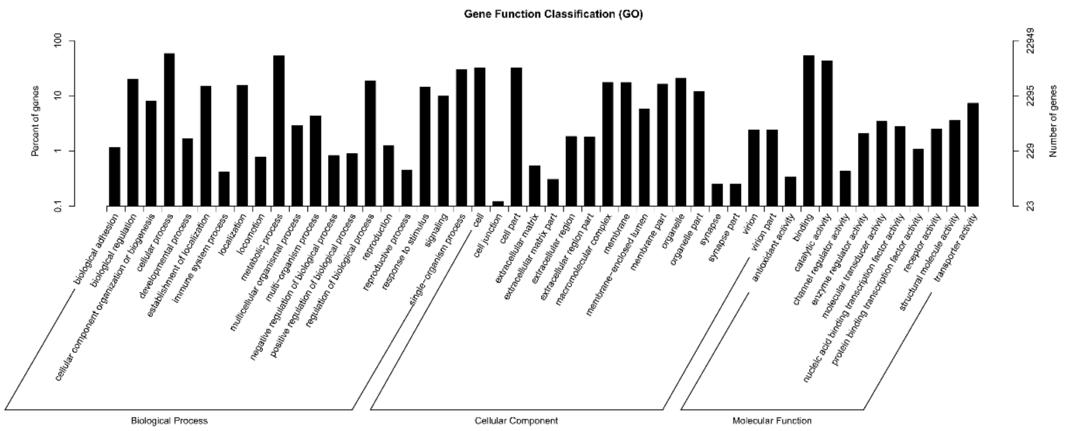

Figure S3. Gene Ontology (GO) results. GO analysis of 22,949 genes in *M. separata*, according to their involvement in biological processes, molecular function, or role as a cellular component.

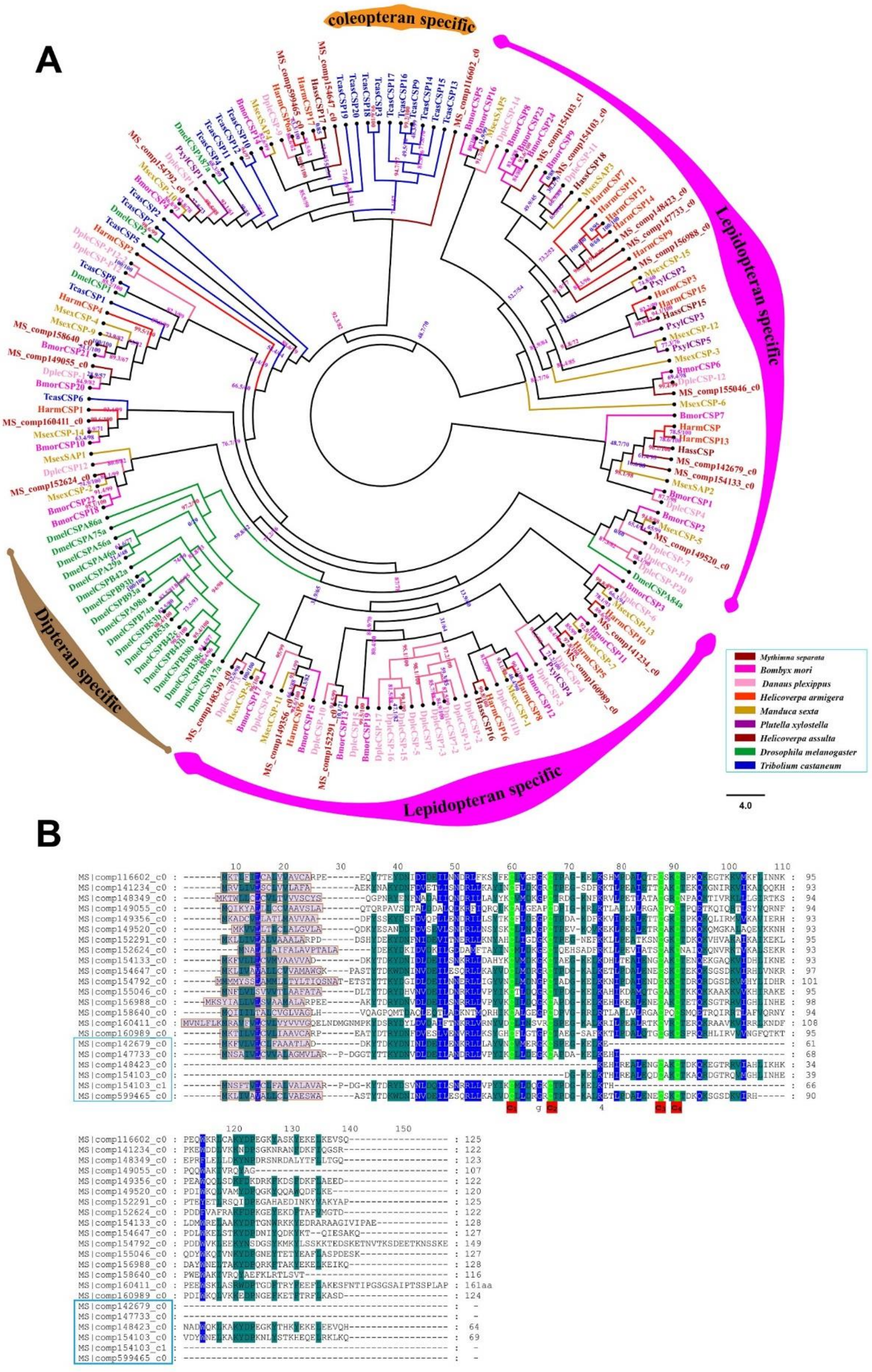

Figure S4. ML CSPs and Alignment of the CSP sequences of *M. separata*.

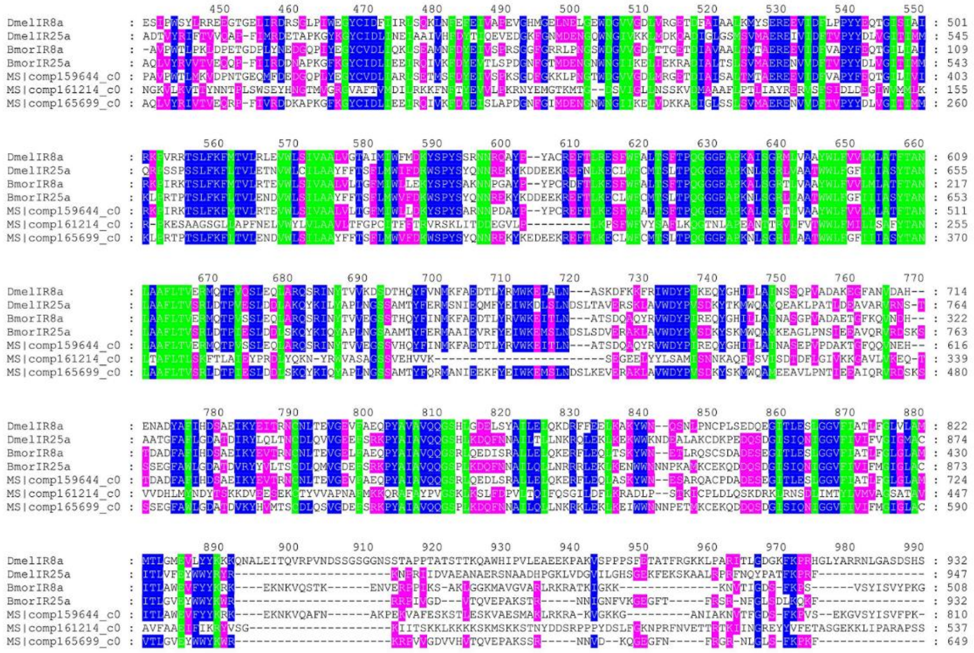

Figure S5. Alignment of the IR sequences of *M. separate*.

Table S1. Component of mixed heads from *M. separata* in different life stages

**Stages**	**Duration (d)**	**Frequency**	**Sample-day counts**	**12:00 AM**	**12:00 PM**	**Number of heads**
1st instar larva	6	Every 2 days	2	5	5	30 *
2nd instar larva	3	Every 2 days	2	6	6	24 *
3rd instar larva	3	Every 2 days	2	6	6	24 *
4th instar larva	3	Every 2 days	2	5	5	20 *
5th instar larva	3	Every 2 days	2	4	4	16 *
6th instar larva	6	Every 2 days	3	2	2	12 *
Female pupa	10	Every 3 days	3	2	2	12 *
Male pupa	10	Every 3 days	3	2	2	12 *
Female adult	5	Every 2 days	3	2	2	12 *
Male adult	5	Every 2 days	6	2	2	12 *
Total	-	-	-	-	-	174 *

* indicates Sampling procedure in the table were repeated for three times.

Supplementary Data S6. The Sensory Protein Fasta Sequences of *M. separate*.

1. Opsin protein fasta sequences of *M. separata*

Complete ORF:

>MS|comp149511_c0
MSLSLDPGPGIAALQAWGGQVAAYGASNQTVVDKVPPDMLHMVDPHWYQFPPMNPLW
HGLLGFTIGCLGFISITGNGMVIYIFMSTKSLKTPSNLLVVNLAFSDFLMMCCMSPAMVVN
CYNETWVWGPLACELYACAGSLFGCASIWTMTMIAFDRYNVIVKGIAAKPMTNNGALLR
ILGIWVFSLAWTLAPFFGWNRYVPEGNMTACGTDYLSKDWFSRSYILIYSVFVYFMPLLLI
IYSYFFIVQAVAAHEKGMREQAKKMNVASLRSSEAANTSAECKLAKVALMTISLWFMAW
TPYLVINYTGVFESAPISPLATIWGSLFAKANAVYNPIVYGISHPKYRAALYQRFPSLSCQAS
PDESGSVASGATAVSEEKPAA-

>MS|comp158503_c0
MAENGPGMAALQAWGGQAAVYGAANHTVVDKVPADMLHMVDAHWYQFPPMNPLWH
ALLGFTIGVLGFISITGNAMVIYIFMTAKNLKTPSNLLVVNLAFSDFLMMVAMAPAMVINC
YNETWVFGPFACELYGCAGSLFGCTSIWTMTMIAFDRYNVIVKGIAAKPMTKTGALQRIL
GIWLFSLAWTLAPFFGWNRYVPEGNMTACGTDYLSKDWLSRSYIIVYSVFVYFLPLLLIIY
SYYFIVQAVTAHEKAMRDQAKKMNVASLRSSEAANTSAECKLAKVALMTISLWFMAWTP
YLVINYTGVFESAPISPLATIWGSLFAKANAVYNPIVYGISHPKYRAVLYQKFPALACTVSP
DEAGSIATNATVVADEKSEKPAA-

>MS|comp163867_c0
MATNYTEDIGPMAYPLKMVSKEVVEHMLGWNIPEEHQDLVHDHWRNFPAVNKYYHYVL
ALIYTMLMVTSFLGNGIVIWIFGTSKSLRSPSNMFVINLAVFDMMMMLEMPLLIMNSFYQ
RMVGYQLGCDIYAVLGSLSGIGGAITNAVIAFDRYKTISCPLDGRINRVQAFILIAFTWFWA
LPFTILPALKVWGRFVPEGFLTTCSFDYFTDDQDTKVFVVCIFVWSYAIPMTLICYFYSQLF
GAVRMHERMLSDQAKKMNVKSLAANKEDASRSVEIRIAKVAFTIFFLFVCAWTPYAFVT
MTGAFGDRNLLTPIATMVPAVCCKVVSCIDPWVYAINHPRYRAELQKRLPWMGVRESDP
DSVSTTTSVATAQSTAQPAAEA-

>MS|comp164210_c1
MYNQTENSYYDAHFAPFKSGGPSGVEMLGEGLTGDELAAVPEHWLSFPAPPASAHTALAL
LYIFFTAAALVGNGLVIFIFSTTKSLRTSSNLLILQLAILDFIMMAKAPIFIYNSAMRGFASGT
FGCQLFALMGAYSGIGAGMTNACIAYDRHSTITRPLDGRLSRGKVLLMIAFVWIYSTPWA
LLPLFKIWGRYVPEGYLTSCTFDYLTNTFDTKLFVACIFACSYVFPMSMIIYFYSGIVKQVFA
HEAALREQAKKMNVESLRANQNASAESAEIRIAKAALTVCFLFVASWTPYGVMALIGAF
GNQQLLTPGVTMIPAVACKAVACVDPWVYAISHPKYRQELQRRMPWLQIQEPDDSVSTAT
SNTVSSAPPAAPA-

>MS|comp165164_c0
MQTLFEDLEDECPWYYFDGSYKLLLGACLFIFGVFGVLLNGWLFATFIHSHLLVFRSHILV
LNLCTASLGRNLVGFPFAGSSAVAKRWLFGPSCCQLFAFLNQFFGVFQMTALFVAVLERYL
LANYYRRERTLYTRFYWSLVGLSWFNSVIFSTPPLFGYGKYSCDSTGTTCTFLWPSTHSGA

KHIGFSIPYILVCGVIPVIAVFYYMGKAVRLERIFYKGEVQKEQTRLTQTIHAVCVATLALWI
PAAILVGWQWLPLLIYGYRPHVPPALAVIAPIASEAATSVPVLCFIAGDERLRAALLGRMR
KHYALLQPERAKRYNRA-

Incomplete ORF:
>MS|comp156702_c0
LDDLKQWKRRRRSADNFKKKLNDESGILKVTVGAVKELASYQNSVDIVETTALSVSDNK
LALIAKFIERWPVERWRSLGLFSDDYLLRINSHWLEFPPPDSWIQYTLGSTYVLLAGTGAF
GNVLVLLMYIRCRSLRTPGNILVANLALSDFLMLAKTPIFIFNSFNLGPALGKTGCVIYGFV
GGLTGTTSIATLSAIALDRYWAVVRPLEPLRALTAIRARLLAIGAWLYACIFAIIPALNIGYGS
YVPEGYLTSCSFDYLTEDFSPRCFIFVFFCAAWLAPFCTITFCYISIFRVVVFNRSMSYGNQD
QRLSSRHVKERTKRKAEIKLAFLVMAVIALFFISWTPYAVVALLGITGRKDLVTPITSMIPAL
FCKTAACINPFIYIITHPKFRKEFKKMLYRDKSKRMRGTLRTTDYCTDTTRMHRPSKDLSD
TDVEIVEMKDIPYQTDEKRDVKIKTISSKVAERQDSQTSMQTIKSLQDSVVSPPSWYMKPQ
FKKKRSFHRRSTRTNSSQTSTSDPII-

>MS|comp41007_c0
VESLRANQNAGAESAEIRIAKAALTVCFLFVASWTPYGVMALIGAFGNQQLLTPGVTMIPA
VACKAVACIDPWVYAISHPKYRQELQRRMPWLQIQEPDDTASTATSNTVSSAPAAAPA-

>MS|comp44349_c0
ENSYYDAHFAAYKSGGAGDTEMLGAGLTGEDLAMVPEHWLSFPAPPASAHTALALLYIFF
TAAALVGNGLVIFIFSTTKSLRTSSNLLILQLAILDFIMMAKAPIFIYNSAMRGFATGTFGCQ
LFALMGAY

2. CSP protein fasta sequences of *M. separata*
Complete ORF:
>MS|comp116602_c0
MKTLFILCALVVAVCARPEEQYTTEYDNIDIDEILNNDRLFKSYFECLVGEGKCTPAGKELK
SHMPDALQTECSKCSPKQKEGTKKVMKFLINNKPEQWKRLCAKYDPEGKYASKYEKEL
KEVSQ-

>MS|comp141234_c0
MRVLIVLSCLVVLAFAAEKYNAKYDNFDVETLISNDRLLKAYINCFLDKGRCTPEGSDFK
KTLPEAIETTCAKCTEKQKGNIRKVIKAIQQKHPKEWDDLVKKNDPSGKNRANFDKFIQG
SR-

>MS|comp148349_c0
MKTWLLCLCVLTVVVSCYSQGPNRYENFNADAIIQNDRILLAYYKCVMDKGPCTRDGKN
FKRVLPETLATACGRCNPAQKTIVRKLLLGIRTKSEPRFLELLDKYNPDRSNRDALYTFLLT
GQ-

>MS|comp149055_c0

MQIKYALLLCCVAAVSLAQTQRPAVSDTALDDALQDKRFIQRQLKCALGEAPCDPIGKRL
KTLAPLVLRGACPQCTPQETKQIQRTLSYVQRNFPQQWAKIVRQYAG-

>MS|comp149356_c0
MKADCVLLATLMAVVAADFYSSKYDSFDVQPLLENDRILLSYTKCFLDEGPCTPDAKDFK
KVIPEALETTCGKCSPKQKQLIRMVVKAVIERHPEAWQQLSDKFDKDRKFKDSFDKFLAE
ED-

>MS|comp149520_c0
MKVVLLTLCLALGVLAQDKYESANDDFDVSEVLSNPRLLNSYSKCLLNQGPCTPEVKQV
KEKLPEALETRCAKCTDKQKQMGKALAQEVKKNHPDIWKQLVAMYDPQGKYQQAWQD
FLKE-

>MS|comp152291_c0
MKLLIVLALVAAALARPDDSHYDEKYDNFNIDEVITNERLLKNYAHCLIGDGKCTPEGNE
FKKLLPEATKSNCGKCTDKQKVHVAKAIKAIKEKLPTEYETLRSQIDPEGAHAEDINKYVA
KYAP-

>MS|comp152624_c0
MNALLIAIFALAVPTALAYDEKYDKIDVDKILGDDAMFTAYINCMLDKGECTQEHSADFR
KLLPEVIATSCAKCNAIQKQNVRKTVKALSEKRPDDFVAFRAKFDPKGEYEKDFTAFVMG
TD-

>MS|comp154133_c0
MKFVLLLCVMVAAVVADDKYTDKYDNIDLDEILSNKRLLDAHYKCVMDKGKCTAEGKE
LKDHLTEAIENGCAKCTENQEKGAQKVIDHLIKNELDMWRELAAKYDPTGNWRKKYED
RARAAGIVIPAE-

>MS|comp154647_c0
MKLIVAVALLCVVAMAWGKPASTYTDKWDNINVDEILESQRLLKAYVDCLMDRGRCTPD
GKALKETLPDALENECSKCTEKQKSGSDKVIRHLVNKRPDLWKELSTKYDPDNIYQDKY
KTQIESAKQ-

>MS|comp154792_c0
MMMMYSSLAMMLLTYLTIQSNATETSTYTTKYDGIDLDEILNNERLLIGYVNCLMDNGPC
TADGKELKKNIPDAIENDCKKCTDRQRDGSDRVMHYLIDHRPDDWVKLEEKYNSDGSYK
MKYLSSKKTEDSKETNVTKSDEETKNSSKE-

>MS|comp155046_c0
MNFLVLSVVVTLAAFATADLTYTDRYDHVNVDEILDNRKLLVPYIKCTLDQGRCTPDGKE
LKAHIKDAMQTGCAKCTKKQKKAAKKVVKHIRAKEQDYWKQIVNKYDPGNEYTETYE
AFLASPDESK-

>MS|comp156988_c0

MKSYIALLVLSVAAMALARPEEAKYTDRYDNVDLDEILSNRRLLVPYVKCILDQGKCAPD
GKELKEHIKEALENECAKCTETQKSGTRRVIGHLINHEDAYWNELTAKYDPQRKFTAKYE
KELKEIKQ-

>MS|comp158640_c0
MQIIILTALCVGLVAGLHVQAGPQMTDAQLEQTLADKNTMQRHIKCALGEGPCDPVGRRL
RTLAPLVLRGACPQCSMQETRQIRRTLAFVQRNYPWEWAKIVRQYAEFKLRTLSVT-

>MS|comp160411_c0
MVNLFLKMRAVFVLCVLVYVVVGQELNDMGNMPKYDSRYDYLDVDAIFTNKRLVRNY
VDCLINSVRCSPEGKALKRILPEALRTKCVRCTERQKRAAVKVIRRLKNDFPEEWSKLASR
WDPTGDFTRYFEEFLAKESFNTIPGSGSAIPTSSPLAPPRPIPTMPPTIASPAPGPTEPTPPRPA
ILNRFGEDGELMQGSPSSGVMTPRPMTQATPRPTTTMRSTVNSRPVPPRPTMMTWAGAAS
NTQPTRFPLRPVSDLPPPYSTAITLIDQIGYKIIKTTELVTDLLRNTVRAVVGR-

>MS|comp160989_c0
MKTILVLCVLIAAVCARPEATYDTRYDNFDVESLVENVRLLKSYGHCFLGTGPCTAEGSAF
KKTIPDALQTGCGKCSPRQRHLIRVVVNGFQTKTPDIWKQLVKKEDPNGEFKETFTRFLK
ASD-

Incomplete ORF:
>MS|comp142679_c0
MKFVLVLCLFAAATLADDKYTDKYDNINLDEILENKRLLLAYVNCVMERGKCSPEGKEL
KE

>MS|comp147733_c0
MNSAIVLCVVALAGMVLARPDGGTYTTKYDNVDLDEILANDRLLVPYIKCLLDEGKCAP
DAKELKEHI

>MS|comp148423_c0
KEHIREALENGCAKCTDKQKEGTRRVIAHLIKHKNADWQKLKAKYDPEGKYTHKYEKE
LEEVQH-

>MS|comp154103_c0
DGKELKTHIREALEQDCAKCTKAQRDGTRQVMGHLINHEVDYWNELKAKYDPKNLYST
KHEQELRKLKQ-

>MS|comp154103_c1
MNSFTVLCLFALVALAVARPDGKYTDRYDSVNLDQILSNRRLLVPYIKCMLDQGKCTPDG
KELKTH

>MS|comp599465_c0
MKLIVAVALLCLVAESWAASTYTDKWDNINVDEILESQRLLKAYVDCLLDRGRCTPDGKA

LKETLPDALENECSKCTDKQKSGSDKVIRH

3. OBP protein fasta sequences of *M. separata*

Complete ORF:

>MS|comp116343_c0
MSKFTCLVLCVVAASISRAYASEEDKAAFRAAIQPIVDECSKEHGVSSDDIESAKTAGSAD
NIKPCFLGCVLKKAEILNAKGEYDSDKALTKLKKFVPDETKYAKYAEIGKKCESVNEKA
VSDGEAGCERGALLTACFLENRADIL-

>MS|comp118070_c0
MIFRFLLSFYLIEFYGAQARTDQEIKAWFFQEGMECNKEHSISPKEILMLKENKIPDTENAK
CFVACIFKKTGMLDSKGMFDAAASVAMTEKDFADDPQKQENSKKLLESCKKVNYEAVT
DGEKGCERSVLLHKCFVETAPQLGIKLP-

>MS|comp129850_c0
MYKFTCFVFYILYAVFTQAESDSSDSSSDEVFEKLSHECMEKFGVTEDDLNGVIKTSDVT
NIDSCYWGCYFTKMGVLNDKGQFDMNNFQTTMKKLMKDDEDYDNLEKLVKKCEPVK
DETVADGEAGCERGTLFAVCFVKNDGDFI-

>MS|comp141177_c0
MIKFSVVCLYFVVVAVHFWNVKCMTKDQEQEIIKAMKPLAEECASYCGLKDEDLKKYQ
GGDDMNPCFKKCMMQKLGLLDQEGKYDKATLHETMSQYGEDKEKAQKIEDQIDSCFM
ANADNNGDDEEAIKKRVDVMFNCIKELKE-

>MS|comp141270_c0
MTMWFRALAMLVAGLAAAQAIEMDEDMAELARMVRESCAAETGADVALVEQVNAGA
DLMPDAKLACYMKCTMETAGMMSDGEVDIEAVLALLPPELAAHKAPSLRACGTVHGA
DHCDTAWKTQKCWQAANKADYFLI-

>MS|comp141612_c0
MKLFVVLCIVLVTEIYAAYVPLPSNHTDSNLEECRKTSEFTDETLNKMKTNPFVEDGGEIF
KKFIKCYLEKTGAITDDGKLNVDEAVPKLGPNFAKKIFEHCKTHVEKVGEEFVVVPTTTA
SDYSECFRQGVSNYIWNAKQEGFEPFTYEWQK-

>MS|comp141702_c0
MSKFSCLAFCVVVVSLNSVLAEDGPANEGDVLDIVFECAKENEVKASEILAVMTSRDVTL
VNPCLWSCCLKKGGFIDDKGQYVLNPGLQYVKNIVKSDQFYTFIEKSAKQCESVKDKAG
SECELGALLAACILEQMMKM-

>MS|comp142589_c0
MLKSSVFLCCLCFCALTPYLALAMTDEQKAQIHQHFETIGKACNTGSNVITADDITNLRA
RKIPSGPNAPCFLACMMKQIGIMDDSGMLQKETLLELAKSIFKEPEEMKLIEDYLHSCAPV
NNESVSDGAAGCERAMLAYKCMYENASQFGIEI-

>MS|comp148301_c0
MLLIEIVKFLILVAMCEAMTMKQIRNTGKMMRKSCQPKNNVEDEKIDPIAEGIFIDEKEVK
CYMACIMKMANTIKNGKLNFDAAIKQADLLLPDEVKEPAKEAILACKKAADGHKDICDA
SFHVTKCIYNQNPGIFYFP-

>MS|comp150111_c0
MDRKGLCLLIVAMFLATGSDAMSRQQLKNSGKVLKKNCMNKNQVTEDQIGTIDKGNFV
EDKKVMCYIACIFEMTNVIKNGKLNYDASIRQIDLMYPPDLKEGAKAAVDKCKDVQKKY
KDICEASFYVAKCMYEFNPADFIFA-

>MS|comp150160_c0
MTKVLLTTVLIVITFALTRAASTQMKDAMPKEAMTTTTMANQDSSIDSTDIDVIAVMNAC
NESFRIEMSYIQAMNESGSFLDETDKTPKCFIRCVFTNVGIVSEDGKQFNPARAAFIFAGER
NGKTMDDIGDMTVACAADRQETCPCERSYQFLRCLMSMEIEKYEKS-

>MS|comp150353_c0
MFKSGIVYFVVVAVFLGNALGISDELRSQIQSKLLTVGAECIKEHPLSIDDLAAFKSRVFPE
GENAGCFSACIFNKLGLFDDKGTWAHVEAFENAKKVFENKEDLKNVQSFLTACAEVNEA
EVTDGEKGCDRAKLAYDCFIKNYEKLGFDFDF-

>MS|comp152336_c0
MYARVLIRFLEAKLCAAKILNLLQMNQSYLLLLIAACVEISYGMTRAQVKKTMTIIKNQC
MPKNSVTEDQVKNIEQGDFNEDPNIMCYVACVYKSLQVVKNDKLDVGLISKQIDALYPP
ELKEPTKKAVALCINSQDNYNDLCSRVFHGAKCLYEKDPACFIFP-

>MS|comp152359_c0
MSKFTCLVFFIVAASISKAYASEEEKAAFREAVKPIIEECSKEHGVSIDELKAAKAAASAD
GIDNCFLGCVFKKAEVINAKGEFDLDNALTKLKGFVSNEDHFAKFEDIGKKCASVNEKPV
SDGDAGCERAAMLTACFLEHKGEMPLNF-

>MS|comp152974_c0
MFNVYFCVFVCGVLSLNIKASSLDDLKLKYVEVIIECSNDYPITVADMTELRKKIMPDSEP
IKCLFACVYKKTGMMNEKGELSVDGVNEMSRKYLADDPAKIKKSEEFTEACKSVNDVA
VNDGERGCERAALIFKCTVEKAPDFDLI-

>MS|comp153447_c0
MFGFGFLSLAAVLLCLGSTSALTPEEESSLKEALHPFVLECADEYGIPEEKFEEAKAKGSA
DDIDPCFISCFLKKAEFFDGDGKLDVEKTNEFVKAHLTSEHVIKFFEAVGGECAKVNDEE
VTDGDKGCDRAKLLFDCIQELKSKIGD-

>MS|comp156324_c0
MTSKCGLLLAVMAAVAGSVMGTAEVMSHVTAHFGKALEECREESGLSAEILEEFQHFW
REDFEVVHRELGCAIICMSNKFSLLQDDSRMHHVNMHDYVKSFPNGEILSGKLVELIHNC

EKKFDSMTDDCDRVVKVAACFKVDAKAAGIAPEVAMIEAVMEKY-

>MS|comp156380_c0
MMFFCSTVFFYPFLQMTREQVKSSGKMIKKTCSVKNNLSEDQVKDVDKGNFIEDKNFMC
YVACVYKMGQTIKGNTINHDMMLKQVEMMFPTEMKAPVKAAIEHCRPVAKKYKDVCE
AAYWTAKCTYEFDPANFMFP-

>MS|comp156923_c0
MFKWLIIVALVAASYGDPISESRDNKSATLKPLSVCCDIPELGDPKHLAKCSNPKLPGPCN
DIQCVFEESGFLTDVNTLNKEAYKNHLKQWEENHAGWSVAVDKAIKDCVDSDPRQHLN
YPCKAYDVFTCTGIAMLKKCPAAAWKC-

>MS|comp158230_c0
MFGSKTVFYLLTVFSACFGAVDIKKYLKVCDRNAIDVNDCMADAVQKGIAVMIHGIPEL
GVPPIDPYLQKEFRVEYKNNQILAKMILKNIYVEGLKEAKVHDARLRADDDKFHLEVDLT
SPMVAVKAQYYGEGQFNSLKIVAYGDFNTTMTDLVYTWKLSGVTEKNGTETYVRIKDF
YMRPDLASIVTEFRNENPESREFTDLGTRFANENWQTLYKEFLPYAQANWKRIGIKVANK
LFLKVPYDQLFPSSS-

>MS|comp158329_c0
MKTFLVLAACILLAQGLTDEQKEKLKKHNTECLTETKVDEALVNKLKTGDYKTESEPLK
KYALCMLMKSELMTKEGKFKKDVALAKVPNAADKPNVEKLIDSCLANKGNTPHQTAW
NYVKCYHEKDPKHAIFV-

>MS|comp158921_c0
MFKFCVFLAFCVAASYAAPGGGTYCGETPSVIYQCLNSPKVISAVPAKCAKYDDECEKLT
CVFRESKWLDGTAVDKAKVLAHLDQYERDHAEWGPAVQFAKTACLGPELKAQGVFLN
CPAYDVTHCILSSFIKHATPTQWSSSASCSYPHAYAAACPVCPSDCFSPQVPYGSCNACYL
QPRTP-

>MS|comp159796_c1
MYRLVILSIVAVSALADEMGMRECGRMFHPHSVRCCKKTSELKDKFMLSEDLKECFQMR
GNPVTCENEVCIAKKKGFATDDDKLDYTKLEEVMTKEIDDKDLLADMIKNCVNGDLEK
YGPPDFCEFMKMRHCISMQMLNHCPDWDDAGECSKLKGAVADCVKLFA-

>MS|comp160581_c0
MIRSCLVLAAVFQVLFGQESGPDPRDGFRQPVPHYCLSPPPGTDLHKCCPIPKLFPDGDME
RCGIEKASVDQSKSPPKPRIPCKESICLMQNANMLLANHSVDYEKLRTFVDIWADSNPEFT
EAILEAKKACAKDGGPSGPPVCEQDRIFYCLTSNVLWNCKLRDFEDCRVLKAHMDECRP
YYWKKREEDEANAQTS-

>MS|comp161605_c0
MRAGGLCGQYSQELYKMKSFVVFCLVLVVGVYANVTLPPTQQEKAQKLAAECVKESGV
STEVLAEAKKGHIVEDENLKKFTFCFFKKAGIVDSDGKLNVEVATAKLPPGVDKEDAKK

VLEGCKSKTGKDTADTVFEIFKCYHKGTKTHILLAGL-

>MS|comp162075_c0
MVLHRSATMSVRLALVVIASLLIAVECSQEIMKNLAINFAKPLEDCRKEMDLPDSVLTDF
NNFWKEGYEFTNRQTGCAILCLSSKLELLDPEMKLHHGRAQEFAQKHGADEAMAKQLV
DMLHSCMQTTPDDANDPCLKTLKVVTCFKTKIHELKWAPSMDLIVGEVLAEV-

>MS|comp163093_c1
MFHGGIWTKLQGFYKWYVLGLVSIGYILGELGHYLIGTTSKVTAEDLHYGDKSCMLNLS
HVSLSDLPVVCEKVNSSDICATLTLNGTRYCEWGYNGLGIDYQVLAGPAFMAVFTVVGV
ILGVVADKYNRARILSFCTLIFVIAILIMGSVKEYWHLVVLRMIMAAGESGCNPLATGILT
DLFPEKQRALALSIFNWGIYGGYGIAFPVGRYIPDVNAWGLSWRVCYYGAGIVGLVITLL
TFLTLREPSRTTIGEEGTGNKAGDSTLESGKKMPQVTIWHVIAQPRIILLCLAASIRHCGGM
CFAYNADLYYRDYFPDVDLGWWLFAVTVGIGSVGVVAGGIISDKYVEKMGVRSRVLVL
ALSQLIATLPAFGSVVFGPLWAMITLAISYFFAEMWFGIVFAILVEIVPLSVRSTTVGVFLF
VMNNVGGNLPILVDPVSKIIGYREAIMIFYAGFYGISSIMFFLTMFLMDGPVDKPAATPEEP
KRTGLDNRAFTQDELPTRNAGRSLPTAPSERL-

>MS|comp167844_c0
MSKFTCLILCVVAASLSSFYVRAHTPTFRDSLQPLIEACSEEYPGVTSDMVNQAAKEGNP
NIIHPCFNGCVFKKAGFINEKGEYDTNSALTNLRKLVTHEEQYRKLAEIARQCTSVKDTVT
DGEAGCERGARISACFLQQKDNVSL-

>MS|comp173090_c0
MSKFTCIVFCIVAASLTKVSHAVTEEEKAAFREVMAPIIDECSGEHGVSKADIQAAKEAGS
ADGIKPCFLGCVMKKTETLDDKGLFDAETALSKLRTFVKSDEDFAKFEEIGKACMSVNEK
SVSDGEAGCERAKLVLACFLEHKADIPF-

>MS|comp60723_c0
MFTGTLPVVLCLVAAAYGGKEKPVFSDEIKEIIQTVHDECVGKTGVAEEDITNCENGIFKE
DTKLKCYMFCLMEEASLVDDDGTVDYDMLVSLIPDEYYERTTKMIFSCKHLDTPDKDKC
QRAFEVHRCSYGKDPDLYFLF-

Incomplete ORF:
>MS|comp108141_c0
MFKFTGLLYCAVAVSLSGVVRAGEEDAEAFREAIKPFITECAKEHGISWEDIAKAKETHTV
SSLKPCFVGCIFKKFEIINDKGE

YDLEANLDKIKIFVKNEDLLTQLRDIMKKCVSVNDESVSDGNAGCERAMLLANC-

>MS|comp114857_c0
MTPTTLVLALGLAAALADVNVMKDVTLGFGQALDKCRQESDLTEEKMEEFFHFWRDDF
KFEHRELGCA-

>MS|comp121812_c0
MDAQKLGIQRELVTVGIQCIKDHPLSLSDIRAFRNKMIPNGNDPKCFVACLFKKIGVMDN
MGMISPAKAKENAKKVFKGSEEHL

KNVDEI-

>MS|comp124490_c0
VRLALVVAAWLFIRVDASQDVIKNLSMNFAKPLEDCKKEMDLPDSVTTDFYNFWKEGYE
FTNRQTGCAILCL-

>MS|comp131182_c0
ENTFLQLPFALMQVSMDCFAGQRVMDASVMFECAVYDCKWENFDKANMRLVLVMLQN
AQKTMTLSAGGVKTLSFSALMSVFRGI

YSAYTTLRTTMK-

>MS|comp133839_c0
VTLPPIQNEKADNYAYACIEESGVTTEALKESRKGNFFDDEGLKKFTHCFFQKADIVTCDG
QLNIDVALAKLPVGVDKVLAKEI

LEGCKTKTGKDSTDKVYEMFKCYFSQTPVHIKLSFDIEKKTVSA-

>MS|comp133858_c0
QIHEDEALKCYMNCLFHEIDVVDDNGDVHLETLFNTVPGTVRNQLINMAKECEHPEGDT
LCHKAWWFHQCWKKADPVHYFLP-

>MS|comp141404_c0
MNTSNFQSIFCILCIVGLFFSYSHAMTRQQLKNSGKLMKKSCMPKNDVTEEEVGDIEKGK
FIENRNVMCYVACIYTMTQVVKNN

KLSYEAVIKQVDIMFPAEMRDAVKAAATFCKDTTKKYKDLCEASYWTAKCMYD-

>MS|comp143661_c0
KFTCLVLCVVAASLSQAYASEEEKAAFREAIKPIVEECSKEHGVSHDELKSAKDNQNADNI
KPCFLGCVYKKAEVFNSKGEYDV

DKALEKLKKFVSNDEAYAKFAEVGKKCVSVNDKAVSDGDAGCERGALLTACFLEHKAEV
PL-

>MS|comp145270_c0
MWNFIVLCLALCSCTYALTEEELKMQMTKLVMKCNKDSQVDMMELVQLQSFVVPTKMA
TKCVLACAYKAADVMTADGLYNIEHA

YKIAEQMKNGDEKRLENAKKMADLCVKVNEERS-

>MS|comp145826_c0
MSKFSGLVLCAVAATFISVASGESLSESLKPVIEKCSKEHGVTDADIQAAKAKGSSDGINPC
FLFCVFQNAGIFDAKGEYDAAT

GLKNLRQIVKDNDQYKNLEHAFNDCSKIKDKPVSDAAGCEKGLHL-

>MS|comp154997_c0
MADSRMRLVCLVCVIFAASSAMASKELLTKMSSGFTKVVDQCKNELNVGEHIMQDMYN
FWREEYALVNRDLGCMVMCMAAKLDL

IGDDQKMHHGKAEEFAKSHGADDALAKQLVGLIHECETTHAGVEDACSRTLEVAKCFRS
GIHELDWQPKVEVIVTEVLTDM-

>MS|comp158322_c0
HELKWAPSMEVVMEEIMTAA-

>MS|comp161014_c0
MSRHFNLLTDSSRMHHDNTEQFIQAFPNGEVLARQMVSLIHGCEKQFDHEEDHCWRILH
VAECFKQACVQHGVAPTMEMMMTEF

IMEAEAR-

>MS|comp161583_c1
MNIKTSFIDYPAHNIPEVIRVGQLFQVATYLADGHKDICDASFHVTKCIYNQNPGIFYFP-

>MS|comp393373_c0
MSRLKTAIAECTKVVESIFEVCEYASVFNDCMHIKMEHILDQVMMERRLEAIGKITTDPEQ
WGDTEDEILKLVKDEL-

>MS|comp427468_c0
MFVYRYMIAFHTLCVVYYVVGQHEKESKFGEIVKRTVIATAHTCMDHVNATVKDLENLR
DREEN-

>MS|comp502645_c0
MKSFVVFCVLVAGAFAANVSLPPKQNEKANQIATECMKESGLKPEVLAEAKKGHISDDEH
LKKFTFCFFKKAGIVSEDGKLNTE

VALAKLPP-

>MS|comp672758_c0
LEKIGVVKSNKYSKLGFMTAVTPLVFTNRKKLDHMKTVSENCDREVNHKQETPCQLGNE
VTTCIFKYAPELHFKS-

>MS|comp71248_c0
KKMADLCVKVNEEKVSDGEKGCDRAALIFKCTVDNAPKFGFKL-

4. OR protein fasta sequences of *M. separata*

Complete ORF:

>MS|comp163536_c0
RRVAMENQNPPERVVPVQYGRGSRAFRQFKNPPQPHMCIQDTIKGTTEKLYINVLGWQKI
ANPKQYSDPIPLYGGMQVPQNYGPNAGRPPMIVFAVMVNPDILKANGKNATSPTDREALV
SLLCDFVEAMNPGLALARNPVIMKDRDLAGELKDVWLAVQNKREREKGMNQDVMYKV
YDIDGIGEDNMNEDDNNQMKQGGSPNRGSGDKKPVKSSKQILLNAGQKSEFDSGMNNC
QINQSHLNRDNKTGNTPDTTYCTPVYGQIVSTRENHNQINDIQQKFASNEIKPSSSFRKDW
NPMHGKTTEGWDEFSKRNISTIKNDEKKRCIKDKSKISNGKSHYDFFPYFDNKTVNETDV
PEEEDSMNKEQNSNEENSKIIIDPMQKLVLHSTDKNICDNNTSALSSISS-

>MS|comp165911_c0
MSRKCNNDPNSFCYVCGILTFKKQRQNFTNLVLECYHQCFGFSVAHQDKFWAPHVCCITC
VKNLTDWKKGARAMPFAVPMIWTEPRDHVSDCYFCLTDIKGINYKKKKQLSTLLPPLHIK
LGLIKNFVKGMDRSGSGVLYLKEKFPKISDAKIKEGIFVGPQIRELIKDSTFEEKLSGLEKC
AWNAFKNVVANLLGNHKGDSYRELVSELLLSYQALGCNMSLKIHFLDSHLDFFPDNLGA
VSDEHGERFHQDISSMEKRYQGKWSPNMLADYCWTIKRDQPEAKHSRKS-

>MS|comp157094_c0
MKLVSDASELEGVEKTEDIPYMQILRKSMWILDAWPKTPNESRIHRYYMVFIKICCLIPGII
YLRNNTGKLSSFEMGHTYITVFMNTIAFSRALMVLSKKYNDILFYFLNEMHLFNYRNKSD
YSYQTHILVHKISHFFTMYLLLMMCLGILLFNLTPMYNNYTDGMYREVKPPNATFDHSVF
YSLPFDYTTNLRGYLALFTFNWYMSCTCSSYFCVVDLTISLLVFHLWGHMRILQYNLQNF
PKPASLLVESKDGEIKDHKYNEQEQIDIHNRLRENLHYHSVIIDFQRRMSDSFGAVLLIYFL
FHQVSECLLMLECSQMDKKALLRYGPLTVVIFQQLIQLSIIFELLGSSNDKLIDAAYCVPWE
YMETKDSKMVLVMLIQSQVSMNLKAMSMLTVGVQTMIAILKTSFSYFVMLQTVAEEEE-

>MS|comp164245_c0
MMTKVKAQGLVSDLMPNIKLMQAAGHFLFNYHSENAGMSNLLRKIYASTHAILIIVHFAC
MGINMAQYSDEVNELTANTITVLFFTHTIIKLGFFALNSKSFYRTLAVWNQSNSHPLFTESD
ARYHQIALTKMRRLLYFICGMTCLSVVTWITLTFFGESVRMITSKETNETLTEVVPRLPLKA
WYPFNAMSGTMYIVAFAFQVYWLLFSMAIANLMDVMFCSWLIFACEQLQHLKAIMKPL
MELSASLDTYRPNTAELFRASSTEKSEKIPDAVDMDIRGIYSTQQDFGMTLRGAGGRLQNF
GQQNANPNGLTPKQEMLARSAIKYWVERHKHVVRLVASIGDTYGTALLFHMLVSTITLTL
LAYQATKINGINVYAFSTIGYLSYTLGQVFHFCIFGNRLIEESSSVMEAAYSCQWYDGSEEA
KTFVQIVCQQCQKAMSISGAKFFTVSLDLFASVLGAVVTYFMVLVQLK-

Incomplete ORF:

>MS|comp11107_c0
YAYLIMMRHLLSVISSWPLKILDPLDTGAIQRRKIWVSIQRFFHMAVCLSTVVGGVMYVM
LHKKSMTFFELGHLYISLLMTFVIF

>MS|comp111125_c0
EEEQLFIDITVKKIEFLFRCAGFNIKCSPKTHMDTIKSRTIFIVNFIWMNIDFVGCVVWFITGI
VNSKSFVELTFIAPCVMP

>MS|comp143892_c0
VGIRLPLFYYMTPERYPLAYTIVVISFFYISHFVMVTDLKMQTHLMHLLCQFTVLGHSTT-

>MS|comp1530192_c0
MSQVVSTCDVLVVVATAGAGVYGAPRRMRKMLKFMENIASVDTSIGGQYSLVTERKLCG
IILGILIFFSVLIADDFTFYALQAKKLDREWEVVTNYLGFYLLWFVVLILELQFAFTALSVRA
RFSAVNDALALTA

>MS|comp1754988_c0
KAKKFNKIYWKAVAFVSITSNCTHTMAPLMLHFGKGMELIFPVCSYAFFSESFRHMFIYPI
YFYQSVGITFHMLYNL

>MS|comp1789939_c0
NSTPKYIIFKLQGIPGWLSGLAPAFGSGRDPGVPGSSPTLGSLHGACFSLLLCLCLSL

>MS|comp1802501_c0
MVVLSLGISSEIGILKLLYMFLYIDNIRGLTDAFLDFDALTTPGSRQASNLLRYMRDVKKRA
IFYWMVLMVNGTIYVFMPLAIP

>MS|comp2429514_c0
MKSFKALKETLVETKRLVKEDSLESVMFIVNVVPNFVGFSYKKDGKVTSWFWILHLSLLF
YVYA

>MS|comp43532_c0
KYKGFNETFKLCAFSLAFAFLYPNRTTAIKRCVIITAIVTFCGGQLFWFITYTFKCLYTLDIY
NFARN-

>MS|comp790023_c0
EETKTWITFLSSISKLLLSQTILGVLAFALGPFSLISSNYLKTGEIKLVLPFIVWYPFDELDTRI
W

>MS|comp957466_c0
WIMGLHQIDCFKIHMKFWRFLAIWPGDDDSRSYNRYSKIFISTFVFVYYVLFTINFYFLPRR
LDIFIDDMMFYFTDCSVVSKVLTFVLMRHKIVAILDVLESEIFQPDDDDGRAIIA

>MS|comp102652_c0

CAQLDIVKEKILSITSINGQRGTREVDEALAKNYNKLVNCIKHHQAIIKFTELVENAYHPYL
LFQLVGSVGIICMSALMILVVDW

>MS|comp102652_c1
VSSQITCGMWALKPLFDDADRKFPFDMWMPLNPEKTVQYYIGYTFQLVTICISAYMYFGV
DSVAFSSVIFGCAQLD

>MS|comp1032844_c0
KFLGVWVPGENGSFLHTGYRAFMMTLQYLFLIFQIIYIIQVWGDLDAVSQASYLLFTQACL
CLKVTVFQVNMGMLKDLP

>MS|comp104640_c0
VGKLEAIYRVTIGIGFGLDAISLCLFFVLPLEVCLNFAPLIYHSLFIFFLYCFQGQRLTTASEK
FEIAVYCCGWENLRVKEQRQVLLMLKQAQKPVIVYAARVIPIRIHTFASTMQSIYKF

>MS|comp107793_c0
QTRAQTPCKLTASNFAEVNLKAFMKVPKPNFY-

>MS|comp110478_c0
LTLVVTALTILCSTGFFMWNAGDITVEAEVLPTAMFSSGWENCWHESSVRVRKLIVIAMLQ
AQEPVALTGLGIIVLSYQSYVSIVKSSR

>MS|comp110712_c0
MLRRIAEQLSKIFGVVIFIHMVSASLVICFFGFLAVVYSGLADTVANLMTVFNAMTTIFFLT
LSG

>MS|comp116110_c0
LSFQSENLREWLYQCPWYEQDTKFNRALFIAMERMKKPIIFKAGHYISLSRPTFVSILRMS
YSYFAVLNQTR-

>MS|comp116394_c0
LRNVKKRAMLYWVVVAGNGVLYVMKPVAMRGRNLPENYFIIYGLEPMFDTPNYQIAYF
MMIAGVFFICYVPATVTAFLIVITGYAEAQMLALTEE

>MS|comp1239304_c0
FWQKYWREVLDYVTEADKFERQSGDPIKTMIVDTYTKYCRRLSYFYWAIVFTTFWTTTG
TPLMTYLSSFTFRENMRN

>MS|comp124817_c0
KWERIPTSQRRPLLMMMMRAQRPLRLTAAGFTHMDNACFLAIMKAAYSYYAVLSQRQE-

>MS|comp133273_c0
KVRQIVSECLASDAAVVAGSRFSSNLLSALRHVKERAMVFWMVIIFNGVVYIVKPLLTPGR
HFTEDKFILFGLEPMQENPNYQIANMLFAAGVFFTVYVPANITAFLIVVTGYVEAQMLSLS

EELLHLWEDAEEHYYKTEQAISIFDSNKTDLKDKAMNEYVAKHLKDIIRSHGRNINL

>MS|comp1348560_c0
KFDMITVEYLAAVALDTFVYCYYGHQIILQADRVSTAAYQSMWHTMGVLPRRVLL

>MS|comp1355432_c0
RISEAVMECKWECIPTSQRRPLLMMIMRAQRPLKLTAAGFKDIDDVCFLSIMKAAYSYYAV
LTQKQEK

>MS|comp139413_c1
GGNRFVHLVQRYEAVTWYTKAVSEVFNGSMSFEFFSCSAIVCVITYRMSFVSFLFLLVSY-

>MS|comp142810_c0
LWRFFAMLTLREIEHEIRKFGLEYCDLPTMLENVSILLRVLTVNIDSNYHKGIKIYSYILTAV
TAVCFYYVFLFSMSWFVFYRCRITGELVGAMVVLSLGISSEIGPFKLFYMCFHMDKTQKI

>MS|comp147212_c0
LKALSVETSNRFYSSGWEQIDDQRVRRMILFMVARAQAPIAITAFNMLAFDMELFTSILQS
SYSMFTLLRS-

>MS|comp150812_c0
SSVIICLDGFNVMAINNIATIITCLSFLFTSLLQIFFLCFFGDLLMTSSIEVSDAVYNCRWYLA
DTRLGKDLLLMQTR

>MS|comp150812_c1
VISVLNVFYFVLAVMVALSPLSLMALNYYTTNELELLLPLLAVYPFDHYNIRYWPWVYLR
QVWSTVIVTIDICTADYIFYIFCTYIRMQFRLLKQNIETFIPDDVIDGRLRNNEAVRTEFVVL
VKWHQDLISSTKMLEAIYTRSTLLNF

>MS|comp150890_c0
CYLVDNKIFQAFEVGDAGWTCGWHETPLGLMIRDDIIIIIIRAQQPVTIKFTGLPHVQLETFS
SCMSSSYSYFNMLRQYSK-

>MS|comp1523813_c0
DKIQMTRLNKSLKHYEVILTFCDTVQDILSVTLFFQYGVSTLIICVVMTGLALPSSIEFRAFL
AMFLFTMTLRIFVPGFLGTQLSHESEEL

>MS|comp152388_c0
MADEELEFKPFSDTYRKITFALSVGMIYPNPKTEKWRLASIPILVTSIMPLACMILYDMYKC
WMVKDIVNIIRHSTVVGPFLGGFFKMILMYHKRIQAKEILDEIDRDHELFNDISDIYKDIAR
ASIKNSQIYSERGWVITVSTCVMTFPVIAIVLNIYNFTFKSEPEKYMIHDLVIPMSESEMRFE
SPYFE

>MS|comp152487_c0

DLKNRTYEQAINCLYPWNFETSLSGYIVANLSGWYGTFLCGSSVSMFDLFLCLMIYNLWG
HFKILIYNLENFPRPAAEVVDAAGEERSGMRIGSEMYSQSELDEVAIQLKDAIQYHKLIVD
FTNNMSDAFGMALFLYYSFHQVTGCLLLLECSTMTAAALTRYGPLTIIMFGELILLSIIFETI
GTMSEKLKDAVYDVPWEYMDTKNRRTVLIFLIKVQEPVHVKAGGLLDVGVTTMASILKT
SFSYFA

>MS|comp1998815_c0
FVFLTLYTVVMTLQVMVPCWFGSRLIEKSSQITFSVYDCDWTPRCRRFKSNLRLLVERAN
RPITIRGGKMFLLSLATFTAV

>MS|comp2023556_c0
IVMVASFTAFLVFGLMQIFLYCYYGDTIMRSSMQISSAIYNSNWYNVGVAERKSFLIVLIRA
QKPCELTANG

>MS|comp33920_c0
KKDFEYICSEGEKYRAQYFEEQLLTWKICIVMCTFNAGIGIAMNIFAYLSLFYFMATHDAD
TEESRPLLFPFWIPNVDLT

>MS|comp35555_c0
SFCIATTHLRLKLKLLTLKVEKAFKNSRSRHELRMKMNEAIRDHQDAYDFYVQLQNVYGP
WLFAVFLLTSFMISFNLYQIYLLQRIDPKYTSFGVVGV

>MS|comp360965_c0
AIVYKKLLIFEQIICGPVVCLTAYCIAEVFFFTIMNLLVFCCHFFSYF-
YFQKIDKGEFQAILLLLCIATIVMYFTPSLLCTYLSIKVRILN
>MS|comp3974_c0
LMSYLCSVSFCMLDLFLSLMVFNVWGHFKILLHDLATFPLPAKNMSSNNYG

>MS|comp447468_c0
LEISDAVYNCKWYFATPRVSKQLLLVQTRAQEPCKLTAAGFADVNLNAFMRVLSSAWSYF
ALLQTVYSGK-

>MS|comp50905_c0
EKWNEVAYKSFLVTALTQTWLLCWYGQKLIDSSKRLADALYDCGWYNGSKRAKSAILIM
LHRAQKGIYVTTHGFSIISLASYSTIIKTAWSYFTLLLNFFKD

>MS|comp55130_c0
LTFILLHMNELVLEMTGIQDHRWRVYCTVVLSQCVDHYVKIKSFSNRLNVICRPFYLALIL
VAIMLVCMCSVKIAISNK

>MS|comp611033_c0
KSKESVMKINKCILHFDEVGKFCDLVEDVFSATLFVQFGMASCIICVCLMRFTMPAPMSYF
VFLGTYMCVMILQILVPCYYGTRITDKSSLLAFSIYNCDWTPGSRQFKS

>MS|comp633077_c0
FIPENSNTTLTYEMYTENEQKNVGVKLGELINYHRDIISFTDKISEVFGPMLFAYYGFHQAS
GCLLLLECSQMTAAALIRYLPL

>MS|comp638207_c0
AYVVGWDTLVVSIMIFFAGQLKITCYYCRMMIDAAKLDKSHRNIADCHRFYTTLVEYTHL
FNSLISPVMFVYLVVISVNLGVNIIQIVEIQDDIPTLVSSVLFVVACLIQLLLFYWFANEVTIE
STFVSYS

>MS|comp79584_c0
QFKSNMRIFVERANKPLSITGGKMFCLSLPAFTSIMNSAYSFFTLLQQMKD-

>MS|comp80641_c0
DKRVRQIVLFMLMRARIPMGITTVFYVINLDTFAEMCRQSYTLFNLMNAAWE-

>MS|comp813906_c0
GSISDRLKFAVYGLPWESMDKKNRRTVAFFLMNVQEPVHVKALGLADVGVTSMTAILKT
SLSYFTFLKSM-

>MS|comp892037_c0
MELWHQIRKFGLEYCDLPTMLWNVSVFLKLLTLNIYGKNKSGIP

>MS|comp90072_c0
LSLMVFHLWGHFKILINLLNDFPRPSSGSNFTTEDGFLIRAEKYSKEELQQVSERLAECIIY
HREIVNFTNTMSDVFGPMLFVYYVFHQTSGCLLLLECSQMTTQALIRYVPLTLTL

>MS|comp904167_c0
LMQVGMDCFTGQRVMDANVKFERAVYDSKWENYSLSNMKIVLTMLQSAQKTMKLSAG
GMIMLSFSCLMQVIRSIYS

>MS|comp926109_c0
MRLKIIRDFLLKESFDFDRPDIDLYNFHPQLRIFLAVKGVFFSNRNSRLRYIWPAICIQLSVV
A

>MS|comp94306_c0
FAQLTVSLIIICVTAFQLSIAHPDNMVRLLSMGTYLLNMCFQVFIYCYQGNQLSEESSEIAG
AAYESPWYKCSLRVRRSLLIVMVRTRRALRLTAGGFTTLSLASFMAIIKASYSLFTLLQQV
DE

>MS|comp94444_c0
EITNQSAKVGEAIYCSGWECGLSKLPGVRSTILFVIARANRPLVLTAGGMYDLSLTSYTTLV
KTSYSALTVLLRFRHD-

>MS|comp948927_c0

RSRQFKSNMRLFVERTNKPLSITGGKMFCLSLPAFTSIMNSAYSFFTLLQQMND-

5. GR protein fasta sequences of *M. separata*

Complete ORF:

>MS|comp134624_c0
GTASRGLAMNLHKALEAEHPALKVAQYRHLWVDLSHMMQQLGRAYSNMYGIYCMVIFF
TTTISLYGALSEILEHGLSYKEMGLFVIVGYCMTLLFIICNEAYHASRKVGLEFQVRLLNVN
LGAVDRSTQREVEMFLVAISKNPPIMNLDGFTNINRELFTANVSFMSTYLIVLMQFKLTLLR
QSARKTIKSVIKAVFNTSTTMLDDDISEDEEEA-

Incomplete ORF:

>MS|comp636901_c0
MRACGLFCVDVCLILHLAELLRTYSIVLLQFAFL-

>MS|comp19407_c0
CANELPKKLLMVELSWMNTDAQTEVNMFLRATEMNPSQISLGGFFNVNRTLFKSLLATM
VTYLVVLLQFQISIPDESQ

>MS|comp121176_c0
AILFVIFLVPHFWIPFVGWGVAHQVAIYKTNWGKFQVRYYRVTGENLKFPNLKTTIVIISVG
CLLLAVCFLLSLCILMDGFLLKHTTA

>MS|comp1194584_c0
MGVESAKVEEVTASPVPTESGTRPTRPTHCVVGGAHAFILKISSFFGLAPLRFESRSNGFTV
S

>MS|comp82756_c0
QLLHTLAGGIKATQKCRPDSSEDHRLMGGYVHSIYFVFSFAFLVVRSLAVSLTAAKVHAAS
LEPAHSLYDVSSANYCVEVERFLAQIHGDTVALTGLQFFQVKRGLVLTIAGTIVTYELVLM
QFTGVSPASTEVS

>MS|comp1687776_c0
IYSEERVSSNMVDKDVQSMLLPLNLMSHTMFCPKYRIKDNIISPNSLISKFVSMIAALLSIFT
F

>MS|comp109140_c0
LSLHKVQILRTAEGKFEEAVIEYLFTVYLFPIIAVPILWYETRKIANVLNGWVDFEMVYNQL
SGRTLPVKLYKKALAIAVIIP

6. IR protein fasta sequences of *M. separata*

Complete ORF:

>MS|comp159644_c0
MIIDLSWSPWRMAEDLASETGLPLVRTLLGSQQLVKALDDYLESRNATDAAIILESESDVD
RTLYELLGASSIRVWVHAGLTRDSAKALKTMRPEPSFYTIVGDSGFVMDTYRRAVKEKLV
RRSYRWNLILTDYSTPDVTQLVLPTVTLQADQVECCKLMKREDCTCPSDFQRKQYILNGL
IQYISETYSKLERDLPLTTTSVDCEDPQPVMNSTRDRLYRQFAEDSEISNETLFYWEAERSG
LFLRSRFILSTYSLEAGQQTIATWSADDKYKLLPGVELEPLKMFFRIGTAPAVPWTLMKVD
PNTGEQMFDEDGQPLYEGYCVDLIARLSETMSFDYEIVSPKSGDFGKKLPNGTWDGVVG
DLMRGETDIAISALTMTAEREEVIDFVAPYFEQTGILIVIRKPIRKTSLFKFMTVLRTEVWLS
IVAALVLTGFMIWLLDKYSPYSARNNPDAYPYPCREFTLKESFWFALTSFTPQGGGEAPKA
LSGRTLVAAYWLFVVLMLATFTANLAAFLTVERMQTPVSSLEQLARQSRINYTVVEGSSV
HQYFINMKFAEDTLYRVWKEITLNATSDQAQYRVWDYPIREQYGHILLAINASEPVPDAK
TGFQQVNEHTDADFAFIHDSAEIKYEVTRNCNLTEVGEVFAEQPYAIAVQQGSRLQEDLSR
ALLELQKERFLEQLASKYWNESARQACPDADESEGITLESLGGVFIATLFGLGLAMITLAW
EVFYYKRKEKNKVQAFNAKPEKVAFESKSTLESKVAESMAKLRKRAKVGKKGANIAKN
VTFGDSFKPVSEKGVSYISVFPKEYRP-

>MS|comp161214_c0
MAGIELIISSICNATFCEVPYNETYKGSGTDPMAEKDTNFMNLMKEVNGKVLKVTTYNNT
PLSWSEYHNGTMVGRGVAFTVMDILRKKFNFTYEVVLPKRNYEMGTKMTGDSVIGLLNS
SKVDMAAAFLPTLIAYRERVSFSIDLDEGIWVMMLKRPKESAAGSGLLAPFNELVWYLVL
AAVLTFGPCITFFTRVRSKLITDDEGVLPLKPSFWFVYSAFLKQGTNLAPEANTTRVLFVT
WWLFMILLSAFYTANLTAFLTLSKFTLAIEYPRDLYQKNYRWVASAGSSVEHVVKSEGEEL
YYLSAMISNNKAQFLSVISDTDFLGIVKKGAVLVKEQTVVDHLMYNDYTSKKDVEESEK
CTYVVAPNAFMKKQRAFAYPVGSKLKSLFDPVLTQIFQSGILDFLKRADLPSTKICPLDLQS
KDRKLRNSDLIMTYLVMVAGSATAVAVFAAEIFIKRYVSGKIITSKKLKKKKSKMSKKSTN
YDDSRPPPYDSLFGKNPRFNVETTRTKIINGREYYVFETASGEKKLIPARAPSSFLYRSDK-

>MS|comp165699_c0
RPTPDANSRDRLGKIKTTYSMNGEPEITSAFYFDLSLRTFLTIKSLLDSGKWPNDMKYITCD
DYDGKNTPNRTLDLKTAFQEIKETPTYAPFFIPQDDPMNGRSFMEFSTELLAITVKDGASIS
SHSLGSWKAGLSSNLTLTDPNNMSNYSAQLVYRIVTVEQRPFIVRDDKAPKGFKGYCIDLI
EEIRQIVKFDYEISLAPDGNFGIMDENGNWNGIIKELVDKKADIGLSSLSVMAERENVVDF
TVPYYDLVGITIMMKLPRTPTSLFKFLTVLENDVWLSILAAYFFTSFLMWVFDKWSPYSYQ
NNREKYKEDEEKREFTLKECLWFCMTSLTPQGGGEAPKNLSGRLLAATWWLFGFIIIASY
TANLAAFLTVSRLDTPIESLDDLSKQYKIQYAPLNGSSAMTYFQRMANIEEKFYEIWKEMS
LNDSLKEVERAKLAVWDYPVSDKYSKMWQAMEEAVLPNTIEEAIQRVRDSKSSSEGFAW
LGDATDVKYHVMTSCDLQSVGDEFSRKPYAIAVQQGSPLKDQFNNAILQLLNKRKLEKLK
EIWWNNNPETMKCEKQDDQSDGISIQNIGGVFIVIFMGIGLACVTLGVEYWWYKWRKRP
VVGDVVHVTQVEPAKSSRNNVDKQGEGFNFRGRNLGLSFKPKF-

Incomplete ORF:
>MS|comp100717_c0
PAVSSVRVVMFSTLLAALFVFTAYSAKIVAILQTPSDALRSIADLTRSPMVVGVQETTYKKV

YFQESPDEATQQLYRRKIEPQGERAYLSVVDGIARVRTGFFAFQVEQSAGYDIIKQTFTERE
KCSLKEIEAFKLPLVAVPMRKHSGYRELFASRLRWQREVGLMDRERRIWLVSRPRCEASSG
GFVSIGIIDVLPALQVLALGAFLSVVLLASEVG

>MS|comp104026_c0
LLDVDTAIEPKGRDGVEIRIVEELCRWMNCTVEIVREDVEQWGDIYPNETGGIGVLGSVV
EDRADVAISE-

>MS|comp119947_c0
FPTTNHLLDFLNAKRKYVFADTWGYRVNGTWNGMTGYLLRGEVEIGGSPMFFTFERVSI
VDYISSPTPTRSKFVFQQPKLSYENNLFRSE

>MS|comp1203294_c0
KSFTRSMAYSIEKLPAGEYITQEAMLDMMLMLEDFYYEQCVVMMRKSSPYTEKVSQLVG
RLHQSGLLLAWETQIALKHLNYKVQVEVR

>MS|comp124776_c0
KKYEKYSEQTLDQTCLPPTWSDITIFLLSAISQQGSSNELKGTLGRLVMFIIFLAFVFLYTSY
SANIVVLLQSTSNQIRTLPDLLNSKLELGVEDVAYNRYYFSAAYTSKDPIKKAIFERKVAPK
GKPNYMSIEDGVKAIQK

>MS|comp129582_c0
KEVMLGRADIGIAGMYLTSDRVRDMDVSMAHSHDCAIFITLMSTALPRYRAILGPFHWHV
WLALTFTYLFGMFPLAFSDKHTLRHLLHNSGEIENMFWYVFGTFTNCFTFLGKNSWSKTN
KITTRLLI

>MS|comp131374_c0
INLNGYKYYRRWDFQNITMRMILVAQPAPKHLDLELLAGPSPVPGVAMITQTCATVLYEVA
VMHNIKYFPTITDRWIGTNERNSSKVVSNSLYFREQDLSPVIRFLKSVQENSDVLSPSLTAIE
TRYYYRIPTVGPGKFE

>MS|comp135198_c0
FVGKYFNEGEKCGLREIQYLQVIDPWLAVRKNTPFREMFKIGTKRIQEHGLQYRENLLMY
EKRPKCS

>MS|comp136712_c0
KRPFAFNMNTGTGYRIVSAFFREHEKCGLQEIDYIQNSKPWLCSQKYSPFGEMFKVGYIRI
QEHGLSDRENRLIYAKKPVCSVWEEASTQSIWWISILCVFYCCMERFWLLSCLALRFWCI
VCRREE

>MS|comp138392_c0
WAAVAFTYFYASIGILTAQGFATTSYPFLKSFGMMIGQSQYQNVASNSWKIRSVTGWLLIA
GLILSSAYGAGLASTFTVPKYEPSIDTVQDIVDRDMEWGATHDAWIFSLTLSTEPLIKQLVN
QFRIYSFDKLKEKSFTRSMAYSIEKLPAGNFAIGEYITQEAML

>MS|comp1582011_c0
SPYLELFRVVFKQLRESGIQSAVSKRLQVSKPHCSSKMSSFSSVGLMDMKPVLIFMLYGIC
LSVAIAVAEI

>MS|comp1872421_c0
IDKITDEVRFEMQRSLTGHFLAAGSGTIKIPSAEEKGLTLEDTQGMFLLLAAGFIIAGTALVS
EWMGGFTRRCRFTRKVDTPISVNSG

>MS|comp2100871_c0
SVNSGEHLIPTPKTDIDSEIRIIGDTESRLNFDSRPSTAVSTDTLDGQIINVSEDNFDVHNTFN
VSRFDS

>MS|comp395436_c0
FAETWGYPINGTWNGMIGDIKRGKVDLCGIVTFITLERLPILEYLNQPTPVTAKFVFRQPPL
SYQNNLFILPFTSNVWM

>MS|comp50464_c0
SFISLIPNSESLVMQIFNVSEMGCSDYIVRMQEPQKFMEAFETVVHKGNVRRSDRKIIFLPY
DEEYNEEYDVDLPSLVFSMKGSAYV

>MS|comp634464_c0
KRRRFVSHDMESLDENRSTVSRAAFGDPIKHDK-

>MS|comp71302_c0
GGSNFVSVSMVDCYPALVILSYGAIVALFLLGLEILVYKREKILRKLSCVRHHNYE-

>MS|comp82009_c0
GRLQRQEAEVGLASFFIRPDRMRVADFISETCVLACTFIFRQPSRSAVSNVFLAPFSGGVWA
ACGGVA

>MS|comp849777_c0
DTIKQLLAGFYRVGTLDRGGWEKWFLNSSDPDTAKLLKKLELVPNVEAGIRNTTKAFFWP
YAFLGSKAELEYIVQANFT

>MS|comp88910_c0
EDHPDCKNGIMCYCQELMDAFVSALDAAIQDEFDVAAQVSDEEWESIRPNKIQRRDMLL
KHMQVKKGLVVHMINSTFFEVMTSKKS-

>MS|comp99629_c0
VTKWEWKKTSHLIESRKRDAGVLRANGVDVIVLIFGAACQQGSPSELKGSLGRVVMLVL
FLTLMFLYTSYSANIVALLQSSSSHIKTLDDLLHSRIKFGVHDTVFNRYYFSTATEPVRK

7. SNMP protein fasta sequences of *M. separata*

Complete ORF:

>MS|comp162080_c0
MLGKHSKLIFAVSIGFLVVAIIMASWGFQKVVDKQIQKSVQLENDSLMFDKWLKLPMPLD
FKVYVFNVTNVEEVNKGEKPILEEIGPYVYKQYRERTVLGYGPNDTIKYMLRKRFEFDAE
ASGGLTEDDEVTVIHFSYLAALLTVHDMMPSLVGVINKALEQFFPSLEDAFLRVKVRDLFF
DGIYLSCDGDNAALGLVCGKIKGDLPPTMRMAEGSNGFYFSMFSHMNRSETGPYEMNRG
RDNIYELGNIVSYKGQEIMPMWGDKYCGQINGSDSSIFAPINEANVPQKLYTFEPDICRSLY
VDLVEKRELFNISAYYYEISESALAAKSANHDNKCFCRKNWSANHDGCLLMGLLNLMPC
QGAPAIASLPHFYLGSEELLEFFQSGIAPQREKHNSHVYIDPTTGVVLSGVKRLQFNIELRK
IDTIPQLSSVPTGLFPMLWLEEGATIPDSIQQELRDSHKLLGYVEVARWLLLTVAVIAVIASA
VAVARANALLSWPRNSNSVSFILGPSVT

>MS|comp162251_c0
MLLPKELKYSAIAGGVAVFGLIFGWVLFPVILKGQLKKEMALSKKTDVRKMWETIPFALE
FKVYLFNYTNAEEVQKGAKPILKEIGPYHFDEWKEKVEIEDHEEDDTITYKKRDTFYFNP
ELSAPGLTGEEIVVMPHIFMLGMALTVNRDKPAMLNMVGKAMNGIFDNPPDIFMRVKAL
DILFRGIIINCARTEFAPKATCTALKKEGVSGLIIEPNNQFRFSIFGTRNNTIDPHIITVKRGIQ
NVMDVGQVVAVDGKPEQTIWKGACNEYQGTDGTVFPPFLTENDRIQSFSTDLCRSFKPW
YQKKTSYRGIKTNRYIANIGNFAEDPELQCFCPDPDKCPPKGLMDLAPCIKAPMYASMPH
YLESDPALLNNVKGLNPDINQHGIEIDFEPISGTPMVAKQRIQFNLQLLKTDKIDLFKDLSG
DIVPLFWIEEGLALNKTFVNMLKHQLFIPKRVVGVLRWWMVSFGSLGAVIGIVYHFRDHI
MRLAVSGDTKVSKVTPEEGQEQKDISVIGAQAQEPAKINI-

Supplemental data S7. Amino acid sequence of Opsins, CSPs, OBPs, ORs, GRs, IRs and SNMPs from organisms other and *M. separata* used in phylogenetic analyses.

1.opsins

>BmorCeropsin
MSISMDAGPGFAALQSWSSQVAAFGNSNQTVVDRVSPEMLHLIDAYWYQFPPMNPLWHA
LLGFTIGVLGFISMMGNGMVIYIFMTTKNLKTPSNLLVVNLAFSDFLMMCAMSPAMVINC
YNETWVFGPFACELYGCAGSLFGCASIWTMTMIAFDRYNVIVKGIAAKPMTNNGALLRIL
GIWAFSLAWTVAPFFGWNRYVPEGNMTACGTDYLTKDWFSRSYIVVYSVFVYFAPLLLIV
YSYYYIVQAVSAHEKAMREQAKKMNVASLRSSEAANTSTECKLAKVALMTISLWFMAW
TPYLVINYTGILESAPISPLATIWGSLFAKANAVYNPIVYGISHPKYQAALYKRFPVLQCHST
TTDEASSVASGTTVMEEKPTA-
>Bmoropsin2
MRREAARRVVAAARRAANAAPGRVVRGKMSSQCLWHACRALSAGLLLMLLGGAMAVI
GYYADTLSVAEEIRGNATVSVKDEARGFHLNNLSYAGPIVMGFGGFIVVAACVMTFEARD
SAAKVTPARPQTLPRPIPRRGPPCTAPARLDQLGVYRLPHVLPLPHALPLAPLPPFRHRPSS
HTRNAADERGKGKARFGSAPDLRIGSTRAALPVSALRRPLRRYALSVDEPPQSAVSASAA
DSECGSQSSLALDLHGSGACAGVTLRVRDNTRRRPLARQQRLRDDTIQPTPVDSMNNINV
IQECTSGTSWEDTGDNTTIEMTHLSVDNEIRACSAPPPANSPPSTPHPADTCVTIFQEDDKDI
THEPPSSQQSVSEWVHQRTTELRTHRNYGAHFAAFKSSGAGAVEMLGDGLSGDDLAAVP
EHWLTFPAPPASAHTALALLYIFFTAAALLGNGLVIFVFSTTKSLRTSSNLLILQLAILDFIMM
AKAPIFIYNSAMRGFATGTIGCQIFSVMGAYSGIGAGMTNACIAYDRHSTITRPLDGRLSKG
KVLLMMAFVWIYCTPWALLPLLKIWGRYVPEGYLTSCTFDYLTNTFDTKLFVACIFVCSY
VFPMSMIIYFYSGIVKQVFAHEAALREQAKKMNVESLRANQSGASQSAEIRIAKAALTVCF
LFVASWTPYGVMALIGAFGDQQLLTPGVTMIPAVACKAVACIDPWVYAISHPKYRQELQR
RMPWLQINEPDDTTSTATSNTVSSAPPAAPA
>Bmoropsin1
MISLDPGPGMAALQAWGGQVAAYGAANQTVVDKVPPDMLHMVDPHWYQFPPMNPLW
HALLGFTIGVLGFISISGNGMVIYIFMSTKSLKTPSNLLVVNLAFSDFLMMCAMSPAMVVN
CYNETWVFGPFACELYACAGSLFGCASIWTMTMIAFDRYNVIVKGIAAKPMTNNGALLRI
LGIWAFSLAWTLAPFFGWNRYVPEGNMTACGTDYLSKDWFSRSYILIYSVFVYFAPLLLII
YSYFFIVQAVAAHEKAMREQAKKMNVASLRSSEAANTSAECKLAKVALMTISLWFMAWT
PYLVINYTGVFESAPISPLATIWGSLFAKANAVYNPIVYGISHPKYQAALYARFPSLQCQSAP
PDDGGSVASGATAVSEEKPAA-
>Bmoropsin-Rh3-like
MDTVPKGINNLTYLKRDDHALFSSKYDDETLTKGDIKLWKRRRRMSEILDRRSNVSGEVK
ETRLPIKYSKYEVNELVSSEANNEVLIKKFKELWPVNLWRRFGLFTDDYLHLVNPYWLSF
APPHPLLHYGLGAFYIMMTTVGSTGNALVMFMYFSLPICLVNFRCRSLRTPGNILVANLAL
SDFMMLAKTPIFIFNSFNLGPALGKTGCVIYGFVGGLTGTTSIAILTAISLDRYWAVVRPLEP
LRALTAIRARLMAIAAWIYATLFAIIPALDIGYGRYVPEGFLTSCSFDYLTEELPPRYFIFVFFC
AAWLAPFCTISYCYISIFRVVVCSRNITTKNQEQRLSTRHVKERTKRKAEIKLAFLVIVVIAL
FFISWTPYAIVALLGIFGKKDLIKPLTSMIPALFCKTAACINPFIYIITHPKFRKEFKRMLSRDK
TERSGTIKTIGYYTEASKFHRPSKDFSDTEVEIVEMKDIPYRNEDTTNTVEEKINTVSAKLA

KREGSQKSQSSISMKSFEENVVSPPSWYCKPKFAKKKSFHRRSTHSMVSQNTDPTV-
>Bmoropsin-Rh-like
MRSALYSSLNILWKSNRSAVYHVPTRQAGSGLAEYPGTPLASHGRPGFEDTHVMIFIGFGF
LMTFLKKYSYSALGFNWLLAALVIQWALLCQSFYHMKNNTIYVTKKSLLEADIMSATVLI
TFGALLGVASGLQLLFIAIVETAVACLNMWLVADVFKASDVGGSIAIHTFGAYFGIGVSMA
LKSKNKDTTPTTNGNTTPSVDLNAPSYISDVTAMIGSIFLWIYWPSFNSGLTNTDAEYQRA
VVNTYLSLAAATVTTFVLSSALNEHKGKFDMVHIQNSTLAGGVAVGSVCNMQIGAGGAV
AIGIGAGILSVLGYRYLTPKLTKIGILDTCGVNNLHGMPGIYSGVLSVLFAGLATSDAYGAE
LGTVFSGIGQNGRTIGQQALNQFLALVVTLVLSLNGRTIGQQALNQFLALVVTLVLSLVSG
FLTGKKQFVSTSEVPKRL-
>Bmoropsin5-like
MGVFGALLNGWLFATFIHSHLLIFKSHILVFNLCAASLGRNLLGFPFSGSSAVAKRWLFGPS
CCQLFAFFNQFFGVFQMTSIFVLILERYIQVKFYKREKSVYIRLYWTLIAVSWINSFLFATPP
LFGYGLYSCDTTGTSCTFLWPSMSSGAKQLGYAVPYILICGLIPIVAMFYYMGKALRLEKIF
YKGEQQREQKCLTQSAHAVCVGTLALWIPAAVLAGWQWVPLLIKGYRPHVPPTLALIASI
ASEAATTVPVLCYLAVDERLRAALLGRMRKQYALLQPERAKIYKRV-
>Bm_Pteropsin
MTSFLENMNDTVTKAYDEGFPLLMPRWGYVASAFVLFLIGFFGFFLNLMVILLMFKDRQL
WTPLNIILFNLVCSDFSVSVLGNPFTLISALFHRWIFGHTMCVLYGFFMALLGITSITTLTVIS
FERYLMVTRPLTSRHLSSKGAVLSIMFIWTYSLALTTPPLLGWGNYVNEAANISCSVNWHE
QSTNTLTYIMFLFAMGQILPLSVITFSYVNIIRTLKRNSQRLGRVSRAEARATAMVFIMIIAF
TVAWTPYSLFALMEQFATEGIVSPGASVIPALVAKSSICYDPLIYVGMNTQFRQSIKRIFGIH
TKSKNSHTERGYNNTLMSPAHKLSLNNTKFNSETNSSTATKALKDKR-
>Bmorparapinopsin-like
MTSFLENMNDTVTKAYDEGFPLLMPRWGYVASAFVLFLIGFFGFFLNLMVILLMFKDRQL
WTPLNIILFNLVCSDFSVSVLGNPFTLISALFHRWIFGHTMCVLYGFFMALLGITSITTLTVIS
FERYLMVTRPLTSRHLSSKGAVLSIMFIWTYSLALTTPPLLGWGNYVNEAANISCSVNWHE
QSTNTLTYIMFLFAMGQILPLSVITFSYVNIIRTLKRNSQRLGRVSRAEARATAMVFIMIIAF
TVAWTPYSLFALMEQFATEGIVSPGASVIPALVAKSSICYDPLIYVGMNTQFRQSIKRIFGIH
TKSKNSHTERGYNNTLMSPAHKLSLNNTKFNSETNSSTATKALKDKRVFYEDASDVTNQE
YHRQYTRNPDLCTIQENKTVSDTDRSGESAYDDSSSNRKIDMFGESCRAVNEKSPVLTIMD
SERLLFSKVPCRKDDETEASSSNVYGNTAFEEHATDDTMNDNSNAENDYKNYVRHKMIR
QSFSLDFPRYSSENKKRKLSIETKLESLMQPKSFLKGTISKLFDHEYKQGHDSFKSYLCDKS
GTGNDEL-
>Dpleopsin-LW
MAITSMDPGPGAAALQAWGGQMAAFGSNETVVDKVLPDMLHLIDPHWYQFPPMNPMW
HGLLGFVMGVLGFISITGNGMVVYIFTTTKTLKTPSNILVVNLAFSDFCMMAIMAPPMLIN
CYNETWVFGPLACQLYACAGSLFGCGSIWTMTMIAFDRYNVIVKGLAAKPMTINGALLR
VLGIWAFSLAWTVAPMFGWGRYVPEGNMTACGTDYFDKSFANRSYIVIYSVFCYFAPLFLI
IYSYFFIIQAVAAHEKAMREQAKKMNVASLRSSDQANTSAECKLAKVALMTISLWFMAWT
PYLVINFCGIFDGAPISPLATIWGSVFAKANAVYNPIVYGISHPKYRAALYARFPALSCQASS
DDNVSAASAATACTEEKPSA-
>Dpleopsin-BL
MATNFTEDIGPMAYPIKMVSQEVVEHMLGWNIPEEHQDLVHEHWRNFPAVSKYWHFGLA

FIYTLLMLASMTGNGIVIWIFSTSKSLRSPSNMFVINLAIFDFMMMLEMPLLVLNSFHQRLL
GYQLGCDIYAVLGSLSGIGGAITNAVIAFDRYKTISSPLDGRLNHTQAALLILFTWVWATPF
SFLPAFRVWGKYVPEGFLTTCSFDFLTEDEDTRVFVMCIFVWSYVIPMCCICYFYSKLFSAV
RLHERMLKEQAKKMNVKSLAANKDDSGKSVEIRIAKVAFTIFFLFVCAWTPYAFVAMTG
AFGDRSILTPVATMIPAVCAKVVSCLDPWVYAINHPRYRAELQKRVPWMGVREADPDNTS
NVSGATAQTQNPAEA-
>Dpleopsin-UV
MIPTITMDNDTDNINVYGAYFAPLRSSEGTKMLIDGLTGEDLAAVPEHWHTYPSPPASAHT
ALALLYCFFTAAALIGNGMVIFIFLTTKSLRTSSNLLILNLAISDFIMMAKAPIFIYNSALRGF
AAGPVGCQIFSVMGAYSGIGASMTNACIAYDRHSTITRPLDGRLSQGKALLMIAFVWIYAT
PWSLLPLFKVWGRFVPEGYLTSCTFDYLSNTFDTKLFVACIFTCSYVFPMTMIIYFYSGIVK
QVFAHEAALREQAKKMNVESLRSNQNASAESAEIRIAKAALTVCFLFVASWTPYGVMALI
GAFGDQRLLTPGVTMIPAVACKTVACIDPWVYAISHPKYRQELQRRMPWLQINEPDDNVS
NTTNGTTNSTAAPTA-
>Dmelopsin1
MESFAVAAAQLGPHFAPLSNGSVVDKVTPDMAHLISPYWNQFPAMDPIWAKILTAYMIMI
GMISWCGNGVVIYIFATTKSLRTPANLLVINLAISDFGIMITNTPMMGINLYFETWVLGPMM
CDIYAGLGSAFGCSSIWSMCMISLDRYQVIVKGMAGRPMTIPLALGKIAYIWFMSSIWCLA
PAFGWSRYVPEGNLTSCGIDYLERDWNPRSYLIFYSIFVYYIPLFLICYSYWFIIAAVSAHEK
AMREQAKKMNVKSLRSSEDAEKSAEGKLAKVALVTITLWFMAWTPYLVINCMGLFKFEG
LTPLNTIWGACFAKSAACYNPIVYGISHPKYRLALKEKCPCCVFGKVDDGKSSDAQSQAT
ASEAESKA-
>Dmelopsin2
MERSHLPETPFDLAHSGPRFQAQSSGNGSVLDNVLPDMAHLVNPYWSRFAPMDPMMSKI
LGLFTLAIMIISCCGNGVVVYIFGGTKSLRTPANLLVLNLAFSDFCMMASQSPVMIINFYYE
TWVLGPLWCDIYAGCGSLFGCVSIWSMCMIAFDRYNVIVKGINGTPMTIKTSIMKILFIWM
MAVFWTVMPLIGWSAYVPEGNLTACSIDYMTRMWNPRSYLITYSLFVYYTPLFLICYSYW
FIIAAVAAHEKAMREQAKKMNVKSLRSSEDCDKSAEGKLAKVALTTISLWFMAWTPYLVI
CYFGLFKIDGLTPLTTIWGATFAKTSAVYNPIVYGISHPKYRIVLKEKCPMCVFGNTDEPKP
DAPASDTETTSEADSKA-
>Dmelopsin3
MESGNVSSSLFGNVSTALRPEARLSAETRLLGWNVPPEELRHIPEHWLTYPEPPESMNYLL
GTLYIFFTLMSMLGNGLVIWVFSAAKSLRTPSNILVINLAFCDFMMMVKTPIFIYNSFHQGY
ALGHLGCQIFGIIGSYTGIAAGATNAFIAYDRFNVITRPMEGKMTHGKAIAMIIFIYMYATP
WVVACYTETWGRFVPEGYLTSCTFDYLTDNFDTRLFVACIFFFSFVCPTTMITYYYSQIVG
HVFSHEKALRDQAKKMNVESLRSNVDKNKETAEIRIAKAAITICFLFFCSWTPYGVMSLIG
AFGDKTLLTPGATMIPACACKMVACIDPFVYAISHPRYRMELQKRCPWLALNEKAPESSAV
ASTSTTQEPQQTTAA-
>Dmelopsin4
MEPLCNASEPPLRPEARSSGNGDLQFLGWNVPPDQIQYIPEHWLTQLEPPASMHYMLGVF
YIFLFCASTVGNGMVIWIFSTSKSLRTPSNMFVLNLAVFDLIMCLKAPIFIYNSFHRGFALG
NTWCQIFASIGSYSGIGAGMTNAAIGYDRYNVITKPMNRNMTFTKAVIMNIIIWLYCTPWV
VLPLTQFWDRFVPEGYLTSCSFDYLSDNFDTRLFVGTIFFFSFVCPTLMILYYYSQIVGHVF
SHEKALREQAKKMNVESLRSNVDKSKETAEIRIAKAAITICFLFFVSWTPYGVMSLIGAFG

DKSLLTPGATMIPACTCKLVACIDPFVYAISHPRYRLELQKRCPWLGVNEKSGEISSAQSTT
TQEQQQTTAA-
>Dmelopsin5
MHINGPSGPQAYVNDSLGDGSVFPMGHGYPAEYQHMVHAHWRGFREAPIYYHAGFYIAF
IVLMLSSIFGNGLVIWIFSTSKSLRTPSNLLILNLAIFDLFMCTNMPHYLINATVGYIVGGDL
GCDIYALNGGISGMGASITNAFIAFDRYKTISNPIDGRLSYGQIVLLILFTWLWATPFSVLPL
FQIWGRYQPEGFLTTCSFDYLTNTDENRLFVRTIFVWSYVIPMTMILVSYYKLFTHVRVHE
KMLAEQAKKMNVKSLSANANADNMSVELRIAKAALIIYMLFILAWTPYSVVALIGCFGE
QQLITPFVSMLPCLACKSVSCLDPWVYATSHPKYRLELERRLPWLGIREKHATSGTSGGQE
SVASVSGDTLALSVQN-
>Dmelopsin6
MASLHPPSFAYMRDGRNLSLAESVPAEIMHMVDPYWYQWPPLEPMWFGIIGFVIAILGTM
SLAGNFIVMYIFTSSKGLRTPSNMFVVNLAFSDFMMMFTMFPPVVLNGFYGTWIMGPFLC
ELYGMFGSLFGCVSIWSMTLIAYDRYCVIVKGMARKPLTATAAVLRLMVVWTICGAWAL
MPLFGWNRYVPEGNMTACGTDYFAKDWWNRSYIIVYSLWVYLTPLLTIIFSYWHIMKAVA
AHEKAMREQAKKMNVASLRNSEADKSKAIEIKLAKVALTTISLWFFAWTPYTIINYAGIFES
MHLSPLSTICGSVFAKANAVCNPIVYGLSHPKYKQVLREKMPCLACGKDDLTSDSRTQAT
AEISESQA-
>Dmelopsin7
MEAIIMTTLPNLTTDAGDSSFWLTGALSLSEMLANSSHSHSTGSTTSTAGSSATESSAVNVG
KDHDKHVNDSVSTGLSNYSNYPSYIHYRDKYDLSYIAKVNPFWLQFEPPKSSTFLIMAAL
YCLISVVGCVGNAFVIFMFANRKSLRTPANILVMNLAICDFLMLIKCPIAIYNNIKEGPALG
DIACRLYGFVGGLSGTCAIGTLTAIALDRYNVVVHPLQPLRRCSRLRSYLIILLIWCYSFLFA
VMPALDIGLSVYVPEGFLTTCSFDYLNKEMPARIFMALFFVAAYCIPLTSIVYSYFYILKVVF
TASRIQSNKDKAKTEQKLAFIVAAIIGLWFLAWSPYAIVAMMGVFGLERHITPLGSMIPALF
CKTAACVDPYLYAATHPRFRVEVRMLFYGRGVLRRVSTTRSSYMTRSRSSFTHRLRTSTTG
EGGMGDHRMENYLMNNNLMMVPEETEENEEIVVVAEINNSISSVMEQSKF-
>Hmelopsin-BL
MATNYTDDIGPVAYPLKMVSQEVVEHMLGWNIPEEHQDLVHEHXRNFPAVSKYAHFVLA
LIYSFLILASVSGNGIVIWIFSTSKSLRTASNMFVINLAVFDMMMMIEMPMLVLNSFYQRLV
GYQLGCDIFAVLGSLSGIGGAITNAVIAYDRYKTISSPLDGRLNRVQASLLILFTWLWALPFT
IMPAFRIWGRFVPEGFLTTCSFDYFTEDQDTKVFVMCIFAWSYCIPMLLLCYFYSKLFGAV
RLHERMLKEQAKKMNVKSLAANKEDAGKSIEIRIAKVAFTIFFLFVCSWTPYAFVAMVGA
FGDRSILTPVTTMIPAVCAKIVSCIDPWVYAINHPRYRAELQKRLPWMGVREADPDTVSTS
SGATAQTQNPTAEA-
>Hmelopsin-LW
MAITSLDXGPGAAALQAWGGQMAAFGSNETVVDKVLPDMLHLIDAHWHQFPPMNPLW
HGLLGFVIGVLGFISVTGNGMVVYIFTTTKSLKTPSNILVVNLAFSDFLMMFMMAPPMVIN
CYNETWVFGPLACQLYACAGSLYGCVSIWTMTMIAFDRYNVIVKGIAAKPMTINGALLRV
FFIWAFSLAWTVAPXFGWGRYVPEGNMTACGTDYFDQSISNMTYILLYSIACYYAPLFLIIY
SYFFIVQAVAAHEKAMREQAKKMNVASLRSSDQANTSAECKLAKVALMTISLWFMAWTP
YLVINYAGIFKTMTISPIVTIWGSVFAKANAVYNPIVYGISHPKYRAALYARFPALSCQTAPE
DTGSVASAATATEEKPSA-
>Hmelopsin-UV

MENETQNHNVYGAYFAPLRSSDGVKMLVDGLSSAELEFIPEHWLSYPAPPASAHTALALM
YCFFTAAALIGNGLVIYIFATTKSLRTSSNLLILNLAVFDFIMMAKAPIFIYNSAMRGFAIGSL
GCQIFALVGSYSGIGAAMTNLCIAYDRHSTITRPLDGRLSRGKALLLIACVWIYTTPWSLLP
LFRIWGRFVPEGYLTACSFDYLTNTFDTKLFTGCIFIFAYAIPLCLIMYYYSGIVKQVFAHEA
ALREQAKKMNVESLRSNHNAAAESAEIRIAKAALTVCFLYVASWTPYGVMSLIGAFGDQ
QLLSPGVTMIPAIACKGVACIDPWVYAISHPKYRQELQRRMPWLQIREPDDNVSTGTNNTT
NSAAPAATA-
>Harmopsin-BL
MASNYSEDIGPMAYPLKMVSKEVVEHMLGWNIPEEHQDLVHDHWRNFPAVSKYWHYVL
ALIYTMLMTTSLLGNGIVIWIFGTSKSLRSPSNMFVINLAVFDLMMMLEMPLLVINSFYQR
LVGYQLGCDIYGVLGSLSGIGGAITNAVIAFDRYKTISCPLDGRINKVQAFILIAFTWFWAL
PFTILPALRIWGRFVPEGFLTTCSFDYFTDDQDTKVFVGCIFVWSYCIPMTLICYFYSQLFG
AVRLHERMLSEQAKKMNVKSLAANKEDASRSVEIRIAKVAFTIFFLFICAWTPYAFVTMTG
AFGDRNLLTPIATMVPAVCCKVVSCIDPWVYAINHPRYRAELQKRLPWMGVREQDPDSVS
TTTSVATAQSTAQPAAEA-
>Harmopsin-LW1
MPLTLDPGPGIAALQAWGGQVAAYGASNQTVVDKVPPDMLHMVDPHWYQFPPMNPLW
HGLLGFTIGCLGFISISGNGMVIYIFMSTKSLKTPSNLLVVNLAFSDFLMMCAMSPAMVVN
CYNETWVWGPLACELYACAGSLFGCASIWTMTMIAFDRYNVIVKGIAAKPMTNNGALLR
ILGIWLFSLAWTLAPFFGWNRYVPEGNMTACGTDYLSKDWFSRSYILIYSVFCYFMPLLLII
YSYFFIVQAVAAHEKGMREQAKKMNVASLRSSEAANTSAECKLAKVALMTISLWFMAWT
PYLVINYTGVFESAPISPLATIWGSLFAKANAVYNPIVYGISHPKYRAALYQRFPSLSCQASP
DETGSVASGATAVSEEEPAA-
>Harmopsin-LW2
MADSGPGMAALQAWGGQAVTHGAANQTVVDKVPADMLHMVDAHWYQFPPMNPLWH
ALLGFTIGVLGFISIAGNGMVIYIFSSTKSLKTPSNLLVVNLAFSDFLMMCAMAPAMVINC
YNETWVFGPFACELYGCAGSLFGCASIWTMTMIAFDRYNVIVKGIAAKPMTKSGALQRIL
GIWLFSLAWTLAPFFGWNRYVPEGNMTACGTDYLSKDWFSRSYILVYSIFVYFLPLLLIIYS
YYFIVQAVTAHEKAMREQAKKMNVASLRSSEAANTSAECKLAKVALMTISLWFMAWTPY
LVINYTGVFESAPISPLATIWGSLFAKANAVYNPIVYGISHPKYRAVLYQKFPALACTVSPDE
GGSVATGATVVSDEKPAA-
>Harmopsin-UV
MYNQTENSYYDAHFAAYKSGGAGDTEMLGAGLTGEDLAMVPEHWLSFPAPPASAHTAL
ALLYIFFTAAALVGNGLVIFIFSTTKSLRTSSNLLILQLTILDFIMMAKAPIFIYNSAMRGFAT
GTFGCQLFALMGAYSGIGAGMTNACIAYDRHSTITRPLDGRLSRGKVLLMIAFVWIYSTP
WALLPLFKIWGRYVPEGYLTSCTFDYLTNTFDTKLFVACIFACSYVFPMSMIIYFYSGIVKQ
VFAHEAALREQAKKMNVESLRANQNAGAESAEIRIAKAALTVCFLFVASWTPYGVMALI
GAFGNQQLLTPGVTMIPAVACKAVACIDPWVYAISHPKYRQELQRRMPWLQIQEPDDTAST
ATSNTVSSAPAAAPA-
>MS|comp149511_c0
MSLSLDPGPGIAALQAWGGQVAAYGASNQTVVDKVPPDMLHMVDPHWYQFPPMNPLW
HGLLGFTIGCLGFISITGNGMVIYIFMSTKSLKTPSNLLVVNLAFSDFLMMCCMSPAMVVN
CYNETWVWGPLACELYACAGSLFGCASIWTMTMIAFDRYNVIVKGIAAKPMTNNGALLR
ILGIWVFSLAWTLAPFFGWNRYVPEGNMTACGTDYLSKDWFSRSYILIYSVFVYFMPLLLI

IYSYFFIVQAVAAHEKGMREQAKKMNVASLRSSEAANTSAECKLAKVALMTISLWFMAW
TPYLVINYTGVFESAPISPLATIWGSLFAKANAVYNPIVYGISHPKYRAALYQRFPSLSCQAS
PDESGSVASGATAVSEEKPAA-
>MS|comp158503_c0
MAENGPGMAALQAWGGQAAVYGAANHTVVDKVPADMLHMVDAHWYQFPPMNPLWH
ALLGFTIGVLGFISITGNAMVIYIFMTAKNLKTPSNLLVVNLAFSDFLMMVAMAPAMVINC
YNETWVFGPFACELYGCAGSLFGCTSIWTMTMIAFDRYNVIVKGIAAKPMTKTGALQRIL
GIWLFSLAWTLAPFFGWNRYVPEGNMTACGTDYLSKDWLSRSYIIVYSVFVYFLPLLLIIY
SYYFIVQAVTAHEKAMRDQAKKMNVASLRSSEAANTSAECKLAKVALMTISLWFMAWTP
YLVINYTGVFESAPISPLATIWGSLFAKANAVYNPIVYGISHPKYRAVLYQKFPALACTVSP
DEAGSIATNATVVADEKSEKPAA-
>MS|comp163867_c0
MATNYTEDIGPMAYPLKMVSKEVVEHMLGWNIPEEHQDLVHDHWRNFPAVNKYYHYVL
ALIYTMLMVTSFLGNGIVIWIFGTSKSLRSPSNMFVINLAVFDMMMMLEMPLLIMNSFYQ
RMVGYQLGCDIYAVLGSLSGIGGAITNAVIAFDRYKTISCPLDGRINRVQAFILIAFTWFWA
LPFTILPALKVWGRFVPEGFLTTCSFDYFTDDQDTKVFVVCIFVWSYAIPMTLICYFYSQLF
GAVRMHERMLSDQAKKMNVKSLAANKEDASRSVEIRIAKVAFTIFFLFVCAWTPYAFVT
MTGAFGDRNLLTPIATMVPAVCCKVVSCIDPWVYAINHPRYRAELQKRLPWMGVRESDP
DSVSTTTSVATAQSTAQPAAEA-
>MS|comp164210_c1
MYNQTENSYYDAHFAPFKSGGPSGVEMLGEGLTGDELAAVPEHWLSFPAPPASAHTALAL
LYIFFTAAALVGNGLVIFIFSTTKSLRTSSNLLILQLAILDFIMMAKAPIFIYNSAMRGFASGT
FGCQLFALMGAYSGIGAGMTNACIAYDRHSTITRPLDGRLSRGKVLLMIAFVWIYSTPWA
LLPLFKIWGRYVPEGYLTSCTFDYLTNTFDTKLFVACIFACSYVFPMSMIIYFYSGIVKQVFA
HEAALREQAKKMNVESLRANQNASAESAEIRIAKAALTVCFLFVASWTPYGVMALIGAF
GNQQLLTPGVTMIPAVACKAVACVDPWVYAISHPKYRQELQRRMPWLQIQEPDDSVSTAT
SNTVSSAPPAAPA-
>MS|comp165164_c0
MQTLFEDLEDECPWYYFDGSYKLLLGACLFIFGVFGVLLNGWLFATFIHSHLLVFRSHILV
LNLCTASLGRNLVGFPFAGSSAVAKRWLFGPSCCQLFAFLNQFFGVFQMTALFVAVLERYL
LANYYRRERTLYTRFYWSLVGLSWFNSVIFSTPPLFGYGKYSCDSTGTTCTFLWPSTHSGA
KHIGFSIPYILVCGVIPVIAVFYYMGKAVRLERIFYKGEVQKEQTRLTQTIHAVCVATLALWI
PAAILVGWQWLPLLIYGYRPHVPPALAVIAPIASEAATSVPVLCFIAGDERLRAALLGRMR
KHYALLQPERAKRYNRA-
>MS|comp156702_c0
LDDLKQWKRRRRSADNFKKKLNDESGILKVTVGAVKELASYQNSVDIVETTALSVSDNK
LALIAKFIERWPVERWRSLGLFSDDYLLRINSHWLEFPPPDSWIQYTLGSTYVLLAGTGAF
GNVLVLLMYIRCRSLRTPGNILVANLALSDFLMLAKTPIFIFNSFNLGPALGKTGCVIYGFV
GGLTGTTSIATLSAIALDRYWAVVRPLEPLRALTAIRARLLAIGAWLYACIFAIIPALNIGYGS
YVPEGYLTSCSFDYLTEDFSPRCFIFVFFCAAWLAPFCTITFCYISIFRVVVFNRSMSYGNQD
QRLSSRHVKERTKRKAEIKLAFLVMAVIALFFISWTPYAVVALLGITGRKDLVTPITSMIPAL
FCKTAACINPFIYIITHPKFRKEFKKMLYRDKSKRMRGTLRTTDYCTDTTRMHRPSKDLSD
TDVEIVEMKDIPYQTDEKRDVKIKTISSKVAERQDSQTSMQTIKSLQDSVVSPPSWYMKPQ
FKKKRSFHRRSTRTNSSQTSTSDPII-

>MS|comp41007_c0
VESLRANQNAGAESAEIRIAKAALTVCFLFVASWTPYGVMALIGAFGNQQLLTPGVTMIPA
VACKAVACIDPWVYAISHPKYRQELQRRMPWLQIQEPDDTASTATSNTVSSAPAAAPA-
>MS|comp44349_c0
ENSYYDAHFAAYKSGGAGDTEMLGAGLTGEDLAMVPEHWLSFPAPPASAHTALALLYIFF
TAAALVGNGLVIFIFSTTKSLRTSSNLLILQLAILDFIMMAKAPIFIYNSAMRGFATGTFGCQ
LFALMGAY-
>Pxylopsin-BL
MATNYTMADYGIVAQPMKMVSQEVVEHMLGWNIPEEHQDLVHMHWRAFPAVSKFWHY
GLALIYTCLMLTSLTGNSIVIWIFSTSKSLRTASNMFVINLAVFDLMMMIEMPLLIMNSFQQ
RMVGYQLGCDIYATLGSLSGIGGAISNAVIAFDRYKTISCPLDGRINKVQAALLILFTWLWS
VPFTALPLFKIWGKFVPEGFLTTCSFDYFTDDQDTKVFVAAIFVWSYCIPMSLICYFYSQLF
GAVRLHERMLKDQAKKMNVKSLASNKDDAGKSVEIRIAKVAFTIFFLFVCGWTPYAFVA
MTGAFGDRSLLTPVATMVPAVCCKIVSCIDPWVYAINHPRYRAELQKRLPWMGVREADPD
SVSTNTSLATAQAANPEA-
>Pxylopsin-LW
MGFANMDPGPGMAALQAWGGQVAAYGASNQTVVDKVLPDMIHMIDPHWYQFPPMNPL
WHGLLGFTIGVLGFVSITGNGMVIYIFTSTKSLKTPSNLLVVNLAFSDFLMMCCMSPPMV
VNCYNETWVFGPLACELYACAGSLFGCASIWTMTMIAFDRYNVIVKGIAAKPMTINGALL
RILGIWLFSLAWTLAPFFGWNKYVPEGNMTACGTDYLSKDWFSRSYILVYAVFVYFMPLL
LIIYSYFFIVQAVAAHEKGMREQAKKMNVASLRSSEAANTSAECKLAKVALMTISLWFMA
WTPYLVINFTGVLDGAPLTPLATIWGSLFAKANAVYNPIVYGISHPKYRAELYKKFPSLAC
QASPDESGSVASGTTTATEEKPAA-
>Pxylopsin-Rh3
MRLTHASAVITTLIITLQLSQTKNVTNNPCDTCACAVKSDASCENCFKVELDSLKQWKRR
RRLCRVERSFDSDMLDVPNMVGSSSRHQPVLATSLSTVAKFKQQWPVELWRQYLFTDDY
LLLINPHWLKFPPPHAAVNYSMGALYILMMLAGGTGNGLVLVMYLRCRSLRTPGNILVAS
LALSDFLMLAKTPVFIFNSFNLGPALGKTGCLIYGFLGGLTGCTSIATLAAIALDRYWAIAR
PLEGLGTLTAVRARVLAVAAWLYAALFASVPAFDIGYGRYVPEGYLTSCSFDYLTDEVKPR
YFIFVFFCAAWVTPFATITFCYINILRVVICKRNVSTTSSNDHRLSTRHVKEQAKKKAEMKL
AVLVIAVIALFFVSWTPYAIIALMGIFGNRDLITPTSSMIPALFCKTAACINPFVYIITHPKFRK
EIKNLFSKGKSRQYGGTMRTIGYTTDATTYHRPSKDSSETDVEVVEMKDIPFQGEGKRGK
EGVKSVSGRLSEREGSQRSINESMKSYEGDVVSPPSWFRQPQFSKKRSFHRRSTRSVVSFD
TNAVL-
>TcasPteropsin
MANTLLLICCLALLASAIALPATNDQLDKVTVNDVAANAVSPESKTDDSNDKRDAATVEIT
GITTGVETPDPSSTPFHPQRAGSMFARFIDDIFQIPINVLQNVARLITSPFVQNKKTPEASAA
SLKSQFRMKNFNSTEIGDELLIPVEGYIAAAVVLFCIGFFGFSLNLTVIIFMLKERQLWSPLN
IILFNLVVSDFLVSVLGNPWTFFSAINYGWIFGETGCTIYGFIMSLLGITSITTLTVLAFERYL
LIARPFRNNALNFHSAALSVFSIWLYSLSLTIPPLIGWGEYVHEAANLSCSVNWEEKSPNST
SYILYLFAFGLFLPLVIITFSYVNIILTMRRNAAFRVGQVSKAENKVAYMIFIMIIAFLTAWSP
YAIMALIVQFGDAALVTPGMAVIPALLAKSSICYNPVIYIGLNAQFQQAWMQKWKKNRRG
SDALGTSRVMLETIHQACRDEKTDKLLEKKTKFCKDFETDVSML-
>Tcasopsin-UV

MLHNLTATPFHQARTMETMANHLGWNVPKDELIHIPQHWLVYPEPEASMHFLLALIYIGF
FIMATIGNGLVIWIFSTSKSLRTASNMFVVNLAICDFAMMIKTPIFIYNSFYRGFALGHLGCQ
IFAFIGSLSGIGAGMTNACIAYDRYTTITRPFDGKITRTKALVMIIFVWGYTIPWAVMPLLEI
WGRFAPEGFLTACSFDYLTDTFDNHMFVTSIFICSYVIPMSMIIYFYSQIVSKVFSHEKALRE
QAKKMNVESLRSNQSQQASQSAELRIAKAAIAICSLFVASWTPYAVLALIGAFGDQSLLTP
GVTMVPACACKFVACLDPYVYAISHPKYRLELQKRLPWLAIKETAASETQSTTTENTTTQS
ATTTT-
>Tcasopsin-LW
MSVMGEPNFIAWAAQRSGYGGGNLTVVDKVLPDMLHLVDAHWYQFPPMNPLWHGILGF
VIGVLGFVSIVGNGMVIYIFSSTKALRTPSNLLVVNLAFSDFLMMLCMSPAMVINCYNETW
VLGPLVCELYGMSGSLFGCASIWTMTFIALDRYNVIVKGLSAQPLTKKGAMLRILIIWVFS
TLWTIAPFFGWNRYVPEGNMTACGTDYLTKDWVSRSYILVYAVWVYFVPLFTIIYSYWFIV
QAVAAHEKSMREQAKKMNVASLRSSEAAQTSAECKLAKIALMTITLWFFAWTPYLVTNFT
GIFEGAKISPLATIWCSLFAKANAVYNPIVYGISHPKYRQALQKKFPSLVCAGEPDDTTSTA
SGVTNVTTDEKPATA-
>Agamopsin1
MAAFVEPHFDAWTQGSGNMSVVDKVPPEMLHMVHPHWNQFPPMNPLWHSILGFAIFML
GMISMTGNGCVMYIFTNTKSLRTPSNLLVVNLAFSDFFMMFTMGPPMVINCWHETWTFG
PFACELYAMLGSLFGCASIWTMTMIAFDRYNVIVKGLAGKPMTNNGALLRILGVWVFALF
WTLAPLFGWNRYVPEGNMTACGTDYLTQTWLSRSYIIIYAIFVYWLPLLTIIYSYTFILKAV
SAHEKNMREQAKKMNVASLRTQEAQNTSTEMKLAKVALVTISLWFMAWTPYLVINFTGIF
KAAPISPLATIWGSLFAKANAVYNPIVYGISHPKYRAALYQKFPSLSCQDAPVDDGQSVAS
GATQASDEKA-
>Agamopsin2
MAAFVEPHFDAWTQSGGNMSVVDKVPPEMLHMVHPHWNQFPPMNPLWHSILGFAIFML
GMISMTGNGCVMYIFTNTKSLRTPSNLLVVNLAFSDFFMMFTMGPPMVINCWHETWTFG
PFACELYAMLGSLFGCASIWTMTMIAFDRYNVIVKGLAGKPMTNNGALLRILGVWVFALF
WTLAPLFGWNRYVPEGNMTACGTDYLTQTWLSRSYIIIYAIFVYWLPLLTIIYSYTFILKAV
SAHEKNMREQAKKMNVASLRTQEAQNTSTEMKLAKVALVTISLWFMAWTPYLVINFTGIF
KAAPISPLATIWGSLFAKANAVYNPIVYGISHPKYRAALYQKFPSLSCQDAPVDDGQSVAS
GATQASDEKA-
>Agamopsin3
MAAFVEPHFDAWTQSGGNMSVVDKVPPEMLHMVHPHWNQFPPMNPLWHSILGFAIFML
GMISMTGNGCVMYIFTNTKSLRTPSNLLVVNLAFSDFFMMFTMGPPMVINCWHETWTFG
PFACELYAMLGSLFGCASIWTMTMIAFDRYNVIVKGLAGKPMTNNGALLRILGVWVFALF
WTLAPLFGWNRYVPEGNMTACGTDYLTQTWLSRSYIIIYAIFVYWLPLLTIIYSYTFILKAV
SAHEKNMREQAKKMNVASLRTQEAQNTSTEMKLAKVALVTISLWFMAWTPYLVINFTGIF
KAAPISPLATIWGSLFAKANAVYNPIVYGISHPKYRAALYQKFPSLSCQDAPVDDGQSVAS
GATQASDEKK-
>Agamopsin4
MLKTEVLIFPDMAAFVEPHFDAWTQSGGNMSVVDKVPPEMLHMVHPHWNQFPPMNPL
WHSILGFAIFMLGMISMTGNGCVMYIFTNTKSLRTPSNLLVVNLAFSDFFMMFTMGPPMV
INCWHETWTFGPFACELYAMLGSLFGCASIWTMTMIAFDRYNVIVKGLAGKPMTNNGAL
LRILGVWVFALFWTLAPLFGWNRYVPEGNMTACGTDYLTQTWLSRSYIIIYAIFVYWLPLL

TIIYSYTFILKAVSAHEKNMREQAKKMNVASLRTQEAQNTSTEMKLAKVALVTISLWFMA
WTPYLVINFTGIFKAAPISPLATIWGSLFAKANAVYNPIVYGISHPKYRAALYQKFPSLSCQ
DAPVDDGQSVASGATQASDEKA-
>Agamopsin5
MMDHRPVGIFGPKSPQALTWTISVANLTVVDKVPPEMLHLVDTYWYQFPPLETKWHAAL
ATTIGLLALISIVGNGCVILIFSSTKGLRTPSNLMVINLAFADFMMMFTMAPPLIINSYHETW
VFGPLMCEIYGMFGSLSGCVSIWSMTMIAFDRYTVIVKGLSAKPLTYVGSVMRILFVWAN
SLVWTLAPLFGWNRYVPEGNMSACGTDYLSKDWISVSYIYAYSVFVYWLPLLLIIYCYTYI
LKAVSAHERNMREQAKKMNVASLRSSDASKTNAEIKLAKVALVTITLWFMAWTPYLVIN
YAGIIDSSPISPLQTIWGSVFAKANAVYNPIVYGISHPKYRAALYKRFPALQLRKENATDQN
SVASCTTAADQCET-
>Agamopsin6
SVDNRTPLCLVVGNCQRRETEMAAFAEPHFSAWTQTVVSNVTVVDKVPPEMLHMVDAH
WYQFPPMNPLWHSILGFAIFVLGVVSIIGNGCVIYIFTNTKALRTPSNLLVVNLAFSDFLMM
FTMAPPMVINCWHETWVFGPFACELYAMLGSLFGCASIWTMTMIAFDRYNVIVKGLAGK
PMTNNGALLRILGIWAFSLLWTLAPLFGWNRYVPEGNMTACGTDYLSQDFTSRSYILIYSG
FVYYLPLFSIIYSYIYIIQAVSAHEKNMREQAKKMNVASLRSQEAQNTSTEMKLAKVALVTI
SLWFMAWTPYLVINYTGIFKAAPISPLATIWGSLFAKANAVYNPIVYGISHPKYRAALYQKF
PSLSCQDNSDDGQSVASAATGVSEEKPAA-
>Agamopsin7
MPYYGPMQQPGLWGQPVANLTVVDKVPPEIMHLVDPHWSQFPPMNPLWHSIIGFVIFVLG
VVSIIGNGMVIYIFSTAKSLRTPSNLFIVNLALSDFLMMGTNAFTMVYNCWFETWSLGLL
MCDLYAFFGSLFGCCSIWTMTMIALDRHNVIVHGLSGKPLTNTGAILRILLCWLIGVVWGI
LPMLGWNRYVPEGNMTACGTDYLTDDWFHKSYILVYSVFVYYTPLFTIIYAYFFIIKAVSA
HEKNMREQAKRMNVQSLRSSDDGKSTEMKLAKVALVTISLWFMAWTPYTVINYTGVFK
TASITPLATIWGSVFAKANAVYNPIVYGISHPKYRAALLRRFPSLACSDGPPADDKSLASEA
SGITSAGNPTTA-
>Agamopsin8
MGLVQLDNQTAYRPEALIGADQSGLRYLGWNVPPEELVHIPEHWLQFPEPEASLHYLLGL
LYIAFTIFSLVGNGLVIWIFIAAKSLRTPSNVFVINLAICDFFMMAKTPIFIYNSFTKGFTLGN
LGCQIFGFVGSLTGIGAGATNALIAYDRYNTITRPFEGRLTQTKAIIFICLIWAYTIPWGVLPL
LEIWGRYVPEGFLTSCTFDYLSGTFDTRLFVASIFTFSYVLPMSLIIYYYSQIVSHVVNHEKS
LREQAKKMNVESLRSNQNQKDASVEIRIAKAAITVCFLFVASWTPYAVLALIGAFGDKSLL
TPGVTMFPACACKFVACLDPYVYAISHPRYRIELQKRLPWLAITETLPAENASTCTEQQDG
NATTQS-
>Agamopsin9
MFLGNESISEGAMLMPMARTAGEMPKLLGWNLPPEEQYLVHDHWKGFPSPPYYMHLML
AMIYFVLMNTSLIGNGIVLWIFGTSKSLRNGSNMFIINLAIFDLLMMCEMPMFLVNSFSERL
VGYGVGCSVYAALGSMSGIGGAISNAVIAFDRYRTISNPLDGRLSRVQAGLLICLTWLWTM
PFTLLPLFEIWGRYIPEGYLTTCSFDYLTDDPDTRVFVGCIFTWAYVIPMIFICYFYARLFGH
VRQHEMMLKNQARKMNVESLTANRSEKAQAVEMRIAKAAFTIFFLFVCAWTPYAIVTMI
GAFGDRTMLTPFVTMVPAVCCKIVSCLDPWVYAISHPKYRQELERRLPWMGIKEADDSVS
TTESKATVVVDGAAGNGGDNAAN-
>Agamopsin10

KYVVRSGYVLPVDPLFVAKINPFWLRFDPPSAGEHYGLAVFYFLMMLFGVIGNALVVFMF
YRYRSLRTPANYLVINLAVADFIIMMEAPMFIYNSIHQGPALGSIGCTVYALMGAVGGTVAI
ATLTVISIDRYNVVVYPLNPNRSTTKLKCYFLIAFTWAYGLLFASFPALEIGLSRYTAEGYLT
ACSFDYLDRTYKARVFMFVYFVFAWLIPFAIISYCYARILIAVINANAIQSSKSKNKTEVKLA
GVVVGIIGLWFAAWTPYAVVAMMGVFGYEQYLTPLNSMIPAVFAKIAASIDPYFYAMNHP
RYRQMLERMFCNRGADQGNSQYQTSHYTRGASRGGDSEGGGGEESGGGGGVGRAPGG
GNAGLGRGGTVRGGGGGGGRLIAGKGGGGANATGSTGGGGVKALKKQISNGDETSLEVS
LEM-
>Amelopsin-BL
MLLHNKTLAGKALAFIAEEGYVPSMREKFLGWNVPPEYSDLVHPHWRAFPAPGKHFHIG
LAIIYSMLLIMSLVGNCCVIWIFSTSKSLRTPSNMFIVSLAIFDIIMAFEMPMLVISSFMERMI
GWEIGCDVYSVFGSISGMGQAMTNAAIAFDRYRTISCPIDGRLNSKQAAVIIAFTWFWVTP
FTVLPLLKVWGRYTTEGFLTTCSFDFLTDDEDTKVFVTCIFIWAYVIPLIFIILFYSRLLSSIRN
HEKMLREQAKKMNVKSLVSNQDKERSAEVRIAKVAFTIFFLFLLAWTPYATVALIGVYGN
RELLTPVSTMLPAVFAKTVSCIDPWIYAINHPRYRQELQKRCKWMGIHEPETTSDATSAQT
EKIKTDE-
>Amelpteropsin
MSLNRSTMEHVIYEDQVSPVMYIGAAIALGFIGFFGFTANLLVAIVIVKDAQILWTPVNVIL
FNLVFGDFLVSIFGNPVAMVSAATGGWYWGYKMCLWYAWFMSTLGFASIGNLTVMAVER
WLLVARPMQALSIRHAVILASFVWIYALSLSLPPLFGWGSYGPEAGNVSCSVSWEVHDPV
TNSDTYIGFLFVLGLIVPVFTIVSSYAAIVLTLKKVRKRAGASGRREAKITKMVALMITAFL
LAWSPYAALAIAAQYFNAKPSATVAVLPALLAKSSICYNPIIYAGLNNQFSRFLKKIFDARG
SRTAVPDSQHTALTALNRQEQRK-
>Amelopsin-LW2
MDTLNITTSFFIEVMPSNISTLTTTGPQFARQLMRFNNQTVVSKVPEEMLHLIDLYWYQFPP
LDPLWHKILGLVMIILGIMGWCGNGVVVYVFIMTPSLRTPSNLLVVNLAFSDFIMMGFMC
PPMVICCFYETWVLGSLMCDIYAMVGSLCGCASIWTMTAIALDRYNVIVKGMSGTPLTIK
RAMLQILGIWLFGLIWTILPLVGWNRYVPEGNMTACGTDYLSQDWTFKSYILVYSFFVYY
TPLFTIIYSYYFIVSAVAAHEKAMKEQAKKMNVTSLRSGDNQNTSAEAKLAKVALTTISLW
FMAWTPYLVINYIGIFNRSLITPLFTIWGSLFAKANAIYNPIVYGISHPKYRAALKEKLPFLV
CGSTEDQTAATAGDKASEN-
>Amelopsin-LW1
MIAVSGPSYEAFSYGGQARFNNQTVVDKVPPDMLHLIDANWYQYPPLNPMWHGILGFVI
GMLGFVSVMGNGMVVYIFLSTKSLRTPSNLFVINLAISDFLMMFCMSPPMVINCYYETWV
LGPLFCQIYAMLGSLFGCGSIWTMTMIAFDRYNVIVKGLSGKPLSINGALIRIIAIWLFSLG
WTIAPMFGWNRYVPEGNMTACGTDYFNRGLLSASYLVCYGIWVYFVPLFLIIYSYWFIIQ
AVAAHEKNMREQAKKMNVASLRSSENQNTSAECKLAKVALMTISLWFMAWTPYLVINFS
GIFNLVKISPLFTIWGSLFAKANAVYNPIVYGISHPKYRAALFAKFPSLACAAEPSSDAVSTT
SGTTTVTDNEKSNA-
>Amelopsin-UV
MSNDSIHWEARYLPAGPPRLLGWNVPAEELIHIPEHWLVYPEPNPSLHYLLALLYILFTFLA
LLGNGLVIWIFCAAKSLRTPSNMFVVNLAICDFFMMIKTPIFIYNSFNTGFALGNLGCQIFA
VIGSLTGIGAAITNAAIAYDRYSTIARPLDGKLSRGQVILFIVLIWTYTIPWALMPVMGVWG
RFVPEGFLTSCSFDYLTDTNEIRIFVATIFTFSYCIPMILIIYYYSQIVSHVVNHEKALREQAK

KMNVDSLRSNANTSSQSAEIRIAKAAITICFLYVLSWTPYGVMSMIGAFGNKALLTPGVT
MIPACTCKAVACLDPYVYAISHPKYRLELQKRLPWLELQEKPISDSTSTTTETVNTPPASS-
>Ceraopsin-LW
MAITSMDPAPGVAAMQAWGGHAEAYSANQTVVDKVLPEMLHLIDAHWYQFPPMNPLW
YGLLGFTICTLAFISVTGNGMVIYIFTTTKSLKTPSNMLVVNLAFSDFLMMAMMAPPLCIN
SYYQTWVFGPVACQFYACFGSLFGCVSIWTMTAIAFDRYNVIVKGIAAKPLTVNGALLQIF
GVWAFSLAWTLAPIFGWSRYVPEGNMTACGTDYLSKDLLSQIYIITYSIACYFLPLALIVYS
YFFIVQAVAAHEKAMREQAKKMNVASLRSSEQANTSAECKLAKVALMTISLWFMAWTPY
LVINFAGVFETAPISPLSTIWGSVFAKANAVYNPIVYGISHPKYRAALYQRFPSLACQPSPSE
ETGSVASAATACTEEKPSA-
>Ceraopsin-V2
MAHNHTVDHGPLAYPFKMVSAEIQEGMLGWNIPPEHQDLVHEHWRQFPAVDKSSHYLLA
LIYATLTIFSITGNGLVIWIFGTSKSLRSASNMFIINLAIFDLTMMLEMPHLIVNSFYQRMLGN
RLGCDIYSVFGSLSGIGGAITNAVIAYDRYKTISCPIDGRINRVQASILIAITWLYALPFTIVPM
TGAWGRYVPEGFLTTCSFDFLSEDPDTKTFVACIFVWSYVIPMSLICTFYSKLFIAVRHHEK
MLKEQAKKMNVKSLASNKDDGGKSVEIRIAKVAFTIFFLFICSWTPYGIVAMIGAFGDRAL
LTPVVTMIPAVFCKSVSCIDPWVYAINHPRFRVELEKRVPWMGVREPDPDAQSTNASTATT
PAEA-
>Ceraopsin-V1
MAENFTENEPIAFPYKMVSHEIQKHMLGWNIPQEHQHLVHEHWRQFPDVDRSSHYLLALI
YTILTISSVTGNGLVIWIFSTSKSLRTPSNMFVINLALFDLAMMGEMPHLILNSFYQRMLGY
ELGCDIYAVLGSLSGIGGAMTNAVIAYDRYKTISSPIDGRINRAQATLLVIFTWLYAMPFTVF
PLTKTWGRFVTEGFLTTCSFDFLSDDVKTFVLCISIWSYVIPMTLICTFYLKLFSAVRHHER
MLKEQAKKMNVKSLASNKEDAGKSVEIRIAKVAFTIFFLFICSWTPYGIVATIGAFGDRSLL
TPVVTMIPAVFCKAVSCLDPWVYAINHPRFRVELEKRVPWMGVREPDPDAQSSTGSAVTN
SAEA-
>Ceraopsin-UV
MIPTQDMDNRTEYYNIYGAYFAPLRSGGERMLGEGLTGEDLAMIPEHWFKYPEPPASAHT
ALCLLYCFFTAAALIGNGMVIFIFASTKSLRTASNLLILNLAIFDFIMMLKAPIFIYNSAMRG
FAAGALWCKIFAVMGSYSGIGAAMTNACIAYDRHSTITRPLDGRLSRGKVLLMIICIWIYTT
PWSLMPLFRVWGRFVPEGYLTSCTFDYLTNSFDNKLFVGCIFTCSYVFPMLCIIYFYSGIVK
QVFAHEAALREQAKKMNVESLRSNQNAGAESAEIRIAKAALTVCFLFVASWTPYGVMSLI
GAFGDQELLTPGVTMIPAITCKAVACIDPWVYAISHPKYRQELQRRMPWLQISEPDDNTSN
TSNTTTNSAAPA-
>Ceraopsin-BL
MFNNVNDFSAEAGPVAYPFKMVSDEVQQNMLGWNVPSEYQDLVHDHWRNFAAIDKFW
HYCLALIYMILWISSVCGNGIVVWIFSTSKSLRSASNMFIVNLALFDMVMMIEMPHLIINSF
YQKMIGYQVGCDIYATLGSISGIGAAISNAVIAYDRYKTISCPIDGRINKVQASILIAFTWFW
TLPFTILPLLKVWGKFVPEGFLTTCSFDYFTDDEDTKVFVACIFVWSYCFPMALICIFYSKL
FTAVRLHEKMLKEQAKKMNVKSLASNKEDAGKSVEIRIAKVAFTIFFLFVCAWTPYAIVT
MIGAFGDRSLLTPHVTMIPAVFAKSVSCIDPWVYAINHPRYRAELQKRLPWMGVREPEPET
QSSNASTTTQSQSAEA-
>Pglaopsin1
MALDSLDPAATFGHAWAGKMEAYGSNQTVIDQVLPEMIHLIDPHWYQFPPMNPLWHGLL

GFVIAVLGFISLSGNGMVIYIFTTTKTLKTPSNLLVLNLAVSDFLMMTCMAPPLVVNSYHET
WVFGPLACALYAAAGSLFGTISIWTMTMIAFDRYNVIVKGIAAKPMTNNGALLRILAIWV
SSLAWTVAPMFGWNRYVPEGNMTACGTDYLNKDWFSRSYIVAYAIFCYFTPLALIIYSYFF
IIQAVAAHERAMREQAKKMNVASLRSSEAANTSAECKLAKVALMTISLWFMAWTPYLVIN
FTGIFETATISPLGTIWGSVFAKANAVYNPIVYGISHPKYRAALYQRFPSLACQPAADDNTS
QASGKTTVCEEKPSA-
>Pglaopsin2
MAIANLDPGLGAAAEVWGGQAAAFSSNQTVVDKVSPDMMHLIDPHWYQFPPMNPMWH
GLLGFTIGVLGFISITGNGMVVYIFTSTKSLKTPSNLLVVNLAFSDFLMMLCMAPPMVVNC
YYETWVFGPLACELYACAGSLFGSISIWTMTMIAFDRYNVIVKGIAAKPMTINGALLRILGI
WLFSLAWTIAPIFGWNRYVPEGNMTACGTDYLNKSWLSRSYILVYSIFVYYMPLLLIIYSY
FFIVQAVAAHERAMREQAKKMNVASLRSSEAANTSAECKLAKVALMTISLWFMAWTPYL
VINYTGVFETAAISPLATIWGSVFAKANAVYNPIVYGISHPKYRAALYQKFPSLACQPSPEE
TGSVASGATTACEEKPSA-
>Pglaopsin3
MALDYLNTGAAKMGTWNGQMSAYGANQTVVDKVLPEMLHLIDPHWYQFPPMNPLWY
GLLGFTIACLAITSITGNAMVIYIFTTTKNLKTPSNLLVVNLAVSDFLMMACMAPPLIINSY
NETWVFGPLFCAIYACGGSLYGTVSIWTMTAIAFDRYNVIVKGIAAKPMSINGALLRILAI
WLSSLAWTVAPIFGWNRYVPEGNMTVCGTDYLSKDMLSRSYIIAYAVFCYFLPLGLIIYSY
WFIIQAVAAHEKAMREQAKKMNVASLRSSDAANTSAECKLAKVALMTISLWFMAWTPYL
VINFAGVFETAPISPVSTIWGSVFAKANAVYNPIVYGISHPKYRAALYQRFPSLACQPSPDES
GSVASGNTAVCEEKPPA-
>Pglaopsin4
HLIDPHWFQFPPMNPLWHGLLGFTIGVLGFISMVGNGMVIYIFSATKNLKTPSNLLVVNLA
FSDFLMMFTMSPAMVVNCYNETWVFGPFACELYGCAGSLFGCISIWTMTMIAFDRYNVIV
KGIAAEPMTKKGALLRILFVWAASLAWTLAPLFGWNRYVPEGNMTACGTDYLTKDWFSR
SYILVYGFFVYFAPLFLIIYSYYFIVKAVAAHEKSMREQAKKMNVASLRSSESANTNAECK
LAKVALMTISLWFLAWTPYLIINFMGIFESMPISPLTTIWGSLFAKANAVYNPIVYGISHPRY
RAALYKKFPSLSCQAASDEVDSVASNVTAVSNEKPAA-
>Pglaopsin5
MIAPAAMDNHTENNYNYGAYFAPYRSDEPVEMLGAGLTGADLAAIPEHWLAYPAPPASA
HTMLALVYVFFTAAALIGNGLVIFIFSASKSLRTPSNLLVVQLAILDFLMMLKAPIFIYNSIK
RGFAAGVIGCQIFAFMGSVSGTAAGLTNACIAYDRHSTITRPLDGRLSRGKVLLMMVCVW
VYTAPWAILPQLQIWGRYVPEGFLTSCTFDYLTTTFDNKLFVASMFVCVYVFPMLAIMYFY
SGIVKQVFAHEAALREQAKKMNVDSLRSNQNASAESAEIRIAKAALTVCFLYVASWTPYG
VMSLIGAFGDQNLLTPGVTMIPALACKGVACIDPWVYAISHPKYRQELQKRMPWLQIDEP
DDNVSNTTNNTANSSAPA-
>Pglaopsin6
MAANYSDDIGPMAYPMKLVSSEIVEHMMGWNIPEEHQAMVHAHWRSFPAVSKYYHFILA
LIYTMLMVTSLIGNGIVIWIFSTSKSLRSASNMFVINLAVFDLMMMIEMPLLIANSFYQHPI
GFQLGCDIYAVLGSISGIGGAITNAVIAFDRYKTISCPLDGRINKVQASLLIVFTWFWSLPFTI
LPALKVWGRFVPEGFLTTCSFDYFTDDQDTKVFVACIFVWSYAIPMALICYFYSQLFGAVR
LHEKMLQEQAKKMNVKSLASNKEDASKSVEIRIAKVAFTIFFMFVCGWTPYAIVTMTGAY
GDRSLLSPVATMIPAVCCKIVSCIDPWVYAINHPRYRAELQKRLPWLGVREQDPDSVSTSN

SVTTTQSHTPNAET-
>Pxutopsin-UV
MIPAAVMDNHTENNYNYGAYFAPYRLEGVELLGAGLTGEDLAAIPEHWLSYPAPPASAHT
MLALVYVFFTAAALIGNGLVIFIFSASKSLRTPSNLLVVQLAVLDFLMMLKAPIFIYNSIKRG
FASGVIGCQIFAFMGSVSGTAAGLTNACIAYDRHSTITRPLDGRLSRGKVLLMMVCVWLY
TAPWAILPQLQIWGRYVPEGFLTSCTFDYLTTTFDNKLFVASMFVCVYIFPMIAILYFYSGIV
KQVFAHEAALREQAKKMNVDSLRSNQNAAAESAEIRIAKAALTVCFLYVASWTPYGVMS
LIGAFGDQNLLTPGVTMIPALACKGVACIDPWVYAISHPKYRQELQKRMPWLQIDEPDDN
ASNTTSNTANSSAPA-
>Pxutopsin-BL
MAANYSDDIGPMAYPMKLVSSEMVEHMMGWNIPEEHQAMVHAHWRSFPAVSKYYHFIL
ALIYTMLMVTSLVGNGIVIWIFSTSKSLRSASSMFVINLAVFDLMMMIEMPLLIANSFYQHP
IGFQLGCDVYAVLGSISGIGGAITNAVIAFDRYKTISCPLDGRINKVQASLLIAFTWFWSMPF
TILPALKVWGRFVPEGFLTTCSFDYFTDDQDTKVFVACIFVWSYAIPMALICYFYSQLFGAV
RLHERMLQEQAKKMNVKSLASNKEDASKSVEIRIAKVAFTIFFMFVCGWTPYAFVTMTG
AYGDRSLLTPVATMIPAVCCKIVSCIDPWVYAINHPRYRAELQKRLPWLGVREQDPDTVSN
SNSVTTTQSHTPTAEA-
>Pxutopsin-LW1
MAMDSLDPGAASAPAWAGKIEAYGSNHTVIDQVLPEMLHLIDPHWYQFPPMNPLWHGLL
GFVIAVLGFISLSGNGMVIYIFTTTKTLKTPSNLLVLNLAVSDFLMMTCMAPPLVVNSYHET
WVFGPLACALYAAAGSLFGTISIWTMTMIAFDRYNVIVKGIAAKPMTNNGALLRILAIWV
SSLAWTVAPMFGWNRYVPEGNMTACGTDYLNKDWFSRSYIVAYAIFCYFTPLALIIYSYFF
IIQAVAAHEKAMREQAKKMNVASLRSSEAANTSAECKLAKVALMTISLWFMAWTPYLVIN
FTGIFETATISPLGTIWGSVFAKANAVYNPIVYGISHPKYRAALYQRFPSLACQPAADDNTS
QVSGKTAVCEEKPSA-
>Pxutopsin-LW2
MAIANLEPGMGASEAWGGQAAAFGSNQTVVDKVTPDMMHLIDPHWYQFPPMNPMWH
GLLGFTIGVLGFISITGNGMVVYIFTSTKSLKTPSNLLVVNLAFSDFLMMLCMAPPMLINC
YYETWVFGPLACELYACAGSLFGSISIWTMTMIAFDRYNVIVKGIAAKPMTINGALLRILGI
WLFSLAWTIAPMLGWNRYVPEGNMTACGTDYLSKSWLSRSYILVYSIFVYYTPLLLIIYSY
FFIVQAVAAHEKAMREQAKKMNVASLRSSEAANTSAECKLAKVALMTISLWFMAWTPYL
VINYTGVFETAPISPLATIWGSVFAKANAVYNPIVYGISHPKYRAALYQKFPSLACQPSAEE
TGSVASGATTACEEKPSA-
>Pxutopsin-LW3
MALNYLNTGAAKMDTWNGQMSAYGANQTVVDKVLPEMLHLIDPHWYQFPPMNPLWY
GLLGFTITCLAITSITGNAMVIYIFTTTKNLKTPSNLLVVNLAVSDFLMMACMAPPLIINSY
NETWVFGPLFCAIYACGGSLYGTVSIWTMTAIAFDRYNVIVKGIAAKPMSINGALLRILAI
WLSSLAWTVAPIFGWNRYVPEGNMTVCGTDYLSKDWLSRSYIIAYAVFCYFLPLGLIVYSY
WFIIQAVAAHEKAMREQAKKMNVASLRSSDAANTSAECKLAKVALMTISLWFMAWTPYL
VINFAGVFETAPISPVSTIWGSVFAKANAVYNPIVYGISHPKYRAALYQRFPSLACQPSPDES
GSVASGNTAVCEEKAPA-
>PxutopsinRh3
YLYFRCRTLRTPGNILVANLALSDFIMLAKTPIFIFNSFNLGPALGKTGCVIYGFLGGLTGTT
SIATLTAIALDRYWAVVRPLEPLTALTAVRARLLAVGAWTYAAVFSAVPAFDFGYGHYVPEG

YLTSCSFDYLTEDLRPRYFIFAFFCAAWLVPFCTIFFCYTSILQVVVCKRNMPSKNQEQRLS
SRHVKEQTKRKAEIKLAFLVIVVIALFFISWTPYAIVALLGIFGKKNLITPTASMIPALFCKTA
ACINPFIYIITHPKFRKELQKIIYRDKSKRMSGTLRTTGYYTDSSRMHRPSKDLSDTDVEILE
MKDIPFRTGLNRNTSYNLETISSRVSEREGSQRSASLKSFEDSVVSPPSWYSKPQFSKKRSF
QRRSSQKSGQ-
>Pglaopsin-LW1
MALDSLDPAATFGHAWAGKMEAYGSNQTVIDQVLPEMIHLIDPHWYQFPPMNPLWHGLL
GFVIAVLGFISLSGNGMVIYIFTTTKTLKTPSNLLVLNLAVSDFLMMTCMAPPLVVNSYHET
WVFGPLACALYAAAGSLFGTISIWTMTMIAFDRYNVIVKGIAAKPMTNNGALLRILAIWV
SSLAWTVAPMFGWNRYVPEGNMTACGTDYLNKDWFSRSYIVAYAIFCYFTPLALIIYSYFF
IIQAVAAHERAMREQAKKMNVASLRSSEAANTSAECKLAKVALMTISLWFMAWTPYLVIN
FTGIFETATISPLGTIWGSVFAKANAVYNPIVYGISHPKYRAALYQRFPSLACQPAADDNTS
QASGKTTVCEEKPSA-
>Pglaopsin-LW2
MTIASLDPGLGPAMLDVWNGQTAAFGSNQTVVDKVPPDMLHLVDPHWYQFPPMNPMW
HGLLGFTIGVLGFISIAGNGMVVYIFTTTKSLKTPSNLLVVNLAFSDFLMMCFMSPAMVVN
CYYETWVFGPLGCDIYACTGSLFGSVSIWTMTMIAFDRYNVIVKGIAAKPLTINGALLRIL
GIWLFSLLWTVAPIFGWNRYVPEGNMTACGTDYINKSWFSRSYILVYSVFVYYAPLLLIIYS
YFFIVQAVAAHERAMREQAKKMNVASLRSSEAANTSAECKLAKVALMTISLWFMAWTPY
LIINYMGIFETAPISPLTTIWGSVFAKANAVYNPIVYGISHPKYRAALYQKFPSLACQPSPEES
GSVASGATTACEEKPSA-
>Pglaopsin-LW3
MTIERQEVGAMTMRAWDGAMAPYTSNQTVVDKVLPEMLHLIDAHWYQFPPMDPLWYS
LLGCTIAVLCLISVTGNAMVIYIFTTTKHLKTPSNLLVVNLAVSDFLMMACMAPPLMINSY
NQTWVFGPLFCAIYACGGSLYGTVSIWTMTAIAFDRYNVIVKGIAAKPMTINGALLRIFAV
WLSSLAWTVAPILGWNRYVPEGNMTACGTDYLSKDLLSRSYIVAYAIFCYFLPLALIIYSYY
FIIQAVAAHERAMREQAKKMNVASLRSSDTANTSTECKLAKVALMTISLWFMAWTPYLVI
NFAGIFETAPISPLGTIWGSVFAKANAVYNPIVYGISHPKYRAALYQRFPALACRPSAEENG
SVASANTAVCEEKPAA-
>Pglaopsin-Rh4
HLIDPHWFQFPPMNPLWHGLLGFTIGVLGFISMVGNGMVIYIFSATKNLKTPSNLLVVNLA
FSDFLMMFTMSPAMVVNCYNETWVFGPFACELYGCAGSLFGCISIWTMTMIAFDRYNVIV
KGIAAEPMTKKGALLRILFVWAASLAWTLAPLFGWNRYVPEGNMTACGTDYLTKDWFSR
SYILVYGFFVYFAPLFLIIYSYYFIVKAVAAHEKSMREQAKKMNVASLRSSESANTNAECK
LAKVALMTISLWFLAWTPYLIINFMGIFESMPISPLTTIWGSLFAKANAVYNPIVYGISHPRY
RAALYKKFPSLSCQAASDEVDSVASNVTAVSNEKPAA-
>Pglaopsin-BL
MAANYSDDIGPMGYPMKLVSSEIVEHMLGWNIPEEHQEMVHEHWRNYPAVNKYWHFCL
GFIYSLLMILSLVGNGTVIWIFSTSKSLRSASNMFVINLALFDLTMMLEMPLLIMNSFYQYP
VGFQLGCDIYGIIGSLSGIGGAITNAVIAFDRYKTISCPLDGRINKVQAYLLILFTWIWAMPF
TVLPAVRVWGRYVPEGFLTTCSFDYFTDDQDTKVFVAAIFIWSYGIPMSLICFFYSQLFGAV
RLHEKMLQEQAKKMNVKSLASNKEDANKSVEIRIAKVAFTIFFMFVCSWTPYAFVALTGA
YGDRNLLTPHATMIPAVCAKVVSCIDPWVYAINHPRYRAELEKRIPWLGVREQDPDTASER
ASVATAQSHTPNAEA-

>Pglaopsin-UV
MIPPAVMDNHTEINYNYGAYFAPYRSEGPVEMLGAGLTGEDLAAIPEHWLKYAAPPVSAH
TALALVYVFFTGAALIGNGLVIFIFSTSKSLRTPSNLLVLQLAVLDFIMMLKAPIFIYNSANR
GFATGVIGCKIFAFMGSISGIGAGMTNACIAYDRHSTITRPLDGRLSRGKVILMIVCVWMY
TLPWAILPLLQIWGRYVPEGFLTSCSFDYLTDTFDNKLFVGCIFVCSYVFPMTAILYLYSGIV
KQVFAHEAALREQAKKMNVDSLRSNQNASAESAEVRIAKAALTVCFLYVCSWTPYGVM
SLIGAFGDSSLLTPAVTMIPALACKGVACIDPWVYAISHPKYRQELQKRMPWLQIDEPDDN
VSNTTSNTANSAAPA-
>Aipsopsin-UV
MYNQTENAYYDAHFAPFKSGGPSGVEMLGDGLTGDDLAMVPEHWLAFPAPPASAHTAL
ALLYIFFTAAALVGNGLVIFIFSTTKSLRTSSNLLILQLAILDFIMMAKAPIFIYNSAMRGFAS
GTIGCQLFALMGAYSGIGAGMTNACIAYDRHSTITRPLDGRLSRGKVLLMIAFTWIYSTPW
ALLPLFKIWGRYVPEGYLTSCTFDYLTNTFDTKLFVACIFTCSYVFPMSMIIYFYSGIVKQV
FAHEAALREQAKKMNVESLRSNQNANAESAEIRIAKAALTVCFLFVASWTPYGIMSLIGAF
GDQQLLTPGVTMIPAVACKAVACIDPWVYAISHPKYRQELQRRMPWLQIQEPDDTVSTATS
NTVSSAPAATPA-
>Aipsopsin-BL
MATNYTDNIGPMAYPLKMVSQEVVEHMLGWNIPEEHQDLVHEHWRNFPAVSKYAHYML
ALIYTMLMVTSFLGNGIVIWIFGTSKSLRSPSNMFVINLAVFDLMMMLEMPLLIMNSFYQR
MVGYQLGCDIYAVLGSLSGIGGAITNAVIAFDRYKTISCPLDGRINRVQAFILIAFTWFWAL
PFTVLPALKIWGRFVPEGFLTTCSFDYFTDDQDTKVFVAAIFVWSYAIPMTLICYFYFQLFG
AVRLHERMLSDQAKKMNVKSLTANKDDASRSVEIRIAKVAFTIFFLFVCAWTPYAIVTMT
GAFGDRGLLTPVATMIPAVCCKVVSCIDPWVYAINHPRYRAELQKRLPWMGVREADPDSV
STTTSVGTAQSTAPSAAEA-
>Aipsopsin-LW1
MSLTLDPGPGIAALQAWGGQVAAYGASNQTVVDKVPPDMLHMVDPYWYQFPPMNPLW
HGLLGFTIGCLGFISITGNGMVIYIFMSTKSLKTPSNLLVVNLAFSDFLMMCAMSPAMVVN
CYNETWVWGPLACELYACAGSLFGCASIWTMTMIAFDRYNVIVKGIAAKPMTNNGALLR
ILGIWAFSLAWTLAPFFGWNRYVPEGNMTACGTDYLSKDWFSRSYILIYSVFVYFLPLLLII
YSYFFIVQAVAAHEKGMREQAKKMNVASLRSSEAANTSAECKLAKVALMTISLWFMAWT
PYLVINYTGVFESAPISPLATIWGSLFAKANAVYNPIVYGISHPKYRAALYQRFPSLSCQASP
DESGSVASGATAVSEEKPAA-
>Aipsopsin-LW2
MADTGPGMAALQAWGGQAMHGTVNQTVVDKVPADMLHMVDPHWYQFPPMNPLWHA
LLGFTIGVLGFISIAGNGMVIYIFSSTKSLKTPSNLLVVNLAFSDFLMMCAMAPAMVINCY
NETWVFGPFACELYGCAGSLFGCASIWTMTMIAFDRYNVIVKGIAAKPMTKTGALQRILG
IWLFSLAWTLAPFFGWNRYVPEGNMTACGTDYLSKDWMSRSYILVYSVFVYFLPLLLIIYS
YYFIVQAVTAHEKAMRDQAKKMNVASLRSSEAANTSAECKLAKVALMTISLWFMAWTP
YLVINYTGVFESAPISPLATIWGSLFAKANAVYNPIVYGISHPRYRAVLYQKFPALACTVSPD
EGGSVATGATVVADEKSEKQQAA-
>Atraopsin-UV
MSKTIQLIFILLSVMQCYNLNDSNSESDVSVNENSVAKSFHTIQTVILKRLKCHQDILWGRS
GNLKLWKKRRRTIQPVKVLLSERVKREKPNYKIRRGEVSEPRTIHLVHMNSGLAVSDVAPL
SLVARFKKQWPERLWREKGLYTNDYLVLINSHWLHFPPPDPSVNYVLGSLYIVMMAVGCS

GNAYVLFMYLRCRSLRTPGNILVANLALSDFMMLAKTPIFIFNSFHFGPALGKTGCVIYGF
VGGLTGTTSIATLSAIALDRYWAVVRPLEAVGAITTIRARILAVVAWLYAAIFASVPAFDIGY
GRYVPEGYLTSCSFDYLTEESRPRIFIFVFFCAAWVAPFCTIFFCYLSIFRTVVCNRNITTKNQ
EQRLSSRHVKERAKRKAEIKLAALVMAVIALFFISWTPYAVVALLGIFGRKDLITPLSSMIPA
LFCKTAACINPFIYIITHPNFRKEFKKLLYRDKSRRMGGTMKTIGYYTESTKMHRPSKDLS
DTDVEIIEMTDIPYQTDQLLERRGPKVKTISSRVIDREGSQKSISMKSFEEDVVSPPSWYSK
PKFAKKKSFQTRSLKSIVSLNTDPTI-
>Atraopsin-LW1
MDEEDLINIDTSEYEFPNILWHVKPAQEIAIKTTAMLAIGFTGIFMNSIIIIVIIRNKWLWSAS
NYLVGNLALVDLITLIFCPWFMLVRDFYQQYVLKTFGCQFEGFLQATLLLAGVGAVMLVS
YDRLAAAALSADARVTRNVAPKAIFCTWFIAIVLSLPWILRREYTERQWLDYLETFCVED
VEVLGIYWHFTLTLLVWIPLGLMVVTYGTIMWRLECSARELSTRGSGHAVTKARSRALRI
TACVLMTAVVCRLPYTVLIYWRNNLSNDINSVEGSYDSMWFAANYLMYLNCAVNPLIYG
FTNTRLRKAMDRTPGVACFKFGTWCCVCTMLHRKQVVVTDKHTEKIFVIESTPRPNKKLT
HVIKNILHINKETVDFSIKNDEITMKPTRVTPV-
>Atraopsin-LW2
MDEEDLINIDTSEYEFPNILWHVKPAQEIAIKTTAMLAIGFTGIFMNSIIIIVIIRNKWLWSAS
NYLVGNLALVDLITLIFCPWFMLVRDFYQQYVLKTFGCQFEGFLQATLLLAGVGAVMLVS
YDRLAAAALSADARVTRNVAPKAIFCTWFIAIVLSLPWILRREYTWLDYLETFCVEDVEV
LGIYWHFTLTLLVWIPLGLMVVTYGTIMWRLECSARELSTRGSGHAVTKARSRALRITAC
VLMTAVVCRLPYTVLIYWRNNLSNDINSVEGSYDSMWFAANYLMYLNCAVNPLIYGFTN
TRLRKAMDRTPGVACFKFGTWCCVCTMLHRKQVVVTDKHTEKIFVIESTPRPNKKLTHVI
KNILHINKETVDFSIKNDEITMKPTRVTPV-
>Csupopsin-UV
MENRTDGYYYGAHFAPLRSGAASSVEMLGEGLTGDDLAMIPEHWLVYPTPPASAHTALA
LLYVFLTAAALVGNGLVIFIFSTTKSLRTSSNLLILQLAILDFVMMAKAPIFIYNSAMKGFAT
GTLGCKIFALMGAYSGIGAGMTNACIAYDRHSTITRPLDGRLSRGKALLMMSFVWIYATP
WALLPLFETWGRYVPEGYLTSCTFDYLTNTFDTKLFVGCIFFCSYVIPMSFIIYFYSGIVKQV
FAHEAALREQAKKMNVESLRSNQTAGQQSAEIRIAKAALTVCFLFVASWTPYGIMSLIGAF
GNQELLTPGVTMIPAITCKAVACIDPWVYAISHPKYRQELQRRMPWLQIDEPDDTASTTTT
NTVNAPPAASA-
>Csupopsin-BL
MAENYTNFGIGPVAYPLKMVSQEVVEHMLGWNIPEEHQDLVHEHWRNFPAVSKYWHYC
LALIYTMLMVTSLTGNGIVIWIFGTSKSLRSASNMFVINLAVFDVMMMLEMPLLIMNSFH
QRMLGYQLGCDIYAILGSLSGIGGAITNAVIAFDRYKTISCPLDGRINKIQAALLIAFTWFW
ALPFTILPAAKIWGKFVPEGFLTTCSFDYFTDDQDTKVFVACIFVWSYCIPMALICYFYSQL
FGAVRLHERMLQEQAKKMNVKSLASNKEDASRSVEIRIAKVAFTIFFLFVCGWTPYAFVA
MTGAFGDRSMLTPVATMIPAVCCKIVSCIDPWVYAINHPRYRAELQKRIPWLGVREADPDT
ASTTTSVATNQTSSQADA-
>Csupopsin-LW
MAFASLDPGPGVAALQAWGGQVAAYGASNQTVVDKVPPELLHMVDPYWYQFPPMNPL
WHGLLGFTIGVLGFISISGNGMVIYIFTSTKSLKTPSNLLVVNLAFSDFFMMCCMSPAMVIN
CYNETWVFGALACELYACAGSLFGCASIWTMTMIAFDRYNVIVKGIAAKPMTNNGALLRI
LGIWLFSLAWTLAPFFGWNRYVPEGNMTACGTDYLSKDWFSRSYILVYSVFVYFMPLLLII

YSYFFIVQAVAAHEKGMREQAKKMNVASLRSSEAANTSAECKLAKVALMTISLWFMAWT
PYLVINYTGVFESAPISPLATIWGSLFAKANAVYNPIVYGISHPKYRAELYKKFPSLSCQASP
DESGSVASGTTAVSEEKPAA-
>Heraopsin-UV1
MENETQNYNVYGAFFAPLRSSDGVKMLVDGLDSVDLAVIPEHWLSYTSPPASAHTALALL
YSFFTAAALIGNGLVIFIFATSRSLRTSSNLLILNLAIFDFIMMAKAPIFIYNSAMRGFATGAL
GCQIFSVMGSYSGIGASMTNACIAYDRHSTITRPLDGRLSRGKALLMIACVWIYATPWALM
PLFNVWGRFVPEGYLTSCTFDYLSNTFDTKLFVGCIFTCSYLFPMCFIIYFYSGIVKQVFAH
EAALREQAKKMNVESLRSNQNASAESAEIRIAKAALTVCFLFVASWTPYGVMSLIGAFGD
QQLLTPGVTMIPAVACKTVACIDPWVYAISHPKYRQELQRRMPWLQIREPDDNASTGTNN
TTNSTAPAASA-
>Heraopsin-UV2
MXNETQNSNVYGAYFAPLRSGDGVKMLVDGLSSAELEFIPEHWLTYPAPPASAHTALALM
YCFFTAAALIGNGLVIYIFATTKSLRTSSNLLILNLAIFDFIMMAKAPIFIYNSAMRGFAIGSL
GCQIFALVGSYSGIGAAMTNLCIAYDRHSTITRPLDGRLSRGKALLLIAFVWIYTTPWSLLP
LFRIWGRFVPEGYLTACSFDYLTNTFDTKLFTGCIFIFTYAIPLILIMYYYSGIVKQVFAHEA
ALREQAKKMNVESLRSNQNASAESAEIRIAKAALTVCFLYVASWTPYGIMSLIGAFGDQQ
LLSPGVTMIPAIACKGVACIDPWVYAISHPKYRQELQRRMPWLQIREPDDNVSTGTNNTTN
SAAPAATA-
>Heraopsin-BL
MATNYTDDIGPMAYPLKMVSQEVVEHMLGWNIPEEHQGLVHEHWRNFPSVSKYAHFVL
ALIYTCLILASLSGNGIVIWIFSTSKSLRSASNMFVINLAVFDMMMMLEMPLLVLNSFYQR
LVGYQLGCDIYGVLGSLSGIGGAITNAVIAYDRYKTISSPLDGRLNRVQASLLILFTWLWAL
PFTILPAFRIWGRFVPEGFLTTCSFDYFTEDQDTKVFVMCIFTWSYCIPMLLXCYFYSKLFG
AVRLHERMLKEQAKKMNVKSLAANKEDAGKSVEIRIAKVAFTIFFLFVCSWTPYAFVTMT
GAFGDRSILTPVATMIPAVCAKIVSCIDPWVYAINHPRYRAELQKRLPWMGVREADPDSVS
TSSGATAQTQNPTAEA-
>Heraopsin-LW
MAITSLDPGPGAAALQAWGGQMAAFGSBETVVDKVLPDMLHLIDPHWHQFPPMNPLWH
GLLGFVIGVLGFISVTGNGMVVYIFTTTKTLKTPSNILVVNLAFSDFLMMFMMAPPMVINC
YNETWVFGPLACQLYACAGSLYGCVSIWTMTMIAFDRYNVIVKGIAAKPMTINGALLRVF
GIWAFSLAWTIAPLFGWGRYVPEGNMTACGTDYFDQSFSNRSYILLYSIACYYAPLFLIIYS
YFFIVQAVAAHEKAMREQAKKMNVASLRSSDAANTSAECKLAKVALMTISLWFMAWTPY
LVINYAGIFKTMTISPIVTIWGSVFAKANAVYNPIVYGISHPKYRAALYARFPGLACQSAPE
DNGSVASAATATEEKPSA-
>Lrubopsin-UV
MIPSLSMDTKNESYHLYGAYFAPLRSSDEPEMLVDGLTGEDLEAVPEHWLSYTAPPASAHT
ALALLYIFFTAAALIGNGLVIFIFATTKSLRTSSNLLILQLAILDFIMMAKAPLFIYNSAMRGF
ATGVLGCQIFALMGSYSGIGAGMTNACIAYDRHSTITRPLDGRLSRGKVILMIACIWIYATP
WSLLPLFKVWGRYVPEGYLTSCTFDYLTDTFDTKLFVACIFVCSYVFPMSMIIYFYSGIVK
QVFAHEAALREQAKKMNVDSLRSNQNAAAESAEIRIAKAALTVCFLFVASWTPYGVMAL
IGAFGDQRLLTPGVTMIPAVACKAVACIDPWVYAISHPKYRQELQRRMPWLQINEPSDTAS
TGTTNTTNSTAPAASA-
>Lrubopsin-BL1

MEGNFTDNIGPLAYPFQMVSKETEEHVLGWNYPEEYQFMVQDHWRSYPAISKYWHYGL
AFIYTMLFLASVTGNGIVIWIFSTSKALRSPSNMFVINLAVFDVMMMLEMPIFILNSFHHRI
VGYQTVCDIYATLGSISGFGGAITNAVIAYDRYKTISCPLDGRVTKTQALLLILFTWVWALP
FTILPAFKLWSKFVPEGFLTTCSFDYLTEDSDTKVFVMSCCVWSYFIPVIILCYYYFQLFGA
VRTHEKMLREQAKKMNVKSLASNKEDGGKSVEIRIAKVAFTIFFLFICAWTPYAFVTLVGA
FGDRSILSPVATMVPAVCAKTVSCIDPWVYAINHPRYRAELTKRLPWLGVRESDPDTASSSS
TATSQTQHHTAEA-
>Lrubopsin-BL2
MAYNFTDDFGPVAHPLKMVSSEAEEHMLGWNIPEEYQYFVHEHWRNFPAVSKYWHYGL
AFIYTLLMCASCLGNGIVIWIFSTSKSLRSPSNMFVINLALFDTLMMFEMPLLIVNSFYQK
MLGYQLSCDIYASFGAMSGIGGAMTNAIIAFDRYKTISCPLDGRITKVQALILIAFSWVWSI
PFTFLPAFKVWGRFIPEGFLTTCSFDYLTDDPDTKLFVMCIFCWSYMIPMIFLCFFYSKLFSA
VRMHEKMLREQAKKMNVKSLASNKDDAGKSVEIRIAKVAFTIFFLFVCSWTPYAFVTMT
GAFGDRNILTPVATMVPAVCAKIVSCIDPWVYAINHPRYRAELEKRVSWLGVKEXNPDTVS
TSSTATSQAPADA-
>Lrubopsin-LW
MSITSLDPAPGVAAMQAWGPQAMAYGGNETVIDKVLPEMLHKIDAHWYQFPPMNPLWH
GLLAFVISIICIIATTGNGMVIYIFSTTKSLKTPSNLLVVNLALSDFLIITTMSPPVVVNTYYET
WIFGPLACDIYACCGSLFGCVSIWTMTMIAFDRYNVIVKGIAAKPLTINGALLRILGIWLFS
LAWTITPLFGWGKYAPEGNMCVCGTDYLDKSWVHRSYIILYSVACYFAPLLLIIYSYWFIIQ
AVSAHEKAMREQAKKMNVASLRSSDAANTSAECKLAKVALMTISLWFMAWTPYLIINWA
GIFKTSLISPLVTIWGSIFAKANSIYNPIVYGISHPKYRAALYARFPSLACQPSPDESGSVAST
GTAVQEEKPSA-
>Msteopsin-UV
MANQSDNLYYGAHFEALKSAGPVEMLGDGLTGEDLAAVPEHWLSYPAPPASAHTALALL
YTFFTFAALIGNGLVIFVFTTAKSLRTSSNFLILQLAILDFIMMAKAPIFIYNSAMRGFATGAV
GCQVFALMGSYSGIGASMTNACIAYDRHSTITRPLDGRLSKGKVLVMIALVWIYATPWSLL
PLFKIWGRYVPEGYLTSCTFDYLTNTFDTKLFVACIFTCSYVFPMTMIIYFYSGIVKQVFAH
EAALREQAKKMNVESLRANQGGASQSAEIRIAKAALTVCFLFVASWTPYGVMALIGAFG
NQELLTPGVTMIPAVACKAVACIDPWVYAISHPKYRQELQRRMPWLQIDEPDDSASTATSN
TTNSGPAATA-
>Msteopsin-BL
MATNFTQELYEIGPMAYPLKMISNEVAENMLGWNIPEEHQDLVHEHWRNFPAVSKYWHY
CLALIHTILVITSLTGNGIVIWIFGTSKSLRSASNMFVINLAVFDLMMMLEMPMLVVNSFYQ
RLVGYQLGCDIYGVFGSLSGIGGAITNAVIAFDRYKTISSPLDGRINTVQASLLIAFTWFWA
LPFTILPATRIWGRFVPEGFLTTCSFDYFTDDQDTKVFVACIFVWSYCIPMSLICYFYSQLFG
AVRLHERMLQEQAKKMNVKSLASNKEDASRSVEIRIAKVAFTIFFLFVCAWTPYAFVAMT
GAFGDRGLLTPVATMVPAVCCKVVSCIDPWVYAINHPRYRAELQKRLPWMGVREQDPDS
VSNTTSVATTQSQAPTEA-
>Msteopsin-LW
MTISMDPGPGLAAMQAWGGQVAAYGASNQTVVDKVTPDMMHLIDPHWYQFPPMNPLW
HALLGFTIGVLGFISICGNGMVVYIFMSTKSLKTPSNLLVVNLAFSDFLMMCAMSPAMVV
NCYYETWVWGPFACELYACAGSLFGCGSIWTMTMIAFDRYNVIVKGIAAKPMTNNGALL
RILGIWLFSLAWTVAPMFGWNRYVPEGNMTACGTDYLSKEWLSRSYILVYSVFVYFLPLF

LIIYSYFYIVQAVAAHERAMREQAKKMNVASLRSSEAANTSAECKLAKVALMTISLWFMA
WTPYLVINYTGVFESAPISPLVTIWGSLFAKANAVYNPIVYGISHPKYQAALYARFPALQCA
PSRDDSASTASATTAVSDEKPSA-
>Mbraopsin-UV
MYNQTENSYYDAHFAPFKSGGPSGVEMLGEGLTGDDLAAVPEHWLAFPAPPASAHTALA
LLYIFFTAAALVGNGLVIFIFSTTKSLRTSSNLLILQLAILDFIMMAKAPIFIYNSAMRGFATG
TFGCQLFALMGAYSGIGAGMTNACIAYDRHSTITRPLDGRLSKGKVLLMIAFVWIYSTPW
ALLPLFKIWGRYVPEGYLTSCSFDYLTNTFDTKLFVACIFACSYVFPMSMIIYFYSGIVKQV
FAHEAALREQAKKMNVESLRANQNASAESAEIRIAKAALTVCFLFVASWTPYGVMALIGA
FGDQQLLTPGVTMIPAVACKAVACIDPWVYAISHPKYRQELQRRMPWLQIQEPDDTTSTAT
GNTVSSAPAAAPA-
>Mbraopsin-BL
MATNYTNDIGPMAYPLKMVSKEVVEHMLGWNIPEEHQDLVHEHWRNFPAVNKYWHYC
LALIYTMLMVTSFLGNGIVIWIFGTSKSLRSPSNMFVINLAVFDLMMMIEMPLLIMNSFYQ
RMIGYQLGCDIYAVLGSLSGIGGAITNAVIAFDRYKTISCPLDGRINKVQAFILIAFTWFWAL
PFTILPALKIWGRFVPEGFLTTCSFDYFTDDQDTKVFVACIFVWSYAIPMALICYFYSQLFSA
VRLHERMLSDQAKKMNVKSLAANKEDASRSVEIRIAKVAFTIFFLFVCAWTPYAFVTMTG
AFGDRNLLTPVATMIPAVCCKVVSCIDPWVYAINHPRYRAELQKRLPWMGVREQDPDSVS
TTTSVATAQSTAQPAAEA-
>Mbraopsin-LW
MSLTLDPGPGIAALQAWGGQVAAYGASNQTVVDKVPPDMLHMVDPHWYQFPPMNPLW
HGLLGFTIGCLGFISITGNGMVIYIFMSTKSLKTPSNLLVVNLAFSDFLMMCAMSPAMVVN
CYNETWVWGPLACELYACAGSLFGCASIWTMTMIAFDRYNVIVKGIAAKPMTNNGALLR
ILGIWLFSLAWTLAPFFGWNRYVPEGNMTACGTDYLSKDWFSRSYILIYSVFVYFLPLLLII
YSYFFIVQAVAAHEKGMREQAKKMNVASLRSSEAANTSAECKLAKVALMTISLWFMAWT
PYLVINYTGVFESAPISPLATIWGSLFAKANAVYNPIVYGISHPKYRAALYQKFPSLSCQASP
DESGSVASGATAVSEEKPAA-
>Msexopsin1
MDPGPGLAALQAWAAKSPAYGAANQTVVDKVPPDMMHMIDPHWYQFPPMNPLWHALL
GFTIGVLGFVSISGNGMVIYIFMSTKSLKTPSNLLVVNLAFSDFLMMCAMSPAMVVNCYY
ETWVWGPFACELYACAGSLFGCASIWTMTMIAFDRYNVIVKGIAAKPMTSNGALLRILGI
WVFSLAWTLLPFFGWNRYVPEGNMTACGTDYLSKSWVSRSYILIYSVFVYFLPLLLIIYSY
FFIVQAVAAHEKAMREQAKKMNVASLRSSEAANTSAECKLAKVALMTISLWFMAWTPYL
VINYTGVFESAPISPLATIWGSLFAKANAVYNPIVYGISHPKYQAALYAKFPSLQCQSAPED
AGSVASGTTAVSEEKPAA-
>Msexopsin2
MNNQSENYYHGAQFEALKSAGAIEMLGDGLTGDDLAAIPEHWLSYPAPPASAHTALALL
YIFFTFAALVGNGMVIFIFSTTKSLRTSSNFLVLNLAILDFIMMAKAPIFIYNSAMRGFAVGT
VGCQIFALMGAYSGIGAGMTNACIAYDRHSTITRPLDGRLSEGKVLLMVAFVWIYSTPWA
LLPLLKIWGRYVPEGYLTSCSFDYLTNTFDTKLFVACIFTCSYVFPMSLIIYFYSGIVKQVFA
HEAALREQAKKMNVESLRANQGGSSESAEIRIAKAALTVCFLFVASWTPYGVMALIGAFG
NQQLLTPGVTMIPAVACKAVACISPWVYAIRHPMYRQELQRRMPWLQIDEPDDTVSTATSN
TTNSAPPAATA-
>Msexopsin3

MATNFTQELYEIGPMAYPLKMISKDVAEHMLGWNIPEEHQDLVHDHWRNFPAVSKYWHY
VLALIYTMLMVTSLTGNGIVIWIFSTSKSLRSASNMFVINLAVFDLMMMLEMPLLIMNSFY
QRLVGYQLGCDVYAVLGSLSGIGGAITNAVIAFDRYKTISSPLDGRINTVQAGLLIAFTWF
WALPFTILPAFRIWGRFVPEGFLTTCSFDYFTEDQDTEVFVACIFVWSYCIPMALICYFYSQL
FGAVRLHERMLQEQAKKMNVKSLASNKEDNSRSVEIRIAKVAFTIFFLFICAWTPYAFVTM
TGAFGDRTLLTPIATMIPAVCCKVVSCIDPWVYAINHPRYRAELQKRLPWMGVREQDPDA
VSTTTSVATAGFQPPAAEA-
>Picaopsin-UV
MIPPIDMDKNETYHLYGAYFAPLRSSDAIEMLGEGLTGEDLAQVPEHWLSYPAPPASAHTA
LALLYTFFTAAALIGNGLVIFIFATTKSLRTSSNLLILQLAILDFIMMAKAPIFIYNSIMRGFAT
GAMGCKIFGLMGSYSGIGAGMTNACIAYDRHSTITRPLDGRLSQGKAILMVAFIWIYATPW
SLMPLFEVWGRYVPEGYLTSCTFDYLSNTFDTKLFVGCIFLCSYVFPMTMIIYFYSGIVRQ
VFAHEAALREQAKKMNVDSLRSNQNAGAESAEIRIAKAALTVCFLFVASWTPYGVMALI
GAFGDQNLLTPGVTMIPAVTCKAVACIDPWVYAISHPKYRQELQRRMPWLQINEPSDSAST
GTTATANSAAPASA-
>Picaopsin-BL1
MAFNLTDDFGPVAALKMVSDEAEEHMLGWNVPEEYQYFVHDHWRAYPAVSKWWHYGL
AFIYTILMFCSCLGNGIVIWIFSTSKSLRSPSNFFVINLAIFDTLMMFEMPMLIVNSFYQTML
GYQLSCDIYAAFGAMSGIGGAMQNAIIAFDRYKTISSPLDGRITNVQAMILIVFTWIWTLPF
TFLPFFRVWGRFIPEGFLTTCSFDYLTEDTDTKVFVMCIFLWSYVTPMTFLCFFYSKLFNAV
RAHERMLREQAKKMNVKSLASNKDEAGTSVEIRIAKVAFTIFFLFVCSWTPYAFVTMTGA
FGDKGILTPVATMVPAVAAKIVSCIDPWVYAINHPRYRAELEKRVSWLGVKEPSPDTVSQSS
TATSQVPQEA-
>Picaopsin-BL2
MDNSTGNIGPMAYAFQMVEEETKEHILGYNYPEEYQFMVQDHWRSYPAINKYWHYSLA
LIYTMLFIASITGNGIVIWIFSTSKALRSPSNMFVINLAVFDVMMMLEMPIFVLNSYHHHIIG
YQAVCNVYATLGSISGFGGAITNAVIAYDRYKTISCPLDGRVTKSQALALIVMTWVWSLPF
TILPAIQVWGKFVPEGFLTTCSFDYLTEDYATRMFVLVCCIWNYFLPVFILCYYYALLFGAV
RTHEKMLREQAKKMNVKSLSNKDDGGASVEIRIAKVAFTIFFMFVCAWTPYAFVTLVGAF
GDRSILTPVATMVPAVCAKTVSCIDPWVYAINHPKYRAELTKRLPWLGVREKDPDTASSSS
TATSQTHHPTAEA-
>Picaopsin-LW
MTIMNLDPAPGVAALQSWGPRASALFNNETGVDKVLSEMLHRIDPHWDQFPPMNPLWH
AILAFITSVILIIASTGNGMVIYIFTTTKTLKTPSNLFIVNLALSDFLIITTMSPPVVVNTYYET
WIFGPLACDIYACCGSLFGCVSIWSMTMIAFDRYNVIVKGIAAKPLTNGGALLRILGIWLFS
LAWTITPLFGWGKYAPEGNMCVCGTDYLDKSWVHRSYIILYSFAYYFMPLFLIIYSYWFIIQ
AVSAHEKAMREQAKKMNVASLRSSDQANTSAECKLAKVALMTISLWFMAWTPYLIINFA
GIFKTERISPLATIWGSVFAKANAIYNPIVYDISHPKYRAALYARFPALACQPSADESGSVAS
SGTAVQEEKPSA-
>Sexiopsin-UV
MYNQTENSVYDAHFAPFKSGSPSEVEMLGAGLTGDDLAAVPEHWLSFPAPPASAHTALAL
LYTFFTAAALLGNGLVIFIFSTTKSLRTSSNLLILQLAILDFIMMAKAPIFIYNSAMRGFATGT
VGCQIFALMGAYSGIGAGMTNACIAYDRHSTITRPLDGRLSRGKVLLMMAFVWIYSTPWA
LLPLFKIWGRYVPEGYLTSCTFDYLTNTFDTKLFVACIFTCSYVFPMSMIIYFYSGIVKQVFA

HEAALREQAKKMNVESLRANQSSGAESAEIRIAKAALTVCFLFVASWTPYGVMALIGAFG
DQQLLTPGVTMIPAVACKAVACIDPWVYAISHPKYRQELQRRMPWLQIQEPDDTVSTATSN
TNAPPAAPA-
>Sexiopsin-LW
MSLTLDPGPGIAALQAWGGQVAAYGASNQTVVDKVLPDMLHMVDPHWYQFPPMNPLW
HGLLGFTIGVLGFISITGNGMVIYIFMSTKSLKTPSNLLVVNLAFSDFLMMCCMSPAMVVN
CYNETWVWGPLACELYACAGSLFGCASIWTMTMIAFDRYNVIVKGIAAKPMTNNGALLR
ILGIWAFSLAWTLAPFFGWNRYVPEGNMTACGTDYLSKDWFSRSYILIYSVFVYFMPLLLII
YSYFFIVQAVAAHEKGMREQAKKMNVASLRSSEAANTSAECKLAKVALMTISLWFMAWT
PYLVINYAGVFESATISPLATIWGSLFAKANAVYNPIVYGISHPKYRAALYQKFPSLSCQASP
DESGSVASGATAVSEEKPAA-
>Sexiopsin-BL
MATNFTDDIGPMAYPLKMVSKEVVEHMLGWNIPEEHQDLVHDHWRNFPAVNKYWHYV
LALIYTMLMVTSLLGNGIVIWIFGTSKSLRSPSNMFVINLAVFDLMMMLEMPLLIINSFYQR
LIGYQLGCDIYAVLGSLSGIGGAITNAVIAFDRYKTISCPLDGRINKVQAAILIAFTWFWAMP
FTILPALKIWGRFVPEGFLTTCSFDYFTDDQDTKVFVGCIFVWSYAIPMALICYFYSQLFGA
VRLHERMLSDQAKKMNVKSLAANKEDASRSVEIRIAKVAFTIFFLFVCAWTPYAFVTMTG
AFGDRSLLTPIATMVPAVCCKVVSCIDPWVYAINHPRYRAELQKRLPWMGVREQDPDSVS
TSTSVGTAQSTAQPTAEA-
>Slitopsin-UV
MYNQTENSYYDAHFAPFKSGGPSDVEMLGAGLTGEDLAAVPEHWLSFPAPPASAHTALA
LLYTFFTAAALLGNGLVIFIFSTTKSLRTSSNLLILQLAILDFIMMAKAPIFIYNSAMRGFASG
TVGCQIFALMGAYSGIGAGMTNACIAYDRHSTITRPLDGRLSRGKALLMMAFVWIYSTPW
ALLPLFKIWGRYVPEGYLTSCTFDYLTNTFDTKLFVACIFTCSYVFPMSMIIYFYSGIVKQV
FAHEAALREQAKKMNVESLRANQSSGAESAEIRIAKAALTVCFLFVASWTPYGVMALIGA
FGDQQLLTPGVTMIPAVACKAVACIDPWVYAISHPKYRQELQRRMPWLQIQEPDDTVSTAT
SNTVSNAPPAAPA-
>Slitopsin-BL
MATNFTDDIGPMAYPLKMVSKEVVEHMLGWNIPEEHQDLVHDHWRNFPAVSKYWHYVL
ALIYTMLMVTSLLGNGIVIWIFGTSKSLRSPSNMFVINLAVFDLMMMLEMPLLIMNSFYQR
LVGYQLGCDIYAVLGSLSGIGGAITNAVIAFDRYKTISCPLDGRINKVQAAILIAFTWFWAM
PFTVLPALKIWGRFVPEGFLTTCSFDYFTDDQDTKVFVGCIFVWSYAIPMALICYFYSQLFG
AVRLHERMLSEQAKKMNVKSLAANKEDASRSVEIRIAKVAFTIFFLFVCAWTPYAFVTMT
GAFGDRSLLTPIATMVPAVCCKVVSCIDPWVYAINHPRYRAELQKRLPWMGVREQDPDSV
STTTSVATAQSTAQPTAEA-
>Slitopsin-LW1
MSLTLDPGPGIAALQAWGGQVAAYGASNQTVVDKVPPDMLHMVDPHWYQFPPMNPLW
HGLLGFTIGVLGFISITGNGMVIYIFMSTKSLKTPSNLLVVNLAFSDFLMMCCMSPAMVVN
CYNETWVWGPLACELYACAGSLFGCASIWTMTMIAFDRYNVIVKGIAAKPMTNNGALLR
ILGIWAFSLAWTLAPFFGWNRYVPEGNMTACGTDYLSKDWFSRSYILIYSVFVYFLPLLLII
YSYFFIVQAVAAHEKGMREQAKKMNVASLRSSEAANTSAECKLAKVALMTISLWFMAWT
PYLVINYTGVFESAPISPLATIWGSLFAKANAVYNPIVYGISHPKYRAALYQKFPSLSCQASP
DESGSVASGATAVSEEKPAA-

2.CSPs

>BmorCSP1
MKVLIVLSCVLVAVLADDKYTDKYDKINLQEILENKRLLESYMDCVLGKGKCTPEGKELK
DHLQEALETGCEKCTEAQEKGAETSIDYLIKNELEIWKELTAHFDPDGKWRKKYEDRAK
AKGIVIPE-
>BmorCSP2
MKLLLVFLGLFLAVLAQDKYEPIDDSFDASEVLSNERLLKSYTKCLLNQGPCTAELKKIKD
KIPEALETHCAKCTDKQKQMAKQLAQGIKKTHPELWDEFITFYDPQGKYQTSFKDFLES-
>BmorCSP3
MKSLIVLSCLLAACLAADLSKYENFDVEPIVTSDRLLKAYINCFLDKGRCTPEASDFKKAL
PDTIATNCGKCTEKQKANVRKVIKVIQQKHSTEWEKLVKKHDPSGKHRADFDKFLLGS-
>BmorCSP4
MFMLFIISFIIVPVLKCCGTETSTYTTQYDEVDIKEIMGNERLLVAYIGCLLDKNPCTPEGKE
LKRNIPDALQSDCSKCSDKQRENADAWIEFMIDNRPEDWTKLEERYNPDGSYRTKYLEGK
HNATSNVDESK-
>BmorCSP5
MKFLVTAVLLSLAIAIQAGSYSDRYDNINVAEILGNKRLLTAYIKCVLEEGKCTAEGKELK-
>BmorCSP6
MKCLTIAALLFVAGLSIAEKYTDKYDNIDVDEILENRKLLVPYIKCVLDEGRCTPDGKELK
AHIKDGMQTACAKCTDKQKVSARKIVKHIKQHEADYWEQMKAKYDPKDEFKEIYEGFL
AGQN-
>BmorCSP7
MKSVILICFLGVATVVIARPKTPFDNINIEEIFENRRLLLGYINCILERGNCTRAGKDLKSSL
KNVLEENCDKCSEDQRKSIIKVINYLVSSEPESWNQLKSKYDPEGKYLIKYEAKMESN-
>BmorCSP8
MNSLIAFCLFAVLAVALARPDDKYTDRYDNVNLDEVLSNSRLLQPYIKCILDKDRCAPDA
KELKEHIREALETECAKCTEAQKKGTRRVIGHLINNESKSWNELTAKYDPENKFTAKYEK
ELREIKA-
>BmorCSP9
MKTVIVCLLALTAVALARPEQYTDKYDTVDLDQLISNRRLLIPYVHCILEKGQCTAEGKEL
KSHIKEALETNCAKCTKAQKGGTEKMIGHLINHEAEFWEELKAKYDPTNEFTKKYETELK
RVTA-
>BmorCSP10
MRAVIFLYTCVFVVVGQDINAMMSMPKYDERYDYLDVDDIFRNKRLVRNYVDCLINAQR
CTPEGKALKRILPEALRTKCIRCTERQKRTSVKVIRRLKNEYPEEWAKLASRWDPTGDFTR
YFEDYLAKEHFNTIPGSGL-
>BmorCSP11
MKTILILCALVSVVVCRPEEYYSSQYDNFDVEQLVGNLRLLKNYAKCFLDQGPCTAEGTE
FKKRIPEALRTKCAKCNPKQRHLIRTVVKAFQTKLPDLWEELAIKEDPKGQYKHEFTAFIN
AMD-
>BmorCSP12

MKGFYVLCFALFAAVYCKETYSSENDDLDIEALVGNIDSLKAFIGCFLETSPCDAVSGDFK
KDIPEAVAEACGKCTPAQKHLFKRFLEVVKDKLPQEYEAFKTKYDPQGKHFDALLSAVAN
S-
>BmorCSP13
MKFVLALIALAVVVAARPNDDLFYDKKYDNFNVDEIIDNPRLLKAYTFCFNDKGKCTAEG
NDFKKWIPESLQTSCGKCSEKQKYLVAKFVHAIKDKMPDEFDILRKLHDPKGEYTENLDK
FLETYGH-
>BmorCSP14
MKILIIVVMACVAVTWARPESTYTDKWDNINVDEILESNRLLKGYVDCLLGKGRCTPDGK
ALKETLPDALEHECVKCTGKQKSGADKVIRHLVNKRPDLWKELAVKYDPDNIYQARYKD
KIDAVKGSA-
>BmorCSP15
MKLTSFLLVGMAMVSAEFYSSRYDDFDVKPLVENDRILQSYTNCFLDKGPCTPDAKEFKK
VIPEALETTCGKCSPKQKQLIKTVIKAVIERHPEAWEELVNKYDKDRKFRPSFDKFINEDD-
>BmorCSP16
AHITDALQTGCTKCTGAQRKGIRRVIKHLIDSEPGYWDRLVDMYDPKRVYTGKYEKELRT
IKA-
>BmorCSP17
MKSSLFCVLVLTVVVSSSRQQSYPRNDNININAILQNDRILLGYFKCVMDRGPCTKDGKTF
KRALPEALPTACARCSNKQKAAFRTLLLAIRARSEPSFLELLDKYDPSRSNRELLYTFLATG
L-
>BmorCSP18
MNNLLIAILALTLPFSIWCYDEKYDKIDVDKILSDDKLFTDYINCMLDKGPCEVEYSSEFK
ELLPEVIATSCAKCTPIQKTGLRKTVKALSVKRPDDFSQFRAKYDPKGEYEKQFAAFVVAT
D-
>BmorCSP19
MIENFYSKCTISKSVLFLCLIFLPYALNQKYYDSRYDYYDIDHLVQNPRLLKKYLDCFLGK
GPCTPIGRLFKQVMPEVITTACAKCTPTQKRFARKTFNAFRRYFPETLMELRRKFDPESKY
YDAFEKVITNA-
>BmorCSP20
MIEWKRFKILHFLSYLGLLVLVVVCAAQQNRPQVTDTALDEALNDKRFIQRQLKCALGEA
PCDPIGKRLKTLAPLVLRGACPQCSPQETKQIQKTLSYVQRNFPQHWAKLVRQYAG-
>BmorCSP21
MPPNLKNIVFIGVCTCLVLTVLAAPQMSDAQLEKTLADKGTMQRHLRCALGEGPCDMVG
RRLRTLAPFVLRGACPQCSVQESRHIRRTLAYIQRNYPWEWARIVRQYG-
>BmorCSP22
MNNLLIAILALTLPFSIWCYDEKYDKIDVDKILSDDKLFTDYINCMLDKGPCEVEYSSEFK-
>BmorCSP23
GNPKLLQTYFNCLLDKGECAPDAKELKIKLSTAKHLSESIETVCAKCTEAQKKGARRVVIT
VGSGLSLPLALMKSMGDGNHSPSGGPSVVSES-
>BmorCSP24
GNPKLLQTYINCLLGKGKCAPDGKELKIKLLVLLHTRCQVVFKINK-
>DpleCSP-1
MSDMQLERTLADRVMMQRHIKCALSEGPCDPTGMRLRTLAPLVLRGSCPQCSSQETRQIR

RTLAFVQRNYPWEWTKILSHVIVIFAMAALCFAETQRPAVSDTALEDALNDKRFIQRQLKC
ALGEAPCDPIGKRLKTLAPLVLRGACPQCSPQETKQIQRTLSYVQRNFPQQWAKIVRQYSG
-
>DpleCSP-2
MLDKFKGGGPKAIFDIVTAHSAKRTVEYLAMAGLVIESNPPYCTELAPCDFYLFQELKIKIE
VFILGALKMRSTINMKIVILLSLLSVVAANDHFYYNLIPLEVTALAKNPKQIFEFLDCLLDK
GPCNDVFEGYRAVSLEAVQQACKRCTADQKRFGNIFLMLLRKLLPQEYHNFRYKYDPKN
KYFDALEAELSKYKYLPCL-
>DpleCSP-3
MKVVIILATLITLVLTQDTYSSDLENFDIDELLENDRLLESYGKCLEYKGPCTSQGREFRKL
MPEAIRTNCLKCTLKQREMVRKAAKALNVKLPKIWNELVKQEDPKGEYKQKFEDFLQRS
D-
>DpleCSP-4
MKTFVVFAMCLAAALALPADTYNPEYDNFNAKELVENPRLLKNYGKCFLDQGPCTAEGS
DFKKTIPEALRTTCAKCTPKQRELIRTVVRGFQKDLPEMWSELVKKEDPKGEYKESFEKFL
NGSD-
>DpleCSP-5
MKIVILLSLLSVVAANDHFYYNLIPLEVTALAKNPKQIFEFLDCLLDKGPCNDVFEGYRAV
SLEAVQQACKRCTADQKRFVQRRLQMNRYKMKIFITLAALVINAVVAEEELYDLPLLDMR
EIARNPDQLKSFLDCLLDRAPCNPLYQGYRDLAPESIRESCRKCTPALKIFAYVFFTTLKTFL
PEEYQNFRNKYDPDNIYIDRLEEEVRKYAFGTFSG-
>DpleCSP-6
MFDNIKALSLSAAASSLGCIESKRIIKMRTFVVLACLVVSILAAEKYNSKYDNFDVETLVSN
DRLLKSYINCFLEKGKCTAEGTDFKKALPEAVETVCGKCTDKQKVNIRKVIKAIQQKHPT
QWEELVKKNDPTGQHRANFEKFIQES-
>DpleCSP-7
MKIFILLLCVFAAVIAEENSDLSTENIDLTEVVGNDKLIESYANCLINKGPCTPEMEKIKGLL
PEAIATKCSKCTEKQKEVGSQLIKGVREKHPEIWKKLTTYFDPEEKYRESVEEFLKA-
>DpleCSP-8
MATLYIFIFATISTLVLAEFYSSRYDDFDIQPLLENDRILFSYTKCFLDQGPCTPEAKDFKKAI
PEALETTCGKCSPKQKQLIRKVIKAIIEKQPQAWEQLVEKYDTEKKYRESFNKFIEEEN-
>DpleCSP-9
MKSCVLLALLSLVAVAWGRPQHYTDKWDNINVDEILESQRLLRGYVDCLVDKGRCTSDA
KTLKETLPDALENDCKKCTEKQKTSADKVIRHLVNKRPDLWKELAGKYDPKDIYQQKYK
NKIEAVKEKH-
>DpleCSP-10
MYLALETSQLSKTKIVKSQSVFLKVTREFYIVACIGAFDFEALVALVAARPEDNYDRYENF
DVDELVSNLRLLKSYAACFLGEGKCTAEGNDFKKWIPEAVQSNCGKCSDHQKHLVGKVI
KACIDKLPEEWNKLNAIHNPDGKYDEKLKDLPGKIRKLNKMKFLVILALVALAAARPEAN
YEKYENFDVDELVSNLRLLKSYVACFIGEGKCTPEGSDFKEWIPEAVQSNCGKCSDNQKH
LVGKVIKACMEKLPEEWKKLNALHNPDGKYDEGLKNFLDNYGH-
>DpleCSP-11
MAVCYCRPDTYTDRYDSIDLDEILRNRRLLVPYIKCILEEGRCSADGKELKSHIKEALEND
CAKCTKAQQDGTKKVIGHLINKENDYWQQLVEKYDPEHKYVEKYEKELRA-

>DpleCSP-12
MDQGRCTPEGNTLKVHVTDAIQNSCSKCTEIQKTKARKVVNYIRENNKDVWDELIKKYD
PKDEYKEKYEAFLEGKF-
>DpleCSP-13
MKSIFLVFLLAFVLNCAIADEQYYVLQKVNLSESSDIIGVMKNLMNCFLERSPCSEAFESY
RVRIPEAFQQACKKCSPEQKRFAAEFIQSLKAEMPEDYNDFIKKYDPENKYFDALEAELN
KFI-
>DpleCSP-14
MDLFPISVLLVACFTAINTQTYEKRYDSVNIDDVLSNKRLLAAYVKCVLDQGRCTPEGKEL
KSHIADALQSGCDKCTETQKDGVRKVIKHLIKNERDYWKQLVEKFDPEGVYAEKYEDEIR
DV-
>DpleCSP-15
MVIIIILAALVINAVVAEQEKYNSSLFDLEEVVNLAPESIRESFRKWTPALKIFAYVFFSTLKT
FLPEEYQNFRNKYDPDNIYIDRLEEEVRKYAFGTFSG-
>DpleCSP-16
MGMRHFPKNCYKMMIIITLAALLINAVVAEEEKYDLPLLDMKEIAGNPNQLKSFLDCLLD
RAPCKDVYQGYRDLAPESIREACRKCTTELKIFAYIFFYTLKTFLPEEYQNFRNKYDPDNIY
IDRLENEVSKYRLAAIAS-
>DpleCSP-17
MKTIIFLAVIAFAVADPELYDVVTLDMDKMEKDPQEFNSLIDCLLDRGPCTPVFQTYRGIV
QEATENICKKCNPYQKRAYWHFLRILRTTNPVDYENFRQKFDADNVYVDILESVLRGFAN
LK-
>DpleCSP-P10
MGIWLSSNNYVILRDLYKSLIMSTQYSFLLFEIFYVFVVWGDIDEMSEASYLLFTQTSVCY
KASVFIINKKNLIVLLVSMKAKIFEPQSSIHYGILYRNARVVKNLCAVFLMSAITTCSLWTIM
PLFDAAETRFFPFKIWMPVDPRDSPQYELGYIYQVMSIYISALLFVAVDNITLGMIMFGCA
QLEIVIDKIKQLKNASSKHNKERRKLICENNKLLDECIKQHQSVIRFIETLENTYHANIFFQL
TATVGIICIIGLRISIVEPRSVQFFSMLNYMVTMLSQLFLYCWCGSHLTTKSEELREWLYQCP
WYNQDPRFRKSVFIAMERMKRPIIFKAGHYIPLSRPTFVSVLRLSYSYFAVLNQAKNK
>DpleCSP-P12
MMPCVGYLFVAIAKFYKIERYKPVFINLISELREMWPEDNVTDEEQVIISSALNRLRLVTKT
YFYCNLILAFIFTLPANINLIKSLFGIEVPRILPFVYWMPFDPSKKVVFEVVLIVQNIHCFLSA
AYMLAGDLLFFVFLSHITTQFSLLAVRIQKMFYVPIDGQLPESYPLGYYNKENSLNNPNQD
TANNRCKVEEEELKNIVIRHKALIRVVILIDHFQSTGVGEALYNSAWYVSSNSIKKSILIMIH
RSQRRVHITTFGFSTVSMECYATVSVNITAVTLH-
>DpleCSP-P12-2
MAKIYKIKRYKPVFINLISELREMWPEDNVTDEEQAIISSALNRLRLVTKTYFSCNSILGLIF
TLPSIINLIKPLFGIEAPRILPFFYWLPFDPYQEVIFEVVVIVQNSHCFLSAAFMLAGDLLFFS
FLSNITTQFSLLAVRIKKMFYAPIDGQLPESYPLGCSNEGILLNNLSQDTSTNRSKVKEEELK
EIIKRHRSLIRLSNDVENMYSFSLLVNFLNSSIIICFCLFCCAVSTKTVKNY-
>DpleCSP-P20
MYPNPVYDEKRKKLIVMAILSVLPAFTLAGNDIRLRILNNDMANVVRQSIILVSITFVIIKFII
SIARKDEFRSLLEEIDADYERFNGMSEQYQDLVEDTIRKTRKVEKSWCFVLLVTTASYPVL
AGACTIYSQLFSDYPRRYMVHELKAILISEEQKYQSPYFEIVSLYTMYIVIFLFIGFTGFDGM

FSVCLLHVSLKLKIYQENLRNLFNEKDIQKIKYNIGLFVKNHCGVLRLIAKIQTCFEVWLV
GIFINAVVQIGMAFTQITNQTESDINQMYYLYALATVVHIYLPCYFASDVTYNAAEIANVAY
SSSWERVQDSKIRSSICFIIAKCQTPVRLTALDMLTFNMELFVSVGHQM-
>DpleCSP1
MDSPYGCRVHRVLQGEASTVPGTSDPGVVSMKTLTPFSLFFMAKLCLGDNSYTTKYDGIN
LDEILASHRLLTGYINCLLDKGPCSPDGKELKNNLPEAIDNDCHKCTQRQKEGADKVMHF
IIDNRPEDWDKLEEKYNSDGSYKLKYLTTKLSENSEDVSKNYDEDDEEVNESRDHTSDND
HDTEMENH-
>DpleCSP4
MGQPEGCNLSDQKMILKSCVVLCCVLVCVLGDEKYTDKYDNVNLQEILENKRLLEAYVN
CILDKGKCSPEGKELKEHIQDALETGCEKCTDKQMEGTTTMIDHLVKHERAMWKELTDK
FDPKGIWRKKYEDRAREKGIVIPED-
>DpleCSP7
MADSPCLPPWILLIEKGGGHERAADALLSFGLVGSMSGLLLSALCRIRRRLVSLKSIRVIFN
GSPFLFLRQIFNMRTLILALVAFAAANSQYYVLEKLDMDLLVKNLDELKIFTNCLLEKIPCN
KVHESYRVKTLEAVQEACKKCNPHTKHMFWEYLQALKADLPKEYIEFRHKYDPDNKYF
DIHEAEISKYAIE-
>DpleCSP7-2
MYHRYIWFEVCDTTLTSRKNARATNPVQSGPQSSWKKLDREMLLEVVLVQSWLSASEDV
EVEVEALWFRISMQRLNLEVLLNLMFKMKSVMILALFAFVAADKDYYVLDKIDLSKIENN
IEELKIFMDCVLDKGTCSDLYNSYKVHIDESFKTACGKCTPEQKQFVSQFFKLYRKVLPQD
IEELINKYDPERKYIDDLLAEVDKYNV-
>DpleCSP7-3
MLKGATWQSAYLMVVPSPMDSLIGEEEERRKRWENGGKSKRGKGKGQPALTLIGRSEAI
KNYFTPIFCERVYKKERGLKHRHTRLYQAHYIKGIKSKKKMKLLFLLVLVSFAAAEEQFYS
LQKIDLSKAEENIGEFKKFTDCLLEKGPCSDVYESYRVRVNESLQSACGKCTPELKQFAAK
FFEILKNYLPQEYDGFLKKYDPENKYYDVFMAEVLKYKV-
>DpleCSP11b
MKVIIVLSALLVLSSAETYNSENDDLDIEKLVSDPASLGAFLDCFNDKGACDELSGDFKKD
LREAVEQACEKCTAAQKHIFKRFLEVIKVQKADDYKIFQQKYDPENKYLPALEAAIAKY-
>DpleCSP12
MILTVTCVLSYDEKYDRLDVDAILADDQHFTSYLDCFLDKAPCTTEYSKEFRELLPEVIKT
ACEKCSDTQKVKVRKFVKAIFEKKAEMAQEFRTKFDPTGEYEPAFLAFLKQNK-
>DpleCSP13
MRCLLLALCALTVVASGVAQQNQFLHRYDNFNPDSIIQNERILLAYYKCVMDKGPCTKDG
KNFKRVLPETLSTACGRCSPNQKIVVRKMLLGIRAKSEPRFFELLDKYDPTRVNREALYSF
LITGDERNF-
>DpleCSP15
MKIVYFIFFIVFGVFADNKYYDSRYDYYDIDHFVQNPRLLKKYIDCFLDKGPCTPIGRVFK
LVLPEIVITACNKCSPSQKRFAHRTFEAFRNISPQNYAELRRKLDPQNKHFNTFLKSIATNS-
>DmelCSP1
MLLLNKNRVISLVVNFIFLIILISSSVQADERNINKLLNNQVVVSRQIMCILGKSECDQLGLQ
LKAALPEVITRKCRNCSPQQAQKAQKLTTFLQTRYPDVWAMLLRKYDSA-
>DmelCSP2

MKASLALVFCVCVGLAAAAPEKTYTNKYDSVNVDEVLGNNRVLGNYLKCLMDKGPCTA
EGRELKRLLPDALHSDCSKCTEVQRKNSQKVINYLRANKAGEWKLLLNKYDPQGIYRAK
HEGH-
>DmelCSPA7a
MGPITLLLSILLVHSCRAEKPYSVELNTFTMDDTIENQENWVDWGTLSMKKVSRNQFVVS
GDFEFKLNMADEQKIVLMVYVYDSNANQRGSMVMAVKKPFCQFIKEDEDSYPSIQKASN
LPDQDTCPFPKGKYTIDNYELETNFLPDNAPKGDYLLQLSLLDREVPVAGLVATVTLT-
>DmelCSPA29a
MWHCELKVFLILWTVSQVYIPCWGKKFISRFESINGIEGEKETLFTCSVRLVGRERMLNGS
IMHQVDLDDSFDVWMDILHFKNGEWAQGNIKVRTKPCDWFTNYFGKYFLPLVKDSNLPP
IQEMCVFPKGEYYLRITKIEPQNWPPILYRGLNQFNINYVRDGKSTGGIQFVIDLEDSTL-
>DmelCSPB38a
MISISTILALVVSSVWATDYTLEFEDSDLYSECSEKLPGAIGLREAFDMRNIVTELDIDGLHL
SGNCTTIWDVPSTDRISLRMTVMHFDRGTWQPTVFNTYARDFCAVMFDKELSWYKYWL
KYFANREEISEKCIGTKGTVLVYKPFIVKPLIQNVIGPIYRGRVKAIFNFESFDKNNVKGATD
VCFEVRGQVEKIK-
>DmelCSPB38b
MIRGLVILVLALANTWATDYNALIDDEGIYVKCSEAPAGTLGPRDVFNIDNMVMHMEPEG
IYVSGNMTVKLNFLPSDRISARFSVMHYERGSWQPTMFNLHSPNFCEVMFDEDQYWFKY
WFRYIRNKEEIREKCLKVKDTVLVYDEFLMVLHLENVNTSNLQGRYKAVITLEAFDEHNV
RRPSSLCVEIRGDLERVT-
>DmelCSPB38c
MFATLLILILGSTDILATDYILLVEDPDIYTPCTDGPPGSVGLNEAFDVSEMQVEMDEEGIH
VSGNITTRWSLPPTYRISARMSVLHFNRGNWEPTVFNTLTPDFCDAMFNPNLFWYKYWF
KNFENREEIQEKCLATQGTVLVYNPFVVVPRLNNVLGPTLKGRYKVVFLFEAFNEQDERQ
PSSVCFEITGDAEKIKN-
>DmelCSPB42a
MKATFTILVLQVVICLAGATEYQLTLDKDGLLAPCENQPGNPSGFEAMVDTSSLKVHNLG
SKVRIEGEQKVVWKDVQPGDTLKVFGQVYRLDKGTWQKTMFTASSNNFCKNMFDKNQ
YWYNFWTKYISNSDEIKEKCLTTPGAVLKYKDYELDLKTSLNVPNLDGRYKLVVQIEAFD
KRNVRRPVPICIEFRGTAGQV-
>DmelCSPB42b
MIFVLLLLLGVTSSWATDYELLLEDPDIFSTCTDGPPGSINIRQALNLDDIVIDQKGDILHVS
GNATVVWDVQPTDRITARLDVFHFNRGTWEPTVFSMATQNFCSIMYDKNQYWYKYWTR
FITNRHEVEKKCFRGPDTVLVHEPFDLILKFENFRGPLLRGRHKLVILFNALDERNIPRPNPI
CLEIIGEPLKLQ-
>DmelCSPB42c
MRTLMLFVFGFASSWAADYELLLEDPDIFSPCTEPPPGSIGFHDAFDIGDLVVDQDMDIIHL
SESVTSIWDVEPTDRISARFAIMHYNRGSWEPTVFSMATPDFCASMFDENQSWFKYWTKH
ISNRDEVMEKCFKTRGTVIMHNPFDLQLRLTDIRGATLRGRYKAVVTFEAVDEKDVPRRN
SICFEIRGEAEKIN-
>DmelCSPA46a
MSLELHKVLLVLSIAHSALVRAGLECRIESISKVFGDNETLFEFNFRVIGRQRLLNGTLNFH
VDLDDDYEMSNEVLALKDGEWESTSVSARFKTCKYMAVIYDKYFAVSFKDSNIPKGTEA

CPIKKGEYYARNVEVIADNWAHYAKLGLVRSNMLVRKNNVVYGGFDIVLVLSQKIV-
>DmelCSPB53a
MLELILILNVVHLSLQISYEFEIEDESIYSDCSDVPPGTLNISGLFDLTNYTTTITADGLSVSG
NMTSVFNAQPTDRIELTGNLLFFDRGAWQPTTLNMMVRDFCAVMYDKKQLWYTDWSSH
VVNRDEIKDNCIKVPGTLFLIESYNMKLVFGSGIPLGTGRYSIRVQVFAYDQKGKKRPNNV
CYEVKGNFYKESNICISKPILITINVLAESLKSQMSSTNHANI-
>DmelCSPB53b
MSQQLFRIWQLLVLMAFIPHSIEVSYEFTMEDERITSDCQNEPPKTLNIDGLFDMSNIDFEM
AEDGVQLSGYKTVVWDIQPTDRVELQGSVQYFDRGTWQPTTLNMLVKDFCHVLFDKKQ
VWYDAYSKHITNSAQINNTCFRVKGSVIEFETYTIGLEFGSGIPLHRGRYAIRLKFRAFDKN
GKVRPNEICFEIKGQFSKKSLG-
>DmelCSPA56a
MSFWWMIQSLLILWTVSVGSKKSNYEVRFESIDAVKGSTETLFLYQLRLLGRNRMINGTLI
FLEDLDETFDVLFESHAFKNGYWVKGIVNAAASKPCEFFNRYYISFFLVKSTESNLPTTGA
EMCPFRKGTYFVKNGVVSTEDWPPIVFKGLNRFTISYLKNGECVGGVQLTISIAEIIT-
>DmelCSPB74a
MDFQFSVLCQILIFLTAAQPSIGGYFEYVLDDESVFSECFNTPPGYANVSGLFDVSTVNFEM
GPEGVHIDGHVTSTWDIQPTDRIEGRLNVVHMDRGTWQPTVLNMVCKDFCKTFLDPNQ
YWYNVFPKHIINKDEARQKCLNYKGTVYFVEPYTLQMHFGLGLTLPSGRNRMVINLVAID
ENNVTRPNGICFEVKGDFFKIE-
>DmelCSPA75a
MKWLVIIVLLQLLEKIKCEQSYEVTNERLEPFEGDSQTLVLFDGLKTIGRERALNGSFKFLG
EMNNDDFKVSVELYSSPNGDGEFKRMVMDVPQTSICECFKKFYVQFVQPSLKTGETTNFP
VVDDDFCPVPEGEFYVKNVILNTQDWPSQVPRGIVKAIITFFSGGKNVGGLIVEVKIEDRQ
S-
>DmelCSPA84a
MKQFLFCVLIMLIGKTCALIPRTYETRFISITSNGTNLFDFSQIRFLGRERMANGTFELKEDL
DNESFSVVGETFIDSVGDGEYKQLPFTAPKQSVCTALKAYWSYFEPSIKYGVKTDFPAHTH
PCPLPKGIYYIKDVVLKNDNWPVIMPRGYLKAVANLFKNDEYGGSLEIVSQISDLS-
>DmelCSPA86a
MLKYTVLVLLIGIPKLLLQAQMSYEAIFVSVTSEENSKPFDLSNLRLIGRERILNGTFEILED
LDDEHFQISVEIYTNPARDGNYKLLPMSVPRQGVCTFFKKYGFYFRDCIKNGINTDLFLNT
TSCLFPKGHYYLKNVTINVQNWPKIMQRGLCRHIAFFYKNNVPMGSYNLTSSIEDRAPNF
NLRPL-
>DmelCSPA87a
MGSSLCPVSYLAVLAIIVLTSNITVQAKRTFRIQKLEKVTEDTSYLRSRLRIAESEENELKVS
GYLDLNQRLDNDWTVVLKVSRSPDSDGDYEKVLTFEMQLCDFMKSYYKDIFYERIKEYS
NAPHPSSCPLPKERYVLEDYPFNVKLLKKLMSPGFYRIKYTLKNEETKILSYVLDLELEEN-
>DmelCSPB93a
MGCETSVLCTLLIFFQIIGRSFEMSHDFVPIKDDLLSKCEDKPEMGYLDAFVDLSNFSRKR
GPGGVNISGNITTIWDVDPSDVVEIDVSILKFEGDKWIPTIIKGNVKDFCKSFYDKNTLYYS
YSTKHVVNKKEAKEKCITTPGAILLWEPYLLKISFSYAVPLNVGRHKAVLIFTAIDKAGVK
RDRDICMEIVGDIVNA-
>DmelCSPB93b

MDRQQVTLLNILISLTLQIISPSFALNHFFIPEREELFSECLDKPGFSYVNELADLSRFNRKK
DADGAMNISGNITMLWDVEPSNRVAVEVNIEKFEGGTWNPTLFKGGDKDFCKNFYDKNT
IYYPFSTKHVINKQQVKDKCITTPGTVLVLEPFILKILINFAVPLSPGIHKAVIIFSAFDKSGVK
RPRDTCIEIVGEIVNI-
>DmelCSPA98a
MDAKIIILALVSLFYKANGAFYELSVADEEIFSSCPNPEPGTLDIHGLFDFSEFSTSLEADGLT
VSGNQTLVWDIQRGDRVQLFIKLFYFDRGTWTSTAFSILSQDFCKTMYDKSNVLYEPWTG
HVMNDVKDQCINAPGTKLILDTYFLSLSASVTVPLREGRYKTTIKFRAFDSKGTERPTSIC
CEVIGDVFKIRN-
>HarmCSP
MKVLLVLCLFAAAALADDKYTDKYDNINLDEILENKRLLLAYVNCVMERGKCSPEGKEL
KEHLQDAIETGCSKCTEAQEKGAYKVIEHLIKNELDIWRELAAKYDPKGDWRKKYEDRA
RANGIQIPE-
>HarmCSP1
MRAVLFVCALVYAVAAQDVSDMVNMPKYDSRYDYLDVDAVFTNKRLVRNYVDCLINAV
RCTPEGKALKRILPEALRTKCVRCTERQKRTAVKVIKRLKNEYPDEWAKLASRWDPTGDF
TRYFEEFLAKEQFNTIPGSAGIGSEIPTSSPLAPPRAPTVAPTVATPTAAATEPTPPRPVVLNR
FGDEGELMMGSPSSAGITPRPMTQATTRPTTTMRPVNTRPVPPRPTMMTWAGAASNTQPT
RFPLRPSPSDVPPPYSTAITLIDQIGYKIIKTTELVTDLLRNTVRAVVGR-
>HarmCSP2
MKTAIVLLLALFGVVLTARFDNIDIDKVLGNQRVLESYLKCMYDEGPCTPEGRDLREKAP
EALETNCKDCTDNQKALVRKASLFLIKNRPDDWKKLSDKFDPEGKYKKAFDEFLKEKN-
>HarmCSP3
MKAVFLLCLVVVAVSARPEAQYTNKYDNVNLDEILVNKRLLVPYIKCALDQGKCSPDGRE
LKSHIREALENYCAKCTPVQQDGTRRVIAHLIKHKLEEWEKLKAKYDPEGKYTHKYEKE
LEEVQH-
>HarmCSP4
MQTRYAVVLCCVVAACVAQTQRPPVSDSALEDALQDKRFIQRQLKCALGEAPCDPIGKRL
KTLAPLVLRGACPQCTPQETKQIQRTLSYVQRNFPQQWAKIVRQYAG-
>HarmCSP5
MKSLLLLCLVIAAVWARPETYDTRYDDFDAETLVENVRLLKAYGHCFLGTGPCTPEGSDF
KKTIPDALRTGCGKCTAKQRHLIRVVVQGFRSKTPDLWQQLVKKEDPNGQYKEVFTRFLN
GSD-
>HarmCSP6a
MKLIVAVALLCLVAESWAASTYTDKWDNINVDEILESQRLLKAYVDCLLDRGRCTPDGKA
LKETLPDALENECSKCTDKQKSGSDKVIRHLVNKRPEMWKELSAKYDPNNIYQDRYKDK
IEAVKGQ-
>HarmCSP6
MKADCFLFVTLIAVVAADFYNSKYDSFDVQPLLENDRILLSYTKCFLDQGPCTPDAKDFK
KVIPEALETTCGKCSPKQKQLIKTVIKAVISRHPDAWDQLTEKYDKDQKYKESFDKFLAEQ
D-
>HarmCSP7
MNSLIVFCVLSLAALTIARPDGATYTDKYDNVDLDEILGNRRLMVPYIKCMLDQGKCAPD
AKELKEHIREALENGCAKCTDKQKEGTRRVIAHLIKHKNADWQKLKAKYDPEGKYTHK

YEKELEEVQH-
>HarmCSP8
MKCIYVLSFLLALAAVQAEDKYSTENDNLDIDAVVANVDTLTSFVACFVDQEPCDAVAAD
FKKDIQEAVTTRCAKCTDAQKHIFYKFILGLKEELPRGYEEFGRKYDPENKHFSALENAVS
PA-
>HarmCSP9
MNSLIVFCVLSLAALTIARPDGATYTDKYDNVDLDEILGNRRLMVPYIKCMLDQGKCAPD
AKELKEHIKEALENECGKCTEAQKKGTRRVIGHLINHEADFWNELAAKYDPERKYTTKY
EKELKEVEA-
>HarmCSP10
MKVLVVLSCLIVAAFAADKYNAKYDNFDVDTLITNDRLLKAYINCFLDKGRCTPEGSDFK
KTLPEAIETTCGKCTDKQKNNIRKVIKAIQQKHPKEWDALVKKNDPSGKHRANFDKFIQG
SR-
>HarmCSP11
MNSAIVLCVVALAGMVLARPDGGTYTTKYDNVDLDEILANDRLLIPYIKCLLDEGKCAPD
AKELKEHIREALENGCAKCTDKQKEGTRRVIAHLIKHKNADWQKLKAKYDPEGKYTHK
YEKELEEVQH-
>HarmCSP12
MNSAIVLCVVALAGMVLARPDGDGDKYTSKWDNIDLDEILGNDRLLVPYIKCALDEGKC
APDAKELKEHILEALETGCDKCTDKQKEGTHRVIAHLIKYKLEEWEKLRAKYDPEGKYA
KKYEKELEELKRA-
>HarmCSP13
MKVLLVLCLFAAAALADDKYTDKYDNINLDEILENKRLLLAYVNCVMERGKCSPEGKEL
KEHLQDAIETGRSKCTEAQEKGAYKVIEHLIKNELDIWRELAAKYDPKGDWRKKYEDRA
RANGIQIPE-
>HarmCSP14
MNSAIVLCVVALAGMVLARPDGDGDKYTSRWDDVDLDEILENDHLLIPYIKCSLDEGKC
APDAKELKEHIQEALETGCAKCTDKQKEGTRRVIAHLIKKKLQEWEKLKAKYDPEGKYA
KKYEKELEEVKNA-
>HarmCSP15
RPESQYTNKYDNVNLDEILVNKRLLVPYIKCALDQGKCSPDGRELKSHIREALENYCAKC
TPVQQDGTRRVIAHLINHEPDYWRQLSVKYDRDGKFAVKYEKELRTIA-
>HarmCSP16
MKILVLLLAAVVTAQYEEDTYGTDHDDLDIVALVEDKDQFNSFIDCFIDEAPCDDVAETFK
SVIPEAVLEVCAKCTPAQKHIVRVFNESFKKKMPEKFQKFKNKYDPEGKYFENFEAAVGA
F-
>HarmCSP17
ASTYTDKWDNINVDEILESQRLLKAYVDCLLDRGRCTPDGKALKETLPDALENECSKCTD
KQKSGSDKVIRHLVNKRPEMWKELSAKYDPNNIYQDRYKDKIEAVKGQ-
>HassCSP
MKVLLVLCLFAAAVLADDKYTDKYDNINLDEILENKRLLLAYVNCVMERGKCSPEGKEL
KEHLQDAIETGCTKCTEAQEKGAYKVIEHLIKNELDIWRELAAKYDPKGDWRKKYEDRA
RANGIQIPE-
>HassCSP15

RPESQYTNKYDNVNLDEILVNKRLLVPYIKCALDQGKCSPDGRELKSHIREALENYCAKC
TPVQQDGTRRVIAHLINHEPDYWRQLSVKYDRDGKFAVKYEKELRTIA-
>HassCSP16
MKILVLLLAAVVTAQYEEDTYGTDHDDLDIVALVEDKDQFNSFIDCFIDEAPCDDVAETFK
SVIPEAVLEVCAKCTPAQKHIVRVFNESFKKKMPEKFQKFKNKYDPEGKYFENFEAAVGA
F-
>HassCSP17
ASTYTDKWDNINVDEILESQRLLKAYVDCLLDRGRCTPDGKALKETLPDALENECSKCTD
KQKSGSDKVIRHLVNKRPEMWKELSAKYDPNNIYQDRYKDKIEAVKGQ-
>HassCSP18
RPDTYTDKYDNVDLDEVLSNRRLLVPYVHCLLEQGKCAPDAKELKEHIREALENACGKC
TDAQQSGTRRVIGHLINKEPEFWKQLNAKYDPNNKYTKKYEKELKEVQEDKQNH-
>MsexSAP1
MKMLAVLCLFVLGALSAPERDGDLYDMFDAEMILEDDKLRSKAIDCLLDRGVCDDYQPI
RDKGPRLIKTRCEDCTPEQKAVFEESMKILEEKFNNDFKEIIAKYA-
>MsexSAP2
MKYLLVLCCVVAAVVCDDKYTDKYDNVNVDEILANERLLKGYVDCVLERGKCTPEGKE
LKEHLRDAIETGCKKCTKPQEEGATKVIDFLIKNKLEVWRELVAKFDPEGKWRKKYEDRA
RANGIVIPE-
>MsexSAP3
MKTFVALCLLSVVAVTLARPDHYTDRYDNVNLDEILDNHRVLVPYIKCILDQGKCAPDAK
ELKEHIREALETECSKCTNAQKNGTRRVIQHLINHEPEYWQELGDKYDPERKYTVKYEKE
LREIKA-
>MsexSAP4
MKMLLLVISCCVALPWALSASTYTDKWDNINVDEILESDRLMKGYVDCLLDKGRCTPDG
KALKETLPDALEHDCSKCTEKQKVGSEKVIRNLVNKRPALWKELSAKYDPNNLYQEKYK
DKIDSIKGQ-
>MsexSAP5
MKPVTAAILISLACMVQCGKDMYTSRYDSMNVDDVIGNHRLLHAYIKCMLDEGRCTAEG
RELKKHITDALQTGCSRCTDAQKKAIRHVIKHLIEHEHDFWALLVEKYDPHRIYTTKYEAE
MKRTMRSKEQMSESGAGHEKADMKMMGDGPGYKKADMKMMEGGSGHEKADMKMM
EGGPGHEKADMKMMEGGPGHEKADMKMMEGGPGHEKADMKMMDKMSSKTGMAEK
KGA-
>MsexCSP-1
MKCICLLFLVVVAVYAEEKYTEENDDLDIEGVIKDADTMKAFTGCFMDTADCDHVSGDF
KKDLPEAIQTACAKCTDKQKHITKRYFEGLEEKYPELYQAFKNKYDPENKYFAALKAAIA
KF*-
>MsexCSP-2
KTLLPEVIATSCSKCNEIQRQNVRKTVKALSNKKPDEFNEFRKKYDPNREHEKDFAAFVL
AVD-
>MsexCSP-3
FWLNPKNFTLDRWDSVDIDSILANRRLLNPYISCILEEGKCTPEGKELKSHIRDAMQTDCA
KCTPVQKAATERVIAHLLKHEHESWNKLTAKYDPTGAYTKAHHDQLKSLAA*-
>MsexCSP-4

MQVTYVLLVCVVVASCVAQQAQRPQVTDTALEDALNDKRFIQRQLKCALGEAPCDPIGK
RLKTLAPLVLRGACPQCSPQETKQIQRTLSYVQRNYPQQWAKIVRQYAG*-
>MsexCSP-5
KSHCSLLANKKHIYFVTMKTLVFFLCLVLAVLADEKYETINEDFDVAQVLENERLLNSYA
KCLLNKGPCTPEVKKVKDKLPEALETHCAKCTDRQKAMGKQLAQEVQKRYPDLWKELV
ALYDPEGKYQDAFKEFLAN*-
>MsexCSP-6
MKWQIAIALMVVVAVVSCDEKYTTKYDNINYKEILENKPLLHNYIKCTLDKGRCTAEGNE
LKSKIKDALQTGCIKCSDKQKQR*-
>MsexCSP-7
MKTILVLFALVAAVACEEYYSTQYDNFDANELVSNVRLLKNYGKCFLDEGPCTVEG-
>MsexCSP-8
MKTWLLCLVLTVVVSRSTQQNYPRNENININAIMQNARILLGYYKCVMDK-
>MsexCSP-9
TLNDKATMQRHLKCALGEGPCDPVGRRLRTLAPLVLRGACPQCSPQETRHIRRTLAYVQR
NYPWEWARIVRQYG*-
>MsexCSP-10
MQILLTHAEENSTYTTEYDGFDIREVMRNERLLTSYVNCLLDKGPCTAEGKELKKNLPDA
AQNDCKKCTQRQKENADLMIQYMEENRPADWNKLELNTSGERVAGASERHISVNTKRA
LFHT-
>MsexCSP-11
NLNRQRKYFRTFLKTMEAKCTLFILLVAMVSADFYSSKYDDFDVQPLLENDRILLSYTKCF
LDEGPCTPDAKDFKKVIPEALETSCGKCTPKQKKLIKQVIRAVIDRHPESWDKLVHKYDE
DNKYKDSFNKFLAEKD*-
>MsexCSP-12
LYLVKMRLLVIMSLFALSLVAARPDASKYPSRYDTVDLDMIVNNKKVLESYLKCVLDEGK
CTPEGKEL-
>MsexCSP-13
MRTVIVLTFLVAACFAAEKYNPKYDNFDVDTLISNERLLKAYINCFLDKGRCTPEGTDFKK
ALPEAVETTCAKCTEKQKVNIRKVIKAIQQKYPKQWEELVKKNDPSGKHRANFDKFIQGS
*-
>MsexCSP-14
MLDTMNAIRVLVFCVCMYVVVGQDINQMANMPKYDSRYDYLDVDAIFTNKRLVRNYV
DCLINAQRCSPEGKALKRILPEALRTKCIRCTERQKITAVKIIERLKYEYPEEWAKLSSRWD
PTGDFTRYFEEFLAKESFNTIPGSGSTVNEVSVTTATP-
>MsexCSP-15
RLETYCAKCTEPQKEGTRFVIGHLVKNEKEWWRKLSDKYDPERKYVTKYEAELKSIS*-
>MS|comp116602_c0
MKTLFILCALVVAVCARPEEQYTTEYDNIDIDEILNNDRLFKSYFECLVGEGKCTPAGKELK
SHMPDALQTECSKCSPKQKEGTKKVMKFLINNKPEQWKRLCAKYDPEGKYASKYEKEL
KEVSQ-
>MS|comp141234_c0
MRVLIVLSCLVVLAFAAEKYNAKYDNFDVETLISNDRLLKAYINCFLDKGRCTPEGSDFK
KTLPEAIETTCAKCTEKQKGNIRKVIKAIQQKHPKEWDDLVKKNDPSGKNRANFDKFIQG

SR-
>MS|comp142679_c0
MKFVLVLCLFAAATLADDKYTDKYDNINLDEILENKRLLLAYVNCVMERGKCSPEGKEL
KE-
>MS|comp147733_c0
MNSAIVLCVVALAGMVLARPDGGTYTTKYDNVDLDEILANDRLLVPYIKCLLDEGKCAP
DAKELKEHI-
>MS|comp148349_c0
MKTWLLCLCVLTVVVSCYSQGPNRYENFNADAIIQNDRILLAYYKCVMDKGPCTRDGKN
FKRVLPETLATACGRCNPAQKTIVRKLLLGIRTKSEPRFLELLDKYNPDRSNRDALYTFLLT
GQ-
>MS|comp148423_c0
KEHIREALENGCAKCTDKQKEGTRRVIAHLIKHKNADWQKLKAKYDPEGKYTHKYEKE
LEEVQH-
>MS|comp149055_c0
MQIKYALLLCCVAAVSLAQTQRPAVSDTALDDALQDKRFIQRQLKCALGEAPCDPIGKRL
KTLAPLVLRGACPQCTPQETKQIQRTLSYVQRNFPQQWAKIVRQYAG-
>MS|comp149356_c0
MKADCVLLATLMAVVAADFYSSKYDSFDVQPLLENDRILLSYTKCFLDEGPCTPDAKDFK
KVIPEALETTCGKCSPKQKQLIRMVVKAVIERHPEAWQQLSDKFDKDRKFKDSFDKFLAE
ED-
>MS|comp149520_c0
MKVVLLTLCLALGVLAQDKYESANDDFDVSEVLSNPRLLNSYSKCLLNQGPCTPEVKQV
KEKLPEALETRCAKCTDKQKQMGKALAQEVKKNHPDIWKQLVAMYDPQGKYQQAWQD
FLKE-
>MS|comp152291_c0
MKLLIVLALVAAALARPDDSHYDEKYDNFNIDEVITNERLLKNYAHCLIGDGKCTPEGNE
FKKLLPEATKSNCGKCTDKQKVHVAKAIKAIKEKLPTEYETLRSQIDPEGAHAEDINKYVA
KYAP-
>MS|comp152624_c0
MNALLIAIFALAVPTALAYDEKYDKIDVDKILGDDAMFTAYINCMLDKGECTQEHSADFR
KLLPEVIATSCAKCNAIQKQNVRKTVKALSEKRPDDFVAFRAKFDPKGEYEKDFTAFVMG
TD-
>MS|comp154103_c0
DGKELKTHIREALEQDCAKCTKAQRDGTRQVMGHLINHEVDYWNELKAKYDPKNLYST
KHEQELRKLKQ-
>MS|comp154103_c1
MNSFTVLCLFALVALAVARPDGKYTDRYDSVNLDQILSNRRLLVPYIKCMLDQGKCTPDG
KELKTH-
>MS|comp154133_c0
MKFVLLLCVMVAAVVADDKYTDKYDNIDLDEILSNKRLLDAHYKCVMDKGKCTAEGKE
LKDHLTEAIENGCAKCTENQEKGAQKVIDHLIKNELDMWRELAAKYDPTGNWRKKYED
RARAAGIVIPAE-
>MS|comp154647_c0

MKLIVAVALLCVVAMAWGKPASTYTDKWDNINVDEILESQRLLKAYVDCLMDRGRCTPD
GKALKETLPDALENECSKCTEKQKSGSDKVIRHLVNKRPDLWKELSTKYDPDNIYQDKY
KTQIESAKQ-
>MS|comp154792_c0
MMMMYSSLAMMLLTYLTIQSNATETSTYTTKYDGIDLDEILNNERLLIGYVNCLMDNGPC
TADGKELKKNIPDAIENDCKKCTDRQRDGSDRVMHYLIDHRPDDWVKLEEKYNSDGSYK
MKYLSSKKTEDSKETNVTKSDEETKNSSKE-
>MS|comp155046_c0
MNFLVLSVVVTLAAFATADLTYTDRYDHVNVDEILDNRKLLVPYIKCTLDQGRCTPDGKE
LKAHIKDAMQTGCAKCTKKQKKAAKKVVKHIRAKEQDYWKQIVNKYDPGNEYTETYE
AFLASPDESK-
>MS|comp156988_c0
MKSYIALLVLSVAAMALARPEEAKYTDRYDNVDLDEILSNRRLLVPYVKCILDQGKCAPD
GKELKEHIKEALENECAKCTETQKSGTRRVIGHLINHEDAYWNELTAKYDPQRKFTAKYE
KELKEIKQ-
>MS|comp158640_c0
MQIIILTALCVGLVAGLHVQAGPQMTDAQLEQTLADKNTMQRHIKCALGEGPCDPVGRRL
RTLAPLVLRGACPQCSMQETRQIRRTLAFVQRNYPWEWAKIVRQYAEFKLRTLSVT-
>MS|comp160411_c0
MVNLFLKMRAVFVLCVLVYVVVGQELNDMGNMPKYDSRYDYLDVDAIFTNKRLVRNY
VDCLINSVRCSPEGKALKRILPEALRTKCVRCTERQKRAAVKVIRRLKNDFPEEWSKLASR
WDPTGDFTRYFEEFLAKESFNTIPGSGSAIPTSSPLAPPRPIPTMPPTIASPAPGPTEPTPPRPA
ILNRFGEDGELMQGSPSSGVMTPRPMTQATPRPTTTMRSTVNSRPVPPRPTMMTWAGAAS
NTQPTRFPLRPVSDLPPPYSTAITLIDQIGYKIIKTTELVTDLLRNTVRAVVGR-
>MS|comp160989_c0
MKTILVLCVLIAAVCARPEATYDTRYDNFDVESLVENVRLLKSYGHCFLGTGPCTAEGSAF
KKTIPDALQTGCGKCSPRQRHLIRVVVNGFQTKTPDIWKQLVKKEDPNGEFKETFTRFLK
ASD-
>MS|comp599465_c0
MKLIVAVALLCLVAESWAASTYTDKWDNINVDEILESQRLLKAYVDCLLDRGRCTPDGKA
LKETLPDALENECSKCTDKQKSGSDKVIRH-
>PxylCSP1
MKSAAFIALFLIGKAVCEDKPTYTTKYDNIDLDEILSSERLLTGYVNCLLDQGPCTPDGKE
LKHTLPDAIDNDCRKCTQKQKEGSDRVMGYIIEYRPNDWAKLEKKYLSDGSYKKKYLEK
KNASENNGDSKSTEAKNKDDEEKKSKGDGEEK-
>PxylCSP2
MQKLTLACLLVAVAAAAARPNDSHYTDRYDNVNLDELISNRRLLVPYVKCVLDQGKCSP
DGKELKEHIQEALENNCGKCTDKQREGTRKMIGHLINHEQEFWDQLIAKYDPERKYVSK
YEKELKEVKASCCWLV-
>PxylCSP3
MNSLVLVCLALVAVAAARPQATYTSKYDGVNVDEILANDRLMMPYIKCALDHGRCSPEA
KELKSHIKEALENNCAKCTDKQKPAVRKVIAHLINHKPAEWRQLSDKYDPAGKYTAQYE
DQLRAVKA-
>PxylCSP4

MQTVTLLCLLAAVAAAAAAPADTYDAKYDSFNAHELVQNQRLLKSYGKCFLSKGPCTAE
GSDFKRVIPEALKTTCGKCTRKQRELVRVVVKGFQEQLPQVWTEIVSKEDPKGEYKDSFA
KFLEGSD-
>PxylCSP5
MKVVFLVFVLTAVVYSHPHDSHYTDKYDNIDLDEILNNKKILTSYINCCLDLGKCTPDGKE
LKSHIREALENKCGKCTEAQKNGTRKVMTHLINFEPDYWNQLCAKYDPEGKYKAMYEK
EYKTLVH-
>TcasCSP20
MRFFVIFFVACVSVALARPEDQYTIKYDNVNLKEILQSDRLTENYVNCLLEKKPCTPDGEE
LKRVLPDALKTSCAKCTDKQKQGAKTVIQHLYKNKQDWWKQLEAKYDPEHTYVKAHE
DELKAL-
>TcasCSP19
MKFFIAFLMLLGAVWCEQYTTKYDNINVDEILASERLLKNYFNCIMDRGACTPDADELKR
VLPDALKSDCAKCSEKQKEMTKKVIHFLSHNKQQMWKELTAKYDPDGIYFEKYKDKFDS
-
>TcasCSP18
MLFTVFLVLTCAHVVFLEEYVIPDNIDIDDILSNERLLKNYVNCLLDKGRCTPEGKKLKSTI
PEALSTDCAKCNEKVKANVRKVLHHLIDNKPDMWKQLEAKYDPSGEYRSKYKDELEKN
GIHV-
>TcasCSP17
MFKVLFVVFACVQAYVYAEEYTVPQNIDIDEILKNDRLTKNYLDCILEKGKCTPEGEELKK
DIPDALQNECAKCNEKHKEGVRKVIRHLIKNKPSWWQELQEKYDPKGEYKSRYNHFLEE
EGLN-
>TcasCSP16
MTAIVFLLALACLKTYVSSQEYLVPQNIDVDEILKNDRLTRNYLDCVLGKGKCTPEGEEL
KKDIPEALQNGCAKCNEKHKEGVRKVIHHLIENKPNWWQELESKFDPQGEYKKKYDELL
KKEGLAN-
>TcasCSP15
MIFKIHFLVFGALLTYVSSVEYLILREIDTILKNDQMTRNYLDCVLDKGKCTKEAEKLKKG
ITETMKNGCVKCEQKQKEDVHKVFQHLMIHRPNWWHELETKFNPHHEIKLQHLHQSKF
NPHEEVKLQHLHQFPHHDFLEREGFIR-
>TcasCSP14
MFATSALFAFICIQGLVSAEEYLVPQNIDLDEILKNDRLTRNYIDCILGKGKCTPEGEELKRD
IPEALQNECAKCNEKHKEGVRKVLHHLIKNKPNWWQELEAKFDPKGEYKQKYNKLLEK
EGLQA-
>TcasCSP13
MFLAIVLVVCACTNVLSEEYTNQYNDELDAALKSERLMKSYFECLLGTGKCTPSGEELKK
DIPDALKNECAKCNDKHKEGIRKVIHYLVKQKPEWWEQLQKKFDPQGIYKKRYQNYLD
KEGLKA-
>TcasCSP12
MKTLVLVLFVAVLSVVFAADKYTTKYDNIDLNQILKSDRLLKNYVNCLLDRGKCSPDGQE
LKNNLADALQTSCSKCSQRQKDGSRTIIRYLIKNKRDWWNELEAKYDPTGIYKNKYADEL
KAEGIVL-
>TcasCSP11

MKTLVPLLFFVIAIASSLAENSKYTTKYDNVDLDEIIKSDRLLKNYVNCLLEKGKCTPDGA
ELKRHLPDALHTECSKCSETQKNGSKKIMRHLIDHKRDWWNELEEKYDKEGEYRKKYE
AEIKGKKD-
>TcasCSP10
MKTFVLVAFAAVLGLALARPQEKYTTKYDNIDLEEILKSDRLLKNYFNCLMERGTCSPDG
EELKKALPDALHSGCSKCTEKQKEGSRKIIHYLIDNKRDWWNELEAKYDKDGVYRQKY
KDVIEKEGIKL-
>TcasCSP9
MTAIVFLLALACLKTYVSSQEYLVPQNIDVDEILKNDRLTRNYLDCVLGKGKCTPEGEEL
KKDIPEALQNGCAKCNEKHKEGVRKVIHHLIENKPNWWQELESKFDPQGEYKKKYDELL
KKEGLAN-
>TcasCSP8
MPLVKSLVVVVLLIGVVYQVQGQLGLAGNNYIEKQLLCALDKAPCDALGNQIKGALPEII
GKNCERCDSRQVANARRIARYVQTKHPDVWNALVKKYSV-
>TcasCSP7
MKLISAVILCAFLVAVSAAENKYTNKYDNVDVDKILNNDRVLTNYIKCLMDEGPCTSEGR
ELKKTLPDALSSGCTKCNQKQKETAEKVIRHLTQKRARDWERLSKKYDPQGQYKKRYEE
HVATSRAA-
>TcasCSP6
MIPLIAIAGILAVSAAPAEFYESRYDHLDVESILNNRRMVNYYAACLLSKGPCPPQGVDLK
RVLPEALQTNCAKCTEKQRTAAYRSIKRLKKEYPKIWEQLRAVWDPDDVFIRKFETSFESG
KPSGVISTNTSPPSPILSNRFGENEEADAASNVISSTPLPPTTSTTTRTTLTTKFTTKPSTKPT
NKPVVVTKPPQAPPFATVGANLQATVSFGTNLVGGIVRSLGTLGSRVVESGTKLANMVISA
AIRP-
>TcasCSP5
MKTFVILFFGVFFIIFSDFVNGKTLHRSTRDDKYTTRYDNVDVDRILHSKRLLLNYINCLLE
KGPCSPEGRELKKILPDALVTNCSKCSEVQKKQAGKILTFVLLNYRNEWNQLVAKYDPDG
IYRKQYEIDDDYDYSELDSAKK-
>TcasCSP4
MYSYLIPLYLFLFVHYGWSEDTTHKYTTKYDNIDLENVVKNERLLKSYVDCLLEKGRCSP
DGLELKKNMPDAIETDCSKCSEKQKEGSDFIMRYLIDNKPDYWKALEAKYDPDGTYKKR
YFESQKDEVSKVEA-
>TcasCSP3
MLFTVFLVLTCAHVVFLEEYVIPDNIDIDDILSNERLLKNYVNCLLDKGRCTPEGKKLKSTI
PEALSTDCAKCNEKVKANVRKVLHHLIDNKPDMWKQLEAKYDPSGEYRSKYKDELEKN
GIHV-
>TcasCSP2
MKIIILAVLIATAVAATYDVYPTKYDNVDIDAILHNKRLFDNYLQCLLKKGKCNEEAAILRD
VIPDALITGCRKCNDHQKVSVEKVIRFLIKERNSDWQQLISVYDPKGEYQTQYAHYLEKI-
>TcasCSP1
MLILQIAHLCAQFCLLAAIFTCVKPQLTRISDEAIESTLNDRRYLLRQLKCATGEAPCDPVG
RRLKSLAPLVLRGSCPQCTPQEMKQIQKVLAFVQKNYPKEWNKILHQYAG-

3.OBPs

>BmorGOBP1
MWKLVVVLTVNLLQGALTDVYVMKDVTLGFGQALEQCREESQLTEEKMEEFFHFWNDD
FKFEHRELGCAIQCMSRHFNLLTDSSRMHHENTDKFIKSFPNGEILSQKMIDMIHTCEKKF
DSEPDHCWRILRVAECFKDACNKSGLAPSMELILAEFIMESEADK-
>BmorGOBP2
MFSFLILVFVASVADSVIGTAEVMSHVTAHFGKTLEECREESGLSVDILDEFKHFWSDDFD
VVHRELGCAIICMSNKFSLMDDDVRMHHVNMDEYIKGFPNGQVLAEKMVKLIHNCEKQ
FDTETDDCTRVVKVAACFKKDSRKEGIAPEVAMIEAVIEKY-
>BmorPBP1
MSIQGQIALALMVYMAVGSVDASQEVMKNLSLNFGKALDECKKEMTLTDAINEDFYNF
WKEGYEIKNRETGCAIMCLSTKLNMLDPEGNLHHGNAMEFAKKHGADETMAQQLIDIV
HGCEKSTPANDDKCIWTLGVATCFKAEIHKLNWAPSMDVAVGEILAEV-
>BmorPBP2
MKLQVVLVVLTVEMVCGSRDVMTNLSIQFAKPLEACKKEMGLTETVLKDFYNFWIEDYE
FTDRNTGCAILCMSKKLELMDGDYNLHHGKAHEFARKHGADETMAKQLVDLIHGCSQS
VATMPDECERTLKVAKCFIAEIHKLKWAPDVELLMAEVLNEVSWKS-
>BmorPBP3
MARYNIVVAVLVLGVVGARGSSEAMRHIATGFIRVLDECKQELGLTDHILTDMYHFWKLD
YSMMTRETGCAIICMSKKLDLIDGDGKLHHGNAQAYALKHGAATEVAAKLVEVIHGCEK
LHESIDDQCSRVLEVAKCFRTGVHELHWAPKLDVIVGEVMTEI-
>BmorOBP5
MKQRLRVLLLRFCILQTVLSESGVDVVKNLSLSFARFFLECDEERHFQPEVRLKVMTFWY
SESSTWDRDVGCAFLCIFKKMEIDNPQDPSYRTHLELLSFANSEDNKIANQMVEIFYACGE
NTETDPCLWALEQVKCYKNRINQLGLTPTF-
>BmorOBP7
AVTEEELKIEFTKLVMKCTKDHPVDMSELMQLQQLIAPKKTESKCLLACAYKLNGVMTS
QGLYNLEHAYKIAEMSKNGDEKRLENGKKVADICVKVNDVEVSDGEKGCERAALIFKCT
LENAPKVFKFGSSEYNCQ-
>BmorOBP8
MLRVVVICVCFLVIAPYGINASSLDDLKMVYKNVIKECVGDYPITAADLKLIKARQIPNDD
IKCVFACAYKKTGMMTEEGMLSVEGIKDMSQKYLSDNPEQLRKSKEFAEACSSVNDQQV
SDGTKGCERAALIFKCSTEKITNFGFEL-
>BmorOBP9
MLRVVVICVCFLVVAPYGINAVSYEQKIKIRDQLDRAGFECFKDHKITEDDIKNLRANKPA
TGENVPCFIACVMKKTGVMNDQGVIRKGPVLELAKKVLADDKDIKKLQDYIHSCSHVNS
ETVHDKGKGCEFAMQAYTCMSANASKFGFNI-
>BmorOBP10
MLRVVVICVCFLVIAPYGINAVSDEQKIKIREQIDKSGFECFKDHKITEDDIKNLRARKPATG
ENVPCFIACVMKKTGVMNDQGVIHTEPVLQLAKKVLTDDKDIKKLQDYIHSCSHVNSKT
VHDKGQGCEFAIQTYTCMSANASKFGFDV-

>BmorOBP11
MSANSFVVLAFCALAVGVNALTEEQKAEITKSSLPLIAECSKEFSVNQGDIDAAKKLGDPS
GLNSCFVGCFMKKAGIINASGLFDVAATIEKSKKYLTSEEDLKAFEKLTETCAPENDKPVS
DSDKGCERAKLLLDCFVANKGSFSVFSL-
>BmorOBP12
MTSFMVFFVLSVLTLKYSDALTDEQKNKIQSKFIEIGAECIVEHPISIDDINSFKNKKFPSGV
NAGCFVACIFNKIGLFDDKGNLSHNSALEKAKGIFNADEEVKNLEEFLNRCAKVNGEAVG
DGVKGCERAKLAYNCLIENSLEFGFNIDF-
>BmorOBP13
MLKIHVLLCFGMAILYFGSAKAVTPEESKAFEAFAKPVIEQCQKDFGMDKESFAQKNLDEI
DECLIACVVEKFGITNDEKIDGDALKALVTKFVGNEEERNKINKIVEECTEDANKSGDGTC
NTSTILFLCLLKNGKDLWGF-
>BmorOBP14
MSRQQLKNSGKMLKKQCMGKNDVTEEEIGDIEKGKFIEQKNVMCYIACIYQMTQIIKNN
KISYEASIKQIDLMYPPELKESAKASAGRCKDVSKKYKDICEASYWTAKCMYEDNPKDFI
FA-
>BmorOBP15
MFLKNIFIECVLLYFVMLNTSFVNTMTKQQIKNSGKILKKACISKNDVTEDQISDIDKGKFI
EDKNVMCYIACVYSMSQVVKNNKFVHDAMVKQVDMMFPTEMRDAVKASIANCRGVAK
NYKDICEASFWTAKCMYEFDPANFVFA-
>BmorOBP16
MRISFLFLISVTIITFDSVFAMTRAQVKKTMTIMKNQCMPKNGVTEDQVGKIEEGIFLENH
NVMCYIACVYKTIQVVKNDRLDKDLISKQIDVLYPQEIRESTKKAVGDCINLQEKYDDWC
EGIFRSTKCLYEKDPANFIFP-
>BmorOBP17
MTRQQLKNSGKIMKKTCMPKNDVTEEEIGQIEQGKFLEQRNVMCYIACIYTVTQVVKNN
KLSYDAVIKQVDVMFPAEMRPAVKAAAENCKDISKTFKDICEASYWTAKCMYDFDPKNF
VFP-
>BmorOBP18
MILIVIAKFLILISLCETMTMKQIKNTGKMMRKSCQPKNNVDDEKINPINDGVFIEENEVK
CYIACIMKMANTMKNGKLNFEAAMKQADLLLPDEMKEPTKEAIVACRKVADSYKDVCD
ASFHVTKCIYNHNPSVFFFP-
>BmorOBP19
MTSAKTDVEIKAWFLGQAVECSKDHPVTTEELRMHKHELPDSKNAKCLMKCVFRKCNW
LDSKGMYDINAAYASSTKDFSDDKTKQENANKLFDTCKSVNEENVGDGEEGCDRSLLLA
KCLTKAAPQVSIYYS-
>BmorOBP20
MAVHIFLILASYMALAAHGQLDDEIAELAAMVRENCADESSVDLNLVEKVNAGTDLATIT
DGKLKCYIKCTMETAGMMSDGVVDVEAVLSLLPDSLKTKNEASLKKCDTQKGSDDCDT
AYLTQICWQAANKADYFLI-
>BmorOBP21
MITASLHVIFALLAFVYGGKDKPVLSEEIKEIIQTVHDECVGKTGVSEEDITNCESGIFKED
VKLKCYMFCLLEEAGLVNDDGTVDYEMFTSLIPEEYFDRATKMIFSCKELDTPDKDKCER
AFEVHKCSYEKDPDFYFLF-

>BmorOBP22
MLKVFVVVVCTLGASQLCAALYTQKVAVSFPKDKTTIVVEAMKSCIAKTGANPNVIEVIS
SGKVSEDEKFKEFFYCACNDIGVVNPDGHIKVKECIELFPKETQPLVEPVIKNCDKEGVNK
YDTLFKYLKCFQETSPVRVTLA-
>BmorOBP23
MTSKVLLSCVVLAVLATTVLAEDSRKLVSFAPEVAKKLKVLIQECLNENGLGEDAIEVIRA
GEYREDEPFQNLVYCAYKKFGALDENNRIISQVAAASFPKDIDVVTVIESCGKEDGNTPVE
QVFKYFKCFQKNSPVRMQLY-
>BmorOBP25
MKSVVLICLAFAVFNCGADNVHLNEDEREKANWYTAECGVETGVSTEVINAAKIGKYSK
DKAFKKFVLCFFKKSAILNSDGTLNMVVALAKLPSGVNKSEAQSVLEQCKNKTGQDAAD
KAFAILQCFHKGTKTHILF-
>BmorOBP26
MKSVVLICLAFAVFNCGADNVHLAETQKEKAKQYTSECVRESGVSTEAINAAKIGKYSKD
KAFKNFVLCFFNKSAIFNSDGTLNMDVALAKLPPGVNKSEAQSVLKQCKNKTGQGAADK
AFEIFRCYYKGTKTHILF-
>BmorOBP27
MKSVVLICLAFAVFNCGADNVHLTETQKEKAKQYTSECVKESGVSTEVINAAKTGQYSED
KAFKKFVLCFFNKSAILNSDGTLNMDVALAKLPPGVNKSEAQSVLEQCKDKTGQDAADK
AFEIFQCYYKGTKTHILF-
>BmorOBP28
MLKVFIVTFFAFQLSAIARLQANGCVAVPFPKDKTIIIVEAMKSCIAKTGANPNFIDVIRSGK
VSEDEKFKEFYYCTCNDTGFVNPDGHIKVKECIELFPKETQPLVEPVIKNCDKEEGVNKYD
TLFKFLKCFQETSPVRVALA-
>BmorOBP29
MTGPAAAAVLLALLAAAGQATTGCKNCVILGKEERAMFRSHSDACLAQSRVEPRLLESM
MNGELIDDAALRKHVYCVLLSCKMIGKDGKLLKAAILGKLAARPAGRDVTKVLEACAE
QPGASPEDVAWNIFRCGYNRKAVLFDYMPAGGASSGNTENHP-
>BmorOBP30
MRSFVILLNYGLLCCGQFMAEDYYYDIVTRDPDDLMREKENEVRALRAFQADCAEDVQ
VKPDLVVNLKSGDWQTEDVSLKKWALCVLMKLGLMTAQGVFKMNEAMSKIPDMNDKII
AEKLIDDCLSLQATTPHDAAWNYIKCHHQKDPEGNFSSLNIF-
>BmorOBP31
MKTFIVFVVCVVLAQALTDEQKENLKKHRADCLSETKADEQLVNKLKTGDFKTENEPLK
KYALCMLIKSQLMTKDGKFKKDVALAKVPNAEDKLKVEKLIDACLANKGNSPHQTAWN
YVKCYHEKDPKHALFL-
>BmorOBP32
MYSHKYLNDFTNIPEILIILLSSVALMSYGYNTKLFSHSLGSEPSLSILYARDKKSDKVTNEC
LMEMYPKNLYKYPLRIDRNDIPCIIHCVLKKFGIISNDGFINIKNYYRRVQAIHRYDPRILIS
DVGETCAQNINGMNLDHDVCKKAKVFNDCTQLYAISYREPEDW-
>BmorOBP33
MYAHDKLSDMIADQCLNEMYPRSKRLEIEESDEPCIIFCVLKKFGIMSPTGVINLEAYRKR
VQLPEQLAQRNSINDFGSACLESAEATQHKQDVCKKAKVFNECTHLYKILLK-
>BmorOBP34

MEKMILLNVFAVVLPCVLASRTRGSSGTLVDFTDPKVQGHLDALVRMAQSCVIKVRATPK
DVRAYFTNSSPVSRSGQCFATCMLEQSDIINHGKVNRDLLVHLAGLVNGKNSRVVRKLNS
VSRLCLDSISGMTDRCQLASTYNDCLNENMIEFAFPLDIAEEAVRKMPFHLIQPK-
>BmorOBP35
GMSTHVLDFKRNMTECLKEVQNNDKRPIKRLSPKQESPIHGECLIACVLKKNGVIQNGKV
NKDNLMALVSKFHAKETKLMKKLEKNLDRCINISVKNHDECSLASQLNDCTNDIMASSK
QKILFNY-
>BmorOBP36
MAVSEISRILTFLTIVSFIYIVYSFKPLTKDEHIERYNKMNEDIEPFRKNLTECARQVKASMA
DVEKFLKRIPQSNMEGKCFVACILKRNSLIKNNKLSQENLLEVNRAVYGDDSEVMSRLKT
AILECSKIVEDIFEICEYASVFNDCMHMKMEHILDKITMERRMEALGQMSSNPDEWSEEE
DEMLKLVKDEL-
>BmorOBP37
MFYPFRFTLLFYGLFVIYLVRAEPEKENHFTLALKKTLFSTARSCMSHVNANETDLEYLRK
DPPFPDKAACIIKCLLEKIGVVKNNKYSKMGFLTAVSPLVFTNKKKLDHYKSVSENCEKEI
NHDQTTECELGNEVVSCIFKYAPELHFKT-
>BmorOBP38
MANLVLLLTFVLMTLSMARLKSTEAPKSKTALFNDQDNMGYEELDMEEIMSACNESFRIE
YAYLESLNDSGSFPDETDKTPKCYIRCVLEKTEILSENGVLNPATAALVFAGERNGKPMSD
LEEMAVACADRHEKCKCEKAYNFVKCLMYMEIDKYEKKN-
>BmorOBP39
MVRKISALLCCFCVLGISMCDSAISTDNEQRCKNPPTAPQKIERVITLCQDEIKLSILREALD
VIKEEHTMPAERKRNKREVPFTHDEKRIAGCLLQCVYRKVKAVDGFGFPTLEGLVGLYSD
GVNERGYFMAVLEASRECLMKNHDKFSRTTPMDNGRNCDVSFDIFECISDRIGEYCGTSG
L-
>BmorOBP40
MSEFIQPSWRTQCNFRLNWDNRNRLSIDISHGAATTQTPVPTTKPKALRDFMVVPQSCDK
TTCVFKKLNIVSDKGVVDVKSFIKLLDKFTNSYPVWNSAKARVITTCLRKSLIAYDGGCEL
NNILACTFDVLSENCPLNGNNQTC-
>BmorOBP41
MLTILFLLPIVVGVLSGNIPEQPRVYCGELPNTIYSCLGNPKIIQPEVSEKCNKPISECDKTRC
IFKESGWAKNNVIDKKKVSDYFEQFAKDNPDWSAAVQNFKTTCLSDSLKPQGVDTNCPA
YDIIHCALISFIKFASPSQWSTSEQCVYPRQYAGACPVCPERCFAPSVPNGSCNACLALLRT
P-
>BmorOBP42
MMGYACVFVILAVLQAISAEDPPGLPPFLKDAPEKCKSPPRVKNPNECCISEPFFKEADFIE
CGIEKPGSERGPPDCSKQNCLLKKYNLLKNDETPDIEAIKSLLDKYIEKNPSFKSSVEKAKE
CLREDLPGPPQICLANRMTLCIGTVLLMECPDEKWNTTDDCKAFKDHMTECQKYFPK-
>BmorOBP43
MKVCVLFAIFTVAQAAKATLKPISACCNIPELGNPEPLAECSNPKLPGPCKDIQCVFEKSGF
LTENKTLIKEAYKTHLRQWAKEHEGWSVAVEKAISDCVDKDLRQYLEFPCSAYDVFTCTG
IAMLKKCPNEHWTC-
>BmorOBP44
MSRLVLFFTILVVLQEFIINLYFNFITEIDSCCVKKYPKLFDSEFITECYNTQRKANDKCERD

MCVARKLNLLTEEDSINKDALLRFVEEGFKTEIDLVNAIKKKCFEEDISNIGKPEMCEVAK
YKICITSRMAEDCPKWDSKGICSSAQQKVENFMKMLS-
>DpleGOBP1
MKKLTTGFVKAMEECKAELNLGDHIIQDFMNYWREEYELLNRDTGCAIMCMASKHDLIT
EDMKIHHENAHEFAKSHGADDDLAKQLVQMIHDCEKQFTDITDDCSKTLEISKCFRTKIHE
LKWAPSMETILEELMTET-
>DpleGOBP2
MAVRGLLLLSAVLAATSVTVDGTAEVMSHVTAHYGKSLEECRKETGLSKEILEEFKHFWS
DDFEIVHRSPFTVRLHPHLHDDARMHHINMNDYVKGFPEGDVLADKLVQMIHKCEKEYD
DIKDDCDRVVKVAACFRADAKKEGIAPEVAMIEAVMEQY-
>DplePBP1
MARDILHIWEESYDLNHDETGCLVLCAMVRLELLDQQGNMIVENTEGFIRANGGDDSMV
SFLIQLYSMCREKTSSISNGCKAAIELSKCFRAAIQQIGWVPDTTSLLVISYD-
>DplePBP3
MNKSCVFVFALIFVNVQKVQSNEVMKGITSSFFKVLDECKRELGLTDNVLTDLYYFWKQ
DHPLMHRDTGCAIVCMSQKLNLLDTIGKLHHGNAQEFAINHGAGEQMAKKLVTMVHEC
EQQFMEQEDSCLRALDVAKCFRTAMHDVNWAPKFDIIVTEVLTEVK-
>DpleABP3
MPSLSLLLYLAMLTLCYGAKEKPELSEEIKEIIQHVHNECVGKTGVAEEDIANCENGIFKED
MKLKCYMFCLLEEGSLVDDDGNVDYDMMVSLIPDQYTDRVLNMINGCKHLDTPDKNKC
QRAFDVHKCSYSKDPDFYFLF-
>DpleABP4
MTRAQLKKTMTIMKNQCMPKHNVNNDKVGQIEQGVFIEDHDVMCYIACIYKTIQVVKN
NRLDKDLISKQIDALYPPELKESTKEAVSKCIKEQAKYEDPCAGIFYACKCLYEDNPANFIF
P-
>DpleABP5
MRVLVVAAVTLITITLTRCVPRTDKMQNNVKDLTSTTPNSMEEQMDKISNDLRPVMAECN
ETFRIEMYYLETLNTTGSFPEELDRTPKCFLRCVLEKAEVASADSQFDVSRTADVFDQIRVL
PHDDLVKMATTCSDRAETCKCERAYQYLKCLMGMIINKYDTT-
>DpleABPX
MAELAKMLRDNCLEETGADVALIDKVNQGATLMPDGKLKCYIKCVMETAGMMSGGEV
DVEAVVAVLPEELQRHADTMRACGTKRGLDDCDTAFLTQACWQAGCPKDYILI-
>DpleABPfmxg18C17
MYLRLVALFFFTSYATANLASVITKCSDEDTKCLKDFTQNLIPLFAKGDPSMKVDPIDPIFV
KTINASSPNLKLVLNDISLTGLKDCVVKKAKRDLQKNKFLFKLMCSVNLEGQYVMDGRL
LILPINGKGRNHVSLDKALFVIETDVAENILEDGKKHWIIPKWSYKYELKEKSNIIFENLFN
GNEVLSKAAEEVIKDNGNEIIQEVGEPIIEYIIGKVVDSMRQFYTAVPVEELVQN-
>DmelObp8a
vpmrsspqslallrardqcgreltaaqrlqldrmqfedaahvrhylhcfwsrlqlwldetgfqaqrivqsfggerrlnveqalpaingcnak
tssrgsgaqtvvdwcfrafvcvlatpvgewykrhmsdvingna-
>DmelObp19a
Gvteeqmwsagklmrdvclpkypkvsvevadnirngdipnskdtncyincilemmqaikkgkfqlestlkqmdimlpdsykdeyr
kginlckdstvglknapncdpahallsclknnikvfvfp-
>DmelObp19b

Deeegsmtvdevveliepfgdactpkpsrenivemvlnkedakhetkcfrhcmleqfelmpedqlqynedktvdminmmfpdred
dgrrivktcneelkaeqdkceaahgiamcmlremrssgfkipeike-
>DmelObp19c
Qtqafdlakllpktgtepiwavidrnlpqvqelvtaarmeciqklqlprdqrplgkvtnpsekekclvecvlkkiklmdadnklnvgqve
kltslvtqdnkmaiavsssmaqacsrgissknpcevahlfnqcisrqlernnvklvw-
>DmelObp19d
Kpheeinrdhllelaneckaetgatdedveqlmshdlperheakclracvmkklqimdesgklnkehaielvkvmskhdaekedapa
evvakceaietpedhcdaafayeeciyeqmrehgleleeh-
>DmelObp28a
Fdekealaklmesaescmpevgatdadlqemvkkqpastyagkclracvmknigildangkldteaghekakqytgndpaklkiald
igetcaaitvpddhceaaeaygtcfrgeakkhgll-
>DmelObp44a
Sdyklrtaedlqsarkecaasskvtealiakyktfdypddditrnyiqcifvkfdlfdeakgfkvenlvaqlgqgkedkaalkadiekcad
kneqkspanewafrgfkcflgknlplvqaavqkn-
>DmelObp46a
Rstppaldedcelnsvdtmhdfccdlhdespqfsdcqmewhekipyetdeeeqtymfctaecsfnstnflgrdrrslnlnevkehlesdl
vndadikllydtyvkcdkhalslmphkgvkqlskrlsrlgchpypglvlecvanemilhcptkrfrqtaqceetrnhlkqcmqylkyks-
>DmelObp47b
Qatidcqrppqlvdpalcckdggrdqvaeqcaqrilgtangqkaggppsldtaaclaeciltsskyidepqklnlanirsdlsakfsndtly
vetmtmafskcepqsqrrlamimqqqqvqqqktqqqprcspfsaivlgctymeyfkncpdhrwtpnaqctlakayvtqcglga-
>DmelObp49a
Dvdcskrpsfvnpktccpmpdfvtaelkqkcikfdmtpppppdgeasgsfeskrrhhhphpppcffscifnetgiyqnrkldeaklna
ylqevfedssdlqttatqafttcatkvadfeanlpprpapspppgfpmcphdaghlmgcvfrnmmkncpdsirndsqqctdmkefftk
ckpprgpppsaedm-
>DmelObp50a
Akcraapksvqnvhvccsaplpnwgvfnrechksaiqasvsinriskskvnlanflikcrldcdfnassvlqgnrliqakvrpmlerafs
neptidayesnfakcstvvrskyqelsplsrqsdacdrhalfyslcayarliftcpdkmwqrnnrmcqeakayakkcpwpalkmfmrn
t-
>DmelObp50b
Vsndmgglqkctellnthklvyccgksfldkfpfvgsnctpfwddygpcryeclyrhwdlldqdnkikkpelylmitslysplngydk
ygaafkaahetcealgsrhadflllysnqvadkmgmasstclpyamlhaqctmvyltancprenwiddpkcnslqkllssctkkldekt
nalkgkdeeltdngcghidsegsnllmacfltlmiakfisdh-
>DmelObp50c
Dpidvdctrrqdfnivkdccvyptfrfdqfksqcgkympvgaprispclyecifnktntvvdgaihpdnarlmleklfgnqdfeeayfn
glmgcsdsvqemisnrrsrpqrkteqcspfslfygicaqryvfnhcpssswsgtescemarlqnmncskpsrgsshrl-
>DmelObp50d
Adpicsqrpdvtalrncck lpnldfssfnskcsqylvngvhispcsfecifraanalngthlvmeniekmmktilgsdefvhvyldgfrsc
gnqekvlikamkrrrvpitgkcgsmaimyglcahryvyrncpesvwsksatcneareysircddm-
>DmelObp50e
Sfncsappnfnnfdintccrtpeldmgdvpqkchkyvsglksanskypsyahlcypdciyretgamvngkikvnrvkqyleehvhrr
dqeivshivqsfesclsnvkghmkslniesykvlphgcspfagiiyscvnaetflncpqqmwknekpcnlakqfaeqcnplphvplps
s-
>DmelObp51a
Lfeseanecakklgitpdyfenfphssrvkcfyhcqmekleiiangvvtpfdlkvlnispesydkygvkvkpclklshrdkcelgylvfq

clkrefnl-
>DmelObp56a
Sslnlsdeqkdlakqhreqcaeevklteeekakvnakdfnnptenikcfancffekvgtlkdgelqesvvleklgaligeektkaalekcrt
ikgenkcdtasklydcfesfkpapeaka-
>DmelObp56e
Vgltdsqkaeakqrakacvkqegitkeqaialrsgnfadsdpkvkcfancfleqtglvangqikpdvvlaklgpiageanvkevqakcd
stkgadkcdtsyllykcyyenhaqf-
>DmelObp56f
Mkssekikaclkrqlgytitentkfdakedslqskcfyhcllevkgviandaisseqprkvlekkygitdtdelekaeekchsikasgkcel
gyeilkcyqsitkh-
>DmelObp56g
Qqanidssvskelvtdclkengvtpqdladlqsgkvkaedakdnvkcssqcilvksgfmdstgklltdkiksyyansnfkdviekdldr
csavkganacdtafkilscfqaan-
>DmelObp56h
Npdfrqimqqcmetnqvteadlkefmasgmqssakenlkcytkclmekqghltngqfnaqamldtlknvpqikdkmdeissgvna
ckdikgtndcdtafkvtmclkehkaipghh-
>DmelObp56i
Gpikdqcmaaagitaqdvanrhetddpghsvkcffrcflenigiiadnqiipgafdrvlghivtaeavermeatcnmiksetshdescef
awqisecyegvrlsdvkkgqrtrnhrg-
>DmelObp57a
Iqslslleetnyvsdclasnnisqaefqelidrnsseeddlentdrrykcfihclaekgnlldtngyldvdkidqiepvsdelreilydckkiyd
eeedhceyafkmvtcltesfeqsdevteagkntnklne-
>DmelObp57d
Ntsvfnpcvsqnelseyeahqvmenwpvppidraykcfltcvlldlglidergnvqidkymksgvvdwqwvaielvtcriefsderdl
celsygifncfkdvklaaekyvsisnak-
>DmelObp57e
Ndpcphnqgidediaesilgdwpanvdltsvkrshkcyvtcilqyynivtasgeifldkyydtgvidelavapkinrcryefrmetdycs
rifaifnclrqeiltks-
>DmelObp58a
Lkcrsqeglseaelkrtvrncmhrqdededrgrggqgrqgngyeygygmdhdqeeqdrnpgnrggygnrrqrglrqsdgrnhtsnd
ggqcvaqcffeemnmvdgngmpdrrkvsylltkdlrdrelrnfftdtvqqcfrylesngrgrhhkcsaarelvkcmseyakaqcedwe
ehgnmlfn-
>DmelObp58b
Vrvhcrhmeriheenihhcckhqdghddvtescakqtnfrlpspneeaivdvtvdqamvgtcwakcvfdhynlmenntldmdkvr
syykryhqtdpeyatemlnayekchtqseeatekflslpivrafstakfckptssiimscviynffhncpasrwsnttecvetlafarkckd
vltt-
>DmelObp58c
Ikidcenteainedhihycckhpdghndliegcaretnftlpnqneealvditadrairgtcfgkcvfsklnlmkdnnldmdavrslfterf
pddpeyakeminafdhchgkseentsmflskplfkqmskqfcdpkssvvlacvirqffhncpadrwsktkecedtlafskkcqdslatl
-
>DmelObp58d
Qdneettavaissgdltedkcntsragccselyigeeedlvkcfvihspklpvdgdadigktlrflscfveclykqkkyigksdtinmkmv
kldaektfvdrpkekdyhiamfefcrkdavgvynllkaspgakvllkgacrpyllmvfmcisdyhqkhecpyfrwegtakagtkdmc
enakaecyqidgitlptkspa-

>DmelObp69a
Veinptiikqvrklrmrclnqtgasvdvidksvknrilptdpeikcflycmfdmfglidsqnimhleallevlpeeiyktinglvsscgtqk
gkdgcdtayetvkcyiavngkfiweeiivllg-
>DmelObp76a
Mtmeqfltsldmirsgcapkfklktedldrlrvgdfnfppsqdlmcytkcvslmagtvnkkgefnapkalaqlphlvppemmemsrk
sveacrdthkqfkescervyqtakcfsenadgqfmwp-
>DmelObp83a
Qrdenypppgilkmakpfhdacvektgvteaaikefsdgeihedeklkcymncffheievvddngdvhleklfatvplsmrdklme
mskgcvhpegdtlchkawwfhqcwkkadpkhyflp-
>DmelObp83b
Qeprrdgewpppailklgkhfhdicapktgvtdeaikefsdgqihedealkcymnclfhefevvddngdvhmekvlnaipgeklrni
mmeaskgcihpegdtlchkawwfhqcwkkadpvhyflv-
>DmelObp84a
Lqdhakdngdifiinydsfdgdvddistttsapreadyvdfdevnrncnasfitsmtnvlqfnntgdlpddkdkvtsmcyfhcffeksgl
mtdyklntdlvrkyvwpatgdsveaceaegkdetnacmrgyaivkcvftraltdarnkptv-
>DmelObp85a
Mspgsvvfsmfltrpsldkgnsecrkslnlpahrkfnfaelytinmcieecnfigcgyieidppfrldlanirtnlqtiapqpqnesipflvd
ayrkcelfrsshgrrftlhlpdiefieepcnpfalqiticvrihamqkcpsefyvdsdecrlareyftqcvgdietnla-
>DmelObp93a
Cdvqkndkainscrksllgnnstnsngevrnlksdkvalhaciaecsfrtngfllsngtvntqalqksyqqryndpnmsqlmlkslnsc
tdyarkrvqefqwmpkkgdcdfypatllacvmekvyincptskwkntsdctamwkylvacddvasnkkk-
>DmelObp99a
Dyvvknrhdmlayrdecvkelavpvdlvekyqkweypndaktqcyikcvftkwglfdvqsgfnvenihqqlvgnhadhneafhas
laacvdkneqgsnacewayrgatcllkenlaqiqkslapka-
>DmelObp99b
Ddwtpktgeeirkirvdclkenplsndqisqlknlifpnepdvrqyltcsaiklgifcdqqgyhadrlakqfkmdlseeealqiaqscvdd
naqknptdvwafrghqcmmaskigdkvrafvkakaeeakkkaa-
>DmelObp99c
Asvwklptaqmvyedlekcrqesqeedaatlrclvkklglwtdesgynarriakifaghnqmeelmlvvehcnrmeqdtshlddwaf
layrcatsgqfghwvkdfmsqkever-
>DmelObp99d
Dhhhhhhdyvvkthedltnyrtqcvekvhaseelvekykkwqypddavthcylecifqkfgfydtehgfdvhkihiqlagpgvevhe
sdevhqkiahcaethskegdscskayhagmcfmnsnlqlvqhsvkv-
>DmelObp73a
VEYLIRFETKKAKCLNPPRTARKVESVIRECQDEVRNKLVNEAYEILKEQVSQNQPPIDPN
DDSIDFIWPSVPEAPSLDHSPNISQYEYIVYDEPEPQRHVARLMRNIRRLDVASSGIYHPTLV
PLEDKRIAGCLLHCVYAKNNAIDQRGWPTLDGLVHFYSEGVHEHGFFMATLRSVNLCLRT
MTARYGVNRKELPKKGESCDLAFDVCSHMNTNIFKDLQFWAPFISYVNESRAINYI-
>HarmPBP1
MEFHRSTMMSVRLALVVAAWLFIRVDASQDVIKNLSMNFAKPLEDCKKEMDLPDSVTTD
FYNFWKEGYEFTNRQTGCAILCLSSKLELLDQELKLHHGKAQEFAKKHGADDAMAKQL
VDLIHGCAQSTPDVADDPCMKTLNVAKCFKAKIHELNWAPSMELVVGEVLAEV-
>HarmPBP2
MADSRWLFARVFCLVLMMGSAMSSKELLTKMTGGFTKVVDACKTELSVGDHIMQDMY

NFWREEYQLVNRDLGCMIMCMTAKLDLIGDDQKMHHGKAEEFAKSHGADEALAKQLV
GLIHGCETQHQAIEDHCSRALEIAKCFRTKIHELKWAPSMEVIMEEIMTAA-
>HarmPBP3
MGSRHVFFALVVLAVSVRKAEPSKDAMQYITSGFVKVLEECKHELDLNEQILADLFHFWK
LEYSLLGRDTGCAIICMSKKLDLLDANGRMHHGNAAEFAKKHGAGDEVASKIVTIIHECE
KKHEQDGDECLRVLEVAKCFRTGIHELDWQPKVEVIVSEVLTEI-
>HarmGOBP1
LLADINVMKDVTLGFGQALDKCREESQLTEEKMEEFFHFWRDDFKFEHRELGCAIQCMS
RHFNLLTDSSRMLHDNAEKFIQSFPNGEVLARQMVELIHSCEKQFDHEDDHCWRILHVAE
CFKGSCVQRGIAPSMELMMTELIMEAESR-
>HarmGOBP2
MTSKSCLLLVAMATLTGSVIGTAEVMSHVTAHFGKALEECREESGLSAEVLEEFQHFWRE
DFEVVHRELGCAIICMSNKFSLLQDDSRMHHVNMHDYVKSFPNGHVLSEKLVELIHNCE
KKYDTMTDDCDRVVKVAACFKVDAKAAGIAPEVAMIEAVMEKY-
>HarmOBP1
MSKFTFFVLCVVAVSLSKVYASDEDKAKLHEALKPLVEECMKDHEVSLDDLKAAKEAKS
ADGVKPCFLACVYKKAEVLNDKGEFDADHALEKLKEFVSDEDVLAKVAEVGNTCKAVN
DKAVSDGDAGCERAALLTACFLEHKAEILV-
>HarmOBP2
MMDRKRLCLLIIALFLAQGSDAMSRQQLKNSGKMLKKNCMNKNQVTEDQIGSIDKGKF
VEDKKVMCYIACIFEMTNVVKNNKLNYDASIKQIDLMYPPDLKESAKAAVEKCKDVQKK
YKDICEASYWTAKCMYDFKPEDFIFA-
>HarmOBP3
MSKFTCFVLCVLAVSLGEVRSNALEKAAIRAAVYPLIVDCAKEHGVTLEQLKAAKASHSA
EGINPCFQSCVYKKTGIFNDNGEYDVANAKTKLQKFVTDEDEYARIAEVGKTCASVNDKS
VSDGAAGCERAALLTACFLEHRAQIII-
>HarmOBP4
MSKLTCVVFAAVAVVFSNVNADDETRASFRQVLGPLVMECRNEFGITEDDLKKAQQERSP
DALKPCFIACVFKKFGIITSAGKYDSDASISRIKDVVKNDDLLAKLKSVGEKCNSVNDASV
SDGDAGCERAALLAKCFIENKSELSI-
>HarmOBP5
MSKFTCLVLCVVAASLSQAYASEEEKAAFREAIKPIVEECSKEHGVSHDELKSAKDNQNA
DSIKPCFLGCVYKKAEVFNSKGEYDVDKALEKLKKFVSNDEAYAKFAEVGKKCASVNDK
AVSDGDAGCERGALLTACFLEHKAEVPL-
>HarmOBP6
MSKFTCLLLCVVAVSLSKVHATEEEKEAIRAAVRPIMQECGKEHGVTLDDLKAAKAAHSA
DGIKPCFQSCVYKKAGIFNDNGEYDIANAKTKLQKFVTNDEEYARIAEVGKMCASVNDK
PVTDGAAGCDRAALLTACFLEHRAQIII-
>HarmOBP7
MFRFGVLSFVVLLFCMESSYALSSEEELSIKEALHPFVVECAEEYGMTEEMFEEAKKKGS
AEDIDPCFMSCFLKKTGFFDDSGKFDAEKSISFAKEHITSESAIKFLEAGAGECVKINDEDV
SDGENGCDRAKLLFDCLTELKKKMSE-
>HarmOBP8
MLLIEIVKFLTLVAMCEAMTMKQIRNTGKMMRKSCQPKNNVADEQIDPIAEGVFNEDKEV

KCYMACIMKMANTIKNGKLNYEAAIKQADLLLPDDIKEPAKEAITACRKVADAYKDICDA
SFHITKCIYTQNPGIFYFP-
>HarmOBP9
MCKFSVLFLYSAVMAVNIWSASCISEEDKAAIITAIAPLAQNCGSECGLDNDDFEKYKEDG
SDMDPCFKACLMTQMGVLDKEGKYDGKGLHKAMEEADYPGDKDDAQKFLDELDRCFD
AKGDNSGSDEEAKMKRADVLFRCMQDMKEK-
>HarmOBP13
MFTGTLPLVVFLATFAYGGKEKPVFSDEIKEIIQTVHDECVAKTGVAEEDITNCENGIFKED
PKLKCYMFCLMEEASLVDDDDAVDYDMLVSLIPEEYVDRTTKMIFSCKHLDTPDKDKCQ
RAFEVHKCSYEKDPDLYFLF-
>HarmOBP16
MFKSIVFCALIIVASHADVLKKRDSKGASLKPLSVCCDIPELGDPKNLEKCSNPKMPGPCD
DIQCIFEASGFLIDRNTLNADAYKNHLMKWQEEHKPWKVAVDRAIEECANNQTRQYLDFP
CKAYDVFTCTGIAMLKKCPEAAWKC-
>HarmOBP17
MKTFVILAACVMLVQASGLTDEQKEKLKKHRSECLTETKVDEQLVNKLKGGDYKTESEP
LKKYALCMMMKSELMTKDGKFKKDVALAKVPNAADKPTVEKLIDACLANKGNTPHQT
AWNYVKCYHEKDPKHAIFL-
>HarmOBP18
MKSFVVFCVLVAGAFAANVSLPPKQNEKANQIATECMKESGLKPEVLAEAKKGHISDDEH
LKKFTFCFFKKAGIVSEDGKLNTEVALAKLPPGVDKAEAEKLLETCKGKTGKDVTDTVFE
IFKCYHHGTKTHILLGF-
>HarmOBP19
MEQCGIKRASGEGSEELEKIQPGPKVPCKEGICLMQKANLLQENNSVDYTKLRSFLDQWA
DTNAEFTDAILTAKKICAQDGGPAGPPVCEQDRIFFCLTSNILWNCNLRKLDGCDILQEHM
DECRQYYVQDEPEE-
>HassGOBP2
MTSKSCLLLVAMATLTASVMGTAEVMSHVTAHFGKALEECREESGLSAEVLEEFQHFWRE
DFEVVHRELGCAIICMSNKFSLLQDDSRMHHVNMHDYIKSFPNGHVLSEKLVELIHNCEK
KYDTMTDDCDRVVKVAACFKVDAKAAGIAPEVAMIEAVMEKY-
>HassGOBP1
MDINVMKDVTLGFGQALDKCREESQLTEEKMEEFFHFWSDDFKFEYRELGCAIQCMSRH
FNLLTDSSRMHHDNTEKFIQSFPNGEVLARQMVELIHSCEKQFDHEDDHCWRILHVAECF
KGSCVQRGIAPSMELMMAEFIMEAESL-
>HassPBP2
MMGSAMSSKELLTKMSEGFTKVVDACKTQLNVGDHITQDMYNFWREEYQLVNRDLGC
MIMCMVAKLDLIGDDQKMHHGKAEEFAKSHGADDVLAKQLVSLIHSCETQHQAIEDHCS
RVLEIAKCFRTKIHELKWAPSMEVVMEEIMTAA-
>HassPBP3
MGSRHVFFAFAVLAVSVRKAEPSKDAMQYITSGFVKVLEECKHELNLNEQILADLFHFWK
LEYSLLGRDTGCAIICMSKKLDLLDANGRMHHGNAAEFAKKHGAGDEVASKIVTIIHECE
KKHEQDGDECLRVLEVAKCFRTGIHELDWQPKVEVIVSEVLTEI-
>HassPBP1
MNFAKPLEDCKKEMDLPDSVTTDFYNFWKEGYEFTNRQTGCAILCLSSKLELLDQEMKL

HHGKAQEFAKKHGADDAMAKQLVDLIHGCSRSTPDVTDDPCMKALNVAKCFKAKIHEL
NWAPSMDLVVGEVLAEV-
>HassOBP11
FCVLVAGAFAANVSLPPKHNEKANQIATECMKESGLKPEVLAEAKKGHISDDEHLKKFTF
CFFKKAGIVSEDGKLNTEVALAKLPPGVDKAEAEKLLETCKGKTGKDATDTVFEIFKCYH
HGTKT-
>HassOBP19
MIRSCFVLVAVLQVLGVSAQEGPGGPPGDPRQHPILSKIPRKCWAPPPGIDIYRCCPIPKLYP
DEIMEQCGIKRASGDPSEEPEKPQPGPKVPCKEGICLMQNANLLQQNNSVDYTKLRNFLD
QWADTNAEFTDAILAAKKICAQDGGPAGPPVCEQDRIFFCLTSNILWNCNLRKLDGCDILQ
EHMDECRQYYVQDEPEE-
>HassOBP17
MKTFVILAACVMLVQASGLTDEQKEKLKKHRSECLTETKVDEQLVNKLKGGDYKTESEP
LRKYALCMMMKSELMTKDGKFKKDVALAKVPNAADKPTVEKLIDACLANKGNTPHQTA
WNYVKCYHEKDPKHAIFL-
>HassOBP16
MFKSIVFCALVIVASHADVLKKRDSKGASLKPLSVCCDIPELGDPKNLEKCSNPKMPGPCD
DIQCISEASGFLIDRNTLNVDAYKAHLTKWEEEHKSWKVAVDRAIEECANNQTRQYLDFP
CKAYDVFTCTGIAMLKKCPEAAWKC-
>HassOBP13
MFTGTLPLVVFLATFAYGGKEKPVFSDEIKEIIQTVHDECVAKTGVAEEDITNCENGIFKED
PKLKCYMFCLMEEASLVDDDDAVDYDMLVSLIPEEYVDRTTKMIFSCKHLDTPEKDRCQ
RAFEVHKCSYEKDPDLYFLF-
>HassOBP9
MCKFSVLFLYSAVMAVNIWSASCISEEDKAAIITAIAPLAQNCGSECGLDNDDFEKYKEDG
SDMDPCFKACLMTQMGVLDKEGKYDGKGLHKAMEEADYPGDKDDAQKFLDELDRCFD
AKGDNSGSDEEAKMKRADVLFQCMQDMKEK-
>HassOBP3
MSKFTCFVLCVLAVSLAEVRSNALEKAAIRAALYPLIVDCAKEHSVTLEQLKAAKAAHSA
QGINPCFQSCVYKKTGIFNDNGEYDIANAKTKLQKFVTDEDEYARIAEVGKTCASVNDKS
VSDGAAGCERAALLTACFLEHRAQIII-
>HassOBP7
MSRFCVLSFVVLLFCMENIYALSSEEELSIKEALHPFVVECAEEYGMTEEMFEEAKKKGSA
EDIDPCFMSCFLKKTGFFDDAGKFDAEKSISFAKEHITSESAIKFLVAGAGECVQINDEDVS
DGDKGCDRAKLLFDCLTDLKKKLSE-
>HassOBP-8
NTGKMMRKSCQPKNNVADEQIDPIAEGVFNEDKEVKCYMACIMKMANTIKNGKLNYEA
AIKQADLLLPDDIKEPAKEAITACRKVADAYKDICDASFHITKCIYTQNPAIFYFP-
>HassOBP-7
MCKLTCLVLAAVAVVFSNVNADDEPRASFRQMLGPFVMECKKEFDITEDDLKKAQQEHS
PDALKPCFIACVFKKFGIITSAGKYDSDASISRIKDVVKNDDLFAKLKSVTEKCNSVNDAS
VSDDDAGCERAALLAKCFIENKSEISFN-
>HassOBP-6
MSRQQLKNSGKMLKKNCMSKNQVTEDQIGSIEKGKFVEDKKVMCYIACIFEMTNVIKNN

KLNYDASIKQIDLMYPPDLKESAKAAAEKCKDVQKKYKDICEASYWTAKCLYDFKPEDFI
FA-
>HassOBP-5
MLNLFIVVLALCSSVAVYALTEEELKLKFTKLIMKCNKDSEVDMQELVQLQSYVVPTKTAT
KCVLACAYKAASVMNAQGLYDIDHAYKVAEMMKNGDEKRLTNAKKMTDICVKVNDIK
VSDGEKGCDRAALIFKCTVDNAPKFGFKL-
>HassOBP-4
MFKLFVFLAFTVATCYGAQGQGVLCGPLPRRLTRCLNMAPAISGEIQDKCHESRTATECER
LTCIFREYNLLDGTTVNKDRTNAFLDNYVKQYPVWTTAVQHAKAACLGDVELKPQGIDL
NCPIYDTLQCIFSSLIKHATPSQWSTTSECQGYRAFAAACPICPEDCFAAQVPIGSCNACLSL
P-
>HassOBP-3
HAGDEDKAKLHEALKPLVEECMKEHEVSLDDLKAAKEAQSADGVKPCFLACVYKKAEV
LNSKGEFDADHALDKLKEFVSDEDVLAKVAEVGNTCKAVNDKAVGDGDAGCERAALLT
ACFLEHKAEVKPIRPLLFPWAHHHRH-
>HassOBP-2
MSKFSCLVLCVVAASLSQAYASEEEKAAFREAIKPIVEECSKEHGVSHDELKSAKDNQNA
DNIKPCFLGCVYKKAEVFNSKGEYDVDKALEKLKKFVSNDEAYAKFAEVGKKCASVND
KAVSDGDAGCERGALLTACFLEHKAEVPL-
>HassOBP-1
MSKFTCLLLCVVAVSLSKVHATEEEKEAIRAAVRPIMEACGKEHGVTLDDLKAAKAAHSA
DGIKPCFQSCVYKKAGIFNDNGEYDIANAKTKLQKFVTNDEEYARIAAVGKTCASVNDKP
VTDGAAGCDRAALLTACFLEHRAEIII-
>MSexGOBP1
MGQDTRSLVLVVLVGLVGAVSADVQVMKDVTLGFGQALEQCREESQLTEEKMEEFFHFW
REDFKFEHRELGCALQCMSRHFNLLTDSSRMHHENTDKFIKSFPNGAVLSKTMVELIHNC
ELQHDAEEDHCWRILRVAECFKISCTKAGIAPSMEVMMAEFIMELKQ-
>MSexGOBP2
MVNRLILVVVVVFITDSVMGTAEVMSHVTAHFGKALEECREESGLPVEVMDEFKHFWRE
DFEVVHRELGCAIICMSNKFELLQDDTRIHHVNMHDYIKSFPNGQVLSEKMVQLIHNCEK
QYDDIADDCDRVVKVAACFKKDAKKEGIAPEVAMIEAVIEKY-
>MSexPBP1
MKVAVVAIVVYLAVGNVDSSPEVMKNLCLNFGKALDECKAEMNLSDSIKDDFANFWVEG
YEVSNRDTGCAILCLSKKLDMIDPDGKLHHGNAMEFAKKHGADEAMAKQLLDIIHNCEN
STPPNDDACLKTLDIAKCFKKEIHKLNWAPNMDLVVGEVLAEV-
>MSexPBP2
MVSTKWRLLVVTIAVLTMEMVSASQEVLKQMSVGFSKVLQTCKTELSVGDHIIQDFYNY
WREDYDLLNRDFGCMVICMAVKHDLINDQLTMHHGNAHAFAKTHGADDDTAQQLVTIL
RECEAKHQSVEDVCNRALEMAKCFRTKIHELKWAPAMEVVLEEIMTSV-
>MSexPBP3
MAIIPIFTVLLMMTAVKEIAPSSDAMRHIANGFLKVLDQCKHELGLTDQIVVDLYQFWKLQ
YALLNRDTGCAIICMSKKLDLLDSTGRMHHGNTQEFAVSHGATDEVASKVVVIIRDCEKQ
QEGEQDDCVRVLEVAKCFRTAIHELNWAPNMEVVVDELLTEI-
>MSexABP1

MSVISFFVLCFGVLAVSVGAVSENERNQISQSILPHIVKCSQEYGVSEGQIKDAKESVNPLG
LNPCFLGCVLKSAGIIDKNGLFDVEATKEKSKKYISSEKDVTNFDKIIKDCTEVNQKNVSD
GNKGCDRAKELVTCFLAKRGDFSVFTF-
>MSexABP2
MDRKDLCLLIIAFILADGVDSMSKQQLKNSGKMFKKQCMGKNKVTEDEIGEIDKGRFVE
QQNVMCYIACIYQMSQVVKNNKLNYEASLKQIDIMYPPELKDTAKGALEACKDIAKKNK
DLCEASFKTAKCMYEYSPKDFLFP-
>MSexABP3
MITATLHVVFALLGFVYGAKNKPVFSEEIKEIIQTVHDECVGKTGVSEEDIANCENGIFKED
VKLKCYMFCLLEVAGLADEDGTVDYDMLVSLIPEEYSERASKMIFACNHLDTPEKDKCQ
RSFDVHKCTYEKDPEFYFLF-
>MSexABP5
MCKPMYYVVFSIVYLSIVLAQKADNGNTKIANVQRNDQGSMDDVDVEDIMNQCNETFKI
EMAYLQALNESGSFPDETDKTPKCFLLCVLDNTGVMTKDGDFDPERTAALFAGERAGKV
MDGIQDMAAACADRKEKCKCEKSYNYLKCLMTMEIEKYANNN-
>MSexABP6
MKVTNGLFLVFLVSVLACGIAASPKKIYRIPPQASEKIVEEVLKCVQKMGLDSTVVNLLKE
GKYTEDDRVIETLMCSNQNVGNVNGDGKVNIDKVMNDIFSNKPEIRSALVACEKDGGKSP
LETFKNFILCFKEKVPVKVML-
>MSexABP7
MQACLFLTLVLAVVGLNAHNVHLTDGQKEKANEPIAACIKETGIKPEVIAEAKKGHYSED
EAMKKFILCFFHKAGIVNADGKLNLDVAIAKLPPGVDKTEATKTLEGCKDNGGKDAADP
AFAIFKCYKDATKTHVLF-
>MSexABP8
MKALLVLAACLVLAQALTDEQKEKLKKHKSECLSETKVEEQLVNKLKAGDYKAENDNL
KKYALCMMMKSELMTKEGKFKKDVALSKVPNPADKPMVEKLIDTCLANKGNTPHQTAW
NYVKCYHEKDPKHAIFL-
>MSexABPX
MISSLVHVLTLLAVGALALDEEQAELARMVRENCVHEIGVDEGLLAKVDDGADLMPDPK
LKCYLKCTMEMAGMISDGVVDVEAVLGLLPDDVKLRTTDIVRACDTQKGADDCDTAFLT
QTCWQQANRADYIFI-
>MS|comp141270_c0
MTMWFRALAMLVAGLAAAQAIEMDEDMAELARMVRESCAAETGADVALVEQVNAGA
DLMPDAKLACYMKCTMETAGMMSDGEVDIEAVLALLPPELAAHKAPSLRACGTVHGAD
HCDTAWKTQKCWQAANKADYFLI-
>MS|comp141612_c0
MKLFVVLCIVLVTEIYAAYVPLPSNHTDSNLEECRKTSEFTDETLNKMKTNPFVEDGGEIF
KKFIKCYLEKTGAITDDGKLNVDEAVPKLGPNFAKKIFEHCKTHVEKVGEEFVVVPTTTAS
DYSECFRQGVSNYIWNAKQEGFEPFTYEWQK-
>MS|comp153447_c0
MFGFGFLSLAAVLLCLGSTSALTPEEESSLKEALHPFVLECADEYGIPEEKFEEAKAKGSA
DDIDPCFISCFLKKAEFFDGDGKLDVEKTNEFVKAHLTSEHVIKFFEAVGGECAKVNDEEV
TDGDKGCDRAKLLFDCIQELKSKIGD-
>MS|comp108141_c0

MFKFTGLLYCAVAVSLSGVVRAGEEDAEAFREAIKPFITECAKEHGISWEDIAKAKETHTV
SSLKPCFVGCIFKKFEIINDKGEYDLEANLDKIKIFVKNEDLLTQLRDIMKKCVSVNDESVS
DGNAGCERAMLLANC-
>MS|comp116343_c0
MSKFTCLVLCVVAASISRAYASEEDKAAFRAAIQPIVDECSKEHGVSSDDIESAKTAGSAD
NIKPCFLGCVLKKAEILNAKGEYDSDKALTKLKKFVPDETKYAKYAEIGKKCESVNEKAV
SDGEAGCERGALLTACFLENRADIL-
>MS|comp129850_c0
MYKFTCFVFYILYAVFTQAESDSSDSSSDEVFEKLSHECMEKFGVTEDDLNGVIKTSDVTN
IDSCYWGCYFTKMGVLNDKGQFDMNNFQTTMKKLMKDDEDYDNLEKLVKKCEPVKDE
TVADGEAGCERGTLFAVCFVKNDGDFI-
>MS|comp162075_c0
MVLHRSATMSVRLALVVIASLLIAVECSQEIMKNLAINFAKPLEDCRKEMDLPDSVLTDFN
NFWKEGYEFTNRQTGCAILCLSSKLELLDPEMKLHHGRAQEFAQKHGADEAMAKQLVD
MLHSCMQTTPDDANDPCLKTLKVVTCFKTKIHELKWAPSMDLIVGEVLAEV-
>MS|comp163093_c1
MFHGGIWTKLQGFYKWYVLGLVSIGYILGELGHYLIGTTSKVTAEDLHYGDKSCMLNLS
HVSLSDLPVVCEKVNSSDICATLTLNGTRYCEWGYNGLGIDYQVLAGPAFMAVFTVVGVI
LGVVADKYNRARILSFCTLIFVIAILIMGSVKEYWHLVVLRMIMAAGESGCNPLATGILTDL
FPEKQRALALSIFNWGIYGGYGIAFPVGRYIPDVNAWGLSWRVCYYGAGIVGLVITLLTFL
TLREPSRTTIGEEGTGNKAGDSTLESGKKMPQVTIWHVIAQPRIILLCLAASIRHCGGMCFA
YNADLYYRDYFPDVDLGWWLFAVTVGIGSVGVVAGGIISDKYVEKMGVRSRVLVLALSQ
LIATLPAFGSVVFGPLWAMITLAISYFFAEMWFGIVFAILVEIVPLSVRSTTVGVFLFVMNNV
GGNLPILVDPVSKIIGYREAIMIFYAGFYGISSIMFFLTMFLMDGPVDKPAATPEEPKRTGLD
NRAFTQDELPTRNAGRSLPTAPSERL-
>MS|comp124490_c0
VRLALVVAAWLFIRVDASQDVIKNLSMNFAKPLEDCKKEMDLPDSVTTDFYNFWKEGYE
FTNRQTGCAILCL-
>MS|comp158322_c0
HELKWAPSMEVVMEEIMTAA-
>MS|comp114857_c0
MTPTTLVLALGLAAALADVNVMKDVTLGFGQALDKCRQESDLTEEKMEEFFHFWRDDF
KFEHRELGCA-
>MS|comp118070_c0
MIFRFLLSFYLIEFYGAQARTDQEIKAWFFQEGMECNKEHSISPKEILMLKENKIPDTENAK
CFVACIFKKTGMLDSKGMFDAAASVAMTEKDFADDPQKQENSKKLLESCKKVNYEAVTD
GEKGCERSVLLHKCFVETAPQLGIKLP-
>MS|comp121812_c0
MDAQKLGIQRELVTVGIQCIKDHPLSLSDIRAFRNKMIPNGNDPKCFVACLFKKIGVMDN
MGMISPAKAKENAKKVFKGSEEHLKNVDEI-
>MS|comp131182_c0
ENTFLQLPFALMQVSMDCFAGQRVMDASVMFECAVYDCKWENFDKANMRLVLVMLQN
AQKTMTLSAGGVKTLSFSALMSVFRGIYSAYTTLRTTMK-
>MS|comp133839_c0

VTLPPIQNEKADNYAYACIEESGVTTEALKESRKGNFFDDEGLKKFTHCFFQKADIVTCDG
QLNIDVALAKLPVGVDKVLAKEILEGCKTKTGKDSTDKVYEMFKCYFSQTPVHIKLSFDIE
KKTVSA-
>MS|comp133858_c0
QIHEDEALKCYMNCLFHEIDVVDDNGDVHLETLFNTVPGTVRNQLINMAKECEHPEGDT
LCHKAWWFHQCWKKADPVHYFLP-
>MS|comp141404_c0
MNTSNFQSIFCILCIVGLFFSYSHAMTRQQLKNSGKLMKKSCMPKNDVTEEEVGDIEKGK
FIENRNVMCYVACIYTMTQVVKNNKLSYEAVIKQVDIMFPAEMRDAVKAAATFCKDTTK
KYKDLCEASYWTAKCMYD-
>MS|comp141702_c0
MSKFSCLAFCVVVVSLNSVLAEDGPANEGDVLDIVFECAKENEVKASEILAVMTSRDVTL
VNPCLWSCCLKKGGFIDDKGQYVLNPGLQYVKNIVKSDQFYTFIEKSAKQCESVKDKAG
SECELGALLAACILEQMMKM-
>MS|comp142589_c0
MLKSSVFLCCLCFCALTPYLALAMTDEQKAQIHQHFETIGKACNTGSNVITADDITNLRAR
KIPSGPNAPCFLACMMKQIGIMDDSGMLQKETLLELAKSIFKEPEEMKLIEDYLHSCAPVN
NESVSDGAAGCERAMLAYKCMYENASQFGIEI-
>MS|comp143661_c0
KFTCLVLCVVAASLSQAYASEEEKAAFREAIKPIVEECSKEHGVSHDELKSAKDNQNADNI
KPCFLGCVYKKAEVFNSKGEYDVDKALEKLKKFVSNDEAYAKFAEVGKKCVSVNDKAV
SDGDAGCERGALLTACFLEHKAEVPL-
>MS|comp145270_c0
MWNFIVLCLALCSCTYALTEEELKMQMTKLVMKCNKDSQVDMMELVQLQSFVVPTKMA
TKCVLACAYKAADVMTADGLYNIEHAYKIAEQMKNGDEKRLENAKKMADLCVKVNEE
RS-
>MS|comp145826_c0
MSKFSGLVLCAVAATFISVASGESLSESLKPVIEKCSKEHGVTDADIQAAKAKGSSDGINPC
FLFCVFQNAGIFDAKGEYDAATGLKNLRQIVKDNDQYKNLEHAFNDCSKIKDKPVSDAA
GCEKGLHL-
>MS|comp148301_c0
MLLIEIVKFLILVAMCEAMTMKQIRNTGKMMRKSCQPKNNVEDEKIDPIAEGIFIDEKEVK
CYMACIMKMANTIKNGKLNFDAAIKQADLLLPDEVKEPAKEAILACKKAADGHKDICDA
SFHVTKCIYNQNPGIFYFP-
>MS|comp148423_c0
KEHIREALENGCAKCTDKQKEGTRRVIAHLIKHKNADWQKLKAKYDPEGKYTHKYEKE
LEEVQH-
>MS|comp150111_c0
MDRKGLCLLIVAMFLATGSDAMSRQQLKNSGKVLKKNCMNKNQVTEDQIGTIDKGNFV
EDKKVMCYIACIFEMTNVIKNGKLNYDASIRQIDLMYPPDLKEGAKAAVDKCKDVQKKY
KDICEASFYVAKCMYEFNPADFIFA-
>MS|comp150160_c0
MTKVLLTTVLIVITFALTRAASTQMKDAMPKEAMTTTTMANQDSSIDSTDIDVIAVMNAC
NESFRIEMSYIQAMNESGSFLDETDKTPKCFIRCVFTNVGIVSEDGKQFNPARAAFIFAGER

NGKTMDDIGDMTVACAADRQETCPCERSYQFLRCLMSMEIEKYEKS-
>MS|comp150353_c0
MFKSGIVYFVVVAVFLGNALGISDELRSQIQSKLLTVGAECIKEHPLSIDDLAAFKSRVFPE
GENAGCFSACIFNKLGLFDDKGTWAHVEAFENAKKVFENKEDLKNVQSFLTACAEVNEA
EVTDGEKGCDRAKLAYDCFIKNYEKLGFDFDF-
>MS|comp152336_c0
MYARVLIRFLEAKLCAAKILNLLQMNQSYLLLLIAACVEISYGMTRAQVKKTMTIIKNQC
MPKNSVTEDQVKNIEQGDFNEDPNIMCYVACVYKSLQVVKNDKLDVGLISKQIDALYPPE
LKEPTKKAVALCINSQDNYNDLCSRVFHGAKCLYEKDPACFIFP-
>MS|comp154792_c0
MMMMYSSLAMMLLTYLTIQSNATETSTYTTKYDGIDLDEILNNERLLIGYVNCLMDNGPC
TADGKELKKNIPDAIENDCKKCTDRQRDGSDRVMHYLIDHRPDDWVKLEEKYNSDGSYK
MKYLSSKKTEDSKETNVTKSDEETKNSSKE-
>MS|comp154997_c0
MADSRMRLVCLVCVIFAASSAMASKELLTKMSSGFTKVVDQCKNELNVGEHIMQDMYN
FWREEYALVNRDLGCMVMCMAAKLDLIGDDQKMHHGKAEEFAKSHGADDALAKQLVG
LIHECETTHAGVEDACSRTLEVAKCFRSGIHELDWQPKVEVIVTEVLTDM-
>MS|comp156324_c0
MTSKCGLLLAVMAAVAGSVMGTAEVMSHVTAHFGKALEECREESGLSAEILEEFQHFWR
EDFEVVHRELGCAIICMSNKFSLLQDDSRMHHVNMHDYVKSFPNGEILSGKLVELIHNCE
KKFDSMTDDCDRVVKVAACFKVDAKAAGIAPEVAMIEAVMEKY-
>MS|comp156380_c0
MMFFCSTVFFYPFLQMTREQVKSSGKMIKKTCSVKNNLSEDQVKDVDKGNFIEDKNFMC
YVACVYKMGQTIKGNTINHDMMLKQVEMMFPTEMKAPVKAAIEHCRPVAKKYKDVCE
AAYWTAKCTYEFDPANFMFP-
>MS|comp156923_c0
MFKWLIIVALVAASYGDPISESRDNKSATLKPLSVCCDIPELGDPKHLAKCSNPKLPGPCND
IQCVFEESGFLTDVNTLNKEAYKNHLKQWEENHAGWSVAVDKAIKDCVDSDPRQHLNYP
CKAYDVFTCTGIAMLKKCPAAAWKC-
>MS|comp158230_c0
MFGSKTVFYLLTVFSACFGAVDIKKYLKVCDRNAIDVNDCMADAVQKGIAVMIHGIPELG
VPPIDPYLQKEFRVEYKNNQILAKMILKNIYVEGLKEAKVHDARLRADDDKFHLEVDLTS
PMVAVKAQYYGEGQFNSLKIVAYGDFNTTMTDLVYTWKLSGVTEKNGTETYVRIKDFYM
RPDLASIVTEFRNENPESREFTDLGTRFANENWQTLYKEFLPYAQANWKRIGIKVANKLFL
KVPYDQLFPSSS-
>MS|comp158329_c0
MKTFLVLAACILLAQGLTDEQKEKLKKHNTECLTETKVDEALVNKLKTGDYKTESEPLKK
YALCMLMKSELMTKEGKFKKDVALAKVPNAADKPNVEKLIDSCLANKGNTPHQTAWNY
VKCYHEKDPKHAIFV-
>MS|comp158921_c0
MFKFCVFLAFCVAASYAAPGGGTYCGETPSVIYQCLNSPKVISAVPAKCAKYDDECEKLTC
VFRESKWLDGTAVDKAKVLAHLDQYERDHAEWGPAVQFAKTACLGPELKAQGVFLNCP
AYDVTHCILSSFIKHATPTQWSSSASCSYPHAYAAACPVCPSDCFSPQVPYGSCNACYLQP
RTP-

>MS|comp159796_c1
MYRLVILSIVAVSALADEMGMRECGRMFHPHSVRCCKKTSELKDKFMLSEDLKECFQMR
GNPVTCENEVCIAKKKGFATDDDKLDYTKLEEVMTKEIDDKDLLADMIKNCVNGDLEKY
GPPDFCEFMKMRHCISMQMLNHCPDWDDAGECSKLKGAVADCVKLFA-
>MS|comp160581_c0
MIRSCLVLAAVFQVLFGQESGPDPRDGFRQPVPHYCLSPPPGTDLHKCCPIPKLFPDGDME
RCGIEKASVDQSKSPPKPRIPCKESICLMQNANMLLANHSVDYEKLRTFVDIWADSNPEFT
EAILEAKKACAKDGGPSGPPVCEQDRIFYCLTSNVLWNCKLRDFEDCRVLKAHMDECRP
YYWKKREEDEANAQTS-
>MS|comp161014_c0
MSRHFNLLTDSSRMHHDNTEQFIQAFPNGEVLARQMVSLIHGCEKQFDHEEDHCWRILH
VAECFKQACVQHGVAPTMEMMMTEFIMEAEAR-
>MS|comp161605_c0
MRAGGLCGQYSQELYKMKSFVVFCLVLVVGVYANVTLPPTQQEKAQKLAAECVKESGV
STEVLAEAKKGHIVEDENLKKFTFCFFKKAGIVDSDGKLNVEVATAKLPPGVDKEDAKKV
LEGCKSKTGKDTADTVFEIFKCYHKGTKTHILLAGL-
>MS|comp393373_c0
MSRLKTAIAECTKVVESIFEVCEYASVFNDCMHIKMEHILDQVMMERRLEAIGKITTDPEQ
WGDTEDEILKLVKDEL-
>MS|comp427468_c0
MFVYRYMIAFHTLCVVYYVVGQHEKESKFGEIVKRTVIATAHTCMDHVNATVKDLENLR
DREEN-
>MS|comp502645_c0
MKSFVVFCVLVAGAFAANVSLPPKQNEKANQIATECMKESGLKPEVLAEAKKGHISDDEH
LKKFTFCFFKKAGIVSEDGKLNTEVALAKLPP-
>MS|comp60723_c0
MFTGTLPVVLCLVAAAYGGKEKPVFSDEIKEIIQTVHDECVGKTGVAEEDITNCENGIFKE
DTKLKCYMFCLMEEASLVDDDGTVDYDMLVSLIPDEYYERTTKMIFSCKHLDTPDKDKC
QRAFEVHRCSYGKDPDLYFLF-
>MS|comp672758_c0
LEKIGVVKSNKYSKLGFMTAVTPLVFTNRKKLDHMKTVSENCDREVNHKQETPCQLGNE
VTTCIFKYAPELHFKS-
>MS|comp71248_c0
KKMADLCVKVNEEKVSDGEKGCDRAALIFKCTVDNAPKFGFKL-
>MS|comp141177_c0
MIKFSVVCLYFVVVAVHFWNVKCMTKDQEQEIIKAMKPLAEECASYCGLKDEDLKKYQG
GDDMNPCFKKCMMQKLGLLDQEGKYDKATLHETMSQYGEDKEKAQKIEDQIDSCFMA
NADNNGDDEEAIKKRVDVMFNCIKELKE-
>MS|comp152359_c0
MSKFTCLVFFIVAASISKAYASEEEKAAFREAVKPIIEECSKEHGVSIDELKAAKAAASADGI
DNCFLGCVFKKAEVINAKGEFDLDNALTKLKGFVSNEDHFAKFEDIGKKCASVNEKPVSD
GDAGCERAAMLTACFLEHKGEMPLNF-
>MS|comp161583_c1
MNIKTSFIDYPAHNIPEVIRVGQLFQVATYLADGHKDICDASFHVTKCIYNQNPGIFYFP-

>MS|comp167844_c0
MSKFTCLILCVVAASLSSFYVRAHTPTFRDSLQPLIEACSEEYPGVTSDMVNQAAKEGNPN
IIHPCFNGCVFKKAGFINEKGEYDTNSALTNLRKLVTHEEQYRKLAEIARQCTSVKDTVTD
GEAGCERGARISACFLQQKDNVSL-
>MS|comp173090_c0
MSKFTCIVFCIVAASLTKVSHAVTEEEKAAFREVMAPIIDECSGEHGVSKADIQAAKEAGS
ADGIKPCFLGCVMKKTETLDDKGLFDAETALSKLRTFVKSDEDFAKFEEIGKACMSVNEK
SVSDGEAGCERAKLVLACFLEHKADIPF-
>MS|comp152974_c0
MFNVYFCVFVCGVLSLNIKASSLDDLKLKYVEVIIECSNDYPITVADMTELRKKIMPDSEPI
KCLFACVYKKTGMMNEKGELSVDGVNEMSRKYLADDPAKIKKSEEFTEACKSVNDVAV
NDGERGCERAALIFKCTVEKAPDFDLI-
>PxylOBP19
MYNSCVLFLLLCVSLTHGNLFLEELRKKGASLKPLSACRDIPDLGDPEHLAACSSPKLQGP
CNDIQCVFEKSGFLVDKQTLDKEAYRTHLRRWAEEHQGWSEAAEKAVRDCVDRELRQYL
DHPCRAYDAFTCTGIAMLKKCPEEAWKCSHKK-
>PxylOBP9
MFPYIFTYVFIGVYFTCTTGTLMKDEERVKFHKTIQSCAQQHPISPEDQGSLVNWRISDTQE
VKCFIACIFQGIGMIDEKGRFDAAHVNDITKLMMTEDDPDVLQQTQDITESCKYVNDRHV
GDPHETCERAASLFRCAAKWTGKKDS-
>PxylOBP3
MFLASLATLVLITFTLSSGKMKAEEPRTREMPDMSSSSSVSFHDGNDEHAEDDMMGIMTH
CNETFRFDPAYWQSLNESGTFPDENDKNPKCFIRCVLEGTRVASLDSVFDAARAAEVFAGE
RGGRPMDDLETLAKNCADDRRETCKCERAYGFMKCLMEAEIETYEVYKADDS-
>PxylOBP32
MKFLVVFAICLVAAQALTDEQKEKLKKHKTECLAETKPEVEHVDKLKNGDYTTENEALK
KYAHCMMIKSELMTKDGKFRKDVALAKVPNPADKPMVEKLIDTCLANKGDTPQQTAWN
YVKCYHEKDPKHAIFLKLG-
>PxylOBP23
MFRAHSDQCLASSGATAADVDSLLSGALVDTPALRRHVYCILLRCKAIGKDGKLQKAAVL
GRLARIDDKNATKVLENCFEQTGETPEDLAWNLFKCGYDKKSVIFEYMPNASPSSGEN-
>PxylOBP13
MTDEKAKLQFTKIAMNCTKKYPLEVHEILDLQKLKVPTKKTAKCLLACAYRLEGSMNEK
GLLDYEHMMKTADLLADGDEKRLKNAKAVADICIKVNDETVNDGEADCDRAALLFKCA
TETAPKYGFKV-
>PxylOBP12
MKDEERLKFHKTIQTCVQRHPISPEDQGSLVNWQISDTQEVKCFIACIFQGIGMIDEKGRF
DAAHVNDITKLMMTEDDPDVLQQTQDITESCKYVNDRHAGDP-
>PxylOBP6
MTRAQLKKTLSMPKKQCMTKHGVTEDMVGGIEGGKFVEDRKVMCYIACIYQTIQVMKN
DRLNKDLVIKQVDLLYPPELKAPTKAAVKQCMPVQDKYPDMCEGVFWSVKCLYEQDPAS
FVFP-
>PxylOBP31
MEGHKVLTPLLFGVLVVSVSAMDEEMAELAKMIRDNCGAETEVDMGLIDQVNGGADLM

PDGKLKCYIKCVMETAGMMSEGEVDVEAVLALLPEDFKAKNEKSLRACGTQKGADDCD
TAFQTQACWQKANKADYFLV-
>PxylGOBP71-like2
MRVLVLCSFALLDLLFHANALTCRSDGGPKEEELKIIYMNCLKSQDGKNSSNSRDYSDDY
SSREKRKHSKGSNFGRRENAFGGREESYGSRDEWSDRDWTRSRDQTQSRGDWSSRDDK
VSSGSTQYGRSESGSSRDSMNNMRTSTNNMRNNMNSRDSRMGGGRDDYADNDYESYTH
GYNNRQQQSRRMKRERSSGHRSQYNPNTSRSGSGSGSGSGHDDSYRNNDKNSSENNSSR
DSDHKACALHCFLEELEMTGDNGMPDRYLVTQVITKDVKNEDLRDFLQESIEECFQILYN
ENMQDKCDFSKNLMLCLSEKGRANCDDWKDDIKI-
>PxylGOBP56d-like2
MKTIVFVCLVATAAAAANVKHAHLTKDQSARVHEYGMECMKKTGVNPELVAKAKKGEF
TDDEALKKFTLCFFQKTGIITSDGKLNEEVALSKLPAEVDKAAVKKVLDECKKKTGKDMA
DSAFEVFKCYHKATPTHVSF-
>PxylGOBP28a-like
MANYIRAICFLTFVLFSKGSSALTNAQNSAVLSTLLSGAIQCFGEHPLSAAEMDAIKNKKTP
ETENAKCFAACVFKRVGIMDNKGNISGTQAVQNAKKVFDKDSVKKLEDFIGECTKVNNQ
HVNDGAKGCDRAKLAFPCFVNNAQKYGVSLNF-
>PxylGOBP56a-like
MKMGSSMYVAFLAVAFVAIFGNIHAISDENREKLKKEMGPIFMECAKEGALNLDDLKQYK
GVKELPADEGVTCFFACAFKKIGMIDDKGMFAVEESVERGKKYMDSEEKQKHLEEAANT
CASVNDESVSDGDKGCERAKHLYECLIKQAEKFGLELPTSAV-
>PxylGOBP72-like4
MFQNILKLVVLELLLFYGVADAMTRQQLKNSGKMMKKSCMPKNDVTEEQVGDIEKGHFI
EERNVMCYVACIYSMSQLVKNNKLNYESVIKQVDMMFPPELREPTKAAAEKCKDVGECP
TPPLESQKPYYKTKTKDNSGLNPVLDY-
>PxylGOBPlush-like
MFLKNHWLIVMVVATGICVVSSITRKQMKNTSKIMKKSCMPKNNVTEDQVGNIEQGEFL
EERKVMCYIKCIYAMGGAIKNDKFVYDAMIKHVNLVFPPEIKEPTLAAINQCRDVDKQYA
DSCEAAYWVAKCMYEYGPEQFFFP-
>PxylGOBP72-like3
MERREFGLLIIAVILTSGAEAMTRAQLKNSAKMMKKSCLGKVDVPEENIVDIEKGKFIEEK
NVMCYIACIYQMSQIVKNNKLSYEASIKQVDLMYPSDIKDAVKKSIEQCKDVSKKYKDLC
EASYWTAKCIYDHDPKIFIFA-
>PxylGOBP72-like2
MVLLVLIAKYVLFVALCDAMTMKQLKNTGKMMRKSCQPKVNAEDAQIDGLKDGVFLEE
KETMCYIACIMKMANAIKNGKLNYEAAMKQADLLLPEEIKEPAKAAITSCRKVADQYKDI
CEACFYSTKCIYTQNPDIFFFP-
>PxylGOBP72-like1
MARSLPVLHPEHSCFNGHLAVMTMKQLKNTGKMMRKSCQPKVNAEDAQIDGLKDGVFL
EEKETMCYIACIMKMANAIKNGKLNYEAAMKQADLLLPEEIKEPAKAAITSCRKVADQY
KDICEACFYSTKCIYTQNPDIFFFP-
>PxylGOBP70
MVCRLSALACCFCMFAISLCGSSIPSESDTRCKNPPTAPQKIERVITLCQDEIKLSILREALD
VIKEEHTMPAQRRRNKREVPFSHDEKRIAGCLLQCVYRKVKAVDGYGFPTLEGLVGLYSD

GVNERGYFMAVLEASRGCLMRHHDRFSRTVPMDNGRNCDVSFDIFECISDRIGEYCGNSG
L-
>PxylGOBP56d-like1
MIAGCHAHLTKDQSARVHEYGMECMKKTGVNAELVAKAKKGEFTDDEALKKFTLCFFQ
KTGILTSDGKLNEEVALSKLPAEVDKAAVKKVLDECKKKTGKDMADSAFEVFKCYHKAT
PTHVSF-
>PxylGOBP83a-like
MLIAAVFGGKSKPEFSEEIKEIIQHVHNECVGKTGVAEDDITNCENGVFKDDQKLKCYMF
CLLEEASVADENGVVDYEMMISLIPEDYTERVSKMIMACKHLDTPDKDKCQRAFDVHKC
SYEKDPDLYFLF-
>PxylGOBP71-like1
MIYMNCLKSQDGKNSSNSRDYSDDYSSREKRKHSKGSNFGRRENAFGGREESYGSRDEW
SDRDWTRSRDQTQSRGDWSSRDDKVSSGSTQYGRSESGSSRDSMNNMRTTTNNMRNNM
NSRDSRMGGGREDYADNDYESDTHGYNHRQQQSRRMKRERSSGHRSQYNPNTSRSGSG
SGSGSGHEDSYRNNDKNSSENNSSRDSDHKACALHCFLEELEMTGDNGMPDRYLVTQVI
TKDVKNEDLRDFLQESIEECFQILYNENMQDKCDFSKNLMLCLSEKGRANCDDWKDDIKI
-
>PxylGOBP1-like2
MWFPVRLSAAVLLTLAAMDAEGSKETMKDITSGFFKVLNECKHELNLPDHLVGDFYHY
WRQEYALLDRDLGCAILCMSRKLELIDASGKLHHGNTQEFAEKHGADNSMASKLVEVLH
SCEKQHEAVSDDCQRALEVAKCFRSSVHELGWAPTIDVIIEEVLTDM-
>PxylGOBP1-like1
MADSTVSRWRRFALCALLVAIQVKKSESSADIMQKLTIGFTEALEHCKKDLQISNEVMQD
FYNFWREDYALVNREMGCVLMCMAARLDLVTEDLKMHHGNAHEFAKKHGADDTMAK
QLVSIIHECEQGAASVPDECARTLEMAKCFKTRIHELKWAPSMEVVLEEVITELKN-
>PxylOBPPA10-3
MKFLLILSVVVAAALARPEDLYDDSNIQLDVDELISNERLMKAYAACFLSKGPCTTEGSKV
KQLLPEAVENICGKCTAKQRGMVRKMVVAMRERLPSEWEQLVATYDPEGKYQPKFEDFL
ALNE-
>PxylOBPPA10-2
WVARPGDLYDDSKSNLDIDELVSNERLLKGYAHCFLETGPCTPEGNNIKKIIPEALENVCK
KCTPKQRVMVRKMIAAFKEKLPAEWSELAKTYDSEGKYKENVKTFLAQSD-
>PxylOBPPA10-1
MKVAIIVSMLVVAACAQEKYKSDLEGDFDVTELLNNERLLLSYTRCLIDKGPCTPEVKAV
KDKLPEALATKCAKCTDKQKELGKKLAVELKRTHPAVWAQLVAKYDPQGQHQAAFQEFL
TKQ-
>PxylOBP-2
CGLMVAALPAARGTAEVMSHVTAHFGKTLEECREESGLSGEIMEEFHHFWREDFEVVHR
ELGCAIICMSNKFQLMQDDARMHHENMHDYIKSFPKGDLLSETMVRLIHNCEKKYDDID
DERSRVVKTAAC-
>PxylOBP-1
VTLGFGEALEHCREQSQLTGEMMEEFYHFWREDFKFEARAVGCAIHCMSRYFNLLGGQQ
RMHHDNTHKFIQSFPNGEVLSHQMVGIIHTCEQQHDAETDDCWRILRVAECFKRESQAQG
LAPSMEMLMA-

>PxylGOBP2
MASVLSLVVCGLMMAALPAVRGTAEVMSHVTAHFGKTLEECREESGLSGEIMEEFHHFW
REDFEVVHRELGCAIICMSNKFQLMQDDARMHHENMHDYIKSFPKGDLLSETMVRLIHN
CEKKYDDIDDECSRVVKTAACFKKDAQAEGIAPELTMIEAVLKKY-
>PxylGOBP1-2
MERRWCLLVLAAAAAGLPGVVRGTVEVMKDVTLGFGEALEQCREQSQLTEEMMEEFYH
FWREDFKFEARAVGCAIHCMSRYFNLLGEQQRMHHDNTHKFIQSFPNGEVLSHQMVGIIH
TCEQQHDAETDHCWRILRVAECFKRESQAQGLAPSMEMLMAEFIMEADV-
>PxylOBP36
MASVWSLVVCGLMMAALPAARGTAEVMSHVTAHFGKTLEECREESGLSGEIMEEFHHF
WREDFEVVHRELGCAIICMSNKFQLMQDDARMHHENMHDYIKSFPKGDLLSETMVRLIH
NCEKKYDDIDDECSRVVKTAACFKKDAQAEGIAPELTMIEAVLEKY-
>PxylOBP24
MIPATVLLAVLPSVLGLSPPLLFESLKSEPTISTLYTTDKKADKITNECLVEMYPRNLYNYPL
NIDKNDVPCILHCVLKKFGIMSNDGKIIIKNYYRRVQQIHRYDPRILISDVGDTCAQKIKSM
DLSRDVCKKAKVFNECTQLFAVSLKDSGR-
>PxylOBP18
MNLPQVSPFQPFSKEEQLTRIKKMNEEVEPFRKNLTECAHQVKAKATDVEDFLKRIPQSTA
QGKCFVACILKRNQLIKNNKIQKESLIEVNRAVYGDDSEVMARLKTAIIECEEVVFDIFEICE
YASMFNDCMHMKMEHILDKVTMDRRMKALGQKASADAYTDDCTEHDDEILQLLKDEL-
>PxylOBP35
MERRLRLVRVLVAASLPMLVLSSVEVMKDVTLGFGEALKHCREQSQLTEEMMEEFFHFW
RDDFKFEQREVGCAIHCMSHYFNLLDDTHRMHHQNTHKFIKSFPNGEVLSQQMVGIIHTC
EQAHDKEPDNCWRILRIAECFKKECQAQGIAPTMELLMAEFIMETDV-
>PxylOBP28
MSECLKEVQASDTRTVKRLSPSIHSPIHGECLIACVLKRNGVIENGKVNKGNLIALVNKFY
AKNTKVMKKLDKNLDRCIGASVQVRDECSMAELLNQCTNELMANNKHKLTVDY-
>TcasOBP01
MKTVAVLLFLALAACTKQEDDDRQETIRQYRDDCIAETKVDPALIDRADNGDFTDDAKL
QCFSKCFYQKAGFVSETGDLLFDVIKDKIPKEANREKALAIIDKCKELKGADSCETVYLVH
KCYFLHSYGTDKKTE-
>TcasOBP02
MKAFIVLVAVAVCAQALTDEQKEKIKNYHKECSAVSGVSQDVITKARKGEFIEDPKFKEHL
FCFSKKAGFQNEAGDFQEEVIRKKLNAELNDLDATNKLIAKCAVKKDSPQQTAFETIKCY
YENTPTHVSLA-
>TcasOBP03
MKFLVVISTVLMANIVQGLTDEQKSKLEEYSKECLKESKVDESVLKEAEKGVYLDDPKL
MNHVYCLVKKINSQKDKGELEVTQIKEKLMMQINDEKEVDKLIQLCLVQEKSARYSLGK
CEVSS-
>TcasOBP04
MRASAVFLSSFIISIQAAAFNNPEDELRRSAACLEQSKVSSESIKNLQIGNFDDDERLKEYLF
CVSKNAGYQDPAGHLQHEMIRLRFKGGRYSDDTINEVLQQCGHQKDTPQETAFQFMKCA
YQNAFPRNYK-
>TcasOBP05

MKTIICFVFVLAGAWFCFQALTKEQIDKLEPISKECRELNGISEDTILKVRRGEAVNEPKLK
NHVLCVSKKTGLASETGETNVEVLRTKLRKVSENDDEVNSIIQKCVVKKSTPEETAFEIFV
CLRKVKPNFSPAN-
>TcasOBP06
MNFVCVIFILVAIIGAHGLSEQQTEKLNQLSKECRALTGVSQETITNARNGNFEEDPKLKLQ
VLCIGKKVGIMNESSQIDENVLKAKLRKVSDNDEEVNKIYNKCAVKKPAPEETAFETIKCV
MKNKPKFSPVE-
>TcasOBP07
MKFLVCLLFVIVAANALTKEQKEKLDKISKECKNQSGVSQELIDKARTGELINDPKLKAQI
YCVSKKAGLATEAGEINMDNLKTKLKKVAANDDEVNKIIQKCVVKKPTPEETAFEVYKC
LHANKPNFSVVD-
>TcasOBP08
MENRLVLLIVINTLLLAQAAAKQDFHKKCLASSGANADTIAKVRNGKFSNDPQTQKYFG
CMLRSVGVVNQAGQLQVAALRKQVPKDMKRDEAMKIYMSCKDKKGANNDETAYLLYK
CFWEASPRHVKIDGQ-
>TcasOBP09
MKYLLFLTVITLTCGIFAFSLSNREQAIFLSTYSTCLETSKVDSERALRTASGIIDDEPKLKEF
LFCINKQNGVQDDAGNFVKDAVRKRIEHPLLTDKTMEIIVNKCTRKRETGEETAYQFLKCS
YFTIMNEKHQ-
>TcasOBP10
MNIYTCLVLVVIAASAQHLTEEQKNNWRKWSNECKVLIGVSQEAINKIRNNEFDSVDDKI
KKHGLCFAKKASLADSSGNIIINQIKIKLKRVIEDDEEVDRIVTKCTIRKNTPEETTFETFRC
LRENSSKFVPV-
>TcasOBP11
MKQIYCLITVVVLIPTLTALENEGQNPDTANCVALGGQRIKDSEIAKMAHCILTKTNLMTD
KGTFNSNLLKERLRQSVHSDELVDKVVMMCTVEKETPLKSAFSGYKCLRYLVPWFPLD-
>TcasOBP12
MKIVLCLLALATVALAKKCFLAEDTDKLEVMINECKTKTGVPDDILQKARNGEKIDDPKL
REHALCMMKKSEMMNDAGEMQMDKIRARIKHAVSNEAEGTRIMNECAVKKDTPLATAY
EMICCLIRNKNSVDE-
>TcasOBP13
MKVFVCLAVFALVAAAQAETAKEKLRKYSDECKSVSGVSEELLNKVRNHEDVHDPKLDE
HGFCILKKAGFMNEAGDILADTIKTKLKENSEHPDTVDALVEKCNEKKDTPQHTASHLFT
CLVDKKVHSH-
>TcasOBP14
MKLFILLSLLSVCYARKWFDKDPQDVAKWQKECFEASGVSMESMNKLPNITLSEDPKLGE
NAFCLLKKLGFISEDGTLLIEKLRTSLKNQWGDEIANKLVNECARQKSTPQETAHEMFLCI
PAKLK-
>TcasOBP15
MKIVLICVLIGLVVAKQQKQDTLDEEKEKMKKWTQECIQESGVTSEILQQLRNQKRVEDP
KLKEYTFCTFKKNGFMNEDGKLQYDVIKSTLMKVSGSEEEANKVVKDCVVEKSTPQDTA
FETVDCWYRYKKN-
>TcasOBP16
MIRYYIVLLLYFFAPPGISEEMQELVNQLHSTCVAETGVSEDLINKVNSDKVMIDDEKLKC

YIKCLLTETGCISDDGVVDVEATIALLPEDMKAKTTPVIRSCGAKMGANPCESAWLTHKC
YLETSPADYVLI-
>TcasOBP17
MSPLLLIFISCLFPRGISEEMQELANTLHATCVDETGVSEDAIESARKGNFAPDDKLKCYM
KCIMEQMACIDDEGIIDVEATIAVLPEEYQAKAEPIVRKCGTKIGANACDNAFLTNKCWYE
EDPEVSLQLN-
>TcasOBP18
MYKTRVIYVLFALCLVEIFVSRAIEMDDDMKELINNLHNTCTGETGATDDQIENARKGNF
AEDDSFKCYFKCVFDQMGCMTDDGKVDSEAVIAVMPPELADKIASTVRGCTEVGANPCE
TAWLANKCYQKSNPDVSKVSSNVRSD-
>TcasOBP19
MSRMLPAALFVVLATLTFATAEIVVPDDLKDYINELHDHCLKEMGLTEGDHKNYNIHVKD
PKMMCYMKCLMTTSKWMNMDESIQYDFILSSVHPAVKNILLPALDKCRDIPKGTMECEK
AYNFNMCLFNADPENWFFI-
>TcasOBP20
MMPLKNLIILIVCPLFVDIPPDLQAEIDGYYDICYKQIGLTKDDLKAYKIGDRDPKIMCFMK
CVFVEAKWMDENENLQYDYIKNTIHHSIRHITLPELENCGKKAEGDKCEKSFSFFNCMNK
AEPEDWVLIQ-
>TcasOBP21
MMHLKNFVVLVVCPLFVEIPPDLEAEIDEYFEQCFEPNGVTMDDIKAYKMGDKDPKIMCF
MRCLFVSGKWMDENENMQYDYIKETIHHAIRHITIPELENCGKEAQTGDKCEKSFNFFMC
MNRAEPEV-
>TcasOBP22
MKMCVIFTLLLLVVLASAEEDNVGKIESVEKKCQEKTGVSEESLQKIMRLEEVDDPLVKE
NALCTLKAYGVMDDDGNIFPDKFEEKLKPEIGADEAKRVAEKCAVKKDSPEETAHQTLW
CATEENALTDTSQEQ-
>TcasOBP23
MKISTLVAILVLAGSAVCADEDNLNTENVQSIEEDCQKETGVSDESLQELSETGDSDDPLV
KKNALCILKAYGVIDDQGEISEDKLEEKLEPDRGKEEAEKVAKSCAVKKDSPEETAHEALL
CMQQKSQK-
>TcasOBP24
MKSFVIFVLIIVITGQINATPSLDDFKKVQKDCQKKTGVSDESINKVNNLEPVCDDLLLQE
NALCILKTYEVMDEEGKICPDKLMEVLEPKFGKEKAEKLIEKCTLEKDTPQLLAHATLFCL
SVQKYVV-
>TcasOBP25
MNSVLFLLVCALLDKEFLMQFLQKIKKVSEDCIAETQATKNDIKTLLEHKIPDSHEGKCMI
FCFHKHFQIQNEDGSLNKVAAISLLEPIKDHSQDIYDKVVKIFNTCFDSAERDDDSCIYASN
LAECAIRESKSVSVQ-
>TcasOBP26
MKLFILAGILFTGVCAVDQEFVEKFLQKMEKIGEECAEETHATSDDIADLIEQRDPKTHEG
KCLIFCYHKKFNTMKEDGSLDKVGSVLALEEVRDADFELYKNILTIFVTCGDKAKIYDDP
CETATALTMCGRDEAKAVSWAYFA-
>TcasOBP27
MKSTWFFLLLACSLTCAFLEKMQEFGAQCAEETDATSDDIAELIARKLPPSTHEGKCMIFC

MQKKFNMMKENGGIDRAGAIAALKPLQKADPELHQKVLKIFVTCGMRVKPSPDPCDTAT
ELALCGKKEAEAVFCRLGWKTLS-
>TcasOBP28
MKYFVVFASLFLATNALSQDFIDKFVAKVKSIGETCVPETNASKDDISSLLAHKMPDSHEG
KCLIFCFHKQFQIQNDDGSINREGAIKALEPLKADDAELYEKVISIFKKCESTPVDGDSCLY
AASLAECAVKEGRAVSFWKNTNFKLI-
>TcasOBP29
MKFLLVFLSVAILCTFAMDESFLQQTRDRVKAIVKECVTEEKATDSDFDDIMALKIPTSHE
GKCVFFCSHKKFNMQHPDGSINKEGALDTFEVVKDVDAEFHDKVITVYNHCLSTPVDPD
PCVYSVNLFQCFMKEAKAVRK-
>TcasOBP30
MKLLITLATLVVATYAIDKEFVQELRQKLRSHVEACAKEVNAGPDDVSAIFAHKLPATHEG
KCIFFCMHKLYNAQNEDGSLNMAGALANLELIKDMDPDVYTKVSTSFKNCESAPFDSDP
CLYAANLVTCIVKEGRAVSNNFSGFFF-
>TcasOBP31
MQLLVVVLAVCVLGANAGVSIILDPKFLEKLTQEVQAVGTSCGEKEHATADDMIEIMEEKF
PPTSHEAKCVVACFYKHYKMMKEDGTFDKDAAVKAFDEIKAQDAEIHAKILKVIDACDA
KKQMSDDHCVSAASMAGCVKTEAIAV-
>TcasOBP32
MLILIAVNCHKCIIVSALSLSATVFGQSLSEDEMRENARKLMTSCKDKVGASDADVEALK
MHQMPESREGFCMLECVFDSAKIMQDGKFSKSGMIEGFKPLIGDDKAKLESLEKLSATCE
SELGDGEDKCETAKRLVECVIKNGKTHGFEVPPPRE-
>TcasOBP33
MNFFAICLCFVASTVGVSSEENDINEIRSVEENCQKQTGVSVEKVNNFELVDDPLVKENAL
CILKAYGIMDEDGNIYEDKLKEQITSELGEKNAEQVAKKCTIKKESPQETAHESLWCVGEQ
KPIPGASPDEKN-
>TcasOBP34
MSKTVFIFVIFFYLDFYATGDESVYLSNHEACVKLSGVDETLLETIYEGDVFEDMKFKTYI
HCFFKKSGFQDENGVMHFDAIKSSFHKDFSQTENIDKTITECEEKKLNGESALETAFLHFK
CFMGEL-
>TcasOBP35
MKAILLLLLVATLSFYHMSEAQMKAALKLVRNVCQPKTKATNEQIEAMHTGNWDLDKNG
KCYMWCILNMYKLIGKDNSFDWEAGIATLKAQAPESVRDPAIASVNNCKDAVKTTSDKC
EAAYEIAHCMYLDNPEKYFLP-
>TcasOBP36
MKYFPHLCLCLIFFELSEAAMSEAQLKAAVKLVRNMCQPKSKATNEDIEKMHHGDWNID
RTAMCYMHCALNSNKLITKENVFNRDYAITLAEKNLPTALKTASIEAANLCKDSAKTLDD
KCVAAYEISKCLYESNPEKYFLP-
>TcasOBP37
MILKASIFLILAVATFGAILEDSELMKVVENCVKKTNANESEFSSPNFLETTPSQPALCTAKC
LLESLEIVNSEGNINMETLKEYAQPFESPAREAVATCGEEIKSVTTCDDMEKYRKCVEPLIK
NS-
>TcasOBP38
MAKKQLVLFFLAFIFLQKFAEVREECLSENSMTMDELHEGWKMENLPESHLCFLKCLLEK

REVIDENGVPQKEKIDEILTVKQLSDEKREEISTCITNVEKIENCETMSEIMRCFPKKRRD-
>TcasOBP39
MAKMCRLFVVLSLFVASQALDLMADKNFVELRNKCLDKLGLKEEDLRDLKFDGDVSED
LMCFGKCIQEEDGLLDSEGNLNEEKLEKKIETMPFLSRVSDDTKNNIMECLKEIGKIETCQ
DFGKQRDCIHKYV-
>TcasOBP40
MNPITSVILTFLFVFSFGEKESEEAQIFTELDGPAAELRDQCLEKNSMKVTDLKTYNTSNDI
PEKELCFYKCFYEGVEFIDANGNLNVNNMKEIPAISELGDEVLNEITACVEKIGKIRCCGDL
RKIEQCYQNITM-
>TcasOBP41
MWSFVTLLFSFLVLASAQGKYWTTISECLTEHSMGVEDMKKFDLPAEKMSEEMLCFNKC
FYDKLLITDENGEINTDNLMSIPLVNAIDASKHDDLVTCLKKVGKIEECDGVKKIEQCFVE
FI-
>TcasOBP42
MATRFCFGLLILFVGTVLVHEILEVRALCMNETGVSEETARNYKPAEDPASEEILCMVKCIF
EKIGCLKDDGSFCVDTMKKKNYIMDVINEENEEKIYECLRGVGKITNCRDMAAVEECFVK
NDSK-
>TcasOBP43
MSFLILLICVIPAIFCRSFSHDELDTDLSFIKTCNRTSPISMSKFGLFLTEFNLTEPGTMNECFL
HCLFMKYGWMDSDGGFLLHDIKQTLEESDVEIASLEFILYKCTATESNNRCERAFVFTQCF
WDKMAEQQPSEDQFFYNIEDKK-
>TcasOBP44
MKKTILLCLLSQLLLLKAAELQPEDRHQIALQCIDIVGIDQKVVEDAINIEIPKNNPKYKEF
LACSYKKQGYQNENGEILMENIKKFLQKFYHPSDLQELNSCSGHNGTNHAENAYQALQC
IYNRLSNMTVVGN-
>TcasOBP45
MKPIFAIITLTLCTTVHSAIRPWRTCGTWPPCPPNGKMLQNFRIKRASVRLTNTETNETTPE
PKAVSSEAQATENCIIQCIFDNLQMTDSTGYPVHTKILDGLLKNTTNRELRDFLQDTTDECF
QVMDKEDTMDPCSYSNKLVTCLAEKGRSNCADWPVGELPFKP-
>TcasOBP46
MLWRRGRFLYGENLDMFDPAGLQACMKKLSVGETELAKALEDKSKDPPEKIMCLFKCAL
EDSGFLQDGVVDKSKWPMPECVQDVVKITNCNDMVALKHCFD-

4.ORs

>BmorOR1
MLLSFKDDSRSPDIQKPQNFQYMKILRFNLKIICAWPEKQLNEIRSLGHSIHRVILPIQSVVC
LACGILYIHFHFNEIPFFILASTFITVMMNLVTCSRTALVMLFERYLVLTGRFITVMHLFNFQ
KNSDYAYKLCTFVNRMSHFYTLYVLFSMFMGLGLFNLLPLYNNYVSGAFSDPYGPNVTFF
HSVYFAFPFDYSHNFRGYIIMALFNSYVSVTCSIGLVMFDLLMCLMVMHVWGHLKILSHN
LINFPRPKASHVITTPNGPTNVETYTEEESKEVFARLRECIKHYGTVDDFANDMSETFGVIL
LVYYGFHQVSLCMLLLECSDLSTKAMLRYGPLTLIMIQQLIQISIIFELLGSVADRIPDAVYQ
LPWECMDVKNRRVVYGFLRRTQNPVRFKAMGMLDVGVQTMASILKTSISYFVMLRTVAT

-
>BmorOR2ORco
MMTKVKTQGLVTDLMPCIRLLQAAGHFLFNYHADTSGMNMLLRKIYSSAHAVLIVVHYI
CMGINMAQYKDEVNELTANTITVLFFAHSIIKLAFFAFNSKSFYRTLAVWNQSNSHPLFTES
DARYHQISLSKMRRLLYFICGMTVFSVISWVTLTFFGESVRMIASKETNETLTEPAPRLPLK
AWYPFKTMSGGGYVFAFIYQIYFLLFSMALANLLDVIFCSWLIFACEQLQHLKAIMKPLME
LSAALDTYRPNTAELFRVSSTDKTEKVPDAVDMDIRGIYSTQQDFGMTLRGAGGKLQNFN
AENNPNGLTAKQEMLARSAIKYWVERHKHVVRLVASIGDTYGTALLFHMLVSTITLTLLA
YQATKINGINVYAFSTIGYLVYTLGQVFHFCIFGNRLIEESSSVMEAAYSCQWYDGSEEAK
TFVQIVCQQCQKAMTISGAKFFNVSLDLFASVLGAVVTYFMVLIQLK-
>BmorOR3
MIFVDDAVIGIKDPREYRHLRVLRTSLRLLGAWPGHYLGEETGSKYECAPMFLLMFIKIAC
LYLTIVYLRNNADVLGFFELGHVYLTIFMTFVTLSRGFSLTWNPNYHKVVKKFITEMHLLY
FKDNSEYAMKTHRRVHKISHFYTVFLKVQMIAGLTLFNVIPMYNNYRQGNYASDRPANIT
YDLSIYYETFDILNTPNGYIFICVFNWFASYICCSFFCSFDLILSLMISTVSGHFRILIHNLLTF
PLPEAITASKKFVDKHRCNGNRSEFVLEEAKLYSPAEMWQVTDRLRQCIDYHRKLVEFTG
DISEAFGPMLFVYYLFHQVSGCLLLLECSQLNTAALVRYGVLTVVLYQQLIQLSVIVESVG
TVTGRLKDAVYEVPWEYMDTSNRKTVAIFLMNVQEPLHVNALGLAKVGVQSMAAILKT
SFSYFTFLRTVSE-
>BmorOR4
MFKIIKNIIVENDALKQVEKPQEFQYMKWVQYHLKYIDGWPNMDMNKKNVSKIRFHKR
HLLVVEQTITFLSQMFYIVKNYGKLSFFEIGHSYITALMTIVIFSRSVVTALGRYRKIARYFV
SSLHLYHYKDISEYALQTHLLVHRLSHYYTVYLISLVVTGMLLFNITPLYNNISSGVFNSPR
PENMTFQHAVYLGLPFDYTTDIKGYFVVFILNWHLSHIAASYFCTFDLFLSLLILHLWGHL
RIILNNLKTFPKPYTNNSMYTEEENQVVLLKLQECIRYHNFIISFTVMMSNVYDVVIIVYYL
FHQVTGCLLLLQCSTLDWESLSRYGPLTLIIFQQLIQVSMIFEILGFLSDKLPNAVYSIPWEA
MNVTNRKLVQVLLQKSQKPIQFKAMNMMSVGVQTMASIIKTSISYFIMLRTIARD-
>BmorOR5
MLLYYPNTQVKEKVNNVEEFTYIKFLKSFCKIMDFWPEREEKNSKTRIFRLRYILVLQFCF
TLVAGVLYLTNSVGKQTFYDLGHTIITVLMNVVSLSRLILRCFKKYDVVGQQFINKIHLYH
YRNDSEYAMKIHTVVHKISHNMTYIFSFCIIFGTVTFNLTPIFNNIGSDAYKNPRPDNVTLQ
QCVYYALPFDYTGNFKWYLLVAIFNVQKTFFCTSLFILFELSLSLMIICLWGHLRIFIHNLNH
IPAPRNSFEYTKEERQEVDDTLKKCIQHHTLIIGFVRIMSETYGLAVLIYYAFQQVVGCLLL
LQCSQMELKTVTRFGFLTLVLNQQLIQISVIFELLGYMSDKLQDAVYCVPWEYMDTSHRK
MVYMMFRQSQIPLQLKAMNMLSIGVKTMVSILKTSVTYYLILKTVTTD-
>BmorOR6
MKEEYYLQHPRTQLFYKVLAHVSTIESTIDLTWWGYTFPKYVGWFYHLQCNVVRLFGKC
VVVSQILFIILNYQTIDKSVFIIAITITPLGALVGIKAESAKAECYVNLMKNFMDKVHIHSIY
RKNENNEFVKKKVIQIERVSRFTAYFLVILIAINCLSWMLKPTLHNIKHFEEIMNKSMEFQY
YIYFWTPLDYKYNLRDYIIIHTLCIYLGATAVTVIVTFDIFNFIAVFHVVAHIQILKNNVKSN
WSDDFNESEKKGYLVSILEYHAYIIRIFGEVQSAFGLNVASNYLQNLIEDGLFLYQIMNGEK
ENVLMYGLMIILYLGGLIFLSIVLEEIRRQNYDLCEYVYALPWEGMSLENQKIFVVFLQRT
QPDLEFETVCGMKAGVKPAFSIVKSMFSYYVMINSRF-
>BmorOR7

MLLYHPNTQVEEKVNNVEEFTYMKFLKSFCKIMDFWPEREEKNSKTRIFRLRYILVLQFCF
TLVAGVLYLKNNFGKKTFYDLGHTIITVVMNVVSVSRLILRCFKKYDVVGQQFINKIHLYH
FRNDSEYSMKTYKAVHKISNNMTYIFSFSIFVCVVTFNLNPVFNNIGSGAYKNPRPDNVTL
QQCVYYALPFDYTGDFKWYMLVAIFNVQKTFFCTSLFILFDLLLSMMIIHLWGHIRIFIHNL
NHIPAPRNSLEYTREERQEVDNTLKKCIQHHTLIIGFVRIMSETYGLAVLIYYAFQQVVGCL
LLLQCSRLDLKTITRFGFLTTMVNQQLIQISVIFELLGYMNDKLQEAVYCVPWEYMDTSH
RKMVYMMFRQSQIPLQLKAMNMLSIGVKTMASILKTSVTYYLMLKTITANEA-

>BmorOR8

MSLSTRCLLKDFCKYVYYAGAGNFWYEDIYKETVPYKMYVVISFFTYTVMIFLENLAALF
GKLPEVEKNSAVMFAAIHNIVLTKMFLLLYHKRSISKLNCEMAAVGENLEEASIMRRQFRK
MRLGTALYFISVYLSLVAYGVESARRTIVEGAPFYTVVTYLPDYDNTTVLASFLRIFFYITW
LYMMLPMMSADCMPIAHLITMTYKFVTLCRHFDQIREKFQINVKIMAKTEATEILKLGFIE
GIKMHQKLMYLADEIHRVFGIIMALQVCESSAVAVLLLLRLALSPHLDLTNAFMTYTFVCS
LFLLLALNLWNAGELTYQASLLSNAMFYSGWYFCDFEKDWCRDIRRLVLIGCAQAQKPLI
LKAFGVLDLSYETFVSVARMTYSVFAVFYKRGD-

>BmorOR9

MLALDDPLQNVDNVEDFKYVKWLRNHLKTVDAWPVYSKSKRKIQKRYVLPIFSAACFIS
QTVYLKNGIGTLSFVVLVHSYICFLINGSCLCRGILIATERCKRLATCYLKTVHLFHHKNRS
EHAMKIHVIVHRLSHYYTIYLISLVFVGMVLFNFMPIYNNINSGAFKSPRPESVTFQHAMY
LALPFDYTTNIKGYFVVFILNWYISLVTTSHFCTFDLFISLMIIHLWGHIKILMCSLEDIEGFV
LGGSFKFTIEQNRKINSILQECIRHHQFTIDFTNEMSSTFGLVILFYYFFYQVSGCLLLLACS
QMDIESLSRFGPMTFILFQQLIQLSIVFELISSLSENLPNAVYNVPWESMDKNNRKMIQVLL
LQSQKLTRFKATSMMNVGVQAMATILKTSVSYFIMLRTMYQEH-

>BmorOR10

MRTNAKSFLFVPSKVLTLCGVWPVEKTSIFSLIYRSIMLSSQFCFLVFNGIYIGLMWGDLKA
VSDALYMFFTQTTCCSKAIGFYFNFMKIKRIVASMDDVLFTAMSIEDQATIFSHSRTVNKLY
KGVLGFTGFTLVQWTVLSLIGSGRTLPFNEMWVPTDISKSPNYEITFVVELWMMVISAALF
MSVDTITVATMMFSCAQLDIIMKKTQQIQEIPLSPDLSSRNRSELHEKNNGILIDCIKQHQAI
VRFSELCEGTFQVHSFFHLGGIVFMICVIGFRMAGESPVSAQFWAALSYLVIILGQLYLYCW
CANELTTKSEQLRDKLYLTPWYDQDVKFKRNLCIAMECMAKALTFRAGSYIPLSRAMFVS
ILRSSYSYFAFLNQANEQ-

>BmorOR11

MDEHSHFETSLNKIKVLFKYSGMNLENTVTNTYEFLNHRWVYILNHAWTLAAVTFICIGIS
NGQNFIEMTCIAPCVAMTVLAVSKSFFHYINENAVKSLLENLIELERTDFERTKSVQRTEIVA
TEKQLLNMVINVLYVLNCSMILVFDMTPLIIIAIKYWTTNKFVRLLPYLDIFVFVPYKFEYW
VMAYILQIWAECIVLLFIGAADCLFFTCCTYIRIHFRLLQYDFERLTSSRRESDGLRDDEDFR
ETYTNLVKRHQGLIESSSILEMIYSKSTLSNFVLSSLVICLSAFNVTVVNDVTIVMTYLIFLA
MSLMQVYFLCFFDMLMSASEEVGNAVYNCSWYTEKASTGKDLLFTITRAQKPCELTAAH
FAYVNLKAFMRVSFTSASITTLPTI-

>BmorOR14

MSNYIFKPFHETYRIITFTMIAAMIYPNPATEKRRLIYIGLMLLSVIPLAFMIVTEMYEFFMA
SDLNNTIRHSTVIGPFIGGFVKVALMYYKRRQANELVSEINRDHLAYNGLKGEDREIAASSI
RNCQIYCELGWTLIVMSCGLSFPVIAILLKIHSFTFKLDSTKHMIHDINNPFTDDPEDRFESP
FFEIMFVYTFFSSFIYIINYVGYDGFFGLCINHACLKMKLYCRALEDAMRSDSRRHEKIVAV

IEEQRRTYEYIALIQDTFNIWLGLIYVATMIQMCTCMYHIVQSFNIDVRYIIFVISIIHIYLPCR
YAANLKCMAAETPTLIYCCGWESVSDLRIKRMMPFMVARSQVIVEITAFNMFAFDMELFV
WIMKTSYSMFTLMRS-
>BmorOR15
MMTLVYQTDIFKPNVFFWKMFGIWADRKSSKTYKYYSFVFLFITLIMYNSLLAINLLYTPL
KIELLIREVIFCFTEITVSTKVLMILFKRNKILDAFDLLNKNEFRGNSEESSAIIQKNNSAYKT
YWKLYAILSNFAYSSQVLGPLIVKLIWKTKLELPICNYYFLNEELRHDFFSGWYIYQSFGM
YGHMMYNVNIDTFISGLLMMAVTQLKIIQTKLLSLKLNPRERKMDRGLMNITEVLKLNEI
LKHYELVLKYCSTVQSILDVAMFVQFGVASAIICVAMCGLIMVRSSTETLLFMVTYLFAMT
LQIFVPAWMGTQLHFQSQELVFAAYNSEWIPRCQSFKRSIIIFVERAKIPITITGLKMFPLSLA
TFTSIMKTAYSFFTLIRNMQALQEE-
>BmorOR16
MSFNSEDLYLNRAKFVMKYLGVWVPPENENFARKFYKIFMMSLQHLFLFFQIIYIVEVWG
DLEAVSQASYLLFTQACLCFKITVFQINMNKLKELLKQMNGYVFQPKNINQQNIIKVQATR
IKRLLFAFMISSQLTCGMWALKPLFDDVGSRKFPFDMWMPVSPERSPHYHLGYSFQLVTIC
MSAYMYFGVDSVAFSSVIFGCAQIGVIKDKIMSIKPLGIYRNHKTYTKISRYNRKTLIECVK
HHQAVISFTELVEDTYNSYLLFQLVGSVGIICMSALRILVVDWRSVQFFSILCYLSVMISQLF
VCCWCGHELSATSEELHTILYNCAWYDQDVKFKRDLNFMMARARRPILLRAGYYISLSR
QSFVSILRMSYSYFAVLDQTNK-
>BmorOR17
MREDKMEINNSQKFYTKMIFRYLYSVGLGDWWYQHEDRSDSHRKLYCLWAVISNAYIFL
NICNELLANFRKDLTDVEKNDAIQFSFAHPLIFAKIASFFFNRKKIREVFGRLLEENRSVYSC
GELEKESMKQIKRYSLAFIGVSYMTLVMSTIDGLRAHFKEGIPIRTEVTYYPSPSNSGVIVNI
LRFLVEFHWWYIVSVMVAIDSLAVASFVFVTFKFKLLQRYFKDMGLTVRRDQSNMTDEAL
ADKFRRDFIVGVKLHENALWCAENVQKAFGWVYSVQVFETVALLVMCLVKLVTTNHNM
IFLLANFAFMLCVIILNGSYMMPAGDVTYEASEVPTSIFLCGWELVRQTDLRFLVVVAIQRS
QVPVIMKAFGIMTLSYSNFIAVSLFKFYVQFQINLF-
>BmorOR18
MGDRMVTRGHFFDFNIKYLFYVGLWPSNEAKRIEKIAYKIYEYQLHVLSLIFLVTTGIGTY
KNHKDIIALLTNLDKTLVAYNFVFKVIVFVYKREELRKLIEQIVQSGDQITEDRKALMAKL
VIVLTGISTVIITAFSCLALFEGEMTIDAWMPFDPMKSKMNLFAASQILAATFVVPCGYRAF
AMLGIVCSLILYLRDQLVDLQNKIRDLRFATGNVEKLRDDFKLIVKKHVRLLGYSKVIEMI
FKEYFFIQNMAVTAELCLNAMMVSVVGLEQKTLAASFLAFLSVALLNAYIYCYLGNELIV
QSEGIAMAAYESSWILWPVDMQKDLLIVITAAQKPMKLSAGGMAVLSVQTYSQTLYNGY
SIFAVLNDIVN-
>BmorOR19
MHEFVINVQNETTKLYDQLNIILYILGLQGIWVDEIKLSRRFHVFFKVVTFILHIMCGMFAG
LQFFAIFTQNSLNSQQKSDVIVIGISNPMAYIFCINFIRNRNEIKDLFYHLAVVLKIYYNDVEI
EKSMVNKIKSYLSTYVFASITILVSNGIIAFFQTINSDEPFLGIITAWPDKTDTSKTASYARIGF
YLFWCIHFFRISTVFAVIVCILISIKYQYKILCSYFESLNKIFDDETSSHEVKEAEFENAFCNGI
KIHTQIIWCVRRCQIMCRTVFSANIMLDTFVLVILMLAMVNSENDFYGLCSQMSSVLVTV
VLMAFFMWTAGDINVQASQLPDAIYGSGWYNCRGKSSARIRSLVTISMNKAQQPILMWA
LGFVELSHKNFVAIIKSAYSVFSVFY-
>BmorOR20

MIQASKYPNSKTKELFRKIAHIAYICGLPNFWIEELNLPKSFIRVYDKIVRIFNVATYFFLGIE
IAAHFTQHHLTNKQKFDLLLYSISHPILNGYGVIVSRQVGNVKKVLLDLIVNLKVKYNDPV
IEEAMIKISMTYSVSFITNCVLSMLTYTFDALLMVYKKGVTFNVIITAWPDVEDTTTEASIG
RIGFHIFWWLFVTRPFAVYVLVINLTTCLSHQYMNLQSYFFHLEDIFKENLSQNEKEAKYE
AEYKIGVMLHANTLRCTRRCHMVWNGVMSGQIIFNISLIVIIMAQMMNSDRTLVNTFGTV
LTASAILISTGFFMWNAGDVTVQASRLATAMYCSGWQNCRGKSSVSIRNMVMNTIAVAQR
PLVLRGLGVIDLSYQSYLSIVKASYTVFSVIY-
>BmorOR21
MNKNMNKNHYILKTYCDKIFLVGSGNFWYQKTESRNDKTLLYKIYSCVLFFTYGFMTVL
EIMAAMMGDFPEDEKRDSVTFATSHTVVMIKFISIIKNKELLKTLNRKMMMICEAHEEQT
LMDEMYRTVKINVVAYCVAVYGSATFYVFEGLRKFYNGSHFVTIVTYYPSNDDDTLAATI
VRIATTLVLLMMLLTMIISVDTYTMAYLIMYKYKFITLRHYFKRLRENVDELVAAGKARL
AAEKLAQGLVEGIKMHNELLSLSKDIDKAFGTVMALQLCQSSGSAVSLLLQIAVTMYLLL
ALFLCNAGEITYQASLLSDEIFYCGWHKCNSPVLSTQRNIRDIVLIAILRAQSPLVMKAFK
MVVRSTYSVFALFYAQNK-
>BmorOR22
MNKNHYILKTYCDKIFLVGSGNFWHQKTESRNDKTLLYKIYSCVLFFTYGFMTVLEIMAA
TMGDFPEDEKRDSVTFATSHTVVMIKFISIIKNKELLKTLNRKMMMICEAHEEQTLMDEM
YRTVKINVVAYCVAVYGSATFYVFEGLRKFYNGSHFVTIVTYYPSNDDDTMLASIVRIATT
LVLLMMLLSMIISVDTYTMAYLIMYKYKFITLRHYFKRLRENVDELVAAGKARLAAEKLA
QGLVEGIKMHNELLSLSKDIHKAFGTVMALQLCQSSGSAVSLLLQIALSDQLTFTMGMKIF
FFLAAMYLLLALFLCNAGEITYQASLLSDEIFYCGWHKCNSPVLSTQRNIRDIVLIAILRAQ
SPLVMKAFKMVELTYATFILVVRSTYSVFALFYAQNK-
>BmorOR23
MRAKTEFEKTIKLTKTALFLSGINIFLGEWNHWTRTFVDSIAYYLNIVGLYFVLIGEMYWLI
DGTITGKSFVELSLIVPCLTISVLATAKVHYLYHNKESLLDVVDKLREIYPDEIEETANDND
QCLNDKKETVYDNDVTEVGIVNEANELLKFVNFLLSTVSFVVTMTFCTMPLFGMAGEFM
ETGKFVVLYPFAVKYPFDVYNTSFWVIVYVNQFWATIIVCTNIFGVDTLFYALCSYIGMNF
RLLSYKFEHLEIKRNDRIINEIIVLIKRHQELIELVNKTQSLYSLSTLFNIVTSSLLICLSGFNIT
ILSRSWSYFALLKTIYS-
>BmorOR24
MPEELFLDRSIKKIESYFRWMGINIRSGDNNNKKDVFKIRCIYFINFVLLNTDVLGAIFWFR
SGLEQGKTFTEVTYNAPCLTFSFLANFKMLSLIFYEKTVHELIAALQKLEIKHFLRQNCAEE
LKMLKDEKNFLHAVFKGSKIVNYASILTFGCSPLVLIASNYYKTGRMDYLLPLIVLYPFDV
DNITVWPIIYVRQIWSVITAVIGVCATDYLFYTFCVYISTQFRLLGHSIERVVPNNGLSVRTR
LNGNLRMKFVENLKWHQELIRAASLLEQIYTKSTLYNFVTSSVIICLTGFNVAVVEDFAVIL
SFLFFLFMSLLQIILLCFFGDKLMKSSTNISDAVYNSKWYLTEKNVGKVLLMVQIRSQRAC
RLTAYGFAEVNLRAFMKILSTAWSYFALLQSLYSSHE-
>BmorOR25
MFEKALRSANFYMRVIGIPTDIRDGNRTLMERLRNRWFYCINFLWLNTDVAGEITWFVKG
LLNGSSTLIENTYLIPCLTLCILGNVKTFFTIKYANHIIDLVAILKDLEIKNNAARKNETEIVK
ERLKFLTTSNKFLLFVIGTGIIAFGIGPLMLTASIYFSSGDMKLKLPFLIWYPFDSSDIRYWPF
VYVHQVWSACIACCAVYGPDCFYFTSCTFIHIHFIHLQNDITNVIVESSRARKNGLYRGCH
QAFLELTNRHKDLIRCVNLLEIIYSKSTLVNVVSSSLLICVTGFNVMVTFCWFAAPFASFLA

LGLVQTYLLCYYGDTIMCSSTEVSDAVYNSTWYGTNISQMRDYLFVMKRAQKPCKLTAY
GFSDVNLRTFSRILSTAWSYFALLITIYRGNGQQ-
>BmorOR27
MPSSFFLPNLENPDYPSLGPTLKGLKYWGMWQSGGIKRILYNSIHAFATFFVITQYVELWII
RNNVELALRNLSVTMLSTVCVVKAGTFVCWQKYWSGIIGFVSNLEKEQLSKNDAATQAA
IVKYIKYSRRVTYFYWSLVTATVFTVILAPLVGFLSSPERELIANGTLPYPEIMSSWVPFDRS
RGFGYWVTALVHTLICFYGGGVVANYDSNAVVLMSFFAGQMKLLSINCSRLFDDGNEVIS
NNEAMKRIKECHYHHVFSTIFNSLMSPVLFLYVIICSLMLCASAVQLTTDGTSNMQRIWISE
YLMALIAQLFLYCWHSNQVLYMALEDRLGGLFEACLESGRFPSKWKTGRLVLLRKDGRP
ADSPAGYRPIVLLDEAGKMLERIVAARIVRHLTETAPDLSAE-
>BmorOR29
MFDFLQNLEDSERPLLGPNFWLINKTGLLLPKTNFGKLAYILVHEIVTFFVVTQYVELYVIR
SDLDLVLTNLKISMLSIVCIVKVNTFVFWQTSWREVLEYVNEADKFERNQTDETRGKMIE
TYTKYCRRLTYFYWSLVFTTFLTTTNTPLMRYWSSPIFRENLRNGTEDFPHIFSSWMPFDK
NHSPGSYCTIVWHVLLCAYGAAIMAAYDTCIVVIMVFFGEKLNLLRERCKKMLANDLYN
HAFVIGQLHDIHVQLIKQSRLFNSLLSPVMFLYILMCSLMLCASAYQLTSATSTAQKLLMA
EYLIFGIAQLFVFCWHGNDVLFKNANVSLGPYESNWWSSSPRVRADVLLLCGQLRVRHV
FTAGPFADLTLSTFIKILKGAYSYYTLLRK-
>BmorOR33
MIYYRKCKMELNFDKIFKIAIISQKFSGTYPYTKRDKKWATHFILMHGELTIICMLFIYNIIE
FDLKAADYSQMCRNMCLSFVYMVITLLYINMLYYQSKLKMLIETMKAEYELAKTMSEEE
QNVILEYAKKGRWLCRAWAILTTCGMAQFFLKSIVCTIYSAIQGNFRIVQYYEVICPEVIER
HRNNPVIFITLYFCTFFYSLYTSALYTSVLPLGPIFLLHGCAKLEIVRLNIKNLFDNDDYVVQ
ERLKKTVLQMQDIYCYSHEINECFQILYEFLLKATSLVLPITIFAVIQALGRGQFIPEFFAFIFG
AFMVGTTPCYYSNMLMEKSEDVRMTLYSCGWETRFDLNTRKCIILMLCRALRPVSIRTIF
RSVSLTTLTDVFQQAYALFNLLNAVWN-
>BmorOR34
MIYYRKSKMELNFDKIFRIAIISQKFSGTYPYTKRDKKWATHFILMHGELTIICMLFIYNIIEF
DLKAADYSQMCRNMCLSFVYLVITLLYINMLYYQSKLKMLIETMKAEYEIAKTMSEEEQ
NVILEYAKKGRWLCRAWAILTTCGMAQFFLKSIIVCTIYSAIQGNFRIVQYYEVIYPEVIERH
RNNPVIFITMYFCTFFYSLYTSALYTSVLPLGPIFLLHGCAKLEIVRLNIKNLFDNDDYVVQ
ERLKKTVLQMQEIYCYSNEINECFQVIYEFLLKSSSLVLPITIFAVIQALGRGQFIPEFFAFIFG
AFVVGTTPCYYSNMLMEKSEDVCMTLYSCGWETRFDLNTRKCIILMLCRALRPVSIRTIFR
SVSLTTLTGVFQQAYALFNLLNAVWN-
>BmorOR35
MKLWQSIREFGLEYCDLPTTLQNVASLLRAITLNIDSRHTARIPFICYVMTVVITLSYFYVFL
VSMAWFVFVRSAETRDYLAAMVVLSLGISSEIGTLKFFYTFIYIKKVQRIVREYLECDHMV
VPESRFADNVLKTMRNVKKRAILYWVVVIGNGVVYVTKPLFMSGRHHMEDRYIVYGLEP
MFESPNYEVAYFLMMFGLCFICYPPANVTVFLIVVVGYTEAQMIALGEEMLRIWEDAVAH
YNNKYHTVGALTNSSEKNKIINQYVKFRLTEIIKMHTTNIQLLRQVEFVFRSAIAMGYVFL
VLGLIAELLGGLENTYLQIPFALIQVLVDCYTGQKVMDASSLFEQAVYDCKWENFDKSNM
KTVLLILQNSQKSMRLSVGGITVLGFSCMMSVMKSIYSAYATLRTTMS-
>BmorOR36
MVFNSKKNIISLFSLLEDSRHPSVGPHLRLLSLTGIWYPNSKTNITLLKRACFYVIVLFFVSQ

YLKCIIKFKIDSLQLILEYAPFHMGIVKTCFFQKDYNVWQDLVSFISKTERDQIAKKDPKSI
KTIQSYISRNRKITYSFWALAFIANIGVFSKPYQNNQSDVNGTVTYNHLFDGYTPFSEEPPG
YYFSMGIETILGHVVSFYVLGWDTLVVSIMIFFAGQMQMSRLQCSRMINGSPERTHKNIIK
CHKFHTDLIKYQKQFNSLISPVMFVYLFVSSINLSVCIVQIAEIEDDFATVLSSFIFLLACLIQ
LLLFYWHSNEVTVQSELVSYSTFESNWTSTQNKLQKEVALLGLTTSKTLVFTAGSFNHMT
LATFISIIRASYSFYALLNSTKY-
>BmorOR37
MELGCSRHLKLPCSLHPIGISKHGNTLSELLIYFPAIPKITYAILAVLLTVYYYIYLCSITWFV
FVRCPQTGDLAAASIVFSLGVSSEIGAIKLFIIAKLRDITGEYLQCEADMAPGRLRARVGRS
LRTVRRRAFVYWLVLVVNAFAYDLMPAFLPGRHLSEDVFVIYGFEPMFESPNFEIASTLMG
VSVVFICYTAGSISAFLIVIVGYSEATMLALSDEISCVWDDACASECQQPNDFIRARLGKIV
AIHTKQIRLIREVEVVFRGALAGGFACVAFGLIAALLGGLENTFLQLPFCVIQISVDCFVGQ
RLRDANVAFETAVYNCKWEYFDKSNMKTVLLILQNSQKTMGLTAGGVAALDFTSLMTIF
KSVYSGVHHSQTDD-
>BmorOR38
MNLSQSVNEQANEYVKMRLERISKIHSPMLPFEDIQDFRELCCIPLAVYAVTGSITASYVYA
FLISLLWFLFARCTDPEDFQVAMVVFSLGISSEIGSTKFFNSIIYIKELRKLFKDYLLYDATCP
AQGRLRLHLLTTLRYVKRRAIIYWLVIIGNGFIFAIKPLLVEGRHLAQDDLVLIGLEPMRQSP
NYEIAYAIMTMGVCFICYPPAHVTMFLIIIVGYTEAQMLALSEELKHLWNDAIEHYEKHSR
TEREADAAMKSKILNSFVNFRLVQIIKSHSTNVNLIGRVENVFRGSLAVGYVFLIVGLIAEL
LGGLENTYLQVPFALIQVAIDCFIGQRVNDANIDFEKAVYDCKWENFDKRNMKIVLLLLQ
NAQKTVSLSAGGIAKLNFSCFMSVIKSIYSAYTTLRTTMK-
>BmorOR39
MLWSVFSYFTRADDVLAGIVIFSLGVSSEIGLVKLCFMYANIDKIQKITEGYLKSDAASAR
NSRFSKNILHTMQSVKKRGVIFWLVIISNGVVYLVKPIVTPGRHFMEDQFIILGLEPKYETP
NYEIGFFMMAVGVCVTCYLPANITAYLITVAGYSEAQFLALGHELANLWPDAQLHCRAM
NLSQSVNEQANEYVKMRLRELVKIHSTNVNLLRDIEGAFRGAIAVEFLLLIVGLIAELLGG
LENTYMQVPFALIQVSVDCLTGQRVMDANLALERAVYDCRWEEFDASNRRVVLLLLQNA
QKVATLSAGGIATLNFSCLMAVIKSIYSAYTTLRTTMK-
>BmorOR40
MTGAGAGTFRTGAGPGRGDGVARRGESGETTTLGRDAFAALGCFGAADGSTARARFFPR
VTVLNPSEVPGSGLAADSNSISDSESEPELDAAQDAIDAGAGVGGDIGESRARTVFGIQGH
DASDSALRMHNNVAIYAKTTMSGNSQLTFATAATIFLKNASGPNGVAIGTDYAICVVSLSLF
FCYRFTELVEDTYNSYLLFQLVGSVGIICMSALRILVVDWRSVQFFSILCYLSVMISQLFVC
CWCGHELSATSEELHTILYNCAWYDKDVKFKRDLIFMMARARRPILLRAGYYIGLSRQSF
VSVSIPRIRFNAILVI-
>BmorOR41
MMGNSTDLFLDRTKRILNFFAMWRSFEKPIPLKVYMAFIMTTQYLFLIFEIIYIVNVWGDM
AEVSEASILLFTQASVCYKMTAFISKTNNFVILLGLIESEIFSAQTELHEKILILKARKIKRLC
MFFLVNAVTTCSLWAVIPLLDISSKMLPFKIWMPVSTGESPHYELGYLYQMITIYISAXLFIS
VDSVPLSMIMFGCAQLEIIMDKIGKVKSWSLDQQPMQKQEVLNSNYELLVECVRRYQSV
VRFIELTEKTYHANIFFQLSGSVFIICNIGFRIAIVDSNSLQFYSMLTYLVTMLSQLFQYCWC
GHELTIRGEELRETLYQSPWHEQDIRFRKVLIITMERMKRPIIFKAGHYIPLSRPTFVAILRCS
YSYFAVLNRVRNE-

>BmorOR42
MDIPKFEELLKQIKMNFWLMGIPFDNPKIQIRYYVLLLPLSLMLIEEIAFFGSRMSSENFLEL
TQLAPCICIGVLSVLKILALTAKRQKIYELTQNLECLHKIILNDTRKTELVRKNLVLIKFITKY
FFVLNAVLIFVYNFSSPVIIAYNYIVSNEVQFVLPYAVLLPFKTDSWIPWLIVYVYSIFCGFTC
VLYYATVDVLYCVMTSLVCNNFSLISFKLQKVNRNTAHLLKEVVKEQQYVLKLAEDLENI
FTAPNLFNVLIGSVEICALGFNLMIGDLTQIPGCILFLSSVLLQILIMSVFGENLISESSRIAEA
AFLCKWYEMDQKSKKTILTIMIRSHKPKKLTAYKFSVISYGSFSKIISTSWSYFTILRTMYTP
PGTKFQDDL-
>BmorOR44
MYTYFKVLVFWLNKDKVISLQKILHCKEFKPKEPEHKEIIRKSIRKARFVMTSYATMCVGA
VSVGIILPLTENFDILPTNVEYPFFDVYKNPTYAYLYLHHIYYKPATCIIDGVMDTILAAFVA
SAIGQIEILAFNLRNFDVLAERRRKRAISGNKYIGKYTNLYFTKRILKECILLHNSIIRYVSVI
ESAFSLASALQFMLSVMVLCLIGIQFLSIENPTSHPMQMVWMAIYLTCMLIEVFILCWFGN
ELIWKSNDLRQAAFDGPWRNLNRKTCMFIIIFMERCKRPMRLSAGKIFTLSLDTYTVLINW
AYKAFAVMRNMKK-
>BmorOR45
MKVLDNVNHAVKVTMNCCRLYGLFVSDDLTKRQLIIMRAFSLMLYLFFVGFFITTQSALII
TMWGDLNLMTNVGLVLGTHLTLSAKVFTLHYKEKEITNVIYKNEVRLRAETREQGKYIIS
EMNRETTLFMRLFIPFGMGTVTAWLLCTPKGELYTPAWYPCNTTKSPAHEIILAHQGIAVIL
TATLEIAIVLLMTSIVAVCRCRLKLVGLSFETICDDLPSNIMNKLTADEQVIVAKRVRENVIE
HQAVLECINDIQDCFSSAMLVHIAISTMIICATAYQLAVEKSLDLTQRMTMASFLGGMSTEI
FLFCYQGGHLSIDSMEVATAVYSCPWYTFPTSLKRSLLVIMIRAQQPALLTAGGFAPLLLDT
FVSIMKASYSFFTVLQNASE-
>BmorOR46
MAFFIRNKMLGLTITLNTLSWAGLIMRDQYTKTQRIIVRVYGWLVFLYLFVAATYVQIADL
IDIWGDLDLMAETSLLLFMELAVISKILTLIFKYDKIMEIINGTEDILCSENRLEGQKIIASIDK
ETTRFFQYYTSSVIFTTFFWFLGEHSSTFFIRAKYPFNELKSPGYEFALIHQCMMMVFTGYF
EFNINIFFASVVAGCRCRLKLVALSLRNICINIPVNKKNLITPEEEKLITERLHCAISQHKYAL
DAAEDVKHCLSKVLLVQLTVSIVIICTTAYQMAVNKSTDTIQKLSMAGYLLGASFEVFLFC
FQGQSLSNASEDIADAVYECPWYTLTQPLKRTLLIIMMRAQSPAILTAGGFVTLDITEYMAV
LTGHGGFGDFLHRTGAEPMAECHHCGCDLDTVQHTLLVCPAWKGWRRDLVVKIGNDLSS
VLWHRCSAATSRGRRCLTSASAPSRRRRRGA-
>BmorOR47
MKLVFDNFIFALKVTLNWCRYFGIFIPDELTGRRQKLLVQAYSVFMFMLFIGFFIITQIILFIL
VWGDLSLMTDVGLVLGTNLALSAKIAVFFFKREELASILKKNDDTLRFETREEGKKIISEID
RETNAFMKVFFCFGVGTVIAWFLSTPKGELHIATWYPCDTKRSPAYEIIMIHQLAITADLLM
LSMIAVCRCRVKLVGLYLQTICDDLPCNVKNKLTSDEEVIVAKRIREYVIEHQAILDCISELQ
NHFSPALLVQLLTSVVIICVTAYQLAVEKSSDLLRKFTMASFLFAMSTEMFTFGYQGGHLS
HDSMEVATAAYSCPWYTFPTSLKRSLLVIMIRAQQPALLTAGGFTTLSLETFVTIMKASYSF
FTVLQEATD-
>BmorOR53
MALKKMLALTKGLEDPTHPLLGPTLKALSVFGLWQTGSQKSTVIYNTFHFLTFLFVITEYI
DLYTVRKELSKMLNNLSVTVLSTICMIKTLSYVCRQSHLKVLVREISELELELMKTTDKNI
VKRLRQYTVYTRAVTYVYWFLVVGINVVLLTSPLLKYASSEIYRSEIKNGTEPPPLILCSWF

PFDSARMPGYFWATMVHIIMSIQGCGVVATYDMNAVAVMSYLKGQTSILKDKCKAIFDET
ASSRDVLNRIRDCHRHHNILLRHYYMFNSLLSPIMFVYMLICSFTICCSIIQLDSSETTISQRI
WIIQYSIGQISQLFLYCWHSNEFAAKVKKKHFPLFPINLF-
>BmorOR54
MGLNTIKEFFVNVKRRFQDVSIDSLLWIVNIVPSLAGFSIRSDRVSAPFWIVHWSLLVYVYA
VGNAVYQWKFANEAIDYITSFINVSLLILIGNNSWWFLANRRLLKSVLHKIEVNDELSRRS
EQSRLKHKKLLKIIKRIVLVFYMSNYVNASFIYLPNRVDVLNNYAMTPCVGMEPLTVSPNR
ELCLTILCMQEFSIMTVVLNFQALLLCFIAHTAVMFQILADEIMALNNYENLEEHQAYVKE
MLPIFVKRHSLTLSAVDNYKSLYSVPLGVNFGSNALTILLILYLPVLEWFKFIPIFVFCFMLFF
LYCFLCQKLVNASEAFETAIYCCGWENFALREMKMIYVMLHQAQKPVELLAADIVPVNM
NTFATTLQAMYKFVTVVKF-
>BmorOR56
MKLLEKLEDPDRPLLGPNVKALKFWGLLLPESRSKKYFYLFMHFAVTVFTATEYIDVWFV
KSDLALLLNNLKITMLATVSVLKVTTFLLWQNAWRDLIGYVSRADLEQRATSDSRKLALI
NGFTGYCRKITYYYWFLMYTTVAIVTVQPIFKFFSSAAYRLDVQSGNGTYLQVVSSWIPW
DKNTLPGYLLASIYQTYAAIYGGGWITSFDTNAIVIMVFFRAELELLRIDCAALFDDEKSFG
DMAFMRRLKECHRRHTELVKHSRLFDSCLSPIMLLYMFVCSVMLCVTAYQITIETNPMER
FLMTEYLVFGVAQLFMYCWHSNDVLYASQDLSRGPYESAWWSRDVKYRKNLYILVAQFN
KVIVFSAGPFTKLTVATFIRILKGAYSYYTLLSQSQMNKT-
>BmorOR57
MPSLLKTESLALTLTLNTLSWAGLILRDDYTKTQRIIMKVYGGLVFLYLFVFTAYVQIADLV
VIWGNIDFMTETSLILFMQLAVSAKVLTLMLKSKKIMEVTNEADAILNSEKKVEGQRIIASI
DKNTTLFLKYYGFFVAFTIICWFMGENTSTFFIRSKYPFNELKSPGREFAFVHQCIVVIFTGS
FDFNVDIIIISLVAVCRCRLKLVALSLRNLCLDIPMNKRNLITSDEEKVITERLRNIISQHKRA
LDAAEAIKHYLSGALLVQLMVSIVVICTTAYQLAVKKSTTMQSLTMAGYLFGTSLEVFLFC
YQGEFLRESSEEIADAAYECPWYTLTRPLKKTLLIIMTRAQRPATLTAGGFVTLDITEYMAI
MKASYSFFTVLQQVSE-
>BmorOR58
MKLVFDNFIFALKVTLNWCRYFGIFIPDELTGRRQKLLVQAYSVFMFMLFIGFFIITQIILFIL
VWGDLSLMTDVGLVLGTNLALSAKIAVFFFKREELASILKKNDDTLRFETREEGKKIISEY
PCDTKRSPAYEIIMIHQTIAVAVIASLAITADLLMLSMIAVCRCRVKLVGLYLQTICDDLPCN
VKNKLTSDEEVIVAKRIREYVIEHQAVLDCISELQNHFSPALLVQLLTSVVIICVTAYQLAVE
KSSDMLRKFTMASFLFGMSTEMFMFGYQGGHLSHDSMEVATAAYSCPWYTFPTSLKRSL
LVIMIRAQQPALLTAGGFTTLSLETFVTVS-
>BmorOR59
MDTNPSAAGDSVAPHLRRLRQVGFCQLDPTSQSRRPILALMHRVYHRLVLAATVLYIFEQL
TYAYQARNDMERLSRVLFLMLCHLTCIAKQFVFHSDADKINQLVVGLDDALCNQPVETH
RLLLLETSRRAARLLMLYSGCAVSTCILWAVFPLLDQLRGRTVEFAFWIPIDYRHNAFQFAV
VLAYAFYSTSLVAVANTTMDAFIATVLYQCTTQLRILRMNFESLPERAYALSRKTRQDYHT
VTHELLVDCLLHYKKITETCNLLEQIFGKAILVQFGVGGWILCMAAYQIVDMEILSIEFAST
ALFMGCILTELFLYCYYGNEVTVQSGLVSESVYAMSWLSLCPRERRALVVVLERARRPLR
PAAGRVVPLTLNTYLKILKSSYSFYAVLRQTK-
>BmorOR60
MVRPCRYFAIHFILLRFLGLGWWHHPHENETRNYPGLYLYYSILTQLVWVVGLVGLETIDP

FVGEKDMDRFMFSLSFVITHDLTLIKLYIFYFRNVEIQDIVRTIEIDLYRYYQNDDKIRATIRI
SRIFTAAFLFFGWVTIGNANIYGIVQDLRWKDIVKNLNETTSKPLRTLPQPIFIPWPYQEDK
HYILTFILETMGLLWTGHIVMTIDTFIASVILHMSTQFAILREAIVTAYDRTMIALSEGALQS
GVLCENSNGNEENNQIFLESFYSKEHIESVLESTLLSCIRQHQLLIGCVEKFSKTYSYGFMT
QLLSSMAGICVVMVQVSQGASSFKSVRLVTSLAFFFAMVIQLAIQCFTGNELTIQAERIAD
AVMESKWEKMPVRLRRLLLVTMMRAQRPLHLTAAGFAYIDNTCFLSILKAAYSYYAVLSQ
KQG-
>BmorOR61
MARITDVFRLNFIFWKFLGIWGKSAPSKYNMAYTALYLSASLFVYDIFLTLNLIHTPRKLET
LLRETMFYFNHLVAMTKILKMFIRRKKILVIFDLLDCEEFKPSDEDSQEIMKRKNEFYYIY
WRIVAVTSNLSCFMQVVGPLIKMLIWKSELGLPVCKYYFMSDEFRNKYFVIWYIYQSFGIY
NQMVNNLNLDTFNCGMLWMAVGQLQILKTKFVNFKLNDIENSLDLKTRDDMQTERLRK
YLTHYEIILKYCATVQDILNITIFVQLGMSSIVICVGLCGFVAMPSNTETAIFMSSYLITMTM
QIFVPSWMGTQISFECGELMSAAYCCEWIPRSKLFKRSLILFVERAKTPVRITGLKIFTLSLD
TFTSIMKTTYSFFTLIRQLQVDEVN-
>BmorOR63
MKLWIRNANFTISLSLTLLRCLGFWSPDGLAGNKRLLYNCYSFVFFMFLLGIYILIQVVDMI
KIWGDLPLMTGTAFLLFTNFAHATKVINIVIRKNRIQRVIQQANAVLMGVQSEEARRIVKS
CDFETSIQLCLYFLLTFVTTVGWATSAEKHQLPLRAWYPYDTSKSPAYELTYIHQVAALLIA
AYINVAKDSLVSSLIAQCRCRLRLVGLALASLGQDLKIDYQSQLSPAQENILNLRLKTCVLE
HQTVLAAVTELQACFSKPTFAQFTVSLIIICVTAFQLVSQTGNLVRLLSMGTYLMNMIFQVF
IYCYQGNKLSVESSEIAGSVYFSPWYLGSVKLRRALLIVMVRSRRVAKLTAGGFTTLSLAS
FMAIIKASYSLFTLLQQVKQKK-
>BmorOR64
MGVSNGRGTVKPFLYPLVDELDYNLIVGVHLPFEYKTPSRYPLAYITVVIAFIYVSYFVMV
TDLIMQAHLLHLLCQFNVLADCFENMLNDCVKGFEGPLVSLHEYIHPLIDEFEYNLMVGL
RLPFSFDTPLRYLFTYVIVLIAFNYTAHYVMVTDLIMQSYLIPLICQYAVLADCFENILIDCS
NDYGDHARRNDIVYSRSMELRAILSRPMLGQLASSGLLICFVGYQATTSISVNIVKCLMSL
FYLGYNMFTLFVVCRWCEEITNKSLNIGNAVYCSGWESGMTVVPTVRSTILLVILRANKPI
VFTAGGMYNLSLTSYTSLVKGSYSALTFLLRIQHE-
>BmorOR65
MRLGFEVSISEYLYRNIFYIYTLFHILLHFYYILHMIKLDLEAIFDDIDESVALLPHRDTRRIE
VQKILNGRMKRVVTWHISVFKAVEAVSSIYGPPLAYQVMFTSIAICLIAIQITQKLENGILDI
RFTMLGVAACLQMWIPCYLGTLLRNKAFGVGEACWNSGWHQTPLGRMIRQDIIIVLLRA
QQPVTIKFPGLQSIQLETFSSVIFNLYGYYYFLLLRWVDELTAHLVLSGYWSP-
>DplePOR85d
MIPVYLILVQGIIKATVVCTKKTEIESIVEGLGAIWRTDEMLTKNQIEKKHYRLKKFNFYLT
VFYWGSMVASYQNLFTTICTILFKRLVLQQDPELEFAFGNAYPYEFTKNWFTYLSVAQETT
EFLKNFSAVLALMGPIYILCYYAEQLQEESVGIANAAYETLWYEGDKTFQKNIFLIILRSQR
ACCLTSLNYAPISLNSFTKVIQHDTEMKKYT-
>DpleOR3
MVFEDRVFDIPLVKSIFIRKPGHGKLLELEKLVVNSQEKLFKLTRVLLKTYVGSVWLCVTL
YLCGPIYQMCVTKDKSLRLLAFDMWFPYGLEDFRVYVATFIFHAYAGYLCCIAYPGLQSTI
ILFVGQIIRQLQILTFILNNLDEIAKELVGKRGELWQETCIQIFSQCVDHYIQTKRFSNKLNVI

CQPFYLALIVVATMLVCVCSVKIAISCEKLEAAVTEKWYLFNSSHKTNIRIFMMAMCQKM
PIYIYGSITLSLQTFTWFIRTGMSFFTFLMSVLED-
>DpleOR12
MGKSIDLLSRVAPCSALCILSTAKTLPMVTNNDILKDAVLKLRRLHQGVDLRNSFECDELM
KTSTILKNIITVLVVGFVCAVVLFTSMPGFLMAYDFFTTGTYQLQLPFFTKYFVDVFADARI
YTFVYLHQFVSTVLVGLNIFAADTLFFAFCSYVKMHFRILGHYYENIVGQSESETRNNLRR
CVRTHQVLIKLVNQINILYSKSTLVNIMTSSFIICLCGFNITITKSKDMSTYLLFIPFLMNSLTE
IFLMCFFGDQMIDSSYNITQAVYSSRWYDAGEDLKRSVLLILFRSQQSCKITAANFSDLHLR
SFTTVCVYIIL-
>DpleOR2ORco
MSAVVYFSLALCMMALYANGSVIKLQDLSLTDGVVLKLVIKEDPEQEGKAVASIKIDMDH
PKPKNVRNLFHKLITTGSIPKFEDRILITNNNCPLGQELKKSIKMITKSENRGLVSDLMPNIK
LMQMAGHFLFNYHSDNSGMSSLLRKCYSSIHAFLIVMHYLCMAANMAKYSEEVNELTA
NTITVLFFSHSIIKLTFFAITSKNFYRTLAVWNQSNSHPLFTESDARYHQLALTKMRRLLYFI
CGMSFFSVICWVTITFFGESVVMLVDKETNETITEPAPRLPLKAWYPFDAMSGTMYIAAFA
FQVYWLLFTIFIANLMDVMFCSWLIFACEQLQHLKAIMKPLMELSASLDTYRPNTAELFK
VSPEKSDKTPDPIDMDIRGIYSTQQDFGMTLRGAGGRLQNFGQNNNNPNGLTQKQEMLA
RSAIKYWVERHKHIVRLVSSIGDTYGTALLFHMLVSTITLTLLAYQATKINGLNVYAFSTVG
YLSYTLGQVFHFCIFGNRLIEESSSVMEAAYSCQWYDGSEEAKTFVQIVCQQCQKAMSIS
GAKFFTVSLDLFASVLGAVVTYFMVLVQLK-
>DpleOR44
MYTYFKVAVFWLNKKKILNLLKFLYCDEFRPEEKEHTEILKKSIKLARFITTYYITICVCAV
SVAIILPITEDFEILPTNVEYPYFDVYKPPLYVIAYVHHIYYKPATCIIDGAMDTICAVFITFAI
GQIDILAYNLRNFDVLAFKKWQRDLKHHKNTSYDCKHYVNYILKKCIVHYNAIIRYVSMI
EDAFSLASALQLMLSVMVLCLVGIQILSIENPREHPMQMAWTAVYLSCMLQEVFILCWFG
NELILKSTELRQAAFDGPWLKMDYRTMMLIVIFLERCQRPLQVSAGKIFILSLNTYTYLIN
WSYKAFALMTNMKK-
>DplePOR8
MLYNINIDSFFIGLLILAIAQIDILDEKLKNIATNIWDADGMVNRDIDEICIRKLNDCILHYN
NISKFCLLVEEVFSVTLFVQFSMSSFVICVCLYRLTQPAPLPYYLFLGSYLFIMIIQIFVPCFFG
ARIMDKSSQLSHAVYSCDWTPRSRRFKSSMRLFVERSLRPHSITGGKIFPLSLVTFTSIMNA
AYSFFTLLTNVQSNET-
>DpleOR18
MSFFKRFTDRVCESDLFDFNLKYLYYVGLWPREDWPKYKQILYKIYEFKLHLLSITFIVITG
IGTYQQRNNVIMLMTNLDKTLVGYNFVLKIFFFVIKREKLNVLINEIKNSGDKVSDDRKTL
MAIHVIFITFISTALVSAFSLLSQYKAEMTVEAWMPFDPFKDRMSLLMAAQILAICFVVPCL
YRAFAMQGIVCSIIMYFCDQLLHLQGNLKKLGNTKHNEMQARKEFKGIVKKHVRLMRYT
KTFTEIFKEFFLIQNLAVTIELCLNALMVTVVGLDEKTLLASFLTFLGLALFNAYIFCYLGDE
LIILSTGISQAAYESEWTSWPIDMQKDLLIIIKVAQRPLKLSAGGMAIMCIQTYSQALYNAY
SIFAVLNDVVD-
>DpleOR42
MDIPAFNDIFEQIKINFWLIGFVCVLYFATVDALYYILTSHICSQFALLSEEIQSLDENTAYSLT
EIIKKHQHILKLSQDLEIIFRAPNLFNVLIGSLEICALGFNLTMGDWSQIPGVILFLLSVLIQIL
MISVFGENLIRESMRVGQAAFFCKWYTMDRASKKNILILMLR-

>DplePOR9
MEVGSSIYNSRWYKMDAKSVKSLLIIHSRSLKPCKLTAFGFFDVNLKSFMSIMSTSWSYFA
LLKTVATPTKT-
>DpleOR56c
MHISVIVFTATEYIEIWFLKNDMNMVLTNLKITLLATVSVSKITSFLIRQKDWKGILNYVND
TDRARRSEEESGKKEIITKYTRYSRMITFYYLLLMYITVIIVICQPIYKYYTSEEYRHNVRN
GTEKYDQVVSSWVPFDKNTVLGYIFACLIQSYAAIYGGGWITSFDTNAIVTMVFVKSEIEM
QIIDGRLIFGTEKEPASVKEAKRRLMRCYDRYSKLIR-
>DpleOR6
MAYTGGPILISFVVDEVKRQVVCILNLLINILINSLLTYFFFLKADDLPDLVYGMPWENMS
VSNQKTAMLLLQKVQIPMEFEALGGLIAGVRPMISILKTTFSYYVMLESSMESKSA-
>DpleOR35c
MPKIFGKFRPEYCDLPTMVWNVEVLLRTLTIRSYRRLNKPISWIFYILTTLSGLFYIYSYVIS
PVWFLMVKSRETGDFTAAIVELSLSMCSLTCVPKIFYMKYYSSTVMDLIDEYLRCNELIVK
GTRFEKNFLKNMKTVKKQATVTWAVSMLNSATYLTVPLLRPGRHFTQDMYVIYGLEPML
ESPNFEIATFMMVLSIIFALKVIVYITLFVIVIIGFLESQMLALSVELTNIWEDSKKFYNYYIL
QHNESGDEYEVRNVFVTNRLRDILKFHIMNINLRNKVEKELRFIFVIDFIFMMLGLVTELL
GGLDNTLFQFSFTLGIVFIDCCIGQRLIDASIAFETAVYDCKWENFNLSNQKTVLFMLQCSQ
KTMTLTAAGIAVLNFIFLMSVLKSTYSAYTTLASFT-
>DpleOR29
MLKALLSSIEDPTRRLLGPNYWILKNMGLVLPQNPIAKVAYIVLHEIVSFFVLTQYMELYVI
RSNLDLVLTNLKISMLSIVCIVKANTFVIWQVQWKEVIEYVTRADQYERDNQSVERRKIID
SYTKYCRGIVYFYCVLVFTTFITVTNTPLMRYLSSAQYREALRNGTELFPHIFSSWMPFDK
YNSPGSWITVIWHVCLCAYGAGIMAAYDTVIMVIMVFFGGKLELLRERCTHIFENTEIASD
DNQTNEKIRKLHNIHVLLMKHSRLFNSLLSPVMFLYVVMCSLMLCASAFQLTSATNTTQK
LLMAEYLVFGIAQLFMFCWHSNDVLIKSQKVMFGPYESEWWTVNSKQARSILILTVTFLV
LLSDKQFDMDNQTNQERTVPIQYVRGSRAFRQFKNPPQPHMCIQDTIKDTTEKLYINVLG
WQKIANPKQYSDPIPLYGGMQVHQGCGPNSNKPPLLVFAVMVNPDILKANGKNAANPTD
REALVSLLCDFVEAMNPGLLLTRNPIIMKDKDITGELKDVWLAVQNKREREKGLNQDVM
YKVYDIDGIGSEDGIEDEQALRNCQQNDTGSPNRIQMSDKKNTVKSSKQILKNAGQKSEF
DSGMNNYQLNQNQSSRDNADTTYCTPVYGQIVSSRENHNQINDIQQKFASNEITKPSSSFK
KDWQSPHGKSTTGWDEFSKRSLPPKTNDKYKSRSDKSNKPMSNGKSHYDFYPVFDNKA
SIVASPEYLDADKNSVIEQDIKDNSKIIIDPMQKLVLHSTDNKICDNKTSALSSFSS
>DpleOR56b
MIFFRGELELLRRDCRSLFGTEENPVTKEEAQKRIDHCHQRHNDILKYFRLFDSCLSPIMLL
YVIVCSVMLCATTVQLTTESSAMQKLITFQYLMFGVSQVFIYCWHSNDVFYVSQDLMLGP
YESYWWTRTLSEQKNLHLLAGQLNKQIVLTAGPFSYINLATFINILKGAYSYYTLLN-
>DpleOR60
MPESGKTKGRRFFVVHYILLRFLGLGWWHSPEENNTNNFPGIYLYYSILTEVVWVAGFVG
LESMDPFIGHKDLDRLMFSLAFVVTHDLTIVKLYIFFFKNQQIQDIIRTLEIDLHQFYQNEEK
NRKTVRITRIFTGLFIFFGWMTIGNGNVYGIIQDLRWKAEVAQLNDTSLRPFRTLPQPIYIP
WEHQNDLSFIITFLLETVGLLWTGHIVMTIDTFIGSVILHMSTQFSIMQEAISTAYDRTMDK
LIKGVIECNDPVALNDCEASLGESRRDMIVRTYCTKQEIEIALENTLKSCFRQHQMLISCVD
KFASTYSYGFMTQLLSSMAAICSVMVQVSRDASSLKSISLVTSLAFFGAMIIQLALQCFTSS

ELTHQAERVSEAVMCCKWERMPVRLRRYLVLMIARSQRPLQLTAGGFATMNNDCFLAILK
AAYSYYAVLSQRQG-
>DplePOR20
MYPNPVYDEKRKKLIVMAILSVLPAFTLAGNDIRLRILNNDMANVVRQSIILVSITFVIIKFII
SIARKDEFRSLLEEIDADYERFNGMSEQYQDLVEDTIRKTRKVEKSWCFVLLVTTASYPVL
AGACTIYSQLFSDYPRRYMVHELKAILISEEQKYQSPYFEIVSLYTMYIVIFLFIGFTGFDGM
FSVCLLHVSLKLKIYQENLRNLFNEKDIQKIKYNIGLFVKNHCGVLRLIAKIQTCFEVWLV
GIFINAVVQIGMAFTQITNQTESDINQMYYLYALATVVHIYLPCYFASDVTYNAAEIANVAY
SSSWERVQDSKIRSSICFIIAKCQTPVRLTALDMLTFNMELFVSVGHQM-
>DpleOR8
MASAMEEIEKENVMKQQKLRVQWGIIFYAVTVYLSLIAYGVESLRKVLVEGTPFYTVVTYI
PDYYDESIIANALRIFFYMTWLYMMLPMMATDCMPIVHLITMAHKFKTLRHYFEDIRVEF
DENLTIMGTEPAANLLTARFLNGVTMHHKLMFLAEEINRVFGIIMSLQVCESSAVAVLLLLR
LALSSHLNLTNAFMTYTFVGSLFYLLALNLWNAGEITYEASLLSNSMFYSGWHLCQLNK
HRHKDIRRLVLVGCAQAQKPLILKAFGIQDLSYSTFVSVARMTYSIFAVFYQRN-
>DpleOR35b
MMWNVEVLLRTLTIRSNRRFDKPIPWIYYVINISTGLCYFYTYMLSTAWFVLVRCRETGDF
ISAMVALSLTMCSITCIPKIFYMKYYQNTVMDLVDNYLHCNELIVSGTRFEKNFFANMRTV
KKQATIAWVILTLNGVVFVTLPFLRPGRHFSQDLFALYGLDPMVESPNYEKASILLTLAVYF
SLTVMVHITLFIIVIIGFLESQMLALSVELSNIWEDSKKFYNYYVLHHNESITKYELRNLFIK
NRLRDIVKFQIMIVSLRNKVEKELRFIFVNDFIFMIFALVSELVGGLENTMVQMPFTFGMVF
IDCFIGQRLIDASKAFERAAYGSKWENFNLSNQKTVLFMLQCSQKTMTLTAGGIAVLDFIY
LMSVLKSTYSAYTTLKSSA-
>DpleOR35a
MTVSKSPVSGYLNKMYQYTENIFPETGDIRRAAVELSLATASGTCVAQFLYMKLCSNRLM
DIVDEYLSCDKLVSRGTRFEKNLSWKLREIKKRALVVWIVLIGNAALYVTVPIFIPGRHFTE
DLFVLYGLDPMVESPNYEKASILLTLAVYFSLTVMVHITLFIIVIIGFLESQMLALSVELSNI
WEDSKKFYNYYVLHHNESITKYELRNLFIKNRLRDIVKFQIMIVSLRNKVEKELRFIFVND
FIFMIFALVSELVGGLENTMIQMPFTFGMVFIDCFIGQRLIDASKAFERAAYGSKWENFNLS
NQKTKKYEVLGNNTTYLKEDLHLVCYRNVQNSRKNSNEDDRQEGEWENFNLSNQKTVL
FMLQCSQKTMTLTAAGIAVLNFIFLMSVLKSTYSAYTTLASFT-
>DpleOR27
MAILFVLSQYVELYFIRNDLAMALKNLSVTMLSTVCVVKAGTFIIWQKYWKDIFNYVSKL
EKNQLYKPDYLTKNTIRKYTEYSRFVTYFYWTLVTATVFSLILAPLASYLSSFQNKELGDS
GASSYPEIMSSWVPFDKTQGCGYLVLTLIHVLICFYGGGIVANYDSNAVVLMCFFAGQLKI
LKINCERLFGNGEEIAGTTSMQQIWIAEYLIALVAQLFLYCWHSHKVLHMVNIFIH-
>DpleOR56a
MILYVLVCSVMMCCSVVQLSLGHLSTSEKLWVIEFTTALITQLFLYCWHSNEITYESNLVD
RGVYASNWWRGDVKVKRQILILAGKLAPSLILKAGPVTTLSMATFISILKGSYSFYTLVTQ
MQENQI-
>DplePOR12a
MAKIYKIKRYKPVFINLISELREMWPEDNVTDEEQAIISSALNRLRLVTKTYFSCNSILGLIF
TLPSIINLIKPLFGIEAPRILPFFYWLPFDPYQEVIFEVVVIVQNSHCFLSAAFMLAGDLLFFS
FLSNITTQFSLLAVRIKKMFYAPIDGQLPESYPLGCSNEGILLNNLSQDTSTNRSKVKEEELK

EIIKRHRSLIRLSNDVENMYSFSLLVNFLNSSIIICFCLFCCAVSTKTVKNY-
>DplePOR12b
MMPCVGYLFVAIAKFYKIERYKPVFINLISELREMWPEDNVTDEEQVIISSALNRLRLVTKT
YFYCNLILAFIFTLPANINLIKSLFGIEVPRILPFVYWMPFDPSKKVVFEVVLIVQNIHCFLSA
AYMLAGDLLFFVFLSHITTQFSLLAVRIQKMFYVPIDGQLPESYPLGYYNKENSLNNPNQD
TANNRCKVEEEELKNIVIRHKALIRVVILIDHFQSTGVGEALYNSAWYVSSNSIKKSILIMIH
RSQRRVHITTFGFSTVSMECYATVSVNITAVTLH-
>DpleEOR30
MVIVGITVIFKHSILINCNDSIKQLLKILDEDYKAAELYGEREKDIILKSAMSGVKICRFWLV
SATLTCFMFPAKAIIEMGNLYMRGEFKLVPMFDFTYPNFIEVHKESTVAYTVLFLMCLSFDL
FSLSIYIGFDPLVPIFMLHTCGQLELISNKLVNVFSKDASRQDIVDDLKRINSLRNREISLEFI
FFFSGACLHFYMPCYYSNLLMEKSESLRNAIYFSGWESRRDIGIRKTILLMLTRTYVPLGIK
TVFYPICLDTFAEMVRQAYGIYNIMNAAWG-
>DpleOR1
MRVLKLGLKVIFCYPHIEGELQSKYVILGKIWVTLLSIATLSGQILYLYHKTGKIIFLKLGHT
YITTAMNSVVSDPDALTRYGILTFLVVEQLIQVSVAFEILETMSSKLEKAVYYLPWECMNV
SQRRVVLIMLMQAQRGGTVKALDMVNVGVQTMAAVGLKHKY-
>DpleEOR15
MAIFIFVNFIIYNSLLILNLVFTPRKIEYMIREVIFLFTEVAVTGKVLMIMFKRKKILSVFDILN
CEVFKGEDDISRRIVESHVSKYKTYWSLYAILSNFAYSSQVFVPIIVYFIFNTNIKLPICKYYF
LDEEYVRRYFVFLYLYQSLGIYGHMQYNVNIDALISGLLILAIAQVKVLKYELKNIKPDSPL
KDKRETERSQIFKLHKHLKHYEELLNYCSGIQDIVGLSMFVQFGIASIIICVVLSGLRFTASV
EALMFMVTYLFTMILQIYVPAWLGTQLNYESQELVFAAYSSQWIRRSEKFKRSLKLFMER
ANTNIVLTGWRIFPLSLDTFTSVITRNIS-
>DplePOR18
MIYPNRENIQTRFKVIFSVIAINGVMLYWFLLYLWKCICNHDMYNVSRNLTIGILVTLFFFK
SFYVTNKTHRFAQLLEKISNDLLKGNDMDDSYQEIYEKYIKLGKLGQTCWIFIPILLTSQFP
IYAGVSTIYESLKTDTGQKYMIHEMDLKYLEDKQYDSPIYELTFAYHLITCLTLSPNFAGFD
GSFCIATNHLRLKLKLLAHKVERAFKDAKNKWELHTMIKETIRDHQEALSFYEDLQEVYG
SWLFTVFAVTSLLISLNIYQIYLSERVDPKYAIFAISGIIHMFAPCYFASNLMKTSEELSLDLY
SVEWEVWAYPAVTKQLIFMIAKSQQTLILTGNGIVYFNMQLFISLPPTIAKVSVDRGLPLPR
SAWGVPPPAPFTFARCSAQPMTARPEPAKGFIYK-
>DpleOR54
MSLIDRFKTKLVDYKETFMDYRIESIILLLNFIPSMAGFSILNKKNSVIFWIVHFFLVLYVYV
LGSIMYQVNVADGPIDLIKSYVNLTLVILIINNSYWFIVKRATLTKLMENVIENDTQSHQLD
FLKPKHTKLLRTIKIIVYIFYAFNILDVIFVYIPRRIDVTNEYYSNVACVGMKPLTHFPKKQL
CQLVLFATEISICTVVLNYQALIFFLISHTAAMYRLLCEEIFALDDDNMDTATVKKRLVVLIG
RHTLTLTITKTIKDLYSMPIGINFGSNAICIILSFALPWEEWVNLSPVYIYCFLVFFLYCHLCQ
MLINASEQFEYAVYSCGWEKFDLKEQKTAYVMLLQAQKPVTLLAADIVPVNIYTFATTMQ
AMFKFITVFKF-
>DpleOR16
MADIKSEDLFLNRAKFVMKLLGVWIIYIVQVWGDLEAVSQASYLLFTQASLCFKVTVFQV
NVKSLRELLKRMNDKIFLPQSSKQNRILSLQAKRIKRLLLAFMISSQTTCGLWALKPLFDD
PGSRKFPFDMWMPVTPQSSPHYEFGYAFQLLTICMSAYMYFGVDSVALSMVIFACGQIDII

KDKILKINSIRKESGDEMSRRIVASDNNKKLIECIRQHQAVVLFKELLENTYHTYLLFQLVG
SVGLICMSALRILVVEWRSIQFFSIVTYLSVMISQLFVCCWCGHELTATSEDLHTVFYRSTW
YEQDVRFQRCLCFAMMRLGQPLVLRAGHYIPLSRQTFVSILRMSYSYFAVLNQTK-
>DplePOR10
MGIWLSSNNYVILRDLYKSLIMSTQYSFLLFEIFYVFVVWGDIDEMSEASYLLFTQTSVCY
KASVFIINKKNLIVLLVSMKAKIFEPQSSIHYGILYRNARVVKNLCAVFLMSAITTCSLWTIM
PLFDAAETRFFPFKIWMPVDPRDSPQYELGYIYQVMSIYISALLFVAVDNITLGMIMFGCA
QLEIVIDKIKQLKNASSKHNKERRKLICENNKLLDECIKQHQSVIRFIETLENTYHANIFFQL
TATVGIICIIGLRISIVEPRSVQFFSMLNYMVTMLSQLFLYCWCGSHLTTKSEELREWLYQCP
WYNQDPRFRKSVFIAMERMKRPIIFKAGHYIPLSRPTFVSVLRLSYSYFAVLNQAKNK-
>DpleOR63
MPFPYSQTFLTSSYSLLFLLPQQLGLPQTSTSLMLQMYLGDGDYFPLSSPYPYDTSVSPAYE
LTYMHQTLSIMIGAVLNISKDSLVTTLLAQTRCRLGLVGLALTTVCSGLLPEKWGRKSLQN
PEISVLMGNVLEEATAKDIKNCGKMLDTPAMKKAIVLEQKMLEVQKEKQKPVKYKEDII
MGLIVQNINFSLRFSLTALKIAGLWAPDGMQTRKKALYYCYTFVLLMFLLGTFIILQMIDLY
LIWGNLSLMTGTALVLFTNISQVTKIINLLARRKHIEFLIKDTNDGLEVMTSSRARELIKSC
DHETRNQQVIFLLVTFVTTVGWATITDKNGLPLPAWYPYDTSVSPAYELTYTHQTLSIMIVA
VLNISKDSLVTTLLAQTRCRLGLVGLALTTVCSDLVPEEDNTYSGGYSNKLFTAQQEMIAK
NRLRICVQQHQRALRAAKILQECFSVPTFAQFSVSLIIICVTAFQVLSSEELARRAYEMPWY
MCSVSVRRSIQIMMIRCHRIVKFTAGGFTTLSLATFTAVIKLSYSMLTLLQQVNEKI-
>DpleOR64
MCKRCEDLKIQSTKVGEAVYFSSWEEVVVTIPRVRTTVLLILLRAHKPIGLTGGGMYELSL
EAYGNVRVLEKKYIF-
>DpleOR1b
MVAEILKRFTSKLSSTFGPVLLHYYIFHIVTLCILLLECSKMDPDALTRYGILTFLVVEQLIQ
VSVAFEILETMSSKLEKAVYYLPWECMNVSQRRVVLIMLMQAQRGGTVKALDMVNVGV
QTMAAVGLKHKY-
>DpleOR58
MSLLFTHIKSSLKFTNVIFTVFGVWPPPGLNDLARRVYSLYSFLLLFLTIGVYIIIQTGDLVQ
VWGDLTLMTASVFMLFTNIAYAIKFIALSINAAVNLCCDVLVATLIAQCRCRLRLIAQSLRT
LCDDVDVDKKGRITADSEKVVSSRVRGIVMRHQLVLAQVEQLQQCFSVPILGQFAVASFII
CVTAYQMAFVTNLYRLISMASFEIAVTIQVYIYCVAGTNLTIDSDEVSRAAYECPWYEFPAS
IRRLLLVIMVRCKRATVITVAGIVEISLDTFMSLTSTRCVKTLTDLHSQI-
>DpleOR1a
MRVLKLGLKVIFCYPHIEGELQSKYVILGKIWVTLLSIATLSGQILYLYHKTGKIIFLKLGHT
YITTAMNSVVSDPDALTRYGILTFLVVEQLIQVSVAFEILETMSSKLEKAVYYLPWECMNV
SQRRVVLIMLMQAQRGGTVKALDMVNVGVQTMAAVGLKHKY-
>DmelOR
MQDQLDHELERIDKLPKLGLLWVEYSAYALGVNIAPRKRSSKYCRLTRILVLIVNLSIIYSL
VAFIMENYMISFETYVEAVLLTFQLSVGVVKMFHFQNKVESCSQLVFSTETGEVLKSLGLF
QLDLPRKKELLSSVSLILLNNWMIIDRQVMFFFKIVCMPVLYYCVRPYFQYIFDCYIKDKD
TCEMTLTYPAIVPYLQLGNYEFPSYVIRFFLLQSGPLWCFFAVFGFNSLFVVLTRYESGLIKV
LRFLVQNSTSDILVPKDQRVKYLQCCVRLFARISSHHNQIENLFKYIILVQCSVSSILICMLLY
KISTVLEVGWVWMGMIMVYFVTIALEITLYNVSAQKVESQSELLFHDWYNCSWYNESRE

FKFMIKMMLLFSRRTFVLSVGGFTSLSHKFLVQVFRLSANFFLLLRNMNNK-
>DmelOR1a
MSKLIEVFLGNLWTQRFTFARMGLDLQPDKKGNVLRSPLLYCIMCLTTSFELCTVCAFMV
QNRNQIVLCSEALMHGLQMVSSLLKMAIFLAKSHDLVDLIQQIQSPFTEEDLVGTEWRSQ
NQRGQLMAAIYFMMCAGTSVSFLLMPVALTMLKYHSTGEFAPVSSFRVLLPYDVTQPHV
YAMDCCLMVFVLSFFCCSTTGVDTLYGWCALGVSLQYRRLGQQLKRIPSCFNPSRSDFGL
SGIFVEHARLLKIVQHFNYSFMEIAFVEVVIICGLYCSVICQYIMPHTNQNFAFLGFFSLVVT
TQLCIYLFGAEQVRLEAERFSRLLYEVIPWQNLPPKHRKLFLFPIERAQRETVLGAYFFELG
RPLLVWIFRTAGSFTTLMNALYAKYETH-
>DmelOR2a
MEKQEDFKLNTHSAVYYHWRVWELTGLMRPPGVSSLLYVVYSITVNLVVTVLFPLSLLAR
LLFTTNMAGLCENLTITITDIVANLKFANVYMVRKQLHEIRSLLRLMDARARLVGDPEEIS
ALRKEVNIAQGTFRTFASIFVFGTTLSCVRVVVRPDRELLYPAWFGVDWMHSTRNYVLINI
YQLFGLIVQAIQNCASDSYPPAFLCLLTGHMRALELRVRRIGCRTEKSNKGQTYEAWREEV
YQELIECIRDLARVHRLREIIQRVLSVPCMAQFVCSAAVQCTVAMHFLYVADDHDHTAMII
SIVFFSAVTLEVFVICYFGDRMRTQSEALCDAFYDCNWIEQLPKFKRELLFTLARTQRPSLI
YAGNYIALSLETFEQVMRFTYSVFTLLLRAK-
>DmelOR7a
MAVSTRVATKQEVPESRRAFRNLFNCFYALGMQAPDGSRPTTSSTWQRIYACFSVVMYV
WQLLLVPTFFVISYRYMGGMEITQVLTSAQVAIDAVILPAKIVALAWNLPLLRRAEHHLAA
LDARCREQEEFQLILDAVRFCNYLVWFYQICYAIYSSSTFVCAFLLGQPPYALYLPGLDWQ
RSQMQFCIQAWIEFLIMNWTCLHQASDDVYAVIYLYVVRIQVQLLARRVEKLGTDDSGQV
EIYPDERRQEEHCAELQRCIVDHQTMLQLLDCISPVISRTIFVQFLITAAIMGTTMINIFIFAN
TNTKIASIIYLLAVTLQTAPCCYQATSLMLDNERLALAIFQCQWLGQSARFRKMLLYYLHR
AQQPITLTAMKLFPINLATYFSIAKFSFSLYTLIKGMNLGERFNRTN-
>DmelOR9a
MSDKVKGKKQEEKDQSLRVQILVYRCMGIDLWSPTMANDRPWLTFVTMGPLFLFMVPM
FLAAHEYITQVSLLSDTLGSTFASMLTLVKFLLFCYHRKEFVGLIYHIRAILAKEIEVWPDA
REIIEVENQSDQMLSLTYTRCFGLAGIFAALKPFVGIILSSIRGDEIHLELPHNGVYPYDLQV
VMFYVPTYLWNVMASYSAVTMALCVDSLLFFFTYNVCAIFKIAKHRMIHLPAVGGKEELE
GLVQVLLLHQKGLQIADHIADKYRPLIFLQFFLSALQICFIGFQVADLFPNPQSLYFIAFVGS
LLIALFIYSKCGENIKSASLDFGNGLYETNWTDFSPPTKRALLIAAMRAQRPCQMKGYFFE
ASMATFSTIVRSAVSYIMMLRSFNA-
>DmelOR10a
MSEWLRFLKRDQQLDVYFFAVPRLSLDIMGYWPGKTGDTWPWRSLIHFAILAIGVATELH
AGMCFLDRQQITLALETLCPAGTSAVTLLKMFLMLRFRQDLSIMWNRLRGLLFDPNWERP
EQRDIRLKHSAMAARINFWPLSAGFFTCTTYNLKPILIAMILYLQNRYEDFVWFTPFNMTM
PKVLLNYPFFPLTYIFIAYTGYVTIFMFGGCDGFYFEFCAHLSALFEVLQAEIESMFRPYTD
HLELSPVQLYILEQKMRSVIIRHNAIIDLTRFFRDRYTIITLAHFVSAAMVIGFSMVNLLTLG
NNGLGAMLYVAYTVAALSQLLVYCYGGTLVAESSTGLCRAMFSCPWQLFKPKQRRLVQL
LILRSQRPVSMAVPFFSPSLATFAAILQTSGSIIALVKSFQ-
>DmelOR13a
MFYSYPYKALSFPIQCVWLKLNGSWPLTESSRPWRSQSLLATAYIVWAWYVIASVGITISY
QTAFLLNNLSDIIITTENCCTTFMGVLNFVRLIHLRLNQRKFRQLIENFSYEIWIPNSSKNNV

AAECRRRMVTFSIMTSLLACLIIMYCVLPLVEIFFGPAFDAQNKPFPYKMIFPYDAQSSWIR
YVMTYIFTSYAGICVVTTLFAEDTILGFFITYTCGQFHLLHQRIAGLFAGSNAELAESIQLER
LKRIVEKHNNIISFAKRLEDFFNPILLANLMISSVLICMVGFQIVTGKNMFIGDYVKFIIYISS
ALSQLYVLCENGDALIKQSTLTAQILYECQWEGSDRIEIQSFTPTTKRIRNQIWFMILCSQQP
VRITAFKFSTLSLQSFTAILSTSISYFTLLRSVYFDDEKKLD-
>DmelOR19a
MDISKVDSTRALVNHWRIFRIMGIHPPGKRTFWGRHYTAYSMVWNVTFHICIWVSFSVNL
LQSNSLETFCESLCVTMPHTLYMLKLINVRRMRGQMISSHWLLRLLDKRLGCDDERQIIM
AGIERAEFIFRTIFRGLACTVVLGIIYISASSEPTLMYPTWIPWNWRDSTSAYLATAMLHTTA
LMANATLVLNLSSYPGTYLILVSVHTKALALRVSKLGYGAPLPAVRMQAILVGYIHDHQIIL
RLFKSLERSLSMTCFLQFFSTACAQCTICYFLLFGNVGIMRFMNMLFLLVILTTETLLLCYT
AELPCKEGESLLTAVYSCNWLSQSVNFRRLLLLMLARCQIPMILVSGVIVPISMKTFTVMIK
GAYTMLTLLNEIRKTSLE-
>DmelOR19b
MDISKVDSTRALVNHWRIFRIMGIHPPGKRTFWGRHYTAYSMVWNVTFHICIWVSFSVNL
LQSNSLETFCESLCVTMPHTLYMLKLINVRRMRGEMISSHWLLRLLDKRLGCADERQIIM
AGIERAEFIFRTIFRGLACTVVLGIIYISASSEPTLMYPTWIPWNWKDSTSAYLATAMLHTTA
LMANATLVLNLSSYPGTYLILVSVHTKALALRVSKLGYGAPLPAVRMQAILVGYIHDHQIIL
RLFKSLERSLSMTCFLQFFSTACAQCTICYFLLFGNVGIMRFMNMLFLLVILTTETLLLCYT
AELPCKEGESLLTAVYSCNWLSQSVNFRRLLLLMLARCQIPMILVSGVIVPISMKTFTVMIK
GAYTMLTLLNEIRKTSLE-
>DmelOR22
MLSKFFPHIKEKPLSERVKSRDAFIYLDRLMWSFGWTEPENKRWDLHYKLWSTFVTLLIFI
LLPISVSVEYIQRFKTFSAGEFLSSIQIGVDMYGSSFKSYLTMMGYKKRQEAKMSLDELDK
RCVCDEERTIVHRHVALGNFCYIFYHIAYTSFLISNFLSFIMKRIHAWRMYFPYVDPEKQFY
ISSIAEVILMGWAVFMALCTDVCPLISMLIARCHITLLKQRLRNLRSEPGRTEDEYLKELAD
CVRDHRLILDYVDALRSVFSGTIFVQFLLIGIVLGLSMINIMFFSTLSTGVAVVLFMSCVSM
QTFPFCYLCNMIMDDCQEMADSLFQSDWTSADRRYKSTLVYFLHNIQQPIILTAGGVFPIS
MQTNLNMVKLAFTVVTIVKQFNLAEKFQ-
>DmelOR22a
MLSKFFPHIKEKPLSERVKSRDAFIYLDRVMWSFGWTEPENKRWILPYKLWLAFVNIVMLI
LLPISISIEYLHRFKTFSAGEFLSSLEIGVNMYGSSFKCAFTLIGFKKRQEAKVLLDQLDKRC
LSDKERSTVHRYVAMGNFFDILYHIFYSTFVVMNFPYFLLERRHAWRMYFPYIDSDEQFYI
SSIAECFLMTEAIYMDLCTDVCPLISMLMARCHISLLKQRLRNLRSKPGRTEDEYLEELTEC
IRDHRLLLDYVDALRPVFSGTIFVQFLLIGTVLGLSMINLMFFSTFWTGVATCLFMFDVSM
ETFPFCYLCNMIIDDCQEMSNCLFQSDWTSADRRYKSTLVYFLHNLQQPITLTAGGVFPIS
MQTNLAMVKLAFSVVTVIKQFNLAERFQ-
>DmelOR22b
MLSQFFPHIKEKPLSERVKSRDAFVYLDRVMWSFGWTVPENKRWDLHYKLWSTFVTLLIF
ILLPISVSVEYIQRFKTFSAGEFLSSIQIGVNMYGSSFKSYLTMMGYKKRQEAKMSLDELD
KRCVCDEERTIVHRHVALGNFCYIFYHIAYTSFLISNFLSFIMKRIHAWRMYFPYVDPEKQF
YISSIAEVILRGWAVFMDLCTDVCPLISMVIARCHITLLKQRLRNLRSEPGRTEDEYLKELA
DCVRDHRLILDYVDALRSVFSGTIFVQFLLIGIVLGLSMINIMFFSTLSTGVAVVLFMSCVS
MQTFPFCYLCNMIMDDCQEMADSLFQSDWTSADRRYKSTLVYFLHNLQQPIILTAGGVFP

ISMQTNLNMVKLAFTVVTIVKQFNLAEKFQ-
>DmelOR22c
MTDSGQPAIADHFYRIPRISGLIVGLWPQRIRGGGGRPWHAHLLFVFAFAMVVVGAVGEV
SYGCVHLDNLVVALEAFCPGTTKAVCVLKLWVFFRSNRRWAELVQRLRAILWESRRQEA
QRMLVGLATTANRLSLLLLSSGTATNAAFTLQPLIMGLYRWIVQLPGQTELPFNIILPSFAVQ
PGVFPLTYVLLTASGACTVFAFSFVDGFFICSCLYICGAFRLVQQDIRRIFADLHGDSVDVFT
EEMNAEVRHRLAQVVERHNAIIDFCTDLTRQFTVIVLMHFLSAAFVLCSTILDIMLNTSSLS
GLTYICYIIAALTQLFLYCFGGNHVSESSAAVADVLYDMEWYKCDARTRKVILMILRRSQR
AKTIAVPFFTPSLPALRSILSTAGSYITLLKTFL-
>DmelOR24
MDSFLQVQKSTIALLGFDLFSENREMWKRPYRAMNVFSIAAIFPFILAAVLHNWKNVLLL
ADAMVALLITILGLFKFSMILYLRRDFKRLIDKFRLLMSNEAEQGEEYAEILNAANKQDQR
MCTLFRTCFLLAWALNSVLPLVRMGLSYWLAGHAEPELPFPCLFPWNIHIIRNYVLSFIWS
AFASTGVVLPAVSLDTIFCSFTSNLCAFFKIAQYKVVRFKGGSLKESQATLNKVFALYQTSL
DMCNDLNQCYQPIICAQFFISSLQLCMLGYLFSITFAQTEGVYYASFIATIIIQAYIYCYCGE
NLKTESASFEWAIYDSPWHESLGAGGASTSICRSLLISMMRAHRGFRITGYFFEANMEAFS
SIVRTAMSYITMLRSFS-
>DmelOR24a
MLPRFLTASYPMERHYFMVPKFALSLIGFYPEQKRTVLVKLWSFFNFFILTYGCYAEAYYGI
HYIPINIATALDALCPVASSILSLVKMVAIWWYQDELRSLIERVRFLTEQQKSKRKLGYKKR
FYTLATQLTFLLLCCGFCTSTSYSVRHLIDNILRRTHGKDWIYETPFKMMFPDLLLRLPLYPI
TYILVHWHGYITVVCFVGADGFFLGFCLYFTVLLLCLQDDVCDLLEVENIEKSPSEAEEAR
IVREMEKLVDRHNEVAELTERLSGVMVEITLAHFVTSSLIIGTSVVDILLFSGLGIIVYVVYT
CAVGVEIFLYCLGGSHIMEACSNLARSTFSSHWYGHSVRVQKMTLLMVARAQRVLTIKIPF
FSPSLETLTSILRFTGSLIALAKSVI-
>DmelOR30a
MELKSMDPVEMPIFGSTLKLMKFWSYLFVHNWRRYVAMTPYIIINCTQYVDIYLSTESLD
FIIRNVYLAVLFTNTVVRGVLLCVQRFSYERFINILKSFYIELLQSDDPIINILVKETTRLSVLI
SRINLLMGCCTCIGFVTYPIFGSERVLPYGMYLPTIDEYKYASPYYEIFFVIQAIMAPMGCC
MYIPYTNMVVTFTLFAILMCRVLQHKLRSLEKLKNEQVRGEIIWCIKYQLKLSGFVDSMN
ALNTHLHLVEFLCFGAMLCVLLFSLIIAQTIAQTVIVIAYMVMIFANSVVLYYVANELYFQS
FDIAIAAYESNWMDFDVDTQKTLKFLIMRSQKPLAILVGGTYPMNLKMLQSLLNAIYSFFT
LLRRVYG-
>DmelOR33a
MDSRRKVRSENLYKTYWLYWRLLGVEGDYPFRRLVDFTITSFITILFPVHLILGMYKKPQI
QVFRSLHFTSECLFCSYKFFCFRWKLKEIKTIEGLLQDLDSRVESEEERNYFNQNPSRVARM
LSKSYLVAAISAIITATVAGLFSTGRNLMYLGWFPYDFQATAAIYWISFSYQAIGSSLLILEN
LANDSYPPITFCVVSGHVRLLIMRLSRIGHDVKLSSSENTRKLIEGIQDHRKLMKIIRLLRST
LHLSQLGQFLSSGINISITLINILFFAENNFAMLYYAVFFAAMLIELFPSCYYGILMTMEFDKL
PYAIFSSNWLKMDKRYNRSLIILMQLTLVPVNIKAGGIVGIDMSAFFATVRMAYSFYTLALS
FRV-
>DmelOR33b
MDLKPRVIRSEDIYRTYWLYWHLLGLESNFFLNRLLDLVITIFVTIWYPIHLILGLFMERSL
GDVCKGLPITAACFFASFKFICFRFKLSEIKEIEILFKELDQRALSREECEFFNQNTRREANFI

WKSFIVAYGLSNISAIASVLFGGGHKLLYPAWFPYDVQATELIFWLSVTYQIAGVSLAILQN
LANDSYPPMTFCVVAGHVRLLAMRLSRIGQGPEETIYLTGKQLIESIEDHRKLMKIVELLR
STMNISQLGQFISSGVNISITLVNILFFADNNFAITYYGVYFLSMVLELFPCCYYGTLISVEM
NQLTYAIYSSNWMSMNRSYSRILLIFMQLTLAEVQIKAGGMIGIGMNAFFATVRLAYSFFT
LAMSLR-
>DmelOR33c
MVIIDSLSFYRPFWICMRLLVPTFFKDSSRPVQLYVVLLHILVTLWFPLHLLLHLLLLPSTAE
FFKNLTMSLTCVACSLKHVAHLYHLPQIVEIESLIEQLDTFIASEQEHRYYRDHVHCHARRF
TRCLYISFGMIYALFLFGVFVQVISGNWELLYPAYFPFDLESNRFLGAVALGYQVFSMLVEG
FQGLGNDTYTPLTLCLLAGHVHLWSIRMGQLGYFDDETVVNHQRLLDYIEQHKLLVRFH
NLVSRTISEVQLVQLGGCGATLCIIVSYMLFFVGDTISLVYYLVFFGVVCVQLFPSCYFASE
VAEELERLPYAIFSSRWYDQSRDHRFDLLIFTQLTLGNRGWIIKAGGLIELNLNAFFATLKM
AYSLFAVVVRAKGI-
>DmelOR35a
MVRYVPRFADGQKVKLAWPLAVFRLNHIFWPLDPSTGKWGRYLDKVLAVAMSLVFMQH
NDAELRYLRFEASNRNLDAFLTGMPTYLILVEAQFRSLHILLHFEKLQKFLEIFYANIYIDPR
KEPEMFRKVDGKMIINRLVSAMYGAVISLYLIAPVFSIINQSKDFLYSMIFPFDSDPLYIFVPL
LLTNVWVGIVIDTMMFGETNLLCELIVHLNGSYMLLKRDLQLAIEKILVARDRPHMAKQL
KVLITKTLRKNVALNQFGQQLEAQYTVRVFIMFAFAAGLLCALSFKAYTNPMANYIYAIW
FGAKTVELLSLGQIGSDLAFTTDSLSTMYYLTHWEQILQYSTNPSENLRLLKLINLAIEMNS
KPFYVTGLKYFRVSLQAGLKILQASFSYFTFLTSMQRRQMSN-
>DmelOR42a
MDLRRWFPTLYTQSKDSPVRSRDATLYLLRCVFLMGVRKPPAKFFVAYVLWSFALNFCST
FYQPIGFLTGYISHLSEFSPGEFLTSLQVAFNAWSCSTKVLIVWALVKRFDEANNLLDEMDR
RITDPGERLQIHRAVSLSNRIFFFFMAVYMVYATNTFLSAIFIGRPPYQNYYPFLDWRSSTLH
LALQAGLEYFAMAGACFQDVCVDCYPVNFVLVLRAHMSIFAERLRRLGTYPYESQEQKY
ERLVQCIQDHKVILRFVDCLRPVISGTIFVQFLVVGLVLGFTLINIVLFANLGSAIAALSFMA
AVLLETTPFCILCNYLTEDCYKLADALFQSNWIDEEKRYQKTLMYFLQKLQQPITFMAMN
VFPISVGTNISVTKFSFSVFTLVKQMNISEKLAKSEMEE-
>DmelOR42b
MVFELIRPAPLTEQKRSRDGCIYLYRAMKFIGWLPPKQGVLRYVYLTWTLMTFVWCTTYL
PLGFLGSYMTQIKSFSPGEFLTSLQVCINAYGSSVKVAITYSMLWRLIKAKNILDQLDLRCT
AMEEREKIHLVVARSNHAFLIFTFVYCGYAGSTYLSSVLSGRPPWQLYNPFIDWHDGTLKL
WVASTLEYMVMSGAVLQDQLSDSYPLIYTLILRAHLDMLRERIRRLRSDENLSEAESYEEL
VKCVMDHKLILRYCAIIKPVIQGTIFTQFLLIGLVLGFTLINVFFFSDIWTGIASFMFVITILLQ
TFPFCYTCNLIMEDCESLTHAIFQSNWVDASRRYKTTLLYFLQNVQQPIVFIAGGIFQISMSS
NISVAKFAFSVITITKQMNIADKFKTD-
>DmelOR43b
MFGHFKLVYPAPISEPIQSRDSNAYMMETLRNSGLNLKNDFGIGRKIWRVFSFTYNMVILP
VSFPINYVIHLAEFPPELLLQSLQLCLNTWCFALKFFTLIVYTHRLELANKHFDELDKYCV
KPAEKRKVRDMVATITRLYLTFVVVYVLYATSTLLDGLLHHRVPYNTYYPFINWRVDRTQ
MYIQSFLEYFTVGYAIYVATATDSYPVIYVAALRTHILLLKDRIIYLGDPSNEGSSDPSYMFK
SLVDCIKAHRTMLNFCDAIQPIISGTIFAQFIICGSILGIIMINMVLFADQSTRFGIVIYVMAVL
LQTFPLCFYCNAIVDDCKELAHALFHSAWWVQDKRYQRTVIQFLQKLQQPMTFTAMNIF

NINLATNINVAKFAFTVYAIASGMNLDQKLSIKE-
>DmelOR45a
MDASYFAVQRRALEIVGFDPSTPQLSLKHPIWAGILILSLISHNWPMVVYALQDLSDLTRLT
DNFAVFMQGSQSTFKFLVMMAKRRRIGSLIHRLHKLNQAASATPNHLEKIERENQLDRYV
ARSFRNAAYGVICASAIAPMLLGLWGYVETGVFTPTTPMEFNFWLDERKPHFYWPIYVW
GVLGVAAAAWLAIATDTLFSWLTHNVVIQFQLLELVLEEKDLNGGDSRLTGFVSRHRIAL
DLAKELSSIFGEIVFVKYMLSYLQLCMLAFRFSRSGWSAQVPFRATFLVAIIIQLSSYCYGG
EYIKQQSLAIAQAVYGQINWPEMTPKKRRLWQMVIMRAQRPAKIFGFMFVVDLPLLLWVI
RTAGSFLAMLRTFER-
>DmelOR45b
MYPRFLSRNYPLAKHLFFVTRYSFGLLGLRFGKEQSWLHLLWLVFNFVNLAHCCQAEFVF
GWSHLRTSPVDAMDAFCPLACSFTTLFKLGWMWWRRQEVADLMDRIRLLIGEQEKREDS
RRKVAQRSYYLMVTRCGMLVFTLGSITTGAFVLRSLWEMWVRRHQEFKFDMPFRMLFH
DFAHRMPWFPVFYLYSTWSGQVTVYAFAGTDGFFFGFTLYMAFLLQALRYDIQDALKPIR
DPSLRESKICCQRLADIVDRHNEIEKIVKEFSGIMAAPTFVHFVSASLVIATSVIDILLYSGYN
IIRYVVYTFTVSSAIFLYCYGGTEMSTESLSLGEAAYSSAWYTWDRETRRRVFLIILRAQRPI
TVRVPFFAPSLPVFTSVIKFTGSIVALAKTIL-
>DmelOR46a-A
MSKGVEIFYKGQKAFLNILSLWPQIERRWRIIHQVNYVHVIVFWVLLFDLLLVLHVMANL
SYMSEVVKAIFILATSAGHTTKLLSIKANNVQMEELFRRLDNEEFRPRGANEELIFAAACE
RSRKLRDFYGALSFAALSMILIPQFALDWSHLPLKTYNPLGENTGSPAYWLLYCYQCLALS
VSCITNIGFDSLCSSLFIFLKCQLDILAVRLDKIGRLITTSGGTVEQQLKENIRYHMTIVELSK
TVERLLCKPISVQIFCSVLVLTANFYAIAVLSDERLELFKYVTYQACMLIQIFILCYYAGEVT
QRSLDLPHELYKTSWVDWDYRSRRIALLFMQRLHSTLRIRTLNPSLGFDLMLFSSIVNCSY
SYFALLKRVNS-
>DmelOR46a-B
MVTEDFYKYQVWYFQILGVWQLPTWAADHQRRFQSMRFGFILVILFIMLLLFSFEMLNNI
SQVREILKVFFMFATEISCMAKLLHLKLKSRKLAGLVDAMLSPEFGVKSEQEMQMLELDR
VAVVRMRNSYGIMSLGAASLILIVPCFDNFGELPLAMLEVCSIEGWICYWSQYLFHSICLLP
TCVLNITYDSVAYSLLCFLKVQLQMLVLRLEKLGPVIEPQDNEKIAMELRECAAYYNRIVR
FKDLVELFIKGPGSVQLMCSVLVLVSNLYDMSTMSIANGDAIFMLKTCIYQLVMLWQIFIIC
YASNEVTVQSSRLCHSIYSSQWTGWNRANRRIVLLMMQRFNSPMLLSTFNPTFAFSLEAF
GSIVNCSYSYFALLKRVNS-
>DmelOR47b
MNDSGYQSNLSLLRVFLDEFRSVLRQESPGLIPRLAFYYVRAFLSLLCQYPNKKLASLPLY
RWINLFIMCNVMTIFWTMFVALPESKNVIEMGDDLVWISGMALVFTKIFYMHLRCDEIDE
LISDFEYYNRELRPHNIDEEVLGWQRLCYVIESGLYINCFCLVNFFSAAIFLQPLLGEGKLPF
HSVYPFQWHRLDLHPYTFWFLYIWQSLTSQHNLMSILMVDMVGISTFLQTALNLKLLCIEI
RKLGDMEVSDKRFHEEFCRVVRFHQHIIKLVGKANRAFNGAFNAQLMASFSLISISTFETM
AAAAVDPKMAAKFVLLMLVAFIQLSLWCVSGTLVYTQSVEVAQAAFDINDWHTKSPGIQ
RDISFVILRAQKPLMYVAEPFLPFTLGTYMLVLKNCYRLLALMQESM-
>DmelOR49a
MEKLRSYEDFIFMANMMFKTLGYDLFHTPKPWWRYLLVRGYFVLCTISNFYEASMVTTR
IIEWESLAGSPSKIMRQGLHFFYMLSSQLKFITFMINRKRLLQLSHRLKELYPHKEQNQRK

YEVNKYYLSCSTRNVLYVYYFVMVVMALEPLVQSCIMYLIGFGKADFTYKRIFPTRLTFD
SEKPLGYVLAYVIDFTYSQFIVNVSLGTDLWMMCVSSQISMHLGYLANMLASIRPSPETEQ
QDCDFLASIIKRHQLMIRLQKDVNYVFGLLLASNLFTTSCLLCCMAYYTVVEGFNWEGIS
YMMLFASVAAQFYVVSSHGQMLIDLSTNLAKAAFESKWYEGSLRYKKEILILMAQAQRP
LEISARGVIIISLDTFKILMTITYRFFAVIRQTVEK-
>DmelOR49b
MFEDIQLIYMNIKILRFWALLYDKNLRRYVCIGLASFHIFTQIVYMMSTNEGLTGIIRNSYM
LVLWINTVLRAYLLLADHDRYLALIQKLTEAYYDLLNLNDSYISEILDQVNKVGKLMARG
NLFFGMLTSMGFGLYPLSSSERVLPFGSKIPGLNEYESPYYEMWYIFQMLITPMGCCMYIP
YTSLIVGLIMFGIVRCKALQHRLRQVALKHPYGDRDPRELREEIIACIRYQQSIIEYMDHINE
LTTMMFLFELMAFSALLCALLFMLIIVSGTSQLIIVCMYINMILAQILALYWYANELREQNL
AVATAAYETEWFTFDVPLRKNILFMMMRAQRPAAILLGNIRPITLELFQNLLNTTYTFFTVL
KRVYG-
>DmelOR56a
MFKVKDLLLSPTTFEDPIFGTHLRYFQWYGYVASKDQNRPLLSLIRCTILTASIWLSCALML
ARVFRGYENLNDGATSYATAVQYFAVSIAMFNAYVQRDKVISLLRVAHSDIQNLMHEADN
REMELLVATQAYTRTITLLIWIPSVIAGLMAYSDCIYRSLFLPKSVFNVPAVRRGEEHPILLF
QLFPFGELCDNFVVGYLGPWYALGLGITAIPLWHTFITCLMKYVNLKLQILNKRVEEMDIT
RLNSKLVIGRLTASELTFWQMQLFKEFVKEQLRIRKFVQELQYLICVPVMADFIIFSVLICFL
FFALTVGVPSKMDYFFMFIYLFVMAGILWIYHWHATLIVECHDELSLAYFSCGWYNFEMP
LQKMLVFMMMHAQRPMKMRALLVDLNLRTFIDIGRGAYSYFNLLRSSHLY-
>DmelOR59a
MAEVRVDSLEFFKSHWTAWRYLGVAHFRVENWKNLYVFYSIVSNLLVTLCYPVHLGISLF
RNRTITEDILNLTTFATCTACSVKCLLYAYNIKDVLEMERLLRLLDERVVGPEQRSIYGQVR
VQLRNVLYVFIGIYMPCALFAELSFLFKEERGLMYPAWFPFDWLHSTRNYYIANAYQIVGI
SFQLLQNYVSDCFPAVVLCLISSHIKMLYNRFEEVGLDPARDAEKDLEACITDHKHILELFR
RIEAFISLPMLIQFTVTALNVCIGLAALVFFVSEPMARMYFIFYSLAMPLQIFPSCFFGTDNE
YWFGRLHYAAFSCNWHTQNRSFKRKMMLFVEQSLKKSTAVAGGMMRIHLDTFFSTLKG
AYSLFTIIIRMRK-
>DmelOR59b
MAVFKLIKPAPLTEKVQSRQGNIYLYRAMWLIGWIPPKEGVLRYVYLFWTCVPFAFGVFY
LPVGFIISYVQEFKNFTPGEFLTSLQVCINVYGASVKSTITYLFLWRLRKTEILLDSLDKRLA
NDSDRERIHNMVARCNYAFLIYSFIYCGYAGSTFLSYALSGRPPWSVYNPFIDWRDGMGSL
WIQAIFEYITMSFAVLQDQLSDTYPLMFTIMFRAHMEVLKDHVRSLRMDPERSEADNYQD
LVNCVLDHKTILKCCDMIRPMISRTIFVQFALIGSVLGLTLVNVFFFSNFWKGVASLLFVITI
LLQTFPFCYTCNMLIDDAQDLSNEIFQSNWVDAEPRYKATLVLFMHHVQQPIIFIAGGIFPIS
MNSNITVAKFAFSIITIVRQMNLAEQFQ-
>DmelOR59c
MTKFFFKRLQTAPLDQEVSSLDASDYYYRIAFFLGWTPPKGALLRWIYSLWTLTTMWLGI
VYLPLGLSLTYVKHFDRFTPTEFLTSLQVDINCIGNVIKSCVTYSQMWRFRRMNELISSLD
KRCVTTTQRRIFHKMVARVNLIVILFLSTYLGFCFLTLFTSVFAGKAPWQLYNPLVDWRKG
HWQLWIASILEYCVVSIGTMQELMSDTYAIVFISLFRCHLAILRDRIANLRQDPKLSEMEH
YEQMVACIQDHRTIIQCSQIIRPILSITIFAQFMLVGIDLGLAAISILFFPNTIWTIMANVSFIVA
ICTESFPCCMLCEHLIEDSVHVSNALFHSNWITADRSYKSAVLYFLHRAQQPIQFTAGSIFPI

SVQSNIAVAKFAFTIITIVNQMNLGEKFFSDRSNGDINP-
>DmelOR63a
MYSPEEAAELKRRNYRSIREMIRLSYTVGFNLLDPSRCGQVLRIWTIVLSVSSLASLYGHW
QMLARYIHDIPRIGETAGTALQFLTSIAKMWYFLFAHRQIYELLRKARCHELLQKCELFER
MSDLPVIKEIRQQVESTMNRYWASTRRQILIYLYSCICITTNYFINSFVINLYRYFTKPKGSY
DIMLPLPSLYPAWEHKGLEFPYYHIQMYLETCSLYICGMCAVSFDGVFIVLCLHSVGLMRS
LNQMVEQATSELVPPDRRVEYLRCCIYQYQRVANFATEVNNCFRHITFTQFLLSLFNWGLA
LFQMSVGLGNNSSITMIRMTMYLVAAGYQIVVYCYNGQRFATASEEIANAFYQVRWYGE
SREFRHLIRMMLMRTNRGFRLDVSWFMQMSLPTLMAMVRTSGQYFLLLQNVNQK-
>DmelOR64
MKLSETLKIDYFRVQLNAWRICGALDLSEGRYWSWSMLLCILVYLPTPMLLRGVYSFEDP
VENNFSLSLTVTSLSNLMKFCMYVAQLTKMVEVQSLIGQLDARVSGESQSERHRNMTEHL
LRMSKLFQITYAVVFIIAAVPFVFETELSLPMPMWFPFDWKNSMVAYIGALVFQEIGYVFQI
MQCFAADSFPPLVLYLISEQCQLLILRISEIGYGYKTLEENEQDLVNCIRDQNALYRLLDVT
KSLVSYPMMVQFMVIGINIAITLFVLIFYVETLYDRIYYLCFLLGITVQTYPLCYYGTMVQE
SFAELHYAVFCSNWVDQSASYRGHMLILAERTKRMQLLLAGNLVPIHLSTYVACWKGAYS
FFTLMADRDGLGS-
>DmelOR65a
MTELRSERKNGNWDRLFGPFFESWAVFKAPQAKSRHIIAYWTRDQLKALGFYMNSEQRR
LPRIVAWQYFVSIQLATALASLFYGISESIGDIVNLGRDLVFIITIIFICFRLVFFAQYAGELDVI
IDALEDIYHWSIKGPATKEVQETKRLHFLLFMALIITWFSFLILFMLIKISTPFWIESQTLPFH
VSWPFQLHDPSKHPIAYIIIFVSQSTTMLYFLIWLGVVENMGVSLFFELTSALRVLCIELRNL
QELCLGDEDMLYRELCRMTKFHQQIILLTDRCNHIFNGAFIMQMLINFLLVSLSLFEVLAA
KKNPQVAVEYMIIMLMTLGHLSFWSKFGDMFSKESEQVALAVYEAYDPNVGSKSIHRQFC
FFIQRAQKPLIMKASPFPPFNLENYMFILKQCYSILTILANTLE-
>DmelOR65b
MDIQRFLKFYKVGWKTYRDPLMEASHSSIYYWREQMKAMALFTTTEERLLPYRSKWHT
LVYIQMVIFFASMSFGLTESMGDHVQMGRDLAFILGAFFIIFKTYYFCWYGDELDQVISDL
DALHPWAQKGPNPVEYQTGKRWYFVMAFFLATSWSFFLCILLLLLITSPMWVHQQNLPF
HAAFPFQWHEKSLHPISHAIIYLFQSYFAVYCLTWLLCIEGLSICIYAEITFGIEVLCLELRQI
HRHNYGLQELRMETNRLVKLHQKIVEILDRTNDVFHGTLIMQMGVNFSLVSLSVLEAVEA
RKDPKVVAQFAVLMLLALGHLSMWSYCGDQLSQKSLQISEAAYEAYDPTKGSKDVYRDL
CVIIRRGQDPLIMRASPFPSFNLINYSAILNQCYGILTFLLKTLD-
>DmelOR65c
MDIRGNVHRFVKFYIDGWKHFRDPTMESSYSAVYYWREQMKAMFLYTTSKERQMPYRS
SWHTLVIIQATVCFLTMCYGVTESLGDKVQMGRDIAFIIGFFYIAFKIYYFQWYGDELDEV
VEALETFHPWAQKGPGAVDYRTAKRWYFTLAFFLASSWLVFLCIFILLLITSPLWVHQQILP
LHAAFPFQWHEKSIHPISHAFIYLFQTWNVMYFLTWLVCIEGLSVSIYVEITFAIEVLCLELR
HLHQRCHGYEQLRLETNRLVQFHQKIVHILDHTNKVFHGTLIMQMGVNFFLVSLSVLEA
MEARKDPKVVAQFAVLMLLALGHLSMWSYFGDLLSQKSLTISEAAYEAYDPIKGSKDVY
RDLCLIIRRGQEPLIMRASPFPSFNFINYSAILNQCYGILTFLLKTLD-
>DmelOR67a
MDNVAEMPEEKYVEVDDFLRLAVKFYNTLGIDPYETGRKRTIWFQIYFALNMFNMVFSFY
AEVATLVDRLRDNENFLESCILLSYVSFVVMGLSKIGAVMKKKPKMTALVRQLETCFPSPS

AKVQEEYAVKSWLKRCHIYTKGFGGLFMIMYFAHALIPLFIYFIQRVLLHYPDAKQIMPFY
QLEPWEFRDSWLFYPSYFHQSSAGYTATCGSIAGDLMIFAVVLQVIMHYERLAKVLREFKI
QAHNAPNGAKEDIRKLQSLVANHIDILRLTDLMNEVFGIPLLLNFIASALLVCLVGVQLTIA
LSPEYFCKQMLFLISVLLEVYLLCSFSQRLIDASENVGHAAYDMDWLGSDKRFKKILIFIS
MRSQKPVCLKATVVLDLSMPTMSIFLGMSYKFFCAVRTMYQ-
>DmelOR67c
METAKDNTARTFMELMRVPVQFYRTIGEDIYAHRSTNPLKSLLFKIYLYAGFINFNLLVIGE
LVFFYNSIQDFETIRLAIAVAPCIGFSLVADFKQAAMIRGKKTLIMLLDDLENMHPKTLAKQ
MEYKLPDFEKTMKRVINIFTFLCLAYTTTFSFYPAIKASVKFNFLGYDTFDRNFGFLIWFPF
DATRNNLIYWIMYWDIAHGAYLAGIAFLCADLLLVVVITQICMHFNYISMRLEDHPCNSN
EDKENIEFLIGIIRYHDKCLKLCEHVNDLYSFSLLLNFLMASMQICFIAFQVTESTVEVIIIYC
IFLMTSMVQVFMVCYYGDTLIAASLKVGDAAYNQKWFQCSKSYCTMLKLLIMRSQKPAS
IRPPTFPPISLVTYMKVISMSYQFFALLRTTYSNN-
>DmelOR67d
MLKMAKVEPVERYCKVIRMIRFCVGFCGNDVADPNFRMWWLTYAVMAAIAFFFACTGY
TIYVGVVINGDLTIILQALAMVGSAVQGLTKLLVTANNASHMREVQNTYEDIYREYGSKG
DEYAKCLEKRIRITWTLLIGFMLVYIILLGLVITFPIFYLLILHQKVLVMQFLIPFLDHTTDGG
HLILTAAHVILITFGGFGNYGGDMYLFLFVTHVPLIKDIFCVKLTEFNELVMKRNDFPKVR
AMLCDLLVWHQLYTRMLQTTKKIYSIVLFVQLSTTCVGLLCTISCIFMKAWPAAPLYLLYA
AITLYTFCGLGTLVENSNEDFLSVIYTNCLWYELPVKEEKLIIMMLAKAQNEVVLTAADM
APLSMNTALQLTKGIYSFSMMLMNYLG-
>DmelOR69
MQLHDHMKYIDLGCKMACIPRYQWKGRPTERQLNASEQRIVFLLGTICQIFQITGVLIYW
YCNGRLATETGTFVAQLSEMCSSFCLTFVGFCNVYAISTNRNQIETLLEELHQIYPRYRKNH
YRCQHYFDMAMTIMRIEFLFYMILYVYYNSAPLWVLLWEHLHEEYDLSFKTQTNTWFPW
KVHGSALGFGMAVLSITVGSFVGVGFSIVTQNLICLLTFQLKLHYDGISSQLVSLDCRRPGA
HKELSILIVHHSRILQLGDQVNNIMNFVFGSSLVGATIAICMSSVSIMLLDLASAFKYASGLV
AFVLYNFVICYMGTEVTLAMQLQDFMRYPDLVCQAAQLPRYTWNGRRSLEVKRNLAKRI
IFWLGAVNLVYHNIGCVMYGYFVDGRTKDPIEYLAELASVASMLGFTIVGTLNLWKMLSL
KTHFENLLNEFEELFQLIKHRAYRIHHYQEKYTRHIRNTFIFHTSAVVYYNSLPILLMIREHL
SNSQQLGYRIQSNTWYPWQVQGSIPGFFAAVACQIFSCQTNMCVNMFIQFLINFFGIQLEIH
FDGLARQLETIDARNPHAKDQLKYLIVYHTKLLNLADRVNRSFNFTFLISLSVSMISNCFL
AFSMTMFDFGTSLKHLLGLLLFITYNFSMCRSGTHLILTSGKVLPAAFYNNWYEGDLVYR
RMLLILMMRATKPYMWKTYKLAPVSITTYMATLKFSYQMFTCVRSLK-
>DmelOR71a-B
MDYDRIRPVRFLTGVLKWWRLWPRKESVSTPDWTNWQAYALHVPFTFLFVLLLWLEAIK
SRDIQHTADVLLICLTTTALGGKVINIWKYAHVAQGILSEWSTWDLFELRSKQEVDMWRF
EHRRFNRVFMFYCLCSAGVIPFIVIQPLFDIPNRLPFWMWTPFDWQQPVLFWYAFIYQATTI
PIACACNVTMDAVNWYLMLHLSLCLRMLGQRLSKLQHDDKDLREKFLELIHLHQRLKQ
QALSIEIFISKSTFTQILVSSLIICFTIYSMQMSPVLQDLPGFAAMMQYLVAMIMQVMLPTIY
GNAVIDSANMLTDSMYNSDWPDMNCRMRRLVLMFMVYLNRPVTLKAGGFFHIGLPLFT
KTMNQAYSLLALLLNMNQ-
>DmelOR74a
MSFHRYRPRLPGGELAPMPWPVSLYRVLNHVAWPLEAESGRWTVFLDRLMIFLGFLVFCE

HNEVDFHYLIANRQDMDNMLTGLPTYLILVEMQIRCFQLAWHKDRFRALLQRFYAEIYVS
EEMEPHLFASIQRQMLATRVNSTVYLLALLNFFLVPVTNVIYHRREMLYKQVYPFDNTQL
HFFIPLLVLNFWVGFIITSMLFGELNVMGELMMHLNARYIQLGQDLRRSAQMLLKKSSSL
NVAIAYRLNLTHILRRNAALRDFGQRVEKEFTLRIFVMFAFSAGLLCALFFKAFTNPWGNV
AYIVWFLAKFMELLALGMLGSILLKTTDELGMMYYTADWEQVIHQSDNVGENVKLMKL
VTLAIQLNSRPFFITGLNYFRVSLTAVLKIIQGAFSYFTFLNSMR-
>DmelOR82a
MGRLFQLQEYCLRAMGHKDDMDSTDSTALSLKHISSLIFVISAQYPLISYVAYNRNDMEK
VTACLSVVFTNMLTVIKISTFLANRKDFWEMIHRFRKMHEQSASHIPRYREGLDYVAEAN
KLASFLGRAYCVSCGLTGLYFMLGPIVKIGVCRWHGTTCDKELPMPMKFPFNDLESPGYE
VCFLYTVLVTVVVVAYASAVDGLFISFAINLRAHFQTLQRQIENWEFPSSEPDTQIRLKSIVE
YHVLLLSLSRKLRSIYTPTVMGQFVITSLQVGVIIYQLVTNMDSVMDLLLYASFFGSIMLQL
FIYCYGGEIIKAESLQVDTAVRLSNWHLASPKTRTSLSLIILQSQKEVLIRAGFFVASLANFV
GICRTALSLITLIKSIE-
>DmelOR83a
MKSTFKEERIKDDSKRRDLFVFVRQTMCIAAMYPFGYYVNGSGVLAVLVRFCDLTYELFN
YFVSVHIAGLYICTIYINYGQGDLDFFVNCLIQTIIYLWTIAMKLYFRRFRPGLLNTILSNIND
EYETRSAVGFSFVTMAGSYRMSKLWIKTYVYCCYIGTIFWLALPIAYRDRSLPLACWYPF
DYTQPGVYEVVFLLQAMGQIQVAASFASSSGLHMVLCVLISGQYDVLFCSLKNVLASSYV
LMGANMTELNQLQAEQSAADVEPGQYAYSVEEETPLQELLKVGSSMDFSSAFRLSFVRCI
QHHRYIVAALKKIESFYSPIWFVKIGEVTFLMCLVAFVSTKSTAANSFMRMVSLGQYLLLV
LYELFIICYFADIVFQNSQRCGEALWRSPWQRHLKDVRSDYMFFMLNSRRQFQLTAGKISN
LNVDRFRGTITTAFSFLTLLQKMDARE-
>DmelOR83bORco
MTTSMQPSKYTGLVADLMPNIRAMKYSGLFMHNFTGGSAFMKKVYSSVHLVFLLMQFTF
ILVNMALNAEEVNELSGNTITTLFFTHCITKFIYLAVNQKNFYRTLNIWNQVNTHPLFAESD
ARYHSIALAKMRKLFFLVMLTIVASATAWTTITFFGDSVKMVVDHETNSSIPVEIPRLPIKSF
YPWNASHGMFYMISFAFQIYYVLFSMIHSNLCDVMFCSWLIFACEQLQHLKGIMKPLMEL
SASLDTYRPNSAALFRSLSANSKSELIHNEEKDPGTDMDMSGIYSSKADWGAQFRAPSTL
QSFGGNGGGGNGLVNGANPNGLTKKQEMMVRSAIKYWVERHKHVVRLVAAIGDTYGA
ALLLHMLTSTIKLTLLAYQATKINGVNVYAFTVVGYLGYALAQVFHFCIFGNRLIEESSSV
MEAAYSCHWYDGSEEAKTFVQIVCQQCQKAMSISGAKFFTVSLDLFASVLGAVVTYFMV
LVQLK-
>DmelOR83c
MSTSESPSSRFRELSKYINSLTNLLGVDFLSPKLKFNYRTWTTIFAIANYTGFTVFTILNNGG
DWRVGLKASLMTGGLFHGLGKFLTCLLKHQDMRRLVLYSQSIYDEYETRGDSYHRTLNS
NIDRLLGIMKIIRNGYVFAFCLMELLPLAMLMYDGTRVTAMQYLIPGLPLENNYCYVVTY
MIQTVTMLVQGVGFYSGDLFVFLGLTQILTFADMLQVKVKELNDALEQKAEYRALVRVG
ASIDGAENRQRLLLDVIRWHQLFTDYCRAINALYYELIATQVLSMALAMMLSFCINLSSFH
MPSAIFFVVSAYSMSIYCILGTILEFAYDQVYESICNVTWYELSGEQRKLFGFLLRESQYPH
NIQILGVMSLSVRTALQIVKLIYSVSMMMMNRA-
>DmelOR85a
MIFKYIQEPVLGSLFRSRDSLIYLNRSIDQMGWRLPPRTKPYWWLYYIWTLVVIVLVFIFIP
YGLIMTGIKEFKNFTTTDLFTYVQVPVNTNASIMKGIIVLFMRRRFSRAQKMMDAMDIRC

TKMEEKVQVHRAAALCNRVVVIYHCIYFGYLSMALTGALVIGKTPFCLYNPLVNPDDHFY
LATAIESVTMAGIILANLILDVYPIIYVVVLRIHMELLSERIKTLRTDVEKGDDQHYAELVEC
VKDHKLIVEYGNTLRPMISATMFIQLLSVGLLLGLAAVSMQFYNTVMERVVSGVYTIAILS
QTFPFCYVCEQLSSDCESLTNTLFHSKWIGAERRYRTTMLYFIHNVQQSILFTAGGIFPICLN
TNIKMAKFAFSVVTIVNEMDLAEKLRRE-
>DmelOR85b
MEKLMKYASFFYTAVGIRPYTNGEESKMNKLIFHIVFWSNVINLSFVGLFESIYVYSAFMD
NKFLEAVTALSYIGFVTVGMSKMFFIRWKKTAITELINELKEIYPNGLIREERYNLPMYLGT
CSRISLIYSLLYSVLIWTFNLFCVMEYWVYDKWLNIRVVGKQLPYLMYIPWKWQDNWSY
YPLLFSQNFAGYTSAAGQISTDVLLCAVATQLVMHFDFLSNSMERHELSGDWKKDSRFLV
DIVRYHERILRLSDAVNDIFGIPLLLNFMVSSFVICFVGFQMTVGVPPDIVVKLFLFLVSSMS
QVYLICHYGQLVADASYGFSVATYNQKWYKADVRYKRALVIIIARSQKVTFLKATIFLDIT
RSTMTDLLQISYKFFALLRTMYTQ-
>DmelOR85c
MKFMKYAVFFYTSVGIEPYTIDSRSKKASLWSHLLFWANVINLSVIVFGEILYLGVAYSDGK
FIDAVTVLSYIGFVIVGMSKMFFIWWKKTDLSDLVKELEHIYPNGKAEEEMYRLDRYLRS
CSRISITYALLYSVLIWTFNLFSIMQFLVYEKLLKIRVVGQTLPYLMYFPWNWHENWTYYV
LLFCQNFAGHTSASGQISTDLLLCAVATQVVMHFDYLARVVEKQVLDRDWSENSRFLAKT
VQYHQRILRLMDVLNDIFGIPLLLNFMVSTFVICFVGFQMTVGVPPDIMIKLFLFLFSSLSQ
VYLICHYGQLIADASSSLSISAYKQNWQNADIRYRRALVFFIARPQRTTYLKATIFMNITRA
TMTDLLQVSYKFFALLRTMYIK-
>DmelOR85d
MLTKKDTQSAKEQEKLKAIPLHSFLKYANVFYLSIGMMAYDHKYSQKWKEVLLHWTFIA
QMVNLNTVLISELIYVFLAIGKGSNFLEATMNLSFIGFVIVGDFKIWNISRQRKRLTQVVSR
LEELHPQGLAQQEPYNIGHHLSGYSRYSKFYFGMHMVLIWTYNLYWAVYYLVCDFWLG
MRQFERMLPYYCWVPWDWSTGYSYYFMYISQNIGGQACLSGQLAADMLMCALVTLVV
MHFIRLSAHIESHVAGIGSFQHDLEFLQATVAYHQSLIHLCQDINEIFGVSLLSNFVSSSFIICF
VGFQMTIGSKIDNLVMLVLFLFCAMVQVFMIATHAQRLVDASEQIGQAVYNHDWFRADL
RYRKMLILIIKRAQQPSRLKATMFLNISLVTVSDLLQLSYKFFALLRTMYVN-
>DmelOR85f
MEPVQYSYEDFARLPTTVFWIMGYDMLGVPKTRSRRILYWIYRFLCLASHGVCVGVMVF
RMVEAKTIDNVSLIMRYATLVTYIINSDTKFATVLQRSAIQSLNSKLAELYPKTTLDRIYHRV
NDHYWTKSFVYLVIIYIGSSIMVVIGPIITSIIAYFTHNVFTYMHCYPYFLYDPEKDPVWIYI
GIYALEWLHSTQMVISNIGADIWLLYFQVQINLHFRGIIRSLADHKPSVKHDQEDRKFIAKI
VDKQVHLVSLQNDLNGIFGKSLLLSLLTTAAVICTVAVYTLIQGPTLEGFTYVIFIGTSVMQ
VYLVCYYGQQVLDLSGEVAHAVYNHDFHDASIAYKRYLLIIIIRAQQPVELNAMGYLSISL
DTFKQLMSVSYRVITMLMQMIQ-
>DmelOR87
MTIEDIGLVGINVRMWRHLAVLYPTPGSSWRKFAFVLPVTAMNLMQFVYLLRMWGDLPA
FILNMFFFSAIFNALMRTWLVIIKRRQFEEFLGQLATLFHSILDSTDEWGRGILRRAEREAR
NLAILNLSASFLDIVGALVSPLFREERAHPFGVALPGVSMTSSPVYEVIYLAQLPTPLLLSM
MYMPFVSLFAGLAIFGKAMLQILVHRLGQIGGEEQSEEERFQRLASCIAYHTQVMRYVWQ
LNKLVANIVAVEAIIFGSIICSLLFCLNIITSPTQVISIVMYILTMLYVLFTYYNRANEICLENN
RVAEAVYNVPWYEAGTRFRKTLLIFLMQTQHPMEIRVGNVYPMTLAMFQSLLNASYSYF

TMLRGVTGK-
>DmelOR88a
MKPTEIKKPYRMEEFLRPQMFQEVAQMVHFQWRRNPVDNSMVNASMVPFCLSAFLNVL
FFGCNGWDIIGHFWLGHPANQNPPVLSITIYFSIRGLMLYLKRKEIVEFVNDLDRECPRDLV
SQLDMQMDETYRNFWQRYRFIRIYSHLGGPMFCVVPLALFLLTHEGKDTPVAQHEQLLG
GWLPCGVRKDPNFYLLVWSFDLMCTTCGVSFFVTFDNLFNVMQGHLVMHLGHLARQFS
AIDPRQSLTDEKRFFVDLRLLVQRQQLLNGLCRKYNDIFKVAFLVSNFVGAGSLCFYLFML
SETSDVLIIAQYILPTLVLVGFTFEICLRGTQLEKASEGLESSLRSQEWYLGSRRYRKFYLL
WTQYCQRTQQLGAFGLIQVNMVHFTEIMQLAYRLFTFLKSH-
>DmelOR92a
MLFRKRKPKSDDEVITFDELTRFPMTFYKTIGEDLYSDRDPNVIRRYLLRFYLVLGFLNFN
AYVVGEIAYFIVHIMSTTTLLEATAVAPCIGFSFMADFKQFGLTVNRKRLVRLLDDLKEIFPL
DLEAQRKYNVSFYRKHMNRVMTLFTILCMTYTSSFSFYPAIKSTIKYYLMGSEIFERNYGF
HILFPYDAETDLTVYWFSYWGLAHCAYVAGVSYVCVDLLLIATITQLTMHFNFIANDLEA
YEGGDHTDEENIKYLHNLVVYHARALDLSEEVNNIFSFLILWNFIAASLVICFAGFQITASN
VEDIVLYFIFFSASLVQVFVVCYYGDEMISSSSRIGHSAFNQNWLPCSTKYKRILQFIIARSQ
KPASIRPPTFPPISFNTFMKVISMSYQFFALLRTTYYG-
>DmelOR94a
MDKHKDRIESMRLILQVMQLFGLWPWSLKSEEEWTFTGFVKRNYRFLLHLPITFTFIGLM
WLEAFISSNLEQAGQVLYMSITEMALVVKILSIWHYRTEAWRLMYELQHAPDYQLHNQE
EVDFWRREQRFFKWFFYIYILISLGVVYSGCTGVLFLEGYELPFAYYVPFEWQNERRYWF
AYGYDMAGMTLTCISNITLDTLGCYFLFHISLLYRLLGLRLRETKNMKNDTIFGQQLRAIFI
MHQRIRSLTLTCQRIVSPYILSQIILSALIICFSGYRLQHVGIRDNPGQFISMLQFVSVMILQIY
LPCYYGNEITVYANQLTNEVYHTNWLECRPPIRKLLNAYMEHLKKPVTIRAGNFFAVGLPI
FVKTINNAYSFLALLLNVSN-
>DmelOR94b
MESTNRLSAIQTLLVIQRWIGLLKWENEGEDGVLTWLKRIYPFVLHLPLTFTYIALMWYEA
ITSSDFEEAGQVLYMSITELALVTKLLNIWYRRHEAASLIHELQHDPAFNLRNSEEIKFWQQ
NQRNFKRIFYWYIWGSLFVAVMGYISVFFQEDYELPFGYYVPFEWRTRERYFYAWGYNV
VAMTLCCLSNILLDTLGCYFMFHIASLFRLLGMRLEALKNAAEEKARPELRRIFQLHTKVR
RLTRECEVLVSPYVLSQVVFSAFIICFSAYRLVHMGFKQRPGLFVTTVQFVAVMIVQIFLPC
YYGNELTFHANALTNSVFGTNWLEYSVGTRKLLNCYMEFLKRPVKVRAGVFFEIGLPIFV
KTINNAYSFFALLLKISK-
>DmelOR98a
MLFNYLRKPNPTNLLTSPDSFRYFEYGMFCMGWHTPATHKIIYYITSCLIFAWCAVYLPIGII
ISFKTDINTFTPNELLTVMQLFFNSVGMPFKVLFFNLYISGFYKAKKLLSEMDKRCTTLKER
VEVHQGVVRCNKAYLIYQFIYTAYTISTFLSAALSGKLPWRIYNPFVDFRESRSSFWKAAL
NETALMLFAVTQTLMSDIYPLLYGLILRVHLKLLRLRVESLCTDSGKSDAENEQDLIKCIKD
HNLIIDYAAAIRPAVTRTIFVQFLLIGICLGLSMINLLFFADIWTGLATVAYINGLMVQTFPFC
FVCDLLKKDCELLVSAIFHSNWINSSRSYKSSLRYFLKNAQKSIAFTAGSIFPISTGSNIKVA
KLAFSVVTFVNQLNIADRLTKN-
>DmelOR104
MASLQFHGNVDADIRYDISLDPARESNLFRLLMGLQLANGTKPSPRLPKWWPKRLEMIGK
VLPKAYCSMVIFTSLHLGVLFTKTTLDVLPTGELQAITDALTMTIIYFFTGYGTIYWCLRSR

RLLAYMEHMNREYRHHSLAGVTFVSSHAAFRMSRNFTVVWIMSCLLGVISWGVSPLML
GIRMLPLQCWYPFDALGPGTYTAVYATQLFGQIMVGMTFGFGGSLFVTLSLLLLGQFDVL
YCSLKNLDAHTKLLGGESVNGLSSLQEELLLGDSKRELNQYVLLQEHPTDLLRLSAGRKC
PDQGNAFHNALVECIRLHRFILHCSQELENLFSPYCLVKSLQITFQLCLLVFVGVSGTREVL
RIVNQLQYLGLTIFELLMFTYCGELLSRHSIRSGDAFWRGAWWKHAHFIRQDILIFLVNSR
RAVHVTAGKFYVMDVNRLRSVITQAFSFLTLLQKLAAKKTESEL-
>HarmOR1
MKILSDGSDLEGVEKVEDIFYINLARKSMWILDSWPKTPNESVTYRYFVLALNVATLVGG
AVYLRNNTGVLSSFELGHTYITVFMNCITCSRCIMILSREYNEVMLSFVNKIHLFHHRHKS
EYAYKTHIFIHKISHFYTVYLLGLALNGLLLFNMIPFYNCYSRGMFRDVIPANATYDHSVFY
SVPFDYTTKFKGYIAMTSFNCFISYTCTSYFCVVDLTVSLVIFHLWGHMRLLTYHLANFKK
PASVLESNENTDAIKDHSYTQEELKEVFGKLREYIRHHNLILNFSAEMSNAFGPALLAYMV
FHQVSGCILLLECSQLDMKTLVRYGPLTVVIFQQLIQISVIFELLGSSNDKLIDAVYLVPWEY
MDTKNRKLVFVMLRQSQRSIDLKMMSMLTVGVQTMTAILKTSFSYFVMLKTVAEEEQ-
>HarmOR2
MYAGNAVTGITGPMDYKYMKVLRFVLRIISGWPGKALGEKTLRIEGMGHAYYNTILSLVY
LALGIAYLKKNFHRFDFLELGQLYIVLLMNMLSTSRAFTLCLSQKYREVAKIFIQKIHLFYF
KEKSDFAMKIHITVHKISFISAVYLSVLLFIAACMFNLIPMYNNYSAGRFASFDNLENTTYE
QAISCLYPWNFETNFNGYLAATLSGWYGTILCGSSVSMFDLFLCLMIFNLWGHFKILIYNL
EHFPRPASEVVDAEGEERSGRTVGSEMYSQSELEEVAVLLRDCIQYHMLIYNFTNNMSDA
FGMALFIYYSFHQITGCLLLLECSQMTAAALTRYLPLTIIMFGELVLLSIIFETIGTMSEKLKD
AVYKVPWEYMDTKNRRTVLIFLIKVQEPIHVKAGGLVDVGVTTMASILKTSFSYFAFLRTF
-
>HarmCOR3
MGLRQFLFENEAVEGINSASDYLYIKVLRFMLLIVNSWPRKEIGEPESPKFSAFVKYFYLVV
TVSGSAGFILYLVKHNSELTFLETGHMYIVLLMSLIDVSRVATLTMSTTYREVARDFLTKIHL
FYYKDRSKQAMETHRAVHKIAHLFTLWLVSQMLSGLSLFNIIPMYSNYAAGRFSGEVSKN
STFEHSMYYPYPFNTSTDIRGYSIACIIHWIISYLCSTWFCMFDLFLSLMVFHLWGHFKILN
YTLNDFPRPSSEVEAAKYSDEELVEVAARLKDCILYHREIILFTDRMSNVFGPMLFLYYMF
HQASGCLLLLECSQMTAQALIRYVPLTIILTQQLIQLSVIFELVGSESDKLKHAVYGVPWEC
MDVKNRRSVVIFLANTQEPVHVKAMGVANVGVTSMAAILKTSMSYFTFLRSL-
>HarmOR29
MGYQQIDCFDIHLKILRILGVWPHDNPSIYYIYFSRIFVFTFTVLYVVIYTMNFYFLPQQLE
VFADELIFYFTNVGALSKALAFIFLRDKVKKMLFMLESEIFQSDDPEEIKLIKEGKEKSNFY
WKITAGLSVSANTVNVCLPLLVHIIFSVELEFPVCRYSFIPEKYEAMFAYPAYFYQSIGITTH
MLYNVNIDTFLLGVMFLAMTQLDILDRKLRKVTDVCINPDAARGSVDKFIDDQNAVLEII
KCIKHYDAICEYCKLIQDAFSEILFVLFSSGSCKICMCLFRFTMPATTGYFVFLYTYVTVMT
LQVMVPCWFGSRLMDKSSQITIAAYDCDWTPRCRRFKSNLRLLVERANRPITIIGGKMFLL
SLGTFTAIMNSSYSFFTLMRHMQSR-
>HarmOR20
MDEELEFKPFHETYRLITFSLCIAMIYPNPRTEKWRLFSIPILIATVAPVAIMIFLDMYKCWK
NGDIVNIIRHSTVVGPFLGGFFKMILMYHKRVQAKQILDEFDRDHLMFNTVAETYKDIAR
ASIRNCQIYSERLWACLVTTCVMTFPVMAIVLNIYNFMFKSEPTKYMIHDLEKPFSKEPEE
RFESPYFELLFVYMFYAAILYVVNFTGYDGFFGLCVNHARLKMELYCKALEEAMMADRE

EVYGRVIAVIREQCRMFRYVDLIQDTFNIWLGIIFIATMIQICTCLYHITEGYGFDIRYMIFVY
GAVVHIYLPCRYAAKLKAMSMETSNRFYCSGWERVDDERVRKMIVFMIARAQVPNEITAF
NMMAFDMELFLSILQTSYSMFTLLRS-
>HarmOR18
MEMKVDVLPEKKYKGFNETFKLCAFSLAFAFLYPNRTTALRRCITITLIVTFCGGQLFWFIT
YTFKCLYTLDIYNFARNMTLAVVLVLFFIKTYYVIYATSKFAPLLDKISDDLLEANNLEEEF
QVLYDDHIKIAKVGEISWLLIPTIMSALFPIYAGALMTIESIQTDDYERRMVHDMELLFVED
IQSETPFFQCMFAYNCVQCVVLVPNYCGFDGSFCIATTHLRLKLKLMTLKVNKAFKYSKS
RQELRMRLYDSIKDHQDALDFYVQLQNVYGPWLFAVFLLTSFMISFNLYQIYLLQRIDPKY
TSFGVVGVLHIYLPCRYASDLTRVSEEIPDDLYLAQWEAWADPSITKLLMFMITRAQKEMI
VTGMGLVVFNMEMFKSILQTSYSFFTLITA-
>HarmOR17
MFLRSECARSVAPHVRVLRCVGFLRGAALSSRGRAERLALRSYHALALAATSLYVLQQAV
YAYQERGDMDKPSQVMFLMLCHVTCVVKQIAFHVDADRIDRLIASLDEPLLNQCAGERG
ALLRGTARGAARLLRTYAGCAVATCVLWIVFPVINRIQGISFEFPFWTGFSYDHNAVFTLVL
LQSFYCTNLVAIGNTSMDAFMATILDQCKTQLRILRINFESLPERARALHVESGENYDTILD
KLFVDCLVHYNKITEMCTELHDVFAVPLLVQFGVGGWIPCMAAYKIVSLDVLSIEFASITLF
ITCILIELFIFCYYGNEVTVESERVSQSLYSMEWRRARLTFRRSLVLVMERAKRPLRPAAGR
VIPLSLDTFVKILKSSYSFYAVLRQTK-
>HarmOR14b
MAGLRDFFFNYEANEAITTPKNYPYLIIMRISLSLIKCWPKKTTENLAAGAKMKAKVWG
MVQNVLHLAFCVLTIVGTATYVMIHKKNMTFFELGHLYITLMLSCVVFSRLATLTFNEEYQ
VVANEFLNKIHLFYYKDNSEFSMQTHKQIHRVSHLFTLYVTGQMLGGLSLFNLTPMYNNY
SAGKYSKGGLKNSTFDHSLYYSYPFDVSTGVRGYIFSNILHWFFSYIVSTWICTLDLFLSVI
VFHIWGHFKILLHDIDNFPKPSKMVSFKLKNTNVTISNENYSTEELEQLADKLKKCIDYHR
EIISFTNKISEVFGPMLLAYYGFHQASGCLLLLECSQMTPEALARYLPLTLILFQQLIQLSIVF
ELVGTVSSKLNDAVYGLPWEDMDVKNRKTVAFFLLNVQEPVHVKALGPADVGVTSMTKI
LKTSMSFFTFLRSM-
>HarmOR14a
MGGIRDFIFNLEAKEGITKPTDYPYMILCRHLLTVITCWPKEPKEGLDTRAKLKARIWVTF
QKIFHLNGCFITTIGMAMYIALHKNSMSFFELGHLYISLLMTVVIFSRVTTLCWNPEYQAVA
TDFLTKIHLFYYKDDSDFSMQTHKQVHKISHLFTLLLTGQMVAGMSLFNLTPMHNNFSTG
KYKKGGLKNSTFEHSLYFSYPFNASSDVRGYILSNIFHWIISYLCSTWFCTLDLFLSIVVFH
VWGHFKILIHDLNHFPRSLNTISFRLDQSNITLTTEMYSSRELVQVSERLNKSVEYHRRIVS
FTDKMSEVFGPMLFVYYGFHQTSGCLLLLECSQMTVEALVRYLPLTIILFQQLIQLSIIFELV
GSVSDKLKDAVYGLPWEDMDTKNRKTVAFFLMNVQEPVHVKALGLADVGVTSMTAILK
TSMSYFTFLRSM-
>HarmOR12
MEDEPLLIDKTVKNIEFLFRCTGINIKSGTKTRKDMIKSRTVYIINFLWLNIDLAGAVMWFF
TGIANSKSFTELTYVAPCITLSFLGNLKSLFLILREKHVDKLIQVLRDLEINEKARPKSEETD
AIIKYEHNFVTTVISVLNVLYFVLLVAFALSPVSLVALKYFTTNELELLLPFLIVYPFDPYDIR
YWPWVYLRQIWSEVVVIIDICTADYLFYTFCTYIRMQFRLLKHYIERVIPEDDGGGRLTNIE
QVRAEFVLLIKWHQDLISSANMLETVYTRSTLFNFVSSSVLICLTGFNVMAISDVAFVATFL
SFLFMSLLQIFFLCFFGDLLMTSSTEISEAVYNCRWYLADTSLGKDLLLVQTRAQTPCKLTA

SDFSEVNLKAFMKILSTAWSYFALLQTLYGAPT-
>HarmOR10
MAVKNTSLFLGRPKKILSAHGVWPHPNNFVILRKLYMLFVMWTQYSFLLFEIIYIADVWG
DIDAVSEASYLLFTQASLCYKSTAFMVNKQSLLELLEIMDCEIFEPKSAEHEKILAAQARKI
KRLCLFFLTSATTTCTLWAMIPLFDAASKRSFPFRIWMPVTPLKSPDYELGYLYQMVSIYIS
AFLFISVDSVAVSMIMFGCAQLEIIMDKIQKIKYVFESADSEEGRRNIIKTNNEFLVECVKQH
QTVERFIQLCEDTYHANIFFQLTGTVAIICNIGLRISIVEPNSVQFFSMLNYMVTMLSQLFLY
CWCGHELTIRSENLREWLYQCPWYEQDTEFKRALFIAMERMKKPIIFKAGHYISLSRPTFV
AILRCSYSYFAVLNRVNTE-
>HarmOR9
MLDQFDRCLKSVNLYLKFLGLYLESKDTDKTFVERTRSHRLYFAHLFSLNLEVVAQVLWV
LEAVITGKSFVEITRLIPCLILCLISNFKTLSLLYYGRHNNEFIVTMRSLLLNQMQVEEKEHR
FRKNLIDKHVLILTSISKKISYVIVLDLLMFALAHAFIIIPHYFKTDEVKLEMPFIAYYPFNEF
DLRVYPWVYFHQVYSAVIAMIMVYGPDCFFFTCCTFIHIQFSLLNNDMERIVTEETPRYDK
TKFKKLAVRHIELMRCVNLLEKIFSKSILFNALTSSVIICVTGFNVLVVDNIVMMASFTAFLI
FGLMQIFLYCYYGDTIMRSSMEVSTSIYNSLWYNIPAADRKGFLIVIIRAQKPCALTADGFF
KMNLSAFASILSKSWSYFALLKTMYHPE-
>HarmOR7
MKIKMSKPLIFDQSIEKLGVLFRFSGMNIKNKIVTPLDTIKYRWLYTLNFLVVFSAIIGSVYY
VILGIKQGKNFIEVTSVAPCLTFSILSMIKSLYHLMYEEHIQELIDLLTEHEIRENNREKCIEK
EEIIANETGFLNKVINVLYVLNCSMIVVFDMTPIVMIAVKYYKTNEFEMLLPYLDVFSFIPY
ELKYWPFAYIHQIWSECVVLLDMAAADYLFFTCCTYIRVQFKLLQYDFERMIPDRSISKGL
FFEENELRNKFTELLKWHQDIIYSSTILEIIYSKSTLFNFLSSSLVICLTGFNVTIVDDIVIIITFL
TFLSMALMQVFFLCFFADLMMTASLEITNSVYNCKWYSANIKVGKQILFVQTRAQEPCKL
TAAGFADVNLNAFMRVLSSAWSYFALLRTVYGAK-
>HarmOR6
MSFRKFLFENEAVDGIKSPSDYLYIKILRFTLDVIRSWPRKELGEPESASFTVFMKYFYLVLT
IATVVGSILYVVVHVSELSFLEAGLMYLIILMSFLDALTVMSLTFSAKYRVLAKDFLTKIHL
FYYKDRSKAAMEIHKKVHLISHLFSLWLLFQMLSGLSLFNLTPMYSNLAAGKYRRGGLG
NTTFEHSLYYLYPFNTSTDVFGYIVACILHWIISYLCSTWFCMFNLFISLMVFNLWGHFKILI
ITLEEFPRPKSIGTSESAYKYSQEELVEVAERLKDCINYHREIKNFTNRMSDVFGPMLFVYY
SFHQASGCLLLLLECSQMTAQALMRYLPLTIILTQQLIQLSVVFELVGSESEKLKDAVYSVPW
ECMDTKNRKMVRFFLMNVQEPIHVKAMGLANVGVTTMAAILKTSMSYFTFLRSM-
>HarmOR3
MTLSVLDRFYLIDDGFFSFNLKYLFFVGLWPEKTLTRNQKILYKMYEHFISFLTTTFIVLAGI
GTYQHKDDLVVVFCNIDRCLVVYNFFFKTIIFFIKRNQLRDLIDEIEMSGDEVTEERKKLM
ANYVMFITGVTAAVIGAFSLLALFEGTMSIEAWLPSDPMESLMNQILSLEILAFCVFPGLCR
AFAMQGLVCSMIMYLCDQLIHLQKELRDLTYVKETEMVMRTKFKNAIRKHIRLMGYSGR
MENIFKEYFLVQNLAVTVELCLNAVMMTVVGVQQITLLITFLAYLMLALVNAYIYCYLGN
ELIIQSQGIALAAYESTWTSWPVDLQKDLLIVILAAQRPLKLSAGGMALLCIQTFSQALYN
GYSIFAVLNDAVN-
>HarmOR83bORco
MMTKVKAQGLVSDLMPNIKLMQMAGHFLFNYHSENAGMSNLLRKIYASTHAILIFIHYAC
MGINMAKYSDEVNELTANTITVLFFAHTIIKLAFFALNSKSFYRTLAVWNQSNSHPLFTESD

ARYHQIALTKMRRLLYFICGMTVLSVISWVTLTFFGESVRMVTNKETNETLTEVVPRLPLK
AWYPFNAMSGTMYIVAFAFQVYWLLFSMAIANLMDVMFCSWLIFACEQLQHLKAIMKPL
MELSASLDTYRPNTAELFRASSTEKSEKIPDTVDMDIRGIYSTQQDFGMTLRGAGGRLQNF
GQQNPNPNGLTPKQEMLARSAIKYWVERHKHVVRLVASIGDTYGTALLFHMLVSTITLTL
LAYQATKINGINVYAFSTIGYLSYTLGQVFHFCIFGNRLIEESSSVMEAAYSCQWYDGSEEA
KTFVQIVCQQCQKAMSISGAKFFTVSLDLFASVLGAVVTYFMVLIQLK-
>HarmOR13
MKILSDGSDLEGVEKVEDIFYINLARKSMWILDSWPRTPNESVTYRYFVLALNVATLVGG
AVYLRNNTGVLSSFELGHTYITVFMNCITCSRCIMILSREYNEVMLSFVNKIHLFHHRHKS
EYAYKTHIFIHKISHFYTVYLLGLALNGLLLFNMIPFYNCYSRGMFRDVIPANATYDHSVFY
SVPFDYTTKFKGYIAMTSFNCFISYTCTSYFCVVDLTVSLVIFHLWGHMRPLTYHLANFKK
PASVLESNENTDAIKDHSYTQEELKEVFGKLREYIRHHNLILKFSSEMSNAFGPALLAYMV
FHQVSGCILLLECSQLDMKTLVRYGPLTVVILQQLIQISVIFELLGSSNDKLIDAVYLVPWEY
MDTKNRKLVFVMLRQSQRSIDLKMMSMLTVGVQTMTAILKTSFSYFVMLKTVAEEE-
>HarmOR11
MHLAGNAVTGITGPMDYKYMKVLRFVLRIISGWPGKALGEKTLRIEGMGHAYYNTILSLV
YLALGIAYLKKNFHRFDFLELGQLYIVLLMNMLSTSRAFTLCLSQKYREVAKIFIQKIHLFY
FKEKSDFAMKIHITVHKISFISAVYLSVLLFIAACMFNLIPMYNNYSAGRFASFDNLENTTY
EQAISCLYPWNFETNFNGYLAATLSGWYGTILCGSSVSMFDLFLCLMIFNLWGHFKILIYN
LEHFPRPASEVVDAEGEERSGRTVGSEMYSQSELEEVAVLLRDCIQYHMLIYNFTNNMSD
AFGMALFIYYSFHQITGCLLLLECSQMTAAALTRYLPLTIIMFGELVLLSIIFETIGTMSEKLK
DAVYKVPWEYMDTKNRRTVLIFLIKVQEPIHVKAGGLVDVGVTTMASILKTSFSYFAFLRT
F-
>HassOR1
MKILSDGSDLEGVEKVEDIFYIRIARKSMWILDSWPRTPNESVIYRYFVLALNITTLVGGAV
YLRNNTGVLSSFELGHTYITVFMNCITCSRCLMILSRKYNEVMFSFVQKIHLFHHRHKSEY
AYKTHIFIHKISHFYTVYLLGLALNGLLLFNMIPFYNCYSRGMFRDVIPANATYDHSVFYS
VPFDYTTKFKGYIAMTSFNCFISYTCTSYFCVVDLTISLVIFHLWGHMRLLTYHLANFKKPA
SVLESNENTHAIKDHSYTEEELKEVFSKLREYICHHNLILNFSSEMSSAFGPALLAYMVFH
QVSGCILLLECSQLDMKTLVRYGPLTVVIFQQLIQISVIFELLGSSNDKLIDAVYLVPWEYM
DTKNRKLVYVMLRQSQRSIDLKMMSMLTVGVQTMTAILKTSFSYFVMLKTVAEEE-
>HassOR1(2)
GLITIWFMLCGDLLFCIFLSHITTQFDLLAVRVRRLVYVPVDKQLVDTYPLGEYCKDYAGR
NKEMINIFDDNDWEVKHQRELSDIIVRHRALIRLSGDVEDMFSFALLVNFFNSSIIICFCGFC
CVIVEKWNEFVYKSFLTTALSQTWLLCWYGQRLLESSEGLSDALYESGWYRASKSVKSSI
LIMLHRAQKDVHVTTYGFSIISLASYTTIIKTSWSYFTLLLNIYKK-
>HassOR2ORco
MMTKVKAQGLVSDLMPNIKLMQMAGHFLFNYHSENAGMSNLLRKIYASTHAILIFIHYAC
MGINMAKYSDEVNELTANTITVLFFAHTIIKLAFFALNSKSFYRTLAVWNQSNSHPLFTESD
ARYHQIALTKMRRLLYFICGMTVLSVISWVTLTFFGESVRMVTNKETNETLTEVVPRLPLK
AWYPFNAMSGTMYIVAFAFQVYWLLFSMAIANLMDVMFCSWLIFACEQLQHLKAIMKPL
MELSASLDTYRPNTAELFRASSTEKSEKIPDTVDMDIRGIYSTQQDFGMTLRGAGGRLQNF
GQQNPNPNGLTPKQEMLARSAIKYWVERHKHVVRLVASIGDTYGTALLFHMLVSTITLTL
LAYQATKINGINVYAFSTIGYLSYTLGQVFHFCIFGNRLIEESSSVMEAAYSCQWYDGSEEA

KTFVQIVCQQCQKAMSISGAKFFTVSLDLFASVLGAVVTYFMVLVQLK-
>HassOR3
MTLSVLDRFYLIDDGLFSFNLKYLFFVGLWPEKTLTRNQKILYKMYEHFISFLTTTFIVLAGI
GTYQHKDDLVVVFCNIDKCLVDLQFFFKTIIFFIKRNQLRDLIDEIESSGDEVTEERKKLMA
NYVMFITGVTAAVIGAFSLLALFEGTMSIEAWLPFDPMESLMNQILSLEILAFCVFPGPCRA
FAMQGLVCSMIMYLCDQLIHLQKELRDLTYVKETEMVMRKKFKNAIRKHIRLMGYSGR
MENIFKEYFLVQNLAVTVELCLNAVMMTVVGVHQITLLITFLAYLMLALVNAYIYCYLGN
ELIIQSQGIALAAYESTWTSWPVDLQKDLLIVILAAQRPLKLSAGGMALLCIQTFSQALYN
GYSIFAVLNDAVN-
>HassOR4
EDIYHETKPYKLYSIVTFSIYTIMIFLENVAALFGKFPEVEKNSAVMFSAIHDIVLTKMFLLL
YHKQSIRKLNYDMSTVGSSFEEDHVMRKQYLKTTVGIWLYVISVYLSLGAYGVESARRSI
VEGAPFYTVVTYLPFYDDNSVVALIFRIFFYITWLYMMLPMMSADCMPITHLITMTYKFIT
LCHHFERIRTEFDEDTKIMNKREAIDKHRAGCLEGIRMHQKLLCLADEIHRVFGIIMSLQV
CESSAVAVLLLLRLALSPHLDLTNAFMTYTFVCSLFLLLALNLWNAGEVTYQASLLSNAM
FQCGWHLCELEKDNHRDIR-
>HassOR6
MSFRKFLFENEAVDGIKGPTDYLYIKILRFTLNVVRSWPRKELGEPESAAFTVFIKYFYLILT
IATVVGSILYVVVHVTELTFLEAGLMYLIILISILDAITVMSLTFSAKYRVLAKDFLTKIHLFY
YKDRSKHAMEIHKKVHLISHLFSLWALFQMLSGLSLFNLTPMYSNLAAGKYRRGGLGNR
TFEHSLYYLYPFNTSTDVFGYVVACILHWIISYLCSTWFCMFSLFISLMVFNIWGHFKILIIT
LEEFPRPKSIGNLQTAYKYSQEELVEVAERLKDCINYHREIKNFTDRMSDVFGRMLFVYYL
FHQTSGCLLLLECSQMTAQALMRYLPLTIILTQQLIQLSVIFELVGSESEKLKDAVYGVPWE
YMDTKNRRMVRFFLMNVQEPIHVKAMGIANVGVTTMAAILKTSMSYFTFLRSM-
>HassOR7
MKIKMSKPLKFDQSIEKLGVLFRFSGMNIKNKIVTPLDTIKYGWLYTLNFLVVFSAIIGSVY
YVILGIKQGKNFIEVTSVAPCLTFSILSMIKSLYHLMYEEHIQELIDLLTELEIRENNREKCIE
KEEIIANETGFLNKVINVLYVLNCSMIVVFDMTPIVMIAVKYYKTNEFEMLLPYLDVFSFIP
YELKYWPFAYIHQIWSECVVLLDMAAADYLFFTCCTYIRVQFKLLQYDFERMIPDRSISKG
VFYEENELRNKFTELLKWHQDIIYSSTILEIIYSKSTLFNFLSSSLVICLTGFNVTIVDDIVIIIT
FLTFLSMALMQVFFLCFFADLMMTASLEITNSVYNCKWYSANIKVGKQILFVQTRAQEPC
KLTAAGFADVNLNAFMRVLSSAWSYFALLQTVYGAK-
>HassOR8
LVQDVFSRSLFVQFGMGSCIICICLMRLTMPAPLGYYIFLGTYLFVMVLQFIVPCWFGQRII
DKSNLLAFSAYACEWTSETRQFKSNMRFFIDRAKKPLSITG-
>HassOR9
MLDQFDRCLKSVNLYLKFLGLHLESKDTNKTFAQRSRSHRLYFAHLFSLNLEVAAQVLWV
LEAVITGKSFVEITRLIPCLILCLISNFKTLSLLYYGRHNDEFIVTMRSLLLNQMQVEEKENR
FRKELIDKHVLILTSISKKISYVIVLGLLMFALAPAFIIIPHYFKTDEVKLEVPFIGYYPFNEFD
LRIYPWVYLHQVGTAIVAIIMVYGPDCFFFTCCTFIHIQFSLLNNDMERIVTEETPRYDKTK
FKKLAVRYIELMRCVNLLEKIFSKSILFNALTSSVIICVTGFNVLVVDNIVMMASFTAFLIFG
LMQIFLYCYYGDSIMRSSMEVSTSIYNSLWYKIPAADRKGFLIVIIRAQKPCALTANGFFNM
NLSAFASILSKSWSYFALLKTMYHPE-
>HassOR10

MAVKNTSLFLGRPKKILSAHGVWPHPNNFVILRKLYMLFIMWTQYSFLLFEIIYIADVWGD
IDAVSEASYLLFTQASLCYKSTAFMVNKQSLLELLEIMDCEIFEPKSAEHKKILAAQARKIK
RLCLFFLTSATTTCTLWAMIPLFDAASKRSFPFRIWMPVTPLKSPDYELGYLYQMVSIYISA
FLFISVDSVAVSMIMFGCAQLEIIMDKIQKIKYVFESADSEEGRRKIIKTNNEFLVECIKQHQ
TVERFIQLCEDTYHANIFFQLTGTVAIICNIGLRISIVEPNSVQFFSMVNYMVTMLSQLFLYC
WCGHELTIRSENLREWLYQCPWYEQDTKFKRALFIAMERMKKPIIFKAGHYISLSRPTFVA
ILRCSYSYFAVLNRVNTE-
>HassOR11
MYAGNAVTGITGPMDYKYMKVLRFVLRIISGWPGKALGEKTLRIEGMGHAYYNTILSLVY
LALGVAYLKKNFHRFDFLELGQLYIVLLMNMLSTSRAFTLCLSQKYRQVAKIFIQKIHLFYF
KEKSDFAMKIHITVHKISFISAVYLSVLLFIAACMFNLIPMYNNYSAGRFASFDNLENTTYE
QAISCLYPWNFETNFNGYLAATLSGWYGTILCGSSVSMFDLFLCLMIFNLWGHFKILIYNL
EHFPRPASEVVDAEGEERSGRTVGSEMYSQSELEEVAVLLRDCIQYHMLIVDFTNNMSDA
FGMALFIYYSFHQITGCLLLLECSQMTAAALTRYLPLTIIMFGELVLLSIIFETIGTMSEKLKD
AVYKVPWEYMDTKNRRTVLIFLIKVQEPIHVKAGGLVDVGVTTMASILKTSFSYFAFLRTF
-
>HassOR12
MEDEPLLIDKTVKKIEFLFRCTGINIKSGTKTRKDMIKSRTVYIINFLWLNIDLAGAIVWFFT
GIANSKSFTELTYVAPCITLSFLGNLKSLFLILREKHVDKLIQVLRDLEINEKSRPKSEETDAI
IKYEHNFVTTVISVLNVLYFVLLVAFALSPVSLVALKYFTTNELELLLPFLIVYPFDPYDIRY
WPWVYLRQIWTEVVVIIDICTADYLFYTFCTYIRMQFRLLKHYIERVIPEEDDAGRLTNIEQ
VRAEFVLLIKWHQDLISSANMLETVYTRSTLFNFVSSSVLICLTGFNVMAISDVAFVATFLS
FLFMSLLQIFFLCFFGDLLMTSSTEISEAVYNCRWYLADTSLGKDLLLVQTRAQTPCKLTAS
DFSEVNLKAFMKILSTAWSYFALLQTLYGAPT-
>HassOR13
MKILSDGSDLEGVEKVEDIFYINLARKSMWILDSWPKNPNESTTYRFVVLALNVTTLIGG
AIYLRNNTGVLPSFELGHTYITVFMNCITCSRCLMILSKKYNQVMSSFLNKIHLFHHRHKS
EYAYKTHIFIHKISHFYTVYLLWLALNGLLLFNMIPFYNCYSRGMFRDVIPANATYDHAVF
YSVPFDYTTKFKGYLAMTSFNCFISYTCTSYFCVVDLTVSLVIFHLWGHMRLLTYHLANFK
KPASILESNDNTDAIKDHSYTEEELKEVFSKLREYIQHHNLILNFSSEMSSAFGPALLAYMV
FHQVSGCILLLECSQLDMKTLVRYGPLTVVIFQQLIQISVIFELLGSSNDKLIDAVYLVPWEY
MDTKNRKLVYVMLRQSQRSIDLKMMSMLTVGVQTMTAILKTSFSYFVMLKTVAEEEQ-
>HassOR14
MSGIRDFIFNLEAKEGITNPTEYPYMILCRHLLTVITCWPKELKEGLDTRAKLRAKIWVTF
QKIFHLNGCFITTIGMAMYIALHKKSMSFFELGYLYISLLMTVVIFSRLTTLCWNPEYQAVA
TDFLTKIHLFYYKDDSDFSMQTHKQVHKISHLFTLLLTGQMVAGMSLFNLTPMYNNFSTG
KYKKGGLKNSTFEHSLYFSYPFNASSDVSGYILSNIFHWIISYLCSTWFCTLDLFLSIMVFH
VWGHFKILIHDLNHFPRPLSMISFRLDHSNITLTNEIYSSRELVQVSERLNKCVEYHRRIVSF
TDKMSEVFGPMLSVYYGFHQTSGCLLLLECSQVTVEALVRYLPLTIIVFQQLIQLSIIFELVG
SVSDKLKDAVYGLPWEDMDTKNRKTVAFFLMNVQEPVHVKALGLADVGVTSMTAILKT
SMSYFTFLRSK-
>HassOR14b
MAGLRDFFFNFEANEAITTPTNYPYLIIIRRSLSVIKCWPKKTTENLEAGAKLKAKVWGMI
QNVLHLGFCAFTIFGTATYIAIHKKNMTFFELGHLYITLMISCVVVFRLLTLTFQEEYQVVV

NEFLNKIHLFYYKDNSEFSMQTHKQIHRISHLFTLYITGQLFTGLSLFNLTPMYNNYSAGK
YSKEGLKNSTFEHSLYYSYPFDVSTDVRGYIFSNILHWFFSYIVSTWFCTLDLFLSVIVFHI
WGHFKILLHDIDNFPRPSKMVSFRLENTNVTISNENYSTKELEQLAAKLKQCIDYHREIISF
TNKISDVFGPMLFAYYGFHQASGCLLLLECSQMTPEALARYLPLTLTLFQQLIQLSIVFELV
GTVSSKLNDAAYGLPWEDMDVKNRRTVAFFLLNVQEPVHVKALGLADVGVTSMTAILK
TSMSFFTFLRSM-
>HassOR15
YLSGQMMLGLFLFNVTPMYSNYSAGKYKSGGLKNSTYEHALYFPWPFNASTDFRGYVV
SNILHWLLSYSCSSWFCVVDFFLSLMVFHIWGHFKILLHDLNHFPRPSNKISFMIEDSCVTI
TYEMYSRNELDQVFHRLNKCIDYHREIVSFTDKMSEVFGPMLLAYYGFHQASGCLLLLEC
SQMTVAALVRYLPLTIILFQQLIQMSIIFELVGSVSDKLKDAVYGLPWEAMDTKNRRIVAFF
LMNVQEPVHVKALGLADVGVTSMSAILKTSVSYFAFLRSL-
>HassOR16
MGLRQFLFENEAVEGINSASDYLHIKVLRFMLVIVNSWPRKEIGEPESPKFSEFVKYLYLVV
TFLLSAGFILYVVKHNSELTFLETGHMYIVLLMSFIDVSRVATLTMSTTYREVARDFLTKIHL
FYYKDRSKQAMETHRAVHKIAHLFTLWLVCQMLSGLSLFNLIPMYSNYATGRFSGEVSKN
STFEHSMYYPYPFDTSTDIRGYSIACILHWIISYLCSTWFCMFDLFLSLMVFHLWGHFKILN
YTLNDFPRPSSKVEAAKYSEEELVDVAARLKDCIVYHREIILFTDRISNVFGPMLFLYYMFH
QASGCLLLLECSQMTAQALIRYVPLTVILTQQLIQLSVIFELVGSESDKLKHAVYGVPWEC
MDVKNRRSVAIFLANTQEPVHVKAMGVAKVGVTSMAAILKTSLSYFMFL-
>HassOR17
MFLRSECARSVAPHVRVLRRVGFLRGAALSSRGRAERLALRSYHALALAATSLYVLQQAV
YAYQERGDMDKLSQVMFLMLCHVTCVVKQIAFHVDADRIDRLIASLDEPLLNQCAGERG
ALLRGTARGAARLLRTYAGCAVATCVLWIVFPVIDRIQGISFEFPFWTGFSYDHNAVFTFVL
LQSFYCTNLVAIGNTSMDAFMATILDQCKTQLRILRINFESLPERARALHVESGENYDTILD
KLFVDCLVHYNKITEMCTELHDVFAVPLLVQFGVGGWILCMAAYKIVSLDVLSIEFASITLF
ITCILIELFIFCYYGNEVTVESERVSQSLYSMEWRRARLTFRRSLVLVMERAKRPLRPAAGR
VIPLSLDTFVKILKSSYSFYAVLRQTK-
>HassOR18
MEMKVDVLPEKKYKGFNETFKLCAFSLAFAFLYPNRTTALRRCITITLIVTFCGGQLFWFIT
YTFKCLYTLDIYNFARNMTLAVVLVLFFIKTYYVIYATSKFAPLLDKISDDLLEANNLEEEF
QVLYDDHIKIAKVGEISWLLIPTIMSALFPIYAGALMTIESIQTDDYERRMVHDMELLFVED
IQSETPFFQCMFAYNCVQCVVLVPNYCGFDGSFCIATTHLRLKLKLMTLKVNKAFKYSKS
RQELRMRLYDSIKDHQDALDFYVQLQNVYGPWLFAVFLLTSFMISFNLYRIYLLQRIDPKY
TSFGVVGVLHIYLPCRYASDLTKVSEEIPDDLYLAQWEAWADPSITKLLMFMITRAQKEMI
VTGMGLVVFNMEMFKSILQTSYSFFTLITA-
>HassOR20
MDDELEFKPFHETYRLITFSLCIAMIYPNPRTEKWRLISIPILIATVAPVAIMIFLDMYKCWT
NGDIVNIIRHSTVVGPFLGGFFKMILMYHKRIQAKQILDEFDRDHYMFNTVAETYKAIARA
SIRNCQVYSERLWACLVTTCVMTFPVMAIVLNIYNFMFKSEPTKYMIHDLEKPFSKEPEER
FESPYFELLFVYMFYAAILYVVNFTGYDGFFGLCVNHACLKMELYCKALEEAMMADREE
VYGRVIAVIREQCRMFRYVDLIQDTFNIWLGIIFIATMIQICTCLYHITEGYGFDIRYMIFVYG
AVVHIYLPCRYAAKLKAMSMETSNRFYCCGWERVDDERVRKMIVFMIARAQVPNEITAF
NMMAFDMELFLSILQTSYSMFTLLRS-

>HassOR21
YKSSNTTEFLINLNKFGFIFGLPNFWIEELDFSDTFLKIIGRLNKYGNWLVFGLILAEYGAYF
TQKNLDERQTSDFILFIISHTIITGFRVRISHQEVQIRNVMYKLGIALKEVYNDSEAEDQMIK
RSKFFSYALVLNCIMSVLMYTVAAVMRVIRAGVTFTTIITVYPTVEDRSTLSDVVRAIFYII
WCIYLTRVFAVYTLVICLTIAMSHQFKNITSYFYSLSNIFEDEQMTQTEKEQEYERSFRAGIK
IHSETLNCTGDIQRMCRDVFSGQIIFNLTLLIVLMYQMVNSPRNLTNALTLVIAGLTILLSTG
FFMWNAGDITVEAQLLPTAMFSSGWENCGRDSSVRVRKLIVIAMMQAQEPVVLTGLGLI
ALSYQSYVSIVKSSYSVFSVLY-
>HassOR25
DAIQNVFSATLFFQFSFGAMTTCVIMCSLLMPATMEYRVFLVIYLISMAGQILVPSLLGTLLT
HESAELVTAAYNTEWIGRSESFKKSLMLFRQRAATPITITGLKMFPLSLVSFIAIMKTTYSFF
TLIRNAQET-
>HassOR26
EVESIFGEAMVVQFFVMAWVICMTMYKIVGLSIYSAEFVSMAVYLGCMLAQLFIYCYFGT
QLKVESELVNQSIYCCDWMKLSPRFRKQLLVMMQFCDRPIAPRTAYVIPMSLDTYIAVLRS
SYTLFTFLNR-
>HassOR27
FFVATLTLCSVAVRLKSEDMSVMQLVSLIQYMCGTLTQLFLFCRYGDAVLHESTMGMGEG
PFAAASWCLSPRVRRDLSMLSAGMMSQRHLRAGPFSFIDLPSFIQVVRAAYSYYAVLGKK
E-
>HassOR28
LVSGYVEAQMLALTEELLHLYEDAEAEYYKTNQAVSVLDHNNERNLKSKAINEYVEKRL
KEIIQSHGRNINLLHKLEHVFSGAIALEFLLLVIGLIAELLGGLENTYLEVPFALMQVGMDC
FTGQRVMDASVKFE-
>HassOR30
SVDRKHGTKEALSQNYKKLVDCIKHHQAIVTFTELVENAYHPYLLFQLVGSVGIICMSALR
ILVVDWRSMQFFSILTYVSVMISQLFVCCWCGHELTATSEDLHTVLYKCIWYEQDVKFKRE
LCFAMMRISRPLVLRAGHYIILSRQTFVAILRMSYSYFAVLNQTT-
>HassOR33
PMLLPGRHFMDDVVLLYGLEPMFETPNYQISFVLMGSSCVLICYLCANISAFLIIVTGYVQ
AQMLALSEELTHLWEDAEENYRGTELEDITDDGDHNDKNKDAILNDYVTVHLKDIAKSH
AENINLLGQIEGTFRGAIAIEFCLLVIALIAELLGGLQNTYMEVPFALMQVGMDCLIGQRV
MDAGAVFEDAVYDCKWERFNKKNMKTAMVLLLNAQRPMTISAGGVTTLSYVSFMTIIKS
IYSTYTTLRSTMHEP-
>HassOR35
GGLAETIANMLTVLNAVFTIFLLSLSGQFLCDTSSEVADAAYESYWYESDHKVKKLILSIIIR
AQRPSYLSALGFSELTLKSFSKILI-
>HassOR37
WKSMDLAKAAFEGPWMNSDRQTNMFIIILLERCKRPLRLSAGKIFTLSLDTYTVLINWSY
KAFAVMRNMKK-
>HassOR38
MYTYVKIAVFWMNKDKVISLLEYLYCKEFKPKEPEHRDIITKSIKSARFVMTYYSTMCVG
AVSVGIIMPLTENFDILPTNVEYPFFDVYRSPAYEAVYIHHIYYKPATCIIDGVMDTILAAFVT
SAIGQIEILAFNLRNFDLVAERQRRRDLAQNKYIEEYPAQHYVRSVLKECIRHHNCIIRYVS

MIESAFSLASALQFMLSVMVLCLIGIQFLSIE-
>HassOR39
ICHSLFIFLVYCYQGQRLTTASEKLEMAVYYCGWENLRVKERKQVLMMLKRAQEPVIVYA
AKVIPIRLSTFAT-
>HassOR40
LEMAMRNLSLTMLSTVCVFKACNLVIWQNSWKELIDYVSELERSQLSKNDAVVNKIISGY
VKYARRVTYLYWALVTATVVTVILAPLFIYLSSPNYQESIKNGTAPYPEIMSSWTPFDRSRG
LGFCGATLYQMLACFYGGTVVANFDSTAVVIMTFFTGQLKVLSVNCERLFGDGNELVDYD
EAVKRIKECHLHHYYMVEFSSVLNSLLSPVLFLYVIICSLMLCASAVQLTTEGTGNMQRIWI
AEYLMALIAQLFLYCWHSNDVLYMSNKVDDGVYSSA-
>HassOR41
CVTAYQITIETSPMQRFLTTEYLVFGVAQLFIYCWHSNDVLFASADLMRGPYESIWWTRSV
RYRKDLYLLAAQFNKTVVFSAGPFTKLTVATFISILKGAYSYYTLLSQSQMK-
>HassOR43
PYDTSKSPAYELTYINQSSAVTLAALVNVCLDTLVTSLIAVCRCRLRLVALSLRTLCDGIPLP
DKQLISPTEERIVLSRLSQCIIKHESALKAARQIQRCFSLPILAQFAVSVVIICVTAYQLAMEL
NNRNWFRSIPMVAYLLCMALEVFLYCYQGNELLEESSEIAGAAYECPWYQCSVRMRRTLL
IVMVRTRRELRLTAGGITTLSLACFTSIIKASYTFFTVLKQVEDRNPK-
>HassOR44
VIMSEVIMQAHLIPLVCQFNVLANCFENVFEECAAEFPDINKHELVKNNTFVEKYRKRLG
DLVKQHREILDQTTDLKTILSAPMLGQLACSGLLICFVGYQATATIAENLGKFVMSLFYLG
YNMFTLYIICRWCEEITIQSQRIGQSAYFSGWESGVSHVPGARATIILVIARSNKPLVFLAGG
MYTLSLTSYTSLVKASYSALNILLTTKHE-
>HassOR51
EALARYLPLTLTLFQQLIQLSIVFELVGTVSSKLNDAVYGLPWEDMDVKNRRTVAFFLLNV
QEPVHVKALGLADVGVTSMTA-
>HassOR53
EVKERQGSAIVIMSMFGCDSLFYGLCVYIKTHFQLLGLRFENIVGATKSETQRNLAKAVVR
HQELIDLVNQMELLYSKSSLVNIIT-
>HassOR54
KHHQFLMNFHGKIREMYKVIFGAHFLSMTVVLVTTLQTMNVWDYRNTILTGMSGIMPLF
LYCFGGEKLISAGLQMSSAVYSCGWEMMEAKQAKVVLLMLCLLQRPLYLTAADIFIMNR
ETFGDGAQVVYKIYAVFN-
>HassOR55
CSMTICGSVVQFTSKEATASQKLWVIQYTCGLVLQLFLYCWHSNEVTLQSKVVDGGIYNS
NWWKSDVRVRKQLILLAGKLNRPLVLDAGPYTTLSVPT-
>HassOR59
SHFVMITDLKMQTHLLHLLCQFTVLVDCFQNVLRDCRIGFEDVAENNLVYEKRFAEKYTK
RLGDLVKQHKLILSQTMNLRDTLRSPMLGQLAASGILICFIGYQATTTIAESPFQGLMSAFF
LGYNLFGFYIICRWGEEITNQSEKIGEAIYCSGWECGLTKLPGVRSTIMYVIARANKPLVLT
AGGMYNLSLTSYTSLVKTSYSALTVLLQFRHE-
>HassOR60
AAYLNVAKDTLVAALIAQCRCRLRLLGYALRTLDKGMENEAYTFSPEQEKTLSLRLGSCV
VQHQKALDVGKELQECFSEPTFAQLTVSLIIICATAFQLSMGP-

>HassOR62
LWAAWVCVFSVYGPDCFFFTCCTFINIQFRILQDNLKEIVKVDARNRPDVTTETFKAELVQ
LVNDHRELIRCVNLLETTYSRSTLFNVVTSSLIICAAGFNLMVIENLALMAPFTSFLTFGLL
QVFFYCYYGDY-
>MsexOR-1
MIFMDDPLSKSIKDPRDYRYMKLFRSTLRLIGSWPGRDLKEEGATKYEIAPLYWVLVIKIT
CFLLTIIYLIENTNKLGFFEIGHVYITVFMTMITLSRSITLSLNPKYRRVMTKYITKMHLFYY
KDMSDIALKTHIRVHKLSHFFTMYLSTQVVLGTVTFNIVPMYNNYKVGRFENNILVNDSY
ELSIYFKTPTKFLSTLNGYIAITTFNWYSSYICSNFFCMFDLALSLLIFTVSGHFKILIHNLNN
FPLPAVVSDSSKVLKTDEIQAPLYNKTEKKDITLRLKQCIDYHREVLEFTQDISEAFGPMLF
VYYLFHQVSGCLLLLECSQMDAAALMRYGLLTAVLFQQLIQLSVVVESVGTVTGYLKDA
VYNVPWEYMDTQDRKTVCIFLMNVQEPVHINALGLAKVGVQAMAGILKTSFSYFAFLRT
VSN*-
>MsexOR-2
MTMLLRKMYSTVHAILIFVQFVCMGVNMAMYADEVNELTANTITVLFFAHSIIKLGFLAF
TSKSFYRTMAVWNQSNSHPLFTESDARYHQIALTKMRRLTYFICFMTVMSVVSWVTITFF
GESVRMIANKETNETLTEPAPRLPLKAWYPFDTMSGSMYVFVFVFQIYWLLFSMSMANLL
DVLFCAWLIFACEQLQHLKAIMKPLMELSASLDTYRPNTAELFRVSSTDKSEKVPDPVDM
DIRGIYSTQQDFGMTLRGTGGKLQNFVQNTVNPNGLTQKQEMLARSAIKYWVERHKHV
VRLVASIGDTYGTALLFHMLVSTITLTLLAYQATKINSINVYAFSTIGYLCYTLGQVFHFCIF
GNRLIEESSSVMEAAYSCQWYDGSEEAKTFVQIVCQQCQKALTISGAKFFTVSLDLFASVL
GAVVTYFMVLVQLK*-
>MsexOR-4
MKFFVDGSEIAHITKPQDIQYMQMLKFFTNSLAGWPIEAVEGIDGKKNFYWRNGLVVIAY
AYFFGQVFYIYRYINDYTFLVMGHSYITVLMTIVTIARHTLPYFKCYDDTTADFVHNIHLF
NYRNKPGYYKEFHLKIHKISHAFSVYLCTLLVTGPSMFNGIPLYNNYASGAFSFNRSPNVT
YEQAVSLLLPFDDTNNFKGYFVVFLANCCVSYISSCCLCIYDLLLSLMVFHLWGHLKILTK
TLDNFPKPGFLNPQAIEADPNKSLKFSDEELKVIHKKLGECVAHHQLISNFSTRMSNTFGLS
LFIYYGFHQLSGCLLLLECAQLEAAAIICYGPLTLVVFQQLIQLSFIFELIGTVNEGLTDSVY
CLPWEAMDQGNKKIVFTFLRQSQKSMNLKALNMLSIGVQTMAKILKTTMTYFLMLQTIA
KDES*-
>MsexOR-5
MKVPLKKFRPTETTKLLDELNKLFYIFAFRGFWVEEVKLPKTFVKIYDAMYPALNVSLVIF
CALQIGAHFTQKHLNIQQKININIMGIAQPLTKLCCLNCMYYKEECKQVLYHLFVAVKEIY
NDEETEKMLVKKLKFSLWVYMFSSVSTTLLYGMYALIEMVRSGSTFVGTVTAWPDTTDTS
ALAACARVYLYFFWIVYTSTGSMVILMVLTFFMGLTYQYKNLQKYFENLNSIFEDKQVTH
EELEMNFEKALQKGIKAHSDTLWCVNQCQTICKRINMAIVLINTGILIVLMQGFLDSKDDI
LKVGTCLIVLGMCLMILAFFMWNAGDVTVEAQKLSDAIYSSGWYNCYGKRSARIRSLVV
IAMIQAQEPVVFTAFGVIELSYETYVAIIKSAYSVFSVLY*-
>MsexOR-6
MEETKKYAPTDTTKLLDKFNKILYVFGFRGFWMGDVKLPKTVMKIIDILYPVLIITQGTFG
TLQLGAYFTQKHLNSVQKIDALVMGFGQPMMLLYCLNCRYYKKACREVFYHLFVVLKT
VYNDKETEENMVKRLKLYFGAYLIFSLLVVLLYGSYASVETVRKGATFVTVVTAWPDVTD
TSKLASYARVGIFMYWSVYSFTTSVVIIIVLIIFLGLTYQYINLQRYFENLNSIFENDRLSHEE

MEKNFEKALQNGIKAHSETLWCVKQCRMICSAINAGVILLTTGTLVILMPAILGSKDDLLE
AMMYLMVSNIVLMIMAFFMCNAGDTTVEAQKLPNAIYSCGWYNCYGKRSARIRSLVVV
AMIQAQEPVVFTGFGVIELSYENFVTIIKSAYSLISVFH*-
>MsexOR-7
MDEPEFKPFHKTYQIITFALSVGMIYPNPATDKMRLASIPISILTILPLACMIFLEMYQCWTQ
GDIVNIIRHTTVLGPFLGGLFKMFLMYHKRKQAKQILDEFERDYHLYNSFTGDYLNIARD
GIRNSLIYSERGWAITVTTCVMTFPVMAILLNMYNYTFKFQATKYMIHDLNKPGASPEAR
FDSPYYEIMFFYMMYCSLLYVINFIGYDGFFGLSINHACMKTNLYCKMLEDAWKAEPNER
YRRVVAVIDEQCRMFEFVNLIQDTFNIWLGIIFLATMIQICTCLYHITEGYGFDLRYMIFVSG
ATIHIYLPCRYAAKLKAMAAETATHFYSSGWERVNDRRLRNMLLFMVARAQTPLQITAFN
MITFDMELFVSIMQSSYSMFTLLRS*-
>MsexOR-8
MAQTLFDKSLSKLSMVFRWSGTNIAIGEAAPTNTKRNRCIYSFNFILQNTNVLGGIYWFIS
GLKTGKSYTELTFIAPCIIISILSVMKSMSIIIYEKKVYQLMENLRMMEAHERNRENTAERR
KIIEKGVNFLNLVINVLCGLYLIMFVCFAFSPLVLMILKYMKMNEIEFKLPFFIAYPFDPYNI
KVWPMVYLRQLGTEIVTVSNMCVADFIFCIFCSYITIQFRLLQYDIEHVITGTRESIYNDEM
HGGKIRNKLVEIIKWHQELIMCVNLLENIYSISMLFNFISSSVIICLTGFNVTENHDIVLVITFI
TFLFMGLVEIFLLCFFGDMLIDASSDVSDAVL*-
>MsexOR-9
MTSPDSKIKEFFRKFTLITYLCGLADFWIEDLDLPTQFIKCYDTFCKIFNNFLYLYVIAQILSL
FTQHNLTEKQRNYQLMFCVAHPFVVTFPTLIAKHRKKLQSVLFRLVVSLKLVYNDLDIEKE
MIKRIKFYLFAVSAPFWISMISYCVDSYVQVVTSGTTFNIMILAWPLVDDHSVIASLARFIY
HIIWILFETRVTAAYVMVISLTTCISYQYRNLRSYFESIHRVFEEDLTQKEKEKKYEKLLKIGI
QAHSDTLRVTKDGTEACSAVLSAQVLFNTVFLVILMYQAVVVNENRSLVKMFSTLCTVIT
LLFSTWFFTCNAGDVTYEASLIGTAMYTSGWQNCRGLSSLSIRKLLVIALARAQEPIILKGF
WILTLSHQSFLSIVKFSYSIFSLLY*-
>MsexOR-10
MALHFDDTIKKNDFVFRYAGINIKSGERKKNDAIKSRRMYVFNFFWLNIDVYGSIFWFIDG
LRMGKDFIGLTYIAPCISLSTLSLIKSLFLISSEKHMFKLIDNLRELETRENARPRSVQKEEII
NTEIKFLNYVLKTINILYVVLVMTFALSPLIIIAIKYMQTGEVELILPFLILYPFDPYNIKLWPF
VYLHQFWSECVVTLNICCSYIRIQFRLLQHDFERIISAPSGNRRVRENDFKAKFIELVKWHQ
DAIESVSLLETIYSKSILFNFMSSSLIICLTGFNITVVNDFAFVVTFLSFLFMGLVQVFFLCFFA
DLLSESSVEVSNAVYNSQWYMADSNIGKQLLLVQTRAQKPCKLTAWGFADVNLNSFMRV
LSTAWSYFALLQTVYGQ*-
>MsexOR-11
YSFRKVFLNETLGYPHILPCWAVLDELSFLGYLLTIVAEAVAAIYCVVVHITFDITAVGIMIFI
CGQFESLRRCSESIGGKGKVCNVTAERDARARFRIKKCHRIHVILIHSIKELKELIKNILGVY
FFVATFKLCSLAVRLKTENMSKMQLVTLVQYLGASITQLFLLCYYGDAVFNESAITMGQGP
FGAAIWCVSPKIRRDIVILGMGMMKPHSLQAGPFNVLNLPSFIQIVRTAYSCYAVIGPK*-
>MsexOR-12
MEQAKREIDESLKLSAFCMRRIGLSFEKHKNASAHLRQQLMFALSVCSICYHVFSEIMYIG
LTLANSPRVEDVVPLFHTFGYGALSIAKVFALWYKKDVFSQLLRELVGIWPTPPLEDEAQA
IKDKSLDALRITHKWYFAVNVLGVWFYNVTPIAVYFYRLWQDGDAQVGYVWVSWYPFD
KHQTIAHVAVYIFEIFAGQTCVWIMVSTDLLLSGMASHISMLLRMLKRRLESLASTEKTDD

EYYHEILENIKLHQRLITYCYDLEDAFSLSNLVNIVLSSLNICCVVFVIVLLEPFVAVSNKLF
LGSALIQIGMLCWYADDIFHANADVAAAAYNSGWYSTNARCRRALLFLMQRAQKPIAFT
AMKFTNISLVTYSAILTRSYSYFALLYTMYNEN*-
>MsexOR-13
MENNRNFRQAIYSCGWEKQPDKGVRQLILFMMTRASLTLGITTVFYEICLDTFAEMCRQS
YAIFNLMNAAWS*-
>MsexOR-14
DRWIQWKVTAVSAFIGVCALMEHILSMMSAIGLHCAPSQYLRKYILNSHGFLLRINEYSLW
FAIPIFILSKISTMLWNFQDLMIIVISMGLTSRYNRLNMYVGHIIKIERKLSDSPKTGSDLHVH
NEIWRRIRESYVRQAELVGMVDKEFGALILLSNINNLFFICLQLFLGLNATARGALINKLYY
FISLGWMLFRACTVVLAASNVHMHSKKALVFLYSCPKSGFNIEAKRLKHQLIHDHVGLSG
MGLFSLKRQLLLQVAAVVMKYELVLIQYDK*-
>MsexOR-15
MLYIYTRWNNIAFSTHGTIFTIVPITFLILTKVLSAQKESYRRLMKTFLQEIHLCNFGSNSYM
KQRAVEVEKYSRYLATFFFVFLVVYRALWTIVPIVFNLKNSKAIENKEIPLKTCFYMWLPF
DYEHEYKYWVITHIANSTLIALGCIVVTSYDTINYSIVFHLIGHIKSLKHLIKTNISQNLSDD
ETKRGLVEVIRYHCFVLKIFGEIERAFGINVTGNYLYNLIADSLLLYHLMLGDKENKLMYG
VMLLVFMGGLIVMTLILEEVRRQTFDIPQTVYDMPWEKMSVSNQKIVVIMLARTQPTLEY
KSAGGLKAGVNPTIQIIKSTFSYYVMLKSSL*-
>MsexOR-16
MGIFVQNVNRSLRFCLTMLKLTGFLVPNGVIEFTVFHNIYWFFWMMFVVGINVITQTGDLI
QVWGNLSLMTSAAFLLLSDVAMMMKIINVVMRGRVIQTVIDGMDLELRSEARAKGRKII
KECDDQTTRHLYLFLCLSGVTVLGWAGSAEHNKLPLRAWYPYDTSTSPAYELTYIQQNQL
ITTGQNTIDLRLRSCVLKHQAALRAASQIQECFSEPILTQFTASTVIICVTAYQLRIEIHQSNL
VRVISMMAYLLCMMLQVFLYCLQGNQLAEESSNIAEAVYECPWYRLPLPLRRSLLLIMVR
SRRVAQLTAGGIATLSLACFTSIIKVSYTFFTVLQSVED*-
>MsexOR-17
ELDDLLGDAMLLTYIFGSVFICLTAFTATVVGDIYMTVRYVSFFLSLLVEVFVQCIIGQILID
HSEKFERAIYSADWPHSELKTKKMLLILLTRAQKPFVYSANGYLVMNLDTFCGICSLSYQF
FNLLRTAYN*-
>MsexOR-18
MLSFLKNLEDSERPLLGPNYWILKKMGLLLPKNKISKIFYILVHEIVTLFVVSQYMELYVIR
SDLDLVLTNLKISMLSVVCVVKVNTFVFWQSDWKQVLEYVTKADMFERQNQDVPKSNII
NSYTKYCRRLTHFYWGLVFTTFLTTTNTPFMRYLSSSNFRNNFANGTEMFPHIFSSWMPFN
KNHSPGCWITILWHVLLCAYGAAIMAAYDTCVVVIMVFFGGKLYLLRERCSNMFKNSDE
NASEATVKQLHGIHIQLIKYSRLFNSLLSPVMFFYMVMCSLMLCASAYQLTSATNAAQKL
LMAEYLVFGVAQLFLFCWHSNDVLIKNENVTLGPYESNWWSASLRQKKNVLILSGQLRIS
NKFSAGPFADLTLATFITIIKGAYSYYTLLRK*-
>MsexOR-19
DLLIACIKHHQAVKRLVLKIEDTFHSSVFCQLCTAVLIICIIGIRLSQDSPNNASFYGIVSYLTL
ILSQLYLNCWCGTEITDRSLDFRDWLYEVPWYDQDKRFTSTLSVLLQCTKKPLEFRAGHY
VLLARATFVSVLRCSYSYFAVLQQANEG*-
>MsexOR-20
MAVSVLKRLLTYGDNIFEFNIKYLMLVGLWHNENWTRNQKLLYKIYDNTLDVLGLIYLTL

TAIGIHGNMDDITAALAGVDKSLVAYNFMLKIIVFHFRKHQLRKLVKEIIASGDVVPEEHKI
LVAKLSLATTTITTIIVTIFTGISVLAGELPAKVWLPFDTSKNFMNLLAGVQICLVTFGVPICY
RGLALKCFVSTMIFYLRDQLIDLQVKFKELDNFKDAEEEVRTNFKKIVKKHIRLIRYSKTID
NLLREYFLIQNLAITIEVCMNSVTLTLSEGISSAAYNTSWTSWPVDMQKDLLTVIIVAQSHF
KLTAGGMAVMSLETYAQTLYNGYSIFAVLIDAVN*-
>MsexOR-21
MLVGFWHNENWTRNQKLLYKIYDLTMHVFGITDIALSAVGIYQTKHDLTVALAGVDKAL
VAYNFMFKIIGFHFTKDQFKKLVKEMIASGDVVPENHEILMAKLSLATTITSTIIVVIFTGSS
MMVGDLPARVWLPFDISKSFMHLLAGVQFCLVIFAIPVCYRGLALQCFVSTLIFYLRDQLI
DLQDKFKELENFQNVETELRRNFKKIVKKHIRLIRYSKEMDYLLREYFLIQNVAFTTEVCM
NAVILTLIGFSQKTLAINFIAFLITALIHAFVYCYLGDEIIEQSKAYRLLLTIQAGPRGPSTMQ
KNLLTVIIAAQSHLKLTAGGMAVMSLETYAQTLYNGYSIFAVLADAVH*-
>MsexOR-22
MGWIERIKGFILKKSFDFDRPDICLYNFHPQLRILFALKGIFFNKQNSKLKIILPTYFNLLTIL
GMVFEGMFAHRGLTIKDYSFAIESFLYFIILTSTPLVYLCLFYHKDKIIQLLDDMNEEFKFVC
SLGPRHRTPFLKGQLLIWKLCYAWYTLSISTGTAFMMFPVMALVYQTLFVTHTEKTIRPLA
FTMWLPNDDPYRTPNYEIFLFFEMNYCVIIVQTFGVYIYTLFHLLLHHYFILDMMILDFEAI
FDGLDESVAALPSKHPRRREVQLILNARIKRIVTWHNSVIKTINTLSIVYKPALVFQILLSSI
MVCLIGYQIAESLDNGVIDFLFIMLGVCTCMQVWIPCYLGTLLRNKVFAVREACWNCGW
HRNSLGTLIRLDILIIIQRTQVPLLIKLSDMSTVELETFSSIMSAAYSYFNMLRNSN*-
>MsexOR-23
MSSFCQTDIFKPNFFFWKCFGIWGGRTENKNYKYYSFSYLFVTLFVFNILLTINLIYTPLKIE
SLIREVIFYFTEIAITVKVLMILVMRSKILDVFNLLDCKEFQGDDEESKQIIETNHSFYRTCW
KLNAVLSNISFASNVFAPLFINLIWTAKIEFPVCKYYFLSDEMRDKYFIFWFIYQSIGIYGHM
MYNVNVDSFIAGLLLMAITQLKVLNAKFTKFKLEKRHEKHHILIQNKIQILRLNRYLKHY
DCVLRYCEIIQDLLSVTMFVQFGMASAIICAIMCGLLLPSTTETFMFMVTYLFAMTIQIFVP
AWLGTQLSHESCG*VFAAYNCEWIPRSMSFKRSIMIFVERANNPIQLTGLKMFPLSLATFTS
IMKTAYSFFTLFRNLQDHDDGAN*-
>MsexOR-24
LLSLLTKYFFVLNAVLISVYNFSSPIIMLYQYIAKNKVVFVLPYAVLLPFPTDGWLSWFLAF
VYSATCGCICVLFFTTIDVLYCVLTSHVCNNFSIISDQLQHLQVNNVNIIGNIVKEHQYILKL
ADDLEDIFTAPNLFNVLVGSVEICALGFNLTTGNLAQLPGTILFLTSVLLQILVMSVFGENIIT
ESRKIGESAFLCKWYEMDEKSKKMILTIMIRSRKPQILTAYKFSIISYGSFSKIISTSWSYFTIL
QTVYKPPELIVKNNIN*-
>MsexOR-25
MELCKTIWRIITPTKALQQSSGHLETLFFESVYRVSYLMGLSTSDHDMFYLLYSTTVKFMI
TLLVCGELWYIFTETSSLDGIASSINVTLIQFITIYRYKNMMDHKDIYKKLATSMESPYFDTS
NEKRKQLVVFWAKRNEKYLKLLLFLGNCTLAAWYLYPLVDDIEYNLIIGFHTPFSFKTPLR
YPVVYLVVVIAFTYISHFVMVTDLIMQAHLIHLLCQFTVLADCFENLLDDCQHGFEDVPR
NMLVNNKQFAAKYIRRLGHLVEQHKKILKHTVNLRNTLSRPMLGQLAASGTLICCIGYQA
TTTMTESIVKCLMSLFYLGYNSFELYIICLWCEEITTQSMNIGDAIYCSGWECGVTKLPGVR
STIMLVLARANKPLVLTAGGMYNLSLTAYTTLVKTSYSALTVLLRFRHE*-
>MsexOR-26
ITYLYLFGLPNFWIEDFKLPKWFMRSFDIFTKIINNVLYFFILMEMIAFFTQENLSERQKSDL

LVYGISHPILYSYRVFISYKEDNLRAVLLDLVVTLKRVYNDVKVERQMIKKSLLYSSALVFS
CILAMFFYTFDSILHVIRTGATFNVVITTWPKVEDRSTLANAGRIVFYILWWFFMSRVSGAY
TTVICLTTCLSHQYTNLRSYFENLNNIFETNFDQAVKEQKYEDGFKVGIALHLDTLRCTRE
CHSICQGVFSGQIILNILLLVVLMSQMVNSERTLVTAFATASSASAVLISTGYFMWNAGDVT
VEASRLSSAMYLSGWHNCHGRSSITIRKLVVITMFNAQKPVILKGLGIVDLSYQSYLSIVK
SSYSVLSLLY*-
>MsexOR-27
MYTYVKVVVFWFNKKKVVNLLEFLHCKEFKAKEMEHREIIHKSIKRARFVMTFYSTMCV
GAVSVGIVMPLTENFNILPTNVEYPHFNVYNSSTYVAIYLHHIYYKPATCIIDAVMDTMLAA
FVASAIGQIEILTFNLRNFDVVAERRRKKAIKGNKYIGLQNNNNYFMKCILKECIVHHNHI
MRYVSMIENAFSLASALQFMLSVMVLCLIGIQFLSIENPSGHPMQMVWMAIYLTCMLVEV
FILCWFGNELIWKSTGLRQAAFDAPWLATDPKNAKYIILFMERCKRPMKVTAGKIFTLSLD
TYTALINWSYKAFAVMSNMKK*-
>MsexOR-28
MAWFGFIHTPHTGDLLAAMVVLSLGISVQIGTLKFFYTFVYINKETNIVKYYLECDSLIVP
GGRFSGNLLRALRNVKKRAIVYWLVIIINGITYVTKPMFMRGRHHMEDRYVIYGLEPMFE
SPNYEFAYFLMTAGLCFICYPPANVTVFLIVVVGYTEAQMLALSKELLHLWTDANEHYQK
NINQHETTLINAQASKNKIINDYVRYRLKEIIKMHAFNIHLVRQVEFVFRGAIAIGYVFLTL
GLIAELLGGLENTYLQIPFALIQVAVDCYTGQKVTDASLIFERAVYDCKWENFDKMNMKT
VLLLLQNSQKTMTISAGGITMLNFSCLMSVIKSIYSAYTTLRTTMK*-
>MsexOR-29
MKNYYILKTYCSKIFLLGSGDFWYENKVIGDDKRILYRMYSCALFFIYGFMTVLEIMAAL
MGEFPTDEKRDSVTFAVSHAIVMFKIISVVFQKELVKTLNRKMVTICEHYEEQALMSEKY
RIMKINVIVYFLIVYGSAACFVFEGLRKLFDGSHFVTVVTYYPDYEDNSMFANSVRILATVI
LFLMLMNMIVCVDSFTMVYLIMYKYKFITLRHYFENLSVTIDKLNTPGNEEVVAKMLTDG
LVEGVEMHSKLLRLSKDIDKAFGTVMALQLCQSSGSAVSLLLQIALSDQLTFVASLKIVFF
VMALFFLLGLFLCNAGEITYQAAQLSDAIFYSGWHVCRPRHSSRHVRKIVLLAIMQAQQP
LVMKAFKMLELTYGTFILVVRATYSVFALFYAQDK*-
>MsexOR-30
LENAERPLLGPNVKALQFWGLLLPKYRLKRYFYLTMHILVTLFTFTECVDVYFVKSDMNL
LLNNLKITMLATVSVCKVNTFLYWQKRWTDIIAYVTRADLNQRNTKDEKKLALIKKFTLY
CRKITYLYWFLMYTTVVIVVAQPIFKYAFSKNYRDNVRMGTETYLQVVSSWVPFDKNTII
GYIAASIYQTYAAKPNSADIHHTGGRSSMHLEGPIRPIVSRITIHWPSFYNVVTGKTLALPN
LIALQHI-
>MsexOR-31
MAQNTELFLGRPKKILTFFGIWLPSKQYQDLVKIYMILVMITQYSFVLFEIVYIINVWGDIE
VVSEASYLLFTQASVCYKTTAFLIKKRKLVLLLEHMEQEIFASQSKDHEKILLELSLRIKRL
CTFFLTSAITTCTLWAMIPLFDDAGHKTFPFRIWMPVTADESPGYELGYLYQMVSIYISAFL
FIAVDSVALSMIMFGCAQLDIIKDKMQKVREVPICLNVDEKKEKLKSNNDLFTECVRHYQ
NVISFVELVEETYHANIFFQLSGTVAIICNIGLRISIVDHHSVQFFSMLNYMVTMLSQLFLYC
WCGNELTIRSEVLRDWMYQCPWYDQDTKFKRTLWITMERMKKPIIFKAGHYISLSRPTFV
AILRCSYSYFAVLNRANT*-
>MsexOR-32
MSCIMYLAVSEQLTMTLNSEDLYLNRAKFVMKFLGVWVPPNDENIIRKLYRYFMISLQYL

FLIFQIIYIIQVWGDLEVVSQASYLLFTQACLCFKVTVFHVNVDLLKELLLQMNGETFRAQ
SFDHEKILESQAARIKRLLLAFMISSQLTCGMWAVKPLFDDVGSRKFPFDMWMPVTPESSP
HYHIGYTFQLVTICMSAYMYFGVDSVALSSVIFGCAQIEIIKDKLMSIQPVDKNANDAINAF
SDNYQQLVNCIKHHQAIVRFTDTVENAYHTFLLFQLVGSVGIICMSALRILVVDPRSMQFF
SIVSYLSVMISQLFVSCWCGHELTATSEDLHTTLYMCAWKDQNTKFQKELCFAMLRMSRP
LVLRARHYIALSRQTFIMILRMSYSYFAVLNQTVN*-
>MsexOR-33
MAETLLDKSLSKINILFRCSGTNIAIGKAAPTNTKRNRCIYVINFILLNTDVLGAACWFISG
LKIEIVAVFSICAADFVFYNLCSYITIQFRLLQYDIEHVITGTRKSIYNDEMRGGRIRNKLVEI
IKWHQELITCVNLLENIYSISMLYNFISSSVIICLTGFNVTSIEVSNAVYNSKWYLVPPKTRK
TILLILTRAQKPYKLTAYGFADVSLKAFMKVLSTSWSYFALLKTVYSTE*-
>MsexOR-34
MKIFIDNANGTLWLSLNLLRWVGFLVPDNFEGRKKKLLPFYSFFWFMFIVGIYVIVQTGDL
IQVWGDITLMTGTSFLLFTNMAFITKIINVMVRRDAVLAIIDEGDEVLRSERRIEGKAIVKS
SNQETSRLLYLYGLLTVVTVFGWAASAEKGSLPLRAWYPYDTSKSPAYELTYLHQSVAVIL
LAFLNVSLDVLVTSLAAVCRCRFQLLALSLRTLCHDIPIDEKHLVSPEHKQIVHERLRLCVL
QHQSILESAAKIKTCFSGHILAQFTVSIVIICVTAYTLALETRDNPIRLIAMFSYLLGMMMQ
VFLYCYQGDYLSEESSDIADAAYECPWYACPIPLRRSLLVIMARSCRVAILTAGGFSTLSLA
CFVSIIKASYSFFTVLQTSRRMSDHESTCCTNSTHPHKIFGSSSF*-
>MsexOR-35
MVVIEKILSFLEDPRYPSVGPHIRLLSLTGLWHPDLKSPKSRFKIIIFLVTVAFFLSQYVKCIIK
FNTDDLKLILQYAPSHMGIIKTCLFQKDYKIWEELIDFISSVELKQISRKDENLDKVMKAYI
RRNRRVSYFFWALAFFSNFSIFTEPYQKNNFNVNGTSTYLYIFDGYTPFSREPNGYYFSMCI
QTMLGHIVSAYVIAWDTLVVSIMIFFAGQLKITRLYCTRMITANNEESHRNIAECHRFHTSL
VKYQKTFNCLISSVMFVYLVVISVNLGVCIKQIAEIEDDLPTLVSSCVFLMACLIQLLLFYW
HSNEVTIESDLVSYSTFQSNWAQSNKNIQKEVALLALTTRKKLVFRAGPFNVMSLSTFVSIL
RASYSFYTLLKGTN*-
>MsexOR-36
MAETLLDKSLSKINILFRCSGTNIAIGKAAPTHTKRNRCIYSFNFIWQNTDVLGAIYWFISG
LKSGKSFTELTFIAPCIILSFLSVMKSISIIIYEKKVYQLIKNLRMMEAHERNRENTTERGKIT
EKGVNFLNLVINVLYVFNMLVLVCFALNPLILMILNYMKTNEIDFILPFPIAYPFDPYNIKV
WPMVYLRQIWTEIVVVLNTIAPDFVFCIFCSHITIQFRLLQYDIEHVITGTRKSIYNDEMRG
GRIRNKLIEIIKWHQELITCVNLLENIYSVSALYNFISSSVIICLSGFNVTENKDIVLVISFIAFL
FMGLLQIFFFCFFGDMLIHASIEVSDAVYNSKWYLVPPKMRKDFTSNSNSCPTSL*-
>MsexOR-37
MKIFIHNANGTLWLSLNLLMWVGFLVPYNFECQKNNLLPLYSFFWFMFIVGIYGSFRTRN
LIQVWGDITLMTGTSFLLVTNMAFITKIINEMVPRDAGLSIFYEGDEVLRSQRRIEGKAIVK
SSNRETSRLLYLYGLLTVVTVFGWAASAEKGSLPLRAWYPYDTSKSPAYELTYLHQSVAVI
LLAFLNVSLDVLVTSLAAVCRCRFQLLALSLRTLCHDIPIDEKHLVSPEHKQIVHERLRLCV
LQHQSILESAAKIKTCFSGHILAQFTVSIVIICVTAYTLALETHDNPIRLIAMFSYLLGMMMQ
VFLYCYQGDYLSEESSDIADAAYECPWYACPIPLRRSLLVIMARSRRVAILTAGGFSTLSLA
CFVSIIKASYSFFTVLQQVEE*-
>MsexOR-38
LSLPLQSSHVTMDLNFDKMYILTVFAMRINRSHPSIAKDTKWFLQLLPMYGIFSIMFALLIN

CIIHYDLKAKDFSSTCRNGCLCVLYFISTLSYYVMLVHQTTLKTIINTMNEDYAQALKFKA
DEQKVVLDYAKKGLYVCRQWLAMSIMGVGLFFVKNVLLYAYNYYVDDMKLVPLHDMT
YPRIIEEKRDDIIVYASLYALTVYYGVFAAIMYMSFVPLGPVFMLHCCGQLELIVMRLENLF
IKYTHEEANEKLKDIIRHLQNIYGFVKNIEKCFTGFYELTLKATTITLPIAVYEIIESCHRREL
RMEFLVFIFGAMIISNSPCYYSHLLMENGEQVRLAVYCSGWEVVPIPRRLALLSW*-
>MsexOR-39
HVVSVEFGQLQGLHRLVYARRVRRQPVLHFISRIPIYHHPPGGTGYPSASHPYLHSAESDE
LVIELVGNKNPSWQTHCTSVLVQCVKHYIKLKRFSNRLNYICRPFYLTLILVSTMLVCMCS
VKIATSEKLSPDTTKYYVHEFCFIQVVHMFCLLGQHVENECEKLEVAVLEKWHIFNKPHQ
TNVRIFHTAVSQRMPIYIFGTIPLSLPTFTWFMKTGSSFFTLVMSVLEEENA*-
>MsexOR-40
MTYKNRQHQELEMELSHLPGDYLKPLIACFDLLARCNIGFFHGNSSYFKKYWRYSYIISC
VVAYYSSLTVYALKIFLGQMELFELAYVVPVFVVCTQAILKAIIVIIHKGEIRALVLQLGET
WRTDNLTTRQLNKKNLLLKKLNFCYGVFRIVYSYLGTEFLLISLCSHLSTEFCLLREDLLN
VKPVGNRRFRILQDSSNIELHDIVVKHQKLIKFSEQLNEIFNKMIFVNLSSVTITVCFFAFAT
KVARGPVDMANNFMAVMALILPIFNLCYYAEMLINASAGNKQESAYHSLWYVANKQYQ
MSIWFIIRRSQKPCCLTSLKFSPVALHTFTAVLSTTWSYFSLASSLFENEN*-
>MsexOR-41
DLIIEELVFYFTELAAISKVLTFVFFRDKLAKILDALEDPMFQAANGREQKIIDGAKRFNKR
YWKIVATVSLTSHATHILSPIVEHLFLSVPLQLPTCRYSFLSENTIQQFIYPLYLYQTLGMHSH
LWFNANIDSFFLGLMILIIAQLQILDLRLRTVTDVKKNDDIGQANTSEARANYSLTQLNKCI
VHFDEVGKFCGLVEDTFSMTLFMQFSMSSCILCVVLFRFTLPAPFEYYIFLGTYMCVMISFI
FVPCWFGTRVMELSVLLCSSVYECDWTAMPKKFKSNLQLFVERAKRPLTITGGKMFMLS
LTTFTSIMNSSYSFFTLLRNVQTHD*-
>MsexOR-42
MASLWRKYFTKEIQVLKRVYEKSDYEDTYETPRKYLKWSGIRMKHNISKSLSICWLVYY
WFCFVNIVFASVAEIIAMCMTASAGTFDDAIAIFQMMPCNGFCGLSLVKSFKMVKHRPVFE
NLITEIGNMWPQRLVDEEEHKIISSALREIKIFVKGYHWCNNYLMLSFLYPPFWELIKRLSG
EKWEPKLHFIYWLPFDPLQPVYYECMLALQTWQAMTVIWTNMSGDFMFCLFLSHITTQF
NLLSVRIRKLIYVPVDQQLIESYPLGQYSEEYLRKNKEPVDSYTPQQWEEKHFKEITEIVLQ
HQALIRLSRDIENMYSLTLLVNVVNSPLVICFCGFCSVVVEKWNETAYKSFLVTALSQTWF
VCWYGQKLLDSSEGVAEAVYNSGWYRASKKIRRSLMIMLHGSQKGVGVTTYGFSIISLAS
YSTIIKTSWSYFTLLLNFSNK*-
>MsexOR-43
MLERLSKYLENPNHPLLGPTLCGLKCWGMWQPLGVNRIIYNAIHFFAILFVISQYVELWFI
RSNMELAIRNLSVTMLSTVCVIKAGTFVFWQKSWNDVIDYVSGLENIQLSKRDRITNSVIS
EYTKYSRSITYSYWVLVTATVFTVILAPLVGFLSSSDKDLMLNGTLAYPEIMSSRLPFNRSR
GFGYWVAAIEHSLICFYGGGVVANYDSNAIVLMSFFAGQLKLLSINCARLFNDNEVLKYS
DTMKRIKDCHHHHVEIVKFSMVLNSLLSPVMFLYVIICSLMICASAIQLTAEGTSNMQRIWI
SEYLMALIAQLFIYCWHSNEVLHMSSKVDEGVYPSHWSAQNVRVRRSVAPPRRATAQAH
RLHSRTLYQAHYIYFCSYSEGIVQLLYAAKQERRLRT*-
>MsexOR-44
FCTIAKTRAKPVNYKINKKKYNFTSITRTRIHFRLISIQPVDKNANDAINAFSDNYQQLVNC
IKHHQAIVRFTDTVENAYHTFLLFQLVGSVGIICMSALRILVVDPRSMQFFSIVSYLSVMISQ

LFVSCWCGHELTATSEDLHTTLYMCAWKDQNTKFQKELCFAMLRMSRPLVLRAGHYIAL
SRQTFIMILRMSYSYFAVLNQTVN*-
>MsexOR-45
MDVCIMYLAVSEQLTMTLNSEDLYLNRAKFVMKFLGVWVPPNDENIIRKLYRYFMISLQY
LFLIFQIIYIIQVWGDLEAVSQASYLLFTQACLCFKVTVFHVNVDLLKELLLQMNGETFRA
QSFDHEKILESQAARIKRLLLAFMISSQLTCGMWALKPLFDDVGSRKFPFDMWMPVTPES
SPHYHIGYTFQLVTICMSAYMYFGVDSVALSSVIFGCAQIEIIKDKLMSIQPVDKNANDAIN
AFSDNYQQLVNCIKHHQAIVRFTDTVENAYHTFLLFQLVGSVGIICTSALRILVSEDLHTTL
YMCAWKDQNTKFQKELCFAMLRMSRPLVLRAGHYIALSRQTFIMILRMSYSYFAVLNQT
VN*-
>MsexOR-46
MVGVSLPFKYNTPFRYVITYILVGIAFNYTSHFCMVTDLIMQSHLIPLICQYSVLADCFTNL
VSDCEVGFEGIAREHLVNNKKFVKLYLRKLGNLVEQHKFILNHSIELRTILSVPMLGQLAA
SGILICFVGYQATTTISVNITKCLMSLFYLGYNMFTLYIICRWCEEITIQSQNIGEAIYCSGWE
QGMSKIRGVRTTILLVMLRSSKPLIFSAGGMYKLSLTSYTTLVKTSYSALTFLLRIRRE*-
>MsexOR-47
PSPSLRMVRQRGLHSRLLLRRSHGLQLPLVLVFCHHRDPERVAGCVLCSHLPCRCGTGLKP
QDSSLDRIICMGILTVQEITSIMVVASYDMTLLFLFSHTTAMFQILYEDVTNFRELAENYYD
SSEMKSAVFERLKNLLIRHSSILRTVQKMQDVYSVVVGIGFGLNAISMCLFFVLPIDVCLNF
APLIFHSLFVFFLYCYQGQRLTTASEKFEIAVYSCGWEHLGVRDQKTILLMLIQAQKPVIM
MAAGVIPIRIRTFAYTLQNIYKFVTLFKI*-
>MsexOR-48
SLDHSQSSNKEVSVICSFQILTVLLDFPHFHYENFSIFNHTHSQSLDFRDWLYEVSWYDQD
KRFTSTLSVLLQCTKKPLEFRAGHYVLLARATFVSVLRCSYSYFAVLQQANEG*-
>MS|comp957466_c0
WIMGLHQIDCFKIHMKFWRFLAIWPGDDDSRSYNRYSKIFISTFVFVYYVLFTINFYFLPRR
LDIFIDDMMFYFTDCSVVSKVLTFVLMRHKIVAILDVLESEIFQPDDDDGRAIIA-
>MS|comp163536_c0
RRVAMENQNPPERVVPVQYGRGSRAFRQFKNPPQPHMCIQDTIKGTTEKLYINVLGWQKI
ANPKQYSDPIPLYGGMQVPQNYGPNAGRPPMIVFAVMVNPDILKANGKNATSPTDREALV
SLLCDFVEAMNPGLALARNPVIMKDRDLAGELKDVWLAVQNKREREKGMNQDVMYKV
YDIDGIGEDNMNEDDNNQMKQGGSPNRGSGDKKPVKSSKQILLNAGQKSEFDSGMNNC
QINQSHLNRDNKTGNTPDTTYCTPVYGQIVSTRENHNQINDIQQKFASNEIKPSSSFRKDW
NPMHGKTTEGWDEFSKRNISTIKNDEKKRCIKDKSKISNGKSHYDFFPYFDNKTVNETDV
PEEEDSMNKEQNSNEENSKIIIDPMQKLVLHSTDKNICDNNTSALSSISS-
>MS|comp165911_c0
MSRKCNNDPNSFCYVCGILTFKKQRQNFTNLVLECYHQCFGFSVAHQDKFWAPHVCCITC
VKNLTDWKKGARAMPFAVPMIWTEPRDHVSDCYFCLTDIKGINYKKKKQLSTLLPPLHIK
LGLIKNFVKGMDRSGSGVLYLKEKFPKISDAKIKEGIFVGPQIRELIKDSTFEEKLSGLEKC
AWNAFKNVVANLLGNHKGDSYRELVSELLLSYQALGCNMSLKIHFLDSHLDFFPDNLGA
VSDEHGERFHQDISSMEKRYQGKWSPNMLADYCWTIKRDQPEAKHSRKS-
>MS|comp11107_c0
YAYLIMMRHLLSVISSWPLKILDPLDTGAIQRRKIWVSIQRFFHMAVCLSTVVGGVMYVM
LHKKSMTFFELGHLYISLLMTFVIF-

>MS|comp111125_c0
EEEQLFIDITVKKIEFLFRCAGFNIKCSPKTHMDTIKSRTIFIVNFIWMNIDFVGCVVWFITGI
VNSKSFVELTFIAPCVMP-
>MS|comp143892_c0
VGIRLPLFYYMTPERYPLAYTIVVISFFYISHFVMVTDLKMQTHLMHLLCQFTVLGHSTT-
>MS|comp1530192_c0
MSQVVSTCDVLVVVATAGAGVYGAPRRMRKMLKFMENIASVDTSIGGQYSLVTERKLCG
IILGILIFFSVLIADDFTFYALQAKKLDREWEVVTNYLGFYLLWFVVLILELQFAFTALSVRA
RFSAVNDALALTA-
>MS|comp1754988_c0
KAKKFNKIYWKAVAFVSITSNCTHTMAPLMLHFGKGMELIFPVCSYAFFSESFRHMFIYPI
YFYQSVGITFHMLYNL-
>MS|comp1789939_c0
NSTPKYIIFKLQGIPGWLSGLAPAFGSGRDPGVPGSSPTLGSLHGACFSLLLCLCLSL-
>MS|comp1802501_c0
MVVLSLGISSEIGILKLLYMFLYIDNIRGLTDAFLDFDALTTPGSRQASNLLRYMRDVKKRA
IFYWMVLMVNGTIYVFMPLAIP-
>MS|comp2429514_c0
MKSFKALKETLVETKRLVKEDSLESVMFIVNVVPNFVGFSYKKDGKVTSWFWILHLSLLF
YVYA-
>MS|comp43532_c0
KYKGFNETFKLCAFSLAFAFLYPNRTTAIKRCVIITAIVTFCGGQLFWFITYTFKCLYTLDIY
NFARN-
>MS|comp790023_c0
EETKTWITFLSSISKLLLSQTILGVLAFALGPFSLISSNYLKTGEIKLVLPFIVWYPFDELDTRI
W-
>MS|comp102652_c0
CAQLDIVKEKILSITSINGQRGTREVDEALAKNYNKLVNCIKHHQAIIKFTELVENAYHPYL
LFQLVGSVGIICMSALMILVVDW-
>MS|comp102652_c1
VSSQITCGMWALKPLFDDADRKFPFDMWMPLNPEKTVQYYIGYTFQLVTICISAYMYFGV
DSVAFSSVIFGCAQLD-
>MS|comp1032844_c0
KFLGVWVPGENGSFLHTGYRAFMMTLQYLFLIFQIIYIIQVWGDLDAVSQASYLLFTQACL
CLKVTVFQVNMGMLKDLP-
>MS|comp104640_c0
VGKLEAIYRVTIGIGFGLDAISLCLFFVLPLEVCLNFAPLIYHSLFIFFLYCFQGQRLTTASEK
FEIAVYCCGWENLRVKEQRQVLLMLKQAQKPVIVYAARVIPIRIHTFASTMQSIYKF-
>MS|comp107793_c0
QTRAQTPCKLTASNFAEVNLKAFMKVPKPNFY-
>MS|comp110478_c0
LTLVVTALTILCSTGFFMWNAGDITVEAEVLPTAMFSSGWENCWHESSVRVRKLIVIAMLQ
AQEPVALTGLGIIVLSYQSYVSIVKSSR-
>MS|comp110712_c0

MLRRIAEQLSKIFGVVIFIHMVSASLVICFFGFLAVVYSGLADTVANLMTVFNAMTTIFFLT
LSG-
>MS|comp116110_c0
LSFQSENLREWLYQCPWYEQDTKFNRALFIAMERMKKPIIFKAGHYISLSRPTFVSILRMS
YSYFAVLNQTR-
>MS|comp116394_c0
LRNVKKRAMLYWVVVAGNGVLYVMKPVAMRGRNLPENYFIIYGLEPMFDTPNYQIAYF
MMIAGVFFICYVPATVTAFLIVITGYAEAQMLALTEE-
>MS|comp1239304_c0
FWQKYWREVLDYVTEADKFERQSGDPIKTMIVDTYTKYCRRLSYFYWAIVFTTFWTTTG
TPLMTYLSSFTFRENMRN-
>MS|comp124817_c0
KWERIPTSQRRPLLMMMMRAQRPLRLTAAGFTHMDNACFLAIMKAAYSYYAVLSQRQE-
>MS|comp133273_c0
KVRQIVSECLASDAAVVAGSRFSSNLLSALRHVKERAMVFWMVIIFNGVVYIVKPLLTPGR
HFTEDKFILFGLEPMQENPNYQIANMLFAAGVFFTVYVPANITAFLIVVTGYVEAQMLSLS
EELLHLWEDAEEHYYKTEQAISIFDSNKTDLKDKAMNEYVAKHLKDIIRSHGRNINL-
>MS|comp1348560_c0
KFDMITVEYLAAVALDTFVYCYYGHQIILQADRVSTAAYQSMWHTMGVLPRRVLL-
>MS|comp1355432_c0
RISEAVMECKWECIPTSQRRPLLMMIMRAQRPLKLTAAGFKDIDDVCFLSIMKAAYSYYAV
LTQKQEK-
>MS|comp139413_c1
GGNRFVHLVQRYEAVTWYTKAVSEVFNGSMSFEFFSCSAIVCVITYRMSFVSFLFLLVSY-
>MS|comp142810_c0
LWRFFAMLTLREIEHEIRKFGLEYCDLPTMLENVSILLRVLTVNIDSNYHKGIKIYSYILTAV
TAVCFYYVFLFSMSWFVFYRCRITGELVGAMVVLSLGISSEIGPFKLFYMCFHMDKTQKI-
>MS|comp147212_c0
LKALSVETSNRFYSSGWEQIDDQRVRRMILFMVARAQAPIAITAFNMLAFDMELFTSILQS
SYSMFTLLRS-
>MS|comp150812_c0
SSVIICLDGFNVMAINNIATIITCLSFLFTSLLQIFFLCFFGDLLMTSSIEVSDAVYNCRWYLA
DTRLGKDLLLMQTR-
>MS|comp150812_c1
VISVLNVFYFVLAVMVALSPLSLMALNYYTTNELELLLPLLAVYPFDHYNIRYWPWVYLR
QVWSTVIVTIDICTADYIFYIFCTYIRMQFRLLKQNIETFIPDDVIDGRLRNNEAVRTEFVVL
VKWHQDLISSTKMLEAIYTRSTLLNF-
>MS|comp150890_c0
CYLVDNKIFQAFEVGDAGWTCGWHETPLGLMIRDDIIIIIIRAQQPVTIKFTGLPHVQLETFS
SCMSSSYSYFNMLRQYSK-
>MS|comp1523813_c0
DKIQMTRLNKSLKHYEVILTFCDTVQDILSVTLFFQYGVSTLIICVVMTGLALPSSIEFRAFL
AMFLFTMTLRIFVPGFLGTQLSHESEEL-
>MS|comp152388_c0

MADEELEFKPFSDTYRKITFALSVGMIYPNPKTEKWRLASIPILVTSIMPLACMILYDMYKC
WMVKDIVNIIRHSTVVGPFLGGFFKMILMYHKRIQAKEILDEIDRDHELFNDISDIYKDIAR
ASIKNSQIYSERGWVITVSTCVMTFPVIAIVLNIYNFTFKSEPEKYMIHDLVIPMSESEMRFE
SPYFE-
>MS|comp152487_c0
DLKNRTYEQAINCLYPWNFETSLSGYIVANLSGWYGTFLCGSSVSMFDLFLCLMIYNLWG
HFKILIYNLENFPRPAAEVVDAAGEERSGMRIGSEMYSQSELDEVAIQLKDAIQYHKLIVD
FTNNMSDAFGMALFLYYSFHQVTGCLLLLECSTMTAAALTRYGPLTIIMFGELILLSIIFETI
GTMSEKLKDAVYDVPWEYMDTKNRRTVLIFLIKVQEPVHVKAGGLLDVGVTTMASILKT
SFSYFA-
>MS|comp157094_c0
MKLVSDASELEGVEKTEDIPYMQILRKSMWILDAWPKTPNESRIHRYYMVFIKICCLIPGII
YLRNNTGKLSSFEMGHTYITVFMNTIAFSRALMVLSKKYNDILFYFLNEMHLFNYRNKSD
YSYQTHILVHKISHFFTMYLLLMMCLGILLFNLTPMYNNYTDGMYREVKPPNATFDHSVF
YSLPFDYTTNLRGYLALFTFNWYMSCTCSSYFCVVDLTISLLVFHLWGHMRILQYNLQNF
PKPASLLVESKDGEIKDHKYNEQEQIDIHNRLRENLHYHSVIIDFQRRMSDSFGAVLLIYFL
FHQVSECLLMLECSQMDKKALLRYGPLTVVIFQQLIQLSIIFELLGSSNDKLIDAAYCVPWE
YMETKDSKMVLVMLIQSQVSMNLKAMSMLTVGVQTMIAILKTSFSYFVMLQTVAEEEE-
>MS|comp164245_c0
MMTKVKAQGLVSDLMPNIKLMQAAGHFLFNYHSENAGMSNLLRKIYASTHAILIIVHFAC
MGINMAQYSDEVNELTANTITVLFFTHTIIKLGFFALNSKSFYRTLAVWNQSNSHPLFTESD
ARYHQIALTKMRRLLYFICGMTCLSVVTWITLTFFGESVRMITSKETNETLTEVVPRLPLKA
WYPFNAMSGTMYIVAFAFQVYWLLFSMAIANLMDVMFCSWLIFACEQLQHLKAIMKPL
MELSASLDTYRPNTAELFRASSTEKSEKIPDAVDMDIRGIYSTQQDFGMTLRGAGGRLQNF
GQQNANPNGLTPKQEMLARSAIKYWVERHKHVVRLVASIGDTYGTALLFHMLVSTITLTL
LAYQATKINGINVYAFSTIGYLSYTLGQVFHFCIFGNRLIEESSSVMEAAYSCQWYDGSEEA
KTFVQIVCQQCQKAMSISGAKFFTVSLDLFASVLGAVVTYFMVLVQLK-
>MS|comp1998815_c0
FVFLTLYTVVMTLQVMVPCWFGSRLIEKSSQITFSVYDCDWTPRCRRFKSNLRLLVERAN
RPITIRGGKMFLLSLATFTAV-
>MS|comp2023556_c0
IVMVASFTAFLVFGLMQIFLYCYYGDTIMRSSMQISSAIYNSNWYNVGVAERKSFLIVLIRA
QKPCELTANG-
>MS|comp33920_c0
KKDFEYICSEGEKYRAQYFEEQLLTWKICIVMCTFNAGIGIAMNIFAYLSLFYFMATHDAD
TEESRPLLFPFWIPNVDLT-
>MS|comp35555_c0
SFCIATTHLRLKLKLLTLKVEKAFKNSRSRHELRMKMNEAIRDHQDAYDFYVQLQNVYGP
WLFAVFLLTSFMISFNLYQIYLLQRIDPKYTSFGVVGV-
>MS|comp360965_c0
AIVYKKLLIFEQIICGPVVCLTAYCIAEVFFFTIMNLLVFCCHFFSYF-
YFQKIDKGEFQAILLLLCIATIVMYFTPSLLCTYLSIKVRILN-
>MS|comp3974_c0
LMSYLCSVSFCMLDLFLSLMVFNVWGHFKILLHDLATFPLPAKNMSSNNYG-

>MS|comp447468_c0
LEISDAVYNCKWYFATPRVSKQLLLVQTRAQEPCKLTAAGFADVNLNAFMRVLSSAWSYF
ALLQTVYSGK-
>MS|comp50905_c0
EKWNEVAYKSFLVTALTQTWLLCWYGQKLIDSSKRLADALYDCGWYNGSKRAKSAILIM
LHRAQKGIYVTTHGFSIISLASYSTIIKTAWSYFTLLLNFFKD-
>MS|comp55130_c0
LTFILLHMNELVLEMTGIQDHRWRVYCTVVLSQCVDHYVKIKSFSNRLNVICRPFYLALIL
VAIMLVCMCSVKIAISNK-
>MS|comp611033_c0
KSKESVMKINKCILHFDEVGKFCDLVEDVFSATLFVQFGMASCIICVCLMRFTMPAPMSYF
VFLGTYMCVMILQILVPCYYGTRITDKSSLLAFSIYNCDWTPGSRQFKS-
>MS|comp633077_c0
FIPENSNTTLTYEMYTENEQKNVGVKLGELINYHRDIISFTDKISEVFGPMLFAYYGFHQAS
GCLLLLECSQMTAAALIRYLPL-
>MS|comp638207_c0
AYVVGWDTLVVSIMIFFAGQLKITCYYCRMMIDAAKLDKSHRNIADCHRFYTTLVEYTHL
FNSLISPVMFVYLVVISVNLGVNIIQIVEIQDDIPTLVSSVLFVVACLIQLLLFYWFANEVTIE
STFVSYS-
>MS|comp79584_c0
QFKSNMRIFVERANKPLSITGGKMFCLSLPAFTSIMNSAYSFFTLLQQMKD-
>MS|comp80641_c0
DKRVRQIVLFMLMRARIPMGITTVFYVINLDTFAEMCRQSYTLFNLMNAAWE-
>MS|comp813906_c0
GSISDRLKFAVYGLPWESMDKKNRRTVAFFLMNVQEPVHVKALGLADVGVTSMTAILKT
SLSYFTFLKSM-
>MS|comp892037_c0
MELWHQIRKFGLEYCDLPTMLWNVSVFLKLLTLNIYGKNKSGIP-
>MS|comp90072_c0
LSLMVFHLWGHFKILINLLNDFPRPSSGSNFTTEDGFLIRAEKYSKEELQQVSERLAECIIY
HREIVNFTNTMSDVFGPMLFVYYVFHQTSGCLLLLECSQMTTQALIRYVPLTLTL-
>MS|comp904167_c0
LMQVGMDCFTGQRVMDANVKFERAVYDSKWENYSLSNMKIVLTMLQSAQKTMKLSAG
GMIMLSFSCLMQVIRSIYS-
>MS|comp926109_c0
MRLKIIRDFLLKESFDFDRPDIDLYNFHPQLRIFLAVKGVFFSNRNSRLRYIWPAICIQLSVV
A-
>MS|comp94306_c0
FAQLTVSLIIICVTAFQLSIAHPDNMVRLLSMGTYLLNMCFQVFIYCYQGNQLSEESSEIAG
AAYESPWYKCSLRVRRSLLIVMVRTRRALRLTAGGFTTLSLASFMAIIKASYSLFTLLQQV
DE-
>MS|comp94444_c0
EITNQSAKVGEAIYCSGWECGLSKLPGVRSTILFVIARANRPLVLTAGGMYDLSLTSYTTLV
KTSYSALTVLLRFRHD-

>MS|comp948927_c0
RSRQFKSNMRLFVERTNKPLSITGGKMFCLSLPAFTSIMNSAYSFFTLLQQMND-
>MsepOR
MKLVSDASELEGVEKTEDIPYMQILRKSMWILDAWPKNPNESRIHRYYMVFIKICCLIPGII
YLRNNTGKLSSFEMGHTYITVFMNTIAFSRALMVLSKKYNDILFYFLNEMHLFNYRNKSD
YSYQTHILVHKISHFFTMYLLLMMCLGILLFNLTPMYNNYTDGMYREVKPPNATFDHSVF
YSLPFDYTTNLRGYLALFTFNWYMSCTCSSYFCVVDLTISLLVFHLWGHMRILQYNLQNF
PKPASLLVESKDGEIKDHKYNEQEQIDIHNRLRENLHYHSVIIDFQRRMSDSFGAVLLIYFL
FHQVIECLLMLECSQMDKKALLRYGSLTVVIFQQLIQLSIIFELLGSSNDKLIDAVYCVPWE
YMETKDSKMVLVMLIQSQVSMNLKAMSMLTVGVQTMIAILKTSFSYFVMLQTVAEEEE-
>MsepOR2ORco
MMTKVKAQGLVSDLMPNIKLMQAAGHFLFNYHSENAGMSNLLRKIYASTHAILIIVHFAC
MGINMAQYSDEVNELTANTITVLFFTHTIIKLGFFALNSKSFYRTLAVWNQSNSHPLFTESD
ARYHQIALTKMRRLLYFICGMTCLSVVTWITLTFFGESVRMITSKETNETLTEVVPRLPLKA
WYPFNAMSGTMYIVAFAFQVYWLLFSMAIANLMDVMFCSWLIFACEQLQHLKAIMKPL
MELSASLDTYRPNTAELFRASSTEKSEKIPDAVDMDIRGIYSTQQDFGMTLRGAGGRLQNF
GQQNANPNGLTPKQEMLARSAIKYWVERHKHVVRLVASIGDTYGTALLFHMLVSTITLTL
LAYQATKINGINVYAFSTIGYLSYTLGQVFHFCIFGNRLIEESSSVMEAAYSCQWYDGSEEA
KTFVQIVCQQCQKAMSISGAKFFTVSLDLFASVLGAVVTYFMVLVQLK-
>MsepOR1
MAYTPRSLRDFLFDYEPPEGVSSPADYAYLIMMRHLLSVISSWPLKILDPLDTGAIQRRKIW
VSIQRFFHMAVCLSTVVGGVMYVMLHKKSMTFFELGHLYISLLMTFVIFSRISTLCFSDEY
VVVARNFLEKFHLFFYKDRSEYSMQTHKQVHRIAHLFTIYLINQMLAGLFLFNVTPMYNN
YSAGNYASGGLKGNATYEHALYFSYPFNASGDLKWYILANIFHWIISYLCATWFCMHDCF
LSLMVFHIWGHFKILLYNLENFPRPANRISFIPDNSNTTLTYEMYTQNEQKNVGVKLGELIN
YHRDIISFTDKMSEVFGPMLFAYYGFHQASGCLLLLECSQMTAAALTRYLPLTIILFQQLIQ
LSIIFELIGSISDRLKFAVYGLPWESMDKKNRRTVAFFLMNVQEPVHVKALGLADVGVTSM
TAILKTSFSYFTFLKSM-
>PxylOR1
MRVFFLTDGSDLEGVEKLEDIKHIKVVKWTLTSLNSWPHPPHRRRRAYVEQFFLNLQSFLC
IPFIVIYLLRNTGKKDFFRMGHVWITFFMNIVASTRLLLPLTKNYQTLTKSFIEELHLFYHRH
TSEYSMKIHLEIHKLSHFFTMYLTGMMVGGIVLFNATPLANNIFSGAFKKDKPPDLEFEHA
VYYGLPFDSETKYSGYIPVFLYNWFISYFCSSCFCIYDLILSLLIFHLWGHLKILNYNMRTFP
RPGCVAPHIKDTSRLRYDDEEMVIVGRMLREQIDYHRFISDFSDHMSATFGPMLAIYYSFH
QVSGCLLLLECSQLDADALIRYGPLTVILFQQLIQLSIVFELIGSSSEKLKDSVYSMPWECL
DDKNRKILLIFLKKVQTPIHLKAMGIADIGVQTMAGIIKTSLSYFAFLRSK-
>PxylOR2(ORco)
MMNKVKAQGLVSDLMPNIKLMQMAGHFLFNYHEENGGMSMLLRKIYASVHAFLIVIHY
LCMLLNMAQYSDDVNELTANTITVFFFAHTVIKLLYFAINSKSFYRTLAVWNQSNSHPLFT
ESDARYHQLALTKMRRLMYFICAVTVLSVISWVTLTFFGESVRFIPDKETNETLTEPAPRLP
LKAWYPFDAMSGGMYIVAFAYQVYWLLFAMAIANLMDVMFCSWLLFACEQLQHLKAIM
KPLMELSASLDTYRPNTAELFRANSADKEKVPDPVDMDIRGIYSTQHDFGMTLRGAGGR
LQNFGGQQVNNPNGLTQKQEMLARSAIKYWVERHKHVVRLVASIGDTYGTALLFHMLVS
TITLTLLAYQATKIDGLNVYAFSTIGYLSYTLGQVFHFCIFGNRLIEESSSVMEAAYSCQWY

DGSEEAKTFVQIVCQQCQKAMSISGAKFFTVSLDLFASVLGAVVTYFMVLVQLK-
>PxylOR3
MPAGAVYLDYVRILRRFLWFNGCWPGQLFGEEVPLFIRYHKYHVLGQFTVNLMAQINFL
WKFHKDISFLMMGHVYITTFLTSVSLVRSSLPHFEEYRNIVNTFLTEFHLYYHKQKDKYEA
EISAYWDKFSYWFSLCQMALMLLGMTSFNVLPIYKSIQAGAFTTRDIDRDNVEFAVYFAIP
GIDCYDYFYILTALNVYFSYITACSICVLDLLLSLIVFQIIGHIQILNYNILNIPMPEGLKYNKE
ENSVIGKHLISIIDQHRYIVRFAATISSFFGPMLAMNYMFHLVSGCILLLECARPDPETLARF
GPLTIIVFGQLIQLSIIFEFVGYISEKLIDAVYCMPWASMDVSNQKTVKFFLSRIQTPIQLTAM
GIVPVGVQTMLKILKTTMSYFALLKSIRAE-
>PxylOR4
MKPGALSLDYVRTVRRFLWFSGAWPGEVFGEAVPRFIRCHKYLILVQYVLGLIGQVNFLW
KFNKEISFLMMGHVYITTFLTSVTLVRSLLPFFQEYKNITEEFLTEFHLYYHKAKGKYEAEI
CAFWDWLSSWFALYQMALMVLGMTLFNALPVYKSVQAGAFTTTDLHNVTVEFAVYFDI
PGFHCYDHFCILTALNFYFSYITSCSVCVLDLLLCLIVFQIIGHIQILNYKIENIRTPEGLKYNE
DENMQVGKTLIAIIDHHRDIVRFAASISSFFGPLLAINYMFHLVSGCILLLECARPDPETLAR
FGPLTVIVFGELIQLSVIFEIVGYKSEKLIDAAYCMPWESMDVSNQKTVKFFLSRIQTPIQLT
AMGIVPVGVQTMLKILKTTLSYFALLKSISE-
>PxylOR5
MSRKAGALDLQYIQILRRFLWFNGAWPGDVLGEPVPLLIRYHKYQIQVQSILVLIAQVNYL
WKFNDEMSFLMMGHVYITTFLTAVTLVRCSLPFFQEYHNISKTFLTEFHLCYHKHKGKYE
AEICAFWDWLSYWFALYQMALMVLGMTLFNALPVYKSIQAGAFRTMNLQNKTLEFAVY
YTLPVYHCADHFYISTTMNVYFSYVTSCSVCVLDLLLSLIVFQIIGHIQILLYNIENISVAKG
LKYNKEENMAVGKKLIEVIDHHRDIVRFAASISSFFGPMLAINYMFHLVSGCILLLECARPD
PETLARFGPLTVIVFGELIQISVIFEIVGYISEKLIDAAYCMPWESMDVSNQKTVKFFLSRIQ
TPIQLTAMGIVPVGVQTMLGILKTTLSYFALLKSISE-
>PxylOR6
MIQTGERSKALEVKYIKALSKFLWSIGAWPGEEFGDSVALPIRFQRLTLPYQCAGILAAQIY
YLVNHRTNIRFFDVGHVIINCFLTLATGTRTALPSFKGYTLIVKKFINDFHLIHFKDKGEYDE
KVYKITDFVSYYFTIMQMTLMVCGMTLFNMSPLYNDYRMGAFSRHRPPNITMDFAVFYE
FPGATQDEHFYAATFLNLWLSWNCSVSVCSIDLLLSLMVFQIIGHIRILMYDFENLERPKSS
ESVKTEGEESLVPVTVELFDRQENMRVHRKLIDMVIRHRLIVEFADDISSFFGPLLALTYSF
HLVSLCILLLECSQNDPQALARFLPLTAIIFGELVQISVVFEVVGYMGEKLIDSVYLSPWEC
MNVSNQKSMKFILSRIQLPLQVTAMGMVPVGVETMTAIIKTTMSFFAILQSIND-
>PxylOR7
MNEKYLDLSYIRLLKKYLWYTGFWPGEALGEHIPLYIKWHRIQIVVQNVIGFVGQAWYIV
ENFTQMPFVVAGLLYIVASLTVLMAAKCSLTNFKGYQEIIRKLLQEFHLIHHSRGGAYEKKI
IAKVDKISHYCTVYHMAIIFFIVLTFNGIPIFNSYKAGAFRGRNLSGMKLELAIYFRYPGFEC
LDYFYLLSFLNIYFTYIGAVAMFAIDAIVSAIIFQIIGHILILKHDIENLPEPKRETVVVFPHTG
YEGHLGARVRLRLYDDEENKYIHNVLVGIIKHHKYILGYVNEVSGFFGPTVGLNYMFHLV
AGCVLLLECIRCDQDVLLRNLTLVGVSLVNLAVHSVTFEIVGSYSEQLIDAVYSMPWESMD
ISNQKCMKLFLSRVQTPIRVTTMGVIPVGIQTMGAILRTSCTYCTFLVTVDEG-
>PxylOR9
MFKPKEKSSTINNDRILNYTHYSELPLKLVGCWDWFPDAVDMKKIIANYVYLCLVIFVLIN
VTAMLMVSLYSEWVDIMSSLDMLADSLPYVASVLVVAYFAVYRAELYELNDFMNKNFKF

HSARGLTNMTMLKSYKMAKSFAYTYTVCSLCSSTMYAVVPMIIHWWTRTPVQGWFFMD
VTRSPFLEISFLRQILSQIFLGLALGQLGVFFASNSILLCGQLDLICCSARNARFTALLQNGV
KHASLVSQYHDILKDEEHNYIYNTSEMVDSIYHYDEKVTNDFSRIDFDIYSATHDAHTAAA
LTAVARQGQVAAAYKRRFERFASPLLVLRVVQVTLYLCTLLYAASRKLDTMTVEYLLAVSL
DIFIYCYYGNQIIIQADRVSTAAYQSSWHTMGLKPRRLLLNILLANKRQMIVRAGGFLPMD
LRTYVNIIKTSFSYYTLLVNVNEHK-
>PxylOR1-like
MGFFVDNRNRSIRISLTALKFAGFWAPDDLDPKLKTFYHIYSAISFMFLLGTYLIIQVVDMC
LIWGDLQLMTGTAFVLFTNLAQAAKLVNLVLRRRRLRAMLADADAVLRGAAGEEARRIV
DRCDRGTSQQQLLYFCLTTVTVAGWAGSAEKNQLPLRAWYPYDTSKSPAYELTYCHQVG
ALFIAAYLNVAKDTLVSTLIAQTRCRLQLLTLALSKLHDGMASGSATLLSPAEESLAASRLR
ACVGAHQAALRAAAELQACFSAPTAAQFAVSMVIICVTAFQLAAVTQTKNYVRLISMGTY
LMNMAFQVFIYCYQGNELREESASVAGGAYECLWYRAAPAPRRALLLVMLRTRRPAALS
AAGFTTLSLASFMAIIKASYSFFTVLQQVEEKV-
>PxylOR1-like(2)
MTFFGVWLPPKRHIILHNIYMLLVMVSQYSFLLFEFIYLFDVLDDLEAASEASYLLFTQAS
LCYKTTVFLVNKRHLIELLELMRCEMFAPETEVHEKFLSLLAVRIRRLCLFFMTSAITTCTL
WAMIPLFDNQGRRSFPFRIWMPVTPERSPQYQLGYLYQVAAIYISAFLFIAVDSVAVCMIMF
GCAELDIIMDKVTKVWVVWRRLXSKEKRTELLDKNYKLFVDCIRHHQGVIEFMVKVEN
NFHANIFFQLSGSVAIICITGLRISIVSPDSVQFFSMCNYMVTMLSQLFLYCWCGHELTIRSE
KLREVVYQCPWYQQGTKFNRLLWIAMERMKKPIIFKAGHYIPLSRQTFIAILRSSYSYFAV
LNQANSK-
>PxylOR1-like(3)
MKFLGVWLPMENERYYRKIHRTITLSTQYLFLLFQIIDIIRVFGDLEAVSQASYILFTQACLV
FKITLFLATKNSLRLLLEQMNSQVFMPQSTEQERILKLQALKIKRLLLAFAVSSQATCGMW
ALKPLFDDAGEREFPFRMWMPVGTEKSLEYIAGYAFQLLSVCTSAYIYFGVDSVALCMVI
FGCAQLDVIKDKIVKIKPVTMEDRAAKRALNIFKDNYKILVECIIQHQKIVKFTERIENTYH
TYLLFQLSGGVGLICMSALRILVVDWQSMQFFSIITYLSVMIMQMYVCCWCGHELTAHSE
ELYWVLSEGPWYEQSVQFQRALCFTMLRMRRPMVFRAGHYVPLSRQTFVSILRMSYSYF
ALLRQTHKN-
>PxylOR2-like
MKNTSCLKISIAAMVVTGVIDDLEETAEVSLLLLTHLAQVAKVMTIWGRQARIKHLLYLL
DDPSFDRSDPAKKAILESTCRFAWFVSKFLVASTLFTALMWGVFPMLKPTLTVPLNYPYMT
SDSPFFIMMFIYQLTCIVLNALGDTAEDFLVGGLIVLASAQLDVLSFELSHIGQHNDIDINYT
EAVNCVKYHMKIISFVNELEAIFGLPVFIQFVASCIVIGLTAFKIVMTNEPIQLLTLVFYLVCI
LSELLVYCYFGNIIMHKRGGGGGGVRQRLGTHPACASAGPAASAGGGGRPPLPTQRMLLL
LMRRAQRPLAMSAGNMFHLSLLTFTAILKSSYSYFAVVRE-
>PxylOR2-like(2)
MFEKLLSKLEDPNYPLLGPNIKCLKFWGLLLPEEKTFMRKFYICVHSCMFCFMITEFIDIW
YIGSDMNQLITNMKSTMLAIVSVNKVVTYLWWQKDWKNIMAYVTKADIEARTCTDEEN
REIITVYTRYCRRISYLYWLLTYTTAAAVIALPMSYWFSSSTYRDNVRDGTEDYYQVVSSW
VPFNKNKLGGYLAASAVQSFATTYCGGWISSYDTNAIVIMVFFKAELQLIKNRCSKIFEVD
DEEEIMNRIKECHRRHNVLVKYVKQFDSCLSPVMFLYMIACSVMLCSSLYQTTSQASFTQ
KLLTTPYLLLGVSQLFMYSWHGNEVSFMSNELIRGIYESGWWKKATVRREIILLVGTLDRP

IEFTAGPFFNLTIAVFVRIVYKTRLILSKDDQDTMSSAVLSPNSILKGAYSYYMIIIKK-
>PxylOR2-like(3)
YLAASAVQSFATTYCGGWISSYDTNAIVIMVFFKAELQLIKNRCSKIFEVDDEEEIMNRIKE
CHRRHNVLVKYVKQFDSCLSPVMFLYMIACSVMLCSSLYQTTSQASFTQKLLTTPYLLLG
VSQLFMYSWHGNEVSFMSNELIRGIYESGWWKKATVRREIILLVGTLDRPIEFTAGPFFNL
TIAVFVRILKGAYSYYMIIIKK-
>PxylOR2-like(4)
MIKSFLRSLEHPKHPLLGPNICVLVSSGMLQYSEGIMKYFINSIHLFASLFILTQFYDLWLNR
ADVEKSLRDLSYVILSVNGSVKTWAFILCQKYWKDILESVSSIELSQLSKHDRKATMIIRQ
YTDYARKFTYFYWTLVTATVSVVVLAPFVSYFLSSKFRQDLSDGTVTYPEILSSWTPFDKT
SGIGLVIYITTDVLICIIGGAVIASYDTTSIVLLIFFAGQLKLLKTNCARIFGEDCAISPTDIYTN
LRDCHRHHVFLVKYAKILNSLLSPVLFFYVITCSLMICASAVQFTMDTISLMHKLWVAEYL
VALISQLFFYCWHGNYVVFKSEQVCQGIYESAWWQHCVPSRKSILLLGGQLRKRIVFTAG
PFATLTIPTFITVMKGAYSYYTLLSKKEK-
>PxylOR2-likeisoformX1
MIAMAGQLEALSEMFRRALDTDSEEDQYRNLINCYKRYADILFTQKRLNKIMSPILFMYL
LIASINMSLILFSLANLKKSSKIASQVLVVSLVVEAFYYYWHGHQVMHQSENISAAVYDSD
WVDKSPKIRRLVYIMSSTVNRKFVYNAGPFNEVTVITFIQVGNDSIILVSYLIKENGHFSKA
P-
>PxylOR2-likeisoformX2
MIAMAGQLEALSEMFRRALDTDSEEDQYRNLINCYKRYADILFTQKRLNKIMSPILFMYL
LIASINMSLILFSLANLKKSSKIASQVLVVSLVVEAFYYYWHGHQVMHQSENISAAVYDSD
WVDKSPKIRRLVYIMSSTVNRKFVYNAGPFNEVTVITFIQIVKVTVSFYKLMCTTTLSDAG
S-
>PxylOR2a-like
MARETLSDTFNQNKLFWKITGIWVTQVKNKRFKYYAIPVIAIVFVAYDLFLTLNLVYTPKK
LEVFMPELIFFFCEVQNAFKVYMVMFKSEQIIQAFNMMDTEEFIGESEEHKRITKKAKLLFI
KAFKVYFVLCNAGFGCISVLPFVNFVLFGKKPNLPVCNYYFLSDEWRDQYFWWILLYQT
YSVYNHMMYNISIDTFMSGLILMGLAQFRALNDSLKNIKCESNKVNDPAEEWRLRTELIK
CLKHYDSLRKYCTHIQDLYDPAMFVQISVGAASNCVILASLLLSMSSNDKSFIILYGSVMAI
EILMPGYLGSQLQYECDELVRSVYSCDWIERPESFKSIMKLLCERAKQPIILTTWYIIPLSLN
TFISIMKTAYSSFTILRAVHTRNMVE-
>PxylOR2a-like(2)
MPPETISSAFYSNVRGWKIFALWIREVNNKYYKCYAALSLVFLYFLYDGLLTLNLIYSPKNL
NTFIPELVFLLTSFVLVFKINMVMFKSKLILRAFSIMDSDIFSGETVEQKRITKRYVEIDKER
DANIYLYWCYQSLGNFAHMNYGVNIDTFIPGLIMIGIGQFKALNVRLSNIKTYSKXNLVDI
EQEKKLEEELIKCLKHYDLLREYCSLIEEIFDTAMFVQFGVGAAINCVTLVAMLQLPSNEM
FFLILYGVIMAIEIFTPGYLGTQLEHESERTTSAVYECDWMDRSPRFKRNMMLLVERANTS
VELTALTMFPLSLETFVSIMKGAYSCFTLVRAMNGREDTK-
>PxylOR4-like
MAETAGGGSFQDAVDIFRMMPCVGYLLMAMSKSYKMVVHRPVYENLVSELRAMWPRG
PVSKEEHEIISGALRTLNYVVQGYYWCNNALLVIFLSPPFVEIGRRLAGLEVPLILPFFYWF
PFDPFQKGYYEVVLAFQTWHGLITIWFMLCGDLLFCVFLSHVTTQFDLVAARARRLVYVP
ADRQMPGEYPLGVYSSAYLLEDKASVESFSYQDWETRHQKELTEIVQRHHALIRLAGDVE

RMFSFALLVNFFNSSITLCFCGFCCVIVEKWNEMIYKSFLTTALSQTWLFCWYGQRLLESS
EGISDSLYKSGWYLAAKKIKSSIYIMIHRSQKEVHVTTYGFSVICLASYTTIIKSAWSYFTLL
LNVYKK-
>PxylOR4-like(2)
MEIPSFQDLVGQIKVNFRYTGMAYERPQHSIIFYIPFIVITGVVIEETAFFVSRMSSENFLELT
ELAPCLAMGLLSQLKILPIFLKKEKVYKLTEALEILYNSIRNDPAQVVYVKNDVVQIKILIK
YYFILNVILIVCYNFWPLLIMLYNYAVNGEIQFLVPYAILVPFSYDSLYTWFPVFFHLISGGFI
CVLYFTTVDALYFFLTSQVCIQFLVLSNELEKLQNNEQTKLKDIVKKHQYILSLSDDLEDIF
SLPNFFNVIVGSTLICALGFNLTTGELAKMPKFLLFLSSVLLQILMMSVFGEHLIQESGRIAD
AVYLCRWYEMDQSTKNSILIIMRRARRAQKMTAYRFSVISYGSFTKIISTSWSYFTILRTVY
KPDIN-
>PxylOR4-like(3)
MFPGNGDYKESEDIDEEAESDIVIDSLKLSRKVVFVLFWTNLLLILLFDLRPLIVLAFKYFA
HGQLELSLPMFMNYPFDPYDLRFWPIAYGHQCYSSACCIYNLIGPDTLFFVCCTHLYMQF
RILKRRLEIFITGSEIGGELEVAKKFSILAKRHQKLIELVGRLERVYSTSIFLNYGCSSFLICFT
GFNVTTLNDAWSVLNYLVFFSSNVSQIFLICLFGDLIMNSSREVADGIYNCKWYATTPKIRR
SILFYLVRSQKPCKLTAINFADVNLISFTTILSRSWSYFALLRTVMSTRSQDQQTLAAIA-
>PxylOR4-like(4)
MDDFIEISNTFAVTFVVLQAVVKSIAVVHRNEDITYIIRQMAALWPKEEHLTNRQTVVKMK
HLKRLNVCVTVYYYLNIVGSWQYILVPLLETLFRIFVLNQQSMLKFPFGCSFPFDPTESWS
RYLMVYAYETFAMIKLVYFLVGTELLLITMTAHLSILFTLLSDDLLILQPSSRVDGEARDDEI
TYFEADRHEKRIGDIVRRHQKLIKLSKMFDDIFNKVIFYNVTVSTVSICFFGFVAKIAKNPG
EIANNVVGVIASILPIFNLCYYGQLLTDASSSLALSAYLNIWYAKDTRFQKMLGLIMLRSQ
APCCITSYKYAPVTLNMFTGVMSTTWSYFSLICSVYEE-
>PxylOR7a-like
MRLSNIKSKSKKVKDLQHEKHLEKELVQCLKHYDMIREYCSLIEELFDSAMFVQFGVGAL
INCVAFIGLLQQPTEGRFFMMLYDVMMTSGLTTSAAYSCDWISRSASFKRSLVLLVERANQ
AVELTACSMFPLSLATFISIMKGAYSCFTLVRAMNIREENE-
>PxylOR10a-like
MTSAFTPAAFSCLSIVGIVLTTTFVYVGNVSQVMYLLRVLPDRLEAFKACNCISATSVPLST
FVFMWTARNSLQKVFAMCRLGLTFASKESLAHAALLRTLHKSQRLAWLVIANQGFSHFIY
LVAPLLLTIFGNGRYLPTTPGDTYGLSPKYESPFFEITFILTTIATAFSAINQTGYIILFITLVGH
ELGNFYAISETLNNLHETMLDRNIPEDEKRTTIHDTLVFCIKHHQFVRKYHAMLKDLYQTI
FGAHFLMMTIVLVTTLQTLNAWDVRNTILTGVTGILPLFLYCFGGELVISAGEEMAAALYS
CGWELMPATQARLVLMMLRVMQKPLHLTAANIFVMNRETFGDVAQTVYKIYTVFN-
>PxylOR13a-like
MESLWQNFRSILRRFLPYGVLDSWDDLNPRLYLAVHIYWLKFFGMWYNKHSPRSLLYWL
QLLYGAVVLFLTCILPTFGEVYYLLQHTDDVGDIAEGLYLFLSEIYTFFKISVFWWNKAAIL
SLLRYLHCPEFRATEPEHRPALRRRVAAARFVMTYYSTMCVGAVSVGQIEILAFNLQNFD
MLAERRRRRDADAGRGVDVGKGVVARPDDYYVMAVLKDCIKHHNSIISYVALIERAFSL
ASVLQLMLSAMVLCLVGVQFLSIENPASHPMQIMWMVIYLSCMLIEVFILCWFGDDLIWK
SSSLRQAAFDGPWRRCGRRAARCVLLLMTRCQRPLAVTAGKIFPLSLQTYTLLINKFKAFL
SL-
>PxylOR13a-like(2)

MTVMSMGYKGCWKPRALSLDYVRVVRLFLWFNGAWPGDELGEHVPLFIRYHKYHVLG
QFTLGLAGEVNFLRKFYKDISFLMLGHVYITTFLTLVTLVRSSLPHFREYRNIIKIFLTEFHL
CYHKHKGKYEAKICAFWDWLSYWFSLYQMALMVLGMTLFNALPVYKSIQAGAFRTTDL
HNKTLEFAVYYTTPLFHCYDHFYLATTLNLYVSYLTSCSVCAQDLLLSLMIFQIIGHIQILNN
SIENIPIAKGLKYNMEENTEVGIKLIAIIDHHRDIVRFAANISSFFGPMLAINYMFHLVSGCIL
LLECSRPDPETLARFGPLTVIVFGELIQISVIFEIVGYISEKLIDAAYCMPWESMDVSNQKTV
KFFRSRIQTPIQLTAMGIVPVGVQTMLKILKTTFSYFALLKSMNE-

>PxylOR13a-like(3)

MRLTRYISRKPGALSLDYVRTVRRFLWFNGAWPGEVFGEAVPRFIRCHKYLILVQFVLGLI
GQVNFLWKFNKEISFLMMGHVYITTFLTSVTLVRSLLPFFQEYKNITEEFLTEFHLYYHKTK
GKYEAEICAFWDWFSSWFALYQMALMVLGMTLFNALPVYKSVQAGAFTTTDLHNATVE
FAVYFDIPGFHCYDHFYILTALNFYFSYITSCSVCVLDLLLCLIVFQIIGHIQILNYKIENIRTP
EGLKYNEDENMQVGKTLIAIIDHHRDIVRFAASISSFFGPLLAINYMFHLVSGCILLLECAR
PDPETLARFGPLTVIVFGELIQLSVIFEIVGYKSEKLIEAVYCMPWESMDVSNQKTVKFFLS
RIQTPIQLTAMGIVPVGVQTMLKILKTTLSYFALLKSISE-

>PxylOR13a-like(4)

MVPAGAVYLDYVRILRRFLWFNGCWPGQLFGEEVPLFIRYHKYHVLGQFTVNLMAQINF
LWKFHKDISFLMMGHVYITTFLTSVSLVRSSLPHFEEYRNIVNTFLTEFHLYYHKQKGKYE
AEISAYWDKFSYWFSLCQMALMLLGMTSFNVLPIYKSIQAGAFTTRDIDRDNVEFAVYFAI
PGIDCYDYFYILTALNVYFSYITACSICVLDLLLSLIVFQIIGHIQILNYNILNIPMPEGLKYNK
EENSVIGKHLISIIDQHRYIVRFAATISSFFGPMLAMNYMFHLVSGCILLLECARPDPETLAR
FGPLTIIVFGQLIQLSIIFEFVGYISEKLIDAVYCMPWASMDVSNQKTVKFFLSRIQTPIQLTA
MGIVPVGVQTMLKILKTTMSYFALLKSISAD-

>PxylOR13a-like(5)

MEPSKVLVSPGHKYFEFNLKFLFAVGLWPHKEWCRNKLELYKMYETNLHILSVVYLIISSI
GTYNIRGNMEILMSNLDKSLIGYVFVFKIFAFEIKRKELRSLVNDIMQSGDKITKKCEDRM
TSLLMFVMILVTMIVSAFSMSALYDGEMTVEAWMPFDPLQSKKHLLMAAQILAVTFMPC
ALRGVGLQGIVCSVIMYFCEQLQDVQMKIRHLHYSPHRDEEVRQEFKDIVKKHVRLIRYA
KSIENVFNSFMLFHNLAMSVELCLNALMISVVGTEEKKTLVNFLAFFGIALLNTYILCYLG
NEMIIQSEGISQAAYEASWSFWPIDMQRDLLTLITVSQRPLKLSAGGIAVISLQTYCQILYNG
YSIFTMLHDMM-

>PxylOR16

MSVDPTVQEQAKAEILQSLNLSIFSMRQFGLSFDKPPNRRAFIKQKLILYLCFFGISYHIFSD
IVNIGVTLATTPRVEFVVPLFHTFGYGALSSFKLWSVWYKKDVFEQRIADLVDIWPVPPLA
PELQAIKDKSLLALRIAHRFFFGLNVSAVWIFNLTPVMIYVYESWWQGRPDAVVGFPWTC
WYPFDKWDPTNHVFVYLFEILSGVTCVWAMSASDLMLTGMASHICMLLRILHQRLTSLA
ASEQPPPDHYRDIVSCIKLHQRLIVYCNDLEEAFSIVNLVNIVLSSINICCVVFVIVLLEPLSA
LSNKMFLGAALIQVGVICWYADDIYHANSAVAAAAYSCQWHKTSPSCQRALMFLIKRSQ
KPIALTAMNFTNINLTTFSSILYKSYSYFALLYTMYKEN-

>PxylOR17

MEITVNNEKKQKYQNFNGTFRYCTFALAIALIYPNPSRRVKKIKFMVFCLIILSVSFVLLWFI
SYLYFCYIARDMFNFTRNMTVGIVIFLFFFKTFYLNYKSDGFGVVMDAISDDLVKANDMD
EVYQEIFDTYIKKGLVAQMIWIFIPIVQTCIFPVHAGLTMFFNLINEVPQPRVMVLEMDILM
VRSKQMESPWFEIVWFYTILGGFVLFPNFIGFDGSYCISVNHLCLKLRVLTEKLRRAFAETT

TDEQLEKRVKEVIKEHQASLVYYNLLQDVFGGWMFVVFFITSLEITFNLYQLSLGGMDPK
YLIFAFSTVVHNFVPCYFCTRLIEHGDDFCEALYMMPWEARHCQSVTRALAFMIARTQGP
LFLTGMGMVTFNMELFVSVMQSSYSFFTLIRD-
>PxylOR18
MARETLSDTFNHNKLFWRITGIWFRQVKNKRFKYYAIPLIAIVFVAYDIFLTLNLVYTPKKL
ETFMPELIFYFCEVQNAFKVYMVIFKSHQIVQAFNMMDSDIFIGESEEHKRITKKAKLLFIK
AFKVYFWLCNAGFACNSIVPFISFLIFRTKLYLPVCNYYFLSDETRDHYFWYILTYQTYCV
YNHMMYNISIDTFMSGLILMGLAQFRALNDSLKNIKCVSKNKITDEAEERRLRTELVKCL
KHYDYLREYCSLIEEIYDPAMFVQISVGAASNCVILASLLLSMSSNDKSFIVLYGSVMALEI
LMPGYLGSQLTYESEELVRSVYECDWIERPESFKRILKLLVERAKQPIILTTWYIVPLSLNTF
ISIMKTAYSSFTILRAVSTRNAEESS-
>PxylOR23a
MGHFSTFECFRPHFNALARVGYFKIVMRDIPRHKYMLHSAYRAVAFCLVLLYNLQHVVKV
YKSRHSTDMIINVLFVLLTTLNTLSKQLAFNLRSRRVDRLIRVIDGPLFACSKPNHDQSMSQ
NATEMSSLLTLYHGAIFTCGTLWAVFPLVNRALGVDVAFTAYFPFDTSMTPRFELAIAYLTV
LITFQAYGHVTMDCTIVSFYAQGKTQLQIFRYNLEHLVDAHDESVKIDGNLNCNNNDLVG
YKDMGYEARERLKKRFVRCLLHFRQIVWFINEVELIFFEAMVLQFLVSAWVICMTLYKIV
SLSILSAEFVSMAVYLGCILAQLLSYCYYGTQLKVESEMVNQSIYCCDWLQLSPGFRRLLL
VLMERCRRAIVPRIAFVIPLSLETYIAVLRLSYTLFTVLDRK-
>PxylOR24a
MLKLIQSKLEDPNEPFLAPNLKSLHFWGIILPNNAFKRKIYMCMHASLLLFASIVYVDIWFI
RSDFDLLLKSIKYNLLTTINCVKIVTFVAWQGEWLDVIKYVSNADKKSRAKNNEVQNGIIQ
SYTRYCRVVTYSYWFLTCCTVLTVVLQPIAKYCFSPAYRESVRNGSELFDQVLSHKAPFNY
DHVPEYIIALTYTPWATIYAGGLINSFDTISICIMVFFKGELQLLESSCTMLFEGGDKGSVFR
NVKECQNRHAELVKYASVFNGCLSPIMFLYVLVTSFGLCASVYQITIETSTMQRLLTAEYAI
FGVAQLLFYCWHGHDVIHASHSPMRGVFESGWWKHNHCRKEILLLTGQLDQRVYFTAGP
FFNLTLGSFVNILKGAYSYYTLITQKQ-
>PxylOR30a-like
MEFENDFSLKEWAKDKPLSILQLDPADRAVLRIAGEPEKKFKPIAETYRKFTLAMVMGMM
YPNPRTERRRLIYLLIISISFWPVYVMWFYDVFHCLQRLDVDNLTRQLSLGVPIMFCQFKM
FLVTILRKKMQNLIEEINADYERYNCMDAGYQAIVMENQKSLLKLEKVWTYMVLATVAA
FPLMALYQTTEHHLFKEGQRFMVHDVDVPWLKEQRFESPYFEILFVYMVYIALVLVLCYT
AYDGMFFLCIFHACLKMKIISHQLMGALRDHDDVEQMKRNIAVVVEDQCETFRFVDRIRE
EYEFWLVQVFGLTGIQICMNMYQIITSDAAEVSNSAYCAGWEQLADSGVRRSIAIMIARA
QIPVQVQALNMVTFNMELFVSVMQTSYSVFTLLRS-
>PxylOR43a-like
MEKLSYKSLSPHVRMLQVSGILPLARDAPPWKYRLHQAYTALMTVIPYLYALQNLGHMY
KSDKILNSAYSMDWLQLPVTHRRSLLIFLERVRTPIQVVAGHIIPLSAATLVQRRVNPPISAS
PVSKQAMSELAFSSMEPHLRVLRASGSYHLDRASTPPWRYRLHRVYMGFVIFLPYLYVAQ
ELAYVYTVRGNPDQVIYGLFKLFSYIDFIYKKMVLLLKADRVENLLNTMKGPLFNQEGLH
HREILLTQVASAKSTLYIFNFMGNLSCLVWLLVPIVQYSRGHEIRFPLLFPVEINKAPNFYII
MVYLSVQWVNHAYINSTLDVFMSCLLGQCTAQIRILRLDLETAVARSHEKARQESISFSEA
FHKVFTVSLLHHKEIVRMAVNIEDIFGGAMFCQIVVTGWIFCTTAYRAVDMNPASVEFCSM
ITYITCVMVELVLYCLFGNRLAIESDKIPNSAYSMDWLQLPVTHRKSLFIFLERVRTPIQVVA

GYIIPLSASTLVQVLKSSYTFYAVLNKSKEI-
>PxylOR47a-like
MYAASFQVRLSEAMVDKQPEMEYQVMKTLLKIWKDKFIHENMSRKDIIKCTLMHGYIIF
VLISVFLPIVFEDDIKVMLEILVPVFTLVESLSCYYPFLVYRKEYYAIMRDFQVFYEKHKHQ
PAYRQLIQTCDGNASSMVRIFRVGLGACVVLVPAYPALKHALQAGPRRVEPPVAAYYPVEI
GEPANLIITYTSQCVCCYFMQSICLSNISAPIYYFMAVVAHFQVTRQKFKEIEDAKDSQEAL
HLQRCAVRTHQQTLEFLQRVQRVGSASYFILISCAAIGYSFAFFQLASTDDLATIIYYAQFSA
GLSSHTFLLCYYGQKVIDEINALTFSIYSVPWYRYNAGVTRSLVLSLSRAQGTAPVHAWKF
ATVSLITFQSVITTAYSYLAFLETMMTQN-
>PxylOR49b-like
MSTYRQIDCFNINVKYWKFLGIYPRRDTTRLYEYYSFLFISFFFVFYISLTTLNFWFIPGPMD
LFIEETIFYTTEVALLAKVFTFVCMKDKVVKLLDILESDMFQPDTNHRIKIVKDAKKFNITY
WKVMAAISYLSNSTHVLSPLFTHLLTGADLVLPLCSYLFLADDFRNAYIYYCYTYQSIGM
HFFMLVNVNVDSFIQGLIILAIAQLDLLDDKLRKVTENNNIDKDSRNESDTNEDIDYVRQL
NKCIIHFDKVAKYCGLIEEVFSLVLFVQFSIASVILCVCLFRFTLPAPREYYIFLVTYVFVMV
GQILVPCWFGTRVIYKSSQLSLAVYDSDWTPRSRRFKSNLRLLVERLNRPLTIIGGKMFPLS
LVTFTSIMNSAYSFFTLLRHMQSREDEAN-
>PxylOR49b-like(2)
MARETLSDTFKHNVLFWKFLGLWIFQVDDKRYRYYAIPVVSIVFVAYNILLTLNLVYTPKK
LDTFIPELIFFFCEIQNAFKVHMVMFKSKQIVEVFDMMDSDVFIGEKEEHNRITIKSKLLFIK
VFKIFFVGCNMSYICHNFLPLISFLVFGTELNLPVCNYYFLSDESRKHYFWWILVYQTYGIY
HHMMYNLSIDTFMSGLILVGLVQCRALNHGLQNIKSSAPKKENDTEDEKRLRMELVKCIQ
HYDSIRKYCAHIQDLYDPAMFVQMGVGAASNCVILASLLLSMPSHEKALYVSVLMYGTV
MAIEILMPGYLGSQLTHESEQIVRSVYSCDWIDRPESFKRILKLLLERAKRPIILTTGYIVPLS
LNTFISIMKTAYSSFTILRGIHKRT-
>PxylOR67a-like
MQLLSSIWRKLTQTRALEYSSGSYETQFFETVYRVMFLAGVSSHDRGLRLAYSYLVKLTL
VVFVGSELWYLFSQTADLDHVIDNINVTLIHLIAMYRYRDMMRHKAIYKRLAGAMESPH
FDVSTPARRALLQAWARRSERYLQLLLALGTCTLAAWYVYPLVDDLDYNLTVAVRLPYD
YRTPQLYAATYLATLVAFNYTSYFVMVNDLIMQVHLMHLLCQYTVLADCFRNILEDCKEN
KNENYHSLAWGDKYVKRLGDLVNQHKFIMSNTLELKRIWSTPMLMQFLASSMLICLAGY
QVTATIKLSITKFLMSLLYLAYNMFELFIFCRWCDEIKIQSENIAEAVYCSGWERGAAARGG
VRARLMLVLTRARRPLVLTAGGLYDLSLASYSTLVKTSYSALTVLLRVSDD-
>PxylOR67c-like
MSLPSFFCTLVQRNLIAFTTVAPNVGVCMISALKYSIIYNNKPYYDRFFKHYGEDIWQTIPE
SKENAKVISKYTFISKVINRVFVCYSLPLVLYVDSFPWLIMKFQTKFLGKEKQLLYPFDGW
YPFDKTVWYFAAYSWESLMTGIVVMIYMYSDMINIFSVTSICMEFRILGISLKNLVSDEDIQ
QMKGKDAEQVNRRIKNDLKTILAKHDVLAGMCKELDQLLGNTMFANYTSGCGFICLTAF
TFTVVDDFYQSIRCFFFFLSLLAAVLDHCIIGQIISDHSMQLADAIYSSNWTHADQSTKRTL
LILLMRTQKPFELTAKGFVTMDLNTFTDIMSTSYQFFNLLRTCYLPQMGEI-
>PxylOR85d
MSATEKFDHSLYRTKMALLASGIKFSEGYMNKFLDHIINYWLFYFNCFWLYMDVIGELIW
TYEGIQEGRSFEEISVMAPCITVCLLATAKSMPLYLQRGILTNVVKELREIHPDTDGDESED
DDGERRNDEDVVVLERDIAKAAIKHFDSIMQLFLFLCVSVFTFFSLIVSLNVYGSDSLFYA

CCNFIQMHFRILQCQIEFLIGRQWGPNDSHTLNEKFKGIVRRHQGLTEIVRQLEFLYSYSSL
FNFVTSSFLICLSGFIVTTSKETRLVLCFVTFLFMSLAQISIFCYGGDLIMQSSKDVSAAGDR
SRWFVADERLKRSMFIVLARAQRPCKLTAANFADLNLSAFATVLRRSWSYFALLKTMNDQ
-
>PxylOR94a-like
MEENLAISVFRPHLKVLRTSGLFQLDPRTTPRSTFLLHQAFMRFSLLVPLVYATQEVVYMY
QVRSDTDKVIDSMYLFLSFVASVYKQVILWLYADEVEGLMDIVKGPLFNRDDERQKVILR
DLARRAKFIHNWAFGLTQVTCVLWTLKSMVMHLSGSEVDFAIWLPFDHNHRTYFYFVLF
YYWLVTSWLGCNDCNADLFASALLAQCMYQLSYLRIDLETLVETSKEKALKEGRVFHDV
FQEKFKMLLLHYNEIIRLAKGMEYIFGGSIFCLFLVTGWIFCTSTYRCVNINPMSIDFASMV
SYMICVLIEIALYCYFGNGLSYESEQLLYSAYNMDWLQLDTRHRRSLLIFMERVKRPVHT
MAGTIIPLSAGTFLSILRSSYTFYAVLKTTSVSEHI-
>PxylOR94a-like(2)
MEEKLAFSGLKLNLRCMRILGTMFLDPGAGLLQYRIHRVYMRMAILLPFLFIGQQLINIYQ
VRDDADMVMDCMFNLLTFVNNINKQMTLIWKKDQLQALLDDLKGPLFNQDDVRHKRIL
LDFYRWSRVVFWVCLSMPITTMVMWSFLSRLPTHRGGNAVEFGIWLPFDPNDDAYFTLV
MLYEFTQLTWLGCNNCNVDTFVTILLQQCTTQIRILRLDLETAVERAQENAESEGITFDEAF
HKVFTLSLRHYNEIIRLSKNIGHIFGRPIFFQFLVTAWIICTIVYRIVDVNPTSVLFISMIAYMS
CIVVELFLYCYFGTILTYESMKLTNAAYYMDWLRLPSRHRRALVIFMERVKSPIELLAGNIV
PQSTNTFVSIMKSSYTFYAVLKSTNQIDAAIP-
>PxylOR94a-like(3)
MEENLAISVFRPHLKVLRTSGLFQLDPRTTPRSTFLLHQAFMRFSLLVPLVYASQEVVYMY
QVRSDTDKVIDSMYLFLSFVASVYKQVILWLYADEVEGLMDIVKGPLFNREDERQKTILR
DLARRAKFIHNWAFGLTQVTCVLWTLKSMVMHLSGSEVDFAIWLPFDHNHRTYFYFVLF
YYWLVTSWLGCNDCNADLFASALLAQCMYQLSYLRIDLETLVETSKEKALKEGRVFHDV
FQEKFKMLLLHYNEIIRFVINPMSIDFASMVSYMICVLIEIALYCYFGNGLSYESEQLLYSAY
NMDWLQLDTRHRRSLLIFMERVKRPVHTMAGTIIPLSAGTFLSILRSSYTFYAVLKTTSVSE
HI-
>TcasOR1
MMKFKVTGLVADLMPNIRLIQASGHFMLNYHADNSGALHTLRLGYCCMHLVFVLVQTFS
CNFVNLVLERGDVNDLAANTITVLFFTHCVTKFVYFAVRSKLFYRTLGIWNQPNSHPLFVE
SNNRYHGIALKKMRRLLYIIIIWTSFSAIAWTGITFVGDSVHNIKDPENENLTITEPIPRLLVK
AWYPWDAMSGMPYYITLVFQVYYYVFFSLAHANLLDSLFCSWLIFACEQLQHLKEIMKPL
MELSATLDTYVPKSADLFRAPSATSQDQLIENGTNPAKKNEDLKGVYSTRQELGGHFRGG
ALQNFGSGGVGPNGLTKKQELMVRSAIKYWVERHKHVVRLVTAIGDAYGVALLLHMLTS
TIMLTLLAYQATKITGVDKYAATVLGYLLFALAQVFHFCIFGNRLIEESSSVMEAAYSCHW
YDGSEEAKTFVQIVCQQCQKAMSISGAKFFTISLDLFASVLGAVVTYFMVLVQLK-
>TcasOR3
MKLSSVTTCLFSSDFHTRMNFDWKDTIKLNFLMMKIVGLWPKEKYKINFYTLYTLISVNL
FICGHVIFHTVAVFVVGRDLKHLIGALYMSLTETLLLVKICYFIKNSRLVKSLLTSLDGDIFQ
PKNEKQLELTNPSLIFWKKVHKSFAILVANTVFLFVSLPILSKSTKLYRLPLEAWYPYNTQK
SPNYEITYLYQFISTLFRGMASVSMDTFIAALNMYIGVQCDILCDNLRNLNETNFMENLSL
CIKHHKAIVSFARECNKFYNGIVLGQFFSTSIALGLAMFLLSLVTPLSTESNTLLFYLGATTS
EIFLYCWFGNEVDVKSSKIPYSAFESDWTGAPIEAKKNLLIFILRTQKPIKMSAINLFSLSLE

TFTTILRTSWSYFAVLRQVNGQA-
>TcasOR7
MNKLQKFDWKATIRPNIAFLHYLGIWPEGEEYYKLNFYTLKTILYIIILVISTIVFQVINIFFT
LDDLTSLTANIYVLLTEILYFIKLCFLVKNMPALKLLMKTLDHKLFQPKANQIVIIQPLLNFW
KLIFLAFVITCSFTVLFWAIFPILDSSEEEKRLPLLAWYPYDTKISPNYELTYLHQVASYIYIC
YSHLNIDTFITALNTYIQCQFDILCDNLKNIKSDTKNVDTKLAKCIKHHLLILMFANTSNEF
FSWIIFFQFTSSAAITGMTLFQLTVVKPFTTEFYNFMAYVTAEVVQIFMYCWFGNEVQVKS
SNIPYAAFGSDWTEFSPNKQKSLLFLITRSQKSVKMSAFNVFDLTTDSFILKSAWSYFALLN
QVNS-
>TcasOR17
MDDFNWISTVKTNLLLLHIGGIWPRGDGTHKLNLYTIYAIFITFTFTTYHCFSQIINFFFVDD
LQALTESIFISLIQSMALVKAFYILKNMRILKNILKNLETNKMLQPRNLKQIKMVQPSLTQW
RLLSQMFWISAVFAMCLFGAFPIVESTYKEFRLPYLAWYPFDTKSSPFYEIMYLHQFVSSY
TIAIVDIGADTLIAALNVFVATQCEILCDNIRNINGSVEEMDSKWKECFTHHKEILKVARHC
QKFFNWIVLMQFCASVICIGLTMFQLTLVVSFSSEFFSSLFYFGAITVQIFMYCWFGNEVEL
KSSKILYATFEANWVEAPHQVKKNILIFAIRCQNPIKMSSLNVFYLTLETFMAIFRTSWSYFA
VLRQIQNRISEE-
>TcasOR20
MNSFNWQESIKTNLKALRLVGLWPKSDFYKFDLYTFCTSLTVGVIVCGHNLSQIVYILQVY
SDLKALTATIFVASINFLGAVKMYFFIKHIKTVKILFKMLKTYQFKPKNIHQTQLIKPFLNLW
KILYVGYSINVYLIVAMWSLLPVLNGWTWQKKLPFPARYPFDVTKSPYYELAYVYQFICI
WYITVANLNLDTINIALMMYTSCQCDLLCDDLKNLTETRFFHKKLIECIKHHKAILVFAEK
SNGLFNMIVLSQIATSTVVLALTMFQLSMVSPLSSEGLNHLFYIGGIIMQILLYCWFGNEVE
AKSSNILYAIYESTWFEASKNSKKNLLIFSIRCQRPIKATAVKLFALSLRTFITIVRSGWSYFA
VLYNVGSK-
>TcasOR24
MEEDFDFLSSLQTTLFCLRCVGTWPSNTYKLDAYTLYATASITICLFGHNFFQTVNIFFIFND
LNTLTGVIFVALTCLVAILKSLLFIFNMRRLKKLLLVDIRQKLFKPRNRQQVVMVQSRVNF
WKKIYFMFTGMGVATMFFWALFPIMDGTVKEHRLPFLAWYPFSVNKSPFYEITYIYQIVSV
FFIVIVNMNSDMLLVALMNILGVQCDLLCDNLKNIQFRERINEEFLRCVNHHMQILSYASD
CNKFFNTIVLAQFFTTVVSLGLTMYQLTIVTPFTSEFYSFIVYGGAVLMEIFLYCWFGNEVE
FKSLNIPFASFGFDWTIGSVGLQKNLIIFIAKSQRPIRMSALNLFHLSLETFVKILRTAYSYFA
LLNNVNSLN-
>TcasOR46
MSKSEKIHTLATYFDSNIAFLKLTAFWIYDDETTRRKKYLQHAYNIFWIFYLFVAYQPAELL
YVYYSFNDLSVFLRALRDIGNHVSLAYKAFNYFIMRRDILKLMETLQHGNYHYEDCGDF
QPKLIVDEEKKEALKWTKYFLNFCNAICLSMFANGVFTFIFLSDKQYVERNGQRVYHQEQ
PVNTVSPFGSGTKLRFFVTFIYTMIALTFYAWTIVALDSLFITIMSCISSHLKILQGAFKTVRA
RFIKLCASLSKLLISVSGKLESIYSTQTFVQTFISLGEMCFSLYLLSETADQNIGNEITYLIATG
FELLMYCWFGNRITEASLKISYALYESDWFPTSLSFKKQIIFTMTRMQKPINVTIGKITPLAF
STFLTIARGAYSFFTFLKQRHGINH-
>TcasOR58
MPFTIKDYDLRNAFETERTLLTLSGFYPRRTKKYNFFYNTSALINLFIAYGQLFSMVVQMVI
DRNELSKLSETLLFFMTHFTFLCKLTNFVYYKKKMFEIEDNLSRKIFYGFELWQIKPKIDSC

KFIAKIFRILCILVVLFYTLVPYLDDKEDLSLPLPGWLPYNTKKYYYPTVIFQVMSVSVSAY
NNSSIDVLTCMLITVASAEFNLLKGALKTIDFHPKGHNTKQLIEAKFENCVNHHKEIVKFA
YQIETIFSKGIFLQFFASIIVICFTGFQMIVVPIPSMQFIFLIIYFSCMMCQVAMYCWYGHDIIT
TSDSIGQAFYMSNWYESDVKIRKNICIFLERTKKPVILTAGKFVTLSLTTFTTILRSSYSYFAV
LQHLYKEDS-
>TcasOR59
MDEEFLIGTFETEKKFLRYGSFYPCGKRIKFIFLGLFMFVYSWTEFLSMITVLFVERDNLTK
LSETLLFCMTQAAFLFKLVNFLYHNKTMLRIESILKNPILNCLDQFEKNIIEKYMIRVKYLA
RLFRILCILTVSFYGLFPFIDEDPDHMLPLPGWFPFDVKTHQIELVIAQTCGIAIGAFLNSTLD
ILPTILITLGSAQFDILKIRLENITSVDTSKSWLVKKAIKKCVIYHTILLNYITQIEILFHKGIFV
QFTASVVVICLTGFQMLVISVRSIQFILLMIYFSTMTCQIALYCWYGNELMYRSMGLSDAC
YMSEWNKCDTSVCKSLAIIMERGKRPVVLKAGNIFSLKLTTLMTVLKSSYSYFAVLQRLY
ATSE-
>TcasOR60
MSEDYTFRNVFAREKKILTISGFYPLREYEKNYFHFFSGTIQWIISLGMLFSMIIQSVIKRND
LMVLSETLYFLTTHLTFVCKLANLEYHKKLLLDIEDMLKTTRFQKTLSLDLIEKTGMNEKI
RKFNLVAKTFRIVCVWCVVLYVLVPYFDPGKSKTLPTPGWFPFNWTDKYYYGTYFFEVA
GISITAHMDSSIDILSWLLVTIASFQCDILKENLKNIYYNYDKEHDIRETFKDCIRHHEEIIKF
TTKVEQSFSQGILLQFLCSALVICFTGFLMLVVPVLTFQFANTIMYFCCMMIQLGMYCWYG
HEIMTTSDEIGQYFYLANWYDSSLTLRKDFAIFLERAKRPITLTAGGFVVLSLNTFTRILRSS
YSYFAVLKHLYNKS-
>TcasOR61
MGDYDFRAAFAFEKAIFSLSGYYQRQAGFSSLIICAIASLITIAQFLSMVMQIIVAGNDLTVL
SETLLFFMTHFTYMCKLVNLLFYKSKLLHIEDLLSRPRFYGFSQNELTIIKDGIEATNTVANL
FRIFCVLACIAYGLVPYLDHTKAMALPLPGWLPYDTTKYYYPTYFFQMVAVSITASVNSTI
DILTWKLITIASVQFDILKRKLKDLDYKLETTSLQIQFKTCVKHHKEIVNYVKNVEKTFSK
GIFIQFFASVIVICFAGFLIIITPVLSMQFLYLTLYFMCMISQVAIYCWYGHYVMTTSDEIGQD
FYMSNWYESDVAFRKDIIIFMERVKKPVTFTAGNFITLSLVTLTRILRSSYSYVAVLQHLYNE
V-
>TcasOR63
MGFMIQDYDLRNAFSLERKLMLVVGFYPKRDNKHEILYWLSAFFNLLISYGQLTTMIIQM
VFDRSDLSKLTESLLYFFTHFTFLCKLLNFQYYSKDLIEIENFLTDPIFYGYSFEQLDIIKAKI
RSCAFISNAFRICCTFTCSFYCLVPFIDESRKKILPLPGWFPYDTTNYYYSTFFVQSLSLFISA
YCNTAIDILTWKLITLASAQFEILKENLTKIDYEGGFNETKGALVRCITHHAKIVNYTERVE
AIFSKGIFLQLFGSVIVICTTGFQLIVVPIPSVQFAVLGTYLCGMTTQVATYCYYGHEVMTTS
DAIGMSLYLSNWYASHVKIRKIVMIFLEKTKKPTIVKAGNFITLSLATLTQILRSAYSYFAVL
QRLYKDS-
>TcasOR64
MMSDEYVKDVFIANRWMLRCAGLWTPSTRSKLVQIPYKIYAIVVFLFVNVYFTSTEFLSLF
YTHKNLYNFIKNVNFFLTHFMGAVKVIFWFFKGHVLRDLMRTLESPEFHYEPCEGFQPGLI
WRKYRRIGFKYSLGFLALAHMTLSSSYIPPLLTKLPYFSWMPFSYSTPRSYLLALGYQAGP
MFSYAYSIVGMDTLFMNIMNFIAAHLVILQGAFASSKMRVLDPGQMNNEMKRNCRHLQTI
LRVSEDLERVHRYLTLGQLTATLFILCTSLYLISTTPASSKQFYAELVYMVAMGFQLYLYCW
FGNEVTLMASEIPVNVWKADWYDCDQSFKKSMIFTMTRMQKPIYMTVGKFAPLTLQTFV

YILRTSYSIFAVIKNTSI-
>TcasOR65
MTATKSLKEIPPIYLRVHLTVLQILGIDILPVESVPQNLFYTYTALIISTMCLFTIAEFLDMVL
NYEDIYRLTFGLCYCVTHVLGTVKMFLMLYLRKKLWGNLTTLEEGIFKPNPTRGGPEELQI
VNDAITMCNRQGYVFYTLVFLIIGARLLYASLANWPYDKHNYFDGNVTVIVNTKEMPYTT
WMPFDYNDSPLYETIFAFQIFSTTVYGFYIGAADAVICGFMMLIKAQFLIVKRELETLIERA
QKAAIAENPDNEDNFGREIERIELLDKRTQDYVAKYANECVYHHQELIALCDHAEEDFCY
LMLLQFISSLLIVCFQLFQVSTLSPDSVEFFSMVCYLLLMLFQLLCYCWHGNEVQIVSGEL
SRYAFGINWIIMRESPKKTLLLLMMRAQRPCYFTAGKFSLLSLQTFMTIVRGAGSYFMFLR
QMNI-
>TcasOR66
MSKNLKEIPPVYLKVHLTVLQILGIDILPNERIPQTLFYTYSVLLIATMVVFTTAECLDLVLN
YEDIYKLTFGLCCCVTHVLGAAKMFLMLYLRKKLWGYFTTLENGIFKPNPCRGGAEEFEI
VTSAINMCKRQGYVFYVLTVGVTGGQGLYAALANLPYDKHNYFDGNVTVVVNTKQMP
YATWTPFDYNDSPLYEIMFAFQIFSTTLYGFYIGAADAVICGFLMLIKAQFLIVKRELETLVE
RAQRAGNPDRGDFGGGINRIEMLDDGTQVFVEKCANECVYHHQELIALCEHAEEDFCYL
MLLQFISSLLIVCFQLFQLSTLSPGTFEFFSMACFLLFILFQLLCYCWHGNEVQFVSGELSR
YAFSINWIIMRESPKKTLLLLMMRAQRPCYFTAGKFSLLSLQTFMTVVRGAGSYFMFLKQ
MNT-
>TcasOR67
MDFTIRDFDLRNSFSLERKLLLVLGFYPIRDKEKHRILHQLSAFLNLLLYYGQLLTIIIQMVI
DRNDLSKLTDSTLYFLTLFTFLCKLFNFQYYGKDLIEVEKSLTDPIFYGYSFHKLQIIKAKVR
SCTLVCLAFRISCTCSCFIYSVVPFIDRSGQKTLSIPGWFPYDTAKHFYITFFLQSLSLFISAH
CNSATDTLPCKLISLATAQFELLKDNLRTIDYENSFEETKHALVKCITHHRKIVNYTKRVETI
FSKGIFLQLFASVLVICTTGFQLVIVPFGSLKFAIHGIYLCAMTAQIAIYCYYGHDVMITSDEI
GTSLYMSNWYASHIKIRKIMVIFLEKTKKPTIVLAGNFITLSLVTLTQILRSAYSYFAVLRRL
YADD-
>TcasOR72
MAKLEYLTGATFTLKCAVLYPIDSNNPKIKKILYAVWAIFFILTFVTGFIQCFVFVCINPFDLV
QEAMIIMSLVFYSTTFFYFIVFYKNWQNMVALVTNINKNFHRATDNVIEKISMDQASELSD
KLAYVWTSSLAVGSVVPVVLAIATGNLEMPMPAWFPYDYNKSPVFEITYLWQVFCLITLAI
IYGASDMFFPCITIIIGQQFKILASNFKNNFYTSLIKLGAEESIVQNFSKDIKTHEFRSFYIKY
GNIFKILNNAKFQTLNRAFLKRNIKHHKLLLRFCEDLNKILNTFLLIRVSAIVFNLIFIGFNIII
NADFLWTLYECPWYLCDVTYQKMLILVQMRVKRMVSTKAGNFFTMIAPSFIAFQRAVFS
YITLLKEVTDLGKD-
>TcasOR73FIX
MTRKHIFLNFTVTILKLSFLWPSNDNYDQWRLVKDASLIVSLMPCALPILAHFVLQITGDV
YNMVTITENLIALICIIGMIYMTICFVKNRKLVKTLVKNLPAFTKYSKTTDIILTDKKANLYT
KIFVFYGVIGNVVYMIMPYLNIEKCQQRQNNDVPCGLVTRCWFPFKFDYSPVFEIVFVHQ
FYTCLMVSVIILDLTMLICGFLMHITNQLKHLRGFIKRFDCSSQKIAEDVIYCVKFHTAIITY
SEKTNEAFGTMMMLHITLTSLVISALGFEILIVDNFNDSLRFTLHLLGWLVLLLLICYYGQL
LIDESIAVAEDIYYVPWHLAPVDVQKDIYMILMRSQKPLTLNAANIGVMSFPTFLRVISSAY
SYFTLLLNIKS-
>TcasOR76

MMESTVTRLKRMYLWPTASVTSRKPAFFLITFSCFLLYGSVMHLIVNDISMEEVHVIETTA
GQFGVLYYLTLFTIYRKGILEIYADLSNFTKFGKPYNFDKRNKQLNQWSRWFSVVLYFFVI
SVFAWPGIFTQSCEDLNVALNKTEVCGVVSPVWLPFRFDYKPMKQFVYFWQSFCCLYSN
GGAGTISFAMSETIEHLILRVEDLKILFPKIVAERSPEVRRKMLAKWVDYHLWLLSIGKLM
NDTYRYSFSVIVLCAGTLFGCIGYTVMKNASTNFNSSFIFFGWMESVFVICVCGQRLMDA
FHSVGTTVYNSEWCDTDVDFQKGVILITIRAQKPVRIYAGPFSYVSHLLILTVFQTSYSYIN
LLNASS-
>TcasOR77
MKYILMKKTIAFLSVTGFWPKTKESTKTRAFCILFSSSFLLFGSLGYLIVYRKFGSDDIDSIE
TATSHFGVLYFMFFWILKRDGLVHIVNLLSDFSKFGEPRFFNDRNRQLDYLLQYCIFVLSVA
TGGVFLCPIIFVKNCEMVKQEKNLTKVCGLVSNVWAPFDYSEYPMKRVVSLWESYCCFIN
FGCGGIMSFTMIKTMEHLHIRVEQLKDMFPDVVNEKNLAVRKQKLEKWVKYHLHLYDIG
ELMNNTYRYCLSVIVLCVGILFGCIGISTMQPGSSHNSLFLFMGWFQSICILCMVGQRLLD
VFLSVGVMAYDSAWYEKDVDFQKAVLMIMIRARRPVLIYAGPFTNLSHLLILGVLQTSYS
YINLLNAK-
>TcasOR78
MGHAIMTEILTYLTLMGFWPRSPKSSKASAFLIILSTSFLFFGILFYLIVNRQFGSSEIDSIETI
TSQFGVLYYLILFTWKRNDIVEIVELLSDFSKFGKPPFFDQRSTRLNYRLSCIVLILIVANIVV
AALPVIYIDSCHKANEQLNLTKTCGLIAPVWLPFDYNEYPRKHLVFAWEVYCCVMNYVG
SGIGALTMVGTMEHVIIRIEQLKYIFPKILDQPNPRIREQMLKNWVRYHLALFEIGRLMND
AYKWSLSVIVLCVGALFACIGISMLQSTASQINSICLFFGWFPSIAFLCMWGQRLLDSSLSV
GTAVYSSRWYDMDVAFQKSVLMILIRSQKPIRISVGPFTHLSMLLLLGVFQSAYSYINLLNA
TS-
>TcasOR79
MGHVIMNEILTYVTLLGLWPRSRKSTKTISYLIILSSSFLFFGSLLYLVVHRKFGSNEIDSIET
VTSQFAVLYYMTFFTLKREGTVRIIDQMSDFSKFGKPPLFDQHNKRLNYLLSYFVICLFVAI
VGVVALPAIYTGSCHKANEQLNLTKTCGLVAPVWLPFDYNGYPLKFLVFAWEGYCCIITYA
CSGISSLVLVGTMEHLIIRIEQLKLMFPEILNEANRHIREQKLKNWVQYHLALFGIGKLMTA
TYTYCLSVIVLCVGILFGCIGVSTMQSASSNNSVFLFLGWFQSLIVLSVCGQRLIDTCLSVG
IAVYNSRWYDMDVSFQKSVHMILIRSQKPILIYTGPFSYLSHLLILSVLQTAYSYINLLSARG
-
>TcasOR80
MGHVIMNEILTYLTFLGLWPRSRKSTKTVAYLIISSTSFLFFGSLFYLIAHRKFGSNEIDSIET
VTSQFGILYYWVLFTLKREGTVEIVERLSDFSKFGKPRFFDQRNRRLNYLLSYFVLVLMVA
IGGVVALPVVYIDSCHKANERLNLTKTCGLIAPVWLPFDYNEYPRKNFVFAWEVYCCIMT
YACCGIAALVLVGTMEHLIIRFEQLKLMFPEILDEPDRHTRQQKLKNWIEYHLTLFDIGKL
MTSNYTYCLSVIVLCVGILFGCIGVSTMQSASSHNSVFLFFGWFQSIGVLCIWGQRLLDTC
LSVGIAVYSSRWYDMDVSFQKSVLMILIRSQKPILIYAGPFSYLSHLLILSVFQTAYSYINLL
GAKG-
>TcasOR84
MTEEKELRLCLWSCYYLKLSLMWPLKREEFKSSKGLYLRLLVFVIISGSTFTAMIFMHLYK
SLKVGSYDVSEDLAILASNIGYVLMMTMYVSRQKDLELLLLDLSDFKTYGKPPNFDKVR
KRMDLYAHLIFFYSMFGSFVYNMDKIILIDKCKEARRINEVCGSAIPFWTPFETEDLFTLTL
VITYVLINIFVVVKVAMTVSVQVLEISSHINLRIEQLKIFIAGCFDRDFKASRERLDFCIRYH

NVIIDFSERFSRCFSYVMFIHLAITGIIIGCLENQIVQEHQPEAMLHMGGWSTATFIACYGGQ
LLMDASTSIADEFYNCPWYEADVKMRKDLILIILRAQKALFVSTGPFNVLSFALFVSIMKL
SYSIFTVLS-
>TcasOR86
MALNQEDAICSKSCFYLRYSFLWPEEAPTRSFYAKFILVLILSFLTAFLPLFIHFLILVERGLD
PSEDLFVIISYTGFALIMIIYVIHVKKTSYLIVQLSDFEKFGKPRGFDYWDKKFRLISSGVYY
YVLIASSGLNLGRWVGMAECRKERDFQVCGIVIPYWLPWKVDSWLFFILLDLYVLKMTL
VVNCALFLIIIQILEITTHLKLRIDHLKEMLVKCFDSDSQTNRKQLVNCIRYHTYIINCSKLF
KKCFTHAMFSLIVTMALSCGCLESQVVKFDLWALPPISAWIFILFIACMAGQILMNASLSIG
DAGYHSKWYQTDANFRKYLILVLMRSHKALVLSAGPFNILCFELFVAIMKFSYSVFMLLN
QN-
>TcasOR87
MKHVIMDELLIFLTFLGLWPRTPTSPKIISYLMIYSTSFLFFGSSIYLILHRKFGSDEIDTIEIIT
SQFGVLYYLTLLVVKRDGITKIVNLLSDFSKFGKPPLFDQRSRRLNLLLRLFVTVLLAATVA
IVSVPVVFINSCNKQNLQLNATKICGLAAPVWLPFDYTQNPRKYFVSAMEIYCATMNYAG
SGSGAFLVIGTMEHLVIRIEHLKNMFPEILNEPDKQIREKRLKKWIEYHLSIFEIGELMNETY
KWPLSVIVLCVGILFGCIGVSTMQSVSFQNSSVFLFFGWFQSIFVLCFWGQRLLDSCLSIRK
AVYNSKWHEMDVSFQKSVLMILIRSERPVLIHAGPFSYLSNLLVLGVLQTAYSYINLLNAR
S-
>TcasOR88
MTEEKQLRICLSSCFFLKWSFMWPTKSEEFRTSKGLYFRLLAFVIISGLTFTAMIVMHLLKS
VEAGDYDISEDIAILATNTGYILMMLLYIIRQKDLESLLVDLSSFKKYQKPPKFDEVNRKLE
WCTRMVFGYCVFGSVFYNLVKILAIPSCKKSRRINEVCGVAIPYWVWFDTENWSIKLPLIL
HTFLVIIIVDKVTLLVSLQVLEIACNIKLRLDQLNCMLVSCFDGDVEASRRRLNECIKYHKE
IISYSEIFSKCFSIEMFTHLTTTGIICGCLENQVVQEHRPEAILHIGGWITAIFVSSFGGQILIDS
SLSVAEAAYSSAWYEADVSLRKDLILVILRAQKALFVSTGPFNVLSFALFVSIMKMSYSILTI
LQ-
>TcasOR89
MKEAVLQQSKKEMHLLNLWPKGHVKHFRFRYVITLIIVSPFTLGTLTHFINVLKENLDVDL
SGDISVIAVVTGLHFMLITFVWGHKKIAYLWENLGPHEYFGKPDNFEKRCKQLNFYSRLYA
YYCYLGLTVYIIMKNRGGIECRRLNVERNLTEICGLVTTFWAPFDIDFFPFRQILFVDQVFAT
YFIVKGGAAISFTTLEVGEYIILKIKHLKRLVKEVFDDPREEVQRKKLVFCIKYHQYIISIQE
LYDGRYKHCNGCYILMVGIIIASLSNEIMKNHNIEALLHLVGWVFSFYICCFSGQSLLSESL
TIPDAAFESKWYEAPVYMQKDLLLMMLRSQKPLMLHATPIGVMSLSLFITLVKTSYSYFT
LLNQST-
>TcasOR90
MAKDTSPVLRESIEVMKYLQLWPQNERTNLRRRYFIVIFLCSPLHLGLATHLVVCLKDNLD
VDLSANIAVLSAVTGLTYMLIVFVWSQDKLVHLLAKLDTHEIFGTPDNLTKRSRRLNFYAK
LYSYYCYFGIVIYSLVQIIEMPQCRKMNEEKGLSEICGMIVPFWAPFDIDWFPLKQIFWLNQ
LLGIYIIIKGGAAVSITTFEVAQYICLKIKHLNRLLREAFDDPCDVVVEQKLLHCIRYQQHIIR
TNELFNVCFKHCNGCYVVMVGIIIASLLNQILKEKSVGALVHFAGWICSFFICCHAGQAVIS
ESLTIPEAALDSHWYEAPVKYKKVLLLLLVRSQKAFNLQATPIGIMSFDLFIALLKTSYSYF
TLLHKST-
>TcasOR92

MKNQEIKICRATLTVLKYSLIWPSEADEMNPGKWYYIRVVTFILFTCPWVLSVFMHLIVSIR
NNADIHLSEDVALMVAFTGVYYMTIIYVKKQPKVAFLLRDLSYFQFGKPPGFDETERILGF
LSKLTFCYSVMAVVIYNYIKYRQKPECERMNKLKGLKENCGMLTPTWWPFEINYSPAFQL
IFLYIFTSTQVMMKLSLMISFNVLEMAHHIILRINHLKTMILESLDEQDYEASKRKIKTCILY
HLEILGFAERMDDCFSNGMFAHLTITAAICGCLEKQFVDGDNQLGSLLHIFGWILALFLAC
LGGQHLINASETISDAIWSSKWYDADLRLRKDLIFMMARSQVGLYLNVGGFGILSYALFLS
VIKMSYSILAMLTS-
>TcasOR93
MTNLEIKICRATLKILKYSLIWPNEADEMNPGKWYYIRVATFLLITSLWVLSVFMHIVMSII
HDADVHLSEEVAFCVAFCGLYYMTMIYVKNQPKVALLLRDLSKFQFGKPPGFEEKERILG
FLSQFFFYYCVMAVMVYNLVKLLQKPDCEKMNEIKGLKENCGLLTPTWLPFDINYFPAFH
LTFLYVFISTQILMKLALIISFNALEMAYHVILRIDHLKIMITECLDQRNYEVSRRKLKTCILY
HLEILSLSNRLNDCFSNIMFAHLTITAAICGCLEKQFVDGDNRLGALLHVCGWISALFVACI
GGQHLLNASLSIPDAIWSSKWYEADVRIRKDLLFMMAKSQVGLHLNVGSFGVLSFSVFFS
VLKMSYSILAMLTS-
>TcasOR94
MAIKICKFTRKNMQISLIWPREFEEINPGKWYYIRIVIFLITYGVFPFCTFLHAVVVIHNNLDI
RISEDIGAVVSNIGISYMAIIYVQQQNQIAYLLKDLSDFKDFGKPPFFEEENKRLNFWSICTF
IYPTCGASLYNLSKILEKSECNKINEENGLPATCGFIFPIWVPFNINYFPLFHIMLISTWFCTT
MFVRLHLSISYNAFEIAHHIILRIKHLNGMIITCFDCQDYKISRQKFTTCVLYYKQILDLSNR
LNQSFSSIMFVHFTMTSAVCGCLEKQFVDGEYVGGFIHLVGWIISLFIASVGGQDLVNASQ
SISEAIWSSKWYLADIRLKKDVLFMLMRSQKDLHMSVGSFGVLSYAFFVSVLKMSYSILA
MLTS-
>TcasOR95FIX
MVVKESEIKVSRVTRKILQYSLIWPKEGDEINPGKWYYIRIFTFLSFTSLWCIAICMHFIIVL
KDKIDWDVTEEIAIIIAIYGTYYMVLAYVKNQKKAARILRDLSNFERFGVPPGFEEEEKRL
KVYIIGIFIYAFLTITFYNFFKLSQKGACERFNEEHHLDENCGLLSPVWIPFKVDRFPQFELV
FLYLFTCCHLLMKLPLVVSYNALEMVHHIILRINHLKIMITECFDEPEYEISRRKLTQCILYH
IEILEFATRVDDCFSNCMFAHLTLTGAICACLEKQIVAGISRFGAILHFIGWILALFIGCLGGQ
HFINASDTIPESIWASKWYNANLRLRKDLLLMMMRSQRDLHITAGPFGVVSYALFLSVLK
MSYSILCVLTS-
>TcasOR97
MNNQKIQISNMTRKVLRYSLLWPKTNEELNPGIEYQFSVLGFFLVTGVLVLCITIRFFITIKA
VHEVDAEVLAILIASYGSYYMICAHLKNQHKVALLMRDLSVFNNFGKPPNFDKRNNQLN
FVAKLLALYSFLATIFYNGEQLINKTECKRINKEKGLSDHYCGLLAPCWLPFEIDYFPVFHL
ILIYAFTSGYLLIKMAIHISYNAFEIVSNIVLRIEHLKAMILETFENRNKQVCHKKFLQCILYH
IEILDFAARLDDSFFNSMFGHLALTGGICACLEKQIVSGVNVVAGTLHFIGWILALFIGCVA
GQYLINASEILPSAIWTAKWYDADLELKKKVLFMLARSQKSLFIRAGPFGILCYPLFVTVL
KTSYSILCMLTS-
>TcasOR98FIX
MVKKESEIKISRVTRKLLQYSLLWPTEGEELNPGKWFYFRIFAFLSFTSLWCIAICMHFIFV
MKDKPDWDPTEEIAIIIAIYGTYYIVLAYVKNQRKAAGILRDLSNFDKFGVPPGFEEEEQRL
RVYIICVFIYGFITITFYNFYKMSQKKSCERFNIEHNLHENCGLLSPVWIPFRIDKFPRYELVF
LYLLTCCHLLMKLPLIVSYNALEMVHHIILRINHLKIMITECFDDPDYEISRRKLTQCILYHT

EILEFATRVDDCFSNCMFAHLTLTGTICACLEKQIVAGFSRFGAILHFFGWILALFIACLGGQ
QFINASDTIPEALWASKWYNADLRLRGDLLLMMMRSQRDLHITAGPFGVVSYALFVSVLK
ASYSILCVLTS-
>TcasOR100
MSPKDKIKICGITRKVLRYSLLWPVENDELSPGIRYKLTILAFFSITGILVFSISVYSVLEIKQG
YDIDVEDVAILIAVYGTYYMVSAYLNNQHQIALLERDLSQFYKFGKPPGFEQLNSQLNFAV
KVLIIYSFLGTFVYNGTKMLLREECKKNSQEKGLSDNHCGLIATFMFPFRVDYFPVFYIVL
VITFLLAHTLIKLCMHISFNAYEIVNHIVLRIEHLKEMILSCFNERNQTIVQKKLRVCILYHIE
ILDMAARLDKNFFNTMFGHFALTGAICACLEKQIVLGVNIVAGTLHFIGWIIALFVGCVAG
QCLLNASEIIPNALWAAKWYHADLRTQKTLLFMLARSQKELTIKAGPFGILCFPLFVSVLK
TSYSILCMLTS-
>TcasOR102
MQNQSKPCQLDMMDETYLQFFVKSFTYLNMLPEKTTFCTTIQQYYVSVIITITTFPILADL
VSQFYEESISFTSVNENFVALSALFAVIYVSVCFINRKHKIRALIADLALFETFSSKAVITETD
KSVKFYTKLFIVYGIVGNLCYGLLPILGYKKCHESKSVHMTRYGIPCGLVVRFLFPFKFDY
SPLAELVALYEILVCILGTSVVIVVTTLICGVLIHITVQLQCLRKIILDLSQVNDLEILEHKMK
FCVKYHTAILDYGIRTDLAFNQMMLLHITWTGFIISVLGFEISTTDDYVEAFRFFMHLLGW
LGMLFVVCYYGQKILDESLAIADAVYTFLWYKKSVIVQRYVLLILLRSQKPLTLRACGVK
VMSLATFLGVLYSAYSYFTLLLKLKP-
>TcasOR103
MKQALKLADVLGFNPLKNDNLTKLKKYSSLICMISVVVSAILEFVSNFSALETYESAPESL
VPQFQTLAKISSLLLSQKDITELIDEIKYFWKLDQFGDFHTRKLKKIYKYVTIFFYFYTLML
SGACVLFTITTVIFTPEKPLFLCYGGLHGLPSPQFEIYFVVDLAAIVIMSFGVAAYDGIFFYF
AFHVYAEFKLVKVAFKGKSTFIEAVKHHDFLLKYLRKLNEIYSPIFLCQFFSNLLGICFCLF
MLSRSGMPPELTSFSKYFISLVAFTVQTYIFCLIGDLVSELSLDISNVIFYVDWLDDEVYKSK
TARLVIMNKAQSPVKLTIGKFTGMDLRTFLLIVRNAYSFLAFVNNALD-
>TcasOR105
MKPALKLANVLGLDPLRNDNYTQLKKMFCALCIVSLFVSAYLEFFSNFTTFETYETAPESL
IPHFQTMFKMYSLIFSRTEIVELIQMAEQFYKFSQCDERKKLTKLYKRVDLFFYVYASLVAA
ACVLFAIVTLIFKPGKPIFLCYGGLHGLESPEFEIYLVVDLIGIVIISVTVPAFDGLFFYFALYI
YTEFKLLKIAFKTMSGQELREAVKHHDFLLKYIKKLNSVYSPIFLYQFFCNLLAICFCLFML
SRSGIPPEMVSFSKYFLCLLAFLVQSYTFCSIGDLITELSEDVSNAIFYTDWLDDEAYENKT
ARLIIMSRAQNPVMLTIGKFANMNLRTFILIVRNAYSFLAFVNHALN-
>TcasOR106
MESALKLIDIIGLHPLKSDKYSTMRKTISFLSLVVILISAQLEFLSHLSVFEVYNSGPHSTIPP
LQSLLKMATLHFYKNELIDLMEKSKSFWKLDKFGDLYKQELSKLHRLVTIIVYIYIALLTAT
CVQLAVLTLIFRRGKPIFLCYGGLYGLESPHYEIYSILDAIGIGVISIAVSGYDAMFFFFALDI
YTEFKMIKSAFKRHSDQTVSSYNKQFIEAVKHHDFLLQYINQVNDIFSPMFLFQFFSGLLGI
CFSLFMISRSGLQDINTLSIYSAGLLGFTAQSYTFCLVGEVISELSEDISNEIFYTDWLDDEV
YRNKTAILIVMNRAQESPKLTIGKFADMNLRTFIMIVRNAYSFLAFINNALD-
>TcasOR107
MENPLKLLHIIGLDPRQSDKYSTIKKVISFLIVLAVLLSALIEFFLHHNESQVYDTAPQSTVP
NLQALLKMFALIIYKKELIDLFTKGNHFWKLDKFGDCHKQKLTKLHKYVDLFFYVYAVIIT
GAFLQLALLILIFEPGKPIFLCYGGLYGLESPQFEFYAVLDFLAIGVIAISVTAYDSIFFYFALY

IYTEFKMIKIAFKRENCAQFIEAVKHHDFLLQYISKVNEVFSVIFLTQFFSGLLGICFNLFMIS
TQGTRDMKSFSTYFVGLVGYTAQSFTFCLIGELISELSEDISNEIFYTDWLDDEVYRNTTAR
LIVMNRAQESPKLTIGKFADMNLRTFIIILRNAYSFLAFINEVLD-
>TcasOR108
MGSILLLNSVLKKMEKALKLVNILGLDPRKNDTFSKFRSIFCFTILISASFSSHLEFFLNFKG
LETCERAAESIIPQYQTMCKMATFLLYKTEMLDLIKKSERFWKLDRFGDLQAKNLHSTYPI
FQIFFYVYVVILFLTCAMFALVNWIFDTGKPISLCYGESEGLETPWVEFYIVLQSVEVTIIFL
GITGYDMVFLYYAGSVCIQFQMLKMAFAERKMNERQFLKAVKHHEFLLQYVEQLGDIYS
MWFLLQYFSSLFGICFGLFLISKEGLPTEPERLSKYFPYIFSFTMQSFTFCMTGTMLSDWSS
EISDEIFHSDWSDDQVYKNKTARLIVMNRAQRPAKISIGKFLDLNLRSFILLMRSVFSFLAF
VNNILNRIN-
>TcasOR109
MGKVKFTEPLEFLNVVGLNPENCSNFSLFRRVISLGFFLVVITLGLLELLLHFEGLETCSRA
SEAMIVQYQLFIKIAVLLKHRKNLVVLMQKTRKFWPLDKFGQDAKIERPHKLLKAFFFAY
KLIMILMALQYILRKFVSKNGKPLAIAFGESKGLSPKVDHLYFVLHSTSTFVVLHAVTGFD
RLFFFLIGHVLTELKLVKKSYRLTQNRREKFLETVQHHAFALEFVRKLNRIYSQVLLNQHL
SCLFGICFGLFLVSKDGIPPDLGHVTKYVPYVISFITQTFTFCFIGSLLITWSLQVPDAIFYND
WGKNQAYKYKTDKIIAMIRGQRAAKLTLGGFGDLDLESFNLVVKNAFSFFTFVNAMNQK
-
>TcasOR110
MDKVEFSDPLFFLNVIGMHPFKADKFSKFRLAFSIAVYFAVIFSGVLELIVNSQGLETYARA
SDTLIPQCQLVCKIFVLAKYKKQIARLLNGSQRFWDLGQFGARYGNSFGKTHKYLKSFFL
LYKVMLTFTCLQFLAVKIIFKIPKPIAISFGETKGLEPLYDHLYLVLHAMITLVTINLVNGFDG
LFFYFIGHVLTELKMVKVAFGDSPIETNWSEEKRFKFAVRHHRFVLDFIEQFNIVYCTMLLV
QHLTCLFGICFGVFLMTKDGVPPDLDRASKYLPYIVTFIFQTFTFCFAGNLLLSWSLEIPNEI
FYHDWAKKTTYENKLAKIISMKRGQRAARLTLGGFANLDLDSFRMVLKNALSFFTFVNA
MMNKKAVTSV-
>TcasOR111
MEKVRLTEPLFLLHIVGMSPHDSGTFARIRKIFSILVYTSTVVLSMAELFFNYKDLETVIRAT
ESFFTQYGLAWKIAVFVVYKTELAQIIRLCDNLWPLDEFGTGHNFQFLHKFLRRFFLLYTG
NLALLCTQFAVTAFFDDQFKSVMVYYGEKESRSQIYDNFVFTLQVIYLYVGCFVVAGFDC
FFFYLLGHAVTELKMLTISFSCKEIGRNWGYEERFKCSVKHHIHVLELLDKINKVYSVMLL
NQHLCSLFGICFGIFLMTKDGIPPNVDHFSKWSTYIFTFILQVWTYCFAGDQIMHWSLKIPD
EIFYDNYWNKYSLKNGLNKIIAIQRGQKAAGVSLGGFAMLDIESFNVVIKNAVNFFMFMD
KMYKRE-
>TcasOR112
MITRLMAQFAIKGRVGTGGYIMDKVKLAQPLAHLNIIGLDPLKNDRFSKIRTVITVAVFALC
NVFSFSELFLHYNNPHVIVRSSEVVFPFFQNDWKIAIMLVYKKNLAQLIQNTSRFWQIDAF
GKNYQYSMGIKHKYVRIFYLVYRLMLMFSCSQYILLTIGSDRPMILSFGETGGLGSGALLF
YLIFHIVYLLIIFNVINGFDGLFFFLVAHVLSELQMVKVAFSSSKVITFWNHKRRFKSAIQHH
RFVLDYINRLNSIYSILLLNQHISCLFGICFGLYLFISDGFPPDYEHISKYVPYVIYYITQVWV
FCFAGQLIIDWSVNISDEIFYHDWTLNRTYENKTDKLIIIQRAQHAARLSLAGYGNLDLQSF
NLVLKNGLSFFTFVNAVIHK-
>TcasOR159

MRGKTIESTTNPYSSLKKVFIDFAYSKLVISYTKASLTFHVLSLLLEVYYLVTNFSVELICRY
GCMMCLMTYMYSKKLKLLEKPCLLDFWKVYNSSTATQRLISEKSSKTNRRLYCALTCCFF
LAIILFPIWGDLNEFFIFSQVYEKYFTSWAPAFCYFYVSTLLWCCFYCFHLPGIIMYLTLHLD
LQFKLIKDKITEIDKNCSQKEIYQILRLCISHHVALKKWMDKLADLLVTIMPFFFLFGALNSI
ATSFFVLYTLQNTTMILKIRLGTLTLCNFIIVSTFAEVGQIFSGQNNSLFEQLMDCSWYLWNI
KNRKTLLMFMLNCMKPKTFSWGGITLNYSFVLFILKTSLSYASVLFKLRGETF-
>TcasOR160
MSGKTKRITTKTIHLSNPYSSFKKVFSDFAYSKIMIFYTIATLAFHMLSLFLQIYYVATNYSV
ELICRYGPMMCLAIYVVTAKVVGVFYYKTFTMLENQCLFVLWKTCNSSPTTQRLILNKSL
KMNQKLHLALMSYFLLAIVMLPTWGDLNELFIFSQVYERYFKFWAPVLYYFYISTFLWCS
YYSFHLPGCILYLTLLLDVQIKLINDKITEIDQNFSQNEISETLRLCISHHIALKRWMSTLAK
MVNSVMPVFVLLGALSTVAVSFFVLNTLQNTTMILKIRLAILTVCNFVIVSTFAELGQIFSD
QNNSLFEHLIDCPWYLWNVKNRKILLMFMANCMKPKTFSWGGITLDYSFAISILKTSFSYA
LILFKLRGETIRN-
>TcasOR164
MSGKTKRTTTTRKINLANPYSSLKKVFIDFAYSKIMMFYTKATLAFHVLSLLLELYYVATN
FSVDLICRYGCMICLMTYVVTAKVVGIMFSKPFKLLEKQCLFVFWKTYNSGPTTQRLILD
DSLKMNRKLYLALMFYLLLAIVLLPVWGDLNEIFIFNQVYETYFKFWAPVLYYFYISTFLW
CCYYSFHLPGSIFYLTLHLDLQIRLINDKITEIDQNFCQNEISETLRMCISHHIALKSWMSKL
AKLVDAVMPVFVLLGALSTVAVSFFVLNTLENTSLILKIRLTTLTVCNVFIVSTFAELGQIFS
NQNNTVFEHLMNCPWYLWNITNRKTLLMFMLNCMKPKTFSWGGITLDYRFALTILKTSF
SYALVLYQLRGETN-
>TcasOR165
MSDNTKKATTKSLDLTNPYSSLKKVFINFAYSKIMIVYTSATLIFHILSLMLEIYYLATNFSV
ELICRYGCMMCLITYMVTAKFFGMLFSNQFKFLEEQCLLDFWKAFNSGPTTQRLILKESSK
MNRKIHLALTFYVILAIIMLPIWEDVNDFFMFSQVYENYFANWAPVLYYFYISTFVWCSYY
SFHFAGVIMYLTLLLDLQFRLINDKITEIDQNSTQNEICGTLRLCISHHIALKRWMNKLANS
VDTAMPVFILLGALSTIAVSFFVLNTLQSTSVILKIRLATITVCNLIVVATFAELGQIFSDQNN
SLLEHLMDSPWYLWDVENRKTLLMFMANCMKPKTFSWGGITLDYSFALSIFKTSFSYALV
LYQLRGNTF-
>TcasOR167
MAKTGDIFPVRDPVKRCLFIPKLLLESTNFWPEKRNFLTKFANWVMLIICVLIESGQIAFVV
VNIKDITKIASAMSTVSTTFQAITKLTVLYIYNDKLRLILKSVWYEFWPSYTAGREINTKLE
TYNKIVIVSFLTILISGICFAFGFLSSPLISGERILPFETVYPFDWTKSPYYEIIYVTEWMTNIA
FILIGICGHDFLFMGLCSNVVGQFTLLRELFGYLGTKNVAQIIKKLGHDTNIEPNRQLLRICI
IHHVRVTEICKEIAEIFSFSCFIQLLSSVTALCVGALIMTFADIDAALFTVSSAYIVGHLLQLF
LYATLGNEVIYYASRLPNAIFHSHWYNIDLEVKKDILFVLQRAQKEVKISAMGVSVLDYQT
FIQVLRLSFSFYTMLSKVTDH-
>TcasOR171
MVKLFLLLKHLTMKAQDSDNPYIVLRRVFVDFAFTSHMIIYTKITFVFHFLTLLLETYYMIT
NFNVELFSRYGCMMCLMTYSNVQIVLAKLLEILFARHIKFLEEERLSHFWKLEESSEETQK
VVNAESSKIRKKTFFVLSWFVALGFVLFPIFGDLNDLFMFGRVYRNYFGSWAIIPFCIYVST
FPSIAYNSICLPAVVSYFIFHLNLQISLINDKLGKISEKSRQSEIYQKLCSCVAHHVRLRRWT
NIFQNELESALPFYLFLGAINSIAVSFFILYNLQNMTLIFEIRLVVISVCNVLILWIFAEAGQEF

SDNSDSIFDAVVACPWYSWNAQNRKIMLIFMLNCLKPMTFSWGGVKLDYQFTVTIVKMS
YSYALVLYNWRYEK-
>TcasOR172
MSFQALKHLLKMCAEKTPDLDNPYLTLRRVFIDFPYSKSMKIHTCITLLFHFLSLILEIHYL
VTNFSFELSSRYGCMMCLMTYVISVKIFVIMFAKPLKILEEQRELHFWKIGDSSHAMQQSV
ATEALQVKKQTYFALSCFVLLAVILYPVWGHVNDLFMFSQVYEKYFGDWSVIPYYFYVFT
FMSSSFNSFQLPGVILYFTLHLNLQISLINEKITKISGENYCQDEVFKQLRDCISYHVALERW
MARLIDLTKTAMPVFILLGALSSIAVSFFVLYSLENTRFILKIRLTVVAICNVLIVATFAKAGQ
RFSDKTGLIFDAIATCPWYSWNVPNRKIVLIFMANCLKPKTFSWAGITLNYQFAIKIVRTSC
SYALVLYKLRNGNY-
>TcasOR187
MSTKREVVKNFPYYYLFKICIDFGYSNVVKRLNICCITMIVMFHLTQIHYMQENFSKELIL
KYGSGIALGIYTILSMSVQMLIEHEIKDLIAEALFSMWAVDSCGPQVEKLILRRAKVMNIIY
CSIFAWFALMATVMLPMWGDHSEWLLYDPILVEDVKTRLKIIYYLSTFIIFPMIAFSAIRLPG
ILLYGILQIHMQIMLINHKLVQVSEDLDDLNNVKKIDQDDYQERIYKELCLCVEHHIKIKL
WLNKLMKIVQLLMPPYFLLGSINAIYLLFFVVYNDTSNILKVRLCILLIVGGQILCMFAEAG
QALGEETGRIFDTLVNCPWYLWNKKNKQALTIFLSNSFQPYTIAFAGFTLNYSLALALLRS
SVSYALVLYNMRN-
>TcasOR188
MFVKRQVLEGFPYYYLLQLCLDVGYSKMMKIANIFCIIINLLNVLAQIGYIKQNFGKELLL
RYACGIQLTIYTIVTMLFEFLVEQNVKKLMDEALSEMWPIDFCGLEIKKLILKRSTVMNSIF
YFMFAWFAILAIVMLPMWGDQSEWLLYDRICKEFFATWWKIPYYFYFTTFPVVAFSGIRLP
GLLLYTILQTHMQIILINQKLVQISGGLDGINDVRMIDQKNYQKRIYKGLRLCVAHHVAIKR
WLQKPVKIVQSLMPIYIIMGSTIFISLLFATVYSFRDSSNILKVRMSVVLMICCLILCMGAEA
GQALSNETSRVFDTLVNCPWHLWDQKNKKALTIFLPNTLQPVTITLAGITLNYSFAVGLLK
SSASYALVLYNMRN-
>TcasOR189
MEKMFPQIRTEDMKKFPYYYLLKICIVFGYSKIVKLLNVVCIIITSSTIVLQVYYLKQNFSK
ELILKYGCGISLTIYTIASMLVEFLIEQKTKKLLNEAGTILWPVNFCGVKVEKLILKRVTVM
NIIYYFMSAWFALMGIIMLPIWGDHSEWLLCDVISNEYFETRWKILYFACSCFSFPVIAFSSI
RLPVILLCTILQTHMQIILINQKLNQISEQMGNLNNIKLVDDKCYQKRIFEDLRLCVSHHGK
IKKWLNKVLKLVQSIMPLYIILGCLNFISLLFFASDGLQNASNILKARLCVVLIVCCLVLSMF
AEAGQALSDETSGVFDTLLTCPWYLWDKNNKKVLSIFLSNSFQPDSISVAGITLNYDFAVA
LLKTSSSYALVLYNMKN-
>TcasOR190
MSTKKQDLLKHFPYYYLWKVFINFGYSKLTKLVTISCIIIHSSSLFVEIYYIYCNYNKEIIFKY
GCMMSLLGYITISMVVELLLEKDTNNLVCEARSLFWTIDSCGVQAQQIIHKRAVVMNATF
GFILMWVATLGVIMFPIWGDQSEWVLCVKIFENYFENWSQMANFVFFSTFPMVAYSTIRLP
AMLLYGILQTHMQIFLINQKITEISRSKDQEKIYKELCLCVSHHVEIKRWLQRFLKMVQLT
MLMLIPLGVLSCVCVLFFVIYSFLDTSNILKMRLTVVVACTVLIVYIFAEAGQDFSDEISCIF
DTLVTCPWYFWDQKNKKALVLFLANSLKPYTLSIAKITLNYDFAVALVRTSVSYALVLYN
MKN-
>TcasOR191
MRLEIEALKNFPYYYLLKICIDFGYSKIVKCINVVCIIINSSTLFIQVYYVQQHFNKELIFKY

GCGMALTIYTIASISVEFLIEKNAKNLVNDATAFVWPVDFCGEKVKKLILKRATVMNKICY
FMSAWFALMGIIMLPVWGDHSEWLLCDLLSKEYFETRWKILYFACSCFSFPVVAFSSIRIPG
ILLCTILQTHMQIILINQKLNQISEQMGNLNNIKLVDDKCYQKRIFEDLRLCVSHHGKIKKW
LNKFLKLVQSIMPLYIILGCLNFISLLFFASDGLQNASNILKARLCVVLIVCCLVLSMFAEAG
QALSDETSGVFDTLLTCPWYLWDKNNKKVLSIFLSNSFQPDSISVAGITLNYDFAVALLKTS
SSYALVLYNMKN-
>TcasOR192
MVSEQTLLKNFPYYYLLRIFIDFGYLKITKVLSVACIIIHSLSTLLEIFYICQNFSKELVFQYG
CITSLATYVITSMTTGFIIENDAKNLIRETVTAFWPIDFCGPQVEQLIFKRVARINTFNFFLLA
WFAIFGIIMFPVWGDESEWMLCVIAFKKYFPKWWRVPYYVFFATYPMVAYSAIRIPAMLL
YGILQINMQFFLISQKIIQISQKPQNKTHQPGFYQKTVYKKLCQCISQHAEIKRWLQRFLK
MVKSVMPVFIFVGGLCFMSILFFVVYTFQSTSNILKVRLGVILMICNLILVTFAQAGQTVID
ESSGIFDTLMTCPWYLWDEKNKKTLVIFFSNSLKPITFSIASITLNYAFAVALLKTSASYAIFL
YNIKN-
>TcasOR193
MSELEKQLPYYFLMQFCINFFYSKTVKVVTSSCIIIQSLSLLLQVYFIITNFSKELILKYGCE
MSLATYLLTSLLVDVVVENTTKQLISEGHTSFWSIDSCGHDVKNHIIANSARLSVVIYFILA
WFAVLGISVLPVWGDQSEWILFVQIFNTWKKILCYVYLSTLAVMVFLSIRLPAMLLYGILQI
HVQIILINQRIIQIGRENTNDIRMMNQMSYQNRIYKELGFCVSQHARIKRWLKKLLGIVQS
AMPIFTVLGGLIFISVLLFVLYSFENASCFLKIRLGMVVISCSLVLCMFAVAGQAFSDETSRV
FDTLMTCPWYLWDQKNKTILLIFLSNSLQPINFSIANITLNYSFAVALLKTSTSYALILYNMK
N-
>TcasOR194FIX
MAMKQYPFLYKIFLDFAYAKIGKMVTYSCIIIQSLALQLQVYFIVTHFSKELIVKYGPGVLV
VTYLVTSLVVELMIENKTRKIIDFARLTFWPTDFCGLEAKNRLIKNSSKVSIVIYLILMWFA
AQGIVMFPVWGDTSEWRLHVEIFDQWKLFYYIYVSTFTIIVFSAVRLPGILLYSIFQTHMQI
VLINQKITQISQNDPNDIRMMNQTGYQKRIYKEMCLCVSQHIAIKRFIKKLLEIVRPVQPIF
MVLGLLGVISIFFFALYNLENTSNILKIRLVMVVISCILILCLFAEAGQAVSDETSRVFDTLLT
CPWYLWDQRNKKALAIFLSNSLQPISFSMAGFTLNYGFGISMLRNSASYALILYKMKN-
>TcasOR195
MFRERVYDDRFIVLKTIFLEFAYCKEMKIYNMFCLVFHLFSFSLQVHFIVLNFSVELITRYG
CMLTVFLYLIAAKSFSIIIEKQVRMLEMEATSFFWPIDCCGPQVKKNIYDRAARQNIQNYFT
LAWFALFGIIMLPVWGDQSEWFLCIQVFQQYFGCWKLFYYFYFSTFPMIAFTAFRLPALML
YGILHEHLQLILVNQKIVQLSVRRSLKENIVDNANYQKTVLKKLKLCISHHVKLRDSLGKL
IGVIQLAMPVFLFIGALGSIAVLYFVLYIFLSSSNILKIRLVVITICNGLIVYTFSAAGQALADE
TGRVFDTLMTCPWNTWNIKNRKVLLIVMSNTIQPLTFTLAGITLDYKFGLTMLRISCSYALI
LYNLH-
>TcasOR197
MFKKRKFDDRFIVFKKIFFEFAYSKEMKIYNMICLVFHSFSFVLQVYFIVQNFSVELITRYG
CILAVFLYLIAAMSFAIFIEKQVKMLEVETTSFFWPIDCCGPQVKKLIYDRSARINILNYFTL
AWFTLFGIIMLPVWGDQSEWFLCIQVFQQYFGSCWKLFYYFYFSTCPMIAFTAFRLPGLM
LYGILHIDLQLVLIYQKIAQLSARRIFSENIVDNAHYQKTVFRKLKLCISHHVKLKTCLRKLI
ELIQMAMPVFIFVGAVCSIAVLFFLLYVFSSSSHILKIRLAISVVSNVLIVYTFSAAGQAIADE
TSHVFDTLMTCPWNAWNNKNRKVLLIIMSNTLRPLTFTLAGITLNYKFGLTMIRISYTYALI

LYNLN-
>TcasOR198
MPNVTNKRQKRLFSKTRTKSEDPFVMIKDVFVDGGYHPVTKMLNYICLVIHSCSLLLELN
YFVHNYHFDLMMKYCCAMSLMGYIIATMLFAIFQEHSAIDLTKDILSLFWPIDYCGPRVKE
EIVKKATKINRIHYIVLLFAGALGITMFPIWGDQKEWFLCVQVYQHYFGKWSKIPYYVYFF
TYPMLAFSSVRLPFMTMYAIVQIRMQVYLLHQHISEISGEYVYDMKNLQILCDQNYQNEI
YDKMRLIISHHIMLKRWMRKLVHTVQISMPVFVLLGTMTSISVLFYAIYSFHNINFILKVRL
ISVSVCTVLVVYMFSEAGQALSTETTGVFDLLMTCPWYVWNIKNRRILLIFMANSLEPMT
FSLAGVTLDYRFALGMLRTSCSYSLILYKLKTGI-
>TcasOR204FIX
MTNFFSNFCSPLKNHWAKTKHLFSKFSLSSDQPFIMIKLVCVDIGYHPVAKTINYICLAIHIS
SFLLEMNYLRLNFSTDLLIKYGCGISAVVYDISTLIVAPMIERPTIGLSEGITTSFWPIDFCGP
KVKQLILEDTKKTSKIYYRTLVTIFGFAAVIMLPIWGDQKEWFLCVQVYEHYFGKWAQIPY
HIYFLSFMWFAFTSVRLPLMMSYAIKNIRVQVFLVNQKIAKMSKEYEEAKIEDVNYQNRV
YKNLRLCISHHVLLKWWLRKLQKIVRFCLPVFVVIGILTESSVVFYLIYNFKKVNLLLKIRF
LLLACTTGVIIYFFSEAGQSLYIETSQVFDSLISCPWYSWNVKNRKVLLIFLTNSLQPMFFSL
VGFTIDYRFALTMIRTSFSYAIILYNLSSGSQIASI-
>TcasOR205
MTNIFSNFSLYFKNTWTKTKQRFSKTLPSSNVPFMMIKLVFVDIGYHPVSKIINYICLAIYM
SSFLLEMNFLRLRFSTHLLIKYGCGSSLSVYFISSMTVAAMTELLAVDLSEGILSSFWPIDFC
GPQVKQLILKQSRADKRMHYVVLLVFSITGLAMLPIWGDQKEWFLCVQVYEYNFGEWS
KIPYYIYFFTFPWVAFSSLRLPFMMNYAILNLRMQVFLINQKIAKMSNAYDQTTIEDVNSQ
KRIFKNLRLCISHHILIKWWLRKFVNHVKFCIPIFVIVGIATSISIVFYLIYSFQQVNLVLKIRF
LSIACCCWFVIYLFSEAGQSLYEYTEIFHSLISCRWYIWNVKNRRILLVFLANSLEPMTFSLA
GITLNYRFALNMMKTSCSYALILYKLNCDSQIMD-
>TcasOR213
MAKFNDPFKFVRTIIFVDMNSYKVIKTCNVLLNIIYSLIHCLLIYYLCKNLEINLLIRYAPAIL
LFILVIFGAVFSIYMDEDILEVRSVFRENRWSLSVLKENSQTKLGRKCQFINIFILLVLLLIVS
TLAINAPCFGNQRELLICIQVFEEYFGEWSFIPYYFFFLGFPLLYYNFFRLWMTFVYGLLEG
QLQFFILEEYLCGIYETEDSKSWKYLQDSRYQQEIEKSLRLCISHHIGLKKFLKMVENQTL
KVMPFYLVFGVLILICYFSFIINFADTVTTIGKIRMFMTAICMMGVAILLSWIGQQLIDVTSD
IYFTLGGAPWYYWSQKNAKLLLMFLTNCTKNESVTLAGISLDFTLFVSIVHTTLSYALVLY
NLRESSLVSSSQK-
>TcasOR229
MSARPLHLRNFPYYFLKVLVFDFEQYSAGKVLSYFCAIVHSISIFLQMHYLVKNFTKETMF
QYGCVLTVLTYCVVALFFAIASGNFVEKLESEISSFVWPLDICGEDVKAAILKRAFYTSLVA
YITIIAFPIFSVIMFPVLGDQSDMFLCVRVFNEYFTKWSQIPISLYFYSFPVIAFSGIRLPGMLL
YAILITHIQMFLLNRRIEQISELSNQRRVFETLCSCIELQAKLKRLIRNVFQLVYIAMPIFILLG
AVSSVFVLFFVVNSLETASYFLVLRMGCFFGANVLVVFIFSQSGQSFSDETGRIFDTLVMCS
WYNWDKRNKKVLLMFLANSLEPMSITIAGITLDYKFALAMLRTSCSYALVLYQMKN-
>TcasOR230
MREKPLHLSHFPYYLLKIMLCDTEQYRLGRFLSYSCAVIHSISLLLQMYYLIDNFNKETVS
RYGCVVIVTTYCVVALIYEILYAQPSVSMMSQQISTLWPMDACGEKVKQMILKRAFFTSV
VTYSILFSFPIFGIIMFPLWGDQSDMFLCVRVFNEYFTKWSKVPIYLYFCSFPVLTFSGIRLPG

MLLYAILITNIQIILLNQKIAHISDLGDQRLVFGTLCSCVSLQIKLRQMLNKVLQFVYLVMP
VFLLLGALTAISVLFFLFYSLENPSDYLMIRLACFLGGNILVVFTFCESGQALSNDTGRIFDI
LLTCPWYKWDKKNKNILLMFLVNSLKPMSITIAGITLDYKLAVTLIRTCCSYALVLYQMKN
-
>TcasOR234
MQQAALRNFPWHYIKRIFIDFGYHRTMKIFTIVYFILYSGSLLLDLYYLFNNFSIAAMVRYG
CMIMLISYVIAGMLFCFIFEKQLLNLLSEAETIFWPPEMITSELPKFIHRTNVLNYFIIAWFGL
LGVILFPVWGDQSEWFLNVWAYKAYFGSWWYIPYNLFYYSQPMAAWTCVRLPFIMMYF
SLQIKLQIFLLNQQILEIPKGHNTNSETAPDDLSYQEAVSQKMCLCISHNVKIKRWTKSFLR
KVIQAMPVFVLLGILGSIFVTFSVLYSFESTSTILKIRLVVVVGCTILSVYMFVEGSQRLCDE
SSQMFEMLAYSPWYLYNKNNRRILLTFMTNTLEPITITWGGIILNYNFGLTMLRMSFSYAL
FLYNIH-
>TcasOR264
MVYLKDPFITLRVMFLNFNKYKIVKCCDFSFIIFYSLVFCLQIYYLISYFSANPLIRYATTILL
VLWGIVGAILSVTLEKQILEATAFLDEMCWPLNMVRKEAQTKLERSCRIINIYITCSLLLILI
TVVFNMLCFSSQRDFFINIQIFEEYFGELSHVFNGLYFTGFPYLCYHGARLCYVFVYAILQI
QLQFSLIEEYLLQVYEIDCLKSWRYLRDTRYQQEMGKSLRLCITHHNALKKFVKMINDMS
LICMPFCLVLGVLILISCLAFVINFGDTLTIFVKLRILIFVVSCLCVLSVFCWSGQQLTDVSSY
IFLTLARAPWYYWRLENIKILLTFSTNCTKNDSIVLAGIRLEYMLFVSMLRISCSYALVLFN
LRK-
>TcasOR276
MTMQFIVKRATRGIFHDLRVLKFISSDIFDIKIMKLCLFITFLIHLTACAITIHAFMFNNFSRR
EFISCAPVLFGCFYGLLGLGTILFKPSMTRTLMLELKAWDITAADDAVSSRIKFEINVITVFC
LVNYLLALVASFFYYMSFYGDEEIFYLIRFLEDHCPNHKRVLIKLYKISFVLLGYVMVVHA
CQVLYATQHVRFQLILCAHFMANVTKQAKNIKDEHLPDDNNYQNMIRERLKFCIIRHQEIR
RFYFDKLEEMGNLIGGFALLGCFLGISFAMHMLTSEFLRYHFARTVSSIIAGVTTFATVIAA
GQSVETEVDISTRVVKEVKWYTFNESNKRSYMLMLLNSMQTYKIKFSENYSINYELGLSI
VRGVFSIVSVVVQLDY-
>TcasOR277
MDQVLEKFPENDWLRGVKFISSDIFQRKLVKAVLFMVLLVHLTASVITIRAILIKDITAKEFT
FYGPVFFGCFYGMLAIYIILFEKNFIANLSGELKMWSFRSAGAEITRQIRFESRVVTIYAIINF
VMVVIASCLHITPLESDYETFYMIRFFEDKIPDYANVCKTSYRSTFLVMGYVMMVHVYQII
YATQHGKFQIMLYLEYVKRVTQFNEKIGEKCLFYNESFQKMVARKLKNCVIRHNEFLKYH
RKNTREMSHWIVAFSLCGCLLGISVFFYILSGVIYREQYFRVAVLLTTAASTFVAFIVAGQSL
ESRVDNGYSVVSRIEWYNFSETNKKTYFLLLVMLMQPWKIKFSDKYSINYELGLSIVRGIY
SIISVMVNIRFDS-
>TcasOR278
MNQPQESFLKNDYLKVLKLISSDVFEPRLVRAILFVVFAVQLTASIITVRALLIKELTAKEFV
LYGPVFFGCFYGMLAIYIIIFQSSFITNMSQELEMWSYSSGGEEINRRVKFQSRVITIYALVNF
LLAIVASYLYFSPLDSDNETFYMVRFIEEKIPDYAKICKIAYRTTFLAMGYVMIVHSYQVIY
ASQHVRFQIIFFTEYVKKVVEFDEKISEECLFYNERFQTIVGKRLQNCVIRHIQFLKFDRIKI
KEMSNLIAAFSLCGCLLGISISFYVLSGIFYREHFLRVALISVTAVSTFFALILAGQSMESKAN
SAHIIMNNIKWYNFNQSNKKAYLLLLMMSMKQYKIKFSENYSINYELGLTIVRGIYSIISV
MANMHFDN-

>TcasOR281
MDYSEKSLIQGDCLKLLKVISSDIFQPKLVKLILLIVFGVHLVVDLLTLRALLVNELDFKEFI
FYGPVFFGSFYGMMALLTLVLKDDFISNLKQEFRLWPLDCAGDEIYSQIKFENKIIKIFVVF
NCIVTFIGSYLYFLPLDSDNETFYAVRFIEENYPDHRNLLHGLYRSTFLIFGYAMTVHVYQV
IYNSQHLRYQIIIFTEYVASIGNPDKRKENELFYDKGFQKVVYERLKFCIMRHQEFLVISNK
KVGDMRVFIVGYSLCGCLLGISLTFYIFSGKFYREHFPRVSVACVGAVTTFWAVITAGQAIE
SEYDSLLSTLLGKIEWYYFNDSNKKNYLIMLINLMQPWKIKFSEEYAVNYELGLAIVRAIY
SIVSVIASMHFEA-
>TcasOR282
MHDYCFEPLTKNDYLKTVRFLCCDVFEAKIVKLGLWITFGTHLIVSVVTVRALLYDLTINE
FVHYAPVFVGSFYGLLALWTILFRIEMVRDVRKQFKFWTIDCAGQEAHSRIKSEIRITTVLS
VLNFIITLYASYWYVYPIEGDKEIYYALKFFEEYCPRHKMVLSVVYRATFPLLSYAMIVQA
YQVIYTTQHIRFQAILFIEFVLNIGHQTKNLSEEKLFYDTDYQKIVGERFKFCIMRHHEFIAF
RRLKLNEMSNLIVGFSILGCLLLFSFGLFVLTGKLHREHFWRFGLGSLAAVCTFGSVIWAG
QSIEIESENVVNSLNSVKWYTFDENNKRNYIIMLVNTMQPYKLKFSENFSINYSLGVSIVR
AFFSILSVAAKLYFNHV-
>TcasOR300
MIGLTNGDYSPRPSMEGDCLKILKFFAVDIFNPKIVRFFLWIMLLYHVVFTLVTAYFMLYVL
SNSEIIGYTPAFLGNFYPMLCVWSVLFISRLIYVKEDMPLWAIDTAGAKVQASIKRKIFLYT
AFGIFNLVLSLSAGSFYLKNVSEDVNVFLALRIFRDYFPNYYQVLDLIYRLIYFCFSYLMVA
PSYLLIYYILHVRIQAIIFAAYVAHIDGHSDYGTDIDLFDNEEFQSEVERRFKFCIKRQIEFLL
MESKKLSQISNLIAAFSLAGCLFGISIIFHLFTGQLIQEYYFRIGLTSLAAIATFSAFIYTGQST
EVQIELVDNAIDNLCWYNFNRSNKLLYLIAKADLARVRKIKFSGQWAVNYDLGFAIVKGIY
SIISVVVSMW-
>TcasOR309
MPFEWTIRKNKIKPILQNDVLLNLMLVPNTIISNKFLVILNYFYFGFIILQSVFVAVIIITKDE
WKLLNGQYAGYTSGCAIVWSSYITMYTYVDKFLNLYKEIFPHLWSLDVVGQDHFNKFSK
MAKVLKLGKNILLVVGFLSATVGLPWYRDEYEIIITVRVYKDYVDKWTTLLYFVLFSSLY
HIALTVIFCVLCLVYMVLHLHNQCVMLNKRLEALDDEQLFLDNDNYQDFVTKELKFCIQ
QHQFLLKFAKRLNDILYYPTFYYVLSGVVTGVSLLLFPKNDIKNLLRCVLIIVLGGGFAISF
CFLGQILENASEELLFSAYSARWYLWNIKNRKLLSVFLLKTQDNIVLSSSGIITINFRLLISLY
QSIYSCLTFLLNIK-
>TcasOR311
MHHKNIQPMTDDYLKFIKFVSSDIFQLLPVKIFLAVVFLTHAVLDLLTIYFVLFVIEPHDFIT
YISVFLGEFYAPLFAIVMLLFRGKITDSLKHKLAMWTITSTDEKTQSDIKRQIVFFNGFVVL
NSVIISIASWFYAARLSDDVNAFFALRLIHEYFPKSIFEVIYRVTNFVLGQMMCVHVHQTLY
YTQHINIQVQMFKKIIRDLENESKIEQQLKFCIERHAEFIKIITLTTKELRGAFVGFAFGGLLL
GVAVAFYIFSGLLTPEYYLRVGAIGLASVVNFAVTIWFGQSTESHLDELMLAVGEVQWYNF
SQRNKKVYLILLMNVMKGRKWRVSEEYSVNYRLGLAIVRGVYSIISVTSSYKKS-
>TcasOR313
MEQLPKNDPLLVLRVLPELLLLHKIVRHFVVFIVCYLTATTIFCLYVLATVRGLWDLFWSQ
YSLLTFGSVIGFSCYFVAFWKGFKFLELRRRVFADYWALTSLGEESFQKIKKLSKSANIFTV
GTILASIATSSTCMPWVGDEYDIMFPVRVYTDYFGERAVPLLVPFYLAMYCTGFVMIATGF
IFVHFALHLKFQFFLLNRRLDGLQTEPLVNDFSYQNRVKEELTCCIEYHQKLLKVAKEMNE

IVYYPIFIVVSSGIICSVCLIFYMKTFENSIVRGTAMAISGGLITFGFGFTGQLMENESGRLFD
TSVMLPWHLWCLSNRKLYHIFLTKSQYHVSFSSSGIINLNHTLFISLYRKVTSIFSFLMNVSN
KNST-
>TcasOR314
MEQLPKNDPLLVLRALPEILMQHKIIKYVVLFIICYMTVTMILCSYVLATVRGLWDLFWSQ
YSLLAFGSSIGFSCYFVAFWKGSEFIKLRRRVFANYWPLTSLGEESFQKIKKLSIFANVFMV
ATILASLATSTAGLPWVGDEYDIMFPVRVYTDYFGERAVPLLVPFYLAMYCTGFVMISTGF
IFVHFALHLKFQFFLLNKRLDGLRTEPLVNDFLYQNHVKEELTCCIEYHQKLLKVAKEMND
IVYYPIFIVVSCGIMFSVCLVFYMKNFKNSFVRGTTMAMTGTLTTFGFGFTGQLMENESGR
LFDTSVMLPWHLWCLSNRKLYHIFLTKCQYHVSFSSSGIINLNHTLFISLYTKITSILSFLLN
VSKKNHTK-
>TcasOR315
MTLVRKLQAAATNAFEIRIKDDILAELFNWPFLVLDSKWSTKFAVFLTVYCVFETLACALV
YSTLDVNMMGTYAIVIARFATTFCSFFSFFTKRKQYFEIINENFPHFWPLQSLGKSTFNRIK
MRASSVKFYSFLNVVVMLIGAVILISFTQDESEVYLSVKIYKDYVNKWTTGFVMFFYVSFI
YIGLVVAAISFVLTYTAFHLIFQCFLLNQKLKQINDSIVENEQKQAKFDEKYQSFIYKELISC
VKLHQRLILFGKRINHLVYAPLLVYIFGGIVVGVALIYYLKSSVQHIFTSLILLLIALINSTTF
VINGQMLENEAENIYISLTNLPWYSLNVQNRRVVYVMLMQSQKIIHMSASGLVSLNYQLTI
VFFRCIYTGMTFLVNVGL-
>TcasOR316
MTLMRKLQTAIRNLFEIQIKDDILAELLDWPTLVLFSKWPKNFAIFSTIYCVFDTLVCTLVY
STLDVEMLGKYAIFIAKSTIALCSFFSFFAKRKQYHKIINENFPHFWQLQSMGESTFDQMK
KIATTVKFYSCLSVVAMLIGAVILILFTEDESEIYLSVKIYKDYVNKWTTGYIMFFYASFLYI
GIVTAAVVFGLTYIVFHLIFQCFLLNQKLKLINSYIVKNGQKLVKLEERNQNFIYKELISCVK
LHQRLIYFSNQINDLLYAPIFMYTFSGIVVGVALIYFLKTSIQYILTSLVLSIVSLIITTTFVING
QLLEDETENIIISLTNLPWYSLNVQNRRVVYVMLMQSQKIIHMSASGIVSLNYQLTIVLFRC
IYTAMTFLVNMGL-
>TcasOR322
MTFHWITTPLEPILKDDPLFVLMALPNKLIGSKLQALVNYFFFVYMVILPVSCFLVIVATNQ
WQIFYSPYSGYASGVFIVWSCYVSFFIFGSKYRRVYRDVFPHLWSLDVAGEEHHNRLKKIG
KQLRTFKLVLITLAFIGATSGLPWFGDDYDFYIPIKLIVDYCDQWKLFFSIFFYLSFYHIGVT
VLSCFFSLMFLVLHLQNQFYLLKTRLQTFATDSGTSDVFLSMKVKDEEYNRSVTQEIVFCI
RHHQSVLMYCDRLNDLLYLPIFYFTLSFIVTGVSVILFPKYDLQALIRSLFVIVLGMCMTLL
FCSLGQLIENESENVLYSLIEAPWYLWNTTNRRLYYLFLLKAQDTVNLSSSGLITINFQLILT
LYRGIYSALTFFLNFS-
>TcasOR328
MSYNIKLTKDDRLKLLKIMASDVFQSKTVKIILIVVFLVHAIANSLTIYFALHVSDTKQFISY
ASVFFSEFYPMLAILTIIFKGEVVQHLTDDINIWTIDGASKKLQSEIKLKIKILTAFVIINSFSV
VIGGFCFVQQLSDDVNLFFAIRLIRDYFPNHSTILEFFYRMTYPICAYLMAVHAYQCLYYTQ
HINFQLQMFTEIITELTDLKTISLPENRLFYNKKYQTVIEQRLKFCIKRSQEFIKVCVTKNKE
IGSLIPGFAICGLFLGIGITFFLSTGKFTTEYYLRMGVTSICGLTTFSALIWSAQTTETMINDL
VMVINKVSWYNFNQSNKKLYLTFLLNTMKERKIKFTEKYSVNYQLGLAIVRGIYSVISVV
ASKRHH-
>TcasOR329

MNCENQFAKDDYLKTLKIMASEVFQSKAVKVILIFVFLVHAIANLLTIYFVLYVSDTKLFV
NYASVFFSEFYPMLAILTVIFKGQIVQHLTDEFKIWAIDSASKKLQSEIKLKIKIITAFVITNSL
IAVWGGFLYVQPLSEDENLYFALSFIHQYFPNQSSTLEFFYRMTYPILGYLMTVHAYQCLY
YTQHINFQLRMFTEVVAEFAPVKRFLLFEHHLFYNKKYQTEIEQRLKFCIKRSQEFVQICVI
KNSEIGSFIPEFAICGLLFGIGVTFFLSTGKFTSEYYLRMGVTSFGGVMTFSALIWSGQTTET
MTSELVKALNEVRWYNFNQSNKKLYLTLVMNIMKERKIKFTENYSMNYRLGLAIVRNIYS
VISVVVSKRRH-
>TcasOR330
MNYKKQFAKDDRLKTLKLMASDVFQSKTVKIILTVVFLVHFIANSLTIYFVLYVFETKLFIN
YASVFFSEFYPMLAILTIIFKGDVVQNLTDEITFWTIDSASKNLQHEIKLKIKFLTAFVIINSFT
VVMGSFSYVQQLSDDVNLFLAIRLIRDYFPNYSTILEFFYRMTYPICGYLMAVHAYQCLYY
TQHINFQLQMFTEVITELNNSKTSSLLENHLFYNRTYQTNTEQRLKFCIKRSQEFIKICVTK
NKEIGSLIPGFAICGLFLGIGITFFLSTGTFTTEYYLRMGVTSICGATTFSALIWSAQTTETMT
SDLVMVINEVNWYNFNQTNKKLYLTFLMNTMKERKIKFTENYSVNYQLGLAIVRGIYSVI
SVVASKRQH-
>TcasOR331
MNFFQKKLAKGDFFKTLKFIASDVFQSKAVKMVLILLFLIHAIIYLLTIYFLLYVLEPKQFV
NYATVFFAEFYPMLAILTVILKGKIIENLTDEIKIWAIENASKNLQSEINLKIKIITTFVIVNTLI
AVSGGFLYMHPLPEDVNLFFALRLIRDYFPNHYTSLEFFYRMSFPIFAYLMTTHANQFLYYT
QHINFQIKMFREVCLEVKAWKTVSPFENHLFYNKKYQTEIEQRLKFCIKRSQEFVKISVYK
NKEIASFIPGFAICGLLLGVGLVFFLSNGKITWEYYLRMGFTSLGGVTTFLALVWTGQTTE
NITSDIERAINEIRWYNFNQSNKKMYLILVMNTMRERKIKFTEKYSVNYRLGLAIVRGIYS
VISVVLSKYQH-
>TcasOR332
MEFGNYKLMTDDYLKTIKFMSSDIFQPIPVKILLGFIFALHSAVNLVTAYYMLTTFDAKLFI
NYSSVFFGDFYPLLATFALISKNNTVRNLKDELEIWTIDSAGEKLRSEIKLKIKFLNIFVVCN
SLLVLVTGLTFIQPLPKDSDIFFAYRLIHEHFPKHGQALEFLYRTTYVLISYIVAVQPFQIFYY
CQHINFQLQISIETLKKISDWKTLSEDGENLIDNVKYQTEIKRRLKFCIQRSQNFICLHTEKI
KEVSTFIAGFAVCACLLGIGVIFYLISGNFTPEYYVRMGFTSVVGIIIFAATIWAGQSTESAID
EMVTSLNEVEWYNFDQSNKKLYLIFLINSMRERTIKFTENYSFNYQLGLAIVRGIYSVISIV
L-
>TcasOR333
MEFEVKTFMTRDYLKVVKFLASDIFLAKPMKILLLLIFIVQASVQAMTGYFMATAFNAKF
FNNYAPIFFGTFFPLLAISILLLKNKIFHNLKNELKIWSLDNAGEKIHSGITTEIKVVTYFVIV
NSVFVLLANSTLAYPLSQDVNVFFGCYLIHKYILTYGRTFEFFYKATYLVIGHTNTGHVYQ
LLYYTQHINYQLQLYIEFIKFLDEGKTISKNEDDLFNNPTYQTLINQRLTFLIKRGQEIVKFHI
KKTNEIRTLIPAFSVCTCTMGIGVVFFIISDNFIREYYFRMGMVSLVTVSTFAAGIWSGQSM
ETNLNEITTALNEVKWYNFNKSNRKLYLIFLTTSMRERKIKITENYSLNYQLGLTIVRGIYS
VISVIINMK-
>TcasOR334
MDHPDIKPMTDDSLKLIRFIASDILQPLPVKIFLGVIFLFFTVGSNLLMIYFVLYVYDIREFM
DYAPVLFASFYSGVAILSAIFKGKIIHTLPDDISLWALDSGGEKIHSEIRFKARMVTIFVICNT
LLIIGGIILNLIPLSDDLHVYFALRFIHEYFPNHKTCLIILLKASIFPVIPHMLVVHAYQILYYT
QHSNFQIQLFNKVIAEVDFWETPLRETELFYSKPYQKGIEKKLKFCIQRLQVLINAYIVKTK

EIGTLIALFAICGVLMGIGFSLYLFSGKFTPEYYLRLTFMTLVAVTTFSSIIWGGQSTETIITEM
ITALCQVRWYNFSQTNKKLYLILLTNMMKDRKIKFTENYSINYQLGLAIVRGIYSIMSVVV
KMRS-
>TcasOR335
MDHPDIKPMTDDPLKLIKFMASDILQPLPVKIILLVTLLALPVGSNVLMIYFVLYVIDIREFI
DYAPVLFGGFYPSLAILIAVFKGKLIHNLQDEIKLWAIDSAGEKIHSKIKLKVRMVTIFAICN
TLLLIVATVHNLIPLPRDLHIYFVLRLIYDYFPNHKTYLLILMKLMSPVTTYMLLVHAYQIL
YYTQHINIQIQLYNKFVADVDFWETPLCEPELFYNELYQKRVEKRLKFCIQRSQHFVYYVHV
AKIKEIGILIALFAVCGVLMGIGISFYLFSGNLTPEYYIRIFIIALVGATTFSSIIWGGQSTETIV
TEMIATISQVRWYNFSQTNKKLYLILLTNMMKERKIKFTENYSINYQLGLAIVRGIYSVMS
VLVKMYSINT-

5.GRs

>BmorGR7
MVLEAHTQIQYCTAKANYCEFHAGLRHLMRLARWAGFFPVQGLSQTNPDDVRFEFRSLY
ALYHAITVIGQTVMTFLAFYSFVDSNVSLSVVSNFLFYFTNYVTLVLLWRLSKNWSALISK
TLEFEQSVTEIRTTRNLVSRTNTLTYVVLIFAMIEHALSKVFNIRSVMCCLGETSLNHTVINN
YFKFKWKFVFDYFSTSTTYSYFVGFIAEFLCMQATFLWSFTDVLIMCFSIYLSSFFEDFNST
VSSFMKKASKTVPWSTLRVQYSQIVLIVKQMDEQLDYFVLISYFTNLFFICFQLYNSLNRIY
DANDVCNENMDIIATASVTYLTYYVFSFLFLVTRALLLSIMAANVHSCAQVPQLALYEVPT
ADYSLDVQRFQLQLRYTTVGLSGVCFNVTRGMILRVIGTIVTYELVLIQLTKKNLDNDTSI
RDYYLPKHLI-
>BmorGR8
MAPRSVRSMVGTSKKDMLKGGFYETVRIPLYIYRLIGILPISGLWHRSSKYNRFSLKSFYTII
YAPTIVMQTFLLLVHIYDLFAFFFGHQRLGRLIYHMNFYTITILIFMGSRKWKNVIKEIETIE
LTLPRLRNSKKALALTKSFVFAFFVFSLAEVVLILQFTLRLTKQRHVLPGDSGLYLRSYFVY
IFPYLYDHFPFSYVMGFIVQIIKVQGIITLNMVNCSVVILSIYLTNRLKHYNRIVFAKGSKTN
NTRLKWVELNLLYTRISNLVKIIDKNLNPFVFISFTANLSYICAQLFYILNKLTSSRTVKITSF
LEDKRSDWETVLYISISFALVVLKVLLVSITAAEVHTTSREPLRLLYTLPTAEYTIETQRLMT
QVYYSNLSLSGLNFFHITRGMLLGMVATLLTYEIVLLQI-
>BmorGR9
MPPSPDLRADEPKTPCLVGGAHAFILKISSFCGLAPLRFEPRSQEYAVTISKGKCFYSYILVT
FLVICTIYGLVAEIGVGVEKSVRMSSRMSQVVSACDILVVAVTAGVGVYGAPARMRTMLS
YMENIVAVDRELGRHHSAATERKLCALLLLILLSFTILLVDDFCFYAMQAGKTGRQWEIVT
NYAGFYFLWYIVMVLELQFAFTALSLRARLKLFNEALNVTASQVCKPVKKPKNSQLSVYA
TSVRPVSCKRENVIVETIRVRDKDDAFVMMKTADGVPCLQVPPCEAVGRLSRMRCTLCEV
TRHIADGYGLPLVIILMSTLLHLIVTPYFLIMEIIVSTHRLHFLVLQFLWCTTHLIRMLVVVE
PCHYTIREGKRTEDILCRLMTLAPHGGVLSSRLEVLSRLLMLQNISYSPLGMCTLDRPLMV
TVLGAVTTYLVILIQFQRYDS-
>BmorGR11
MKPFRFFLFVENVICVYRNYSFHKRYARAIILSRVMFEVSLIILTLHSCRNFGAVKYKTEIIFT

YLATASSTILILLALYKTNRFTELFLNFKAFYRNRNLDVDHLEKWNRKQKMATVIIVLFCVI
KFSTLIYTDLIGEYSTPCRGYFTEYLFYTNLFMCNARYLFEFSTACVVLHLVSEQLDYIAIS
MDCTMFLYIDISKKNIMSSAKKRKLKYFDIFKQFEKWTDAYMNVKRSANLCDTVFRAQL
AIMITTITLYYIILLYGITSFNIERGKFSVVKSLSYLISLFGFLIALLLLSKAGQRIQKSAENLR
RKLSKFLLHSLEDPEFHRAATNLLRLVCTHHIKMRCFGFIDIDMTLLPSCLMFVTSYTVIAL
QFNNVV-
>BmorGR13
MEDSFNRLLSIRNMIIFQNVCGFYHMCTEKLYISRIIKMYCVALAIVLSVFCFQNPDITYLS
WDVVWVTFGYTLNVIICLRYNGNYFFQYWNGLHEIDIKMNLTSIDKEKVPISRAVFTVFLI
LRSTAFAMTIFVFGYLETGILSNTIISIYSINLTEFYRNMSNIPMILMFETFYVRIKILKEQLCS
ELSTVLGCNNDARQLKLILKYLRNYRSLVRHLMDTTLPFKILILVILVGSFLRSLLIGYAFV
YNSDQIILLSLPVMFSTKILSEVVEIKLICTKELLKNKNEGLVLLDLDSKKPTFLTSKACGEQ
LQDALSFLNNRSYSYTLLQVIEFDCSLAFVFTSFCITHLIVVVQFTHVLD-
>BmorGR14
MNLHKNIIPIRNNLFANKVTAIALPKTLSVLFKLIHIFFLLDLGVYEYKTFKIKCIVKFLTISG
SLTISVVCFSFMVSNLSEHTFVGWYGFFISTYIFVVLFFNLSNRMTFVEFYKTLLRFDANYG
IDSNEYKFNFKIIFVNILFIANRMVLSFVYCSYYPQNCIRPRYAQILFMLPWLTLDVLLTTNM
FLFYATYCRIAKFPMLIKNSMNIVALRNSYKLIVDSLEKTQTSFDIVFIIALVFSVPEIMMSIY
STLLEVISKHFLEVASILSLNYVAIAQSLLLTLAPSLCAGVLPWKTNNIKIILHEKLFTEKDK
ASAREIELFIKYIESRPLKLRACNLVPLDFSLTIIVLNICVTYLIVIIQFTHLY-
>BmorGR15
MISSSDINHKRNKVFAYNVPGIALSKTLTVLFKLLHYVLLLDVGIYEYKTFKNKCIVKFLTI
ATGVSVSIVYFCLIATVLRKNAFFYWFYVLFISQYMIIVFIFTLSNGMSFTDYYKMLLRFDA
KYQINSNNYYFNIKIILVIIISILNRIGMAIIYCSYYTKNCYEMSFSQIIFVLPWLTRDVILIMN
VFLFYVTYCRITKFPALLENTKNVGSLRNSYKLIVDSLEKTQKPFDFVFTISLVFNIPEIMLSI
YFTLLQVIHSHFLEVAPTLSISYFSITHSVVLILAPSLCAGVLPWKTNTIKIVLHDKLFLEKD
KNSARNIKLFIKYIEARPLKLRACNLVPLDFSLPVIVLNLCVTYLIVIVQFSHLS-
>BmorGR16
MIMNLTTDRISKRNKVFAYNVPEVTLPTTLKVLFKLIQFTLSLDFGVYKYKTFKMKCVAK
VLTLAGCLAASAACVSLIISNIFENQLFFGWYTLFVCQYTIVIFMFTFSNGMTFIDYKMMLL
RFDAKYQIDSNVYHFNIKIVLVVVISVTSRLFLCAVYCIYSTENCIKPWYNQLLFFPWLSLD
IVLIMNMFLFYATYCRLAKFPSLFENPKNVVPLRNSYKLIVDSLEKTKKSFDAVLIAALIFNI
PEIMMSIYYTLFQVMNKHFQEVAPVLSLSYFTIILSVLLILAPSLCAGVLPWKTRHMRLILL
EKLFAEKDKNSAREIELFIKYIEARPLQLRACNLVPLDFNLPVIVLNLCITYLIVIIQFTHLF-
>BmorGR17
MGFSLGTTALSMFFFEKPVVFTIIQITMIIVKPAKYKLSDPFRPKDTSKLSESIIMYFKLFHIF
LGIDLGGFRYQNRQVKYAVRLISLIQPLAIYGLCIYALLKIIANTEFLWYTISFTEYVAMSVAI
TLFSNEMTYCNFMINLKFIDTKLKIGDESFRIGVKLISSTILIGVTRCFTTTTYCLLGFCAKP
TAAQILFQIPWLTIDLMLLQYMFIFYACYCRLVKILRILKKRNTDIEEMRRIYKTLVDVLDR
ARAPFDLAYLLGLLFSIPDVLYSIYESIIKVGEINTAKALSMSIIYITNIQSLALMFAPALTAGF
LPSLTMKMRIILHDKLLEEQDKKTYRHIVLFIKYIETCPLKLKACQIIPLDFSFPIIILNIVVTY
LIVAIQLTHFL-
>BmorGR18
MRRSTKVISMVNQSDKGEIKTCSRFMKIYFFVIYILTGFNFGFYTGRGLNFLRVIQASVLLL

RFIIASNCIYIAFHFRLLEAIWYSLTFSESLAIVVCFMLSRSALSCKNLFEYLYSVDQELKKS
VGPSIEVKLALYTVVVSVLRLTVYVFCAIAYYETLHEGFCVELVYNTPCYCSDLYLVIHFTI
FHSVYCRLKALRISMNEKFDVYKGTLIYKSLIDNLEEIKKSLDVPFFVILLNAVAIAMINILV
TLEISYGQTMKFIRTAPRYLETVLLFSSAFAPVLAADMMASEAQKIKVTLNNILQRDDSLL
EDDRRKVKQFAGYVSARPFRLRACRVLSLDCTLPVTVLSICVTYLIVVVQFTHLY-
>BmorGR22-like
MNRHDHRFSIYNPKRNEAMWKRELFVNNEGKDIKDFQIKDIYGPEITDKDGALLDKHDSF
YLNTKSLLVLFQIMGVMPIMRVPKSIRTTYNWISKATLWAYLVWGLECIIVVKVGQERLAN
FQIGSNKRFDEVIYNIIFLSILIPHFLLPIASWRHGPQVAIFKNMWTHYQLKYLKITGKPIVFP
NLYILTWGLCIFSWVLSFAVVLSQHYLQDDFELWHSFAYYHIIAMLDGFCSLWYINCNAFG
TASRGLAINLHKALEAEHPALKLAQYRHLWVDLSHMMQQLGRAYSNMYGIYCMVIFFTT
TISLYGALSEILEHGLSYKEMGLFVIVAYCMTLLFIICNEAYHASRKVGHEFQDRLLNVNLG
AIDRSTQREVEMFLVAIAKNPPIMNLDGFTNINRELFTANISFMSTYLIVLMQFKLTLLRQG
ARKTVTAIVRAIFNTTITDNGAGGSDEDQE-
>BmorGR26
MNKTKIYRKKLDKNERLVCSVQPAMFARLIVGLYYDIKVSNRVKWMIKSYCISLSSFICYL
IIFRDDNFSLHPKLTSVMEYITYVTFSFLTCDKYLFRYLRFNPRTDGYPIFLYLCKKFEKFFKI
IICLFVSFKILGVVLMMQSWPILSTPKYIWGTLALHFLWLASHMGRLVFILVYGILFCRMRT
IRIIFENRGFQNTPQNRLTPKRYILMYEAVLNSIESVDFPVKFLIFTFICCFAPKLVVSLFEIME
EMKKGELSLTTFIWFLVELSPSYLFLLLSAIALDLVSEDVQELLSITIDRRLNCKNEKERSEI
QEFFQYLRNNPFNYTLWQVVSLNLRTLLVATSFSIANVIAIMQIKNSKI-
>BmorGR27
MVFKYKIMTKAPKSLPVLKILMLFRLVFGNYFRLSSNRYINFLVKSYCSTFTILLSVMCGK
RLKNDSPYMLSLTEYILNKILNYATSEGYIFKYCNSIKTCDKIMGFKKLPIITIDVFIAIIITVIT
RTAITIYFGFLFPFDKYQVVLYVGCIVFSNDLNSLTIMNVFGLLNNRMNLLRKSLEAMTVPI
NIIGKNEVAPKVRLVRNAFRYYSNLLDNLDSVNHCVQYSLSVTLLLKFPKAVLLCYDSIKT
YFVKIDNNFAMDIVDPTEIILSIVVMSFPAMLCEMITNEVEKIKAILTKHLIQCSDNSLRFEL
NITLLYICHRPFKYILWRAIPLDTSVPIGIVSLIITYVIVLIQLLHFST-
>BmorGR29
MYLRSKKSRFKLFSFERMIKILLMICGHYVQTDSSNVVSSIHRIFSIVITICLCPYFQFNPFFF
HVIESVWYSILSQFTQYGFFFRYCSTIKTFDLLSGFKQIPLYTKRVCFFLLITLLVRLIIVLIHF
SAHQTKLKTFCAFLIILSANTGHILMTIMFSILNTRMTLIQKLFANNPIPVNIVGKNQNASHI
KRVRKGLICYNNLLDTLKVAEKEIQFTLTVTYLCHVPTIICYVYFVITVIYKSKFSGYNLIPM
LDMILACMAVTAPALFAELTKNTVDKIKKILGSQLLRCSDESLRYELEITLEYVIQRPFSFSI
WRAVSLDASLPVAMTSLCITYVIVILQLTQLRP-
>BmorGR30
MYLRSKKSRFKLFSFERMIKILLMICGHYVQTDSSNVVSSIHRIFSIVITICLCPYFQFNPFFF
HVIESVLYSILSQFTQYGFFFRYCSTIKTFDLLSGFKQIPLYTKRVCFFLLITLLVRLIIVLIHFS
AHQTKLKTFCAFLIILSANTGHILMTIMFSILNTRMTLIQKLFANNPIPVNIVGKNQNASHIK
RVRKGLICYNNLLDTLKVAEKEIQFTLTVTYLCHVPKIICYVYFVITVIYKSKFSGYNLVPLF
DMILACMAVTAPAVFAELTKNTVDKIKKILGSQLLRCSDESLRYELEITLEYVIQRPFSFSIW
RAVSLDASLPVAMTSLCITYVIVILQLTQLRP-
>BmorGR33
MCYTNFVSRQVSKCIHFFSTIRYIIYLRMFCGLYYNCSSSFKIRCIARLYCFIIYCLNLHYNS

YIFTTNVSLTNFFHTFIILAEVSVHILFSLYTGESNFISFCIEMNKLTSDPNEFIATKCVTTHFIA
YLVIVSHILSSTLICGARASCFTFSVILTSMTFLTTLLSRFTTIIMFDVVWIRMRSLRKILVNA
LESDLAENEKAKSIESFLNAYKQIIASTRITKLATRNLVIFNFVSMFGRIMTLIYFCINNPGYL
DTYHMSLWIFGILLAGFVTCAPPVLVEMNVNELDEIKYALADQLVDYTDDNYRTAIYNAL
DYVEVHSIRYTLWKNFPMDLTMFFGFAGFCATYIIGLLQFTY-
>BmorGR45
MKSPEYLSKDILDEDFVRVFSFPFLVQMALGSCRVHLKARFITVPTLGQKLYTVMCIIICSL
MYFNMTKLYLPLYYEHSIVYYIFVTVTGLDQLSFFANLIHLRFLNGETNTAFYIMMQRIDR
NMKIDHNNIFNKTVTLANILTITLIILHYVGLVISTIILKEYSLLSLFGLLYGQLMLMVEMAL
CSNLIIFFFMRVRFVNAIIKNHVHPENQNQPPKLVRYFITNRITRYLAAQTHDFIVNDTDVY
LKQIFEGFSMFIDIYRFQVCPLCIKLVVLTLLNFEFCLVAIQRNVLGPNHIGNYYIIVNSVMG
FFTALYVSGRCELFFREIRETKRLSVAVLLQYQEGPLREKATRMLKIIEESTPQFSIYDMWQ
MDGYTFVKICSLVTNLIVTLLQFAYL-
>BmorGR46
PFLVQMALGSCRVHLKARFITIPTLGQKLYTVMSIIICSLLYFNITKLYLPLYYQHSIVYYLFL
AVTGLDQLSFFANLIHVRFLNGETNTAFCIMMQRIDRNMKIDHNNILNKTVIRANIFTITFII
LIYVVLVISTIMLNEYSLVTLFGLLYGQLIFMVERAHCSNLILFFFTRVRFVNAIIKNHVHPE
NQNQPPKLVRYFVTNRITRYLAAQTHDFIVNDTDVYLKQIFEGFSMFTDIYRFQVCLFCIKI
VVLSLLTFELCFVAVQRNLLETKNLTNY-
>BmorGR47
MKIRFLFGFYCDFPFNKRFQNILKFYCISVLVVLILGSWACSTGFRSDKKIVIYCEYIAYFLIS
LSTKDRYIFDYYKQQPLIDGSTTSKVLYKKLERLLKYFVTITIVLKMLNIFVFCGWNLTKCI
NELDGVLFINLLWIGLLLARLSLPVIYGLLYFRLRVLRMTLESKGFSNSPQNRFTPKKYITIY
EKIMKDLLKMDYPLKYVFIIFLIGSVPKLLQNSWQFLNSLKNYGPEISKILEFTLECLHSYIV
IILPIVVALDLSEDEIKKMKIITLNKRLACLNERQKMEIQQLFLLLKNNSLRYNLWRVVPVN
LKSVLIFLSF-
>BmorGR50
MAGIRTISSKVKPLELPDVSENNFADDGLKIVQPFKFFIYIQAITGINRLYLLKCNKFVLMFS
YLYAIFLISFVALVYWTTEPKKNSHLVIRLFTFFEYTLLACISVFLKKKKMIKFFENLSLLDK
MLKINKNVNSTCCMKQVFFWVTGSIVYNLIEFYAMEFYDNTNKGLKTIICTYAIALAHDC
EQIFFFTLQRVVYLRLLVVKRHIQEYFKVDEDSSRKKPNKYEMLSNNVQLNLTALHEVYA
LLHNCAEKLNTVMSIPVLLMLFTSGLSTTILLKFFVRVIQLTDPSNPGSAIGVCMYLIVRCIK
YTLLVVISCYYSSITATQVSLIRITIHDAINTVPLGKLQRRKVKAFYLMTKEYSFVYALAGVI
KLNMSLPLSYISLCTTYLVIIIQFSKFLD-
>BmorGR51
MAMGIRTILSKVKPLELPDVSENNFADDGLKIVQRFKFFIYIQVLTGINRLYLLKCNKFVML
FSYLYAIFLISFVASVYWTKEPMKNSHLVIRLFSFIEYILLICISVFLKKKKMMKFFENLSMF
DQILKIDKNVNSTFCMKRVFFWVTGSIVYNLIEFYALEFYDNTSKGLMTIICTYTIALTHDC
EQIFFFTLQRVVYLRLLVVKRHIQEHFKVDEDSNRKKPNKYEMLSKNVQLNLTALHEVYG
LLHNCAEKLNKIMSIPVLLMLFTSGLTTTILLRILVRVIQLADPSNPGSAIGLCVYLIVRCIKY
TLLVVISCYYSSITATQVSLIRITINDAINTIAFGKLQRRKVKAFYLMTKEYSFVYTLAGVIK
LNMSLPLSYISLCTTYLVIIIQFSKFFD-
>BmorGR53
MAHIKDENQSKQQQKEHETLNKNKLKKVVYTLKPALMLENWFGLSDFLLVNEDELVLL

MQTEKFGVILSIFFIVMFAVFVDFPDTETESIMELMDEVPSMVVLSQYFIASITTSSCLSAIAI
RIFETFADLDSMLLITTTQDFYNKSRYQTNKYLIILGVSHIISSTLDLLTDDEIVWCKFFVLPI
YFLQKLEVLTFCKLIVMIQCRLQIINKYLTNFIEEQEKNKALVFTLAESNPKKTDKFNWIGC
PSPNNMKIRDLATMYDVIGTICSLINDLFNIQIFMTLVSTFTYIVIAIWSTLYFYRAPNFTFGT
LTTIIIWCITIILSVVVMSFVCERLVSVRNNTKILVNKVIMNYDLPKTMRVQAKAFMELIES
WPLKIMVYDMFSVDISLMLKFISVATTYLIVIIQLSHFV-
>BmorGR58
MSSRRVLYRAEVLLSNNVDAHVQDMLKPLNFFQFILFFPKYTIRDGYITPNSLIRNIWSATG
AFVFISICVFRILTMNKIAVYDTFTTMLLISKYFDVALYCIGFIVNTYVNIAYSNVNVLLYLK
LQTIKTFIPRNNEIMKNVKWYSVILIIVLFCGTLAMFSFFHLSFSYFNIFDLTTDLAVFSFDLN
LVYACSVLNFLAQSLDELNKEIWRLGNAKVTVCKDGSKPDWNGINLTYINVLDAYNYFK
EAFRLLIFFHTFKTLTHMFIYIQSIIELCKKFYPGDDYDAITVGAVVGVWFFRNITLQCLVGV
SCQNFYSATSNTESICAVQVGSIVSDEHKLFLKAVRRLNNVVFYKWSMYGMFIVDATLPR
RLIELIATYTVVFLQFAFK-
>BmorGR56
MKKIRLLRSIVFLENLLCIYRNFLFFNKKARAIILIHITIELVLYVLSIVNNSFIIYSYFHSDNR
SMLIVFTTICCFYVVTFVSIVMGILRSEEFKDLVTSLELINKFFTNNKTYLKSLGRSNTMIIAI
TTILYCVTCIGIAVDKITLNDFYEFTSSDVIWTVSSTLLELRYQTECVVYFGIEYLFLIFTKHL
NLLVKEAIKKVSLDNNGTVKDVPISSDAVTKNEVKRWATIYRQLMMSSKLLQACFSLQIIC
VFVSAVINFITTAFRMVKVSVLGSIATDMNEIIIVNLIFTLLYQNIGLVLIIVTGQRVWNQILL
LNVLLARLYNGILIQPCRDTLRTLKNLQRMVVKNPVQIKMLSVLPVGSYMLPMFMTLSVS
YIIVMLQFGHVV-
>BmorGR57
MEEIKAIKLVTFIENCICVYRNYAMCTKRNKKIISLRIIVEIIIVFFVNINNILLLHKYYNGSGL
LYIIYLFLVVYYINYMFCIFYGALQGKAYRQLIFCFNKINAIAKRDKSYKKSLARLKNMCIV
ISIALLIISALSVFVDRSNSWNIYEVSLRDSLLILSKIHMDFFYHFEYVVYFTHIKIFHLTLRY
LNSRVKMAQFEMKMTRRDVHDEGERNIRILLTKELTTEWAVLYKCLVFGTKTMKSLFGLQ
MLIAMVMSFVNFTLSLYGIILICSIEQSQTASQHNLLLILTYYTATMLLIFIVAQSVYNEVEM
LKRNLARMYNILAVDSDETQQKLVKDFLRMVYKNKVEIKMLSIFPVGMPMLTFFLSLSAS
YVVVMVQFSNVF-
>BmorGR60
MLTPRSDLCNEKLSPSFPSGKTTAADKDDTEARCQVDSSLERLLLPFNLVQHVSFIPMYSIR
RGLVSPDGPLAYLYSLLGFCLFTSVSVYRNAIMHGTRLSSLHLFTLYSDLVSFVINYSLSLIC
NVVNSKSNVEFVCRLQRLQTVLRRNQREQEQFARSNWAHLAVVTALYLAVVGLLNVVVL
KQSLPDTLYLLLLFCIDVNVLYATRMLALLRCYLQLWTRKINEKAFNPVHHNMFTAYLDIL
QEYEVYTTLFKKIITYYVLETFLHGLLYVQVAIQICKSIRRSGRFSEQLMMIVSIFTWTIKNM
IIMTLHNVECEKFYLAVEQAVAACQTQRASTTRCREEKRLYKNVCRVSRAAFSRERGWGL
LAAGAALTLRFMDLATTYVTVLLQFAFVSRT-
>BmorGR61
MSIRFEKDLLHNYVEIELQYFLRPFNVMQSLFFQSKYRIVDNFILPNTLFKNIMSFVVSVLC
ALSFIYTIISVWQNTHATSFHALVTSVYLSYNIYGILIGSVLIIWLSDRNIEFVLKIQDLIKILE
FNKCFLIEYAFINSIIMAAIFILNFLLYGYFVVHLQKFALGLTFSAIVCILNQDLDIIYVIIFANI
LKKCASRWTVEARQKNNFNDQGKWVKLFNAFLNLTESYQLYQKIFEFYELLRRVGIVFLG
LQLTVCRVCSNDIKSIQCTVMLHAFQLICVWIVKKFITLSILSFEMEIFYEKLREIETVCIILV

SSDNPSERELKIWKNIIRVSSCSVRKTTACGLCEVGAALPQWLLQATTAYTIVLLQFHITTFS
RATNDIYDLD-
>BmorGR62
MNDLFLSKIVKWTKTTKYKLDDDFQSLFRVFNIAQAMNLCPKFLIYDKYITNNAWFIHILA
ISSFIVLVCLDSFFANFRLVLSEAMGPPFYGFSFYFISILYENIGVIIQITMNGYLTKNNVLIIT
KLQDTFKDFRTTDYITKSNRWTNWFIFFIYMNFIANYSYFNFYVNTFSFHKFCFAFIKMCF
DLNIVYTIFIFKMIGDSLTMFKDTAFCSKNMKLYEVSNRVYWNKMLRLYSNILDVFELSKR
TLNFFIFYFVSNILLRILSHVQLAILMNSINWLQHVAYSNIVMVLLTLAKEGIILIVLIAKCEK
IYCVIGDVQTACQLALGNAACPEKRRFCKNVRRSSSAAFSKIYICNILAVDAKLAVSLMSV
TTTYTIVMLQAILIK-
>BmorGR63
MQIGNAVIHLKSTKLTTMNTISPTTKLLKIFALNSNIEEIDLKCSTKLRITMTAFVLCSLIFYS
LYYKFIYVFDYVNISIKITDCVQMVYDFCQYIVDLYFVTNYGRNISSEYFQQYKIIDKILEV
VCYEIIKHRIVKLLWVFMCIWFSSSCFDFIAWFLNYGWITPLVYSVAYIFLLIKILTTLDLSAH
IMNVEIRLKMIADLIHHYYMSCEDNFQAEETLCHKNWLNSKERAKYYELQFRIHALKQLS
CNNNEIKLLSRCYLMLTEQVEIINRMYGFRILLNSLSLLIDMVRFTNISVRIMIGSQNLAYN
CGYFPAVSSIFRLLTCGAVIINLVSHCERVYYQRTRICNVIDHMIVNKNLSRESTEALQEFRN
LVQNHPIEFNMANFFQLNYSLLVSIASVVVTYTIILLQSVN-
>BmorGR64
MKISLRKIVSIRNMTLIQNMFGFYHKFTDNRAIGVLLKIFCGFYSLFLSFLCINCTPRFTNDF
LTYDIFFFVIEYLTSVLVCLLYDGQYFLNYLYDLKLIDREAGIEESLEKLPISQPLFSLIFITRVI
YLLSCLLMFDGIKDSLFLPAQSSVFGANFTEFARTIGYFPRVIMFEMFYKRVNYLKSQLRN
DLAHANLYPIGFVCSKVIMKYINFYKLLLRNLQQNSLQFKILMSMSSLYIIIKALASAYAFIY
REDGVHVFIFIEFATGVFLFFVMSSIIISIFNEIEDIRQIVLAQLRYCKQGANTKRVQDALTILN
IRCFKYALCRIYTVDFTFILRILDVSVTYVIVLVQFTHILD-
>BmorGR66
MKRKLKKFFPNKEYNNIVEATHLWKLIRKLTGLSVLTLESKEGNRIETRFSSLGFVFFLLWF
TIYFYCTYKAHNEDQTILRNIYSTKLQRYGDDFERITSIIYVLYSMWKLPFQISGNRLLLQEI
VDIDKAIESVGVTIDYKKNATFALFIYIGQIATYLFRLFCVWGCLGNLNSPVPVEKLYQDIF
TDALSLLLTSQYCFSLVILRDRCRYINKILCGIENRESSRLRLFVYSSMPGAEKDITCRKIKD
CSKIYGMIYKAVESTNITYGFALVLTMLLYLIFIILYMFYFMEATAAGLFLDTKKYIDFLICV
LSELLHAMLIIFLNIYFSEETVKETRTTSFV-
>BmorGR67
MRERKKKFNKLLNTRNYNNIVEALLPSDSIRKISGVSVVYLAVNSENRIVTKFSFIGTIFFLF
WYILYFYCTYKAHSEDQTILRTIYNTKLKRYGDDFERIASIIYVTYSMWKVPFRMSGNQVF
IQRIVDIDSAIENMGEAVDYNKNAKTALVISIAQLGDFLVRMFCIWLSLENLSVIVPTEKLY
QVVYTDALSFVITSHYCFSLIVLRGRYKYINKVLSEIKTRSAWEYKVFVRNKVAPDLEKVQ
RLQDRIVCEKIKACARIYSMLYKATEAINRMYGTALVLTMLLYLVFIILYMFYFMEATASGL
LYDIKKYVDFLICVFWQMSHALSIIYANVYFSESITREVCKF-
>BmorGR68
MRFGLKAGAAVVTILRPYNLCLKNIFKPFYVMLSLLGLFPYSIRFLGGKQFLIKPKSIYTNA
VCALSLMLSMTLFLIFHIDHIIYKSTEDNSLTEGFMTQVNYIIEMLNLEIFCVVYYFSSFLNR
NKFVKVLNTVAVWSDRISISGIKTLSFLRLKIHFSIGILMFLLISQVCVNFTRVDSLWKKVLV
MFTFNIPQMIQFTAILFYYILVNMVITLLVIIQENISISTRDTKTSSFIRVEHRMPLSLKQLELI

YIKAFELKRDINKAFEAPILLTTMQCFHSIVSESHIIYHGAVMEPHMVLHSIMNCSVWILYQ
LFKLYILASTGHLLQEKIQHFSNLIHFHGKGLTVYGLFPLDGTLMFKVVASAAMYLIILVQF
DKRN-
>DpleGR24
MGVLPLTRTSPGHNQFKMLSPSMVYSIFCFLGLVSYVMYLSLHKVQILRTTEGKFEEAVIE
YLFTVYLFPMMVVPIIWYETRKIANILNGWVDFEVCYKKLSSRVLPIKMYKKALTMAVVI
PILSTSSVIITHVTMVDFKVMQILPYVFLEILTYLLGGYWYLLCETLSFCAGILAEDFHQAL
RHVGPAGKVAEYRALWLRLSKLAQDTGLANCYTFTFVNLYLFLIITLSIYGLLSQISEGFGI
KDIGLFVTACCSIFLLFFICDEAHYASLNVRTNFQKKLLMVELSWMNTDAQTEVNMFLRA
TEMNPSQISLGGFFDVNRNLFKSLLATMVTYLVVLLQFQISIPDNTQDTYEDEKLITNDTTA
TDATKITTTLATTILTTLAKKKKKN-
>DpleGRP59
MPLQPIKLPLKKHRKGRPRWQYLPEVPGYVPVYIRNGDTPLEEINPELAEAFHALPSGRSA
GKQVEASEAIETEQGNIPEKPSYNDRRHEKKALEKKKKYENVRPAERR-
>DpleGRP21a
MIPDHLFDEGINNSLLRNDMKHVHLNRIVYNKTQKDYERDQRNLLSSQDGDTCEIHDQF
YRDHKLLLVLFRALAVMPITRSRPGTITFSWKSRATTYAIFFYIVTTIIVLVVGYERLMILRSI
KKFDDYIYSVLFVAFLVPHFWIPFVGWGVAHQVAIYKTNWGKFQVRYYRVTGENLKFPNL
KTSIVIISVGCLLLAVCFLLSLCALLDGFLLRHTTAYYHIITMINMNCALWYINCKGIKIASQ
SLSNCFSRDVSIECTASLISSYRFLWLNLSELLQSLGNAYARTYSTYCLFMFFNITIAVYGAL
SEIVDHGFRFSFKEMGLIVDAAYCSTLLFIFADCSHKSTLKVAAGVQDCLLSIDVLSVDRPT
QKEVAAGVQDCLLSIDVLSVDRPTQKEIDHFIQAIEMNPAVVSLKGYAHVNRELLTSVCIN
S-
>DmelGR5a
MRQLKGRNRCNRAVRHLKVQGKMWLKNLKSGLEQIRESQVRGTRKNFLHDGSFHEAVA
PVLAVAQCFCLMPVCGISAPTYRGLSFNRRSWRFWYSSLYLCSTSVDLAFSIRRVAHSVLD
VRSVEPIVFHVSILIASWQFLNLAQLWPGLMRHWAAVERRLPGYTCCLQRARPARRLKLV
AFVLLVVSLMEHLLSIISVVYYDFCPRRSDPVESYLLGASAQLFEVFPYSNWLAWLGKIQN
VLLTFGWSYMDIFLMMLGMGLSEMLARLNRSLEQQVRQPMPEAYWTWSRTLYRSIVELI
REVDDAVSGIMLISFGSNLYFICLQLLKSINTMPSSAHAVYFYFSLLFLLSRSTAVLLFVSAIN
DQAREPLRLLRLVPLKGYHPEVFRFAAELASDQVALTGLKFFNVTRKLFLAMAGTVATYE
LVLIQFHEDKKTWDCSPFNLD-
>DmelGR8a
MSGHLGRVLQFHLRLYQVLGFHGLPLPGDGNPARTRRRLMAWSLFLLISLSALVLACLFS
GEEFLYRGDMFGCANDALKYVFAELGVLAIYLETLSSQRHLANFWWLHFKLGGQKTGLV
SLRSEFQQFCRYLIFLYAMMAAEVAIHLGLWQFQALTQHMLLFWSTYEPLVWLTYLRNLQ
FVLHLELLREQLTGLEREMGLLAEYSRFASETGRSFPGFESFLRRRLVQKQRIYSHVYDML
KCFQGAFNFSILAVLLTINIRIAVDCYFMYYSIYNNVINNDYYLIVPALLEIPAFIYASQSCMV
VVPRIAHQLHNIVTDSGCCSCPDLSLQIQNFSLQLLHQPIRIDCLGLTILDCSLLTRMACSVG
TYMIYSIQFIPKFSNTYM-
>DmelGR9a
MSLWLEHFLTGYFQLCGLVCGWSGSRLGRLLSSTFLVLILIELVGEIETYFTEENPDNESVP
AYFAKVIMGVNMAYKMIHAWIALSALFECRRFRYLLEELPPVKATSFIYRHLILEIILFACN
AFLVLSEYTIRGIYLENLRYAYSLQAVRARYLQMMVLVDRLDGKLEQLHHRVISGSSDYKT

LRLDYAHLAKVTRSLSHLFGLSLLLLNVLCLGDWIIVCNVYFMVAYLQVLPATLFLFGQV
MFVVCPTLIKIWSICAASHRCVSKSKHLQQQLKDLPGQTPVERSQIEGFALQIMQDPIQIDV
CGIYHLNLQTLAGMFFFILEALVIFLQFVSLVRT-
>DmelGR10a
MTSPDERKSFWERHEFKFYRYGHVYALIYGQVVIDYVPQRALKRGVKVLLIAYGHLFSM
LLIVVLPGYFCYHFRTLTDTLDRRLQLLFYVSFTNTAIKYATVIVTYVANTVHFEAINQRCT
MQRTHLEFEFKNAPQEPKRPFEFFMYFKFCLINLMMMIQVCGIFAQYGEVGKGSVSQVRV
HFAIYAFVLWNYTENMADYCYFINGSVLKYYRQFNLQLGSLRDEMDGLRPGGMLLHHC
CELSDRLEELRRRCREIHDLQRESFRMHQFQLIGLMLSTLINNLTNFYTLFHMLAKQSLEE
VSYPVVVGSVYATGFYIDTYIVALINEHIKLELEAVALTMRRFAEPREMDERLTREIEHLSLE
LLNYQPPMLCGLLHLDRRLVYLIAVTAFSYFITLVQFDLYLRKKS-
>DmelGR10b
MRVGKLCRLALRFWMGLILVLGFSSHYYNPTRRRLVYSRILQTYDWLLMVINLGAFYLY
YRYAMTYFLEGMFRRQGFVNQVSTCNVFQQLLMAVTGTWLHFLFERHVCQTYNELSRIL
KHDLKLKEHSRFYCLAFLAKVYNFFHNFNFALSAIMHWGLRPFNVWDLLANLYFVYNSL
ARDAILVAYVLLLLNLSEALRLNGQQEHDTYSDLMKQLRRRERLLRIGRRVHRMFAWLVA
IALIYLVFFNTATIYLGYTMFIQKHDALGLRGRGLKMLLTVVSFLVILWDVVLLQVICEKLL
AEENKICDCPEDVASSRTTYRQWEMSALRRAITRSSPENNVLGMFRMDMRCAFALISCSL
SYGIIIIQIGYIPG-
>DmelGR22d
MFRPRCGLRQKFVYVILKSILYSSWLLGIFPFKYEPKKRRLRRSMWLILFGVVISSSLLILM
VKQSAEDREHGIMLDVFQRNALLYQISSLMGVVGVVSICTVHLRTLWRSKHLEEIYNGLM
LLEAKYFCSNAVECPAFDGYVIQKGVVIVVGLLAPWMVHFGMPDSKLPVLNVLVVSMVK
LGTLLLALHYHLGVVIIYRFVWLINRELLSLVCSLRGNHKGSSSRVRFLLKLYNKLVNLYS
KLADCYDCQTVLMMAIFLAANIIVCFYMIVYRISLSKMSFFVMLIMFPLAIANNFMDFWL
SMKVCDLLQKTGRQTSMILKLFNDIENMDKDLEISISDFALYCSHRRFKFLHCGLFHVNRE
MGFKMFVASVLYLLYLVQFDYMNL-
>DmelGR21a_isoform_A
MSFWAVSRGLTPPSKVVPMLNPNQRQFLEDEVRYREKLKLMARGDAMEEVYVRKQETV
DDPLELDKHDSFYQTTKSLLVLFQIMGVMPIHRNPPEKNLPRTGYSWGSKQVMWAIFIYS
CQTTIVVLVLRERVKKFVTSPDKRFDEAIYNVIFISLLFTNFLLPVASWRHGPQVAIFKNMW
TNYQYKFFKTTGSPIVFPNLYPLTWSLCVFSWLLSIAINLSQYFLQPDFRLWYTFAYYPIIA
MLNCFCSLWYINCNAFGTASRALSDALQTTIRGEKPAQKLTEYRHLWVDLSHMMQQLGR
AYSNMYGMYCLVIFFTTIIATYGSISEIIDHGATYKEVGLFVIVFYCMGLLYIICNEAHYASR
KVGLDFQTKLLNINLTAVDAATQKEVEMLLVAINKNPPIMNLDGYANINRELITTNISFMAT
YLVVLLQFKITEQRRIGQQQA-
>DmelGR22b
MFGSSREIRPYLARQMLKTTLYGSWLLGIFPFTLDSGKRIRQLRRSRCLTLYGLVLNYFLIF
TLIRLAFEYRKHKLEAFKRNPVLEMINVVIGIINVLSALIVHFMNFWGSRKVGEICNELLIL
EYQDFEGLNGRNCPNFNCFVIQKCLTILGQLLSFFTLNFALPGLEFHICLVLLSCLMEFSLNL
NIMHYHVGVLLIYRYVWLINEQLKDLVSQLKLNPETDFSRIHQFLSLYKRLLELNRKLVIA
YEYQMTLFIIAQLSGNIVVIYFLIVYGLSMRTYSIFLVAFPNSLLINIWDFWLCIAACDLTEK
AGDETAIILKIFSDLEHRDDKLEMSVNEFAWLCSHRKFRFQLCGLFSMNCRMGFKMIITTF
LYLVYLVQFDYMNL-

>DmelGR22f
MKMFQPRRGFSCHLAWFMLQTTLYASWLLGLFPFTFDSRRKQLKRSRWLLLYGFVLHSL
AMCLAMSSHLASKQRRKYNAFERNPLLEKIYMQFQVTTFFTISVLLLMNVWKSNTVRKI
ANELLTLEGQVKDLLTLKNCPNFNCFVIKKHVAAIGQFVISIYFCLCQENSYPKILKILCCLP
SVGLQLIIMHFHTEIILVYRYVWLVNETLEDSHHLSSSRIHALASLYDRLLKLSELVVACND
LQLILMLIIYLIGNTVQIFFLIVLGVSMNKRYIYLVASPQLIINFWDFWLNIVVCDLAGKCGD
QTSKVLKLFTDLEHDDEELERSLNEFAWLCTHRKFRFQLCGLFSINHNMGFQMIITSFLYL
VYLLQFDFMNL-
>DmelGR22e
MFRPSGSGYRQKWTGLTLKGALYGSWILGVFPFAYDSWTRTLRRSKWLIAYGFVLNAAFI
LLVVTNDTESETPLRMEVFHRNALAEQINGIHDIQSLSMVSIMLLRSFWKSGDIERTLNELE
DLQHRYFRNYSLEECISFDRFVLYKGFSVVLELVSMLVLELGMSPNYSAQFFIGLGSLCLM
LLAVLLGASHFHLAVVFVYRYVWIVNRELLKLVNKMAIGETVESERMDLLLYLYHRLLDL
GQRLASIYDYQMVMVMVSFLIANVLGIYFFIIYSISLNKSLDFKILVFVQALVINMLDFWLN
VEICELAERTGRQTSTILKLFNDIENIDEKLERSITDFALFCSHRRLRFHHCGLFYVNYEMG
FRMAITSFLYLLFLIQFDYWNL-
>DmelGR22a
MSQPKRIHRICKGLARFTIRATLYGSWVLGLFPFTFDSRKRRLNRSKWLLAYGLVLNLTLL
VLSMLPSTDDHNSVKVEVFQRNPLVKQVEELVEVISLITTLVTHLRTFSRSSELVEILNELLV
LDKNHFSKLMLSECHTFNRYVIEKGLVIILEIGSSLVLYFGIPNSKIVVYEAVCIYIVQLEVL
MVVMHFHLAVIYIYRYLWIINGQLLDMASRLRRGDSVDPDRIQLLLWLYSRLLDLNHRLT
AIYDIQVTLFMATLFSVNIIVGHVLVICWINITRFSLLVIFLLFPQALIINFWDLWQGIAFCDL
AESTGKKTSMILKLFNDMENMDQETERRVTEFTLFCSHRRLKVCHLGLLDINYEMGFRMI
ITNILYVVFLVQFDYMNLKFKTD-
>DmelGR28a
MAFKLWERFSQADNVFQALRPLTFISLLGLAPFRLNLNPRKEVQTSKFSFFAGIVHFLFFVL
CFGISVKEGDSIIGYFFQTNITRFSDGTLRLTGILAMSTIFGFAMFKRQRLVSIIQNNIVVDEIF
VRLGMKLDYRRILLSSFLISLGMLLFNVIYLCVSYSLLVSATISPSFVTFTTFALPHINISLMV
FKFLCTTDLARSRFSMLNEILQDILDAHIEQLSALELSPMHSVVNHRRYSHRLRNLISTPMK
RYSVTSVIRLNPEYAIKQVSNIHNLLCDICQTIEEYFTYPLLGIIAISFLFILFDDFYILEAILNP
KRLDVFEADEFFAFFLMQLIWYIVIIVLIVEGSSRTILHSSYTAAIVHKILNITDDPELRDRLF
RLSLQLSHRKVLFTAAGLFRLDRTLIFTITGAATCYLIILIQFRFTHHMDDTSSNSTNNLHSI
HLGD-
>DmelGR28b_isoform_A
MIRCGLDIFRGCRGRFRYWLSARDCYDSISLMVAIAFALGITPFLVRRNALGENSLEQSWY
GFLNAIFRWLLLAYCYSYINLRNESLIGYFMRNHVSQISTRVHDVGGIIAAVFTFILPLLLRK
YFLKSVKNMVQVDTQLERLRSPVNFNTVVGQVVLVILAVVLLDTVLLTTGLVCLAKMEV
YASWQLTFIFVYELLAISITICMFCLMTRTVQRRITCLHKVLKNLAHQWDTRSLKAVNQKQ
RSLQCLDSFSMYTIVTKDPAEIIQESMEIHHLICEAAATANKYFTYQLLTIISIAFLIIVFDAYY
VLETLLGKSKRESKFKTVEFVTFFSCQMILYLIAIISIVEGSNRAIKKSEKTGGIVHSLLNKT
KSAEVKEKLQQFSMQLMHLKINFTAAGLFNIDRTLYFTISGALTTYLIILLQFTSNSPNNGY
GNGSSCCETFNNMTNHTL-
>DmelGR32a
MSPNTWVIEMPTQKTRSHPYPRRISPYRPPVLNRDAFSRDAPPMPARNHDHPVFEDIRTILS

VLKASGLMPIYEQVSDYEVGPPTKTNEFYSFFVRGVVHALTIFNVYSLFTPISAQLFFSYRE
TDNVNQWIELLLCILTYTLTVFVCAHNTTSMLRIMNEILQLDEEVRRQFGANLSQNFGFLV
KFLVGITACQAYIIVLKIYAVQGEITPTSYILLAFYGIQNGLTATYIVFASALLRIVYIRFHFIN
QLLNGYTYGQQHRRKEGGARARRQRGDVNPNVNPALMEHFPEDSLFIYRMHNKLLRIYK
GINDCCNLILVSFLGYSFYTVTTNCYNLFVQITGKGMVSPNILQWCFAWLCLHVSLLALLS
RSCGLTTTEANATSQILARVYAKSKEYQNIIDKFLTKSIKQEVQFTAYGFFAIDNSTLFKIFSA
VTTYLVILIQFKQLEDSKVEDPVPEQT-
>DmelGR33a
MIQIMNWFSMVIGLIPLNRQQSETNFILDYAMMCIVPIFYVACYLLINLSHIIGLCLLDSCNS
VCKLSSHLFMHLGAFLYLTITLLSLYRRKEFFQQFDARLNDIDAVIQKCQRVAEMDKVKVT
AVKHSVAYHFTWLFLFCVFTFALYYDVRSLYLTFGNLAFIPFMVSSFPYLAGSIIQGEFIYHV
SVISQRFEQINMLLEKINQEARHRHAPLTVFDIESEGKKERKTVTPITVMDGRTTTGFGNEN
KFAGEMKRQEGQQKNDDDDLDTSNDEDEDDFDYDNATIAENTGNTSEANLPDLFKLHD
KILALSVITNGEFGPQCVPYMAACFVVSIFGIFLETKVNFIVGGKSRLLDYMTYLYVIWSFT
TMMVAYIVLRLCCNANNHSKQSAMIVHEIMQKKPAFMLSNDLFYNKMKSFTLQFLHWE
GFFQFNGVGLFALDYTFIFSTVSAATSYLIVLLQFDMTAILRNEGLMS-
>DmelGR36a
MFDWVGLLLKVLYYYGQIIGLINFEIDWQRGRVVAAQRGILFAIAINVLICMVLLLQISKKF
NLDVYFGRANQLHQYVIIVMVSLRMASGISAILNRWRQRAQLMRLVECVLRLFLKKPHV
KQMSRWAILVKFSVGVVSNFLQMAISMESLDRLGFNEFVGMASDFWMSAIINMAISQHYL
VILFVRAYYHLLKTEVRQAIHESQMLSEIYPRRAAFMTKCCYLADRIDNIAKLQNQLQSIV
TQLNQVFGIQGIMVYGGYYIFSVATTYITYSLAINGIEELHLSVRAAALVFSWFLFYYTSAI
LNLFVMLKLFDDHKEMERILEERTLFTSALDVRLEQSFESIQLQLIRNPLKIEVLDIFTITRSS
SAAMIGSIITNSIFLIQYDMEYF-
>DmelGR36b
MVDWVVLLLKAVHIYCYLIGLSNFEFDCRTGRVFKSRRCTIYAFMANIFILITIIYNFTAHGD
TNLLFQSANKLHEYVIIIMSGLKIVAGLITVLNRWLQRGQMMQLVKDVIRLYMINPQLKS
MIRWGILLKAFISFAIELLQVTLSVDALDRQGTAEMMGLLVKLCVSFIMNLAISQHFLVILLI
RAQYRIMNAKLRMVIEESRRLSFLQLRNGAFMTRCCYLSDQLEDIGEVQSQLQSMVGQL
DEVFGMQGLMAYSEYYLSIVGTSYMSYSIYKYGPHNLKLSAKTSIIVCILITLFYLDALVNC
NNMLRVLDHHKDFLGLLEERTVFASSLDIRLEESFESLQLQLARNPLKINVMGMFPITRGS
TAAMCASVIVNSIFLIQFDMEFF-
>DmelGR36c
MDLESFLLGAVYYYGLFIGLSNFEFDWNTGRVFTKKWSTLYAIALDSCIFALYIYHWTGNT
NIVNAIFGRANMLHEYVVAILTGLRIVTGLFTLILRWYQRCKMMDLASKVVRMYVARPQ
VRRMSRWGILTKFIFGSITDGLQMAMVLSAMGSVDSQFYLGLGLQYWMFVILNMAMMQ
QHMIMLFVRTQFQLINTELRQVIDEAKDLLLSPRHQGVFMTKCCSLADQIENIARIQSQLQ
TIMNQMEEVFGIQGAMTYGGYYLSSVGTCYLAYSILKHGYENLSMTLSTVILAYSWCFFY
YLDGMLNLSVMLHVQDDYWEMLQILGKRTIFVGLDVRLEEAFENLNLQLIRNPLKITVV
KLYDVTRSNTMAMFGNLITHSIFLIQYDIEHF-
>DmelGR39a_isoform_A
MSKVCRDLRIYLRLLHIMGMMCWHFDSDHCQLVATSGSERYAVVYAGCILVSTTAGFIFAL
LHPSRFHIAIYNQTGNFYEAVIFRSTCVVLFLVYVILYAWRHRYRDLVQHILRLNRRCASSC
TNQQFLHNIILYGMLTILCFGNYLHGYTRAGLATLPLALCMLVYIFAFLVLCLLLMFFVSLK

QVMTAGLIHYNQQLCQGDLISGLRGRQQILKLCGGELNECFGLLMLPIVALVLLMAPSGPF
FLISTVLEGKFRPDECLIMLLTSSTWDTPWMIMLVLMLRTNGISEEANKTAKMLTKVPRTG
TGLDRMIEKFLLKNLRQKPILTAYGFFALDKSTLFKLFTAIFTYMVILVQFKEMENSTKSIN
KF-
>DmelGR39b
MLYSFHPYLKYFALLGLVPWSESCAQSKFVQKVYSAILIILNAVHFGISIYFPQSAELFLSLM
VNVIVFVARIVCVTVIILQVMVHYDDYFRFCREMKYLGLRLQCELKIHVGRLKWQSYAKI
LALGIGFLVTVLPSIYVALSGSLLYFWSSLLSILIIRMQFVLVLLNVELLGHHVSLLGIRLQN
VLECHLMGANCTLDGNANRLCSLEFLLALKQSHMQLHYLFTHFNDLFGWSILGTYVVLF
SDSTVNIYWTQQVLVEVYEYKYLYATFSVFVPSFFNILVFCRCGEFCQRQSVLIGSYLRNLS
CHPSIGRETSYKDLLMEFILQVEQNVLAINAEGFMSTDNSLLMSILAAKVTYLIVLMQFSS
V-
>DmelGR43a_isoform_A
MEISQPSIGIFYISKVLALAPYATVRNSKGRVEIGRSWLFTVYSATLTVVMVFLTYRGLLFD
ANSEIPVRMKSATSKVVTALDVSVVVMAIVSGVYCGLFSLNDTLELNDRLNKIDNTLNAY
NNFRRDRWRALGMAAVSLLAISILVGLDVGTWMRIAQDMNIAQSDTELNVHWYIPFYSL
YFILTGLQVNIANTAYGLGRRFGRLNRMLSSSFLAENNATSAIKPQKVSTVKNVSVNRPAM
PSALHASLTKLNGETLPSEAAAKNKGLLLKSLADSHESLGKCVHLLSNSFGIAVLFILVSCL
LHLVATAYFLFLELLSKRDNGYLWVQMLWICFHFLRLLMVVEPCHLAARESRKTIQIVCEI
ERKVHEPILAEAVKKFWQQLLVVDADFSACGLCRVNRTILTSFASAIATYLVILIQFQRTNG-
>DmelGR47a
MAFTSSQLCSLLTKFTALNGLNTYYFDTKTNAFRVSSKLKIYCAIHHALCVLALAHMSYST
ASNLRVSVTVLTIGGTMACCVKSCWEKAQGIRNLARGLVTMEQKYFAGRPSGLLLKCRY
YIKITFGSITLLRIHLIQPIYMRRLLPSQFYLNVGAYWLLYNMLLAAVLGFYFLLWEMCRIQ
KLINDQMTLILARSGQRNRLKKMQHCLRLYSKLLLLCDQFNSQLGHVAIWVLACKSWCQ
ITFGYEIFQMVAAPKSIDLTMSMRVFVIFTYIFDAMNLFLGTDISELFSTFRADSQRILRETSR
LDRLLSMFALKLALHPKRVVLLNVFTFDRKLTLTLLAKSTLYTICCLQNDYNKLKA-
>DmelGR47b
MQRDDGFVYCYGNLYSLLLYWGLVTIRVRSPDRGGAFSNRWTVCYALFTRSFMVICFMA
TVMTKLRDPEMSAAMFGHLSPLVKAIFTWECLSCSVTYIEYCLSLDLQKDRHLKLVARMQ
EFDRSVLMVFPHVQWNYRRARLKYWYGTVIVGFCFFSFSISLIFDTTRCTCGIPSTLLMAF
TYTLLTSSVGLLGFVHIGIMDFIRVRLRLVQQLLHQLYQADDSSEVHERIAYLFEMSKRCSF
LLAELNGVFGFAAAAGIFYDFTIMTCFVYVICQKLLEREPWDPEYVYMLLHVAIHTYKVV
ITSTYGYLLLREKRNCMHLLSQYSRYFSGQDVARRKTEDFQHWRMHNRQAAMVGSTTL
LSVSTIYLVYNGMANYVIILVQLLFQQQQIKDHQLTSGKDVDIVGPMGPITHMD-
>DmelGR57a
MAVLYFFREPETVFDCAAFICILQFLMGCNGFGIRRSTFRISWASRIYSMSVAIAAFCCLFGS
LSVLLAEEDIRERLAKADNLVLSISALELLMSTLVFGVTVISLQVFARRHLGIYQRLAALDA
RLMSDFGANLNYRKMLRKNIAVLGIVTTIYLMAINSAAVQVASGHRALFLLFALCYTIVTG
GPHFTGYVHMTLAEMLGIRFRLLQQLLQPEFLNWRFPQLHVQELRIRQVVSMIQELHYLI
QEINRVYALSLWAAMAHDLAMSTSELYILFGQSVGIGQQNEEENGSCYRMLGYLALVMIP
PLYKLLIAPFYCDRTIYEARRCLRLVEKLDDWFPQKSSLRPLVESLMSWRIQAKIQFTSGLD
VVLSRKVIGLFTSILVNYLLILIQFAMTQKMGEQIEQQKIALQEWIGF-
>DmelGR58a

MLLKFMYIYGIGCGLMPAPLKKGQFLLGYKQRWYLIYTACLHGGLLTVLPFTFPHYMYD
DSYMSSNPVLKWTFNLTNITRIMAMFSGVLLMWFRRKRILNLGENLILHCLKCKTLDNRS
KKYSKLRKRVRNVLFQMLLVANLSILLGALILFRIHSVQRISKTAMIVAHITQFIYVVFMMT
GICVILLVLHWQSERLQIALKDLCSFLNHEERNSLTLSENKANRSLGKLAKLFKLFAENQR
LVREVFRTFDLPIALLLLKMFVTNVNLVYHGVQFGNDTIETSSYTRIVGQWVVISHYWSAV
LLMNVVDDVTRRSDLKMGDLLREFSHLELVKRDFHLQLELFSDHLRCHPSTYKVCGLFIF
NKQTSLAYFFYVLVQVLVLVQFDLKNKVEKRN-
>DmelGR58b
MLHPKLGRVMNVVYYHSVVFALMSTTLRIRSCRKCLRLEKVSRTYTIYSFFVGIFLFLNLY
FMVPRIMEDGYMKYNIVLQWNFFVMLFLRAIAVVSCYGTLWLKRHKIIQLYKYSLIYWK
RFGHITRAIVDKKELLDLQESLARIMIRKIILLYSAFLCSTVLQYQLLSVINPQIFLAFCARLT
HFLHFLCVKMGFFGVLVLLNHQFLVIHLAINALHGRKARKKWKALRSVAAMHLKTLRLA
RRIFDMFDIANATVFINMFMTAINILYHAVQYSNSSIKSNGWGILFGNGLIVFNFWGTMAL
MEMLDSVVTSCNNTGQQLRQLSDLPKVGPKMQRELDVFTMQLRQNRLVYKICGIVELDK
PACLSYIGSILSNVIILMQFDLRRQRQPINDRQYLIHLMKNKTKV-
>DmelGR58c
MNQYFLLHTYFQVSRLIGLCNLHYDSSNHRFILNHVPTVVYCVILNVVYLLVLPFALFVLT
GNIYHCPDAGMFGVVYNVVALTKLLTMLFLMSSVWIQRRRLYKLGNDLMKMLHKFRFN
LGNDCRNRCLCKGLLTSSRFVLLTQQLLTRDSVVNCESNSSLRQAMVPYQSAAIVYALIMI
LLMSYVDMTVYMVEVAGNWLLVNMTQGVREMVQDLEVLPERNGIPREMGLMQILAAW
RKLWRRCRRLDALLKQFVDIFQWQVLFNLLTTYIFSIAVLFRLWIYLEFDKNFHLWKGILY
AIIFLTHHVEIVMQFSIFEINRCKWLGLLEDVGNLWDINYSGRQCIKSSGTILSRKLEFSLLY
MNRKLQLNPKRVRRLHIVGLFDLSNLTVHNMTRSIITNVLVLCQIAYKKYG-
>DmelGR59a
MKRIGQAYNVYAVFIGMTSYETMGGKFRQSRITRIYCLLINAIFLTLLPSAFWKSAKLLSTA
DWMPSYMRVTPYIMCTINYAAIAYTLISRCYRDAMLMDLQRIVLEVNREMLRTGKKMNS
LLRRMFFLKTFTLTYSCLSYILAVFIYQWKAQNWSNLCNGLLVNISLTILFVNTFFYFTSLW
HIARGYDFVNQQLNEIVACQSMDLERKSKELRGLWALHRNLSYTARRINKHYGPQMLAM
RFDYFIFSIINACIGTIYSTTDQEPSLEKIFGSLIYWVRSFDFFLNDYICDLVSEYQMQPKFFA
PESSMSNELSSYLIYESSTRLDLLVCGLYRVNKRKWLQMVGSIVVHSSMLFQFHLVMRGG
L-
>DmelGR59b
MVYWMIKLYFRYSLAIGITSQQFSNRKFFSTLFSRTYALIANIVTLIMLPIVMWQVQLVFQQ
KKTFPKLILITNNVREAVSFLVILYTVLSRGFRDTAFKEMQPLLLTLFREEKRCGFKGIGGV
RRSLRILLFVKFFTLSWLCVTDVLFLLYSTDALIWVNVLRFFFKCNTNNILEMVPMGYFLA
LWHIARGFDCVNRRLDQIVKSKSTRKHRELQHLWLLHACLTKTALNINKIYAPQMLASRF
DNFVNGVIQAYWGAVFTFDLSTPFFWVVYGSVQYHVRCLDYYLIDNMCDVAVEYHDSA
KHSWSEVRWTKEISSYVIYANSTKLQLWSCGLFQANRSMWFAMISSVLYYILVLLQFHLV
MRK-
>DmelGR59c
MVDLVKTILLIAYWYGLAVGVSNFEVDWLTGEAIATRRTTIYAAVHNASLITLLILFNLGNN
SLKSEFISARYLHEYFFMLMTAVRISAVLLSLITRWYQRSRFIRIWNQILALVRDRPQVVRG
RWYRRSIILKFVFCVLSDSLHTISDVSAQRKRITADLIVKLSLLATLTTIFNMIVCQYYLAMV
QVIGLYKILLQDLRCLVRQAECICSIRNRRGGVYSIQCCSLADQLDLIAERHYFLKDRLDE

MSDLFQIQSLSMSLVYFFSTMGSIYFSVCSILYSSTGFGSTYWGLLLIVLSTASFYMDNWLS
VNIGFHIRDQQDELFRVLADRTLFYRELDNRLEAAFENFQLQLASNRHEFYVMGLFKMER
GRLIAMLSSVITHTMVLVQWEIQNDES-
>DmelGR59d
MADLLKLCLRIAYAYGRLTGVINFKIDLKTGQALVTRGATLISVSTHLLIFALLLYQTMRKS
VVNVMWKYANSLHEYVFLVIAGFRVVCVFLELVSRWSQRRTFVRLFNSFRRLYQRNPDII
QYCRRSIVSKFFCVTMTETLHIIVTLAMMRNRLSIALALRIWAVLSLTAIINVIITQYYVATA
CVRGRYALLNKDLQAIVTESQSLVPNGGGVFVTKCCYLADRLERIAKSQSDLQELVENLS
TAYEGEVVCLVITYYLNMLGTSYLLFSISKYGNFGNNLLVIITLCGIVYFVFYVVDCWINAF
NVFYLLDAHDKMVKLLNKRTLFQPGLDHRLEMVFENFALNLVRNPLKLHMYGLFEFGR
GTSFAVFNSLLTHSLLLIQYDVQNF-
>DmelGR59e
MDSSYWENLLLTINRFLGVYPSGRVGVLRWLHTLWSLFLLMYIWTGSIVKCLEFTVEIPTI
EKLLYLMEFPGNMATIAILVYYAVLNRPLAHGAELQIERIITGLKGKAKRLVYKRHGQRTL
HLMATTLVFHGLCVLVDVVNYDFEFWTTWSSNSVYNLPGLMMSLGVLQYAQPVHFLWL
VMDQMRMCLKELKLLQRPPQGSTKLDACYESAFAVLVDAGGGSALMIEEMRYTCNLIEQ
VHSQFLLRFGLYLVLNLLNSLVSICVELYLIFNFFETPLWEESVLLVYRLLWLAMHGGRIWF
ILSVNEQILEQKCNLCQLLNELEVCSSRLQRTINRFLLQLQRSIDQPLEACGIVTLDTRSLGG
FIGVLMAIVIFLIQIGLGNKSLMGVALNRSNWVYV-
>DmelGR59f
MRSSATKGAKLKNSPRERLSSFNPQYAERYKELYRTLFWLLLISVLANTAPITILPGCPNRF
YRLVHLSWMILWYGLFVLGSYWEFVLVTTQRVSLDRYLNAIESAIYVVHIFSIMLLTWQC
RNWAPKLMTNIVTSDLNRAYTIDCNRTKRFIRLQLFLVGIFACLAIFFNIWTHKFVVYRSILS
INSYVMPNIISSISFAQYYLLLQGIAWRQRRLTEGLERELTHLHSPRISEVQKIRMHHANLID
FTKAVNRTFQYSILLLFVGCFLNFNLVLFLVYQGIENPSMADFTKWVCMLLWLAMHVGK
VCSILHFNQSIQNEHSTCLTLLSRVSYARKDIQDTITHFIIQMRTNVRQHVVCGVINLDLKFL
TTLLVASADFFIFLLQYDVTYEALSKSVQGNVTRYK-
>DmelGR61a_isoform_A
MSRTSDDIRKHLKVRRQKQRAILAMRWRCAQGGLEFEQLDTFYGAIRPYLCVAQFFGIMP
LSNIRSRDPQDVKFKVRSIGLAVTGLFLLLGGMKTLVGANILFTEGLNAKNIVGLVFLIVG
MVNWLNFVGFARSWSHIMLPWSSVDILMLFPPYKRGKRSLRSKVNVLALSVVVLAVGDH
MLYYASGYCSYSMHILQCHTNHSRITFGLYLEKEFSDIMFIMPFNIFSMCYGFWLNGAFTF
LWNFMDIFIVMTSIGLAQRFQQFAARVGALEGRHVPEALWYDIRRDHIRLCELASLVEAS
MSNIVFVSCANNVYVICNQALAIFTKLRHPINYVYFWYSLIFLLARTSLVFMTASKIHDASL
LPLRSLYLVPSDGWTQEVQRFADQLTSEFVGLSGYRLFCLTRKSLFGMLATLVTYELMLLQ
IDAKSHKGLRCA-
>DmelGR63a
MANYYRRKKGDAVFLNAKPLNSANAQAYLYGVRKYSIGLAERLDADYEAPPLDRKKSS
DSTASNNPEFKPSVFYRNIDPINWFLRIIGVLPIVRHGPARAKFEMNSASFIYSVVFFVLLAC
YVGYVANNRIHIVRSLSGPFEEAVIAYLFLVNILPIMIIPILWYEARKIAKLFNDWDDFEVLY
YQISGHSLPLKLRQKAVYIAIVLPILSVLSVVITHVTMSDLNINQVVPYCILDNLTAMLGAW
WFLICEAMSITAHLLAERFQKALKHIGPAAMVADYRVLWLRLSKLTRDTGNALCYTFVFM
SLYLFFIITLSIYGLMSQLSEGFGIKDIGLTITALWNIGLLFYICDEAHYASVNVRTNFQKKLL
MVELNWMNSDAQTEINMFLRATEMNPSTINCGGFFDVNRTLFKGLLTTMVTYLVVLLQF

QISIPTDKGDSEGANNITVVDFVMDSLDNDMSLMGASTLSTTTVGTTLPPPIMKLKGRKG-
>DmelGR64a
MKGPNLNFRKTPSKDNGVKQVESLARPETPPPKFVEDSNLEFNVLASEKLPNYTNLDLFH
RAVFPFMFLAQCVAIMPLVGIRESNPRRVRFAYKSIPMFVTLIFMIATSILFLSMFTHLLKIGI
TAKNFVGLVFFGCVLSAYVVFIRLAKKWPAVVRIWTRTEIPFTKPPYEIPKRNLSRRVQLAA
LAIIGLSLGEHALYQVSAILSYTRRIQMCANITTVPSFNNYMQTNYDYVFQLLPYSPIIAVLI
LLINGACTFVWNYMDLFIMMISKGLSYRFEQITTRIRKLEHEEVCESVFIQIREHYVKMCE
LLEFVDSAMSSLILLSCVNNLYFVCYQLLNVFNKLRWPINYIYFWYSLLYLIGRTAFVFLTA
ADINEESKRGLGVLRRVSSRSWCVEVERLIFQMTTQTVALSGKKFYFLTRRLLFGMAGTIV
TYELVLLQFDEPNRRKGLQPLCA-
>DmelGR64b
MPQGETFHRAVSNVLFISQIYGLLPVSNVRALDVADIRFRWCSPRILYSLLIGILNLSEFGAVI
NYVIKVTINFHTSSTLSLYIVCLLEHLFFWRLAIQWPRIMRTWHGVEQLFLRVPYRFYGEY
RIKRRIYIVFTIVMSSALVEHCLLLGNSFHLSNMERTQCKINVTYFESIYKWERPHLYMILP
YHFWMLPILEWVNQTIAYPRSFTDCFIMCIGIGLAARFHQLYRRIAAVHRKVMPAVFWTEV
REHYLALKRLVHLLDAAIAPLVLLAFGNNMSFICFQLFNSFKNIGVDFLVMLAFWYSLGFA
VVRTLLTIFVASSINDYERKIVTALRDVPSRAWSIEVQRFSEQLGNDTTALSGSGFFYLTRSL
VLAMGTTIITYELMISDVINQGSIRQKTQYCREY-
>DmelGR64c_isoform_A
MQQSGQKGTRNTLQHAIGPVLVIAQFFGVLPVAGVWPSCRPERVRFRWISLSLLAALILFV
FSIVDCALSSKVVFDHGLKIYTIGSLSFSVICIFCFGVFLLLSRRWPYIIRRTAECEQIFLEPEY
DCSYGRGYSSRLRLWGVCMLVAALCEHSTYVGSALYNNHLAIVECKLDANFWQNYFQR
ERQQLFLIMHFTAWWIPFIEWTTLSMTFVWNFVDIFLILICRGMQMRFQQMHWRIRQHVR
QQMPNEFWQRIRCDLLDLSDLLGIYDKELSGLIVLSCAHNMYFVCVQIYHSFQSKGNYAD
ELYFWFCLSYVIIRVLNMMFAASSIPQEAKEISYTLYEIPTEFWCVELRRLNEIFLSDHFALS
GKGYFLLTRRLIFAMAATLMVYELVLINQMAGSEVQKSFCEGGVGSSKSIFS-
>DmelGR64d_isoform_A
MLSTKIVLNDGLQLYTMGSLSFSVICIFCFGSFIKLSRRWPHIIRETALCERIFLKPCYANQEG
LNFTRFLRRWALILLVAALCEHLTYVGSAAWSNYVQIRDCNLKVGFVENYFLRERQELFS
VFEYRAWMVFFIEWNTMAMTFVWNFGDIFLFLMCRGLKIRFQQLHWRIRQNLGKPMAK
EFWQEIRSDFLDLDSLLKLYDKELSGLILVCCAHNMYFICVQVYHSFQVKGAFMDELYFW
FCLLYVISRLMNMMLAASSIPQEIKDISNTLYEVRSSPWCDELGRLSEMLRNETFALSGMG
YFYVTRRLIFAMAGALMGYELVLFRQMQGAVVQKSICSRGPGSSMSIFFS-
>DmelGR64e_isoform_A
MARTTGDPAKRRRCMSRIKFWRRSRVGSEVVEKDTKRFKLSLIKAWLLRIRQEDYKYSGS
FQEAIKPVLIIAQIFALMPVRKVSSKFAEDLTFTWFSVRSYYALVTILFFGVSSGYMVAFVTS
VSFNFDSVETLVFYLSIFLISLSFFQLARKWPEIAQSWQLVEAKLPPLKLPKERRSLAQHIN
MITIVATTCSLVEHIMSMLSMGYYVNSCPRWPDRPIDSFLYLSFSSVFYFVDYTRFLGIVGK
VVNVLSTFAWNFNDIFVMAVSVALAARFRQLNDYMMREARLPTTVDYWMQCRINFRNL
CKLCEEVDDAISTITLLCFSNNLYFICGKILKSMQAKPSIWHALYFWFSLVYLLGRTLILSLY
SSSINDESKRPLVIFRLVPREYWCDELKRFSEEVQMDNVALTGMKFFRLTRGVVISVAGTIV
TYELILLQFNGEEKVPGCFEN-
>DmelGR64f
MKILPKLERKLRRLKKRVTRTSLFRKLDLVHESARKKAFQESCETYKNQIENEYEIRNSLP

KLSRSDKEAFLSDGSFHQAVGRVLLVAEFFAMMPVKGVTGKHPSDLSFSWRNIRTCFSLLF
IASSLANFGLSLFKVLNNPISFNSIKPIIFRGSVLLVLIVALNLARQWPQLMMYWHTVEKDL
PQYKTQLTKWKMGHTISMVMLLGMMLSFAEHILSMVSAINYASFCNRTADPIQNYFLRTN
DEIFFVTSYSTTLALWGKFQNVFSTFIWNYMDLFVMIVSIGLASKFRQLNDDLRNFKGMN
MAPSYWSERRIQYRNICILCDKMDDAISLITMVSFSNNLYFICVQLLRSLNTMPSVAHAVY
FYFSLIFLIGRTLAVSLYSSSVHDESRLTLRYLRCVPKESWCPEVKRFTEEVISDEVALTGMK
FFHLTRKLVLSVAGTIVTYELVLIQFHEDNDLWDCDQSYYS-
>DmelGR66a
MAQAEDAVQPLLQQFQQLFFISKIAGILPQDLEKFRSRNLLEKSRNGMIYMLSTLILYVVLY
NILIYSFGEEDRSLKASQSTLTFVIGLFLTYIGLIMMVSDQLTALRNQGRIGELYERIRLVDER
LYKEGCVMDNSTIGRRIRIMLIMTVIFELSILVSTYVKLVDYSQWMSLLWIVSAIPTFINTLD
KIWFAVSLYALKERFEAINATLEELVDTHEKHKLWLRGNQEVPPPLDSSQPPQYDSNLEYL
YKELGGMDIGSIGKSSVSGSGKNKVAPVAHSMNSFGEAIDAASRKPPPPPLATNMVHESEL
GNAAKVEEKLNNLCQVHDEICEIGKALNELWSYPILSLMAYGFLIFTAQLYFLYCATQYQSI
PSLFRSAKNPFITVIVLSYTSGKCVYLIYLSWKTSQASKRTGISLHKCGVVADDNLLYEIVN
HLSLKLLNHSVDFSACGFFTLDMETLYGVSGGITSYLIILIQFNLAAQQAKEAIQTFNSLND
TAGLVGAATDMDNISSTLRDFVTTTMTPAV-
>DmelGR68a
MKIYQDIYPISKPSQIFAILPFYSGDVDDGFRFGGLGRWYGRLVALIILIGSLTLGEDVLFASK
EYRLVASAQGDTEEINRTIETLLCIISYTMVVLSSVQNASRHFRTLHDIAKIDEYLLANGFRE
TYSCRNLTILVTSAAGGVLAVAFYYIHYRSGIGAKRQIILLLIYFLQLLYSTLLALYLRTLMM
NLAQRIGFLNQKLDTFNLQDCGHMENWRELSNLIEVLCKFRYITENINCVAGVSLLFYFGF
SFYTVTNQSYLAFATLTAGSLSSKTEVADTIGLSCIWVLAETITMIVICSACDGLASEVNGTA
QILARIYGKSKQFQNLIDKFLTKSIKQDLQFTAYGFFSIDNSTLFKIFSAVTTYLVILIQFKQL
EDSKVEDISQA-
>DmelGR77a
MPLPLGDPLALAVSPQLGYIRITAMPRWLQLPGMSALGILYSLTRVFGLMATANWSPRGIK
RVRQSLYLRIHGCVMLIFVGCFSPFAFWCIFQRMAFLRQNRILLMIGFNRYVLLLVCAFMT
LWIHCFKQAEIIGCLNRLLKCRRRLRRLMHTRKLKDSMDCLATKGHLLEVVVLLSSYLLS
MAQPIQILKDDPEVRRNFMYACSLVFVSVCQAILQLSLGMYTMAILFLGHLVRHSNLLLA
KILADAEHIFESSQKAGFWPNRQELYKGQQKWLALELWRLLHVHHQLLKLHRSICSLCAV
QAVCFLGFVPLECTIHLFFTYFMKYSKFILRKYGRSFPLNYFAIAFLVGLFTNLLLVILPTYY
SERRFNCTREIIKGGGLAFPSRITVKQLRHTMHFYGLYLKNVEHVFAVSACGLFKLNNAIL
FCIVGAILEYLMILIQFDKVLNK-
>DmelGR85a
MYSLIEAQLLGGKLVNRVMASLRRIIQRSLGYFCALNGILDFNTDIGTGNLRRYRVLFMYR
LLHNFAVISLTLKFLFDFTDHFKYIESSTLITVNFFTYFTLVFFALLSSMGSCYQWQNRILAV
LKELKHQRDLSRHMGYRVPRSKQNSIDYLLFALTVLLILRLSIHLATFTLSARMGFNHPCN
CFLPECMIFSMNYLLFAILAEITRCWWSLQSGLKMVLLNRQLSTVAFNLWEIERLHTRFQC
LIDLTSEVCSIFRYVTLAYMARNLWSGIVAGYLLVRFVIGNGLQDVELVYLVFSFITCIQPL
MLSLLVNSMTSTTGSLVEVTRDILKISHKKSVNLERSIEWLSLQLTWQHTHVTIFGVFRINR
SLAFRSASLILVHVLYMVQSDYISITN-
>DmelGR89a
MLRFPHVCGLCLLLKYWQILALAPFRTSEPMVARCQRWMTLIAVFRWLLLTSMAPFVLW

KSAAMYEATNVRHSMVFKTIALATMTGDVCISLALLGNHLWNRRELANLVNDLARLHRR
RRLSWWSTLFLWLKLLLSLYDLLCSVPFLKGAGGRLPWSQLVAYGVQLYFQHVASVYGN
GIFGGILLMLECYNQLEREEPTNLARLLQKEYSWLRLIQRFVKLFQLGIFLLVLGSFVNIMV
NIYAFMSYYVSLHGVPLTISNNCLVLAIQLYAVILAAHLCQVRSAKLRKKCLQLEYVPEGLT
QEQAMASTPFPVLTPTGNVKFRILGVFILDNSFWLFLVSYAMNFIVVILQTSFEHINHGEI-
>DmelGR92a
MFEFLHQMSAPKLSTSILRYIFRYAQFIGVIFFCLHTRKDDKTVFIRNWLKWLNVTHRIITF
TRFFWVYIASISIKTNRVLQVLHGMRLVLSIPNVAVILCYHIFRGPEIIDLINQFLRLFRQVSD
LFKTKTPGFGGRRELILILLNLISFAHEQTYLWFTIRKGFSWRFLIDWWCDFYLVSATNIFIH
INSIGYLSLGVLYSELNKYVYTNLRIQLQKLNTSGSKQKIRRVQNRLEKCISLYREIYHTSIM
FHKLFVPLLFLALIYKVLLIALIGFNVAVEFYLNSFIFWILLGKHVLDLFLVTVSVEGAVNQF
LNIGMQFGNVGDLSKFQTTLDTLFLHLRLGHFRVSILGLFDVTQMQYLQFLSALLSGLAFI
AQYRMQVGNG-
>DmelGR93a
MFSSSSAMTGKRAESWSRLLLLWLYRCARGLLVLSSSLDRDKLQLKATKQGSRNRFLHIL
WRCIVVMIYAGLWPMLTSAVIGKRLESYADVLALAQSMSVSILAVISFVIQARGENQFREV
LNRYLALYQRICLTTRLRHLFPTKFVVFFLLKLFFTLCGCFHEIIPLFENSHFDDISQMVGTG
FGIYMWLGTLCVLDACFLGFLVSGILYEHMANNIIAMLKRMEPIESQDERYRMTKYRRMQ
LLCDFADELDECAAIYSELYHVTNSFRRILQWQILFYIYLNFINICLMLYQYILHFLNDDEV
VFVSIVMAFVKLANLVLLMMCADYTVRQSEVPKKLPLDIVCSDMDERWDKSVETFLGQL
QTQRLEIKVLGFFHLNNEFILLILSAIISYLFILIQFGITGGFEASEDIKNRFD-
>DmelGR93b
MSGLLVMPRILRCLNVSRISAILLRSCFLYGTFFGVITFRIERKDSQLVAINRRGYLWICLVIR
LLASCFYGYSYDAWSGQYEDMYLRAFFGFRLIGCLICSVIILVMQFWFGEELINLVNRFLQ
LFRRMQSLTNSPKNRFGDRAEFLLMFSKVFSLLFVFMAFRLMLSPWFLLTLVCDLYTSVGT
GMITHLCFVGYLSIGVLYRDLNNYVDCQLRAQLRSLNGENNSFRNNPQPTRQAISNLDKC
LYLYDEIHQVSRSFQQLFDLPLFLSLAQSLLAMSMVSYHAILRRQYSFNLWGLVIKLLIDV
VLLTMSVHSAVNGSRLIRRLSFENFYVTDSQSYHQKLELFLGRLQHQELRVFPLGLFEVSN
ELTLFFLSAMVTYLVFLVQYGMQSQQI-
>DmelGR93c
MIERLKKVSLPALSAFILFCSCHYGRILGVICFDIGQRTSDDSLVVRNRHQFKWFCLSCRLIS
VTAVCCFCAPYVADIEDPYERLLQCFRLSASLICGICIIVVQVCYEKELLRMIISFLRLFRRVR
RLSSLKRIGFGGKREFFLLLFKFICLVYELYSEICQLWHLPDSLSLFATLCEIFLEIGSLMIIHI
GFVGYLSVAALYSEVNSFARIELRRQLRSLERPVGGPVGRKQLRIVEYRVDECISVYDEIER
VGRTFHRLLELPVLIILLGKIFATTILSYEVIIRPELYARKIGMWGLVVKSFADVILLTLAVHE
AVSSSRMMRRLSLENFPITDHKAWHMKWEMFLSRLNFFEFRVRPLGLFEVSNEVILLFLSS
MITYFTYVVQYGIQTNRL-
>DmelGR93d
MKATKYSVGILRFMSFYARFLSLVCFRLRKQKDNNVWLEEIWSNRSRWKWISVTLRIVPL
CIYAFTYAEWISNRMLITEKFLHSCSLVVSIPCYLSIIHLKICHGPEVTKLVNQYLHIFRLGTL
DIRRRSQFGGGRELFLLILSVCCQIHEYVFILVIASRLCGFQHIIWWVSYTYVFIICNSIMCFG
FIWHLSLGVLYAELNDNLRFESGFQTAFLRKQQRIRVQKSMALFKEISSVVTSLQDIFNVHL
FLSALLTLLQVLVVWYKMIIDLGFSDFRIWSFSLKNLIQTLLPVLAIQEAANQFKQTRERAL
DIFLVGKSKHWMKSVEIFVTHLNLSEFRVNLLGLFNVSNELFLIIVSAMFCYLVFVTQCVIV

YRRRYVI-
>DmelGR94a
MDFTSDYAHRRMVKFLTIILIGFMTVFGLLANRYRAGRRERFRFSKANLAFASLWAIAFSL
VYGRQIYKEYQEGQINLKDATTLYSYMNITVAVINYVSQMIISDHVAKVLSKVPFFDTLKE
FRLDSRSLYISIVLALVKTVAFPLTIEVAFILQQRRQHPEMSLIWTLYRLFPLIISNFLNNCYFG
AMVVVKEILYALNRRLEAQLQEVNLLQRKDQLKLYTKYYRMQRFCALADELDQLAYRY
RLIYVHSGKYLTPMSLSMILSLICHLLGITVGFYSLYYAIADTLIMGKPYDGLGSLINLVFLSI
SLAEITLLTHLCNHLLVATRRSAVILQEMNLQHADSRYRQAVHGFTLLVTVTKYQIKPLGLY
ELDMRLISNVFSAVASFLLILVQADLSQRFKMQ-
>DmelGR97a_isoform_A
MRFLRRQTRRLRSIWQRSLPVRFRRGKLHTQLVTICLYATVFLNILYGVYLGRFSFRRKKF
VFSKGLTIYSLFVATFFALFYIWNIYNEISTGQINLRDTIGIYCYMNVCVCLFNYVTQWEKT
LQIIRFQNSVPLFKVLDSLDISAMIVWRAFIYGLLKIVFCPLITYITLILYHRRSISESQWTSV
TTTKTMLPLIVSNQINNCFFGGLVLANLIFAAVNRKLHGIVKEANMLQSPVQMNLHKPYY
RMRRFCELADLLDELARKYGFTASRSKNYLRFTDWSMVLSMLMNLLGITMGCYNQYLAI
ADHYINEEPFDLFLAIVLVVFLAVPFLELVMVARISNQTLTRRTGELLQRFDLQHADARFKQ
VVNAFWLQVVTINYKLMPLGLLELNTSLVNKVFSSAIGSLLILIQSDLTLRFSLK-
>DmelGR98a
MEQMSGELHAASLLYMRRLMKCLGMLPFGQNLFSKGFCYVLLFVSLGFSSYWRFSFDYE
FDYDFLNDRFSSTIDLSNFVALVLGHAIIVLELLWGNCSKDVDRQLQAIHSQIKLQLGTSNS
TDRVRRYCNWIYGSLIIRWLIFIVVTIYSNRALTINATYSELVFLARFSEFTLYCAVILFIYQEL
IVGGSNVLDELYRTRYEMWSIRRLSLQKLAKLQAIHNSLWQAIRCLECYFQLSLITLLMKF
FIDTSALPYWLYLSRVEHTRVAVQHYVATVECIKLLEIVVPCYLCTRCDAMQRKFLSMFYT
VTTDRRSSQLNAALRSLNLQLSQEKYKFSAGGMVDINTEMLGKFFFGMISYIVICIQFSINF
RAKKMSNEQMSQNITSTSAPI-
>DmelGR98c
MEMEAKRSRLLTTARPYLQVLSLFGLTPPAEFFTRTLRKRRRFCWMAGYSLYLIAILLMVF
YEFHANIVSLHLEIYKFHVEDFSKVMGRTQKFLIVAIATCNQLNILLNYGRLGLIYDEIANL
DLGIDKSSKNFCGKSHWWSFRLRLTLSIGLWMVIIIGVIPRLTLGRAGPFFHWVNQVLTQII
LIMLQLKGPEYCLFVLLVYELILRTRHVLEQLKDDLEDFDCGARIQELCVTLKQNQLLIGRI
WRLVDEIGAYFRWSMTLLFLYNGLTILHVVNWAIIRSIDPNDCCQLNRLGSITFLSFNLLLT
CFFSECCVKTYNSISYILHQIGCLPTAEEFQMLKMGLKEYILQMQHLKLLFTCGGLFDINIK
LFGGMLVTLCGYVIIIVQFKIQDFALIGYRQNTSDTS-
>DmelGR98d
MEANRSRLLAAARPYIQIYSIFGLTPPIQFFTRTLHKRRRGIVILGYACYLISISLMVIYECYA
NIVALQKDIHKFHAEDSSKVMGNTQKVLVVAMFVWNQLNILLNFRRLARIYDDIADLEID
LNNASSGFVGQRHWWRFRFRLALSVGLWIVLLVGLTPRFTLVALGPYLHWTNKVLTEIILI
MLQLKCTEYCVFVLLIYELILRGRHILQQISVELEGNQSRDSVQELCVALKRNQLLAGRIW
GLVNEVSLYFTLSLTLLFLYNELTILQIVNWALIKSVNPNECCQYRRVGTCLLLSINIFLSCLY
SEFCIQTYNSISRVLHQMYCLSAAEDYLILKMGLREYSLQMEHLKLIFTCGGLFDINLKFFG
GMVVTLFGYIIILVQFKIQFFAQSNFMQNINSTELKAYTA-
>DmelGR98b
MVAQKSRLLARAFPYLDIFSVFALTPPPQSFGHTPHRRLRWYLMTGYVFYATAILATVFIVS
YFNIIAIDEEVLEYNVSDFTRVMGNIQKSLYSIMAIANHLNMLINYRRLGGIYKDIADLEMD

MDEASQCFGGQRQRFSFRFRMALCVGVWMILMVGSMPRLTMTAMGPFVSTLLKILTEFV
MIMQQLKSLEYCVFVLIIYELVLRLRRTLSQLQEEFQDCEQQDMLQALCVALKRNQLLLG
RIWRLEGDVGSYFTPTMLLLFLYNGLTILHMVNWAYINKFLYDSCCQYERFLVCSTLLVNL
LLPCLLSQRCINAYNCFPRILHKIRCTSADPNFAMLTRGLREYSLQMEHLKLRFTCGGLFDI
NLKYFGGLLVTIFGYIIILIQFKVQAIAANRYKKVVN-
>HarmGR1
MIDYCSFCTRLSRGSFYPTDATSIPLSIFGNKGTSMNKEEHGFRVYNTNTVHKNETRKREM
FQRIDEKDGIKEYDAKDLYGPEITDKDGALLDAHDSFYITTKSLLVLFQIMGVMPIMRVPK
NAQTTKRTTFNWISKATLWAYLVWSLECIIVVKVGRERLANFQSSANKRFDEVIYNIIFLSI
LIPHFLLPIASWRHGPQVAIFKNMWTHYQLKYLKITGTPIVFPNLYSLTWGLCVFSWGLSF
AVILSQHYLQDDFELWHSFAYYHIIAMLDGFCSLWYINCNAFGTASRGLAMNLHKALEAE
HPALKVAQYRHLWVDLSHMMQQLGRAYSNMYGIYCMVIFFTTTISLYGALSEILEHGLSY
KEMGLFVIVGYCMTLLFIICNEAYHASRKVGLEFQVRLLNVNLGAVDRSTQREVEMFLVA
ISKNPPIMNLDGFTNINRELFTANVSFMSTYLIVLMQFKLTLLRQSARKTLKTIVRAVFNTT
TTILDDDFTDEVDEE-
>HarmGR2
MTIPDHLFDEGINNTLLQHDMRHVQQNRIVYEKTQREYEQEQRDMLSSQDGDTCEIHDQ
FYRDHKLLLVLFRALAVMPITRSRPGTITFSWRSTATMYAVCFYIAATAVVMIVGYERIMIL
RSIRRFDEYIYAILFVIFLVPHFWIPFVGWGVAHQVAIYKTNWGKFQVRYYRVTGENLKFP
NLKTTIVMISVGCLLLAVCFLLSLCILMDGFLLRHTTAYYHIITMINMNCALWYINCKGIKI
ASQSLSECFRRDVEAECSAKLISRYRYLWLNLSELLQSLGNAYARTYSTYCLFMFANITIAV
YGALSEIVDHGFGFSFKEMGLFVDAAYCSTLLFIFVDCSHNSTLTVAAGVQETLLSIDVLSV
DRPTQKEIDHFIQAIEMNPAVVSLKGYAHVNRELLTSAISMIAIYLIVLLQFKISLPRDPQIVA
T-
>HarmGR3
MSFHTSNSLFPMTVPIPNGFPVQINSKPKNKIIFLDVTPVSTPIKPHSPNVVVPMRNNLVAPH
ISNDIIYENIKPVFTLLRIMGVLPITRPSACVNQFQIASSSMLYAILVFLSLVSYVLYLSLHKV
QILRTAEGKFEEAVIEYLFTVYLFPMIAVPLLWYETRKIANVLNGWVDFEMVYKQLSGRTL
PVKLYKKALAMAVIIPILSTTTVIVTHVTMVHFKPMQLVPYVFLEILTYMLGGYWYLLCET
LSICANILAEDFQNALRHIGPAGKVAEYRALWLRLSKLSRDTGIANCYTFTFVNLYLFLIITL
SIYGLLSQISDGFGIKDIGLALTAFCSISLLFFICDEAHYASHNVRTNFQKKLLMVELSWMN
TDAQTEVNMFLRATEMNPSQISLGGFFNVNRTLFKSLLATMVTYLVVLLQFQISIPDESQN
RDEEEEVPYNITSATTEAMTTSTTTIMTTVLTTLAKKKKKN-
>HarmGR4
MLNLCVSEIENKSVRNISSMREVLIDKIVKQKLDGLIGRLSGAIFYGNALISLFLSSKFVHS
WRSLSNYWLRMETSTALDFPPDVRIRKRTIYITAFVVSVAVVEHILSMISATGVGFPPEEFLY
RYVTLSHGFILKAQDYTIWKAIPIFVLSKLATALWNFQDLIIILISMGLSSRYNRLNLYVRHI
VSVEKQFESKQRFGTELYLQIQVWRRLREAYVRQSTLVRMVDRNLGSLVLLSNINNLYFIC
LQIYLGIHKSSGSTISRCYFLFSLGWLIFRACSVVLAASDVHLHSQRALKSLHACPSAAYN
VEIKRLQYQLAHDFVALTGMGFFSLRRELLLEVAAAILKYELVLIQYDK-
>HarmGR5
MQNGWNNVISNISVGSVNTVNYLFRTWERLAPNRNMDLYSLEKFKKYKNDWNYPVHVR
YQDQVMAEKEKPRTTFQTAMKVTLTIGQCFGLNPVQGIREKDASKLRFKLLSGRCLFTFFS
LIGQFIMAFVLFLSLFKETSSTVDTATALIFYSFGFTTTILFFRIATNWPKLCMHIAKVESVDP

NTDNKLGKKFNIACISILFLALMEHLFSELHGISIALDCFPDTPVYESFMKLSFQWLFGFIPY
SDFAGGMAHFSNLQCTFNWNFADVFVICMSMYLTARLEQVNQRIIAAKDKNSPSSFWRT
MREDYNRSVHLVRQVDKIIGGVVFMSFASNLFFVCSQLLHTLAGGIKASQRCKPEIGADR
RFFYGYEHSIYFVFSFSFLVIRSLAVSLTASKVHAASLEPAYSLYDVSSANYCVEVERFLDQI
HGDTVALSGLQFFHVKRGLVLTIAGTIVTYELVLMQFTGITPTTSPESVSGVIK-
>HarmGR6
DCSLTRKCNITCAVVLILALCEHILSLLSAFAGASACYSGMDTYEGFVTHFYPWVFSYLPY
SIVLGVITQFLHFQSTFIWNFSDLFVICMSYYLTSRLEQVNRKLLAAQGKYLPEIFWRATRE
DYCRATQIVRKVDEVISGVVFISFANNLFFICLQLFNTLEDGLKGTGECTPKLKKIVVSKSG
PLGGHEAAAYFLFSLVYLLSRSVAVSLIASQVNSASSVPAPVLYDVPSPVYCVEVQRFLDQ
VNGDKVALSGLQFFSVTRGLLLTVAGTIVTYELVMFQFNSSTPTLNITSP-
>HarmGR7
AILSLVLSSRLYRSWGQLSALWARVERIMAVKAPPDKTLKRRMYFFLGFMTVCSLLEHIMS
VVSAIGLDCPPALIIKRYVLISHGFMILRHEYSDWYALPLIFMSTLASLLWNFQDVLIVLISM
GLTSRYSRLNQCLAKICALERKQMDSDKKNETTKVYAWRKLREAYVKQAMLVRKVDDA
IGGIIILSCFCNFYFICLQLFLGITQSKASEPIKTAYYFMSLGWICFRVICVVLAASDINVHSRL
GLKYIYTHDSHSYNIEMGRLQDQLSKDYVALSGKGFFYLSKSILLQMAGAIITYELMLIQF
DDQGTDDV-
>HarmGR8
YNWRTNIAGLWGKVERSVGVKIPVDKTLKCRMSFVAGLMTFCSFFEHALSILASVGFDCP
PSLILKRYVLVSHGFIFMGQDYSEWFAMPLVIISTIATLLWNFQDQLIVLISMGLTSRYRRLN
ECLAKFCELEKQHMDSDKKVEAVKVYTWRKIREAYVKQAMLVRKIDVALGGIIILSCSCN
FYFICLQMFLGITQGMSTDFLTGLYYMVSLAWLCIRVLSVVLAASGVNTHSKLALNHLYT
YETHCYNVEVERLQDQLTKDYIALSGMGFFYLNKTILLQMAGAIITYELVLIQFDDQGSDG
IALNA-
>HarmGR9
MGVESAKVEEVTAAPVPSESGARPSRPTHCVVGGAHAFILRISSFFGLAPLRFESRSNGFTV
SISGAMCVYSYILVTVLVICTIFGLVAEINVGVELSVRMSSRMSQVVSTCDVLVVVATAGAG
VYGAPRRMRNMLKFMENIASVDTSIGGQYSLVTERKLCGIILAILIFFSILIADDFTFYALQA
KKLDREWDVVTNYLGFYLLWFVVLILELQFAFTALSVRARFSAVNDALALTARQVSIPVE
KPKSSSPLNIYAIRVAPVDSQRSANVSLLVDTMTGREHVVIIKRTASGEPRLVVSPCDAVRR
LAALHGTLCDVVNSIDDSYGLPLVVILISTLLHLIVTPYFLIMEIIVSTNRIHFLVLQFLWCVT
HMLRMIVVVEPGHYTIAEGKRTEGLVCRLMTSAPSTGVLPSRLEIFSRQLMLQSVSYAPM
GMCTLHRPLIASVIGAVTTYLVILIQFQRYDN-
>HassGR1
MGQTSFRRNMSFWIPVKKNKVDVAKPKVKNITSFQDALRATLIIGQVFSLLPFVGVFTNVA
SNVKFVKTSWKCVYSLLSLIGQMFMAVLCINKLAKTTVSLNGTSPVIFYVTTCVTMMLFF
QVARRWPALVQHISKAEDMDPNFDCSLTRKCNITCAVVLILALCEHILSLLSAFAGASACYS
GMDTYEGFVTHFYPWVFSYLPYSIVLGVITQFLHFQSTFIWNFSDLFVICMSYYLTSRLEQ
VNRKLLAAQGKYLPEIFWRATREDYCRATQIVRKVDEVISGVVFISFANNLFFICLQLFNTL
EDGLKGTGECTQKLKKIVVSKSGPLGGYEAAAYFLFSLVYLLSRSVAVSLIASQVNSASSV
PAPVLYDVPSPVYCVEVQRFLDQVNGDKVALSGLQFFSVTRGLLLTVAGTIVTYELVMFQF
NSSTPSLNMTSPTVVTHTVTTLAT-
>HassGR2

GYERIMILRSIRRFDEYIYAILFVIFLVPHFWIPFVGWGVAHQVAIYKTNWGKFQVRYYRVT
GENLKFPNLKTTIVIISVGCLLLAVCFLLSLCILMDGFLLRHTTAYYHIITMINMNCALWYIN
CKGIKIASQSLSECFRRDVEAECSAKLISRYRYLWLNLSELLQSLGNAYARTYSTYCLFMFA
NITIAVYGALSEIVDHGFGFSFKEMGLFVDAAYCSTLLFIFVDCSHNSTLTVAAGVQDTLLS
IDVLAVDRPTQKEIDHFIQAIEMNPAVVSLKGYAHVNRELLTSAISMIAIYLIVLLQFKISLPR
DPQIVAT-
>HassGR3
MTVPIPNGFPVQINSKPKNKIIFLDVTPVSTPIKPHSPNVVAPMRNNLVAPHISNDIIYENIKP
VFTLLRIMGVLPITRPSACVNQFQIASSSMLYAILVFISLVSYVLYLSLHKVQILRTAEGKFEE
AVIEYLFTVYLFPMIAVPLLWYETRKIANVLNGWVDFEMVYKQLSGRTLPVKLYKKALA
MAVIIPILSTTTVIVTHVTMVHFKPMQLVPYVFLEILTYMLGGYWYLLCETLSICANILAED
FQNALRHIGPAGKVAEYRALWLRLSKLSRDTGIANCYTFTFVNLYLFLIITLSIYGLLSQISE
GFGIKDIGLALTAFCSISLLFFICDEAHYASHNVRTNFQKKLLMVELSWMNTDAQTEVNMF
LRATEMNPSQISLGGFFNVNRTLFKSLLATMVTYLVVLLQFQISIPDESQNRDEEEEVPYNI
TSATTEAMTTSTTTIMTTVLTTLAKKKKKN-
>HassGR4
MLWIQTHHYIGVESAKVEEVTAAPVPSESGSRPSRPTHCVVGGAHVFILRISSFFGLAPLRF
ESRSNGFTVTISGAMCVYSYILVTVLVICTIFGLVAEINVGVELSVRMSSRMSQVVSTCDVL
VVVATAGAGVYGAPRRMRNMLKFMENIASVDTSIGGQYSLVTERKLCGIILAILIFFSILIA
DDFTFYALQAKKLDREWDVVTNYLGFYLLWFVVLILELQFAFTALSVRARFSAVNDALAL
TARQVSIPVEKPKSSSPLNIYAIRVAPVDSQRSANVSLLVDTVTGREHVVIIKRTASGEPRLV
VSPCDAVRRLAAPHGTLCDVVNSIDDSHGLPLVVILISTLLHLIVTPYFLIMEIIVSTNRIHFL
VLQFLWCVTHMLRMIVVVEPRHYTIAEGERTEGLVCRLMTSVPSTGVLPSRLEIFSRQLLL
QSVSYAPVGMCTLHRPLIASVIGAVTTYLVILIQFQRYDN-
>HassGR5
MSSKEFKQFMQQKKLLLPQQPIHDDFLDVIEKVFHWSCFYGVFVSKRFISLIWSTLILGSLV
IIEALAIWKVIRVLAGVARDMSGHRSVTARLAGTIFYSISILSLVLISKLYYNWRTDIAGVW
GKVERSVGVKIPVDKTLKCRMSFVAGLMTFCSFFEHALSILASVGFDCPPSLILKRYVLVS
HGFIFMGQDYSEWFAMPLVIISTIATLLWNFQDQLIVLISMGLTSRYRRLNECLAKFCELEK
QHMNSDQKVGAVKVYTWRKIREAYVKQAMLVRKIDVALGGIIILSCSCNFYFICLQMFLGI
TQGMSTDFLTGVYYVVSLAWLCIRVLSVVLAASGVNTHSKLALNHLYTYETHCYNVEVE
RLQDRLTKDYIALSGMGFFYLNKTILLQMAGAIITYELVLIQFDDQGSDGIALNATNI-
>HassGR6
IGQMFMAVLCINKLAKTTVSLNGTSPVIFYVTTCVTMLLFFQVARRWPSLVQHISKAEDM
DPNFDCSLTRKCNITCAVVLLLALCEHILSLLSAFAGASACYSEMDTYQGFVTHFYPWVFS
YLPYSVVLGVITQFLHFQSTFIWNFSDLFVICMSYYLTSRLEQVNRKLLAAQG-
>HassGR7
APPDKTLKRRMYFFIGFMIVCSLLEHLMSVVSSIGLDCPPSLIIKRYVLISHGFMILRHEYSD
WYALPLIFMSTLATLLWNFQDVLIVLISMGLTSRYSRLNQCLAKICALERKQMDSDKKNE
ATKVYAWRKLREAYVKQAMLVRKVDDAIGGIIILSCFCNFYFICLQLFLGITQSQASEPIKT
AYYFLSLGWICFRVICVVLAASDINVHSRMGLKHIYTHDSHSYNIEMGRLQDQLSKDYVA
LSGKGFFYLSKNILLQMAGAIITYELVLIQFDDQGTDDVQP-
>HassGR8
RKIDVALGGIIILSCSCNFYFTCLQMFLGITQGMSTDLLTGVYYMISLAWLCIRVLSVVLAA

SGVNTHSNLALNHLYTYETHCYNVEVERLQDQLTKDYIALSGMGFFYLNKTILLQMAGAI
ITYELVLIQFDDQG-
>HassGR9
VRARFSAVNDALALTARQVSIPVEKPKSSSPLNIYAIRVAPVDSQRSANVSLLVDTMTGREH
VVIIKRTASGEPRLVVSPCDAVRRLAALHGTLCDVVNSIDDSYGLPLVVILISTLLHLIVTPY
FLIMEIIVSTNR-
>HassGR10
VVNILDISIRVTDMTQMICDFFQYSVDLFFVYKFGRNLYIEYFRQFEIIDVCLETSCYAEMK
RRLLKTMTFFLVIWFISSFTDLGAWVITYGWMIPVVHSLSYLYLLIKILATLDLIANIIQVEV
RLRIINNFIKNCYNYASACPVGMLADCIRNKNWLYGEDGSPDQSLKARSIDSHEIKRLSKC
YLMLTEQVMFINKMYGFRVSTYIDTIIIRSYGSCTTTDIKQCNCRNKKFLSFPDIEQDT-
>HassGR11
HFAAIFLWIMNDLVLDAVFCSNCERFYLTVEEAQMTCIQLLKNVTCPKSQRHLYKDVLYA
NRCFTKMTACGLFTIDAMLPISCIGAVGYYALVLLQF-
>HassGR12
MLKSQLVNWFNNFIDKDLQSMLLPISLMQNFTFCPKFRIKNNRITPISSVHKFVALVGTIMF
IYFYVQRVYIQSVGNYLKNNFFEVYTCCYYSIGIAMNFIDSVIQSKLYVDFILLIQKVHRLL
NDENHFRLYVISNWLRIIAVYSFFIFILIEVSIWIRLPSYYIASCYPMVAFDLNSVYAISLITLL
KDKIILWNLRVSNLQAMQEENEPKKMYQIYINILKCYEIYAKCFER-
>HassGR13
ARFLDVESSVKLCQNLQKVDNILRLKQFKRYNEQQYYWNIIVLVFIITSFECGFLVHIWYT
VEYPILAFFAGIGLLNVYMELVLAASLIVYLAIRLKLLNKITHHNFKIKDYYNNTRAAVVV
DEHLLVNSDVKDANIDLANFLICMKE-
>HassGR14
LNIIHSDCSISLILTLQKVFDSLDFSDKIMVITRWNWFAMCIAFGTNISLYMLYYVTYHYFNP
VDLVMDIMFITFDLNLVYGILVITWLRKFLEKWIEDVLAFDDGDAEFYSDYFQIYRNILNA
YNLYKTLFQLLVRI-
>HassGR15
IFVILLLGLDYLVIQKYDKILFDRETIYYLSICVTGLQTITFLCNIINVRFINGDANVELLVNL
QQIDRRMNINRNKSITTLLVKANVISLVVVLIMFIALLGVASAKGTAAFWPYIGIAYSQFSF
VIEL-
>HassGR16
ITVLPLYFIQRVEIFIFCGAVYMLKCRVKIINNYLKEFIQKQDKKSVMVFTVGKAKPKPDTT
LNYIGLPSIRNAKIRDLATAYD-
>HassGR17
NNSSNKVTEMFLENRLDKEVERTAFSFNFPLNLLLFSKYRLKDNRIYPNGIKYGIYALLCTL
FLGVLCFYRIYTSDITNASMSSIERAILTVVPIIFFIIYFLGYVIVFVSDIMYKDNNVLLILTIQT
IHRGISF-
>HassGR18
FSKTIHSFNMWSHISFATVILVNLITRATFYLTCRYPYVSDLISDIIRDFSFITVDVNMVIATRII
ILLKQYIDLWIKAILTVNMAQETDLYCQNLFGVYMNILKAYNIYRKVFQVLVSF-
>MsexGR1
MNKDNHRFRIYNPHQHERVRKTDASRKSDGKDIKDYEAKDVYGPQITAKDGPLLDKHDS
FYLTTKSLLVLFQIMGVMPIMRVSKNAQTTKRTTYNWISKATLWAYLVWGLECIIVVKVG

KERLANFQNSSNKRFDEVIYNIIFLSILIPHFLLPIASWRHGPQVAIFKNMWTHYQLKYLKIT
GTPIVFPNLYSLTWGLCIFSWALSFGVILSQHYLQDDFELWHSLAYYHIIAMLDGFCSLWYI
NCNAFGTASRGLAINLHKALESDHPALKLAQYRHLWVDLSHMMQQLGRAYSNMYGIYC
MVIFFTTTISLYGALSEILEHGLSYKEMGLFVIVAYCMTLLFIICNEAYHASRKVGLEFQVRL
LNVNLGVIDRSTQREVEMFLVAIAKNPPIMNLDGFTNINRELFAANISFMSTYLIVLMQFKL
TLLRQGPRKVIKNLVKAIFNTTTTLAVDYDDEENEE-
>MsexGR2
MMMIPDHLFDEGINNSMFENEMKYVQNKKTVYDRTQRDYEQEQRDLLNSQDGDTCEIH
DQFYRDHKLLLVLFRALAVMPITRSRPGTITFSWKSSATMYAICFYIAATAVVLVVGYERL
MILRSIQRFDDYIYAILFVVFLVPHFWIPFVGWGVANQVAVYKTNWGKFQVRYYRVTGEN
LQFPNLKTSIVIISVGCLLLAVCFLLSLCALLDGFLLRHTMAYYHIIIMINMNCALWYINCK
AIKIASQSLAESFRRDVERECSAKLISRYRFLWLNLSELLQSLGNAYARTYSTYCLFMFFNI
TIAVYGALSEIVDHGFGFSFKEMGLIVDAAYCSTLLFIFADCSHKSTLKVAAGVQDTLLSID
VLAVDRPTQKEIDHFIQAIEMNPAVVSLKGYAHVNRELLTSAIRMIAIYLIVLLQFKISLPKE
PQAAAIV-
>MsexGR3
MTFYTSSSLYTTHAIPNGIPVRMEEKPKNKMIFLDVTPSRTPKPASPNAVVPMKNNLIDPHI
YKDIVYENIKPVFTVLRLMGVLPLTRPSPGVNQFKIASPSVVYSLILYIALISYVLYLSLHKV
QILRTAEGKFEEAVIEYLFTVYLFPMLVVPIIWYETKKIAGVLNGWVDFEVAYKDLSGRAL
SIKLYKKALIIAVIIPILSTGSVIVTHITMVHFKAMQIIPYVFLEILTYILGGYWYLLCETLSVC
ANVVAEDFQQALRHVGPAGKVAEYRALWLRLSKLARDTGLANSYSFTFVNLYLFLIITLSI
YGLLSQISEGFGIKDIGLALTAVCSIFLLFFICDEAHYASQNVRTNFQKKLLMVELSWMNTD
AQTEVNMFLRATEMNPSQISLGGFFDVNRTLFKSLLTTMVTYLVVLLQFQISIPDESHSWES
HDNELVSNATSSVTETTTTTVMTTIMTTLAKRKKKN-
>MsexGR4
DEFLPTIANIFKIARIFGISKYGFNLAFMWTLMIFITLISVEVTSFWKFVKVLDGWAEDKFA
RRGFTERVSGSVFYGNATLSLILSCKFVNSWKRLSRRWRKVEIEGSLRFPPDRWIQWKVTA
VSAFIGVCALMEHILSMMSAIGLHCAPSQYLRKYILNSHGFLLRINEYSLWFAIPIFILSKIST
MLWNFQDLMIIVISMGLTSRYNRLNMYVGHIIKIERKLSDSPKVRSDLHVHNEIWRRIRES
YVRQAELVGMVDKEFGALILLSNINNLFFICLQLFLGLNATARGALINKLYYFISLGWMLF
RACTVVLAASNVHMHSKKALVFLYSCPKSGFNIE-
>MsexGR5
EETCGQKTQSQQINVTKIYAKNKVSDLKNTNEDGNQFLSFQEAMKIMLIYGQCLGLLPVS
GIFEKDVTKMRYRWFTWKVFYSLAIGFLQGTAAILCVENIINLTFTIRGLAHIFFYLITCSCT
FMFLRVAVKWPSLMKELYNCGLQYYIDPKTKSKCHWACYFILFLAIYIFIICISIYLSSILNSII
KKIDSVYKNNYLPPSFWRSLREDYNRATQLVRSFDDSISGVVFISYANNMFFICLQLYYSLG
NGLKGRQNYFRTACPNYPSGPFGGYEASIYLIYTVAFIFSKFLAVSLITSDVHTSSMKAAPA
LYNIASSMYCEEIQRFLDQVHSDTVALSGFQFFYVTREIILSVVGTIVTYELVMLQFGS-
>MsexGR6
MGRQNTQKNLAFWVPVKRNKVHPANLKKQTPATFQKSLRATLLIGQAFSLLPVVGIFSND
ANNVKFIITSWKCFYSFLSFFGQIFIVVMCIIRVVSTEATLNATTPIIFYGTTCFTMLMFFRVA
TAWPDLVQHVAKTEELYPNYDNKLTRTCQITCAVVLLLALSEHILSLLSAFAGAIMCFPNKS
VYEGFARHFYPWVFNCLPYSPLLGMITQFLHFQSTFIWNFSDLFVICMSYYLTSRLDHVNK
KLAAAQGKYLPEIFWKSTREEYCRATQLVRKVDEVISGIVFVSFANNLFFICLQLFNTLED

GIKGNGDCNSRSKKTTPLSGYEAATYFLFSLVYLISRSVAVSLIASQVNTASMVPAPILYDVP
SPVYCVEVQRFLDQVNGDKVALSGLQFFSVTRGLLLTVAGTIVTYELVMFQFNSPTADTDT
TTLIPPNISTTATP-
>MsexGR6.2
SQNKLNVSASIIFYTSNCLTTVVFLRIATQWPNLCREISSAETTDPNMDTNLMKKCNRSCVI
VLILALLEHTLADLKGIATAVDCEPHRPLYEGFIIHSFPWMFSIIPYNTALGFFSHFINLQCTF
NWNFVDLFIICMSLYLVSRLEQVNKRIVAVKGRYLPPSFWRTMREDYNRAAHLVKVVDDII
GSVIFISFASNLFFICLQLLHTLADGIKPVPRCRVGVPDKRPLRGYEQAVYFVYSFLFLVARS
VAVSLTASRVHTASREPAYALYEVSSETYCIEIQRFLDQIHGDTVSLSGLQFFNVKRGLALTV
SMFYY-
>MsexGR8
MSPEMAWSSNSVLREKRLLNGGFYKTIRLTLLTSRFAGVLPISGLTNHSSKLTRYTWKSFYS
LIYVPRLLLQVLLFSYLLVDLIRGGISIQKLFVCKYMGTFIIESIITLRLCIKWRTMITEVEAIE
RKLPPVNKPRKAVFVANLTMFAIQLCWAVCHWLYLMFLAKMTLICYLQPDDADSYIKSYL
LVNYSWLYDQIRFSYFSGLILQVFQAQASFISNFITILVAITSMYLRNRFQTFNRIFFQRQHKS
RHSKPSWMELQMYYARLTRLACVVNDYIHPFVFTTFWSDLFFISFQTYYTLNKLTLDNFY
FNKCFESHRFDKESVAFCVYYFIFSVLKLSLVWFLAAELHETSRKPLNALYTVSSKAYTVE
MQRLMTQIYRNKICLSGLNLFDITRGTILTIMATILTYELVLLQI-
>MsexGR9.1
MSEDDDFDGLESTKVEGATAESSSPSTDHGTDYPSYCIVGGAHAFILRISSLFGLAPLRFKP
HEKGFSVSLSSAMCVYSYILVTLLVILAVFGMVAEIQVGVEFSVRMTSRITQVVSTGDMLV
VVVTACAGVYGAPKRMRIMLGFMEKVASVDNSIGCQYSGLTERKLCAILLAILIFFTVLIA
DDFCFYALQATKVDREWDMVINYIGFYLLWYVVMILELQFAFTALSVHARFRAVNEALAL
TARQVAVPVDLPRCPPLNIYAIRVSPVVSQRSANVSLLVDSMPTKESTVIIKRIVSGEPRLVV
APCEAIRRLAVLYGTLCEVVHSIDDSYGLPLIVIIISTLLHLIITPYFLIMELIVSTNRIHFLVLQ
FLWCGTHMLRMFVLVEPCHYTANEGKRTESLICRLMTSTPSNGVLPSRLELFSRQLMLQS
VNYSPMGMCTLDRPLVASVIGAVTTYLVILIQFQRYGT-
>MsexGR9.2
MSEDDDFDGLESTKVEGAAAESSSPSTDHGTDYPSYCIVGGAHAFILRISSLFGLAPLRFKP
HEKGFSVSLSSAMCVYSYILVTLLVILAVFGMVAEIQVGVELSVRMTSRITQVVSTGDMLV
VVVTACAGVYGAPKRMRIMLGFMEKVASVDNSIGCQYSGLTERKLCAILLAILIFFTVLIA
DDFCFYALQATKVDREWDMVINYIGFYLLWYVVMILELQFAFTALSVRARFRAVNEALAL
TARQVAVPVDLPRCPPLNIYAIRVSPVVSQRSANVSLLVDSMPTKESTVIIKRIVSGEPRLAV
APCEAIRRLAVLYGTLCEVVHSIDDSYGLPLIVIIISTLLHLIITPYFLIMELIVSTNRIHFLVLQ
FLWCGTHMLRMFVLVEPCHYTANEGKRTEGLICRLMTSTPSNGVLPSRLELFSRQLMLQS
VNYSPMGMCTLDRPLVASVIGAVTTYLVILIQFQRYGT-
>MsexGR10.1
MFKSKKSRPPECVVPKSIGVVLRISQAFGVAPVRLTVKPDGYVVQFSPSVSLYGYAIIIAFSL
TSVIALGFDLSVPLGQSVRMRSETRRVVWVVDLAIVTILGIVGGYQAPAHMKSLIDIVNRV
QKISSELKVSVKSPKTSDNRQYRYVIIALIILFTIMIFDCFNIIYITLTKEHRVWVAAMYSCYY
CSYIITQLLEIQFILLAAYILSALKLINSRLRNLQKKYNHYLFSPAKGEDAQTIRHLSLSYGA
VCEIIKEMNKSYSTVLLLLIGSFLLHLIVTLYYIITNIFFKDIEYCMFEKIITPILQVVWCAFHT
WDLVMVVEPCHRTHEEMERTKELISQFMCTLTLKVSDPMQVELELFCRRLMFSQVKYSP
HQVCTLTRSLIATVLGSITTYLVVIIQL-

>MsexGR10.2
MFKIKKWLPPECVVPKSMGIFLWMSRAFGIAPVRLTVKPDGYVVRFSKSICLYGYAIIFTFS
LITMLALGFDLAIPLGTTRTPSETRRVMWVIDLAVVTIIAIVGGYQAPAHMKRLIDFVSRVQ
QITSDLNASSKCPKSSDDRMYCCVVFALIFLFTILFGDLLSYIYIAIEKEERVWVAVMYSGY
YCSNVITRLIKVQFMLTATYVLSALRLINSKLKNLQKKYDQYHVTSAGESDAQTIRHLSLS
YGAVCKIIKEIDKSYGTVLLLLLGMMFLHLIVTLYYIINDIIGIDSTGYDKFETFTKPILQAIW
CAFHTWNLFMIIEPSHKTHEEVERTKELISQFMCTLRLQMSDPLQMELEVFGKRLLLDCVA
YSPHQVLTLTRGLVVTIFGSMTTYLVVIIGL-
>MsexGR11A
MLDIKAIKIFLFIENILCIYRNYTFYKKKTQKIVLFHVIITLSFCVAVVANDIYVTYEYFRGNL
STLFLILIYHFQFTVYTMLSIVCGISQSSSFEELINTLEKMHNKFKDDVYHANSLKKMNTH
CILPVVYFIVLTLSVLYERMNYVFLDHLIVFDVLQYISEIAMSYCYLTQYFVYSLYLTIIYEL
FKRFYILLNEVKTKLDEKIVAGEKITDNINLKIIEEWAEMYGDLVKISKAFRKCFSGQNFKG
KGGGVTMMMTMFFDITYHLTAVTTAMLAAQRVQNGVPVLKRILASFYNILTAYPNRPELG
RIKNFSRMVAQNPLSTAEHRPPLKRATLFGPQLSLSSVHRRSYEYRRTSGTEVVLRYELYH
DPESPHLRPPIDPKDFKASNTPKEKIREKSVLEQVHHFFVRMRQFDLLLNGVGSNRMTTT
KDDYPSAVATILPACWNRIYTGCSWNAAHLNSRLMELENQYHFARLGIRFRDLRVASYRA
FKSSDDYLPPWDGREGVSDFY-
>MsexGR12
MCDIKPINTIIFVENMLCIYRNYIFYSENAKRIRILRITIEIILYAAVTFVDVIILYHHFQKSSET
VLLIILNNILFLGYSILCMINGMRNSNYFKELIDTFGKLHRNCGNDITYNKSLNCLNLTFVV
ASVTYSVILSVVLTQDKINHEIILLIWVKIIKWYRVISEYLVYYILVTILHIFFKRFNGSLDKT
QRLLNENIIKVGDMTDQTNLKIIEESTEMYTIPV-
>MsexGR13
MRSISTISKVSPQVKYNNFLYKSITIIKQIKYLIYVQCVLGLSRLYLLQSKKICLYTSYLYAFFI
ILSTLYHESSTIGSPYHTFVLIQGVMCFEYILLVINALLTKKKKMHNYFKNLSQFDQTLKVS
SDNETLNAKKGSLIWIFIIFIYNAIEICMILFKIMNSNEVTEYVNMIFYYIISLVHDMEQIFFFT
LLRTIFTRLKLLRAHVRKTFRVNDAGTSDMKTDKIETLSNNAQLDIGSLHKVYELLHKCSE
QLNSIMSFPMLMMLFTSGFSTTLLLKILVKIIQVQETMDPKIVTIAVIYLVVRCVKYTLLVV
MPCYYSSITSSQVATIRTTLHDSLNTMPLDKLERRRVKAFFLLTKESEFVYALAGFIRLNMT
LPLSYTSLCTTYLVIIIQFSKFLD-
>MsexGR14
MAPKIYNMIPKIYNSKARSRCYEAILFILLLCGYNYNYFDKNMFYKITAKFYCLILVSVMV
YTTFACRNTQNWPHIWLLLEYVFSALIIILFYRTKMNLFLQKLKLIDMYLRINYRHYNWEL
WKIIGFTIVLWVLRIGHMYLYCAKDWCYDIFSIFLISQFSTIALDVNGVWRCAIFDIIRYRLK
ILRIRLEESPENNFYLYVNKNKTLKENKIRLMLFIYRTIADVLAMISPELDATVSITFYLEPTL
LFVCGFLMLHTLQIILFLFTPFLIVEFYSIEVERILLFLIHKVIDETDPGTKVDAKLFIKYIQIR
SFRYKIWRILRVNADLPFELINVCVNFVLVLRNFMHLY-
>MsexGR15
MSAYQKMVQLSSYVTVLNIIKLWRLACFQYLHTEHFRIIVWPFKLYCVILEIFILYGSYDSF
TIPFFLNTVQYICISLHSLKTGDEYLYKYILAANTNDVIVGRRTPVFDKEIRFAIIFLIISRFITI
CISTSKFHSIFLCVQIVILSTRLTGLVLIIIFLMLYVRMKYLRRRFERNTIPVNIIHKNGVVSKT
REMRKCLIYYNNLLDNFSDIDQELQILTWVFSDESLRFELEIALEYVERRPFNFVICRVFPV
DISMPISIVTLCITYVIIIIQFAEI-

>MsexGR16
MFTYKEKIKSYSKHMTILNLIKTWLFCCGHYVVTITAKPLIYVHKVFCIVFAGVVAETIFVK
GNVSFFILGIEYILSVTLSLMFGHEYLSRFYRAIKINDVMMGIKKESIFDDVIRIILFIFLTVRI
SLTCLEIYFQSGFEYQMSCVCITVVSSNMICMIISIILYTCYVRMRLLRRRFEEITIPVNIINEV
GAMCKVREVRRCLQHYHNLLDIMKDIDREFKYLVNIFKPCFSLSVMWQHDTLAKEEHLS
PRSLDIFHDESVRFELQRALQYITIRPFEYLLWGAVPLDVTFVVKLITLCITYVIVALQIVKF
TGMI-
>MsexGR17
MNSELTKYFTKHLTILKMLKFLRILMGYYNPTNLSQPLVWTARAYSFTIWLITLYSLIKYFF
VLYVFHIADITLSLYTGDEFFFKYYRATETNDIIIGNKLSVFNIGIKFIILVTIAFRLLLTCLKIR
HFKSHTNFNLNFFTINFVIIASRLTCLLVTTVLVVLYVRMKYLRQRMERNTVPVNIISKNFIL
SKVREVRRCLLYYNTLLDSVADIDRELQCSVNIIYILTRLNLTCLGYENHLQMFT-
>MsexGR18
MSSFKKAVDIWYKNVSILGLIKVLKFITAHYFYTGSCKFVKCVYLLYCIVLCVVSAYGAFP
LIPFRSIEYVLSVIISLLTADDYIFRYFRSLETLDVVMGIKSRSIFNNKVRCLVAYMTLFRILFL
FIHYSGDEDVYSLLSYNIPLLNQRIDSFVSVVIFYTLLVRMKILRVRFERNPIPINIVNKNNV
MNNINEVRRCLLDYNNLLDNFHDIEEELQYLMAIIIIFCMPKLITLDVELIGSVLLETWDFS
KLMLQIEVTQGFILVCLPAVIADLLRHEVAKIKKTLLRQAISCSDESLRYELELALDYVTRR
PFNFVLFGAVPLNMDVPVGIISTCITFIIVTIQFTHFNNI-
>MsexGR19
MLRKVNNLFKTEEVKISKPITMIMKIIHILYSLDMGGFNIKTKKVKRITKVLSLAKCLIVGII
TVVTMYMSHKNVTSSMYYVQIGHNVIATLTFAYFDSGMTYCDYQKILLKFDTALRINRKK
QYLDIKMFIFAITGVALRLSFVFVYCSFFDASCVRPLLARQLSIFLFISFDLILIVYFFIFYTTY
WRLSNIVSIIDNSSTSIVFLQYLYKGLIDAIQTAKSAFDVVVSYINLLAFARGFARVKVFSRI
KFHYKNTYSDIKRFISYVEARPFKLRACRIIPLDTSLPVHALNIFITYLIIIVQFTHLF-
>MsexGR20
MFEKMTTKERKLKSLDLQKTTVSKAMFIFFVLIHVIFCLDFGFIRFKSSKAKLVFKALTLFL
SAVIVSTCFISFYLSSDIHTHITSSMHLLRYIIYVFILSFRNDSTLFDLQETLNIINSKYGYTSF
KLDIKIILCTLSIFMFDTVLAIMKYTLIDPNCEILKPLSLLIIVPIIAIDFVLIVYFFLFYSVFCH
LRVFSLHLPKSDIVVSQTVYKEFVDITEKTRKVFDCACSQIKFRILSFVIIKMAILTFAPVLA
CEKLAFQAEDIKAILHDRLLEEKNECNVKYIKRFINYVAARPIASRALKIVPLNFSLIIIILQL
CIDYIIITIQFSHLY-
>MsexGR21
MPDQLVCNIDFYSYTRFFCGFYHEFQTSTPVRWLARLYCSLICASITFCYNLLVYDHIVLKI
LACISSMEYILYMLISLLKSNKYLLQYYKKTPLIDISVSTYRLMRVCLVGYICVMFLLTLSY
FTQNLILHEESNVAKYVCALINFIMWFIGIIGRSPLLFVFALVYTRVRLMPQTLQSNDFDCRI
LGQQHPRRYIQMYEAIMDGLEAIDGTVKLQTFNFLCRRARPRGDPEAVPIPETQPVPVQLV
ARGAAQRAQSVVFLLLYCHYRYRDFANTNLDYIIQFIYAVYGLIR-
>MsexGR22
MADQLACDINFFYYIRFFFGFYHKFQSSRQIRWLAKCYCCLIYVNIIFFFNYLKTYRIYPKL
FIYFISVEYLVFISISLLEGNKYLLKYYKKKPLIDASASTYGPMRVCLFAYMCLMLLVKLLFI
IEVSIVHREYTTLEHLFNIFGLITWYVIIIGRSPSLFVFALLYTRVRLMRQTLENNDFDCRNQ
GKNHPRRYMQMYEAIMDGLKEYEGPKKLQRDHEEIQKLFQFLESNTLEYCVWRVLPLNL
RSLLSFLSFTITNSIAILQIQDWAA-

>MsexGR23
MADSPVCNMNFYWYTRFVFGFYHEFQTSKPVRWLAKCYCCLICASISFFYLYLPSDAFYT
KKFLFLVSTEYFMYILISFLNTNKHLLQYYKKTSLIDASASTYRQMRLCLVGYLCIMFFIRL
LGFIKVLILREEYNLQQIYFSFIDFIMWLVVLIGRSPLLFVFALLYTRVRLMRQTLENNDFDC
RIQGKNHPRRYIQMYEAIIDRLEANNETVKLQVTNTH-
>MsexGR24
MCLDERDRAEIQKLYQYLKSNPFRYVVWGAVPLNVHCILSFTSSCITTIIAILQINDWVA-
>MsexGR25
MPDQLVCNIKFYLYTRFFLGFYHEFQTSKPVRWLARCYCFLICACVIFCFNFLIYSDLYPQT
LINLFSMEYSVYMLISLLKNNRNLLQYYKKTPLIDASACTYHPMRIRLVGYTCMMFLIRFS
SFMKVVIFNGPYTKNELECWFSLFDFIVWFINIIGRSPLLFVFVLLYTRVRLMRQTLESNDF
DCRILGLHHPRRYIQMYEAIMDGLEASNTDLKLEVIDISPIHGTKARIYRDREEIQKLFLFL
KRNPFEYSLWRVVPLNVCSLLSFFSFTVTTIIAILQIQTWNI-
>MsexGR26
MPDQLACNIDFYLYIRFFCGFYHEFQTSTPMRWLARLCCLLICASITFCYNLLVYDYIVLKI
LACISSMEYILYMLISLLKSNKYLLQYYKKTSFIDISVSTYRLMRVCLVGYICVVFLMMLSY
FSRILILHEENNVAKCVDALIDFIMWFSMIIGRSPLLFVFVLVYTRVRVMRQTLQSNDFDCR
ILGQHHPRRYIQMYEAIMDGLEAIDGTVKLQVTDILHICSTRDREEIQKLFLFLKRNPFEYS
LWRVVPLNVCSLLSYLSFTVTTVIAILQIRTWTT-
>MsexGR27
MPDQLVCNIDFYLYIRFFCGFYHEFQTSNPVRWLTRLYCWLICASITFSYNLLVYDHIVSKT
LVCISSVEYVLYMLISLLKSNKYLLQYYKKKPLIDASPSTYRQMRVCLVGYNCVMFLTTLS
YLLLIPITLRGEYTGPLYIYGLFYFLTWFIGIIGRSPLLFVFALLYTRVRLMRQTLENNDFECR
ILGKHHPRRYIQMYEAIMDGLEASDGTMKLQVTVTRYFNWLYIYFRRLIFCIDERDREEIQ
KLFLYLKRNPFEYSVWGVVPLNVRSLLSFFSFIITTIIAILQIQTWTT-
>MsexGR28
MADHLACDINFFFYIRFVLGFYHEFQISPRMRRLTRCYCCLVFVFYLILTYSTNPNYHSVFV
IFVSLSELSFYIVYSLLMADRYLLQYYKKTPLIDEGANYRSIRVSLMIVMCTMTALKILIITI
NLAYKKGLTIYELYYGFLKIVAWICIIMGRLPGLFVFALLYSRVRLMRLKLENNDFDCRTLG
QHHPRRYIQMYEAIMDRLEATDGAVNILGGRHYICKHNKER-
>MsexGR29
LCVVERDREEIQKLFQFLKNNPFQYNMWRVVPLNVRSLLSVLSFTLTTSIAILQIQG-
>MsexGR30
MLFKPLLIIQAPLGCARINDFGISIQPLKTWQKIYSICYMILCALAYILYTTNKEGAPTLKSGI
VEFIFSAAQLITNTVVNCAVVIVNNLIYTKENVYIFNCLQHIDTKLKVVGKPEHYNAVFNFI
ICALIFNSFFHVYYIIWGVKSLPLISKWVPLLSHLMIVDDLEMIFIVSVVFLMYKRVKYINKI
LNQFSSTKWRHSTHVNESVVFRRMWEAFIGIATTVRCLENTSSIY-
>MsexGR31
MWPRFDGVNKHPLRTLFKPLLIIQAPLGCARINDFGISIQPLKTWQKIYGISYMILCALAYIL
YTTNKEGAPTLKSGIVEFIFSAAQLIANTVVNCAVIVVNNLIYTKENVYIFNCLQNIDTKLK
VIGKPEHYNAAFNFIICALIFNSFFHVYYIIWGVKSLPLISKWVPLLSHLMIVDDLEMVFIVS
VVFLMYKRIKYINEILNKFSSTKWRHSTHVNESIVFRRMWEAFIGIATTVRCIEKTSSVYV-
>MsexGR32
MSANDNSKLILIAQIPKDLFFASIKFMLLCRFFCGLYYEMSSSRVVQLLGQLYCVSMLFMR

VTLLISLLPFMNLIPLYLFIFILIVLSVTVSVASLLSKDIYFMEYLSDLEGHAANVTQKTLTSF
IPFIITLTIIFGNVLISHTFSINMVIKPTIIIPVSMFTFYAYFYPVIVMLHIHYNEIKELQHKLRVN
LNEDLIEDEKINAIPSFLNEYRFLFSVGVLSQSARMLCTIYYYLSEELGIEVRFLLNGFFDVI
MSIGITFMPVMLLEMSMHITNDIQRMLSRHLLQYKGPRLREAIYDSLDYIEIHGMKVTLW
AHFPMDLSFFFSFVSLVTAYAIAVLQFKY-
>MsexGR33
FCSYNVISVREEEVRARSRYRRVKHIEIETDILKYLNIISKMLKVISSIKFVLLYRFCLGLYYE
MGPSKVVQYFGKMYCILLLFLKEALYTYDLLINSYSFSILFHRLISMILILVVPLASVVNKEI
HFMKYMLELNDHSRDFRENLNSIIPFIVTVTGLTYKITMAVFIYTKHTYFMKFLPSFIAMFS
YYPYYYTYIVMYHVLYNEMKVLQRKLRVGLTEDLTEDEKINVIQRFRSSSVPSVIRFLFKT
LNKPSKTIRITLSFGVVTQWLRMMNMAYYNISENFQGITDALFGVFYESMVIFLPAILLEM
SHNVADDFKHILSKQLLQYQDYRLRQSVYDSLDYINTHSMKFSLWFQYSMDISLLIRYATF
GTTFIISLLQFKY-
>MsexGR34
MTREINRVLFARTAFVTKEKVPKEVMLGTQRILDAFADFMNLFEYTVFSFVYQVMVCNLI
AVQNLISSLKPQGIGDIRVFICIVSVLLSMIIMSIYDIYTFGIRLPLNLFAVTASYTTDTRIKKC
EYVSKDILEEDFVKSFKLLIYIQMTLGSCRVYNKDRFLTSPTIFQKIYTILCIIITITLYAVILND
YSWKYGKTSYLYYLITAVFLVDLITYICNMIQVRFLNGDANIDFFIHLQNMDRLMKIEHNG
TIWTTLRKINNVSLIYFIAGLFGYLIFAIYKGGLINWSYSSLVNSQLTLMLEIGNCSNFVAHFI
IRLYIVNYIMCNHLNPNKSKKRPKVYLTVTGLKRWIRYTTAKTHNFALNDTEKYLKAIFDG
FKKFQEIYKFQCVFCFKIVIMSLITFEFVLTGVQNNLLKLAEIITVVMFSVINYLVALVLSVR
YELFFREVKRTKRLSISMLRQYQEGPLREKAKRMLKMIEESPPSFSVYDMWYMDASIFLK
MINLVTSLIVTLLQFAFL-
>MsexGR35
MSNIKKSELEKTVDILLNNAIGKDIQSIMRPLNLIQNIYCAPKYRIKDNFIIPNGIYENLISFS
AVLALLIICVSNIAFSSCPIKILSADLICISDVLNEVISLMGFIINSWVSITQSNNNVQLLLLLQ
TNHRLMEFAERDVSLLKISNWIKVIALYSCSLGVYTSLHMYFHIPYIHTVFFDVIVLCFDM
NMVYAIFIMKLIVKNLIHWHKEIQKPENALILYKDRMFHLYMNIMLAYKLFKKAFQLLVSS
SIHDFY-
>MsexGR36
MPDQLVCNINFYLYTRFFVGFYHEFQTSTPMRWLARLYCSLICASITFCYNLLVYDHIMSK
MLARIISMEYTMSVLISLLKSNKYLLQYYKKTPLIDASPTTYRPMRVCLIGYNCVMFLMTL
PYFLQISFATCGENIRPKYIFALIDFIVWIIYIIGRSPVLFVFALLYSRVRLMRQTLESNDFDCR
ILGKHHPRRYIQMYEAIMDGLEACDGTVKLQTNIYRRFFCTDERDREEIQKLFLFLKRNPF
QYSLWRVVPLKVRSLLSFFSFTVTTIIAILQIQSWNT-
>MsexGR37
GTAANIFKTYVNICEMADPLVCNINFYWYIRFFFGFYHEFLDVSSKPVRWLAKCYCCLICV
SVIFFYLYIPSDGFYSKYLLYLITTEYFIYVTISFLNTNKSLLQYYKKMPLIDASPSTYKPMR
VCLVGYLCIMFFIRLSGFIKVLILHGEYNLQQIYFNFIDFIMWLVVLLGRSPLLFMFALLYTR
VRLMRQTLENNNFDCRNLGKHHPRWYIQMYETIIDMLEEDNDTVKLQYYFYMYIRRLDL
CVDERDREEIQKLFLFLKRNPFEYSLWRVVPLNVRSLLSLLSFTVTTSIAIMQIHGWIA-
>MsexGR38
MADQLVCNINFFLYIRFLFGFYHEFQTSKLVRWLARCYCCLICANIIFFLSYFHTYKLYPKIF
IYLLSMEYFMHMLMSLLKSNKYLLQYYKKSPLIDTSVSAYRQMRVCFVRYISVLFFIRLCG

FLKIFICHGELNLHQYIFSFMDFIMWVTIVIGRSPLILVFALLYSRVRLMRQILESNDFDCRN
QGKHHPLRYVQMFEAIMDRFEASDKYYIHMFISRLTIDERDREVIQKLFLFLKRNPFEYSL
WRVVPLNVRSLLSFFSFTVTTIIAILQIQSWNA-
>MsexGR39
MPDQLACDINFYLYTRFFVGFYHKFQTSTPVRWLARLYCWLICASITFCYNLLVYDHIMSK
MLVWILTMEYIMYELISLLKSNKYLLQYYKKTPLIDASPSNYRPMRICLVGYICIMFLMRL
YSFINNLILHGERSVADHLYGLIDYIMSFIGIIGRSSVLFVFALLYSRVRLMRQTLESNDFDC
RILGKHHPRRYIQMYEAIMDGLEACDGTQDREEIPKLFLFLKRNLFQYSLWRVVPLNVRSL
LSFYSFTVTTIIAILQIQSWNT-
>MsexGR40
MTDQLACNIDFYLYTRFIFGFYHEFQTYKPVRWLARGFCGLMSTCIIFFYDSFFIRSLNAVFI
MYLQNIEYVLYVLISLLKGNDHLIHFYKKTPLIETRATNYTPTRVRLMVYICLMFLIRSFGV
IKTIFIHRDKSVTEHHFAIIDFIMWFTMLLGRSPLLFVFALLYSRVRVMRQTLESNDFDCRIL
SKHHPRQYIQTYEAIMDRLDENTNSMKLQVLHRYDLCPDERDRVEIQKLSQMMRSRPINF
TLCGVVSLNVRSVFSFISFTLTTFIAILQTKQWST-
>MsexGR41
MNNSQQNTYKTFTPLIWILKIFAINSNVEDARMTLSSIIRIIVTASILGTLIIISFYFKVKYIFSD
IIISIKLTDAIQMVYDYCQYIVDLYFVYKYGRRISAEYFKQYDKLDKVLGMNSYQTIKKRII
KLILLFTGLWIVSSSFDMFAWVLSYGWRTPLVYSVAYIYLYIKIATMLDFTSHVIHAEVRLKI
IGDLVQVHYNDTECSTDLIGDCIFNKNWLYANENSSTSKRKLHPDPIKSLPHPGHHEVKSL
TRCYLMLTEQVVYYVNSMYGLRILLNSLSLLIDMVRYTNIGVRIVLKSQDTPYDSGYLPAVS
NLIRLLTCAAIFVNLADHCENVYRKRERIITIIDHLLIYKSPSKNLSTEASELRALITNRPVYF
NMANFVCINYSLLASIASIVTTYTMILLQSPN-
>MsexGR42
MPRKKTKTSKNLGLQEVLLNNRVDEDIQKLLFPMDLIQRLLLIPKYKIRDNFITPNGLWAN
IINIIFTCIVTMIFLHYTMYTKRIIDVTDVKYVQTCLWLDFITYSMCVIINCLTCIFQSKNFVH
LLLKIQESHRRLYSEKFQSFVKLNWVFCVILVIYQIKEAIIIHVLYLNGNMIVTAMTQYLLSS
IDFNILIAIRWMQFINLKFEVWLKEFRIYTNIKESSYGRLKSELMWKRMYHMYSCITKCYN
LFKKSFQYVV-
>MsexGR43
MFIKSKYLNQIHSLPEVSENNKVDEDIQSLLAPMRLIQNICLLPKYQMSKKIIKPNGFWLNF
TCVIGTSLTIAVLAFDYIVTDESLNHSTLNVLRKSLLFDMIYYAVGLIVCCSVCVFLSKWYV
LLILKIQEIHRKLYDTRFRCFVRRNWASVIFLVLCYMTFYFLAQLTFYEESGTFLARLIALVN
FDFNIVTVIRLMKFINMKFDIWIKKFKIYSSIGIIYGHNTNKILWTKMYHTYWCVMQSYSL
VKKLFQYLV-
>MS|comp134624_c0
GTASRGLAMNLHKALEAEHPALKVAQYRHLWVDLSHMMQQLGRAYSNMYGIYCMVIFF
TTTISLYGALSEILEHGLSYKEMGLFVIVGYCMTLLFIICNEAYHASRKVGLEFQVRLLNVN
LGAVDRSTQREVEMFLVAISKNPPIMNLDGFTNINRELFTANVSFMSTYLIVLMQFKLTLLR
QSARKTIKSVIKAVFNTSTTMLDDDISEDEEEA-
>MS|comp636901_c0
MRACGLFCVDVCLILHLAELLRTYSIVLLQFAFL-
>MS|comp19407_c0
CANELPKKLLMVELSWMNTDAQTEVNMFLRATEMNPSQISLGGFFNVNRTLFKSLLATM

VTYLVVLLQFQISIPDESQ-
>MS|comp121176_c0
AILFVIFLVPHFWIPFVGWGVAHQVAIYKTNWGKFQVRYYRVTGENLKFPNLKTTIVIISVG
CLLLAVCFLLSLCILMDGFLLKHTTA-
>MS|comp1194584_c0
MGVESAKVEEVTASPVPTESGTRPTRPTHCVVGGAHAFILKISSFFGLAPLRFESRSNGFTV
S-
>MS|comp82756_c0
QLLHTLAGGIKATQKCRPDSSEDHRLMGGYVHSIYFVFSFAFLVVRSLAVSLTAAKVHAAS
LEPAHSLYDVSSANYCVEVERFLAQIHGDTVALTGLQFFQVKRGLVLTIAGTIVTYELVLM
QFTGVSPASTEVS-
>MS|comp1687776_c0
IYSEERVSSNMVDKDVQSMLLPLNLMSHTMFCPKYRIKDNIISPNSLISKFVSMIAALLSIFT
F-
>MS|comp109140_c0
LSLHKVQILRTAEGKFEEAVIEYLFTVYLFPIIAVPILWYETRKIANVLNGWVDFEMVYNQL
SGRTLPVKLYKKALAIAVIIP-
>PxylGRP2a
MLLNFLIYASAFIHADRHVRVLNTVSAAWTELPFVPNSQITNSIRYNIKRRGFLGLAAVTAA
HILLISTEVESQDTVIVNTLSIFGVFIQYLMIVHFYCLIQLAISILQNITENAAHFMNLKKSVL
GNNDCWSEDWEGKSGTTLSRLEVNYSKAYVAVAEVNACFQLPALASTVQAFHAMVGEA
HALYHGVVVDHDVGPQKAIIIFCTVLSQVFKIYLLGHIGSILKDEASKIGPALHDIPTDKQD
LRLMLEIQHFSNQIQFEEMELTVYGYFPLDATLLYNIITAAMMYLIIFVQFDSDA-
>PxylGR22
MNDNRFRVYNPNQNEEIRKRDMMGKNGSTDLMGNGSYQAKDLHGPQITAADGYMLDE
HDSFYLTTKSLLVLFQIMGVMPIMRVSRHAKTTKRTTFNWISKATLWAYLVWSLECIVVVK
VGRERLETFQNSSNKRFDEVIYNIIFLSILIPHFLLPIASWRHGSQVAIFKNMWTHYQLKYL
KITGTPIVFPNLYSLTWGLCIFSWGLSFAVILSQHYLQDDFELWHSFAYYHIIAMLDGFCSL
WYINCNAFGTASKGLATNLHKALEADHPALKLAQYRHLWVDLSHMMQQLGRAYSNMY
GIYCMVIFFTTTISLYGALSEILEHGLSYKEMGLFVIVGYCMTLLFIICNEAYHATRKVGHEF
QVRLLNVNLGSIDRNTQREVEMFLVAIAKNPPIMNLDGFANINRELFTANISFMSTYLIVLM
QFKLTLLRQGARKMLKTIVMAVFNSTTTLADDDEEEEPDVGQ-
>PxylGR22-like
MIPDHMFEDGINNSLLRRDMRHIQQSKTVCEKSLRDYEQEQRDLLSSQDGDTCEIHDQFY
RDHKLLLVLFRALAVMPITRSSPGTITFSWRSSATIYAVCFYIAATAVVLTVGYERIMILRSIR
RFDDYIYAILFVVFLVPHFWIPFVGWGVAHQVAIYKTNWGKFQVRYYRVTGENLKFPNLR
TLIVVISVGCLLLAVCFLLSLCALLDGFLLRHTAAYYHIITMINMNCALWYINCKGVKIASQ
SLAECFRRDVDAECSAGLISRSRYLWLNLSELLQLLGNAYARTYSTYCLFMFANITIAIYGA
LSEIVDHGFGFSFKEVGLFVDAAYCSTLLFIFADCSHKSTLMVAAGVQETLLSIDVISVDRA
TQKEIDHFIQAIEMNPAVVSLKGYANINRELLTSAIAMIAIYLIVLLQFKISLPKDPQVN-
>PxylGR24-like
MSLYSSNAIFPKTEMPIPNGFPPHLNGNRNPKNKMIFLDVKPPRTPNMKSSAPTIRTLKNNL
TVPQINRDIIYENIKPVVTILRVLGVFPVSRPRPGVNLFHLASPSMLYSVIAFFSLVTYVLYLS
LHKVQILRTAEGKFEEAVIEYLFTVYLFPMTAVPILWYETRKIAGVLNGWVDFEVAYKSLS

GRVLPVKLYKKALIISIIIPLLSTASVIITHVTMVHFKLTQIIPYVFLEILTYILGGYWYLLCET
LSVCANILAEDFQQTLRHIGPAGKVAEYRALWLRLSKLARDTGIANCYTFTFVSLYLFLIIT
LSIYGLLSQISEGFGIKDIGLALTACCSIFLLFFICDEAHYASHNVRTHFQKKLLMVELSWM
NTDAQTEVNMFLRATEMNPSQISLGGFFTVNRHLFKSLLATMVTYLVVLLQFQISIPDDSQ
GQLMAPPEVVSNTAQSAAEPLSTTMMTILTTVVSKKKNNKSLV-
>PxylGR28b
MPKTKKSTPKKVITKIPEIISKQTVLVLQLIGISVFEVYYDKSGKLAKRVSYLGIFGSLFWLG
SFGFSAIHACVNDHTILRAYYDTKLKNYGDAYERITSLLYSETVILKVALQVSSCLPFTQYI
VDIDKTLASRGVKVDYQHKKTFFITTLQLIPVVLRALTICATLWAIDAVIPVDRIFQLTLTDG
VSLMATTFYCHYLYLLMDRYKHINEILSGVKEEATRKVRIDAATIILENDLNIENARRAEDF
CERIRLSSKVHSMLFKATATVNEVFGMILLVTSLLPLSYIILNMFYFMEATSAGLAHDVKRY
VNFLLYIAWAVLYNVLVIFMNVYFSESTVSEAKKTNIIVHDILNSEVSSQVKKEARQLSLQL
LHQTPAFTAWGLITLDYALILEVPTLTSITLLRFNCHCGLLVEALLHNS-
>PxylGR43a-like1
MTVQVQDHKKRPIQRQRGVRQGDVISQKLKTDALEDVFKTLDWNGHMSMSHLCYADDI
VNMAECLPELSWMLMLITYTGLLYDLSVGPSRGVRVQSNMSLVVWACDLSCIAAACLAG
AATAPARVKQMLVVIREVDSINAALNSDLNERGERKKKALCVLIVAALLLLFVLDFRHMT
EEAYNEGREWRISFFYLSFYCLYIIMFILELQFVLAVAHVNCALRAVNDALARELRDMEYIE
LKPPTSNAIHIRRLSQLHGAAAAAARALDRGWGPVLFLITANLVLHLVETPYYVITDMFQI
NGVEPTFLMIQLMWCFTHTINMYGVIEPCHTTVVEMSETRELVSQLLRRADSPSDALRRQ
LALFAEQLMLSRAEYAPLGVVTLHRPLIATVSL-
>PxylGR43a-like2
MQQLLVGGPMLMHFEDSKSMKITVIGIDPVKEGAAAVPRARSVEKGTCVVDGAHAFILR
VVSTCDVLVVAATAGAGVYGAPARMRSMLRYMESVASLDVSLGAQYSSVSERKLCAVLL
SVLFLFSVLIVDDFCFYARQASRFDRTWDVVTNYIGFYLLWFVVVILELQFAFTALSVRAR
FRAVNDALAHTASCVAVPLEKVKEAHHMNMFAIRVAPADSTRPTNVSLLVDSMSPQDHTV
IIRNAVSGEPRLAVPPCEAVRRLGALHGSLCEVIQRVDCSYALPLVVILISTLLHLIVTPYFLI
MEIIVSTNRVHFLVLQFLWCAMHMLRMFVVVEPCHYTITEGKRTEELVCRLMTHAPSTGV
LPSRLELFSRQLMLRSVQYSPLGMCILDRPLVASVIGAVTTYLVILIQFQRYDN-
>PxylGR64a-like
MNEIGKVFEGDKESGPHPQHNDKNKFKKQAHNSVRVDDVDFIRTMHSVFSAARCFGVPV
CGGGAARAWAALLLALHVCIEGGSVYKLVKNLAGLTAYSTGNRSVVARLSGAIFYGNALL
SLVVFWRLRAAWARLLRDWAGAERGGALPLPPDKALRRNLWLVVAVVAVCSCVEHGMS
MAANINLDYPPPIVFKEYILNSHAFLIIPSDYSLPLGIAIFVISKAATILWNFQDMVIILVSLGL
ASRYGRLNKYVHHLTKMESISGHTGSENDSCMDEYASLQSWRRVREAFVCQAALSCMDE
YASLQAWRRVREAFVRQAALVRRADAELGSLILLSNSNNFYFICLQVFNGAGGEDGMSLN
KVYYLTSVAWLCARACAVVLAAAEVNVRSRTALGFIYEYPDRSYNVEVQRLEKQLTKDV
VALSGKGFFYLNRGILLEVVSSIIKYELVLLEFDKK-
>PxylGR64e-like
MHAHHKTYDVIGYVFFYSSVFVNSIMFCRISVKWPALAAAVEETEEADPLKDPAVTSRCN
RAVVVIATLALTEHLLMWISKVARLSDCYPEPRRSYQTFIHSNYPFVFELGLPFSLPLAFAL
QYVTFITTMNWNYSDVFIICISYYLRARFHQLNNKINDAKGKYVGAWFWRRARSEYTRL
VELVHRVDDVISPVVFVSFASNLFFICFQLYYSIAIFFGYERETYLVFSVTLLLLRSVAVSLIA
AGVHTATLEPAAALYSVPSPVFCIEVQRFIDQVHGSIVALTGLRLFNVTRGLFLSVAGTVVT

YELVLFQFSAAADRHHGDPGDDNSTFFVPHMD-
>PxylGR64f-like1
MERVAGRAAARGASFQESLRVVLAIGQLFAVMPIYGVSSRHSEEVKFVKVSWKCVYTAL
MLSGQLFMTLTCVIEVFYADDVMQSITSTVFYGTACLTMVAFAQTARRWPALLRFIGRVEE
VDPASDGRLVWRCNVTCVIILSMSFLEHLLSILSDVYWANACSPSPVDTQEEFYRLLYPWI
FRSLPYNPLLAVMCQIFHIQATFTWNFSDLFVICISYYLIRRLQLVNKKLHAICGKKYGESF
WRSTREQYGRATQLVRRVDRVISGVVFISFANNLFFICLQLFHTLETGKRGKSVCLHPVPE
VQPVAGFITTLYFSFSLVYLLTRSVSMSLIAAQVHTASTVPAPVLYNVSSSEYCEEVQRFVS
QVNGEKVALSGLQFFSVTRGLLLTVAGTIVTYELVMFQINSPGSRNYVTPGPPTVTNISSVF
ST-
>PxylGR64f-like2
MGQKNKKNVSWIPTKKNRVSVPAVKPAAAVATLQAALRTTLLVGQAFSLIPVQGVAASTA
KNVKFEKKSWKCVYSFLSLLGQIFITVMCFYKIVYSDSSLKATTSVIFYGTTCITMVMFYQ
VARVWPRLAHFVARTEQLDPSLDQRLARRCNATCAVVLTLALMEHILSLLQAFAGAAHCF
PDAPVYEGFVRHFYPWVFNFLPYSPYLGIMTQFLHFQSTFIWNFSDLFVICMSYYLTSRLD
LVNQKLLNAQGKYLPELFWRSTREEYGRATQLVRKVDEAISGVVFMSFANNLFFVCFQLF
NTLENGFKGRVECSGTTSSSTTGAGPGKGYEAAAYFLFSLVYLISRSVAVSLVAAQVHSAS
AVPAPVLYDVPSPVYCIEVQRFLDQVNGDHVALSGLQFFSVTRGLLLTVAGTIVTYELVMV
QFNAPSSASPASATAATLLNLNESSTLPMP-
>PxylGR64f-like3
MFERLRRKSTQIVMAKNVVLEKKLQILSDEGGVQGFHRALRLTMLAPRLLGMLPLCGLT
CPDSSQLRFRLISPYLPIFIFALFGQLFMITCNLYRMFDIGCSLGTITNCLFYASGCFSTIFMIR
IGQRWPDTVQFIEAVERRLPPLPSQVTRQCNGIMIFVLTAALVEHVLCDVYLYKEASACHD
GDIWRKYFTDNIPWIYNYIPYSPWKAVVTEMFNIHSTFMWSFNDALVMVCSIYLTHHFLN
HNRLLENVMGQETFHLKEFRIQYKNMMNLVNIINKEIGVVIVISFFCNLYWICMQLFNSLN
KSDNSDNDFPSVECQQRSIANKSSLEHNMYFAFSFTFLFVKALLVSFLGARIHSNSLVPLSM
LFEVSSSNYDIEVQRFIDQVKHSHVAISGLDFFHVTRQMILTLVATIVTYELVLLQLNIDSIIT
ADGNATTTTVKPLSV-
>PxylGR64f-like4
MLVFSLNYKNDKYAFTYSVYALAWLSVRCFSLLVAASDVHARSRAGADYFKNIQTTSYN
TEVRRLQFQLSKNEIALSGMGFFKLKRSLILKVLSVIITYDLILLQYNDNL-
>PxylGR68a-like
MVKNTFNNSTNSDLGEIFKPMYILLSIVGLFPYSITFNKSKKQFTTQPNSLYFNLLPAVTGAT
TSCVFFCLHLHHVSQAGLEGNLSEAVVTTTNYCTELVLSLLCCVAPYCCSVLRRRAPAAM
LDRVAAAWAAAPFHLASTILPQLQHNINISVAAVVFHLIVHTVIIGALNTDLWKKVLLIFSF
NFPHFIQCVFVAYLNVMALLIVSVLQNIRETVAVHAKESAALGLRKAMSAPLNVRHLEET
YSEAHRALAELNRCYQGPILFTLLQCFHSILSNSHIIYYGIMMQKDFNVLDGIDCGVWIVW
QILKIYVLGRAGSLLNIESRRIGETLHDLPVDKLESRTLLEIQHFSTQIRFKKMILTVYGYFP
VDSTLLFNMVTAAMMYLLILVQFDIPENNPSQELH-
>TcasGRa
MCQIKNKLLLLDSSTQPNYLILSNKNNTYLVLNKYLLVKHSKPKMPLLPKTPLPLTLLYKL
LGIIQFPISAHFSFMSRFLCLPFYSYFFYLSYIYTTYSRKLSGIFKYIDQMAGYTGFLAMLTS
MVMFYKRSNDLKTLLSNLESIQIYSIKPKERNSNHWVRSGLFALITGNVLFYPFLPSDVSY
NLFSFVPLVVNALDHLFLNDILSDICDKFEQINQHFRRQIKSVDLFVIFPLTKAEKVRNLKE

DEVTFSVQKIQELSHLHYKLANFTVKISGLFEITTITAMVMWFGYVIDTMYLFIHIRSRQED
TDTLVVIYTFNLFYLCFCFYWLLVMVAMFSRTQQSANKTATFVHEIWNKYALKNEVDKRV
RHLQLVAIRLLNTKLQFTAKDFFNLDWTFCHMVSHKWLNYINQFEANFR-
>TcasGRb
MHTLQHFTIDTRSTHSMKNFKYLKVLVTFAHFICLFPITINIRKNGLNYNFTKKCYFLRMV
LIDLLIVGCILKHIFNILMKNVTLNDVVFIFCSAPIIITILDIEILGYVNKAKFGKLLINLYTINY
NFKESERNDYVSLLQLVFVFCYFIYLFTVYYIYYSVHEASIINFGYTLAKFMIFTSTCIYTNL
LRIIEADFSKLNHLLSGTENLDLVLPIYSQLVFMCKKINKLYGHQLLLTILTYLIWTIYEMYH
LAILWSCTSTNCPRFLIMLALSYTVIQEVMLFTILWNCQNTRVASEDFKAIWYSILIKKADS
FCKKKLENYSLQLINHRVVFTAMGLYVLNMEHFFSVKCSIQSESLLNDILFPVAGFISDFDC
DINSIQYFCLMARCLNTLIFYYHISTYEYSI-
>TcasGRc
MSEREVILKAALTSRFFLITIQYVSNLLIPDHEADAYTYPKNEDNGIFDKLVTHLLGGFVRW
DAHHFMHIAIFGYTYEHTLAFFPLFPYSAKPVVAILSYLLPFLSTDSLTLITLITVNIFCFAQS
ALCLYQLSALIMNKDLALKAAILFCCNPASVFFTAPYSESLFCYLTFKSMLNSVLLYKKYK
NQGYLLSDVAYIIPICLSTCTRSNGVLNIGFLAYALICLFLEKIKLEKQICNLLLCLAKFITLA
AVLVLICLVPFICFQFYGYQTFCKNFKSYQVPLVLGHKNIDNFVLPGTFSQHNQSWCYKK
MPLAYSYIQSHYWKVGFLQYYELKQIPNFLLASPIILIILGHSLHFLKEFPKSISKLFNFDLIS
VKSVRTKKFFPVMAVFIVHASVLTLFCVFNIHVQVTTRMLCSASPVIYWFCSYYVTDVNL
FKNLIARKCNWGELLVLSYFLGYYFVGTVMFCNFLPWT-
>TcasGR1
MRNDHGSNTHLHPDDAIRRAKIVKVAASPTSANPDEEPDPELLDRYDNFYQTTKSLLVLF
QIMGVMPIERSGKGRTTFRWLSSTSIYAYFIFGAETIFVTMVFKERLYLILRPGKRFDEYIYG
IIFLSILIPHFLLPVAAWTNGTEVAKFKNMWTRFQLKYYQVTGTPIIFHNLTLITYSLCVISW
AVGIGIMLAQYYLQADMLLWHTFGYYHILAMLNCLCSLWFINCTAKGRVAVWMCNNLH
KALESRNPAKILGAYRDLWVDLSHMMQQLGKAYSGMYSMYCLLILLTTIVASYGSVTEIM
DQGISFKEAGLFMIAFYCMTLLYIICNEGHHATRKMGPEFRERLLNVNLSAVDQKTRQEV
HMFLMAIEKNPPIMNLNGYANVNRKLISSNLNERNCFSNIVKLRTLSPTQT-
>TcasGR2
MEISDLAQLYGNELHIKQISKWLRGSARAQEIQKRSELDSKDGHVIDEHDQFFRDHKLLLV
LFRVLGVMPIQRGEIGRITFGWTSIPMLYAYVFYVVTTVLVVLVGYERFDILLNKSKKFDEY
IYSIIFIIYLIPHFFIPFVGWGVAYEVCDYKNSWGGFQLHYYKITGKNLQFPLLSTLIIIISLGC
LILAVVFLLTLSALLEGFTLYHTTAYLHIITMINMNCALWYINCRAVGNASTALAESFQNDV
DRNCSAYIIAHYRVLWLSLSDLLQKMGNAYARTYSTYSLFMMANITVAVYGFTSEIVDHGI
RFSFKEIGLLVDSTYCLFLLFVFCDCSHQASLNIARRVQVTLLQVNLSQVDPATRKEIDIFLV
AIQMNPPKVSLKGYTVVNRELVTASVATIAIYLIVLLQFKISLLNMRG-
>TcasGR3
MPKTHRSIPKSALNSNAAGWGCLPSSSAFSAGMKSGCGSAQSMGKRLDTHLGLVRALSR
RWPTKNTDTTHAIQFSSALSWPQADASHPRPGCVTSRLSRTLLLLLLGSAQRRRQRRVWT
TPRRPKAAGEELGVVSTINSSTMYHQDQAVSILGEAIPKRRSVFLESGVNSADSFKASKVG
PAPPIKFINKSSTDKFGNGAIYEVLKPIYALMRIVGIFPIKNTEPGMFRVAPELLGYSVVVFV
VVMGYIGFIEWDKVEIVRSQEGRFEEAVIDYLFTVYLLPIIINPLVLYEARKLANVVTDWV
NFERIYYKLTKKKLSVFFGNKPVILTVVLPLLACGVMVVTHITMAHFKIIQVVPYCYINCLI
YLIGGFWFMQCDVVGKVASQLAEDFQMALKHVGPSSQVADYRSLWMLLSKLIRDVGNA

SGYTVTFLCLYLFLIITLTIYGLLSQLQAGFSTKDIGLTINAGLAIFILYFICDEAHYASNCLRV
QFQKKLLLVELSWMNDEAQQEINMFLKATEMSPTDISLVGFFDVNRNLFKSLLATMVTYL
VVLLQFQISIPEEASPTNSTTITTQTPN-
>TcasGR5
MSLKLVNLVFKIGSLLALTPAKIEKNGLVFPTKAYSLLWAVLFSGALSITAIFRKASYEKLSP
VVLFIQVAADTVLFILNISTIIITARKKQQWNSLIKILKTVSNRNDKGDIFWFSPFLVANLAF
VTIVTYETFVWTQIMGAEFFKLYAVEYFQMYAQFIVYYLIYVFLNSILEGFQHLSKTMCKY
LKLPNRSNNFSLKKIRSEFCALAIFVDVFNDIFGWLILQSIGFTFLQLLSYMQHLIVGTGHTI
PTLIYRLSFITWYMVGTFNSVFICDLIEQKVKNIQMLVYQNEAEEVKILLDVINHFPHFTAA
RFFDLNRKTILGVLNALFTFLIVVVQFENLTS-
>TcasGR5a-trehalose-like
MTEFWLEMRRDYDRLSHLCKELDDGISGLILMSFAYNLFEVISYLFHQLMMDSQQNVAFY
FFFPYMVLRLLAVCLYTSWINDESLAPVNILNSVPSRNYNPEIGRWLVQMSFDNVALTGW
KMFKVTRGIFLGVASIVVTYELVIMQFYGFSGKT-
>TcasGR12
MKKNSKYVQNCIFYAMKRPLLVAQIFGYFPLYGTNSDPTCLKFKWISFKTTYSVFTLFVTF
FIAVCQLHKMIAVEMNILQMNYFVFLLCSILVNIAFIKLATEWPQLMKAWLKIELLVGNLG
MRRNFRKKLDFIFTFITLLTIVEHLLMELSRAIDSVACSKTVSDGIRHYYVNVTFPHLFNGL
VDYSLWKALIFQISNLQTTFGGTFGDTFIILLSMAFATRMKQSRTKIEALVKSHVKATTPWR
KIREEQCSLLYLCTLLEQKISYLVLLSFCSNLYFVLVQLFSALKQMGDTLQKTYFFISFGILIF
RIIFVSLSAASINEESRKILILLLSTPSELYSVEVERLTNQINYKAMAISGKNFFIITRGLILKIA
GAVVTYELVLIQFNKKLLNEFDETSVQQLEILHNNTYWLC-
>TcasGR26
MANCATALTLVCWGFWGFVHDMEIASFVSLGFTGSVDVVISSFDISDVLLSCLYFIVSMPF
KCAKLSIVFHNLNKVDAIITPVFCDRFYSNLVWFSRCWFVFLPVLYTLDVFMWGNTSWLG
VNNYFAYYVSYSIVVLHELQYYQVVKMAQLRVSGINKTVKENIKKDTSRIKLEFIFDLIHC
YNNTTDAIETINSSFNKTVTLMLFSCYVHLVTCPYQLFVMITSNETSILNYVYCLWVLLQIF
RLVLVVEVCHNCEEEIQNTRILVSQLLNCRLDKNVKKEANTFLFLMVKKKIKFSAYGLPK
VGRHLLLSVASSIGGYWMILLQFSSRTSKI-
>TcasGR27
MQVASFTALGFRGTADFVIACFDVNDVIVSAIFFVTSTPFKFKHFVQIVENFDRIDARISPIL
VEQIRKRSNIFVKVLVTFLPTLYVLDLFMWGKNNWEGLNNYFAFYIMYSIVVVHELQYW
HIMTMMYARILGLNKTLRDYFKNKTGFCEHEILVVTQSFNSINDSVEEINKCFSYSTTTIIFS
CYIHLVISPYQLFVVVSSTETSLFNYVYLLWISLHIMRVLTIVEVCQKCENENRKTRSLVYQ
LLLCKLNEKVKNMVRVLFFLVTTRKILFSAYALPKINRRLIISILSSISTYWMILMQSTSRTIQ
VV-
>TcasGR-SG43a
MTITISKELFHVLSPVLYLSRFFCLQPLKWTKTSAGNYIITKSRFYTIYTLAASCLLVITSITG
LSQVYQLDVIYLVRLGDTTRRFVTYSDIVVVLLPCVIGPVFALFKTNQTINYLSHLKQFDSL
QNQPTKSTKIFQITALTTFCTAFTLSMDLFLWLKLSHNYIFLLCLPYYISYWSTVVIELLFW
HFVHLIQIRISVINKKLAKMVVTGLNSVTTLKKPHAEVEDLVKGYEKLIEATNSINYCYGF
PILVIILGCLIHLLVTPYGLYSIIMSTGDSTSILSQTVWMTAHILRLFLIIEPCHECFIKTKETSQ
LICKLLCLSVNQEVKKSLEFFLTYLGECKIEFSVYGFTKINRELLTTIAGAITTYLVILFQFK-
>TcasGR-SG64b

MNILQMNYFVFLLCSILVNIAFIKLATEWPQLMKAWLKIELLVGNLGMRRNFRKKLDFIFT
FITLLTIVEHLLMELSRAIDSVACSKTVSDGIRHYYVNVTFPHLFNGLVDYSLWKALIFQISN
LQTTFGGTFGDTFIILLSMAFATRMKQSRTKIEALVKSHVKATTPWRKIREEQCSLLYLCTL
LEQKISYLVLLSFCSNLYFVLVQLFSALKQMGDTLQKTYFFISFGILIFRIIFVSLSAASINEES
RKILILLLSTPSELYSVEVERLTNQINYKAMAISGKNFFIITRGLILKVSNKH-
>TcasGR-SG64e
MTTTTHSSLRFILIAAQIFGMFPVSGVAKKDPTFLKFKWTSKRTIYSIIFALAAVVNTIIFLVH
RVSLGRLKFADWVTLVFFSVTFLIVVLLLQIAKHWPSLMKKWTQVDEAMSGYGFPPKLE
RKLRIIFAITVVASLVEHGLFIGVEYMSCRGNNLSEALDRFLMFHYDYVFALVPYHVVLGII
LEIVNIFSTISWTFMDLFIILVSLSLSARFKQVAKYIKFLVERNVLNKNSWQRARQDYTRLT
NLCKDLDEVMSSTILLSFGNNIFIILVHLYNSLQKPLEFGYLDEIYYLYSFICLLVRISAVALH
AATINTESKRPIYLLSTIPHHRYNLEIDRLLLYTKYETAALTGYKLFRITRTLILKITLAIVIYE
LVLVEYLKVESY-
>TcasGR-SG64f
MKNIISHNTENTIHSSLKFSLKILHIFGLFPVSGLSGPDYKSLKFSWRSFKFLYSLCFFCILCL
FVLTLLYNVFFVKEATQEITNVLFYLSAAATNAVFLQLAKNWSRFIHEWHCVEVIMGSVAI
NHSLKKRLKIITIVILVVATVEHLLIQCYIAVSIFGSSSFEADLRQFYKTAYSAIFTVIDFSLCK
AILVHAITIRSTFSWTFIDVFIMLTSTAFVFRLKQLNAKVEMLKNARVKNTALWKQLRYEH
YRLYQLSVLIDNNMSYIIIVSFATNLYFIIIQLFGSMKIVKGTLKTAYYLISFALLIMRLISVCL
CGASVHSESSKVLPLLFSVSSSSYNCEVERFIDQVIKNEIILTGKKFFKITKQLILQIAGAIVT
YELVVIQFNLRLTEDNDSNEANLLA-
>TcasGR82
MPTRGTISPFHRFVIHLCNVFSEILSCTQILFFMVLTSFMIHHMLRVNIAIAIVEMVVKPNTT
TPTEENFGPKYAWNEREKNDLFAWFFWGFLITQIPGGRFSEIVGSRIVLGLGILVASVATLLL
PLCCNVHYYLVVASRFCVGLGLGVHWPAIPPIAIRWSSSATARTMFMTHLFAGSLGAAIVL
PVSGHLIAYVGWPSVFYVTGGMGVLWSVMWFYLIYDSPGQHPRISAKEKEILEQKIRNEIT
PQVRHIPWIKIFTSLPVWAIVVANASICFGFYIIFNHLPTYMSSVHNVEIEKNGWISSLPHLG
EFFREKFVISAYFISGRYITTVAVSYLGARMLHKNKFSTLTIRKFLSVVCSWSAVLLFGMEA
LFGYHYYVTNFVSITFFLFLSLSIPGMIVNILDISPAFSGTIIGFNQVIVCLSGITSAKVVAFFT
ATKQSFEQWRYVFIIVAIVNFVGGLFFLIFASADVQSWNPKENVSQKKNTLLNDTTSKDHE
EF-
>TcasGR87
MKEVPGWRNMKPLYYLCNFLAVTPPHSLGNTVTPVSLRFKLYTIVHIFIIIALYAHSSYGRE
NFIYGSMNMTVAITDKIANFMLTFFNVSLRIILVFSKGKVIKSFFNQGYELSKQEIFNCCSKK
SFRMNFAIFNLYMVLLLLFDAYLWISSVGVRMFQYYIGRSFTYYVCNTTIFLIFHSVLPIKR
FFTSLGIAFDNIMKNLICEIDGRHEFFLAEFKSAKTPPNKLNCYDLRRVRKNYNSICELVDA
FNNIYGLAMLEIIIVVITYVLNLTDLFLVYGMSKSRNIEGVSFGTNLVILCALWITTLLLFTIL
LAYGCAGATSEAEKIAKICFYWLNEIPTMPVSIKDQTIKEELALLAQQSTSRTSKFSAAGFF
PVDFTLLGFIFGSVTSYIIISIQFIE-
>TcasGR93a-bitter-like
MVTKMVCQFSRNNDLLRNSVSKPLFWYLIVQVAITGNKILGAVLTISPDAEVYLNVLFYNF
AELFSYNINILIFFYLIIFQNAYTSYFKAITRKNTYNDPKIVVQIFYKFYEISNNINETLQVPIL
LRIFTDFVFTISSMFYLSVFLYIIPELSFFVLAKMVLWLVNTLFAIFIMAYFFDQMTEKRNTI
METLEDLPSSHTLISKFHSSYKRKELLRLRLKHERFCFNVCGFFPLNNSFIYLMIAGIVTYT

TYLVQFGKAANAKQ-
>TcasGR97
MKNHFSKLMKNRRGRKTSKMMNGMTKQIQDVMPGGNRLKISLTLFAELTMSSQSLHSTL
ETLCFLNQLIGAPFSQTRHKLYPTYCKILLFTHLTTLLFTFCNIWWRCSNSQRISTKLIKTLN
YFFKVTIIMSTILNSMILKKKNVAQLIPTLKEAKVMLPDVKQKWSCFTHFDSIFVLFSIMTS
FIFHLTYKSGCYLIQDLLDDVEMVQSLIVLLTSLKFLELLSNYFDQTNNYFDIILKSEIFISD
MKMRRVNQLYRTLYRILELWNNIYGVFLLQLFLHIMVVLIDNLLSLVEEFKLGYSFSWKV
VIRSQTIAIYLVLALLLSKIGYQISEKKYKTSKTVLNGLVEITLRGGKQSQLNLLLLKLKTW
PNFVSASGYFRLDYGFFLSLLAAVVSYIVILLQLS-
>TcasGR117
MCLAIILMNNKLNMVLVKSMLQRIKMEEKLVVEKFLNSLQIYLQHNQIFGFVTFTCTRSNF
RSSKLLILYNIILQVLFVSFVSYWLYLVLEADDMLPIYKNTYLIILFADFAYLETTWICTLLK
KDKLLELFKRLIHFDTKCQENSTVIDYKRHKKRLLCYLLARYVALALVILFSEILVIVSEQE
WSFSTGLLVMIFNSALSYKASEIVVMLRSRFAILNKQIRFLNQYLRLKPEGRISNRRVFISFS
KICYLHQHLSKSVKLFNEVFGVSLLVLFGNSFLSIVLALFRTAAELQASQIKWTRIAYMAL
ASVPFIFDSIHLCDVCYSTIGTVSKAGELIHQIQTEDHDIIDEIEMFSLQIANEQVEFNAAGFF
PINYTLVFSIIGGATTYIIILIQLSATLNE-
>TcasGR118
MRNVPQSYCFVTILFDSVNKLGTQIVNPYSVKQYVMQQQVKNFRHSVRSVFFLSEIFGLV
NLKYRETYFRLSKTKTFCTLVTALVYCSLAIFVLCELLIEGTTSILINVPSLIIHVSTSAYVAT
VWINSVINRWKFIEFIRKVLEFDVKCVSNYTKQQSKIHLIVRSVFVTTYLMFDYCTVLRVQ
RFNNYQSLAHYLRVFFTVFNVVHCYLASELVLMLKNRFVTLNVQLTKLTKNCATKAQSV
VLGRICTLHHHLSKLVTRFNEIFGLGLLLMFGVSFLLITQTIFIICVIVQSEQIAWLHLLYIFLV
GIMYAADVFYICHVCCSTIHEVSKAGELIHKIETNDHEIIDKIEMFSLQILNERAGFSAAGFF
PIDYSLVFSFLGGVTTYIIILLQLSSSVPV-
>TcasGR125
MKKLNYTVEISYDLEEGVKTAHCTCPRGNVACHHMAAALYYAHYNVSATDIECQWSAPS
KTTPQTEVIKLADVYKPKLSNYTALSRSSTEDEIIQFRAEIGVTNVVGFTWLLRPEASEEAR
KIIADIEEILQSLEYVQAIDKQKFLLEKCRIDEARIKLVEACTRGQHVNENWHVARKHRLTA
SRFGMVLSACSRRRFPPSLFKNLAEGYSLDRVAAVQWGKTHEKTALREFEEATNLKVQET
GFWLEESGFLGASPDGLVEEDGILEIKCPYKYRDTDSLSEALKDKKYFYWRDENEDINLN
SNHNYYHQVQGQMHITGRINVKSSSNMIKFSKVKSYFTTHHDTLHNIYTSMKPVYAICKL
IGLNTLRIGRKGELKQHKSDYFYFSFYITSYTLLSVYSLFRIATNENNSLVINKRLIFIECFVM
MALTLIVTLFTFLARGTLIKSFDMLSHVDVSFIKAGFRLEYKQLLKRSYLIISFVLFSLLARV
PVMLMTISADFIQQIMLFVSALIKAFSKYQFVVLVLQLQHRFGKINRTMRSFFSDNKQDDK
IPQISDNLYILCRLHYKLTSVMQKINSAFSVQLLVSIGVSLFDVLFQAYYLYYVATGKASFV
TVPMIVCPIVWLMDEVVEIYLLVYACASTCEQANDTPSILHELRNNYFHMDLENNVQSYS
LQLLHQKVQFSVLGFFVVDYTLLYSIVGAVTTYLVIFIQFDQSSNSRNNYVLTNNSTC
>TcasGR153
MTIITRSNNVVGGKFYKKRNKVSNFGLTFEFIFGIIILYTTYSMSFVKRNHVRDVVLQLNRI
DELLAKMKQKFRYTRAVWYQLIIFFSGFLMILIIASMQIQNIRIENFPPLSLFMCIIFLFPLVIL
YDLNSQYGFTAILIYERCFIKCKTTQKHTFRYFKQNNDFDGNPRNLMQGGKHHKLKLQHS
DATSHNARIFHQSLLNFLLLLDGNVTVVDTILNLQIFQHSSKPFSASCWLAWGFLKSYEILH
VTISCHLASQQANIVGRKVHKVLIRTQNDEIEEKLLMFSKQVQHSSFKFTLCGLFNIDAGL

LFNMIGSSTTFIVIMIQFQETITPTICVSNKKAMF-
>TcasGR154
MSTQDIYDAVYPLLLTTASFGLSAIFVETKNNTRKLAVFNLLKILNIIYLSLFSALLYVAFTSL
KNTHLHVNYNSGVTKIGIIFQILANIMATYVIYFINISKSHNILTCIENIKKADLMFRDLGEKI
IYRKHFLYEVCMILFGVLAILGRSIVTHIYMGRNIFLTENETHFALFFPLFVSYLVQINFVLLI
TLVHERFALINRLLDNFNEEKVKHNYLSLKPPYEYQTKYNQAKIELLMELHDFLTDVGNK
LNDSFSIQILSCLTSQFLTEVFTIFYLYYESLILKNKIAALVWLMWSIWTTLEIFYVTVNCHL
TTKEAKNTGIAIHKVLMNESDPDAKRKLMVFSQQVNHRSLQFTACGLFYIDATLIFTIVGA
AATYLLIMLQFQEGIEAQCSNNTLLN-
>TcasGR164
MSAAHDIYSVVKPLLIFSKVFGLYPNTVENFETQQLTSPWSNGLDIVWSATIMIFLTFYSIYT
MRMGEKPATSTELIVEIGDIIYMSVSLIDAFLLSLFSITKRTKMVQFLQKFANFEKKMQQLD
LPLDLANEYTRAKVCVLFAIATAISLIISYVIIDIILFLGNVTFEDIGAEIVAYVYPLVTMMTM
VMQFCTLCLLTRQKFRWINLKLDQIRKQWQGKNKTTYYSNKIKFVSPKETTKAITLEKLIY
FLEQFRRRHYELCSLTENLNRIFNVQILFTCLNLFITITFTVYFYFISNSDAKIRSPVLYSTYY
ALNGFINVSALFGLVWAASQTKNEVKSEPKKTLSLKTNIERRSGKLGADVKSPSQFKQFQF
PDNAFLVPTFAKQCRIFRLSCVVGAITTYLVIVIQFQYQN-
>TcasGR166
MLPKQFLKLFKDPCDVYTAIHPLFYVCTFFGLAPYSLVRVENGKKVFKFAWWPLTRNALL
VLILLGALTYHAIFDLISFKDSDLQQKLRYFEEVFSSLLSCCSVIFGCIFALKVIEVFKNIEEV
DVAFRSLAVWVPYKHLYVNILIHLSGLVTIVATLTVTIIFFASYQYGTKTYSLFIVFMTVILPY
FINLLMELQYCHYLNILRVRYQLLNEYLETLVQETNRTSVEGWTDVSNVKRKSKEISKLPK
SMLAISDPVFIVDQVAALHIKLTDTAHMINYAFCVQQLLRITVAFISIVTALFLVAINFNKSSS
EENEGKTTQLDYFFTFWAFSNACEVMAIVWITSETCEEANTCPRILHKIRNNTTNTNLQDT
IEIYSLQMYHNRLYFTVCGLFPLDYTLLYTIVAGVTTYLVILIQFNNSDFVQRNSTEFDNATE
SY-
>TcasGR168
MPLLDEIPFYWKYIYKIIGIIQFPTEENASNIVPHLWPLPQNCLFIYICVKYYVSINHLKFLGI
FYFVDALTSIGTALSLVISITIFIQRSRDLKKLLLQLKEIKIDSVHRKTRSKANHYLRIILIITIF
YYFLFIPFESEPLFYTLFFIYPGIILLFDHIFLSDILEIICNEFEQINREIKYQTTSHRIIFKTKKIN
DEEINDKMVRAEQLSLCHCDLTALTLNICHHFETTIIVAMITSFNYVTSTVYFLIYFLARSYE
ANLFVLFGNNFAFLSFYVFWLLLILEMFTRTEKEANITGRYIHDIWNKFEANGNMTKKLR
HLQLVSIRFLNNKLEFRARNFFRLDWSFCHTMIAAITTYVIILVQFHI-

6.IRs

>BmorIR7d.1
FNLPLKLTILSLFLGIILLNMLRKTIFFNNIRRVCNITPPKRNSLFYAWLLFLGLPLEKFSSRK
HFKIIILAWIWFSFVIRCAYQVTLVTSLKSITYNYNLRYDSDILKYPFGGMSSIRDYFIEDKD
FYENWTSVDMQKAYKLLDEIMEEKTDFVLALNKDTILHHAAEHIGSKRIQVIDNCIVNSPI
VLYFRKHSPMTDPIAKIMNAALECGFIQYSYQTNWKRQKHLLNSHYAYNLQPLTLDNFSG
CFFLLIIGYGISILYFVLEVVCHKIDKTNQRIDLRVDQE-

>BmorIR7d.2
MSPRNLSDASHFCEENSNEITTAALNIALHNFKWRILTYVFFNATFLCNLNIFLKTYNKGV
VVGNGLVEPRIDGKIQQLVLFCDDIVGITLALNSLPNQFDETGKVIVICQSPISWKCSAEEA
MRSFWSVKITNVVFLKKDVFVMAYTYMPVYNEQCEISDPIPLFGLKPCIINATKCGVFDKK
LDNLNKCKIVVSTLIRRPFMIINNGIPEGADGDLLLLIMERLNATLEVIIPGDHNYWGKLDS
NGTWSGSLGDVYYGAADISMTSAALTASIISYFKISIPYRSTNVVWISHPPKALSPALKLLH
PFKPSTQIALGIIFFIVIACVLFVSSKKMWLLCCRRVRPTKKKPSLLFNTWMICIGVPIAHLP
STSTFLSLIVLWIWYCFLIRTFYQVWLINSLQGKFYLDGFEKIDEAIEAGYDIGGGIFLKEYF
VDYPYIYNNWKETVSLNVTLHEISEGSNFIAATIYDLAKSLTNFEKINVHFLAEKVVVSPSV
LFFNKNSPLVAPINELLQQLTESGFVEKISRNYFTHNVTNWKRQKHLLNSHYAY-
>BmorIR7d.3
MRTEPEDITLFLQHFHGSAVIVPLDYQNMKAVSELNKATGFKQTVLFAVSVEEFILFITTLN
LDLIVPIRMVLVLTTQLTDLAMITKEAWKHDLAEIIIISKDENEEIRLTTYFPYKNGICGDYT
PHSISNEKELFPEKFKNLHGCPIKVTLLNFLPYVGLQKVNGTITFIFGIDGSVFILLIKELNAI
MDIVSSTDHGGMGVFVNGSWKGSFGDIVRREADIFAPAGIITQKRFSVAQMSHTYETLNIH
WCAPPRREIYAWAKVLLPFLTNITPFLVLAFTVFVITIVLVKRSKLHGIKSNKNVFLQSFMIF
LGQGVKFETKSSVINSFFVAWLWFCLIVRIAYQGDLVNGLQKKIYEPPFESVEQALQELDG
YGGTELFREYYAGSPIADNYQVIKIGDLPRYIRDVIAGKRFLIATDILMHQYAKKFQILQEP
LTHSPTCLFMRPGWPVSRRVDVIIIRAIEAGLVQKIIYDFHYTVRLRRHEKEEETGTRPLGM
STMFACYYGLILLWIFSFVIFLFEVLYYNWKHKIAYIKRKRNKLFKFHH-
>BmorIR8a
AVPWTLPKLDPETGDPLYNEDGQPIYEGYCVDLIQKLSEAMNFDYEIVSPRSGGFGRRLPN
GSWDGVVGDLTTGETDIAVAALTMTAEREEVIDFVAPYFEQTGILIAIRKPIRKTSLFKFMT
VLRTEVWLSIVAALVLTGFMIWLLEKYSPYSAKNNPGAYPYPCRDFTLKESFWFALTSFTP
QGGGEAPKALSGRTLVAAYWLFVVLMLATFTANLAAFLTVERMQTPVSSLEQLARQSRIN
YTVVEGSSTHQYFINMKFAEDTLYRVWKEITLNATSDQAQYRVWDYPIREQYGHILLAIN
ASGPVADAETGFKQVNDHTDADFAFIHDSAEIKYEVTRNCNLTEVGELFAEQPYAIAVQQG
SRLQEDISRALLELQKERFLEQLTSKYWNETLRQSCSDADESEGITLESLGGVFIATLFGLG
LAMITLAWEVFYYKRKEKNKVQSTKENVERPPIKSAKLGGKMAVGVARLRKRATKIGKK
KNVTIGDSFKPSVSYISVYPKGDYR-
>BmorIR21a
MDRRSLYGLLFIFYIISSQEIISYHSESLLKNASRNLLWNKKITSIIKEHNDFAYHYESDLHFG
NRIKNVKSKRAVDPVFHGHPKTREELWYERFLNRSSVFDQTPSLIKLIQNITLTYLNECTPV
ILYDSQIKLKESYLFQNLLRNFPVSFVHGYINEHSQLQEPKLLQPVRECLHFIIFLSDVKVSA
KVLGKQSESKVVVVARSSQWAVHEFLSSSFSRGFINLVVIGQSFKEDDDSTIESPYILYTHK
LYTDGLGASKPVVLNSWSHGKFSRNVNLFPPKMTGGYAGHRVVVAAANQPPFVFRRIFY
KKIYRIKSDLDGGNPRVVWDGIEIRLLHLLAEKNNFSIEIVEPQELHLGSGDAVAKEIAKGR
ADIGVAGMYLTIDRTREMDVTFAHSQDCAVFITLMSTALPRYQAILGPFHWHVWVALTLT
YLFGMFPLAFSDKHTLRHLINNSGEIENMFWYVFGTFTNCFTFLGRNSWSKTDKITTRLLI
EIGKIFPCYYFRMVLDFTIIITSCYTGSIIAFVTLPMFPETVDTIHQLLAGFYRVGTLDRGGW
ERWFLNSSDPNTNKLLKKLELVPNVEAGIMNTTKAFFWPYAFLGSKAELEYIVQSNFTKT
TSKRAVLHISNECFVPFGVTIGFPNNSLYTAKLNNDLRRMVQSGIVDKIVDEVRWEMQRSS
NGKLLSAVGGSLKVSAAEEKGLTLEDTQGMFLLLAAGFLIAATALISEWIGGFSKLCRFRK
KKNTLVNSSTKEDSINMPPTDSKDFKTETESVLHFCSRSTSPGSNESLDGQIINVTEESIEIH

KQFTSEWDSRRSSSVDLEKEVKEIFERDLRRRGAALXXXXSTASNNAFGDAVK-
>BmorIR25a
MCRSSHIFQNSMPLFVVFLQFFIFRLIVSQTTQNINVLLINEENNALAEKSFEIAKEYVRRNP
SLGLAIEPVIVVGNRSDAKTFLENVCRKYNDMLSSKKTPHVVLDFTMTGVGSETIKSFTA
ALALPTISGSFGQTGDLRQWRSLNANQTKFLLQVMPPADILPESIRAIVTKQDITNAAIIFDE
LFVMDHKYKSLLQNIPTRHVITPVKSFNKEDIKTQLRSLRELDIVNFFIVGSLRTIKNVLDA
ADENQYFGRKTAWFAFSLDKGDITCGCKDATIVYMRPTPDAKSRDRLGKIKTTYSMNGEP
EITSAFYFDLSLRTFLAVKSLLDSGKWPNNMKYITCDDYDGKNTPNRTLDLKLAFQEVKE
TPTYAPFYIPGDDPMNGRSYMEFSTDLSAVTVKDGASIGSKALGTWKAGLNSPLSLTDSD
NMSDYSAQLVYRVVTVEQQPFIIRDDNAPKGFKGYCIDLIEEIRQIVKFDYEVTLSPDGNFG
TMDENGNWNGIIKELIEKRADIALTSLSVMAERENVVDFTVPYYDLVGITIMMKLPRTPTS
LFKFLTVLENDVWLSILAAYFFTSFLMWVFDKWSPYSYQNNREKYKDDEEKREFTLKECL
WFCMTSLTPQGGGEAPKNLSGRLLAATWWLFGFIIIASYTANLAAFLTVSRLDTPIESLDDL
SKQYKIQYAPLNGSAAMTYFERMAAIEVRFYEIWKEMSLNDSLSDVERAKLAVWDYPVS
DKYSKMWQAMKEAGLPNSIEEAVQRVRDSKSSSEGFAWLGDATDVRYYVLTSCDLQMV
GDEFSRKPYAIAVQQGSPLKDQFNNAILQLLNRRRLEKLKENWWNNNPKAMKCEKQDD
QSDGISIQNIGGVFIVIFMGIGLACITLGVEYWWYKWRRRPIVGDVTQVEPAKSTRNNIGN
FVKGEGFTFRSRNFGLSDLKQKF-
>BmorIR40a
MTKLPKDFNVAIKDIAESLPSKEMTVVRGNSTNIRSQDVFELLRLLCQHNIQVVNLDIAAM
ENKEMYYGYLKKALDVSDERTNLILCEPYECENLLLELRENNLIHRTILYIFFWPYGSVSD
RFLNTMVEAMRVAVITNPRESVFRIYYNQATPNRLNHLSLVNWWAFRLYKSPLLPSADKV
YKNFRGRVFDVPVLHAPPWHFVKYNNDSSINVTGGRDDKLLKLIANKLNFRYRYYDPPD
RSQGSGIIGNGTFKGTLGLIWKRQADFFLGDVTMTWERLQAVEFSFLTLADSGAFLTHAPA
KLSETLAIIRPFRWEVWPLVCATLFITGPALWIVIAAPSLWQRKKRDQMGLLNNCCWFTVT
LFLRQSSTKEPSSTHKARLVTVLISLGATYVIGDMYSANLTSLLARPAKEPPIGTLPALEEA
MREHGYELVVESHSSSLSILENGTGVYGRLAKLMKRQRVQRVHNVEAGVRLVLNRRRVA
VLGGRETLYYDTERFGSHNFHLSEKLYTRYSAIAFQIGSPYLETINNVVMTLFEAGILGKM
TTDEYKNLPEQSRRSEPVTESENLSTEKTGETAAVTQIQNETSKGLEPVSLTMLRGAFCLL
GIGHLLAGVTLLIEIQLYRRARKRALPPQTRNPTNTFKAKAKKCILRGWRRIKAAAILAIDR
ALAPDRGID-
>BmorIR41a
IEILLQIIINKYLSESYCLVVISETPLSVKLPMSFTYLDPKKEHFSVETLLKLSEEGCSDYIIRM
EDPRQFMNALEEIRPMSMVRRSDKKLVILPVTDDENSMEPILNLLTMKESSYYAHILLILPT
QTERFECLAFNLITHRFVGSDSESKLPIILDRWYSCTNHFENNVYLFPNDLKNLNGKTMKI
STFIYKPYVLLDVDTAVAPLGRDGIEIRMIDEFCRWINCTVQIIREDVDLWGEIYENETGIGV
IGSVVEGRSDFGIAALYSWYEEWKAMDFSVSVVRSAVICLVPAPRVLESWELPFLPFGKSI
WIAVVITFVYASIGLTIAQGCSSNKALLIVFGTIISQSQYIVSDSWRIRSVIGWLLVSSLILVSA
YGAGLASTFTVPQYEPSIDTVQDLLNSRMEWGANHEAWTFSLALSSEPVAKKLIKQFKIYS
FEELQRRSFLRKMAFSLEELPAGTFAIGEYLSKEAVQDMQLMLEFFYFDHCVAMLHKNSP
YTEKLSELIGRLHQSGLLLAWESQVSLKYLDYKIQLEIRLSRARSDVGDLKPLNFNHVEGI
FLIFITGTILSTLFFALEIFIGKQARKK-
>BmorIR64a
MNILGLNIISFLCSLDISSVIEVFKCKHVRDVIVFHCFKENQLILPQRMFHFNNFRTVFVFIS

NNISWELPNSYPKIGVLINTSCDGWEKFQEFQNSHTWVYYTDNLTSTITALSTFPIEINSDV
TVVYKENSAYQVFDTYNTGRKNNGVFNVNYIGHINPGLQTNLKFTTRNLNGVTLKSTVVI
LKKVQYESFEEYLRKTEQTGLDSVHKHKFFQLLQYISEMYNITYDLIRTNTWGYAHDGRI
DGMVGSLQRHEADVGGSPIFFKTDRAYVVDYVAETWPSKQSFIFRHPKHPTGVHTVYSRP
LSNSVWYCVIAFLFVTASTVFFMLKFNIDEIERAETSQSLAFLFAWSAICQQGMSLRRNSLA
LKVVVFVTFVCSITLYQYYNATVVSTLLKESPITIRTLKDLLQSDLKVGVEDVAYVKDYFA
HTKDPIAITMYEKKIVTGNNRNFFDPEYGMSLVKKGGYAFHVDTVYSYGIMKKTFTEREI
CEIHDVTMYPPQKMGAVLKKNSPYRNYFAIGIRRLWETGLMQRMKHIWDEPKPPCVRTQ
DSSIFSVSILEFSTPLFIVVFGVIASVVVLLCETLFDTLFNMR-
>BmorIR68a
TIMPLTLFSRSPRSGKTRAVSKASPILEDIYEQKDLEFVLVDLLNHAGRYHDFTCVAVICDAI
YYNVFDGAFFKRIDTVPFVMIVVEEYDDLLSPNFDILEALREARRDGCNMYIILLANGLQA
ARLLKFGDRHRVLDTRAKYIILHDYRLFHSDLHYLWKRIVNVIFLKHHRKIGSVAKSQAW
FDLSTVPFPNPIKGVFVPRRVDLWKSGKFHYNTVPFDDKTSNLNDEVLHVVYLDHVPSVV
VVNSNETGQIGGVEIEIINTLSEKMNFRPKLYQPMNVELHKWGQKQPNGSFSGLLGEMVN
GRADLALGNLQYTPYHLELIDLSIPYTSQCWTFLTPEALTDNSWKTLLLPFKLYMWIAVLL
VLXITGTIFYGLARYQTYLHGLKRQEEMKKPVYSKPVGLYLFGEIINSILYTYGMLLVVSLP
KLPTGWSIRFLTGWYWLYCILLVVSYRASMTAILANPAPRVTIDTLVELAASKLTCGGWGI
ETKNFFQDSLDEIGQKISDRFEISNDPNIAADKVAQGTFAYYDNKNFLKYITVRRQNGFIME
TIDNTTNFTSISTKSNNERNLHIMSDCVVNIPISIGFHKNSPLKPLTDIYITRIVEVGLVEKWL
NDAMYTIKTLETNEEEIKALMNLKKLYGAFIALAIGYFLSVMCLIGELAHWNCVVKKDPN
YDKYALHKYYEKINKK-
>BmorIR75d
VNIQNNFANAPELPAYDFRREGVVLDLNCPNSKLILEKASKNRAFIHRYTWLLIHNSTYKL
ETIQKILSDAAVLPDADVTWCAADDILDIHRLNEHQPYVVMDLGLSVNSTIEDLDAVWSTI
PTAATRRRHLNNLTINAVVIVSQPQYFKGWSDLSNRQIDTFPKLTYPMLMLCAEDLRFRFN
LKQVDEYGVELNGSFTGTVGLLQRGRAELGVASMFMRSDRWRVLHFSSATVALLNAFML
RAPAQSSVSNIFLLPLSRGVWCCAAALLCGSAVLLAVLSCRLVAADPTLQLLTLPEIFVFSIG
TVCQQGFYIMPKLSSIRMIMFLTLLTSLFTFTAYSAKIVAILQTPSAAVRTVADLADSHMDV
GIQETTYKKVYYAESTDPSILRLFHRKVAPLGERVYMSVVEGVERMRTGLFAFQVERSSG
YEIISKTFTESEKCGLMEIEAFKLPMVAVPLRKHSGYRELFGTRLRWQREVGLMSRVRAIW
LAARPRCEGRGVGFRAVRLLDMLPALQMLAAGGLVAVVLLILENVYHHYSRTGNVLRRT
YRNVFKLVCYKCYTF-
>BmorIR75p
MVDVRPHRELFKRRRDVMGRPLTMANVIQDSNNTRYHLPREDALELQYDVIPKICWMTA
KLAFQMLNATPRYTFSYRWGYKVNGQWSGMINDLHTSKADLGTNCVVSDVERLSVVTY
TDMLAPFRVRFVFRQPPLPSVANIFYLPFTGRVWAAVAVCAMVYTAAIYWASKWEFNLEK
RSASQFDGTVGDAMLLTMSALSQQGCFIEPKRAPGRIMLFVLFTALMALYAAYSANIVVL
LQAPSNSITSLAQLAASKVTLAANDVDYNHFVFSLYKDPVRVMIHKRIDPETGNGQFYSLE
DGVDMIRQGFFAFHSIVEPVYRRIEETFLETEKCDLTEVDFLSSFDPFVPVKKDSPYLELLR
VVKKKKIRCSFKQIRESGIQSALNRRYQVPKPRCSNKVAAFSSVGIVDLRPVLIMMIYGIISS
CLILIMEMLVFKM-
>BmorIR75q.1
IFKIKNPVAITFGKIGNVEEVTPSNHILFLVDTTCNNSHIVLQEADAHQQFRRSYRWLVLET

QGSGYNSKLLEIEPLNILIDSDVLLATKIENVTYVLKKIYKISTQSEWITEDYGNWTAEHGLI
MSNIISSDASRRRNIRGHPVTTSIIVTENRTKSELDDLKNLLSDSLAKICFRHTKNLCQFMN
ASHKIGFASMWGYKTNGTWNGMMGDLAKGTVDFGGTIAFLTSQRLQVVDYLSSPVPINA
KFVFREPPLSYQNNLFLLPYKANVWYCTAAFVVLLVIILYINAKWEIKKAEYEQAVTLQPS
VSDVTILVISAISQQGSSNELKGTLGRAVLFLLFLTFLFLYISYSANIVALLQSNSKQIRTLQD
LLNSNLNIXDGKPLFQTATEPIRKAIYETKVAPKGSKANFMSIEEGVKKLQKSPFAFNMNIG
TGYKIIERYFEEHEKCGLQEINYIESSIPWMSCRKNSPFREIYKLGLFKLQEHGITDRENRLL
FARKPVCIVRGGNVGSVNMVDVYPVILMFLYGLFLAFLILLVEIVVHRKL-
>BmorIR75q.2
MSGGFQKNTNINEVIAVRRRDLEGYEIKICYVLTDNDSIHHLSDEVNDHIDTITKVNFPSTN
HLLDFLNAERKYVFVNTWGYRINGTWNGLTGFLVNGDVEIGGSPMFFTAERTAVVDFISSP
TPTRSKFVFQQPKLSYENNLFLLSFRTAVWYSTLALISLIFTMLLSVTAWEWKKMSQIKTR
DIDAGVLRPSVTDVTMLVFGATCQQGSTVELKGSLGRVVMLILFLTLMFLYTSYSANIVAL
LQSSSSQIKTLEDLLHSRLKFGVHDTVFNRYYFSTADEPVRKAIYEKKIAPPGVAPQFMSM
EEGVKKMRKGLFAFHMETGVGYKFVGKYFKESEKCGLKEIQYLQVIDPWLAVRKNTPYK
EMFKIGMKRIQEHGLQNRENRLLYEKRPKCSGRESNFVSVSMVDCYPALLVLSYGIIIAIAL
VIMENLWQYRHLIKGKLEFFSSVNTIENFNQFEKHPSHNWKKFYVIDSAKIKKINPSTN-
>BmorIR76b
MLFSNAQRSGQNFPLSWIERDENGTVQAYGVAFKIIDILQQKFNFTYEIVIPHRNFEIGGSK
PEDSLIGLTNTSKVDMIAAFIPRLVRFRKLVTFSRDLDEGVWMMMLRRPKESAAGSGLLA
PFNNFVWYVTLASVLCYGPCICFLTHVRSKLIKNEERPLRLSPSFWFVYSAFIKQSTNLAPE
ANTTRVLFATWWLFIILLSAFYTANLTAFLTLSKFTLDIETPEDLYKKNYRWVSVEGGSVQY
TVKTQDEDLYYLNKMVTSGRAEFRTLSPDQEYLPIVKAGAVLVKEMISLEHLMYGDYLTK
TREGVEEAKRCTYVVAPKPFMKKPRAFVYPVGSKLKSLFDPTLAYILQSGIIDYLEHKDLP
STTICPLDLQSKDRQLTNSHLMMTYYIMCVGLASGLAVFVVEILVKRYINIKIKPIDKVKLK
KFKRSKRSPRYDDSGPPPYESLFVKPKFKDSEKRWKMINGREYYVYEDARGGTRLVPVRT
PSAFLYR-
>BmorIR87a
MTTGNSDQIAKTAECVLKLSAKYFVERKALSGSIVIINVNSYSSTTQGLLLKTIHSSIKYSV
MAKDSFYPHANASHFPEKAKNYMLILEERTELKRNIFQLNKLPSWNPLAKAVVFYQIKGN
ESAQRIAIEFINELREHKFFRSIIFINNGTESGVTSYTWRPYSENNCGGKCDSVYVLDRCKN
NIVEQIEPQPEWFPSNMNGCPLTTYAIVSEPYVMPPIRKIPNAKFDDVYEFQKGGETNLVKT
IAEFSNMTLIVRLSAIEENWGIIYANGTATGAYGVLRNDSVDIVFGNIEVTKQIRKWFHPTIS
YTQDEITWCLPKAGQASAWDNLVIIFQWTIWVATFTSLILMGLLFHYMYYREKNKKITKW
PTNSLLMTFSMLLGWGSHFEPKTATFRILIFGWLCFSINMGISYESFLRSFLMHPRFEKQIAT
ESDLIQSGIRFGGREIYRTYFESNDASSSYLHTEYSSTTFSEGIRRAALNRDFAVVSSRRQAE
YQDQKLGKGASLIYCFPESDNLYKYSVVLLARKWFPMLERFNGIIRSVSENGLINKWNDE
MFIHRVSLEGASTIVPLSIQHLLGAFMFIGFMYGTSAFIFLVEVFVGFVQRRAFLSAFFCGK
KKRFSAVFKVKV-
>BmorIR93a
MKIWVLGVLCLAISVQGEDFPSLITANASIAVILDRQYLGDKYQTVLDELKDYIKELARVE
LKHGGVLVHYYSWTNISLNKGFLAVFSIASCEDTWELFSRTEEEDLLLFALTEVDCPRLPQ
RSAITVTYSEPGEELPQLLLDLRSSNAISWKSAVILHDDTLGRDMVSRVVQSLTSQIDEESA
RPVSVTVFKMKHEMNEYLRRKEMHRVLSKLPVKYIGENFIAIVTSDVMTTMAEIARELL

MSHTMAQWLYVISDTNAHASNLSGFINTLNEGENVAFIYNITENGPDCKNGLMCYSQEM
MSAFISALDAAIQAEFDVAAQVSDEEWEAIRPSKVQRRDILLKHMQQYILAKSVCGNCTL
WRALAADTWGVTYRQNDVPEQINEHANGSTGVIEHLELMNVGIWRPIDAMTFADLLFPH
VHHGFRGKELPIITYHNPPWTFLQANESGAIVKYSGLMFDIVNQLAKNKNFQRLPHPSNR
NALLLHGRNRQGGGTYPCGLTKGPITYNNIPLYFRAVFIAHQAGVNLKNNYYRCINYTIPV
STQPHTFIVARPRELSRALLFLLPFTTDTWLCLGFAVILMGPMLYIVHRLSPYYEAMEITRE
GGLATIHNCLWYIYGALLQQGGMYLPRADSGRLVIGTWWLVVLVIVTTYSGNLVAFLTFP
KLEAPVTTISELLKNSDAYTWSVTKGSYLEMELKNSEEPKYKRLIKEAELLKETGGIEGTI
HAARGTLDRVRGQRHLIFDWRLRLTYLMSADHIATETCDFALAVEDFMEEQVAMIVPAGS
PYLPVINKEINRMHKAGLISKWLSAYLPKPNRCLKISTVTQEVSNHTVNLSDMQGSFFVLF
LGNDKIYVYMYIAELI-
>BmorIR143
MFSVKYPRFYLFIIQIASNFGDAVTMPQLNSSINSKSATDCLIKVCYADLSFRRTVILKHVSY
ESDEENAFYNEIIHAVNNNNIQLVVLEEIDNLNDTINIDDADWLVVVYFKNCKALTEFNVK
IVFEKIKYFIIVSDDLNEDCTSKMKTIGNVINKYDVTFVFNENKEDNFKFMTFIPQIDEETC
KEIVTLPKIVNICANGQIERKSIFPSKNPKDIKKCPINVGMGSLYPFGIINHKEKYKTFDPLN
ETEVRGLDVDLVKVLVNQFNGTLNLYFIYKKEENPFGQLDFIPLVLNGSLDVIAGGFYRIY
GNVVAYSGIYTSQAVTWMYVANRTTKSWQSLIVKIDGLYIFVIFHLIYSYVWYFVRKFDEQ
AVDFRNTILYSWGALVGTTSLQDALSLKQRILNLTYLIMCVHLSAYVSLHLYYFLTVLEPPE
LLKSNDDVMRSGRPAFLIPISKYFVLDEKYLSFANASEECTKFQDCSDLSLLRNGVTIILQG
FFLNYQARTAINYEAKVLSAAENVLTVYYEMLLRKNSPYVERLQKLMTHLFEAGIPDRFY
RHAIGLTVIGKAHSACQNTVSNSYSCQSGCKITFDQFAGVFYLWLFGCVLSCGAFIFELFSK
FGRA-
>DpleIGR1
MGHPLTISNVIQDSNTSQYHIIKENRLELHYDGTTKLSYVHVQIAFQMLNATPRHVFSHRW
GYKKNGQWSGMINDINTGRADLGTNCVPAVERLSVVVFTDCIANFEVKFIFRQPPLSYVS
NIFTLPFSKSVWIAIATSFAISTITIYIATKWEDHTQLDGSIFDAMLITMSALSQQGCSKEPKK
LSGRIMSFVIFSALMAVYTAYSANIMVLLQAPSSAITTLEQLANSDIKLAAHDVDYNHFVL
KAQKDPIRKAIYRKISPEKGKENFYNFNEGVELLRQGLFAFHAILELVYLRVEETFLENEKC
DLMQLDFMNSHDPFVPVYKHSPYLELLRVVFKRIRESGIQMANHRRFQVPKPRCTEKISTF
SSVGIVHMKPVLLFITYGFLAAFLIMLAEIFVFRMKMFKRKELKYFSLRNRPSKENLTIKFP
N-
>DpleIGR2
MPEGNWKVETFGTWTYEMGLKKLITMNIPSSQRRRNLEGTKIGTSMVVNDNDTRMDILS
MRNLNTDHLTKAGYRQILPLYYFMNATLITVFTDTWGYLRNGSYDGMIGEVTRGTAELTG
TLGRVVMFLMFFVFLFLHTSYSANIVALLQSSSNDIKTLADLLNSKLELGAEDTPYNRQHL
STASEPIRKAIYEKKIAPAGSKPNFMTLEEGVKRIQMKPFAFHMYLGGGYRLVEKYFLEHE
KCGLQEIQFNHETIPWVTCRKNSPYKEIFKIGLLRNQEHGLNDRVNRLIYSRKPVCSVHGG
TFGSVNMTDFYPALLMLVYGMIASLLLLAIECLASQHLCHIRNRI-
>DpleIGR3
MSPPPKKFDPQMILTWNPASNVPALTHTSSIFMHEIAEIHNIRYNYTVVDVWIGDYKREHA
YVPNYYYNTKHAKAEDELKKKMKKLEKKGKPILIFPKDGISLVKEGGYVYHGEVDSANK
IIAETFNQRELCDLGSLQSMDKTFAYISVQKRSPYKEFFTWSSLRLMETGILNRIHKRTLSG
ANKCEGSTPRALALGGAAPAFILLAAGYILAVILVVLESSKGFQISKYIQTKVSIIAEFLIINR

ALPPLIGEEGEETMRKLEGGKTERAGKGGNREETEKEGKREGQPGLAPIETRRNLSAFHG
FIEPVPGPSLCRERSLNCAWDLRPRVNVMTPRKPFIFGDICGEDTVSQDTMSKILIVLLLVF
VAGDFAQRYRRPIYRRRGRSVEEPSYNTDDYQPDPEYTIPFLLGDIKLEQQRQQTGLDLSD
AKPGHELSYATSASALSTPDKDGIVPESPPASIFLNPSALPSKHRK-
>DpleIGR4
MLGDVFRSLQKPCFVVAFSCWTQEQKLMAYSALAGRNVENDIIIKFVESDKIFNNKEIKH
HTVFLADLNCPQDHVNVNWTNPALFRFPYRWVIIDKDSGENGTVPDVIRDIDVLIDSEVV
VIRRINDASFQIYYVFKIGPNTTWLNELYGQWNESGLFKTESMLEVTALRRMNLNNYEIRI
CYVLTDNDSINHLADEVNDHIDTITKVNFPTTNQLLDFLNASRRYVFTNTWGYKHNVTW
SGMTGFLDREEVDIGGSPMFLTGERLSVVEYISSPTPTRSKFVFQQPKLSYENNLFLLSFKR
SLWLGSVVLISMLFLFLFLVTVWEWKRTSIHKRTIRTRNAGILRPNVVDVALLIFGATCQQG
STVELKGSLGRMVLFLLFIMLTFLYTSYSANIVALLQSSSSQIRTLEDLLHSRISFGVHDTVF
NRYYFSTETEPVRRAIYEKKVAPPGSAPRFMTMEEGVQEIRKGLFAFHMETGVGYKFVGR
YFEEGEKCGLKEIQYLRVIDPWLAVRKETPFKEMFKIGTKRLQEHGLQQRENRLLYEKKP
KCTGRQANFISVSMVDCYPALLILSYGTLISISVLFAENLYYNRRRLLHRMQSKKKNMVKE
IYKIYPPKAVQFIEINDNIVPDSLTNTIIVMDMKCPKTSEFLKKVETFGTWTYEMGLKKLIT
MNIPSSQRRRNLEGTKIGTSMVVNDNDTRMDILSMRNLNTDHLTKAGYRQILPLYYFMN
ATLITVFTDTWGYLRNGSYDGMIGEVTRGTAELTGTLGRVVMFLMFFVFLFLHTSYSANI
VALLQSSSNDIKTLADLLNSKLELGAEDTPYNRQHLSVS-
>DpleIGR5
MADSNTRYHAMIYKDDYLPQNLCIECMQRLKNCYKFRTQTLQAQTIIVDLIRIGNFTTEAI
KSLNHNPKCKLQMQRFDANHCDAYINKEDVNPIQEIVIEEQVKIEKEFINEQDTVAMGYTT
DDSLPLETQRTKAKKTKKKKEKKIQEPKVDRRRKPFLNDDLNESLFTITDLTLEEQIADIQ
KRQESSNFKNSVYKCMECFKGFLDEGAYNGHMTRHTTSVYGVMEDYRGVRMLPVSARR
RDLRKHNLTMANVITDSNETRQHLDDRLNLHQDSITKMSYVVAKICFDMLNATENRIFTH
TWGYKDKNGNWQGIIDHLLKKKADLGTLTIFTQERMKAIDYIAMVGSTAVRFVFREPPLA
LLENIFTLPFTSAVWIAIGICVLGCAVFLYITSKWEATVGMHPLQLSGSWADVLILIIGAVLQ
QGCTLEPRYAAGRCVTLLLFVSLTVLFAAYSANIVVLLRAPSSSVRSLPDLLNSPLKLGASD
FEYNRYFFKKLNDPIRKAIYSKKIAPSGKKPNFYSMKEGVEKIRKGLFAFHMELNPGYRLI
QETYQEEEKCDLVEIDYINEIDPWLPGQKRSPYKDLFKIRS-
>DpleIGR6
MRILCLCVALCSVFSQNVASIDADLAVDYFLKKHVPFVCYLTCGLRRISFDLAQHNKLVKA
FLRSNIRISLMRVEKHALDLNKNLHQWTFPIGVLMDGSCNGISNVLEQASRSALFDDGHM
WLIFENMNEKRRGDVRIENLLQNLNLSINTDIVVALNTEDTDIQLMDVFNFGKIQGNNLEK
KFIGTWSPGRGLNISLSRFKYYDRWDLHNLTLRAITVVLGAPQGYDPEMILQPGYDWRVA
LITKSAAQLLAIIRENHNFRFNYTIADLWVGSPLKNSTLGVTNSIYWGEQDISFTSLRMFSP
WMEWVDPFFPPTTKLETKFFYLILDKGVGNYENRFLTPLSSGVWWCTAVASVVCVSVLA
VTAVLEERDEPGLYALFSVFAVICQQAYEDGVQLFDEFSSSQGRRLILLVVGLMSMLLYNY
YTSSVVSWLLNAAAPTMSNIDELIDSDFELVFEDTSYTRGWLNNPGFFYYSGLKNPKEDIL
RNRATVTKRTTKLLQTAAEGMELIRSGDYAFHTEPYTAYQAISRSFKEKDICTLGSLQIMIP
ANTYIVGQKRSPYKKFFVWSLMRLLERGHTSAVRARASGVTPICSGTVPRALALGQAAPA
FAILAQTAVVSFIILLFEIYWHRRRSHDKKQVRVLNNRAHNAQEVGKNDSLRV-
>DpleIGR7
MNTYTCSEVSGTGRTPAPLDRPSSEHTDRQAPSARWSSLTAYKIAEYSEVILSLRAVWRPA

RYDFKNSTLVEIVKQNKIVKSVYGVMEDYRGVRMLPVSARRRDLRKHNLTMANVITDSN
ETRQHLDDRLNLHQDSITKMSYVVAKICFDMLNATENRIFTHTWGYKDKNGNWQGIIDH
LLKKKADLGTLTIFTQERMKAIDYIAMVGSTAVRFVFREPPLALLENIFTLPFTSAVWIAIGI
CVLGCAVFLYITSKWEATVGMHPLQLSGSWADVLILIIGAVLQQGCTLEPRYAAGRCVTLL
LFVSLTVLFAAYSANIVVLLRAPSSSVRSLPDLLNSPLKLGASDFEYNRYFFKKLNDPIRKAI
YSKKIAPSGKKPNFYSMKEGVEKIRKGLFAFHMELNPGYRLIQETYQEEEKCDLVEIDYIN
EIDPWLPGQKRSPYKDLFKISFIKIRESGVQSCVHRRLHVGRPRCSGSVSTFSSVGITDMYP
ALQATLYGAVMSVAVLMMEKVHYKL-
>DpleIGR8
MKRNSRIDLSGVVLKCVVVVLDPIVNQTFKQYLDNAKPGIVDSLHKLKFYVLLKYLEDL
CKFRYKLERTNSWGYLRNGSFDGMVGALQRREADIGGTPIFYRPDRARFIDYTTPTWQSR
HY-
>DpleIGRIa
MESVCSGVAEGIPEVAVLLEQAVAAGGGAAAGERGQSLEPYEPLAVPEHICTQASEGLLAS
VGGVEAADAAARAGLLLLLASPVAVPSPAMTIASTEPDHPLSAALEFYPRYDVLAEACAA
LCEAKGWKHAVLLHDGSGSAAPLIVPDHDTLALRVRQLPSREDDDALRNLLLVLKKFGA
VNFIVWCSAECSVRVLDAAQRVGLLSERHSYLMLSLDLHTLPLEDFSYGGANITSLRLFDP
ESSAVNVSMEKWQQQYINLLGNEANEEIDKIISNPPTSLLLSYDAAKIVSEGMEYLDLPFM
EDSPSCQQGTAAFHADTLLNYLRSEENSGATGPLWWEATGARGGVRLHVAELERGGFLR
AAGDWSRTGGLTWRPRPPAPPPPPDAMTNRTFTVLIAQNQPYVMRQQSSERLSGNARYEG
FCIELVDRLAQLLHFNYTFIEQADRAYGSLNKTTKQWNGMMRRLMDDKNVDFAITDLTIT
AEREEIVDFTTPFMTLGISILFHKPQPPAPELLAFLLPFSNGVWMCLGLAYVGSSLVLYVVG
RLCPEEWQNPYPCIEEPSALENQFTLANALWFNLGAVLQQGSEIAPIAYGTRAVASIWWMF
ALVITSSYTANLATLLASKTSTELIRNVRELAENDQGITYGAKSSGSTYTFFEMSSSEPYKS
MFQKMKDVTMPSTNEEGIEKVMNEKYAFFAESTTIDYTTERNCEVTRVGDLLDSKGYGIA
MKKNSPYRQALNLALLNLQEAGILREMKHRWWKEMHGGGACQDKEDHATERLTIDNFK
GLILVLTVGCALGIVMSCCDLAWSAWRHPRDPTRSFAASFWSELRFVFRFEQSEKPVRGAL
TPAPSSHDSPPSAHSERSELTTGSGVDGRGRGREEDDNHGEDDVGSRFSARSRRTSARRCS
MHAASLRLARHTTPRR-
>DmelGluRIIC
MWQRILLLGCMWSAFFMCRSRGQQINIGAFFYDDELELEKEFMTVVNAINGPESEQTMR
FYPLIKRLKPEDGSVTMQEHACDLIDNGVAAIFGPSSKAASDIVALVCNSTGIPHIEFDISDE
GIQAEKPNHQMTLNLYPAQAILSKAYADIVQNFGWRKFTIVYDADDARAAARLQDLLQL
REVHNDVVRVRKFHKDDDFRVMWKSIRGERRVVLDCEPNMLVELLNSSTEFGLTGQYNH
IFLTNLETYTDHLEELAADNETFAVNITAARLLVNPDPPPYSLPYGYVTQRDNIVYESSDPP
RTLIHDLIHDALQLFAQSWRNASFFYPDRMVVPRITCDFAASGGRTWAMGRYLARLMKG
TSGVNNTNFRTSILQFDEDGQRITFNIEVYDPLDGIGIAIWDPRGQITQLNVDVKAQKKMIY
RVATRIGPPYFSYNETARELNLTGNALYQGYAVDLIDAIARHVGFEYVFVPVADQQYGKLD
KETKQWNGIIGEIINNDAHMGICDLTITQARKTAVDFTVPFMQLGVSILAYKSPHVEKTLD
AYLAPFGGEVWIWILISVFVMTFLKTIVARISKMDWENPHPCNRDPEVLENQWRIHNTGW
LTVASIMTAGCDILPRSPQVRMFEATWWIFAIIIANSYTANLAAFLTSSKMEGSIANLKDLSA
QKKVKFGTIYGGSTYNLLADSNETVYRLAFNLMNNDDPSAYTKDNLEGVDRVRKNRGD
YMFLMETTTLEYHREQNCDLRSVGEKFGEKHYAIAVPFGAEYRSNLSVAILKLSERGELYD
LKQKWWKNPNASCFEEPDPDATPDMTFEELRGIFYTLYAGILIAFLIGITEFLVYVQQVALE

ERLTFKDAFKKEIRFVLCVWNNRKPIVAGTPISSVRTTPRRSLDKSLDRTPKSSRRVVIGRSS
EEMREMAQGSGSSSGSNNAGRGEKEARV-
>DmelGluRIIA
MRLCPVVIYAFIIIIGFLEGIIALGGDDRNEITVGAIFYENEKEIELSFDQAFREVNNMKFSEL
RFVTIKRYMPTNDSFLLQQITCELISNGVAAIFGPSSKAASDIVAQIANATGIPHIEYDLKLEA
TRQEQLNHQMSINVAPSLSVLSRAYFEIIKSNYEWRTFTLIYETPEGLARLQDLMNIQALNS
DYVKLRNLADYADDYRILWKETDETFHEQRIILDCEPKTLKELLKVSIDFKLQGPFRNWFL
THLDTHNSGLRDIYNEDFKANITSVRLKVVDANPFERKKTRLTKVDQILGNQTMLPILIYD
AVVLFASSARNVIAAMQPFHPPNRHCGSSSPWMLGAFIVNEMKTISEDDVEPHFKTENMK
LDEYGQRIHFNLEIYKPTVNEPMMVWTPDNGIKKRLLNLELESAGTTQDFSEQRKVYTVV
THYEEPYFMMKEDHENFRGREKYEGYAVDLISKLSELMEFDYEFMIVNGNGKYNPETKQ
WDGIIRKLIDHHAQIGVCDLTITQMRRSVVDFTVPFMQLGISILHYKSPPEPKNQFAFLEPF
AVEVWIYMIFAQLIMTLAFVFIARLSYREWLPPNPAIQDPDELENIWNVNNSTWLMVGSIM
QQGCDILPRGPHMRILTGMWWFFALMMLSTYTANLAAFLTSNKWQSSIKSLQDLIEQDKV
HFGSMRGGSTSLFFSESNDTDYQRAWNQMKDFNPSAFTSTNKEGVARVRKEKGGYAFLM
ETTSLTYNIERNCDLTQIGEQIGEKHYGLAVPLGSDYRTNLSVSILQLSERGELQKMKNKW
WKNHNVTCDSYHEVDGDELSIIELGGVFLVLAGGVLIGVILGIFEFLWNVQNVAVEERVTP
WQAFKAELIFALKFWVRKKPMRISSSSDKSSSRRSSGSRRSSKEKSRSKTVS-
>DmelGluRIIB
MHGLQFLVLLALAIASGANEDTLVIKIGAIFFDTEMKLADAFSAALEEVNAINPALKLDAI
KRYVTVDDSIVLQDISCDLIGSGVAAIFGPSSKTNSDIVEVLCNMTGIPHLQFDWHPQQSNR
ERMNHQLTVNVAPMELFLSAAFSDILASKTFDWKSFTIAYERSSHLIRLQHILAWKQLHKA
GIKMQEFERGDDYRILWKRINNAREKFVLLDCPSDILVDVINASIGYNMTGSFNHLFLTNL
DTHLSGIDGFYSRDFTVAVAAVRIRTYVPPPVHDEIDVFDNSVDTRFSSLGSQLVYDSIVLF
YNALLEISQRPGFYIPNFSCGRGFWQPGPRLVEQMKQITPKMVKPPFKTQRLQINADGQRE
DFNLEVYNPIIDRVTHIWNKEFQLVDFEKLRENSTQALKQKRLQNKEDFSQKPIRYTVATR
VGKPYFSWREEPEGVHYEGNERFEGYAVDLIYMLAQECKFDFNFEPVRDNKYGSYDANT
DEWDGIIRQLIDNNAQIGICDLTITQARRSVVDFTVPFMQLGISILSYKEPPPKADIYAFLNP
YNAEVWLFVMIAMMITAFALIFTGRIDQYEWDQPVENVNREMERQNIWHLSNALWLVLG
SMLNQGCDLLPRGLPMRLLTAFWWIFALLISQTYIAKLAAFITSSKIAGDIGSLHDLVDQNK
VQFGTIRGGATSVYFSESNDTDNRMAWNKMLSFKPDAFTKNNEEGVDRVKLSKGTYAFL
METTNLQYYVQRNCELTQIGESFGEKHYGIAVPLNADFRSNLSVGILRLSERGELFKLRNK
WFNSNESTCDSNVPTIDDGQFDMDSVGGLFVVLIVGVVVGLVIGVAEFLWHVQRISVKEK
IPPMLALKAEFYFVIRFWLTRKPLHTYRQSRDSTSTGYSSLEQITSASSAKKKKKTRRIEK-
>DmelClumsy
MYSLFLTHFLLIALPVLADIDRSQFMVGSIFTSDKDESEIAFRTAVDRANILERNVELVPIVV
YANTDDSFIMEKMVCNLISQGVIAIFGPSTGSSSDIIASICDTLDIPHIVYDWIPNESIPDREH
STMTLNVHPDNLLLSQGLAEIVQSFAWRSFTVVYETDKELQQLQDILQVGEPISNPTTVKQ
LGPGDDHRPFLKEIKLSTDNCLILHCAPDNLLKILQQANELKMLGEYQSVFIPLLDTHSIDF
GELSGVEANITTVRLMDPSDFHVKNVVHDWEEREKREGRYFKVDPNRVKSQMILLNDAV
WLFSKGLTELGIFEELTAPDLECRRKKPWPFGKRIIEFIKARSEETSTGRIDFNENGQRSFFT
LRFMELNSDGFLDLATWDPVNGLDVLNDDEESEKRVGQKLSNKTFIVSSRLGAPFLTLREP
QEGEILTGNSRYEGYSIDLINEIAKMLNFKFEFRMSPDGKYGALNKVTQTWDGIVRQLIDG
NADLGICDLTMTSSRRQAVDFTPPFMTLGISILFSKPPTPPTDLFSFLSPFSLDVWIYMGSAY

LFISLLLFALARMAPDDWENPHPCKEPEEVENIWSIMNTTWLSIGSLMGQGCDILPKAAST
RLVTGMWWFFALMMLNSYTANLAAFLTNSRQANSINSAEDLAAQSKIKYGAMAGGSTM
GFFRDSNFSTYQKMWTAMESASPSVFTKTNDEGVERVQKGKNLYAFLMESTTLEYNVER
KCDLVQIGGWLDYKSYGIAMPFNSPYRKQISAAVLKLGELGQLAELKRKWWKEMHGGG
NCEKSDEDGGDTPELGLENVGGVFLVLGLGLLSAMVLGCTEFLWNVKSVAIEEKISLKEA
FKSEALFAARIWITTKPVHTSSESGSSNSSSSSSSRSKHSFKSQGLSMKSLKSSGYQDVEAS
VHSKLKKIGSMFSLKSQKTVTPPPEIGWKLDKSTQIDVVPTSDVDQELIPEVEPHLPHRHH
HHHHHRHHHHHHQPDQEHDRNPSPPE-
>DmelGluRIID
MHFCWISLIILSLSRVQAQFYGGNAYEASSGQSIRLGLITDDATDRIRQTFEHAISVVNNEL
GVPLVGETEQVAYGNSVQAFAQLCRLMQSGVGAVFGPAARHTASHLLNACDSKDIPFIYP
HLSWGSNPDGFNLHPSPEDIANALYDIVNQFEWSRFIFCYESAEYLKILDHLMTRYGIKGP
VIKVMRYDLNLNGNYKSVLRRIRKSEDSRIVVVGSTTGVAELLRQAQQVGIMNEDYTYII
GNLNLHTFDLEEYKYSEANITGIRMFSPDQEEVRDLMEKLHQELGESEPVNSGSTFITME
MALTYDAVRVIAETTKHLPYQPQMLNCSERHDNVQPDGSTFRNYMRSLEIKEKTITGRIYF
EGNVRKGFTFDVIELQTSGLVKVGTWEEGKDFEFQRPPQAVNFNDIDDGSLVNKTFIVLIS
VATKPYASLVESIDTLIGNNQFQGYGVDLIKELADKLGFNFTFRDGGNDYGSFNKTTNSTS
GMLKEIVEGRADLAITDLTITSEREEVIDFSIPFMNLGIAILYVKPQKAPPALFSFMDPFSSEV
WLYLGIAYLGVSLCFFIIGRLSPIEWDNPYPCIEEPEELENQFTINNSLWFTTGALLQQGSEI
APKALSTRTISAIWWFFTLIMVSSYTANLAAFLTIENPTSPINSVKDLADNKDDVQYGAKR
TGSTRNFFSTSEEPIYIKMNEYLNAHPEMLMENNQQGVDKVKSGTKYAFLMESTSIEFNT
VRECNLTKVGDPLDEKGYGIAMVKNWPYRDKFNKALLELQEQGVLARLKNKWWNEVG
AGVCSAKSDDDGPSELGVDNLSGIYVVLVIGSIISIIISILCWCYFVYKKAKNYEVPFCDAL
AEEFRIVIRFSENERPLKSAQSIYSRSRNSSQSIESLKTDSEENMPVED-
>DmelGluRIIE
MFFNHFVILWSLFSIHISVNWAQYENFGGYDNYQSLESVPIGLLTDQNTEQMNIVFDHAID
VANQEVGTSLTSLKEEVNYGDAYQSYGKLCRMLETGIAGVFGPSSRHTAVHLMSICDAMD
IPHIYSYMSENAEGFNLHPHPADLAKALYSLITEFNWTRFIFLYESAEYLNILNELTTMLGK
SGTVITVLRYDMQLNGNYKQVLRRVRKSVDNRIVVVGSSETMPEFLNQAQQVGIINEDY
KYIIGNLDFHSFDLEEYKYSEANITGLRLFSPEKMAVKELLMKLGYPTDQDEFRNGSCPIT
VEMALTYDAVQLFAQTLKNLPFKPMPQNCSQRTESVRDDGSSFKNYMRTLRLTDRLLTGP
IYFEGNVRKGYHLDVIELQPSGIVKVGTWDEDRQYRPQRLAPTTAQFDSVDNSLANKTFII
LLSVPNKPYAQLVETYKQLEGNSQYEGYGVDLIKELADKLGFNFTFVNGGNDYGSYNKS
TNESTGMLREIMTGRADLAITDLTITSEREQALDFTIPFMNLGIAILYLKPQKATPELFTFMD
PFSEEVWWFLGFSFLGVSLSFFILGRLSPSEWDNPYPCIEEPEELENQFTLGNSIWFTTGAL
LQQGSEIGPKALSTRTVASFWWFFTLIVVSSYTANLAAFLTIEKPQSLINSVDDLADNKDGV
VYGAKKTGSTRNFFMTSAEERYKKMNKFMSENPQYLTEDNMEGVNRVKTNTHYAFLME
STSIEYNTKRECNLKKIGDALDEKGYGIAMRKDWPHRGKFNNALLELQEQGVLEKMKN
KWWNEVGTGICATKEDAPDATPLDMNNLEGVFFVLLVGSCCALLYGIISWVLFVMKKAH
HYRVPLRDALKEEFQFVIDFNNYVRVLKNSASIYSRSRQSSMSVASVAQESQ-
>DmelCG3822
MRSSGVLVLPLLLLQLILNCRKAQSLPDIIKIGGLFHPADDHQELAFRQAVDRINADRSILPR
SKLVAQIERISPFDSFHAGKRVCGLLNIGVAAIFGPQSSHTASHVQSICDNMEIPHLENRWD
YRLRRESCLVNLYPHPNTLSKAYVDIVRHWGWKTFTIIYENNDGIVRLQELLKAHGMTPF

PITVRQLSDSGDYRPLLKQIKNSAEAHIVLDCSTERIHEVLKQAQQIGMMSDYHSYLVTSL
DLHTVNLDEFRYGGTNITGFRLINEKIVSDVVRQWSIDEKGLLRSANLTTVRSETALMYDA
VHLFAKALHDLDTSQQIDIHPISCDGQSTWQHGFSLINYMKIVEMKGLTNVIKFDHQGFRT
DFMLDIVELTPAGIRKIGTWNSTLPDGINFTRTFSQKQQEIEANLKNKTLVVTTILSNPYCM
RKESAIPLSGNDQFEGYAVDLIHEISKSLGFNYKIQLVPDGSYGSLNKLTGEWNGMIRELLE
QRADLAIADLTITFEREQAVDFTTPFMNLGVSILYRKPIKQPPNLFSFLSPLSLDVWIYMATA
YLGVSVLLFILAKFTPYEWPAYTDAHGEKVESQFTLLNCMWFAIGSLMQQGCDFLPKALS
TRMVAGIWWFFTLIMISSYTANLAAFLTVERMDSPIESAEDLAKQTRIKYGALKGGSTAAF
FRDSKISTYQRMWSFMESARPSVFTASNGEGVERVAKGKGSYAFLMESTSIEYVTERNCEL
TQVGGMLDTKSYGIATPPNSPYRTAINSVILKLQEEGKLHILKTKWWKEKRGGGKCRVET
SKSSSAANELGLANVGGVFVVLMGGMGVACVIAVCEFVWKSRKVAVEERLSAILNE-
>DmelCG5621
MISTEASFPLGFILTSLLLAFPGCRGERTNVGLVYENTDPDLEKIFHLAISKANEENEDLQLH
GVSVSIEPGNSFETSKKLCKMLRQNLVAVFGPTSNLAARHAMSICDAKELPFLDTRWDFG
AQLPTINLHPHPATLGVALRDMVVALGWESFTIIYESGEYLPTVRELLQMYGTAGPTVTVR
RYELDLNGNYRNVLRRIRNADDFSFVVVGSMATLPEFFKQAQQVGLVTSDYRYIIGNLDW
HTMDLEPYQHAGTNITGLRLVSPDSEQVQEVAKALYESEEPFQNVSCPLTNSMALVYDGV
QLLAETYKHVNFRPVALSCNDDSAWDKGYTLVNYMKSLTLNGLTGPIRFDYEGLRTDFKL
EVIELAVSGMQKIGQWSGEDGFQENRPAPAHSLEPDMRSLVNKSFVVITAISEPYGMLKET
SEKLEGNDQFEGFGIELIDELSKKLGFSYTWRLQEDNKYGGIDPKTGEWNGMLREIIDSRA
DMGITDLTMTSERESGVDFTIPFMSLGIGILFRKPMKEPPKLFSFMSPFSGEVWLWLGLAY
MGVSISMFVLGRLSPAEWDNPYPCIEEPTELENQFSFANCLWFSIGALLQQGSELAPKAYS
TRAVAASWWFFTLILVSSYTANLAAFLTVESLVTPINDADDLSKNKGGVNYGAKIGGATFN
FFKESNYPTYQRMYEFMRDNPQYMTNTNQEGVDRVENSNYAFLMESTTIEYITERRCTLT
QVGALLDEKGYGIAMRKNWPYRDTLSQAVLEMQEQGLLTKMKTKWWQEKRGGGACS
DADEDSGAVALEISNLGGVFLVMGVGSFFGIFVSLLEMVLGVKERSDENQEAPDSDASSL
GFANLGGVYLVMFVGSCFGSIYGLVNCVVSVYLRARENKVSFKTELLDEIRFILQCSGNTK
AVKYPKNSSRSNASSKSKGSSMSVDSLPEDTSEADASGKHNHGKK-
>DmelCG9935
MLIASGFLLFQFLSYGLGVPPLVRIGAIFSNQPGMYNSELAFRYAIHRLNMDKSLLPETTVD
YYVEYVNRFDSFETVQKVCKLIRVGVQAVFSPTDSVLATHINSICDALDIPNIGRSAHDFSI
NVYPSKQLVNYAFNDVIQYLNWTRFGILHEKENGIINLHQLSRSFHGEVHMRQVSRDSYV
SALNEFKGKEIHNIIIDTNSNGISILLKNILQQQMNEYKYHYLFTSFDLETYDLEDFKYNFV
NITSFRLVDTADVGVKQILKDIGLYSHHIFKKPYLNLHIKKSTILESEPALMFDSVYVFAIGL
QTLEQSHSLTLLNISCEEENSWDGGLSLINYLNAVEWKGLTGPIQFKDGQRVQFKLDLIKL
KQHSIVKVGEWTPHGHLNITEPSMFFDAGSMNVTLVVITILETPYVMMHYGKNFTGNERF
YGFCVDILETISREVGFDYILDLVPDRKYGAKDPETGEWNGMVAQLMKYKADLAVGSMT
ITYARESVIDFTKPFMNLGISILFKVPTSEPTRLFSFMNPLAIEIWIYVLIAYFLVSLCIYIVGK
LSPIEWKCINACDLENISIGNQFSLTDSFWFTIGTFMQQSPDIYPRAMSTRIISSTWGFFSLIIV
ASYTANLAAFLTTERMINPIENAEDLASQTEISYGTLDSGSTMTFFRDSVIETYKKIWRSMD
NKKPSAFTTTYEDGIKRVNQGNYAFLMESTMLDYIVQRDCNLTQIGGLLDTKGYGIATPK
GSPWRDKISLAILELQERGDIQMLYDKWWKNTDETCTRKNTSKQSKANSLGLESIGGVFV
VLIAGIIVAAVVAFFEFWYNFRYNYEATPSQSVVNNKYNQDGILESERNYTPPDRSFWIEIA
EELRYASWCMNKQKRPALTRTCSKCTIPKGQRINKL-

>DmelCG11155
MVRKKREIVIKENIQGRSYLKKICCSYIILSILVISNALPPVIRVGAIFTEDERESSIESAFKYAI
YRINKEKTLLPNTQLVYDIEYVPRDDSFRTTKKVCSQLEAGVQAIFGPTDALLASHVQSIC
EAYDIPHIEGRIDLEYNSKEFSINLYPSHTLLTLAYRDIMVYLNWTKVAIIYEEDYGLFNLM
HSSTETKAEMYIRQASPDSYRQVLRAIRQKEIYKIIVDTNPSHIKSFFRSILQLQMNDHRYH
YMFTTFDLETYDLEDFRYNSVNITAFRLVDVDSKRYLEVINQMQKLQHNGLDTINGSPYIQ
TESALMFDSVYAFANGLHFLNLDNHQNFYIKNLSCTSDQTWNDGISLYNQINAAITDGLTG
TVQFVEGRRNIFKLDILKLKQEKIQKVGYWHPDDGVNISDPTAFYDSNIANITLVVMTREE
RPYVMVKEDKNLTGNLRFEGFCIDLLKAIATQVGFQYKIELVPDNMYGVYIPETNSWNGI
VQELMERRADLAVASMTINYARESVIDFTKPFMNLGIGILFKVPTSQPTRLFSFMNPLAIEI
WLYVLAAYILVSFALFVMARFSPYEWKNPHPCYKETDIVENQFSISNSFWFITGTFLRQGS
GLNPKATSTRIVGGCWFFFCLIIISSYTANLAAFLTVERMISPIESASDLAEQTEISYGTLEGG
STMTFFRDSKIGIYQKMWRYMENRKTAVFVKTYEDGIKRVMEGSYAFLMESTMLDYAVQ
RDCNLTQIGGLLDSKGYGIATPKGSPWRDKISLAILELQEKGIIQILYDKWWKNTGDVCNR
DDKSKESKANALGVENIGGVFVVLLCGLALAVVVAIFEFCWNSRKNLNTENQSLCSEMA
EELRFAMHCHGSKSRHRPRKRSCLNCSSVPTYVPSNVSTSNVGVYYNYFN-
>DmelGlu-R1
MHSRLKFLAYLHFICASSIFWPEFSSAQQQQQTVSLTEKIPLGAIFEQGTDDVQSAFKYAML
NHNLNVSSRRFELQAYVDVINTADAFKLSRLICNQFSRGVYSMLGAVSPDSFDTLHSYSNT
FQMPFVTPWFPEKVLAPSSGLLDFAISMRPDYHQAIIDTIQYYGWQSIIYLYDSHDGLLRLQ
QIYQELKPGNETFRVQMVKRIANVTMAIEFLHTLEDLGRFSKKRIVLDCPAEMAKEIIVQH
VRDIKLGRRTYHYLLSGLVMDNHWPSDVVEFGAINITGFRIVDSNRRAVRDFHDSRKRLE
PSGQSQSQNAGGPNSLPAISAQAALMYDAVFVLVEAFNRILRKKPDQFRSNHLQRRSHGG
SSSSSATGTNESSALLDCNTSKGWVTPWEQGEKISRVLRKVEIDGLSGEIRFDEDGRRINYT
LHVVEMSVNSTLQQVAEWRDDAGLLPLHSHNYASSSRSASASTGDYDRNHTYIVSSLLEE
PYLSLKQYTYGESLVGNDRFEGYCKDLADMLAAQLGIKYEIRLVQDGNYGAENQYAPGG
WDGMVGELIRKEADIAISAMTITAERERVIDFSKPFMTLGISIMIKKPVKQTPGVFSFLNPLS
QEIWISVILSYVGVSFVLYFVTRFPPYEWRIVRRPQADSTAQQPPGIIGGATLSEPQAHVPPV
PPNEFTMLNSFWYSLAAFMQQGCDITPPSIAGRIAAAVWWFFTIILISSYTANLAAFLTVER
MVAPIKTPEDLTMQTDVNYGTLLYGSTWEFFRRSQIGLHNKMWEYMNANQHHSVHTYD
EGIRRVRQSKGKYALLVESPKNEYVNARPPCDTMKVGRNIDTKGFGVATPIGSPLRKRLNE
AVLTLKENGELLRIRNKWWFDKTECNLDQETSTPNELSLSNVAGIYYILIGGLLLAVIVAIM
EFFCRNKTPQLKSPGSNGSAGGVPGMLASSTYQRDSLSDAIMHSQAKLAMQASSEYDER
LVGVELASNVRYQYSM-
>DmelGlu-R1B
MRFGLKLSCLWPSFLLWLTWSSGGGGGSGVGVSAQPSLTEKIPLGAIFEQGTDEVQSAFK
YAMLNHNLNVSSRRFELQAYVDVINTADAFKLSRLICNQFSRGVYSMLGAVSPDSFDTLH
SYSNTFQMPFVTPWFPEKVLTPSSGFLDFALSMRPDYHQAIIDTIQFYGWRKIIYLYDSHDG
LLRLQQIYQGLRPGNESFQVELVKRISNVSMAIEFLHTLEQIGRFENKHIVLDCPTEMAKQI
LIQHVRDLRLGRRTYHYLLSGLVMDDRWESEIIEFGAINITGFRIVDTNRRLVREFYDSWK
RLDPQMSVGAGRESISAQAALMYDAVFVLVEAFNKILRKKPDQFRNNVQRRSQTLMVAQ
AAASTSSDGYNYSASGGGGGNGGAGGGFAGSDSGGSGGMASRALDCNTAKGWVNAWE
HGDKISRYLRKVEIEGLTGDIKFNDDGRRVNYTLHVVEMTVNSAMVKVAEWNDDAGLQP
LNAKYVRLRPHVEFEKNRTYIVTTVLEEPYIMLKQVAFGEKLHGNNRFEGYCKDLADLLA

KELGINYELRLVKDGNYGSEKSSAHGGWDGMVGELVRKEADIAIAAMTITAERERVIDFS
KPFMSLGISIMIKKPVKQTPGVFSFMNPLSQEIWVSVIFSYIGVSIVLFFVSRFSPHEWRLVQ
QQPQQSQSPDPHAHHEQLANQQPPGIIGGAPLPAPPGPPTPGAQTAAGAAALQAALSAGSP
GSGGSSSAVVNEFSVWNSFWFSLAAFMQQGCDLSPRSVSGRIAAASWFFFTLILISSYTAN
LAAFLTVERMVTPINSPEDLAMQTEVQYGTLLHGSTWDFFRRSQIGLHNKMWEYMNSRK
HVFVPTYDEGIKRVRNSKGKYALLVESPKNEYVNAREPCDTMKVGRNLDTKGFGIATPLG
SALKDPINLAVLTLKENGELIKLRNKWWYEKAECSTHKDGETSHSELSLSNVAGIFYILIGG
LLVSVFVAILEYCFRSRDSRSASSGSGMGLGMGLGGGMSGGSLGKANGSMMLGPSSAVP
GGMPSSHQRSTLTDTMHAKAKLTIQASRDYDNGRVGYLNCASLQYYPPAQLSATPPDAG
DSLHMNAHGQV
>DmelNmdar2
MMPSRVKLKRGTDGPTPTPTPMPTTMRKHTPIATLNTASCQHNSTTSRRKRILTPPSGPISL
LLLTVLTLLILDTRSCQGLRLTNGGGSLSKGAAANKEQLNIGLIAPHTNFGKREYLRSINNA
VTGLTKTRGAKLTFLKDYSFEQKNIHFDMMSLTPSPTAILSTLCKEFLRVNVSAILYMMNN
EQFGHSTASAQYFLQLAGYLGIPVISWNADNSGLERRASQSTLQLQLAPSIEHQSAAMLSI
LERYKWHQFSVVTSQIAGHDDFVQAVRERVAEMQEHFKFTILNSIVVTRTSDLMELVNSE
ARVMLLYATQTEAITILRAAEEMKLTGENYVWVVSQSVIEKKDAHSQFPVGMLGVHFDTS
SAALMNEISNAIKIYSYGVEAYLTDPANRDRRLTTQSLSCEDEGRGRWDNGEIFFKYLRNV
SIEGDLNKPNIEFTADGDLRSAELKIMNLRPSANNKNLVWEEIGVWKSWETQKLDIRDIA
WPGNSHAPPQGVPEKFHLKITFLEEAPYINLSPADPVSGKCLMDRGVLCRVAADHEMAAD
IDVGQAHRNESFYQCCSGFCIDLLEKFAEELGFTYELVRVEDGKWGTLENGKWNGLIADL
VNRKTDMVLTSLMINTEREAVVDFSEPFMETGIAIVVAKRTGIISPTAFLEPFDTASWMLVG
IVAIQAATFMIFLFEWLSPSGYDMKLYLQNTNVTPYRFSLFRTYWLVWAVLFQAAVHVDSP
RGFTSRFMTNVWALFAVVFLAIYTANLAAFMITREEFHEFSGLNDSRLVHPFSHKPSFKFG
TIPYSHTDSTIHKYFNVMHNYMRQYNKTSVADGVAAVLNGNLDSFIYDGTVLDYLVAQD
EDCRLMTVGSWYAMTGYGLAFSRNSKYVQMFNKRLLEFRANGDLERLRRYWMTGTCR
PGKQEHKSSDPLALEQFLSAFLLLMAGILLAALLLLLEHVYFKYIRKRLAKKDGGHCCALI
SLSMGKSLTFRGAVFEATEILKKHRCNDPICDTHLWKVKHELDMSRLRVRQLEKVMDKH
GIKAPQLRLASSSDLLNHHHLKERPPLLGNLSLAASAQDLYRWSYKTEIAEMETVL-
>DmelNmdar1
MAMAEFVFCRPLFGLAIVLLVAPIDAAQRHTASDNPSTYNIGGVLSNSDSEEHFSTTIKHLN
FDQQYVPRKVTYYDKTIRMDKNPIKTVFNVCDKLIENRVYAVVVSHEQTSGDLSPAAVSY
TSGFYSIPVIGISSRDAAFSDKNIHVSFLRTVPPYYHQADVWLEMLSHFAYTKVIIIHSSDTD
GRAILGRFQTTSQTYYDDVDVRATVELIVEFEPKLESFTEHLIDMKTAQSRVYLMYASTED
AQVIFRDAGEYNMTGEGHVWIVTEQALFSNNTPDGVLGLQLEHAHSDKGHIRDSVYVLA
SAIKEMISNETIAEAPKDCGDSAVNWESGKRLFQYLKSRNITGETGQVAFDDNGDRIYAGY
DVINIREQQKKHVVGKFSYDSMRAKMRMRINDSEIIWPGKQRRKPEGIMIPTHLRLLTIEE
KPFVYVRRMGDDEFRCEPDERPCPLFNNSDATANEFCCRGYCIDLLIELSKRINFTYDLALS
PDGQFGHYILRNNTGAMTLRKEWTGLIGELVNERADMIVAPLTINPERAEYIEFSKPFKYQ
GITILEKKPSRSSTLVSFLQPFSNTLWILVMVSVHVVALVLYLLDRFSPFGRFKLSHSDSNEE
KALNLSSAVWFAWGVLLNSGIGEGTPRSFSARVLGMVWAGFAMIIVASYTANLAAFLVLE
RPKTKLSGINDARLRNTMENLTCATVKGSSVDMYFRRQVELSNMYRTMEANNYATAEQA
IQDVKKGKLMAFIWDSSRLEYEASKDCELVTAGELFGRSGYGIGLQKGSPWTDAVTLAIL
EFHESGFMEKLDKQWIFHGHVQQNCELFEKTPNTLGLKNMAGVFILVGVGIAGGVGLIIIE

VIYKKHQVKKQKRLDIARHAADKWRGTIEKRKTIRASLAMQRQYNVGLNSTHAPGTISL
AVDKRRYPRLGQRLGPERAWPGDAADVLRIRRPYELGNPGQSPKVMAANQPGMPMPML
GKTRPQQSVLPPRYSPGYTSDVSHLVV-
>DmelIR8a
MELPLLVLLLALRFAGSEVLKITFWIEPVQRAEFDTDIAMVLKELDALRLDVKVDDTTLTL
TRSEDGLDMQRFCEILSTVGASAVIDLTYSHWEEGYNLVRSLGIGYVRLERIMRPFLDMFG
DFMRQKRANNVAMVFMNARDAVEAMQQMLVGYPFRTLIMDASQTDPGQHFLERIRSLR
PAPTYIALFARAAAMNGIFEKVQKADLFQRPLEWHFVFLDTRDRVFKYRRQAELCTRFTL
NPRAICRSMPMPDLYCGSGFTMQRAMLLNVLRSLINAAQVSPGYPLAIYQDCNATASSSE
VSDPLEKDDYNWLDMVHWSNFLAYAPPLPHIQDQFQSPVPGLTFAVNISAGYYSSEHEAK
TDLAAWSSVGEMRLLNETISPARRFFRIGTAESIPWSYLRREEGTGELIRDRSGLPIWEGYCI
DFIIRLSQKLNFEFEIVAPEVGHMGELNELGEWDGVVGDLVRGETDFAIAALKMYSEREEV
IDFLPPYYEQTGISIAIRKPVRRTSLFKFMTVLRLEVWLSIVAALVGTAIMIWFMDKYSPYSS
RNNRQAYPYACREFTLRESFWFALTSFTPQGGGEAPKAISGRMLVAAYWLFVVLMLATFT
ANLAAFLTVERMQTPVQSLEQLARQSRINYTVVKDSDTHQYFVNMKFAEDTLYRMWKE
LALNASKDFKKFRIWDYPIKEQYGHILLAINSSQPVADAKEGFANVDAHENADYAFIHDSA
EIKYEITRNCNLTEVGEVFAEQPYAVAVQQGSHLGDELSYAILELQKDRFFEELKAKYWNQ
SNLPNCPLSEDQEGITLESLGGVFIATLFGLVLAMMTLGMEVLYYKKKQNALEITQVRPVN
DSSGSGGNSSTAPPTATSTTKQAWHIPVLEAEEKPAKVSPPPSFETATFRGKKLPARITLGDG
KFKPRHGLYARRNLGASDSHSGYME-
>DmelIR25a
MILMNPKTSKILWLLGFLSLLSSFSLEIAAQTTQNINVLFINEVDNEPAAKAVEVVLTYLKK
NIRYGLSVQLDSIEANKSDAKVLLEAICNKYATSIEKKQTPHLILDTTKSGIASETVKSFTQA
LGLPTISASYGQQGDLRQWRDLDEAKQKYLLQVMPPADIIPEAIRSIVIHMNITNAAILYDD
SFVMDHKYKSLLQNIQTRHVITAIAKDGKREREEQIEKLRNLDINNFFILGTLQSIRMVLES
VKPAYFERNFAWHAITQNEGEISSQRDNATIMFMKPMAYTQYRDRLGLLRTTYNLNEEPQ
LSSAFYFDLALRSFLTIKEMLQSGAWPKDMEYLNCDDFQGGNTPQRNLDLRDYFTKITEP
TSYGTFDLVTQSTQPFNGHSFMKFEMDINVLQIRGGSSVNSKSIGKWISGLNSELIVKDEE
QMKNLTADTVYRIFTVVQAPFIMRDETAPKGYKGYCIDLINEIAAIVHFDYTIQEVEDGKF
GNMDENGQWNGIVKKLMDKQADIGLGSMSVMAEREIVIDFTVPYYDLVGITIMMQRPSS
PSSLFKFLTVLETNVWLCILAAYFFTSFLMWIFDRWSPYSYQNNREKYKDDEEKREFNLKE
CLWFCMTSLTPQGGGEAPKNLSGRLVAATWWLFGFIIIASYTANLAAFLTVSRLDTPVESL
DDLAKQYKILYAPLNGSSAMTYFERMSNIEQMFYEIWKDLSLNDSLTAVERSKLAVWDYP
VSDKYTKMWQAMQEAKLPATLDEAVARVRNSTAATGFAFLGDATDIRYLQLTNCDLQVV
GEEFSRKPYAIAVQQGSHLKDQFNNAILTLLNKRQLEKLKEKWWKNDEALAKCDKPEDQ
SDGISIQNIGGVFIVIFVGIGMACITLVFEYWWYRYRKNPRIIDVAEANAERSNAADHPGKL
VDGVILGHSGEKFEKSKAALRPRFNQYPATFKPRF-
>DmelIR21a
MSYYWVALVLFTAQAFSIEGDRSASYQEKCISRRLINHYQLNKEIFGVGMCDGNNENEFR
QKRRIVPTFQGNPRPRGELLASKFHVNSYNFEQTNSLVGLVNKIAQEYLNKCPPVIYYDSF
VEKSDGLILENLFKTIPITFYHGEINADYEAKNKRFTSHIDCNCKSYILFLSDPLMTRKILGP
QTESRVVLVSRSTQWRLRDFLSSELSSNIVNLLVIGESLMADPMRERPYVLYTHKLYADGL
GSNTPVVLTSWIKGALSRPHINLFPSKFQFGFAGHRFQISAANQPPFIFRIRTLDSSGMGQLR
WDGVEFRLLTMISKRLNFSIDITETPTRSNTRGVVDTIQEQIIERTVDIGMSGIYITQERLMD

SAMSVGHSPDCAAFITLASKALPKYRAIMGPFQWPVWVALICVYLGGIFPIVFTDRLTLSH
LMGNWGEVENMFWYVFGMFTNAFSFTGKYSWSNTRKNSTRLLIGAYWLFTIIITSCYTGS
IIAFVTLPAFPDTVDSVLDLLGLFFRVGTLNNGGWETWFQNSTHIPTSRLYKKMEFVGSVD
EGIGNVTQSFFWNYAFLGSKAQLEYLVQSNFSDENISRRSALHLSEECFALFQIGFLFPRES
VYKIKIDSMILLAQQSGLIAKINNEVSWVMQRSSSGRLLQASSSNSLREIIQEERQLTTADT
EGMFLLMALGYFLGATALVSEIVGGITNKCRQIIKRSRKSAASSWSSASSGSMLRTNAEQL
SHDKRKANRREAAEVAQKMSFGMRELNLTRATLREIYGSYGAPETDHGQLDIVHTEFPNS
SAKLNNIEDEESREALESLQRLDEFMDQMDNDGNPSSHTFRIDN-
>DmelIR31a
MNLLISMFILILAAGEGEIIPSMEESVVTNFVKSLVKTKQAIVFSCLFKDFKEISLALMRINQ
FVSVVNLNQSYSLTSILTRENYARTSVMVNARCSGSSELLFEASENRYFNKTYQWFLWGV
DLEVQSLFPLNLNYVGPNAQITYVNETADGYAYWDIHSKGRHLKSNLEINLIATLINDTLNI
ARDIFHLQSIDFRGQFNGLTLRGASVIDKEDIISNEQIESILSRPTKDAGVAAFIKYHYELLG
LLRERFNFTVNFRNSRGWAGRLGNTTFRLGLLGIVMRNEADIAASGAFNRINRFAEFDTIH
QSWKFETAFLYRYTSDLDTHGKSGNFLSPFSDRVWLFCLLTLGAFSIIWVLFEIIDYKILRIR
VNSQKLEHLNQKSSVICIKTTCIERILQTFGACCQQGLDPNPVDRSVRFLVMTLFLFSLVM
YNYYTSSVVGGLLSSSDQGPSTVDEITASPLKISFEDIGYYKVLFRESQNRSITRLIEKKLSS
SRSLNELPIFSHIEDAVPYLKAGGFAFHCEVVDAYPVISEYFDANEICDLREVSGLMEVEIL
NWILHKNSQYTEIFKTAMCNAQEKGFVERILRRRQIKKPACQSLYTVYPVSLSGVLPGFVI
LICKSINKFS-
>DmelIR40a
MHKFLALGLLPYLLGLLNSTRLTFIGNDESDTAIALTQIVRGLQQSSLAILALPSLALSDGV
CQKERNVYLDDFLQRLHRSNYKSVVFSQTELFFQHIEENLQGANECISLILDEPNQLLNSL
HDRHLGHRLSLFIFYWGARWPPSSRVIRFREPLRVVVVTRPRKKAFRIYYNQARPCSDSQL
QLVNWYDGDNLGLQRIPLLPTALSVYANFKGRTFRVPVFHSPPWFWVTYCNNSFEEDEEF
NSLDSIEKRKVRVTGGRDHRLLMLLSKHMNFRFKYIEAPGRTQGSMRSEDGKDSNDSFTG
GIGLLQSGQQADFFLGDVGLSWERRKAIEFSFFTLADSGAFATHAPRRLNEALAIMRPFKQ
DIWPHLILTIIFSGPIFYGIIALPYIWRRRWANSDVEHLGELYIHMTYLKEITPRLLKLKPRTV
LSAHQMPHQLFQKCIWFTLRLFLKQSCNELHNGYRAKFLTIVYWIAATYVLADVYSAQLT
SQFARPAREPPINTLQRLQAAMIHDGYRLYVEKESSSLEMLENGTELFRQLYALMRQQVIN
DPQGFFIDSVEAGIKLIAEGGEDKAVLGGRETLFFNVQQYGSNNFQLSQKLYTRYSAVAVQI
GCPFLGSLNNVLMQLFESGILDKMTAAEYAKQYQEVEATRIYKGSVQAKNSEAYSRTESY
DSTVISPLNLRMLQGAFIALGVGSLAAAALNNTINVRSLNSRDKFICGGPVKIWYYLVLLL
WYYFNRGLVGIYQLWHKTSIRNTGKGMPFLGE-
>DmelIR64a
MHWWLLVFLPLSCQGLPEHELLELELDYGLAEPQRTSLLQSSLILQFSQDYKHIPRITYFTC
QKPHLQTPNQIPNAAEHRDAFAAKNFQLIKSLYESELFVRIVLLDVLAQSPTSGRPNRPGN
GPTGGFSQTPSQAQSNSEWLEGVLRMEALRQIAVVDLACGAVSRRFLELASAKMLYSEKF
HWLLIEDFAWHGRTQTAEGSGKRDDGEMEEEPPGQQIQATDDEDLPSIESFLGGMNLYM
NTELTLAKRMSEAAHYTLFDVWNPGLNYGGHVNLTEIGSFTPTEGIQLHTWFRTTSTVRR
RMDMQHARVRCMVVVTNKNMTGTLMYYLTHTMSGHIDTMNRFNFNLLMAVRDMFNW
TFVLSRTTSWGYVKNGRFDGMIGALIRNETDIGGAPIFYWLERHKWIDVAGRSWSSRPCFI
FRHPRSTQKDRIVFLQPFTNDVWILIVGCGVLTVFILWFLTTIEWKLVPHDGSALIKPKGGA
PPRHHYQQQQQQEQVEAPVRPITAVSVVVSKEKVEEKQEEYEDSTPIDAGTLWQRCYQKL

NKYIKDRKAKQKKAPERVGLFLESVLFFVGIICQQGLGFSTSFVSGRCIVITSLLFSFCIYQF
YSASIVGTLLMEKPKTIKTLSDLVHSSLKVGMEDILYNRDYFLHTKDPVSMELYAKKITSV
PTTKENEADEDEPVDPNPVSTDPAKSYRDIVHSHETGAHAKDNAASNWLDPETGLLRVK
HERFAFHVDVAAAYKIIAETFSEQDICDLTEVSMFPPQKTVSIMQKNSPMRKVISYGLRRVT
ETGILTYHFNVWHSRKPPCVKKIETSDLHVDMDTVSSALLILLFSYAITLMILGTEILYSKW
HNRIQLKWVGAT-
>DmelIR75a
MQLVQLANFVLDNLVQSRIGFIVLFHCWQSDESLKFAQQFMKPIHPILVYHQFVQMRGVL
NWSHLELSYMGHTQPTLAIYVDIKCDQTQDLLEEASREQIYNQHYHWLLVGNQSKLEFY
DLFGLFNISIDADVSYVKEQIQDNNDSVAYAVHDVYNNGKIIGGQLNVTGSHEMSCDPFVC
RRTRHLSSLQKRSKYGNREQLTDVVLRVATVVTQRPLTLSDDELIRFLSQENDTHIDSLARF
GFHLTLILRDLLHCKMKFIFSDSWSKSDVVGGSVGAVVDQTADLTATPSLATEGRLKYLSA
IIETGFFRSVCIFRTPHNAGLRGDVFLQPFSPLVWYLFGGVLSLIGVLLWITFYMECKRMQK
RWRLDYLPSLLSTFLISFGAACIQSSSLIPRSAGGRLIYFALFLISFIMYNYYTSVVVSSLLSS
PVKSKIKTMRQLAESSLTVGLEPLPFTKSYLNYSRLPEIHLFIKRKIESQTQNPELWLPAEQG
VLRVRDNPGYVYVFETSSGYAYVERYFTAQEICDLNEVLFRPEQLFYTHLHRNSTYKELFR
LRFLRILETGVYRKQRSYWVHMKLHCVAQNFVITVGMEYVAPLLLMLICADILVVVILLV
ELAWKRFFTRHLTFHP-
>DmelIR75b
MNFSVLESHFKEAQIFVDADVTYVTHDPFSKNFLLYDVYNKGRQLGGELNITADREIFCN
KTNCRVERYLSELYTRSALQHRKSFTGLTMRATAVVTALPLNVSIKEIFDFMNSKYRIQLDT
YARLGYQARQPLRDMLDCKFKYIFRDRWSDGNATGGMIGDLILDKADLAIAPFIYSFDRA
LFLQPITKFSVFREICMFRNPRSVSAGLSATEFLQPFSGGVWLTFALLLLLAGCLLWVTFILE
RRKQWKPSLLTSCLLSFGAGCIQGAWLTPRSMGGRMAFFALMVTSYLMYNYYTSIVVSK
LLGQPIKSNIRTLQQLADSNLDVGIEPTVYTRIYVETSEEPDVRDLYRKKVLGSKRSPDKIW
IPTEAGVLSVRDQEGFVYITGVATGYEFVRKHFLAHQICELNEIPLRDASHTHTVLAKRSPY
AELIKLSELRMLETGVHFKHERSWMETKLHCYQHNHTVAVGLEYAAPLFIILLGAIILCMG
ILGLEVIWHRHCTLH-
>DmelIR75c
MTSWPLYRLIVFNLLEINLSNLMVFHCWSIKEAFPLVEMLNQNGIFSQYIDVQNPDNLANV
HKEYLDSDLVSLNADVTYVSREDEERFILHDVYNKGSHLGGKLNITVDQTLQCNRSHCQ
VKEYLSELHLRPRLQHRMDLSSVTFRLAALVSVLPINSSEEELLEFLNSDRDSHMDSISRIG
NRLIMHTQEILGFNVQDAFGGAIGMLTNESAELCTTPFVPSWNRLHYLHPMTEQAQFRAV
CMFRTPHNAGIKAAVFLEPFMPSVWFAFAGLLIFAGVLLWMIFHLERHWMQRCLDFIPSLL
SSCLISFGAACIQGSYLMPKSAGGRLAFIAVMLTSFLMYNYYTSIVVSTLLGSPVRSNIRTIQ
QLADSSLDVGFDTVPFTKTYLVSSPRPDIRSLYKQKVESKRDPNSVWLSPEEGVIRVRDQP
GFVYTSEASFMYHFVEKHYLPREISDLNEIILRPESAVYGMVHLNSTYRQLLTQLQVRMLE
TGITSKQSRFFSKTKLHTFSNSFVIQVGMEYAAPLFISLLVAYFLALLILILEICWARYAKKKF
STIIPQNQ-
>DmelIR75d
MKVQVAHWLPLIFFLLVSGTPRVAGSWRSEYSRQDPDPKTRWGNQLPDMLVAYYRHHGV
HSLMLVVCHTDIADFRLWKLWQHFNLNNFYVQVSTESSLRDLQHVDALDEHKDAPPPKS
FHANNSTHWETSFLLPALPYKMGILLLEFSSECALNLLRWSAASEHNYFTTNRFWLLLTED
PGDIDLLEDPEIFIPPDSELRVLHYENVGNFSCSLIDLYKVAAWKPLKRTLVGHNIRNSRHVI

HALQHFGSAITYRQDLEGIVFNSAIVIAFPDLFTNIEDLSLRHIDTISKVNHRLMLELANRL
NMSYNTYQTVNYGWRQPNGSFDGLMGRFQRYELDLAQLAIFMRLDRIALVDFVAETYRV
RAGIMFRQPPLSAVANIFAMPFENDVWVSILMLLIITTVVLVLELFFSPHNHDMSYMDTLN
FVWGAMCQQGFYVEVRNRSARIIVFTTFVAALFLFTSFSANIVALLQSPSDAIQSLSDLGQS
PLEIGVQDTQYNKIYFTESTDPVTKNLYHKKIASKGENIYMRPLLGMEKMRTGLFAYQVE
LQAGYQIVSDTFSEPEKCGLMELEPFQLPMLAIPTRKNFPYKELIRRQLRWQREVSLVNRE
ERKWIPQKPKCEGGVGGFVSIGITECRYALGIFGCGAAVSFVLFLFEFIFRHFKQVYRIIKGY
REVQR-
>DmelIR76a
MENLLVESYYFSTVLSFFAQQFFADSHATCIFWHPAFDFRLETVHPMPLIIMDWHRWANRS
DQDVYDYKIKEDEFEGKGIPYNDWTLRLTVAIERSHCETFIAFQEQIPEFARYFYHASIYSI
WRSLRNRFMFVYTKEFEDKKDSYLSGYIFQDQPNILVITSQYLNSSTFEIKTNRFVGPRNFN
KNPEPVEFYILQRFDAKGTKATWETQSAMSSKMRNLKGREVVIGIFDYKPFMLLDYEKPP
LYYDRFMNTTDVTIDGTDIQLMLIFCELYNCTIQVDTSEPYDWGDIYLNASGYGLVGMILD
RRNDYGVGGMYLWYEAYEYMDMTHFLGRSGVTCLVPAPNRLISWTLLLRPFQFVLWMC
VMLCLLLESLALGITRRWEHSSVAAGNSWISSLRFGCISTLKLFVNQSTNYVTSSYALRTVL
VASYMIDIILTTVYSGGLAAILTLPTLEEAADSRQRLFDHKLIWTGTSQAWITTIDERSADPV
LLGLMEHYRVYDANLISAFSHTEQMGFVVERLQFGHLGNTELIENDALKRLKLMVDDIYF
AFTVAFVPRLWPHLNAYNDFILAWHSSGFDKFWEWKIAAEYMNAHRQNRIVASEKTNLDI
GPVKLGIDNFIGLILLWCFGMICSLLTFLGELWRGQG-
>DmelIR76b
MATGIELLVAAALCVACPPLNDSPPTNLIQMGENGTLSPVTELPMDVDASEAGFDADAPV
ETLETINRKKPKLREMLDWIGGKHLRIATLEDFPLSYTEVLENGTRVGHGVSFQIIDFLKKK
FNFTYEVVVPQDNIIGSPSDFDRSLIEMVNSSTVDLAAAFIPSLSDQRSFVYYSTTTLDEGE
WIMVMQRPRESASGSGLLAPFEFWVWILILVSLLAVGPIIYALIILRNRLTGDGQQTPYSLG
HCAWFVYGALMKQGSTLSPIADSTRLLFATWWIFITILTSFYTANLTAFLTLSKFTLPYNTV
NDILTKNKHFVSMRGGGVEYAIRTTNESLSMLNRMIQNNYAVFSDETNDTYNLQNYVEKN
GYVFVRDRPAINIMLYRDYLYRKTVSFSDEKVHCPFAMAKEPFLKKKRTFAYPIGSNLSQL
FDPELLHLVESGIVKHLSKRNLPSAEICPQDLGGTERQLRNGDLMMTYYIMLAGFATALAV
FSTELMFRYVNSRQEANKWARHGIGRTPNGQSVAPSRWLRGWRRLNSGHGQLLGASTHG
QNVTPPPPYQSIFNGGSHGDPLNRWRRPLANGNALGNGVLLGGDSEGGVRRLINGRDYM
VFRNPNGQSQLVPVRSPSAALFQYSYTE-
>DmelIR84a
MIKLQVKVISWPLIILTAFLRVLQIESINTNFLELAAFEDFLRSEHLSHVLVVRGDDADGDW
KIECHQKLLANYRVQFYRPEMSANFEDLMFYGSPRTAVLVLNSEHVLVRRQVFGVASEAG
YFNNSLAWFILGSGRESLPVEQLIDQLLSGYRMGIDADITVALRGPDNASMLFYDVYRISR
QANTPLIIEKKGLWTHSGGYQKFGNFKNTWVIRRRNFLNVTLIGSTVLTEKPPGFGDMEY
LADDKQLQQLDPMQRKTYQLFQLVERMFNLSLAISLTDKWGELLDNGSWSGVMGQVTS
READFAVCPIRFVLDRQPYVQYSAVLHTQNIHFLFRHPRRSHIKNIFFEPLSNQVWWCVLA
LVTGSTILLLFHVRLERMLSNMENRFSFVWFTMLETYLQQGPANEIFRLFSTRLLISLSCIFS
FMLMQFYGAFIVGSLLSESARSIVNLQALYDSNLAIGMENISYNFPIFTNTSNQLVRDVYV
KKICKSGEHNIMSLQQGAERIIQGRFAFHTAIDRMYRLLLELQMDEAEFCDLQEVMFNLP
YDSGSVMPKGSPWREHLAHALLHFRATGLLQYNDKKWMVRRPDCSLFKTSQAEVDLEH
FAPALFALALAMVASALVFLLELFLHWLPDFRRRLGTMST-

>DmelIR92a
MLLQPLVMHLSQLLRIIVGQYFAEFPSILIVYNNSASTTPLQLEYLSALELVLRELSKPIRLQ
WINVAFLKDLNDLEDQVMGALNSSVTEGFITILSQTHHFIHARYYATRNANVRLKDKRYL
FLCEDESPAELLCMDILQFYPHHLMVRPGTETAPTGPTGPHPDPRRGGGASVSTKNKDDG
EGGAGNKTTSPYRDINFELWTQKFVGAVGNLDALLLDAFLPNETFANRVELYPNKLLNLQ
RRSLLVGSITYVPYTITNYVPAGQGDVDPIHPQWPNRSLTFDGAEANVMKTFCQVHNCHL
RVEAYGADNWGGIYDNESSDGMLGDIYEQRVEMAIGCIYNWYDGITETSHTIARSSVTIL
GPAPAPLPSWRTNIMPFNNRAWLVLISTLVICGTFLYFMKYVSYRLRYSGTQVKFHHSRKL
EKSMLDIFALFIQQPSAPLSFDRFAPRFFLATILCATITLENIYSGQLKSMLTFPFYSAPVDTIE
KWAQSGWKWSAPSIIWVHTVQSSDLETEQILARNFEVHDYSYLSNVSFMPNYGFGIERLS
SGSLSVGDYVSTEALENRIVLHDDLYFDYTRAVSIRGWILMPELNKHIRTCQETGLYFHWE
LEFIDKYMDKKKQEVLMDLANGHKVKGAPQALDVRNIAGALFVLAFGVAFAGCALVAE
LLIHRMDLSK-
>DmelIR93a
MNPGEMRPSACLLLLAGLQLSILVPTEANDFSSFLSANASLAVVVDHEYMTVHGENILAH
FEKILSDVIRENLRNGGINVKYFSWNAVRLKKDFLAAITVTDCENTWNFYKNTQETSILLI
AITDSDCPRLPLNRALMTVECRINAVVFVDQTILEENALLVKSIVHESITNHITPISLILYEIN
DSLRGQQKRVALRQALSQFAPKKHEEMRQQFLVISAFHEDIIEIAETLNMFHVGNQWMIFV
LDMVARDFDAGTVTINLDEGANIAFALNETDPNCQDSLNCTISEISLALVNAISKITVEEESI
YGEISDEEWEAIRFTKQEKQAEILEYMKEFLKTNAKCSSCARWRVETAITWGKSQENRKF
RSTPQRDAKNRNFEFINIGYWTPVLGFVCQELAFPHIEHHFRNITMDILTVHNPPWQILTKN
SNGVIVEHKGIVMEIVKELSRALNFSYYLHEASAWKEEDSLSTSAGGNESDELVGSMTFRI
PYRVVEMVQGNQFFIAAVAATVEDPDQKPFNYTQPISVQKYSFITRKPDEVSRIYLFTAPFT
VETWFCLMGIILLTAPTLYAINRLAPLKEMRIVGLSTVKSCFWYIFGALLQQGGMYLPTAD
SGRLVVGFWWIVVIVLVTTYCGNLVAFLTFPKFQPGVDYLNQLEDHKDIVQYGLRNGTFF
ERYVQSTTREDFKHYLERAKIYGSAQEEDIEAVKRGERINIDWRINLQLIVQRHFEREKEC
HFALGRESFVDEQIAMIVPAQSAYLHLVNRHIKSMFRMGFIERWHQMNLPSAGKCNGKSA
QRQVTNHKVNMDDMQGCFLVLLLGFTLALLIVCGEFWYRRFRASRKRRQFTN-
>DmelIR7a
MFHHLWLLMGLRSLAMGALHPPQPEAMTPLVAAALEILAEQVSPSQSTLAVMDLTQDAE
HRDERQEQLMTIILRSVGSEMALRTFQKPPAEVPASFVVFLVNSAQAFNTLGFHFTDIHSTR
EFNFLILLTHRMSSRAERLQVLRDISRTCVRFHTSNVILLTEKRDGVVLVYAYRLLNMDCD
LSVNLELIDIYKNGLFRHGHEARSFNRVLSLSGCPLQVSWYPLPPFVSFIGNSSDPEERAQI
WRLTGIDGELIKLLASIFDFRILLEEPCNKCLSPDIKDDCSGCFDQVIISNSSILIGAMSGSHQ
HRSHFSFTSSYHQSSLVFIMHMSSQFGAVAQLAVPFTVIVWLALVVSSLLLVLVLWMRNRL
VCGRSDLASHALQVLTTLMGNPLEARSLPRSSRLRILYAGWLLLVLVLRVVYQGKLFDSFR
LPYHKPLPTEISELIRSNYTLINQEYLDYYPRELTVLTRNGSKDRFDYIQGLGKEGKFTTTSL
IATMEYYNMMHWSTSRLTHIKEHIFLYQMVIYLRRHSLLKFAFDRKIKQLLSAGIIGYFVRE
FDACQYRKPFEEDYEVTPIPLDSFCGLYYISLIWLSAAVVAFILELLSQRIVWLRRIFE-
>DmelIR7b
MKYWLYILSCCSLVASTMESSSDWDLAEALAQVVANSEMGRFKTLYIYTHTNSQSTGGHL
EELLDQVLMIVPNNLQARRLLLQQSMEYKPYVHAVLALVDGLPSLSAIYARIRATQDLSHT
LIYMSMPTDAYGEEMQATLRFLWRLSVLNVGVVLRPPGDHILMVSYFPFSALHGCQVISA
NVVNRYQVGTKRWASQDYFPSKLGNFYGCLLTCATWEDMPYLVWRPDGSGSFVGIEGAL

LQFMAENLNFTVGLYWMNKEEVLATFDESGRIFDEIFGHHADFSLGGFHFKPSAGSEIPYS
QSTYYFMSHIMLVTNLQSAYSAYEKLSFPFTPLLWRAIGLVLILACLLLMLLVRWRHHHEL
PRNPYYELLVLTMGGNLEDRWVPQRFPSRLVLLTWLFATLVLRSGYQSGMYQLLRQDTQR
NPPQTISEVLAQHFTIQLAEVNEARILASLPELRPEQLVYLEGSELQSFPALAQQSGSSARVA
ILTPYEYFGYFRKVHPMSRRLHLVRERIYTQQLAFYVRRHSHLVGVLNKQIQHAHTHGFL
EHWTRQYVSAVDEKDESVARIASTSYSTLDGIDGDPSLSESEEDQQVAPVRQNVLSMREL
AALFWLILWANLGAVVVFVLELLLPRIKLRKILRKMKKSTRASATTTSTLSSPSTTKDIPFS
CKDGFQDSWPKCSLLVS-
>DmelIR7c
MLHSAVHNVSLVYALVWAIDNYYGMATSTPLAVVQFPTSRESRRLHNDLIDAALGRSSGT
GRIQFLLEDDRVEMTETDTDPPPPSGLTGRPIAIWFLDSLRSYFRLEMYLNQLGSPYKRNG
FFLVIYTGLEDQPMESLKIMFRRLLNMYVLNVNVFLQRDGTVHLYTYYPYGPHHCQSSLP
VYYTAFQDLAAPANGFGLTKPLFPRKLTNMHGCEMVVATFEHRPYVIIEDDPKTPGGRSIH
GIEGLIFRSLAERMNFTIKLVEQKDKNRGEILPDGNFTGILKMMVDGEVNLTFVCFMYSKA
RSDLMLPSTSYTSFPIVLVVPSGGSISPMGRLTRPFRYIIWSCILVSLIFGFVLICLLKITALPGL
RNLVLGRRNRLPFMGMWASLLGGLALYNPQRNFARYILVMWLLQTLILRAAYTGQLYLL
LQDVEMRSPIKSLSEVLAKDYEFRILPALRTIFKDSMPTTNFHAVLSLEESLYRLRDEDDPGI
TVALLQPTVNQFDFRSGPNKRHLTVLPDPLMTAPLTFYMRPHSYFKRRIDRLIMAMMSSGI
VARYRKMYMDRIKRVSKRRNLEPKPLSIWRLSGIFVCCAGLYLVALIVFILEILTTNHRRLR
RAFNVINRYAA-
>DmelIR7d
MDIRCVVALLLGLCKVQAVVWPHQHLLEEQLASQISATLQKIFINGLAVYNFGVFISTSYE
EMDRDRVILVHQVLNRNLYPPNFPVAVVLASKMNRKITAQVFTQLLFVQNAEQAIAIAEG
VNRNGLCVIVLLTSQPERPIMTKIFTYFMQERYNINVVILVPRLHGVQAFNVRPYTPTSCSS
LEPVEIDIKDGDLWDVFPRRLKNLHGCPLSVIVWDIPPYMRINWKSSDPMDGLDGLDGLL
LRIVARKMNFTLKLIPNEPNGLIGGSSFMNGTFTGAYKMLRERRANITIGCAACTPERSTFL
EATSPYSQMSYIIVLQARGGYSIYEVMLFPFEKYTWLLLSTILGLHWIVGSRWRMPSPILA
GWMLWIFVIRASYEASVFNFIQNSPVKPSPRTLDQALSGGFRFITDHASYRMTLKIPSFQGK
TLISAGQPVDVFDALLKAPWKTGAFTSRAFLADHLVRHRKHRNQLVILAEKIVDNMLCM
YFPHGSYFAWEINKLLFNMRSFGIFQHHSQILAWDNLPTTTDTDTPGKRIHSSTESVATGFA
ESMSFVVAALNCLMGALCISIVVFGLELLSRRRHWTGLEWLFERV-
>DmelIR7e
MNHINEFVARAVLHVVHHYILSVTPSLVLTLCCRSNHTCNFYNKMMSTLFREWGLAPLQI
VNVLRGVPWHPVPGRRHFNVIFTDSFAAFEEIRMEYYSREYNYNEHYFIFLQARDRLLQG
EMRLIFDYCWRYRLIHCSIQVQKSNGDILFYSYYPFGEHGCSDMEPQLINRYNGSMLVEPD
LFPRKLRNFFGCPLRCALWDVPPFLTLDEDQEEVLRVNGGYEGRLLLALAEKMNFTIAVR
KVHVNMRDEALEMLRRDEVDLTLGGIRQTVARGMVATSSHNYHQTREVFGVLASSYELS
SFDILFYPYRLQIWMGILGVVALSALIQLIVGRMLRERMGSRFWLNLELVFVGMPLLECPR
SHTARLYCVMLMMYTLIIRTIYQGLLYHLIRTHQLNRWPQTIESLVQKNFTVVLTPIVQEVL
DEIPSVQHMRFRLLEANSELDPLYFLEANHQLRQHVTASALDIFIHFNRLSADKVHQRGEQ
GSGAHFEIVPEDIISMQLTMYLAKHSFLIDQLNEEIMWMRSVGLLSVWSRWELSESYLRNE
QSFQVLGTMELYAIFLMVLVGLIVGLLVFILELVSMRSIYLRKLFT-
>DmelIR7f
MQGEDANLYVARALRLVIENVLAQLSTTLVVTISTRHLGTAHWFEYMMNILMDSWRMVA

VQLLRIRPDLVVNPVPGRKRVSLLMVDSYQGLLDTNITASNANFDDPDYYFIFLQARDHLI
PKELQLILDHCLAHFWLHCNVMIQTAQVEVLVYTYYPYTADACQKAYPIPVNTFDGRKW
KASQMFPDKLSQMHGCPLTVLTWHQPPFVELVWDPKHNRSRGSGFEIQLVEHLARRMNF
SLELVNIALLRPNAYRLAEGSSEGPIEKLLQRNVNISMGYFRKTARRNQLLTTPMSYYSAN
LVAVLQLERYRIGSLALLVFPFELSVWMLLLLALLIHLGIHLPSARRGNEEDGGGGLQVVA
LLLGAALARLPRSWRHRFIAAHWLWASIPLRISYQSLLFHLIRLQLYNTPSFSLDQLLAEGF
QGICTANTQRLLLEMPQLARDPDSIQSVDTPFDWDVLNVLTRNRNRKIFAVANQDVTLSFL
HSSAHPNAFHVVKQPVNVEYAGMYMPKHSFLYEKMDDDIRRLDASGFIHAWRRASFASV
HRKEQVHMTSRRYINHAKLSGIYMVMAGLYLLAGLLFAGEVLLRQRN-
>DmelIR7g
MNVTSLLNFESMKYIGAQTQAASINHHVAQALRVFIEDFYQRIAPAFIVVLSCRRPSPMNF
YRNIMQLLYESVDTMIVQLVLVELGRPRRIAGPRTHNLLLVDSLDALLDIEIHTYTAQSDTS
EYYFIFLQQRDALIPHDMQGVFAYCWRHQLINCNVMTQSSGGQVLLHTYFPYAPGQCND
SQPTRINMFLGESWKHRDYFPSKLHNLNGCPLIVLARKVSPFLDLDEGQRELRGLEGRLL
QELSRRMNFSIQFSGLQDQLKNRTTWTEKQLLQKLVQERIAHLAIGYVRKRIQYATNLTPV
FPHYSNRVVGCLLLNAHNLTSLEIWSFPFQALTWICLVAGDRLALVLAVYAASLGLPIDPPE
RPSLQLLFASWLIFGLIVRSMYSALLFFILRYHLHQRLPGNLQDLTHGDYAAVMGRTTLQD
LREVPSLQDLLGLKSVIVTSEREEEVLRTLDRCTLREGAGSHPLFFGLISQDALLHLTQRGH
RAGAYHIIPQDVLEQQLAIYLQKHSHLASHLDHLVMSIRSVGLVHHWAGQMASERYFRSR
FLYREKRIRQPDLWAVYILTAGLYLLSLVVFICELLASRRAGL-
>DmelIR10a
MAVLGTVFLLFMLDLKTLNLTRLNGLLVEPTRDLPQLELWLRAGSDHQDAENPYVQWFL
LRTEIPLSIVTYQENRYWMDDPFGRRNLVLVMSLDQLLTNRGAAAPIQKASTFFYILADQD
KDLSADEQLRLEGSCRQLWTQHKVYNRFFLTRDGVWIYDPFKRRDSAFGRLVRYYGSET
LDKLLFRDMAGYPLRIQMFRSVYTRPEFDKETGLLTRVTGVDFLVAQMLRERLNFTMLLQ
QPEKKYFGERSANGSYNGAIGSIIKDGLDICLTGFFVKDYLVQQYMDFTVAVYDDELCIYV
PKASRIPQSILPIFAVGYDIWLGFVLTAFACALIWLTLRVINLKLRIVSLGNQHIVGQALGIM
VDTWVVWVRLNLSHLPASYAERMFIGTLCLVSVIFGAIFESSLATVYIHPLYYKDINTMQE
LDESGLKVVYKYSSMADDLFFSETSPXWNRDLRADVIDEVARFRNKAGVSRYTSLILESS
HFTLLRKIWVVPECPKYYTISYVMPRDSPWEDAVNALLLRFLNAGLIVKWIQDEKSWVDI
KMRSNILEADAESELVRVLTIGDLQLAFYVVIGGNLLAFLGFLAEHFRWKLQKKGV-
>DmelIR11a
MRFAILWLFSGCLLPGIQVGIWVVVRAQPTGRDVLLSRLGNQQNELNTRRLANASSYLTR
NYIANRINTLVVREICVECPYELSERQRQLVDQILASLAPELSVLLHKGTAEETTWEYTLFV
VNDHTAFTGQVFIFPDELLEREFFCIVVVSEIQSRQFVRQTVGSIVKSNLQMHFVNVVVVA
QLEDGTVGTYSYKLFKANCTPGITVRQINHFDRITGKPQQSMPDLYPVRNGHLGDCPFNV
GAAHMPPHLIYKRHKDPPPASNVSIPAEDLAGIDWDLLQLLAKALKFRIQLYMPQEPSQIF
GEGNVSGCFRQLADGTVSIAIGGLSGSDKRRSLFSKSTVYHQSNFVMVVRRDRYLGRLGP
LILPFRGKLWGVIIVILLLAVLSTCWLRSRLGLSHPIEDLLTVIVGNPIPDHRLPGKGFLRYLL
ASWMLLTLVLRCAYQARLFDVLRLSRHRPLPKDLSGLIKDNYTMVANGYHDFYPLELTCR
QPLDFSARFERVQRAAPDERLTTIALISNLAYWNHKHPNISRLTFVRQPIYMYHLVIYFPRR
FFLRPAIDRKIKQLLSAGVMAHIERRYMQYENKRKVASNDPVLLRRITKSIMNGAYRIHGL
VIVLATGMFILELLAGRSNGRLRRWMEWVHQ-
>DmelIR20a

MLASLNRSTGLSAELLDLYGLVVHFLLSGEHTTLVYFNPAGLDCSWGVLWQRNLTAHPQI
VWQRNYSYPDLYYQFNAKLLVLACLPMDSRAAIQLEILANSLSHLRTVVRLLIEVAGPDQ
VTLARQYLSFCLRRSMLHVELYFRDYHHSLILYSFRAFPSFELVMRWISVGQGVKLFLHKL
DDLRGHRLRVIPDLSPPNTFFYRDARGDNQVTGYLWDFLATFAGRLNAGLEVVRPSWRA
GSASDSSYMLEYSAKGLIDVGLTTTLITKWNLWAIHQYTYPLLVSSWCTMLPVEKPLATPD
LFGRIVCPTLAMTLLLIILVTWLVFRQLRCLTRLKNSRPARIVPHLLTLLLLTTCSAQLLSLLI
FPPYHVRIASFEDLLRGDQKILGMRNEFYNFDGAFRARYAGVFYLIDDPNELYDLRNHFNT
TWAYTMPYIKWLVIKTQQRHFSKPLFRWSKDLCFFDFMPTSVIVAPDSIYWESIKDFTFRIH
QAGLMKHWIRKSFYDMIKAGKMSIKDYSDLETLKPLNIGDLEIVWRVCGAAIAVASAIFIM
ELLYFYINVFFNSL-
>DmelIR41a
MFIDLSWSLVLSAIVGKYLNESTICIFWNDKFEFQLLHKSDYISFVGINIKSFDDNGGHYIID
TGLKKKELQNKHLFLDELVIKIIISIEVTHCETFVVFDKDIDRFVNAFNKASVYSIWRSLHN
KFVFAHIANESPESRNHFFEDQPNILFVVRDHSSASSFDIKTNKFVGRKAENPSQMILVDRY
LASEQRFQFGKSLFADKLNNLQGREVIIAGFDYPPYTVIKHNMSTNAQDMGVSGESDFKN
VYIDGTETRIVLNFCEQFNCTIQIDSSAANDWGKVYPNMSGDGALGMLINRKADICIGAM
YSWYEDYTYLDLSMYLVRSGITCLVPAPLRLTSWYLPLEPFKETLWAAILLCLCAEATGLV
LAYKSEQALYVLPGYREGWWTCTSFGVCTTFKLFISQSGNSKAYSLTVRVLLFACFLNDLII
TSIYGGGLASILTIPSMDEAADTVTRLRFHRLQWAANSEAWVSAIRASDEALVKDILYNFHI
YSDDELLRLAQDQHMRIGFTVERLPFGHFAIGNYLGPQAIDQLVIMKDDIYFQYTVAFVPR
LWPLLDKLNTLIYSWHSSGFDKYWEYRVVADNLNLKIQQQVQETMTGTKDIGPVPLGMS
NFAGFIIVWILGSAIATLTFLLELSLTYILKQSNLK-
>DmelIR47a
MRQIKLLVWLLVVGVVSSTEQLQFLKNFLEAVHKERSISTILLIQRKVHKNDFLHGLYPIF
WPIICLDETKRVELVNNFNKDFLALVYMESEADTLLLSALAADLNHIRDARIMIWLQMSPS
ENFLDRIVFQASKQKFLNLVVIENTLKTRRFYPFPQPKVQVIDKPFEEKEIYPALWRNFMG
KNAIAVPDLVPPRSFNSFDPKTGHRRESGSIYNVFKAFTQRYNITMLLKWPLIRNTTQEEIIG
KSVRGEIDLPITGQLISFRHPNGSRSQPLLGMTALSIAVPCGPELPMFDRFFLFYGLATPITIT
GYYVLLNTIEIILGTLSDRIKRHPRRKKILNLVLNLRVFSCILSLPTPQGNRLRSVKGQLTMV
MSITGLILSCIVAAQTSTILTMKPQYRHIKNFQELSDSNITVVCNHLNYLTIKQQMDPKFMA
KFMQNIWIVNSIEQMKMIFDLNTSYAYQTFSYKKDPFTLLQMHTTRKAFCRTPGLDLVSG
LAYTAVLEKNSIYALALQDYTLKAFSAGLVYYYWAEESIRDLISTVGRTQFEKLPIVIGYQSL
KLQDYNVCWKILLIGGALAFCVFIVEVVVGLINRRI-
>DmelIR47b
MREAQIIIFLLTSAAAVTLKQYEFLXSFLKAGEQEQTITTLLMMQKHVHTKNLLQGLYPXP
WPIIHFVETQRIKFIALLYMSSEKDIFLSSLAANLKFERLDKPFGKSNIFPVLWRNYMGXIAL
TLDHLVEPRSFYWTDPRTNIKRRTGYIYMLITNFAEQHNITLQLXSPPNEDMSQMVIIERTH
KGPRSTHNWADDQLETFERXQDSLLPWHGSMAIVVPCGQEMSAYERFHAAHAFRAPIIFF
GFHIFLSLIDFLLRTISDRIRCNPRRIQLLQTVLSLCVLRCILSTSLPNSNXLRSRLRDNSPXX
XVLQAXSYSALWXLTGTAXQXHNRDFQSHKLHDYXTTDGSXHSIEVPGLLKARNXXIXL
FHIFSSLGTKFDLRIGSAGSHTSGVEFRYYELLDRXSSLENNIVSQVFTILKLPYSRFRVLKL
EDCRGCWQTLFVGFSIATFVFIVNVLMGFFRNINQKK-
>DmelIR48a
MHLLITETYMIIGKTLHDILNELNERLIISTNIIFCKQFDNLIHFEAQTSRFVYSSLEAFNITSL

WNHVGNDNKLFVIVGNVPPYELFAKLELSSPENCTQFILNNTVDMCADALVKNSKAFSVS
RELRIAPANVIVPHGKPLLSYRYLAAPFNTKVWIALGTYVFLISGFLCLIHWLRSGKWDFS
QNLLEVYSSLLFTVFHLKATNGIERYILFGVLFISGFVYSTSYLRLLKSMLIAETFEKQIQTF
EELAESNIPLLINPYDRMIFQHHHIPKSLWTAVRTVSSETLLNHRSHGYVRLCPAILTASKIPS
HTHRHLFSVCRFSHEQEVVPKGSSXXSLVPCIRKRNREXNHLGCLSGVSWPGISXFFHYG
ALGGEAFGSILLHDANYFPSPRLFRRLAELHYGSY-
>DmelIR48b
MILQQSSNLLKLLLLLAISSVRTQGLNDIIIELNQRLLISNNFLYCNQSDKLNEYEIKYLQHM
PPISLMIFTSIESMNFTQVEYNLGADNKLFLIMGNEEPPYDFLHALNLHFQFAEYIIVIDEPV
DLKKSTKWLDFVNHLWQQGYVQLLIYTSYDEKLYHKIIFPETVIEETLVEQYISIRGSFNNL
YGYPVRVAAYNNAPRSMLYVNRWGKHIFAGFYMRFLRAFIDARNGSFVPVLTPSNSPGNC
TLNLVNETVDVCADALAANPAAFSLTHGFRIASANVLVTHAKPLHSYRYLTAPFQWSVWA
CLVIYVLLVVNFLSFIGWLRSGKWEFSKYLLEVFSSLLFSGFYLKEIRGRERYILFGVLFIAG
FVYSTEYLGLLKSMLISEVFEKQIDTFEALVESNITLMVDPYDKILFAKYNMPEILSPIMELV
SFETLLKHRNRFDQDYAYILFSDRMALYDYAQQFLKHPKLLRIPIDFSFLYTGIPMRKRWFL
KHHLGRAWYWAFESGLTRKLALDADFEAVRVGYLSFLITEHVEAQPLNVDYFVMPAIALA
IGYILALLSFVIEMTAWRIREFLGCRKATMTSTGCSEGGHVDVD-
>DmelIR48c
MSLLRIILIIIFLRIVSSIPDTIISHLSAELQIKIQIYFGLGNDLYDFSRLDGNYQKIIISHNISEEF
KTYHDEPVLIIIRLERDLNLNLATLDVLRSYLTDRQYNDILLIDNDEENLNSYVDIRKAYW
NAGFSQVLIYNSQQRTWSIKPYPYLQIRPTSLKEYIENRNTRNLMGYPLRVLVTNDPPHCF
VDKDELPGSPNRYKGSIVTMLKIFADQLNATFQANPFREFRRYSTADCVQMVSDDEIDAC
GSIFIRTYTYATSQPVRLNRVVIMAPFGNPIEKFYYFFRPFDLYVWIGTGIIVVYIAVMGSLL
HRWHFKEWNVGQYLLLAVQTLLNRELSLPQSSSGSKFMLLLLLFAIGFILSNLYVALLSMM
LTTKLYQRPIENLADLKAANVNILLQTHNIRPNSVYGSSEELRERFLLVEESQHLEKRNGLD
PSYAYVDSEDRMDFYLYQQKFLRRRRMKKLSNPVGYTWAVQVIKQNWVLEKHYNDHVQ
RFFETGLQNKLVDDVHELAVKAGFLHFFPTQTQTIEPLRLEDIVMAAMVLGGGHALAVIC
FLVELFA-
>DmelIR51a
MYNVLVLFLLLFTRAQMEPHRRGHNMTLLRSVLTVIRGRENWKNTPIFLGGHCNSDDLN
NLMSWLQNTMEVTCHTVDTSTSAKNENALGHFNINADNSLGLLFCQSSHELIWFNMDKR
LRRLRGIRLIVILSDKRSSSSKAIMSTFKRLWHFQFQXNFQGYVVSTPVENDIPRVFFVKDK
KTGRKQIRGFGYRTFVEYLHRYNASLHVSNSQQEHAINSSVNMGRIINQIVDGQLEISLHP
YVDVPENMGDNSYPLLIASNCLIVPVRNEISRYMYLLLPLNQSSWILLLGSVIYISGVLYYI
QPGLLHRTWDQRIGLNILDSISRIINICSPSRIYNPSLRYFIVSVHLSILGFVVTNLYSIMLGSF
FTTLVVGEQVDSMQQLIQXQQKVLVKYYEVSTFLRHVEPDLVDGVAQLLVGVNASEQVS
ALLGFNRSYAYPFTLERWEFFSLQQQYAFKPIFRFSSACLGSPIIGYPMKSDCHLQSSLNMFI
MRIQAAGLLRHWVVSDFNDAMRAGYVRLLENFLGFHSLDVDSLRLRWAVLLCGWLLST
LIFLCER-
>DmelIR51b
MCKVLTLLVVILLLALTNAAYNVTLLKSVLSLISTREPWINTPIFVGHNTQGGDLNDLIIWL
HQTMGVTSLTMNLFLQPEHIRPLGHFKITRYNGIALFFCHDKHDIMWLTLDRNLRKLRRIR
LIIILRNQRSGSQGAIKSIFNALWQYQFLNVLVLQRDQLYSYTPYPAMRFFKLDIHTEPLFPH
AARNFHGYVVSTPAENDIPRVFHVHDPLTKSRKVLGYAYRTFVEYLDHYNASLRLTNPDE

NLDPTTSVNMNHIVQLIIDGQLEISLHPYVFTPPTATKSYPLLIYPNCLIVPMRNEIPRHMYL
LRPFQLYSWYILLFAVFYITGILYCISPKLNKSSWPQRLGLNFLDAISKILFISPPITIYRPTWR
HLIIFLQLSVLGFMSTSWYNIELDSFFTTIVVGEQVNSMDQLVHQQQRVLVKEYEINTFLR
HVEPRLVEKVSRLLVPVNASEQVSALLSFNRSFAYPFTEERWQFFAMQQQYAFKPIFRFSSA
CLGSPHIGYPMRVDSHLETSLNHFILKIQDTGLLNHWVVSDFNDAMRAGYVRFVDNVLG
YQSIDVDTLRLGWCVLGIGWILSALVFSCEYWHLYPWRFIA-
>DmelIR52a
MALGWSVIILGFIGQLSAQILNYTQSRDLELLEGSLFRVLSRLNLEEEYNTLLIYGKECVFH
SLLRKLEISAVTVPSGSTDYDWSFSTAILILSCGYDAENEENSYTLMKLQRTRRLIYLEDNS
EPESVCMRYSLKEQHNIAMVKSDFDQSDTFYSCRLFQTPNYVEGHFFKDQPIYIENFQNM
RGATIRTVADSLVPRTILYRDEKSGETKMMGYLGHMINTYAQKLNAKLHFIDTSKLGAKK
PSVLDIMNWVNEDIVDIGTALASSLQFKNMDSVWYPYLLTGYCLMVPVPAKMPYNLVYS
MIVDPLVLSIIFVMLCLFSVLIIYTQHLSWKNLTLANILLNDKSLRGLLGQSFPFPPNPSKHL
KLIIFVLCFASVMITTMYEAYLQSYFTQPPSEPYIRSFRDIGNSSLKMAISRLEVNVLTSLNN
SHFREISEDHLLIFDDLSEYLVLRDSFNTSFIFPVSVDRWNGYEEQQKLFAEPAFYLATNLCF
NQFMLFSPPLRRYLPHRHLFEDHMMRQHEFGLVTFWKSQSFIEMVRLGLASMEDLSRKR
NEEVSLLLDDISWILKLYLGAMFISSFCFILEILRCGERCKRLWRCRW-
>DmelIR52b
MTWLVILLCFLGYMAAHIADISVQNQSLMDNELINLLLKLRNEEFYDTLLVYGKDCEFHS
VIKNVDVAVVLVSDSMNFEWNFSSLTLILSCGPDIDNGGPNSTSIKLQRNRRLVLLKEDFQP
SNICNIYTQKEQYNIALVRENFTKSKSIYTCRYFQDPNVDEVNLSGTKPIFIEQFQNMKGKA
IRIVPDLLPPRVMLYQDANDGELKMIGYVANLITNFAQKVNATLQLDFLKPSTSITEISRMA
KDDELDMGITLEASLNTSNLETSSYPYLLTSYCLMVQVPAKFPYNLVYALIVDPLVLGIIFV
LFLLLSVLLIYSQKMSWQDLSVANILLNDKSLRGLLGQSFPFPLNASKKLRLIFTILCFASIM
LTTMYEAYLQSFFTNPPSEPEICSFQDVGSYNRRIAMSALEVNGLIKTNNSHFREIRMDDLE
IFDNMPECYELRDAFNLSYNYVVTGDRWRSYAEQQTLFKEPVFYFARDLCFSRLIFLSVPL
RRHLPYRHLFDEHMMQQHEFGFVNYWMSHSFFDMVRLGLTSLKDLSRPLAYTPSLLMD
DISWIMKIYLAAIVLCVFCFLLEIGVDKWKRWMKFRNLQILNTC-
>DmelIR52c
MVWLIIILFCLGNSSSQILDVTNNSHLDFDYRLFGLLQRLQVEKSYDTLLVYGEDCAIPSLF
ERLQVPAVLVSSGSTNFDWNFSSLTLILSCNFQDEREENYRTLMKLQTSRRLILLKGHIKPE
SVCDFYSKKEQHNVAMVKENFYQLEVVYSCRLFQDQNYEKLNLFDGKSIYKDQFRNMH
GAPIRTLSDKEPPRTIPYIDSKTGEEKFKGYVGMLISQFVKKVNATMQIREDLIKDDEEVSF
VDITNFTSNDILDIGICEARTLEMSNYDAISYPYLMSSYCFMAPLPDSLPFSDVYMAIVAPSI
LIMFLIIFCICSVLIIYIQERSYRSLTIRSVLMNDICLRGFLAQPFPFPRQYNRKLKLIFMLVCF
SSLISTTMYTAYLQAFLWGPPIEPRLTSFDDVKKSRYTMAINIYEREFLEALNVSLEDVEIYD
YGKFSKLRSTFNTNYLFPVTALQWFTINEEQKLFKYKIFYYCDAFCLNQFDILSIPLRRHLP
YRDIFEEHMLLQKEFGLTKYWIDQSYRDMIRANLTTFKDFSPLLENDYIEVHNLYWVFTM
YFVGMGMGLCFFILEILRPLRYWRNCKIKCEYCYAFLKNFAK-
>DmelIR52d
MVRIIIILLCLGYTKARILDATNTNHTDLEERLLSLLLRLQQEQFFNTLLIYGEDCAFSSLSR
RLQVPTILVSSGSTSFEWNYSSLALILTCEFKAEREENYQTLKKLQMNRRLILLNGNIKPDS
VCDFYSKKDQYNIAMVNNNFHQVGIIYACRLFQERNYEKVYLSEGNPIYVDQFRNMQGA
LLKSITFNLIPGSMAYRDPKTGQEKHIGYVANLLNNFVEKVNATLDMQVKLHKAGKKTSF

YNITKWASEDLVDIGMSYAAYFEMTNFDTISYPYLMTSTCFMVPLPDMMPNSEIYMGIVD
PPVLVVLIAIFCIFSVMLNYIKQRSWRSLSLVNVLLNDICLRGFLAQPFPFPRQSNRKLKLIS
MLVCFFSVITTTMYTSYLQSFMWGPPIDPKMCSFADLENSRYKLAIRRYDIEMLRPFNVSM
DHVVVFDESSQLEYLRDSFDDNYMYPMSALSWSAFKEQQKLFAFPLFYYSEKLCLKPISF
FSFPIRRHLPYRDLFEEHMLQQNEFGLSTYWIDRSFSDMVRLKLATMNDFSPPRLEDYIEV
SDLSWVFGMYFTGLGISCCCFGLELLGLPSWTRRLRLTNWLRVRN-
>DmelIR54a
MWTVITGIVLWAPVLVAGSAVDFIFRAAAEHSLSVIMIRIDYCPYNWAKDIFENQTIPVVVL
SDSETFINIRMFSRPLHVACLPGHELQKDLALLENFTSSLMDFPSQKKIVYISNNFSDPTRM
DYIFETCYHRRIWNIVGLLASDEHRYFYRYHLYPSFRTEYRSLESSTIFDKDFPNMHGHPLT
VMPDQWLPRSVLYVDRRTGKQILAGSVGRFFHVLSWKLNATLQLSKKVTTGRFLNATAL
KELSESFSVDVPASLTIMERVEQLASTSYPMEVTHVCLMVPVARRIPIKDIYFILSSASNMFL
AIVIVSSYGLALNLLRNMTHRDVRLVDFVLNDKALRGILGQSFNLPLSRSFSTRLIFLMLGI
VGLNVSSIFGAGLDTLMAHPPRQFQARSFAGLRRTKIPLVTTEEDFPTWMKLRVPMLVVN
VSEYNHLRNGRNTSNAYFASRLYWNLFSEQQKRFTRELFIYSTDDCLWSLALLSFQWPQN
SLFTEPVSQLILEVNANGLYDFWVGMHYYDMTAAGLSGLEDPSLQLKEREHPTSLRIVDF
QWMWQAYGTFMVIAILVFLLEVSWHRITSLFVSLVY-
>DmelIR56a
MGSRFFIRNLILFGLLASSNMQIPFGELEKKFELDVDFLLGVTELVGHIQGLYSITVYADCID
IHPSIQQRIMDKFMVPVNTIGSNLSRPNYHKLDNSRIRIVLFTGLNDTILVNLNKTDVPYSD
NFYMLAYASAIKNKCIELDFIEEVFTLLWKMSIQNAILLIRGEFMMEMWSYLYMGKIHKIK
LTKPNSYLESLRKYNYRFSLEVINDPPAIFWYNSSEQADVTGGGNLSVSGPLGLIIINFLRHL
NVTIDIVPIPGKQTSQYELFQQPDNLRAENGVNMVGSALLKYSPMVTQSRMCLLVSNRR
MIPFSRFLDRLVSPGVHKLTFVSSIGIFVIKYFSHRPRSFVDAIFCTIRFFFAIPLPSIILNRLPV
VDRFIEVFIIIFVQILLSSNISITTSALTTGFWEPPIINVETMRASGLHILTEDPTILQAFKENILP
SSLADLVILVDEDTYFHHVTTLNNSYVYVVQAHNWQIFRLYQQQMTNEPFEIASEELCSK
WRILGIPLNPKSPLRFMFKDYFYRILESGLREQWVHSGFKKFCEFNNLKKLPVDSVDSWQ
PLSIEFYSNVIRAYIIGLVIATLAFVAELLHNGYRRKNVKKT-
>DmelIR56b
MLLDTDLASGVIRSPYSFDIPHAFIFNETQFVVPKFCGPYMEIVKHFAEVYHYQLFLDSLES
LPKKSVVEQDIISGKYNLSLHGVIIRPEETSDFFNATQHSYPLELMTNCVMVPLAPELPKW
MYMVWPLGKYIWTCLFLGTFYVALLLRYVHWREPGNATRSYTRNVLHAMALLMFSAN
MNMSVKLKHASIRVIIFYTLLYIFGFILTNYHLSHMTAFDMKPVFLRPIDTWSDLIHSRLRIV
IHDSLLEELRWLPVEYQALLASPSRSYAYVVTQDAWLFFNRQQKVLIQPYFHLSKVCFGG
LFNALPMASNASFADSLNKFILNVWQAGLWNYWEELAFRYAEQAGYAKVFLDTYPVEPL
NLEFFTTAWIVLSAGIPISSLAFCLELFIHRRKQRRPQYERFECYDY-
>DmelIR56c
MRSSFRICLFLLTTYHPSHGWNMQHLLNLLAPFGRMNVFQEIVWFVSPHQRLDQLDEFIM
RIDEAFGKSATQTVVNNNTEMRMIYSSARRNHMSFVFTTGAEDPIMKVFSKVLLGRHFYV
SMVIYVDKVGDMHPIYDLLTFAYNQQFFNSMVHFESMEGVNQLFGVSKFPVMSFENRTD
FLKYMGKIWKQVQNARSDVGGFGFTTPLRQDLPHLFQSQGHYDGSTYRIIETFVRFINGSF
KELIMPPDSLGGQVINMKDALQLIRERKMEFCAHAYALFMSDEELEKSYPLLVVQWCLM
VPLYNSVSTYFYPLQPFDWNVWFFALGALLALVLLELMWLRMFGGWSGYRGAVLNSFC
YIINVPIEGQLQQPCLLRFLLLATVFFHGFFLSAYYTSNLGSILTVNLFHAQINTMNDIVSAQ

LPVMIIDYEMEFLLNLNKELPQEFLELLRPVDSAVFSEHQTSFNSSFAYFVTEDHWEFLDEQ
QKHLKQRLFKLSSICFGSYHLAFPLQMDSSLWRDIEYFTFRIHSSGLLNFYARSSFGSALHA
GLVQRMPDTQEYTSAGLQHLAIAFILLLVMSFLAGIVFVLETLSR-
>DmelIR56d
MDNRAAELILRERNIFPTNGSDNITLLNNMFVLEMFYRITQLYHFKNFIFYISERLDLNNKD
SQEFFHNFWTYFPMAPNLIITREHHLGIPMMQFISTPSLVMVFTTGKDDPIMELASHNQQGI
HWLKTIFVLFPSLQSRDFETNPESLAQFTAEIKDVYDWVWRKQFINTFLITIKDNVFILDPY
PTPSIVNKTGVWQAEEFFHKYAKNMKGYLVRTPILYDMPRVFKSDRPTNRYEKNFIHGTS
GNLFLGFLEFVNATLMDTSANVTADYLNMTNLLDLVSQGVYETLIHSFTEITTKFVVSYSY
PIGINDCCIMVPYRNQSPADQYMHEALQENVWVLISLFTLYITVAIYLCSPLRPRDLSAAFL
QSICTLTYSVPTFIIRTPTLRMRYLYILLAIWGIVTSNLYISRMTSYFTTAPPVRQINTVQDVV
EANLRIKMLAIEYERMAKSPLQYPESYLNQVDLVDKHMLDLHRDPFNTSFGYTVSSDRW
RFLNLQQLHLRKPIFRLTEICEGPFYHVFPLHKDSHMRSVMTEYIMIAQQAGLMNHWERE
TFWEAVHLHRIHVHLFDDEPMALSLDFFSSLLRTWTLGLILAGLAFAAEMKWHEHVTFKR
RPVIRITRKPRSFLRRFMKL-
>DmelIR56e
ERXAFRNQWAFCFPRTXAIEVVLSAWSPXCPGQRSKPQPISXPHHXGSCWRKRKWKXKP
RLLVVDKRTLVEHLNSLNDGYAYCIIAGHWQVGMM-
>DmelIR60a
MWCNNPGLIIIIFLGQILNLCQGIVNLSNETANTVIFMLPEKDLGPDVWKAGVGCLDSFAQI
FFFRNPKERFTRAYNLMLVHAFHLSSPADQIQEGFSKLINEAVTNPGPPDREELFQMRVASD
YNITNGTEDKGELILADNYVIVVDSVDRLKELMKKKIVEMRSWNPGARFLVLFHNATCR
NRPLGVASNIFKDLMEMFYVHRVALLYANSTMNYNLLVNDYYSNVNCRILNVQSVGQCH
DGKLYPNNAVVKASMQDYVSGFSPRNCTFFACSSISAPFVEADCILGLEMRILGFMKNRLK
FDVNQTCSLESRGEMDGPANWTGLLGKVQNNECDFVFGGYYPDNEVADHFWGSDTYLQ
DAHTWYIKMADRRPAWQALVGIFEAYTWIGFILILIISWLFWFTLVMILPEPKYYQQLSLTA
INALAVTISIAVQERPICETTRLFFMALTLYGLNVVATYTSKMIATFQDPGYLHQLDELTEVV
AAGIPFGGHEESRDWFENDDDMWIFNGYNISPEFIPQSKNLEAVKWGQRCILSNRMYTMQ
SPLADVIYAFPNNVFSSPVQMIMKAGFPFLFEMNSIIRLMRDVGIFQKIDADFRYNNTYLN
RINKMRPQFPETAIVLTTEHLKGPFFILVVGSCWAALTFIGELIIHRWRTQLVSTSEQQDRRS
DKRRRRRRRRKPEKDNRWQRQVQVAPVVRFTPVKRRKVFQGQTSQK-
>DmelIR60b
MRRSLYLIIAIGLVDVHCVSLRYILNALENELQYRAILLVESASEIESCWEQKYIQGAVPILN
FNANQSLYLKDALNTNILALVCLNENVESTMQALYENLEDMRDTPTILFVLSDSKVQDVF
LECLRRKMLNVLAFKGLDRGFVYSFRAFPTFRVIERNVMDILQYFEQQLEDLGGHTLTTLP
DNIIPRTVVYKSPDGSRQLAGYLYPFLRNYVSTINATLKVCWHLVPEDGMIQLGEVVRLSE
IHDVDFPLGMHGIEHGSTSQNVPLEVSSWFLMLPMEPSLSRAQFFIMLGFEKVTPVLLLLTI
LLSTAHRIEMGLRPSWRCYVLGDRVLQGTLGQAFFLPRRLSVKLMLVYSLILLNGFTFSNY
SITSLETWLVHPPSGHPIHSWEQMRTLNLKVLIVPSELDSMTKALGKQFTESNSDLFELSKS
GNFQDKRLAMDQSYAYPVTCTLWPLLEHAQIRLPKPEFRRSREMVLIPLLIMAMPLPKNS
MFHKSLNRYRALTHQSGLYEFWFKRSFNELVALRKIHYKVNGDHQIYRDFEWQDFSYVW
LGFVGGTIASILVLLAEIGYHRWQLNQN-
>DmelIR60c
MEMRLALFFTFACLAGAHDGSLRNMLKSLEDELGYRTILLLEGFVYSFKAFPTLRVVKRR

VKDVRRYFEPQLEDLGGCVLKVVPDGIMPRTMVYQGEDGELQMGGYLSHFIRNYVSTIN
ASLQIRWDLFPEDGDFDMDSLTGSNHVDFPLGLGSLSFQTLHKDVAMEISSWFLMLPMEP
SLPRARFFIRFGISLYLIPLIILLAIVLSNAHRFEAGLTPSWRCCSMGNTVLRGVLAQAFVLP
KGLSPKLMFVYWLLLVSGFFVSNYVIVYLTAWLIQPPTSDPVTDFDQMRRAKLKILMVPT
DMDYLKSIRGAEYVDAHSDVFQTADSTDFQTQRMSMELHFAFSVTGTLWPLLRQAQVKL
HRPIFRRSKEMVFLPFVIMGMTMPNNSIFLSSLKQYRLRTSEAGLYLLWFKKSFSELVAIHK
ISYKEDWVHDSYSDLKWEDFLFAWLGFLGGTTVSCLALLAEIGYHRWLWKRTHQ-
>DmelIR60d
MRLAIYVAFLSSIGNRSGFLSSLLMSLGKELHYKTILLVGGSSTCWSLEPFETGVPILNLRG
ENNAYPQDTFNSQMLALACLQTESEDAVKLLYRSLKDMRDTPTLLFASSEEHIHDTLFLGC
FRENMLNVLALTASSKEFIYSYQAFPTFRVIKRKLVEIHRYFEPQLKDLGGHIVSALPGNIM
PRTMCYRNAEGERQLAGYLNTFIRNYVESINGTLRISWGLVPEDDMRHLTISRLSKIQHVD
FPLGIIPLYNKTDKQHVYMEISSWFLMLPMETSVPRAHLFVKLGLERLLPIIVVVGAVLGN
AHRIEVGLGPSWRCYYLADKVLRGALAQPIVLPRRLSPKLMLIYSLLLLSGFFLSNYYMA
SLTTWLVHPPASDRILEWDQLRYLHLKVLTIPEEFKYMSLILGTDFMTAYGSIFQLTNSTDF
QRRRISMDPSYAYPVTTSLWPFLELSQVRLRRPLFRRSYDMVLQPFQVMSLPLPRNSIFHK
SLLRYAALTRETGLYYYWFRRSYYELVALGKISYKEEEGNPYCDLKWNDFRIVWLAFLGG
TIISCLALLLEVAHYRWHLGNSSL-
>DmelIR60e
MVIKMISFLLVSVLLCLVGASDSESMQVQVLQDLNLALQTELNVFIDFECCATSEILHKLD
SPRILLSSNSREARDLRIRGNFTESTLIIVSVMDSDLNPLVASLLPRLLDELHELHIVFLSNEE
PGFPKQDLYTYCFKEGFVNVILMSGKGLYSYLPYPSIQPISLSNVSEYFDRARIIRNFQGFPV
RILRSTLAPRDFEYSNEQGGLVRAGYLFTAVKELTYRYNATIESVPIPDLPEYDVYLAVAEM
LHTKKIDIVCYFKDFSLEVAYTAPLSIIREYFMAPHARPISSYLYYSKPFGWTLWAVVISTVL
YGTVMLHLAARGARVEIGKCLLYSLSHILYNCHQKIRVAGWRDVAIHGILTIGGFILTNVYL
ATLSSILTSGLYDEEYNTLEDLARAPYPSLHDEYYRSQMKAKTFLPERLRRNSLSLNATLL
KAYRDGLNQSYIYILYEDRLELILMQQYLLKTPRFNMIRQAVGFTLESYCVSNSLPYLAMT
SEFMRRLQEHGISIKMKADTFRELIHQGIYTLMRDDEPPAKAFDLDYYFFAFVLXTVGLISS
LLVFFAELVSGHL-
>DmelIR60f
MRFHLNIANSGLLGLHLCPTRSALPEQNPCFSKAGAVIXNLTLPWRRWRERCLLGALRPX
TLPTPELQCXSKYLPXRKSQQENASSGLPGFCXGDXQTELHRGSRAIALPRSPYHDLYYV
WIAYLGGTMIGIGMLAVEIACFKWDLLRRPPIXMY-
>DmelIR62a
MYLQFLFALFLSRYQIVATENFDRAFELALFLDRIGRVHRLHAITIVNSLGSVDPSYLDDLH
RGLMCNSSNHFYMLPQMTATDKDSSHVHFSSLQDEETIYLVFARDSKDAVIYLQAERARG
RRYTRTMFLLRKQESQKDIKYFFELLWKLQFRSALVVVAARNFYQMDPYPTVRVIRMRRL
SSYDPHHVFPPANRKNFRGYRMRLPVQQDVPNTFWYKNRRTKAWELAGLGGILINQLM
MHLNVTMDLFRFEVNGSSLLNMAALTDLIVKGKVELSPHLYDTLQSNTSVDYSYPTQVAP
RCFMIPLDNEISRSLYVFLPFSLTMWLCLLFVLLVVHFVYVRRLIPDGHFWAILGVPGAGQ
VRYGNRKPVRRFSTFLILFGIFILGQTYSTKLTSSLTVTLIRRPDNSLEELFLLPYRILVLPTD
VYAIVDSLGHAEQFSTKFSCTDAENFSQKRISMHPEYIYPISTIRWRFFDMQQRFLRKKRFY
FSKICHGSFPYQYQLRVDSHLKDALHRFLLHVQQAGLHDLWLDTCYRKAHRMGYLKDFS
TLAELEEKLRLRPLALNLLVPAFSLFLCGMLGSGIAFLVEIRHSFGCRQKPPSINRNPGD-

>DmelIR67a
MLPILVPVLLLFNETSWINPILTSIYKDRHHETVLLLQHSQHGNASGLERFPWPVFSFNEQM
DFYVRGKYNSEMLVLIWQTGNSDWDLDLWQALDRSLLNMRKVRVLLLRKWEKIPTADV
AATAEHLLFLHVAVIGQGNRIYRLQPYAPQSWLQVDPIESPIFIKIRNYFGRYIVTLPDQFPP
RSIVYRNPKTDEIQMTGYVYKFLLEFIRIYNFTFRWQRPIVQGERMNLILLRNMTLNGTINL
AISLCGFETPSXLGVFSDVYDMEEWYIMVPRAQEISIADVYVVMVSGNFLIVLIIFYFIFTIL
DTCFGPLLLKERVDWSNLMLNERMISGIMGQSFNMSARNTISSKVTNATLFLLGLVLSTLY
AAHLKTLLTKRPTSQQISNFKQLRDSPVTVFFEEAERFYLKHAWDRPIRYIKDQLNFRETIE
YNALRMGLNRSNAFSALTSEWMIVAKRQELFKQPIFTVQPELRVIQTSVLLSLVMQSNSIY
EDHINDLIHRVQSAGIVEYWKHQTLREMITMGMISQKDPFPYVAFREFKVGDLFWIWLLW
VSFLFMSFVIFLCELLVDCFISKTLIRNKRPH-
>DmelIR67b
MELLYLNTLQSLSLLEGNRLVQTVQELNNIYQTELNVFLEFGNGADILESAQGTFVPTLWI
KNPQNQKVMKGNFTSCTLTILYLEDEHLDRGLYYLANWLWEYHHLEVLIFFNGGSYDKLI
QIFSRCFNEGFVNVLVMLPGSDELYTFMPYQDLKILNLKSIKEFYSLSRKKMDLNGYNITS
GLVIAGAPRWFSFRDRQNRLILTGYMLRMIVDFTNHFNGSVRLMNVLTVNDGLELLANRT
IDFFPFLIRPLKSFSMSNILYLENCGLIVPTSRPLPNWVYLLRPYAFDTWIAWLIMLIYCSLA
LRILSKGQISISAAFLKVLRLVMYLSGSRDMGTRPTTRRLFLFVILTTSGFILTNLYVAQLSS
NSAAGLYEKQINTWEDLDKSDSIWPLIDVDIKTMEKLIPDRTKLLKKIVPTLEADVDTYRR
NLNTSCIHSGFFDRIDFALYQQKFLRFPIFRKFPHLLYQQPLQISAAFGRPYLQLFNWFVRKI
FESGIYLKMKDDAYRHGIQSGLLNLAFRDRHLEVKSNDVEYYYLIAGLWFGGLTLATVCF
LLELLIGYAKIKVTISCKMNIM-
>DmelIR67c
MFCWLIFLNIILLSDRSESWSAREVIHQFNHDQQLQLNIYLDCNDVELQIGQEVSNLFVNS
TADKMKILGRFSSHSLIIACFKDSTRNRTLNGVKELLWGLQYLPILFVVDSNMDFYFQQAL
RHGFIHVLALNFMNGSLYTYKPYPKVEVHQIKDMQKFYKLTKLRNLQGQAVRTTVETMT
PRCFRYRNRHGQLVYAGYMYRMVKEFISTYNGTEEHVFGNVDTVPYKEGLAALKNGEID
MMPRIIHALEWYYFYRSHILYNIKTYIMVPWAEPLPKSLYFIQPFRGTVWITIMVSFVYASI
VIWWIRYRQQGNSSLTQSFMDVLQLLFQLPLSKIWHFNMGTHQVVSFIVLFVFGFMLTNL
YTAQLSSYLTTGLFKSQINTFDDLFREKRTLLVESFDAEVLHNMTKEKIIQKEFESIILITSIE
EVFKHRKSLNTSYAYEAYEDRIAFELSQQRYLRVPIFKILKEVYDQRPVFVALRHGLPYVEL
FNNYLRRIFESGIWIKLQEDSFLEGIASGEISFRKSKSREIKIFDKDFYFFAYILLGMGWCVS
TIALFLELWSFKYSVTNVLHEG-
>DmelIR68a
MRCLWILIVAFISLAMATSIPIPIANPAPLSGYEMQLKILLQKILWVANVKRCFAVITDDLHY
PIYDRIFFESVGRRVIPFFVMRTNESDDLQRPSRQVELFVKAIKSSDCELNVITILNGWQVQ
RFLGYIYDNRSLNMQKKFVLLHDLRLFESDMIHLWSVFIDAIFLKRQLDNKYTISTIAFPGI
LSGVLVMKNIANWELGKGLNGRILFADKTSNLFGTSLPVAISEHVPMVLWANATKSFQGV
EVEIMNALGKALNFKPVYYKPNQTENMDWTELDGGASVAYGSGNPDGYAQNGTHIDSM
LVDEVAAHSARFAIGDLHLFQVYLKLVELSAPHNFECLTFLTPESSTDNSWQTFILPFSAGM
WVGVLLSLFVVGTVFYAISFLNAIINGNVSSEFFRCLRPNRNVPMDPKIYRRISFRIAISRYR
SSKGDRMPRDLFDGYTNCILLTYSMLLYVALPRMPRNWPLRVLTGWYWIYCILLVATYRA
SFTAILANPAARVTIDTLEDLLRSHIPPSTGATENRQFFLEANDEVARKVGEKMEVFGYSDD
LTSRIAKGQCAYYDNEFYLRYLRVADESGSALHIMKECVLYMPVVLAMEKNSALKPRVD

ASIQHLAEGGLIAKWLKDAIEHLPAEALAQQEALMNIQKFWSSFVALLIGYVISMLTLLAE
RWHFKHIVMKHPMYDVYNPSLYYNFKRIYPQH-
>DmelIR68b
MKFLVGLLLQWYLPGIYALAEIACRIAVEQNVQVTYLYRCASCPASFDADYSALELDLYRC
VGSRLPVITRNMEAHELEPFRRTDSLSIFQIPAAEKGDSLVRRILDMLNPHQRRKHMHKYL
FVWPNAGRHQLLRLFRGSWAKKLLYGLAITGRENGTFDFDPFAWGGLQVIQRLDGEVPY
ARKVKDLRGYPLRFSMFTDPLMAMPRSPVETAGYQAVDGVAARVVGEMLNASVTYVFP
EDNESYGRCLPNGNYTGVVSDIVGGHTHFAPNSRFVLDCIWPAVEVLYPYTRRNLHLVVP
ASAIQPEYLIFVRVFRRTVWYLLLVTLLVVVLVFWVMQRLQRRIPRRGVIQFQATWYEILE
MFGKTHVGEPAGRLSSFSSMRTFLMGWILFSYVLSTIYFAKLESGFVRPSYEEQVDRVDDL
VHLDVHIYAVTTMYDAVRSALTEHQYGLLENRSRQLPLGIATSYYQPVVRRRDRRAAFIM
RDFHARDFLAITYDSQAERPAYHIAREYLRSMICTYILPRGSPFLHRLESLYSGFLEHGFFEH
WRQMDLITRVGASPDAEEFLEDLGDQTDTDSGSNELAIRNKKVVLTLDILQGAFYLWSVG
IGISCLGFAVEHAHWFWRRQTLRNAVEARTS-
>DmelIR85a
MSIQWLKHILLLAILVNLAGTRENHIPLDLKKSSIVMVKMSQILCKARIKVLFVYFENQTS
HEHTGQILKEVTKCDISNQNTPLEAVKDDGILMYMVMITTNISQPLELSLIRKKSAAKHRS
HVFLLVRDADTVSDAWMRASFRQFWKIWLLNIVILYWRDGRLNAYRYNPFMDNYLIPVD
NKPNEVPTLEQLFPKTIPNMQRKPLRMCIYKDDVRAIFWRQGTILGTDGLLAAYVAERLN
ATMMITRPHSYNNHNLSSDICFLEVAKEYVDVAMNIRFLVPDTFRKQAESTVSHTRDDLC
VIVPKAKTAPTFWNIFRSFGSLVWALILVSVLVANVFCYILKSEVGRVPMQLFAGALTMPM
TQIPPNHSIRLFLIFWLYFGLLICSAFKGNLTSMMVFQPYLPDINQLGALARSHYHIIIRPRH
VKHIQHFLTLGHKHESRIREQMLEVSDTQMYEMMRNNDIRFAYLEKYHIARFQVNSRVH
MHLGRPLFHLMNSCLVPFHAVYIVPYGSPYLGFLDSLIRSSHEFGFERYWDRIMNSAFIKS
GVKVVNRRRGSGNDEPVVLKLQHFHAVFALWLVGIGMACIVLAWEHLTHNYNLAVTKRR
D-
>DmelIR87a
MSTPEQRFWLAALLFLLSQHSEVRGFGINLMKVQTEDKGQEACILALLRKYFDSGDGLSG
SVLCINRNYQLPNIEEQLLRGVNNYENYPWSLLITNSREGPSPAKFLMNEKPQCYFLIVDN
LEDEDLDEVFEHWKGMVNWNPLAQFVVYLASLEETDEEMNDLMVELLLTFINKKIFNVN
VIGQSEENQFYYGKTVFPYHPDNNCGNRVISVELLDACDYPSEETDSEDENDEDEGDGAQ
EEDDGPQEEGDGEQEEEDGPQEQEDGDQAKGDEGQENDDGGLENKVENEFRIGASDDD
ELENDLSSNSSEPEAIIEEFFRAKFEDKFPRDLSGCPLTASFRPWEPYIFRNSEEQPVDDYYY
GLQGDEDDYNDTSPNYGESDDESYADPGEDGDGAIPDTETQSGGKLKLSGIEYEMVQTIA
ERLHVSIEMQGENSNLYHLFQQLIDGEIEMIVGGIDEDPSISQFVSSSIPYHQDELTWCVAR
AKRRHGFFNFVATFNADAGFLIGIFVVTCSLVVWLAQRVSGFQLRNLNGYFPTCLRVLGIL
LNQAIPAQDFPITLRQLFALSFLMGFFFSNTYQSFLISTLTTPRSSYQIHTLQEIYSNKMTVM
GTSEHVRHLNKDGEIFKYIREKFQMCYNLVDCLNDAAQNEHIAVAVSRQHSFYNPRIQRD
RLYCFDRRESLYVYLVTMLLPKKYHLLHQINPVIQHIIESGHMQKWARDLDMRRMIHEEIT
RVREDPFKALTFDQFRGAIAFSGGLLLVASCVFAFELCYVKYVYRTEKRERKTKKITKKVH
NIKIQHD-
>DmelIR94a
MALPKQLKFINIFLVLLIIYGSSDGTENQHEIFLNRLLQAVHNERSVETLFLLHHSNLANCS
LQDWNPPRIPTIRSNELTVFNVEKTFNHNALALVCLMKNSYREILNTLAKSFDCMRQERIIL

MIHRKSDSKFIEDITHEVKNLQFLHLIVLIVQEKYNGQVFASTLRLQSFPEPHFKRIRNVFAI
QRIFYRPINFHGKVLNAIPNDIPILFVALNEMFTEYARRYNSTLRIQNRTIKEDIEITEDNYDI
DMKIQLHNSQNFLHHMNIAMDIGSNSLIILVPCATELRGLDIFKELGVRTLTWLALLFYIIFV
LVEMLFVFISNRFNGRNFTMRYTNPLINLRAVRAILGQTSPISNRYSLSIQHFFVFMSLFGTL
FGGFFDCKLRSFLTKRPYYSQIENFSELRKSGVTVVVDHTTRQFIEQEINANFFRDEVPNVR
TTTIQELINHVYSYDRKFAFVANSIPWRTFREEMKSINQKILCDSKNLTILENVPLTFSIRRN
AIFSHHLRNFIINAADSGMITCWFKMAGKVIRKHIKTTLRESEQQPSHLPLSFDHFKWLWA
VLCIAYVMSFMVFVMEILWSKYQRRTRSVSIV-
>DmelIR94b
MSLIFNLLFILILSQAVSQETEFLQLKYLNNIVRSMIKLHKMETLVIVKHHLDNNCSLQNW
NAHGMGIIRTNDQGKLIMKDTFNSRTLAIICIGQNSHITLLRNVFETFGKVQQKKIILWTQM
ELKEKFFQEISKKSRDLKLLNLLVLKAVTKDKLLIYRLNPFPSPHFKRIENIWTPNDTLFMD
TKFNFHGMTAVVKHDYNWTIQMGNIRKFPISRIEDKEVIEFALKYNLTLQFFNDVERFDIEL
RKRIILKSNSTQPIDSGIPMVFSSLLIVVPCGNYLSIQDVIKVSGIEKWIFYIILVYVIFVLIEIT
FLGVTILISRQSRHQMIPNTLVNLCAFRAILGLPFPETRRTSLSLRQLFLAIALFGMIFSIFINC
KLSSMLTNPCPRPQVNNFEELKTSGLTVVMDHDAENFIEKEIGVDFFNQYMPRKVTLTFTE
RAKLLFSLKGNHAFTLFSESFAIIESYQRSKGLRAHCTSEDLIVAERVPRIYILENNSILDRPL
RRFIRQMQESGITNHWLKNIPSSLEKNLMQITIPYDRERVHPLSIEHLTWLWCILILGYSISM
IVFFVEMSLKRRKKNLENRAPNICIC-
>DmelIR94c
MSKVFKLLVLPLIYLSLTKGSKNPQLKFLRELINVIEEGREIRTIMVIKHSRDEYCHLDQWN
PRGSPILRTNEMGSIRISGYFNDQAVILACMGENSDYGLLKSLANAMDNMRQERIILWSER
EPTKMLMDYISQQADRYNFAQIIIVTMNEDVDAVPSLHQLNPYPTPRFRQITNISNIRRTSFF
GCGLSFQGKTAILKESVVSNIRFKVWSPSGPIPLSELKDYEIVQFAVKYNLSLKLYDQNESK
SDHFDIQLGPLFITKDFPTQMAFVSPNTACSLIVIVPCSPKWRFMDVLHKLGVLKLIGCLLI
AYAVFVLIETLILWLTHRISGREVRLTSLNQLLNPRAFRGILGLPFPEFRRSSISLRQLFLVISV
FGLVYSNFVSCTLSALLTKPAQNPQVRNFKELRDSGLITIMDKYTHSFIEKHIDPEFFDHVL
PHYLILQKKEALRMIWNFNDSYSYVMYTTTWKSLNTVQKSFDERVFCESESLTIAWNLPR
MYVLGNNSVLKWMLSRYITYMPQTGIPDSWTEQLPKVLKLLYNVTSPRRIKEGAVPLSIQ
HLSWIWHLLFIGESIATLVFIVEILLQKSNQHTSNMRERSSEDDDFV-
>DmelIR94d
MGQLHLLLVALVLLSPGGDSFYHSLIHHLNRELKIEYVLLLGNFDTTWLDILWQLPVSVLQ
IKEHSRETYSLLENPSHNVLTIAFVNDSPEDILEILYRNLRMLNTQPVLLVIRKSTIRVNSLLE
WCWHHQLLKVVAIAQDFMESLIVYSYNPFPVLQFIERRLDNSTVIFEKRLENLHGYEVPIA
LGGSSPRLIVYRDLEGKLIFSGPVGNFMKSFEQRYNCRLVQPYPFDESAISPARDLIASVQN
GSVQIALGAIYPQVPYTGYSYPIELMSWCLMMPVPEEVPHSQLYSMVFSPMAFGITIVAM
VLISLTLSMALRLHGYRVSFSEYFLHDSCLRGVLSQSFYEVLRAPALIKAMYLVICLLGLLI
TSWYNSYFSTFVTSAPRFPQLTSYESIRHSNIKIVIWKPEYEMLLFFSENMEKYSSIFQLQED
YKEFLHLRDSFDTRYGYMMPMEKWSLMKEQQRVFSSPLFSLQDDLCVFHTVPIVFPMVK
NSIFKEPFDRLILDVTATGLLSRWRDMSFTEMIKAGQLGLEDRGHPKEFRAMKVGDLIQIW
RFVGWMLGLATIVFLLELICFWRHKMWQNMKYMFCRNKNI-
>DmelIR94e
MDCPKWILSGLCLISLVSGATVIELLGTLKLELDFEYVLLMKNRNFSLSDQVWNGTSLTKD
VMDEVQVPVLQFNENVSYFLHNSISRRLVTLGFMSDANLDEHRGLLTALVANLRHMTTSR

VIFLVQSKASTDFLYELFRNCWRKKLLNVIVIFQDFETTSTFYSYSNFPILQIEERIYETSLQT
LPIFPDRLRNLHGYEMPVILGGTAPRMIAYRNKKGNVVYDGTVGHFMTAFQQKYNVKFV
QPLQAKNPLDFAPSMQTVGAVRNETVEISISLTFPTIPPFGFSYPYEQMNWCVMLPVEADV
PPFEYYTRVFELAAFLLTLGTLVLISCLLASALSLHGYATNISEFLLHDSCLRGVLGQSFVEV
FRAPTLVRGIYLEICVLGILITAWYNSYFSSYVTSAPKQPPFRTYDDILASKLKVVAWKPEY
AELVGRLLEFRKYETMFLVEPDFNRYLALRDTLDTRYGYMITTNRWVLINEQQKVFSRPL
FQKRDDFCFFNNIPFGFPLHENSVFMEPVQKLIMELAETGLYYHWITTGFSELIDAGEMHF
VDLSPHREFRAMQIQDLQYVWYGYAFMVVLSSLVWLLENLAYTVKSKTIFPTHFMQRNK
K-
>DmelIR94f
MSGMWQQVLLAETSNWFRSDVLQRFWTHLRVEIRFRTMLNYRLESCDCWFDNVLGSDN
STALLWNDQTYPHYLRRRQDTDILVVSCLRFHQYQEVLLALSLMLDQMRSMPVVLQLCG
DEDSMQELNSARLLLKHSQDLKMPNVVLLSSTFFTSATLYSYEMFPEFNVQKLVYQAYLT
LFPYKLGNLKGHPIRTVPDNSEPLTIVRKTLNGSIAIDGLVWQFMIEFAKHINATLQLPIEPH
PEKSIKLVQILDLVRNQTVDIAASLRPYSLNVQRSSTHIYGSPMMVGNWCMMLPTERVIGS
HEALTRLMKSPWTWLILLLFYSVHRFLAQKTRLRSSLIHLIKLLINLSLICFLQAQLSAYFIG
PQKVNHISNMQQVEESGLKIRGMRGEFMEYPIDMRSRYASSFLLHDLFFDLAQYRNSLNT
SYGYTVTSVKWELYKEAQRHFRRPLFRYSEEICVQKLSLFSLIQQSNCIYCYRSRIFILRMH
EAGLIRLWYRRSYYVMVTAGRFPIGDLSTVHRAQPIRWTEWQNVVLLHGVGLLFSVVVF
VIELTVHYANVCLNNL-
>DmelIR94g
MSTAVNSVHSKLVSLISRGQELTSIFFYAPAKEKCHLEDTISSATWGLPLVIWRTDRTVILNG
FIGEGLLVLACLPGFHWRALLGSLARSLKYLRQARILIELMQDRDEFLVSEVLQFCLSQDM
INVNAIFDDFPETENLSSFEAYPSFEVVNQTFTPDTQVSDLYPNKMLNLRGGVIRTMPDYSE
PNTILYQDKEGNKEILGYLWDLLEAYAHKHNAQLQVVNKYADDRPLNFIELLDAAQSGII
DVGASIQPMSMGSLSRMHEMSYPVNQASWCTMLPVERQLHVSELLTRVIPYPTLALLLLL
WIFYEVLRGRWRRHSRLQSIGWLVLATLVSSNYVGKLLNLFTDPPSLPPVNSLAALMESPV
RIISIRSEYSAIEFTQRTKYSAAFHLALHASILIGLRNAFNTSYGYTITSEKWKIYEEQQKRSS
KPVFRYSKDLCFYEMIPFGLVIPENSPHRAPLHSYTLLLRQAGLHDFWVNRGFSYMVKAG
KINFTAVGERYEAKTLTITDLRNVFIIYVSVLLISLILFTCELFVSWVNYWLGF-
>DmelIR94h
MLSNISFSSAPELVDLYGLVLKFLVSSETTLFYFNPTGQKCSWETLPRTILSNHPQIIWFREE
TYPGLYKRHSSNLFVMACLSSTSYDGQLQLLAESLTRYRSVRVLIEVQDKEGSFLASQILL
LCQQHSMLNVVLYFSRWTRTLNVFSYLAFPYFKLLKQRLSGSLRPKIFINQLKDLQGYKIR
VQPDLSPPNSFSYRDRHGECQVGGFLWRIVENFSKSLKGDTQVLYPTWAKAKVSAAEYMI
QFTRNGSSDIGVTTTMITFKHEERYRDYSYPMYDISWCTMLPVEKPLSVEILFSHVLSPGS
ALLLILAFILFFLIVPQLIKCLGITFRGRLIGMASRIFALVMLCSSSAQLLSLLMSPPLHTRIKS
FDDLLTSGLKIFGIRSELYFLDGGFRAKYASAFHLTENPNELYDNRNYFNTSWAYTITSVKW
NVIEAQQRHFAHPVFRYSTDLCFSSETPWGLLIAPESFYREPLQHFTLKINQAGLITQWMT
QSFHEMVRAGRMTIKDYSRTNLMKPLRIQDLRKCWVIFAVGLGTSTVVFTIELLLIYTNVF
LNSL-
>DmelIR100a
MATTLQLIMLALVGGTLGQANNTDHKQVLTSIVKQLEGGLELHLRTSEDGGNDLVQFLM
QEKSSIIISAKQEEVPSRAKIMRHHFFIFDGVHQMQEIRTSLFNTDGFYILALENNTIEDDVL

LMEFAADVWLQHGHSRIYYVQLSKKSVLLFNPFLQRLVVVQDSKTYSRIYKDLEGYHLRI
YIFDSVYSSVIGDGENKVLSVTGADAKLAKTVARQLNFTADFVWPDDEFFGGRLANGEYS
GGVGRAHRGEVDIIFAGFFIKDYLTTHIQFSAAVYMDELCLYVKKAQRIPQSILPLFAVHMD
VWLCFLLVGLLGALVWLILRAVNLILGIEGVPDGSRATRISYFGAARRIFVDTWVIWVRVN
VGRFPPFHSERIFVASLCLVSVIFGALLESSLATVYIRPLYYRDVNTLRELDESGQPIYIKHPA
FKDDLFYGHNSEVYRRLDAKMMLVAEGEERLIEMVSKRGGFAGVTRSASLQLSDIRYVM
TKKVHKIPECPKNYHIAYVLPRPSPYLEEVNRIVLRLVAGGIVGLWTGEAKERAKWSIQRF
PEYLAELDVGRWKVLTLSDVQLAFYALTIGCLLSAIVCMAEILLGRQRRLHSPK-
>HmelIR1
MWRTVILAACLTPDPGASLGAGSAAVDYFVQKGAPYLCYLTCENKKLVHEFMDRGIRVS
LQLIDKSCDLERNLLQWNVPVGVLLDAGCENTEDILNTASRGILFDAMHMWLIKEIADHE
GGNILNVLQRMNLSVDADVVVAFDKGDRVELIDVFNYGRIQGNDLEKKYIGEWSPEGGL
NVSLNRFKYYDRWDFHNLTLRAVTVILGDPKDFMPEMLSDVGYKAGVPLLTKIPTQLLYI
LKDIHNFSFKYTVAGRWIGAPERNSTLAVTNSLFWGEQDISCTSARMFKHWLEWVDVFSP
PATSFETKFYYLILDQGIGSYENRFLTPLSSGVWLWTAAAGVACTVLLGVAAVLEARSEPG
LYAFFSVFAVICQQAYEDGVNLFEEMSSSQGRRLVLLVVGITSMLLYNYYTSSVVSWLLNA
AAPTLADMDALINSDLELVFEDIGYTRGWLDNPGFFYYSGYKNPKEDELRLKKVTRARRT
TALLQPAAAISLIRTGTYAYHTEPYTAYQVISRTFSERELCELGALRMLGPDNVYIFGQKRS
PYKQFFVWSLMRLLERGHTSLASARVSGPRPTCSGRAPRALALGQAAPAFAALGYAALLS
VIIAVVEVWFHKAQNKKQARGEFDNRAHNAERSDFFSEA-
>HmelIR7d1
MNSSSSENLLYTDLSVLENMGRAAAKIAFYNFDWRYVALVVHNALNMISLEIFLKHYNKS
VVLKFGKFIPQKRLTVIPQFVIFGKDSNDVINIILWLNKLKYDNSGKYIIICAPYGYEDKCDE
IDIFVTLSRVYIVNAIYIRPKSEADFEMFAYDIVVPEKCINNVPYKLDVSNNCTNDDCFINLY
PRKLTNFYKCPLTVSTFHQPPFMILNNETSEPSGGDGEILKILISALNATLKIKIPAEGNTWG
RYYNGNWTGSLGDVYNNHAHMSVCSLPVSARKYANFTTSFIYNSMDIVWTAAIPLQKPS
WEKLMNPLDTSIRVGLFLIFSCIAIINAVTRLDIWRRIRMVLKIGPIRSSLLFYSWALFLGMSI
IKLPKNRSLLILVYSWIWYCFVIRNAYQAALMNSLKNKIYEEDYPSLDSVLKGRHLYGGLP
ALRQYYSDDPFIYENWKVLDFNESYNILDQISEGTSDFVLAFNKENIIEYLMESNGDKRLQ
IIPQKIISSPIAMYFKKHSLMASPVNRILSFAVESGFSQIVHRNYLRHKKYLFQLTRHKEYNP
LTLDNFKSCMLLMVAGWLLSLIFFVVETYCGQLEEDE-
>HmelIR7d2
MVANRYMESNIQFNDCVHIEMLAKSAARIALHNFRWQYITLVLLNTAVYCGANAFLESYN
KTAVIATKKWKPDLHKITKQFVLFGNDLGSILNLLAWMNDHKFDNTGKFLIICQSMVDGD
CDKLKAVEMFWHHKIVNIIFINNMPNIGAKGYTYDYGSNCQNSPATEVKDWNNCIKMDQ
KYCKNKYPIKLRNLHGCPIIVSTFMQKPYMNFSTGTPTGADGDLLNIIITALNASLTIRTPFK
GDGWGNLDENGTWVGSLGDLFYDLANFSMTSASITETRHRDFQISNFYYSIILGWVTHPP
EKEMASYKLLRPFKMDTRIALIISFGIVVCIVLFLKTKFCTIIFKQFSSGRDPENVLFLSWLM
CMGQPTLTLPSKMALSYLVLIWIWYCFLMRTFYQVHLINSLKNDIYLNDFSSINDAIKANY
PFGGGSALKDYYIDQHIIYNNWKHKDSSEYESLMLNLTHGMRFVLAMNLATAQDFLKEP
GRNLHILPQVILNCPSSIFFKKFSPLAKPINKILERLIEFGWTDMFFKNSTMGNNIQKTASED
KPITLSNFAGCYLILICGWLSSLIFFFLEIHLMKRHTQPIIPYRN-
>HmelIR7d3
MRILVFILVIFKIINILECTFTINKAVSIANKVFTYHKTAVVWWSFHVSDINKFLQQFRGTVLT

VSIENHANFNDSHFHKKIPYIQTIFFAMNPYEFDYFLEKVNYVRTVPINLILVLNEEYDTNL
TMYTKIAWEKDVSDILIISNNSSNEVIVSTFKPYRDGVCGDHTPMDLKTDSEQFFMRKYTN
FYKCPIRITLLEHKPYVILKFENDTVTSVTGMEGNLLMLLLNVLNSTANIVSCRDHGGFSG
TINGTMGFSDLYYNRADLMVPALIMQVNRYLYSQISYAHDSMHIVWCMPTRREIYEWAK
VILPFFNILTPFIIVSFLIMLVTINAVKKYALLQDNSKNDVAFRLLGIFMGQDTTYLSRYWLV
NSLFVCWIWLCMIIRISYQGNLVDGLQKTILEPRLSTLEEAVDVVDQVGGMRVFVDFYRN
TSLEKKYVQFLVKNVPSELKKVGDGKRNLIAADRTLVQFYRHNLEILDEPVMTTPICIHMR
PRWPAAPEISQLISRVVEAGLYTMIIRKEHSTWMILYGNLTETYEFKPIKLKTFHSCFYGLII
MYVMSSLVLVGEICFNRYKRREKY-
>HmelIR7d4
MAVFVVFVLSFIITSATANLTIKNEHNPRNEHVIDSATDIANSYFNFRTPTVVVSKEVDKVL
VTKFIQSYRGLIILDHTIGGPPKQVVIIIESYYSLIRTLGKLKPDLKGKTLLHSGAHFLIVVLA
TPHRLQRINSILWDYYATNVVIVIMNKNNKIVLYTYYPYKNHLDCQNIEPALIGFWDDGAV
LEKDLFPDKMSDMKGCPLYISTNKVYHPATEQKIPLEIIKRAIIRYLRDVMNFTPIISSRDYL
SIDSDGAKNWSETLDDILTGSANISTCSISPGMDRVGILDYSIPYFRISIAWLGPPLPPGPIWW
RLLSPLNGYLWLAILLVVIIVKSIPFIMKIGRVKQFCSQYFKNSNKLDGVVIRIWAVMMGQS
IRVKPRRFRDLYILSLWIWFTFVVRSAYQSVLIGALKSDIAVVRFVDLQQAVDNGYSFGGR
AGVLPHFEHDPFIRDGYEILQEEEFERVLQEVLEGKKTFIFAISLEYVWAYCMAQGINENEC
GHILPDSIMTVPLVIWMPKNSAFKRSLSVWLIRFLETGLLEKDTMKMSSTASVLKSTDPSA
LEIEQVVSSILCLAVGYLISIIVFIVEIILFKMKQSSIVKKSNNFLKHISK-
>HmelIR8a
MDLQLLFLFLLINLGCVISELSLRFVFITENHETELPQEISRAIKATEQISPGLLINEDVIQLDR
ENDEESFSKLCASLSKGVSSIIDLSWSPWEKIEELSSTGLVIVRSLLGSQQMIRGFDEYLESR
NATDAAIILESESDVDQALYELLGRSHIRIWVHAGLTRDSAKALKNMRPEPSFYVIVGDNN
FVTSTYKRAVKEKLVKRPYRWNLIITDYKTLDPSILVLPAVVLQSDKADCCKLLATKECSC
AADMQKKQHILNNLIKYMAESLSRIDSDQIVNIDCDNQQGQMNATKEKLYQIFDEDSLSN
ETLFYWDEERSGIYHRSHFVLSTYKPESGLQRIASWSADDEYKLLPGVTLEPLKNFFRIGT
SAAVPWTLPKYDPETGQPMFTEDGHPIYEGYCIDLIDKIAEAMDFDYEIVTPKTGTFGRRL
PNGSWDGIIGDLMTGETDIAIAALTMTAEREEVVDFVAPYFEQTGILIVIRKPIRKTSLFKFM
TVLRTEVWLSIVAALLLTGFMIWLLEKYSPYSARNNPDAYPYPCREFTLKESFWFALTSFTP
QGGGEAPKALSGRTLVAAYWLFVVLMLATFTANLAAFLTVERMQTPVSSLEQLARQSRIN
YTVVESSTIHQYFINMKFAEDTLYRVWKEITLNATSDQSQYRVWDYPIREQYGHILLAINA
SGPVPDAKIGFQQVNEHPEADFAFIHDSAEIKYEITRNCNLTEVGEVFAEQPYAIAVQQGSR
LQEDLSRTLLDLQKERFLEQITSKYWNESARQACPDADESEGITLESLGGVFIATLFGLGLA
MITLAWEVFYYKRKEKNKVTTIDATLEKKEAFIEKIPKKFEGIVKLKKRNKKSGNIVKNVT
IGDHFKPADKGISYISVYPKNSDYRP-
>HmelIR21a
MKFIIQILLTLNLTCVLISCNEKYITKFGLNNLGEKSILRYHQLFSKTYRKSRNFHKHIKETKI
QKLFSMNTSMKTVITKRNADPVFHGHPKTREEKWHEMFLSQTKTFDQNPSLIQLIHNITLT
YLNDCTPVILYDDRIKAREGYLFKDLFKSFPVTFIHGYIDEKDIIKEPRLLQPRYECFHFIVF
LSDVKSSAKVLGKQSKSKVVVVARSSQWAVQEFLAGPLSRYFINLVVIGQSFKDDDNSEA
AYILYTHILYTDGLGASKPKVLCSWSHGKFSRHVNLFPTKMTKGYAGHRFVVAAANQPPF
TFRRIHTDADGGNPKIIWDGLEIRILNLLAEKNNFSVEIIEPHSGELGPGDAVTNEVAMSRA
DIGVSGMYVTSERTSNIDMSFSHSQDCAVFITLTSTALPRYRAILGPFHWHVWVALTFTYLI

GIFPLAFSDKHTLRHLINDKGEIENMFWYVFGTFTNCFTFVGKKSWSKTTKITTRLLIGWY
WVFTIIITSCYTGSIIAFVTLPIFPETVDTIQQLLSGFYRVGTLDRGGWERWFINSSDPLTNKL
FKKIEFVPDVKAGIRNTTKAFFFPYAFLGSQAELEYIVQSNFSMSESKRSVLHISDKCFVPF
GVSIGFPNNSLYGTKLSNDIRRMLQAGILDKLNDEVRWEMQRTSSGKLLSVGSSDLLKIAS
IEEKGLTLEDTQGMFLLLGAGFLLAAVALLSEWMGGFSRGCIFGRKKTKSSITSAKKLINTP
QNSVEDIHFNSGPRSDLHFISRSPSNESQDTLEGHIIDVTQEAITVHKNFDEHKWDSRRSSS
VELDREVQVIFQKNSSKDLEDLVNDKSEDTAKDVFGDYL-
>HmelIR25a
MANMFEMKFVFAIYSLYLIPITVAQTTQNINVLLVNEENNALAEKSFEVAKEYVRRNPSLG
LAVDPVVVVGNRTDAKAFLENVCRKYNDMLSAKKTPHVILDFTMTGVGSETIKSFTAAL
ALPTISGSFGQAGDLRQWRSLNANQTKFLLQVMPPADILPESIRAIVTKQDITNAAIIFDEFF
VMDHKYKSLLQNIPTRHVITPVKSFNKDEIKAQLRSLRELDIVNFFVVGSLRTIKNVLDAA
NDNQYFGRKTAWFALSLDKGDISCGCKDATIVYMRPTPDAKSRDRLGKIKTTYSMNGEPE
ITSAFYFDLSLRTFLAIKSLLDSGKWPSDMKYINCDDYDGKNTPDRALDLKSAFQEVKETP
AYAPFFIPEDDLINGRSYMEFNTDLTAVTIKDGASIGSKSLGSWKAGLSNPLLLTDPDNMSD
YSAQLVYRIVTVEQQPFIIRDDEAPKGFKGYCIDLIEEIRQIVKFDYEISLSPDGNFGTMDEN
GNWNGVIKELIEKRADIGLTSLSVMAERENVVDFTVPYYDLVGITILMKLPRTPTSLFKFLT
VLENDVWLSILAAYFFTSFLMWVFDKWSPYSYQNNREKYKDDEEKREFTLKECLWFCM
TSLTPQGGGEAPKNLSGRLLAATWWLFGFIIIASYTANLAAFLTVSRLDTPIESLDDLSKQY
KIQYAPLNGSSAMTYFERMANIEVRFYEIWKEMSLNDSLSDVERAKLAVWDYPVSDKYS
KMWQAMKEAGLPNSIEEAIERVRSSKSSSEGFAWLGDATDVRYYVLTSCDLQMVGDEFS
RKPYAIAVQQGSPLKDQFNNAILQLLNKRKLEKLKENWWNNNPKAIKCEKQDDQSDGISI
QNIGGVFIVIFMGIGLACVTLGVEYWWYKWRKRPTIGEITQVMCGNLY-
>HmelIR31a
MLPTKVIADLFHYRTISSVIVLTCWPYFDRAKFIREINKYNIPVTISCDPAILDVVDPYKLQG
VLYIPSPKILEHKIKQKYFSNWYKWMILNNTIPHFIQNARYDADILLLKPSPTYNNSSLETE
DITIYDVYTHPRDDASVFLWAHWDLHAGLQVLFDKERIDRRRNLSKYPLKIAAPLGSYSN
NTYNGGFLEYLMDMSIRERDSGVRIGYSSSSLIIEILNATEILLPTLLWSTEIHNSSMILELKY
GSSELSGGVLRLMKERFEKLDYIMPIWPFHVGFTYLAERESSSNMFLEPFTVNVWWCCLI
LFLILAAAQKVTAKSKEEKEGAYIATLATFLQQDASAVPKGISGRWNFLVLSISAMLIHAYY
TSAIVSALMSTGRSGPDSLRSLGDSKYAIASEDYDYMRYLMFDVKTNRDDLEYLKKRKLT
PKFYQDVHAGVKLIQTGSTAYHTEYNQLYPHLKTFTDDQLCKLQYVDTVPEVLSWITTTK
RGQWTNTFKVAGAWLQETGLAHRLVSRVRIKPPPCRAALLAERVNIHDIMPVLICTFAGAV
ISLILLGLELFVSKFKGKWLKKSSSDSIEVFDVDDPIY-
>HmelIR40a
MKMKILLLLLFHIFIYCKNTECLHNIEAMTNRSLERLPKDFSKAIMDIAIGLPTNTLTIVSSNT
TNLRSEDLFKIMCLLAENNIQVINLDITSENKEVYFSYLKNGLDTSEERTSLIFCDSYECENL
LFEITINNFIHRSMLYIFFWPRGEISTLFLSGIKEAIRVAVITNPRPGVFRLYYNQATPNKLNH
LTLVNWWSGSLYKSPALPPADKVYQDFQGRIFDVPVLHAPPWHFVIYNNDSTVNVTGGR
DDKLLSLLAKKLNFKYRYYDPPERSQGSRIAGNGTFKGTLGLIWKRKADFFLGDVTMTW
ERLQAVEFSFLTLADSGAFLTHAPDKLSETLAIIRPFRWEVWPLVGATLLITGPALWIVIAAP
SLWQRRQTDQLSLLNNCCWFTTSLFLRQASTKEPSKTHKARLVGILISLGATYVIGDMYSA
NLTSLLARPTKEQPIGTLQALEEVMRDKGYELVVESHSSTLAILQNGTDVYGRLARLMRR
QHIQRVRSVEHGVRLVLSKRRVAILGGRETLYYDTERFGSHNFHLSEKLYTRYSAIALQIGC

PYLETFNNIVMTLFEAGILAKMTTDEYKNLPEQSRRSNPVTESDSKENTDVTENTQNAQP
QNESTIGLEPVSLTMLRGAFCLLGIGYFLAGTVLITEIHIQRKKKNQNLIKIPDESTNQLKSS
RFYLKHHFEKIFKIMYILIDRALRHDARD-
>HmelIR41a
MIPLPQALTALEILLKILIGQYLNSSYCITIISDEYLFLQSPKSFMYIYASNNETLTYQMLNAS
EMGCSDYIVQLNEPNMFFVAYENVARMGNVRKGDRKIIMLPTFHHLINGSSDVILNVLSN
REASFLANILLIMKAESSPECHIYDLVTHKFVGIEENDKPVVLDSWNSCTEEFEKNVYLFP
HNMSNVYGKIVKVTAFTYKPYVLLDLEPNITPNGRDGLDIRIVEEFCRWINCTIEMVGDDG
REWGEIYDNRTGIGILGNVYEDRADLGITALYSWYEEYIVMDFSTPYVRTGVTCVAPSPRL
LASWRMPLLPFNLYMWICIFFTFIYASLALMIAKGFSTTDIFLTTFGIMITQSQPESGFSTWR
TRSVIGWMMMTGLVLDNAYGGGLASTFTVPRYEPSIDTIKDIVDRKLIWGATHDAWTFSLI
LSQEPLLKKLVKLFRTYPSEDLKRRSFNRSMAFSIEKLPGGTFAIGEYITKEATFDLTIMLEE
YYFEQCIVMMRKNSFYTHKLSKLIGRLHESGLLLAWETQIALQYLDYKVQLAVKLSRSKK
DVENIEPLALRHVEGIFIIYGVGVVISIISFVIEIIQKKRNNKRR-
>HmelIR60a
MINILNIISLLSIGVGVTQSFYMQKTIHGTYSRTSTTAVTSCLKHIIQENFINPGFLVLACPKE
PSAKIFNIRRDVLKFLHKTEKYAVEITNPDDIPIFNNNVNINIEFWSFDPFNFTPTADYFLIILD
NYEDFTYIARKIIRSRFWNPQAQFIIILFQLNNTDTNNKRTAERILSCLFKFNVVNVIICIPSM
KNVLNTLIYSWQPYDPPNFCGHFNETAENRTIVLNYCENGKVKYVNDLWINKLPLDMQG
CTLRILALERQPFVSINKFDPNIEKWLINLLFKKFNFSVEYEIINAFRGERNEKGLWNGALK
ELSARKGHILLGGIFPDYDVHEDFECSSSYLSDSYTWIVPRAYPSPPWVSLHIIFQNLVWFS
AIAGFITCVLTWVFLGHLSKDSNYNQSFGHCFLNTWLCLLGLVAYVRPNKESLRVFFVFLN
LYCIIFLTAYQTKLIDMLQNPSFQDQIDTVEKLLQTDLKIGGSEELHDLFYNSTDPFDQLIDK
KWITISNMTKAMHDVAVNRNLSVLCSRFELVHLSSVLPELSDSNGHNNYYAFPLNAFSVPL
EIIALRGFPFIREISRDLNKYKQLGINAKVRNYFNVLSMRKRSRLVNTSKIEGKFYALSVQH
LQGGFFALAVGAFGGLIVLIFELLSTTTYTSRQRQLKGK-
>HmelIR60a1a
MWVALILISLYSPTYEFKDFPDQNNRLEKCLCHIIKEYFSHYSLLTYVDLQYENEDIIKIVHS
LTAVPLLAKSNRLSKSSLDESYLIVAENVTNFYHIISFLVKEPSWNPNGRFLILVGNIKGGMR
KIFDILLEHHIVDVLLLSDFINPNLYTYNPFENYGCGRRYDRIIEFGKCIEAENLNLYPKKLL
TALENCVMQIVSPHWPPYTINPLKTAKTQMGIEEYILREISILEKFSINFTFTDDAETFTMIK
NDMSAVGPLYSLQEGKADIILGGMILTHPRAKAFNYIYGHLSFVEDIRFQVQKSSAVPSWK
NMYLEFHSIVWTLFILTFATFFIIFIILVKPKDKGQIILKMFAYLFLNGRKIGGNYFTKFLFIIW
IWFAYLINTYYQSSLVSLITNPSLNYQVSNEKDIENFNLKPCVSTGIRKYLLSVENISLETLD
RDGCETMLESIKAVSERNDIYTVVLHSVYKYNEYKFFDDWGDPLVYTFKQPLSKVVYAIY
VNKGFPMLERLRLQALRLRENGLIYHYTRNMHWQNNRNYHFFEKTRKFYIIVPWYILAG
GYGLSIAVFFLEIMIKKHSSKMHNLLINCHTSI-
>HmelIR60a1b
MLVAITLVCFCIYPLNEAYITKVKQSNTEILKQCVCNIVKNYFSNYSVITYLDLYYDSDEIYE
ALHSLKSINVIQRDKIIGVNKMDESYLIASSNTSTLIKVFPLIQREPSWNPHAYFLLIIKSLAE
RELKEVFDILLKAHVLNVLVLNGHSDPDIYTYNPYENYGCGRRYHRIIEYGKCLSPNTEN
WYPYKLVTGLENCTIRVTSPHWPPYSIDPNRRNNSHVGVEEFIFRQMGILEHFQVEFQYSD
DAEIFTMINNDMAAVGPMNLIQSDVTDAVLGGMILTHTRAHAFSYVYGHLSYTDEIRYQV
QKAQPVPTWKYMYLEFNILIWIVFVLTFITFFLIFITLVKPGDKIRVIFVMFRYLLLNGVRIK

GTYFTKYFFLIWIMFAFLINIYYVSSFVSIATDQPVSYQISNEQDMYDYNLKPCVSDLMRN
YLLSVENMTLGKNVGENCNGLLDSIQTVSTSSSIYTVVLYSIYMYRRNQFYNRFGNPTTYS
FPKPVSKIVYAIYLYKGFPLVEKLTKHAVRLRETGLIEYHLKDLHRQNENKYMFARETRKS
HVIFPWQILTVGYVVCSIVFLLEIIISKKLYLRKLILPSQRNVIT-
>HmelIR60a2a
MKLLTMLLQIFLIIFHISDNIAVSQNLNSVLSTSNTLHDIYENAVTQCIVSTSYKYIPCGTHYT
LVSSTYFIDGVNSLLSYKTCYTFLSRSFETRKWKTWTDVYIFYIKNYEEMYISFTTLSKDGF
WNSRALFFIVIDDLYKSEYSEVFNILLNHNIFNVVLITKSNGHFTAYKYDAFDEGNCGQSIS
KIEEISDCRNLHSVRFNYTYLENKLRNCTITVAAFEDMPNVIFHTKKVSKIPQWTEKGLEQ
YIIDNIAEKENLTIKYIYDLTHGNGVVLHNLTVTGVLSYLHNNTASVVIGGFALIKNRIKLFE
YISGYCTYNLCLFTPASVEETWKKVYQEFGISTWLLIGLTYIFAAVVITVTRRFLLGDEDRK
RIILKIWGYLYGQTDDVLIKTNKMKKIVIFWIWFTFFMTSFYNSALCSLLTRNVKVNTKIDT
SSLSALPWELLPCISNVARTFYQFTFNETFSGNTNANCKLSEDVLDTIANNVKYFGLDADY
TYQMRKCKYIDENGNSKINSRHYPNDLMLAMYTTRGFPLHHKFQKYTTFHFESGLLQRQ
RAKIYHQLVTPSHHYKETFKILRLSDFRIHYAVLIIGYTVSLILFIIELSLNKVLQTFNNYQ-
>HmelIR60a2b
MKLLNMLLQIFFIIFHTSNNIAVSQNWNSDLSTNNILHDIYENAVIECIVSTSYKYIPCGTHF
TLVSSTYFIDGVNSLLSYRSCYTFLSRSFETRKWKTWTDVYIFYTKNYQEIYISFTTLSKDV
FWNSRAVFFVVVDDLYKSEYSEVFNILLNHNIFNVLLITKSNGHFTAYKYHAFDEGKCGQS
INKIEEISDCRNLHSIRLNYTYLENKLRNCTITVAAFEDMPNVIFQSKKVGKIPQWTEKGLE
QYIIDNIAQKENLTIKYIYDLSHGNGVIFYNFTVTGVLSYLHNNTASVVIGGFALIKNRIKLF
EYISGYCTYNLCLFTPASVEETWKKVYQEFGISTWLLIGLTYIFAAVVITLSRRFLLGDEDR
KIIILKIWGYLYGQTDIVLIKTNKMKKIVIFWIWFTFFMTSFYNSALSSLLTRTVKVNTKIDT
TSLSALPWEPCISDATRTFYKFTFNETFPGNTNANCKLSEDVLDTIANNVNYFGLDADYTY
QMRKSKYVDENGNSKINSWHYPNDLMHAMYTTRGFPLHHKFQKYTTFHFESGLLQRQR
AKIYHQLVTPSHHYKETFKILRLSDFRIHYAVLIIGYTVSLISFIIELSLNKVYYIYSTINH-
>HmelIR60a2c
MKLVTLLQISFIIFHFCDYLATSQGLNSNLFTNNYLHDTHQNSAIDCIVSTSYKHIPCGTMF
TLVYSSYFIDDVNKLLSNKNCYTFLSRSFEIRKWRTWTDVYIFFTKNYHEVYTSFTTLSKD
VVWNPRAHFFIIINDLHEAEYSEVFNILMNLNIFNVLLITKSEKGIFSAYIYHALEGGNCGRS
LNKIEKINDCRNSYSVKINYTHLDHKFRNCVISIAASEDMLNVIFESKKVNKRPRKVERGIE
QYILDNIAEQENLTLQYINADSKHEYGVILSNSTITGLLGYLHNNTASIVIGGFLLMRNRISL
FEYIWGYATGNICIFTPALGEENWKKVYQEFDVGTWLLICFSYLFVTVVITMSRRLLMGHE
DKGRVILKIWGYLYGQTDIELMKFKKMKKIIIFWIWFTFFMTSFYNSALYSLLSRNVEVKS
KIDTKSLSTLPWEPCISNAMRTFYKFTFNETLPGNTKANCNLTDDALDTVANNNKFYALE
MDYSYHISYNKNGNPKLKSWQLPSDLMLAMYTTRGFPLQHKFQKYATFHLESGLLQRQR
ATISPKYVTPNHRRRNTFKIFHLSDFRIHYAILIFGYTISFVCFIIEINHNYLRY-
>HmelIR60a2d
MKLFTQFQKFLMIFHIYNNFVTLHGLNSNIFINNSLYDTYQNSAIDCIVSASYKHIPCGTMF
TLVYSSYFIDDVNKLLSNKNCYTFLSRSFEIRKWRTWTDVYMFFTKNYHEMYTSFTTLSK
DVVWNPRAYFFIIINDLHEGDFLEVFNILMNLNIFNVLLITKSEKGIFSAYIYHALEEGNCGR
SLNKIEKINDCRNSYSVKINYTHLDHKFRNCVISVAATEDMPNVIFESKEVNKRPRKAEKGI
EQYILDNIAEQENLTLQYIYTDSKHDYGFILSNSTITGLLGYLHNNTASIVIGGLMLMRNRIS
LFEYIWGYDTASLCLYAPALSEENWKKVYQEFGVGTWLLICFSYLFATVVITMSRRLLMG

HEDKRRVILKIWGYLYGQTDNELIKLKKMKKIVIFWIWFTFFMTSFYNSALYSLLSRNVEV
NSKIDTKSLSTLPWEPCISNAMRTFYKFTFNETLPRNTKANCKLTDDALDTVANNNKYYA
MEMDYSYHMREPYYLDENGNPKLKGWQFTSDLMLAMYTTRGFPLQHKFQRYATFHLES
GLLQRQRAAIYHQYVTPNNHHKKTFKIFHLSDFRIHYAILIIGYTISFVCFIIEINGSYVRK-
>HmelIR60a2e
MKLFTLFQKFLIIFHICDYFATSYGLYSNIFIKTLLRDTHQNLAVDCIVSASYKHIPCGTMFT
LVYSAYFIDDVNKLLSNKSCYSFLSRSFEIRKWRTWTDVYIFFTKNYHEMYTSFTTLSKDV
VWNPRAYFFIIINHLREVEYSEVFNVLMNLNIFNVLLITKSEEGIFSAYIYHALEEGSCGRSL
NKIEKINDCRNIHSIRMNYSYLDHKFRNCIISVAATEDMPNVIFQSKEVNKRPRKVEKGIEQ
YILDNIAEQENFTLQYINADSKHEFGVILSNSTITGLLGYLHNNTASIVIGGLMLIKNRVSLF
DYIWGYDAASFCIFTPALREKNWKKVYQEFGVGTWLLICFSYLFVTVVITMSRRLLMGHE
DKGRVILKIWGYLYGQTDMEMIKFKKMKKIVIFWIWFTFFLTSFYNSALYSLLSRNVEVNL
KIDINSLSTLPWEPCISNAMRTFYKFTFNGTLPANTKADCQLTDNALDTVANNNKYYALE
MDYSYHMRQTYYLDENGNPKLKVWKYASDLMLAMYTTRGFPLQNKFHRYATFHLESGL
LQRQRAAIYHQYVTPNNHHKNTFKIFHLSDLRVHYAILIIGYTISFVCFIIEINRNYLRN-
>HmelIR64a
MDLLKFALILNFTIIMVLPVPVYIEILKSRNIGNVILSHCNRNIIESQKNLIANNIRSVFLANS
TDLSNLSYAKVGFILDTHCESWNQIFNVVDERFFRGPFTWLVNTDDLNSTVDVLSKYPIDI
NSDFIVITKNRKVFNLYEVYSKGFYTNGSFIVRDLGHFDSELHLKRNIRADLTGVVLKCVV
VIVDPIVNETFEHYLENTSPGKLIDSVHKLKFFRLMKYLQNIYNFSLMLQRTNSWGYLRN
GSFDGLVGSLQRRDADIGGSSIFYRSDRLKIIDYVTSTWQTRHCFILRHPKYPGGFYTIYTG
PLTDIVWYWILFVLTLAGLVTCIMLKIRAIKRPGDDEDSSSSMALIFIFSAISQQGTTLNRGSI
SIKIVTFVTFIFTLTLYQYYNATVVSTLLREPPKTIRNLKDLLESNLKVGVEDVLYNKDHFR
RTTDAVTLELYHKKIVTGKEYNFFKPDHGMRLVKAGGFAYHIDSIIGYRIMRNTFTEREICD
AHEIYLYPPQKMAVTLQKNSPYREHVVIGIQKIYESGLMYRLKSVWDEPKPQCVHTPDSS
VFSVSLREFSSVLIMLALGMVLSVSILFLEIIVGRYKVKVYDFCH-
>HmelIR68a
MAGALVKLVILITIVLSSTVKGDVGAIVEDLQIRKDLEVLLIDILNGLARNDAVTCLTIICDD
VYLNVFEGALFIRTLDVPYIMIVVEDYEDLLSPNYNTLEPLRQARNDGCNVYAILMANGL
QLTRLLKFGDRYRVLDTRAKYIMLHDVRLFHSNLHFIWKRIVNVVFIKYHRKVSSKSEAW
FELSTVPFPNPIKGIFISRRVDIWKNGKLHYGRDLFADKTGDLNGELLNVVYFDHLPSVVI
MKSSNKVGGVEIEILNTLAQKMRFQPKLYQSIDPDKFKWGQKLSNGSYSGLLGEMVHGK
ADMALGNLQYTPNHLELTDLSIPYTTQCWTFLTPEALSDNSWKTLILPFTLYMWIAVLMV
LFVTGSIFYGLAMYYINLLDYRADSVHVKKQKIHYDAKPVGLYLFGEITNSILYTYGMLLV
VSLPKLPTGWSIRLLTGWYWLYCILLVVSYRASMTAILANPAPRVTINTLKELVDSKIACGG
WGEETKVFFQQSLDEIGQDIAKMFVEVNDPDEAARKVAKGVFAYYDSSDYLKYLSVKRK
NDMNFNNSTINASFSYNSNERNLHIMTDCVVNIPISIGFHKNSPLKPLADVYIRRIVEVGLV
EKWLNDAMYRIRSMDTTEEEVKALINLKKLYCAFIALAIGYTISLFCLFGEWLHWHYIVM
KDPNFDKYALDIYYKNKNKKT-
>HmelIR75d
MRGRAKRTFNSSANMDFFSFIVAYCVSKKLTLITAFLCWGPDDINKLLKLSRDAGLRVRVH
TDLTDPHLLPDLPAYRTFREGMLLDTHCDNVSQLLNKASDTRAFNLRHSWMLIDDSFYNF
TNVDNILSDTLILPDGDVTWVSKDTMVDVYRVKRDQDLMALNLGFRDSQLELELERLWL
KLPTTVTRRKNLNNVYLESATIVTQPQYFKGWSDLSDRQIDTFPKVTYPLLMLCGQDLNF

RFNLKQVDLYGEEHNGTFDGLVGRMQRGELEVGITSMFMRADRWRVVHYSAETIELKGA
FLFRQPSRSAVSNVFVLPFSRGVWAACAAVFAGAGLLLTVFGHVARQNVLDPTAQRLTLPE
SLTYAIGTICQQGSDLSPQLWSVRLLMFCTLLASLFAFTSYSAKVVAILQAPSDALNTIADLT
HSPMDMGVQETTYKRVYFAESNDPATQALYRRKLLPLGERAYLSVVEGIARVRTGLFAFQ
VEQSSAYDIISKTYTEREKCGLKEIKAFTLPMVAVPLRKHSGYRDLFASRLRWQREVGLMD
RFRRVWMSSRPKCDEGRGGFVSVGLVDVLPAAQALLAGMLLATAVLLAEKLYYFVKTLV
QPNLWSVAEVANIK-
>HmelIR75p1
MNRTEYFLHQIVILADLSCPGADGFLMQASDDGYFKSPYRWLLLDPERGPLKFNILEKLD
LPLNSDVVIAKKVNGENYQLIEAYKISESSNVIYTTRAAWTPARELRSLYKLEIIKENTKNM
SFSLTENDNSKNLMTSKYGTIEDYRDSKIISVRRKNLRKHTLTIANVITDSNETRQHLDDRL
NLHQDSITKMSYMVVKICFEMLNSTEKRLFTHTWGYKDKNGNWQGIIDHLLKKEADIGT
LTIFTQERMKVIDYIAMVGSTAVRFVFREPPLALLENIFTLPFTGAVWIAIAICVLGCALFLYI
TSKWEASVDMHPLQLNGSWADVLILIIGAVLQQGCTLEPRYAAGRCVTLLLFVALTILYSA
YSANIVVLLRAPSSSVRSLNDLLNSPLKLGASDFEYNRYFFKKLNEPIRKAIYNKKIAPKGK
KPNFYTMKEGVEKIRRGLFAFHMELNPGYRLIQETYQEDEKCDLVEIDYINEIDPWVPGQ
KRSPYKDLFKINFIRIRETGVQSNIHHRLHVGKPRCSGSISAFSSVGITDMYPAVIATLYGML
ISVVVLFLEIVYKRLLLLKERRMAVKNLYF-
>HmelIR75p2
MKLFSLLIIPIFIYLVQTKDENEISFIKEFVNNERKPTFVIFSNLCWKHSKKIMLFRKLNEAGI
RATTNFQAISALQDHALLFYVDLDCPKAEEVLNVSKLLRSPYRWLAVHDRSSNKFNISKL
WELPILADSDFVLAEKDQNIFTLTELHKPSPNGPTYTNPRGFYNGTLKDTRPHRELFRRRQ
NVMGHPLTMANVIQDSNTTQYHLPREDRLKLHFDTIAKMSWINVHIAFEMVNATPRYIFS
HRWGYKKNGQWSGMINDIKTGRADLGTNCVANTERLSVVVFTDSIAKFQVKFIFRQPPLS
YVANIFTLPFSVNVWIALVLSIVGSTFTILLASKWEAVKIGTNPTQLDGSIGDAMLLTMSAL
SQQGCYKEPKKLSGRIMLWVIFTALMALYAAYSANIVVLLQAPSTSIRTLEQLANSKITLAA
LEVDYNHFVLKAGKDAVRRKIYKKIEPDKGKPQFYDLNEGVERVRKGLFAIHILTGQLYR
RVEETFLETEKCDLVEVDFMNIVHPFVPVYKHSPYLELLRVAFKRIHESGIQMAVDRRLSV
RKPRCSNKMSSFSSVSLLNIRPVLMFMLIGIGISVLILILEILHDK-
>HmelIR75q1
MSEFNKMTPPKRIQFIDDIDDIFDSFQQHNTLFIVDTDCPHSGGFLEKANRTQKFRNPYRW
LMITKPEKNKTSLSLRKIKILHILIDADVLISERHGDAFSLYSPYKLQSNNSEWKIENFGTW
TRERCLNNNKEMFSELSMRRKNVQHLPLPTSIVITNNKTKKSLLSLKEPDIDLVAKASFRQ
LAPLYQFMNATLVLHFTDSWGYPVNGTVNGMMGALANGICEIGGTVIFMRPERLSLADFI
AAPIMPTVRFVFRQPSLSQQNNLFFLPFKPVVWGCILGLIFFIFIIALIITKWENSKTEKENSK
TDTTIQADSSEIAMMIIGAITQQGSYTELKGTLGRIVMFITLFVFVFLYTSYSANIVALLQST
SNQIKTLADLLNSNLELGCEDTLYNRYYFKMVTDPVKKAIYEQKIAPRGSQPNFMSLHEGI
LKLQKKPFAFNMFLGSGYAAVEKYFLEHEKCGLQEITYVDEKLPWLTCRKNSHFKEIYKIG
FTRILEHGMNSREYRKSFVKKPVCISRGGTFDSVYMRDFYPALLMLAYGMILSVILVLLEIL
HWRRFKTIQFTL-
>HmelIR75q2
MSIGYVMAKIPFIILFLFFKYAFSEKVIETNIIANIIQTLERPTTIIVITCLTPYNKVQFHSKLAG
RNSDKRNLIEFIHPGEIPKNIENNHHILFIADLGCQNVLDSLKIYDNLKLFRSPFRWIFFNKN
CDNINFKRTIQNITDINILLDSEVLVIQQYCGYFCEIYYTYKRSSESEWETELYEKWNITNG

MEKKSRHNEATAIRRLNLNNYELKICYVLTDNDSINHLTDEVNDHIDTITKVNFPTTNQLL
DFLNASRKYIFTETWGYRHNNTWNGMTGYLVRQEVDIGGSPMFFTSERMSVIEYISSPTPT
RSKFVFQQPKLSYENNLFLLSFKYSLWIGSIALIGLLYLVICAVTIWEWKKKSSSKNGVESV
GILRPNLVDIALFIFGCTCQQGSVVELKGSLGRMVLLLLFVSLTFLYTSYSANIVALLQSSSS
QIRTLEDLLRSRMKFGVHDTVFNKYYFTTETEPIRRAIYETKVAPPGGTPHFMSMEDGVK
AMQKGLFAFHMETGVGYKFVGKYFEEGEKCGLKEIQYLRVIDPWLAVQKNTQFMEMFKI
GTKRLQEHGLQQRENHLLYEKRPKCSGRQANFISVSMVDCYPALLILTYGALLAAMVGVI
EILYYRRAEIMSKIK-
>HmelIR76b
MAGVELILATICNATFCDNIFDNPLLDTQLTARQEEIQDLKDEVNGKHLKIATLNNYPLSW
TEKLENGTIVGNGVAFIIIDILRQKFNFTFEIVLPEKNVEMGTGKKPDESVINLVNSSKADM
AAAFLPMLVKYNKLVTFSNDLDEGVWMMMLKRPKESAAGSGLLAPFATYVWYLILAAVI
CYGPCITLLTWLRAKCVKDNEYHISLSPSVWFVYGAFIKQGTTLSPEANTTRVLFATWWLF
VILLSAFYTANLTAFLTLSKFTLDIENPIDLYKKNYRWVATEGGAVQNTIKSPEEDLYYLSR
MVSNGRARFRTIKFNEDFLPSVTGGAVLVKEQDAIDNLMYNDYLRKTKEGVAETERCTY
VVAPNAFMRKRRAFAYPRNSTLKILFDTVLSHLIQSGIVKFLERMDLPSTKICPLDLQSKDR
QLRNSDLMMTYLIMVTGLAAAIAIFIGEITYKRYLNKKLLNGISTKPNKNNKKQRNKVTR
THYDDSRPPPYDSLFGKTKYKFNDSEKRIVNGREYYIVHNAVGDTRLIPVRTPSAFLYH-
>HmelIR85a
MFKVILALSVFKLCLCLNESSPLYTEIFTYHNETYLRADFAFQVVNKIFNAFEQWFFTITFC
EFTYFENRILKYTENYGHGYPVMLLNGCQDPNATEVKPKLDTHGNTAYLVTSKSLTIESSE
YALQALIRTGVFKPRSSIIFVVNTPVEINSYFNYDMFNHFQLLWSRSITNSIIMVWTDKLRM
YLYNPFYSKIKDVTDVRDIAHILAKQHEDLNGHEIRLSVFRKIFISDETGPVYCNSKLASTV
ISILNATCKPLAPRDGNTVGDMLPNGTATGVTADLLDGYTDMELNSRILKNSYYGYIDTTY
PLVQDELCFLIKKSMSQSTFMTTIRLISIDMLFAFIFIVIALIGVSLLIRKVETRIWEIKDRRSQ
SETVLDLIKCFIRQTVDIKYPGPLFRAVALLIMIYSLIVDCAIDGIITSAIAYPRYKEDINTLDE
ILANNLTLGVHNRHMRIFKRSINPEHLDQIMERTEAINDQKIKQVIEERQYQYAVLLKKSD
AQYISRKPSNMRNGRPLYHTAGDCPVPCSVVYGLRYGSPYLPRLNYLLNHLNQGGILRH
WSVSDEYTLLQTKNKVSYSENNKEKKPLNMKNVREVIIVWSFGLFISTTVFIIELITNFVNK
IPVI-
>HmelIR87a
MRFRCLFKYLLFIKLALIASSENPFLKDAENVEQITNSAECVLRLSGKYFVEKKALSGSIIIV
SVNSKASAAENFLLRTMHGGKNYSIMVKNAFRQHANASHFPEKAKNYLMILKKKFELLN
HLIQLSGLPSWNPLAKAIIYYRLKTGEKGEDVSKAFINELRHYKLLKSIAFIYSPVTDEVTS
YSWTPYDSKNCAGKCKHVYVLDVCKNNNIRQLEVQRDMFPLDLKGCPLITYAIISEPYV
MPPERKVANTMWNDAYEFQKGGEINLVKIISQFTNMSLIIRMSEKVENWGEIYPNGTAGG
GYGLLMNDSVDLLIGNIEVTRKTRKLFHPTISYTQDEMTWCVPKARQAVTWDNLIIIFQW
TTWLATFSSVLVMGLIFHYLHYQENRDTKWPTYSILMTLCMLLGWAAKFDAKSSQFRIL
MFGWLCFALNMGITYESFLRSFLMHPRFDKQISSQSELIDSGIPLGGRQIYRAYFETNNASS
FYLYRKYNSTTFAEGIRRAALSRNFGVVASRKQAEYMDQELGKGAGLIYCFPESDNLYKY
GVVLLFRKWFPMLARFNNIIRSVSENGLIDKWNEELFIHRISSDGTGTIMPLSIQHLLGAFM
LIGILYGVSVAVFLFEIFLGFFKKNNRIQDVNVRTPSL-
>HmelIR93a
MKLWMVACVIWSSLQYGQAEDFPSLITANASIAVVLDRQFLGDEYQTTLDEIKDYIKELA

RVELKHGGVNVHYFSWTAISLKKGYLAVFSVASCEGTWSLFQKTEEEQLLLFALTEVDCP
RLPTDSAITVTYAAVGQELPQLFLDLRTQKGMNWKSAIILHDDTLNRDMISRVVESLTTQI
DDDDVPSISVTVFKMKHEVNEYLRRKEVNRVLSKLPVKYIGEKFIAIVTTAVMATIAEAAR
ELLMSHTQAQWLYVISDTSGRGNFSNLINDLYEGENVAYIYNVTENDEGCKVCLICYAKE
MMSAFISELDSAVQEEFDVAAQVSDEEWEAIRPTKLQRRDTLLKHMQQYIMVKSECGNC
SWWRALAADTWGATYREKTYETKRNVTSIVIEHVELLNVGIWRPIDGIKFEDVLFPHIVH
GFRGKELPIITYHNPPWTYLQSNESGSIVSYRGLLFDIVEQLAKNKNFTIRIVLPGNIKQNH
TNDTATDMSHSVSSMLTLSAIAKGRAALAAAAFTVLSDHNPGINYTIPISTQTYSFMIARPR
ELSRALLFLLPFTTDTWLCLGFAVILMGPTLYIIHRLSPYYDAMEITREGGLSTIHNCLWYIY
GALLQQGGMYLPRADSGRLVVGTWWIVVLVVVTTYSGNLVAFLTFPKLEIPVTTISELLES
KTYTWSISKGSFLEMQLKSSDEPKYKALVKGAEVTGGINVVEGSLVSGSEILNRVRNQRH
ALIDWRLRLSYLMRAETVKTDTCDFALSAEEFMDEQIAMIVPAGSPYLPVINKELNRMHK
AGLITKWLSAYLPKRDRCWKTSTVEEVNNHTVNLSDMQGSFFVLFLGVFSATTVLLLEWI
FKRRQKKVDQIIIKPYTD-
>HmelIR143a
MWSMYNLQSLERTRKILYTYYVVKSLNANMLLRNSYKELLIFLMTARIIAGINKGIFQVEE
NNDSLQFMKCFTIIPEVYQISYKRTIVIYEDENTLEDNIINVFMDKLIENQMPFMKINTREEL
LKLSKVYDGTMLIVSYFNNCEETLKVDLSIFYLNFKYIFVIDSYIDEVCIQALEHTLSPVLY
YDITFLTRAQNDTYNLATLIPEIDEHSCRVSENAPMKVINTCHNGTLEKTKITDIFPIKTPRN
LKKCNFNVGMATLFPYSVLDNKESYKDLDLVNDKIYGADIEYMKIFANIFNATLKMHYIF
REEENPYLDFNFLKYLFNGTLDACAGGLYRIYGDVVAYSGIYSGQSVIWTYTVERQIRSW
QSLIVKITGLYIFFIFYIIYTALWKLISKYDNRSDSIQDTLIYAWGALMGTSGLQDARCLKQK
ILNVVYLIMCIHLSSYIGTQLYYYLTIEQPPQTFKTVDELALSDRTPYLRSLTKYFIDDKKHK
AFANTSLECDNFKDCERAMLKHKGSTIIIDGQLPPLQAATSVNDEARVLGVPQVILFVYHE
MLMRTDHMLVTKFDKITGRLFEAGICHKLYIEAIGITVVDKAKIANENILSNSYSCLVGCQI
TLRESAGAFYIWIIGCGLSCCVFVIEILLKRKLKSKTT-
>iGluR1
LICNQFARGVFAMLGAVTPESFDTLHSYTNTFQMPFVTPWFPEKVIPPSSGLIDHAVSMRPD
YHKAIVDTILYYGWKEIIYMYDSHDGLLRLQQLYQTMQPGRTAFRIALVKRINNASDAIEF
LLALEQYDRWGNKRIVLDCNAKNAKSILVEHVRKVQLGRRTYHYMLSGLVMDDHWENE
VTEYGAVNITGFRIVDHSRKIVRDFMDGLRRMDPRFKGTISAETALMYDGVQVLMDALG
RLWRKKPDAFRSALRRAAGQANSTKVIDCNPGKSWVVPFEHGDKISRLIKKTDIEGLTGNI
SFNEEGHRHNFTLHVVEMTVQSAMLKVATWSDAHGLQVATPRYVQLRSPASYDTNKTYI
VTSFLEEPYLMQKPIEFGQKEELFGFCKDLMDVIAKKMGIKYKLKLSNDANYASDALPDI
HSGVVGEIVRKEADIAIAPFAVTPERERLVDFSEPFLTLDTPIAYTRTPRQLSDTFSFLRPLSK
EIWLCVLFSFFAVSIVLFLVSRFSPHEWKSVSISDTQLDHTMSSTSEIILHNEFSIWNSFWFSL
GSFMQQGSDVVPRSLSGRIVGTVWWFFALILVCSYTANLAAYLIVERIAEPALSTVSYSPNI
AHTESSSLNFRNNFVKDPVLNEEAISAYSDDGEACGPSRVCRYKHVNFAFATAKGSPLREA
INLAIVNLKKEDFITKLWRKWATYNKKPDCEMIKDEETTIT-
>iGluR2
MSRLLLKWLAATLCLLRVHGDRTLGAIFDDGTFLLEAAFNVAIAAASEDQENPFVANVIKT
SPSDITEAENAMCTLLESNVFGVFGPTKKGSLQHIQSIADYLEIPHIITDPVETQNRNWSVIN
LFPHHLAYSQLFADLIELKGWTDFTIIYEGAELLPFFDSILSMQDLDTGQKILIKIVQLPDGD
DFRSQLKFIKKSGSVNYIINCRRETLPLVLEQAQQVGIMSDEHSYLIMNPDFQTIDIDPFKH

GGSSITGIRMFDPSLESIQNFITSLNEKVAELSENEIENAIAENGLTLDLALVYDAVTLFVSTL
NAMSLEEGSNVTCDDAESWGFGSSIVNYARTMEVDGLTGIIKFDEDGFRSEIEIDVLEIMSY
GLDKVGTWTLEDGFVETKDNVSPAEQEGSESMKGKHFVVLTALSAPYGMLKESLKKLEG
NDRYEGFGIELIDELAKINEFNYTFDIQEDGVYGSYDKKTGKWNGMMEKIMDGRADFAIT
DLTITAARQKAVDFTSPFMNLGITILYKKPTKEPPDLFSFISPFSMGVWGWLAGAFVGVSCL
LFILGRLAPEEWQNPYPCIEEPETLDNQFTLANSFWFTLGSVLTQGSEIAPIAVSTRMAGSM
WWFFTLIMVSSYTANLAAFLTVESKFYAIKSVNDLASNPYGMTYGAKKGGATFSFFKESD
NLLYQKMYHYMEDHPELQTATNDQGLDRVKSDSENYAFLMESTSIEYMVERNCDVAQV
GGLLDSKGYGIAMKKNSPYRQPMSESILQLQEEGKLTRMKDKWWKEKRGGGACADDD
AGGGEAQPLVLANVGGVFIVLAAGSGMAVVCAFVEMVFDVWMISRKMKVSFREELKAE
LKFILSFSGDTKPVRHRESTGSGSGGSKDDGEKNADAESPDDDDRADPSPTPRSERSGHSH
HTLHSRRQSNAVQMAKMRKYSLRSAM-
>iGluR3
IHPGNVSCDKDTPSLHGKAIYDNINTIQAHGLTGPLEFKQGIRKNFHLQLMRLTGGEKGG
MVVSGTWSPAEGLAITDPAAYTRDPPPNVTLTVVTVEEKPYVMVKEGWNLQGNARFEGF
CIDLLARVAARAGFHYRLRLVPDNMYGARDPDTGHWNGIVRELMDRKADIAVASMTINY
AREAVIDFTKPFMNLGIGILFKVPTSQPTRLFSFLNPLAIEIWLYVLAAYILVSFTLFVMARFS
PYEWSTSTHVCGHETKLLTNQFSVCNSFWFITGTFLRQGSGLNPKATSTRIVGGIWWFFTL
IILSSYTANLAAFLTVERTVLPIQSAADLAAQHHIHYGTLNGGSTMSFFRDSNIDIYQKMWE
HMSSASPPALVSSYEEGVRRVLAGNYAFLMESTMLDHRVQRDCNLTQIGGLLDSKGYGIA
TWKGSPWRDKISLAILELQEKGVIQILYDKWWKNTGDVCNRDGKDSKANPLGVQNIGGV
FVTLLCGLALAIVVAILEFCWNTKKNASQGRQSLCSEMGQELRTAMRGGSSSRTVLRPGC
SRCSPATHVPPATSRYQHSRSS-
>iGluR4
MRGANAIFLILFFGHLSALPDTIRIGGLFHPEDEKQEVAFRYAVERVNADRAVLPRAKLLAQ
VETISPQDSFHASKRVCHLLRSGVAAIFGPQSAPAAAHVQSICDTMELPHLETRWDYRTRR
ESCLVNLYPHPAALSRAYVDLVRAWGWRSFTIVYENSDGLVRLQELLKAHGPSELPVAVR
QLPDSHDYRPLLKQIKNSAESHIVLDCTTERIRDVLQQAQQIGMMSDYHSYLITSLDLHSV
DLEEFKYGGTNITALRLLDPERADVQRVVRDWVYDEARKGRKLQLGHTTAKENMTFIKT
ETALMYDAVHLFAKALHDLDTSQQIDVRPLSCEAEDTWPHGYSLINYMKIVEMKGLTGVI
KFDHQGFRSDFTLDIIELTRDGLQKAGTWNSSEGVNYTRSYGENQKQIVEILQNKTLIVTTI
LSSPYCMRREASEKLTGNAQFEGYAIDLIHEISKILGFNYTFKLAPDGRYGSYNRETKEWD
GMIRELLEQRADLAIADLTITYDREQVVDFTMPFMNLGISVLYRKPIKQPPNLFSFLSPLSL
DVWIYMATAYLGVSVLLFILARFSPYEWDSPRNCLDEPPVLENQFTLLNSLWFTIGSLMQQ
GSDIAPKAVSTRMVAGMWWFFTLIMISSYTANLAAFLTVERMDSPIESAEDLAKQTKIKYG
ALKGGSTAAFFRDSNFSTYQRMWSFMESARPSVFATSNKEGEERVVRGKGAYAYLMESTT
IEYVVERNCDLTQVGGMLDSKGYGIAMPPNSPYRTAISGAVLKLQEEGKLHILKTKWWKE
KRGGGSCRDETSKSSSTANELGLANVGGVFVVLMGGMGVACVIAVCEFVWKSRKVAVDE
RASLCSEMASELRSALKCPGGGAGGGGGGPGGARDGAGSPYLHYGFSTKSQLH-
>iGluR5
MWSYHVFLAVVYCGGQLVTADIDRRRFSNPTYYNVGGVLSSNESIAFFKDTISNLNFKDQ
YVPRGVTYHDYSMLMDPNPIKTALNVCKDLIAHRVYAVVVSHPLTGDLSPAAVSYTSGFY
HIPVIGISSRDSAFSDKNIHVSFLRTVPPYSHQADVWVDVLKHFNYMKVIFIHSSDTDGRAI
LGRFQTTSQSVDEDVDRKVVVEQVIEFEPGLDSFSDRLMDVKGAQARVFLMYASKTDAEI

IFRDATFLNMTTVGYVWMVTEQALDAANAPEGLLGLRLVNATNEHAHIQDSIYVLASAIR
DMNTSEEIHAPPSDCDNSGSIWTTGRLLFDYVRKQRLENGATGHVAFDDHGDRVHAEYD
MVNVRAQGEHVAVGKYFYSKETQKMRLELKEQEIIWMGRSTSKPEGFMIPTHLKVLTIEE
KPFVYSRRIDDGSECTPEEIPCPHYNASEDTDQLYCCKGFCMDLLKHLSKAINFTYSLALSP
DGQFGNYIIRNFSQPGAKKEWTGLIGELVYERADMIVAPLTINPERAEFIEFSKPFKYQGITI
LEKKPSRSSTLVSFLQPFSNTLWILVMVSVHVVALVLYLLDRFSPFGRFKLANIDGTEEDAL
NLSSAIWFAWGVLLNSGIGEGTPRSFSARVLGMVWAGFAMIIVASYTANLAAFLVLERPKT
KLTGINDARLRNTMENLTCATVKGSAVDMYFRRQVELSNMYRTMEANNYDNAEQAIED
VKNGKLMAFIWDSSRLEFEAAQDCELVTAGELFGRSGYGVGLQKGSPWADLVTLAILDF
HESGIMESLDNLWILRNNMLNCEENEKTPNTLGLKNMAGVFILVLAGIIGGIVLIVIEVVYK
RHQIRKQKRMEIARHAADRWRGAVEKRKTLRAAILPSQRRAKSNGVKEAGSISLAVERGA
RRRDEPRVPRYLPAYTPDVSHLVV-
>iGluR6
MHVLVPLLLVCVSQCVSAQEGPPIGGIFYKDSEDMKAALEVSAKSFNFTASIKEVSTRGEV
LEISKYVCQLAEEGVIGIIDGTGGRSSEIIQGLCDALELPHISIEHNDLYSDDWFVLNMYPSP
TAYNMVLQKLILHKEWKNFTLLYTKGHSLIRVSELLQMGNDTLVVSLRELSGSDYRDVLI
DAKHNGYKNFVVDSPSRYLEQVLLHAQQVGMMAEEHSYIFVSPDLFTLDMSRFKYGGV
NMTGFRLVELQDKDNEKLWNFTSTLNLETGKTFKPEQLKTQVLLIHDAVEVFAAAFKKV
KVQPEALSCDNYQAWSFGSTLLNFMKTNKVEGLTRSLIFDGVGQRTDITFNILELTSAGNQ
SIGNWTNNELKINRPLVADAEITQESALRNKSLRVLISLAPPYGYMRKSDKKLEGNDQYEG
FTIDLIDKLSEILGFSYEFAVEDDYGTKTESGEWTGMALQLREERADLAICDLTITAVRQSGI
DFSTPFMTLGIGILYKEPSKQPPEMFSFMAVFSKEVWYYMMLIQLALGATMIFVGRISNKE
WQNPVPCIESPEELNNQFSFANSVWLIIGSVMQQGSEIAPIAIGPRMITSVWWFFTMVMVA
SYVGTLVAFLTVEKNVLPFETVQELYESKSITYGAKEKGSTKQFFENSTNPIYQAMFKKMK
AHNWLAKENDIGVYWAETQNYAFFMESTSLEYYKERHCDLLQVGGLLDSKSYGIGMKK
KSPYKKYIDDALLKLKENGEIEKLRNIWWKEKRGGGKCGEKRDADQKQLGMKNMLGAF
VVLGVGCLIGLFISIIDMLWGVFKRSVKYSTTFKYELIEELKFALKFSGHIKPVKRPQKAID
GSFEALAKAEGKDDIRSLHSIRSCDTHRTHHSHSSRHSSRSLSVAFAKRRSYS-
>iGluR7
MCWYLLVLLLCVQKCTPQFVVYTEMTEISYQIVGIFEKDATIQMAAFNDSLNHVHVQEVTI
RPATLQPRRTDSYSVWRELCSNNAIQAVAVFGPQNPITDGAIRDQCAIANIPHIQATWQSMD
PDLELNEETTAQEEGEDEESEIPFKKISINFYPDSEEIALAYGKLLQYYKWGGFAALYEDNF
GLLRVQKILAEVSLQSQIFMYKLDPKGDNRRIFKALRKFQVSRFLLDCHSDHILRYLGEAD
NAKLVSEYQHFVLVSMDTSTVAESLIKMPSNITWLSITQYDKLKDGGHYLATRVGNWRSN
EESPSVVQFKLDSLIMDDVASHLVKALDVKDIAQPPVFSCTGDEEPWAHGAAYQQKILKT
QSYGVTGNVEFDQRGRRINYVLYINEIHIRDRQTIGRWESATGMINETKRLDSSAANQQSS
KEFVVISRRAKPYFSFKEKCEKTECKDDDRFEGFSVDLVDNIFRILREEKYNYTYRFIHDY
DMEYGKVDPTTHTWTGLVGYLLDKKADLAVCDLTITEERKKVVDFSVPFMSLGISILYTQ
DRKVQPGMFSFLNPYTFEVWMHTATAYCVVSIVLFICARISPADWENPEPCEKDPEELENI
WTFKNCAWLTMGSIMTQGCDILPKAIGTRWVCGMWWFFAVIVCQTYIAQLSASMTSALE
NEPINSVDDLAKQTKIRYGAIVGGSTLEFFKASKDKTYRHMYETMAANPAVLVTSNDEGE
ERVLKSKNTYAFFMESSTIEYKLKRNCKLKKVGGELDSKDYGIAMPANSPFRTDINRAILR
LKELTTLDKIKNKWWHEKYGAQKCEPTVDENDIEGDLEMENLMGAFVVLVVGLVFCLFI
TAIEFMNEVRNIVVREQVTHKEVFIKELKASLNFFQLQKPVLRNPSRAPSIASSDSDERREN

QAKAIENFMNLEKAV-
>iGluR8
RTSPAPPPPRAPSSITAALVVPHKAFGTRDYTKAEKAALSKLPRKLKLFSHVRLNITLSTQG
LTPSPMSILDSLCKEFLAVNVSAILYLMNHEQYGRSTASAQYFLQLAGYLGIPVIAWNADN
SGLEKRASHASLRLQLAPSIEHQTSAMLSILERYKWHQFSVVTSAIAGHDDFIQAVRERVT
ALQDRFKFTILNAIVVKRSSDLNELVTSEARVMLLYATREEAAEILSAAGDLHLTGENFVWI
VTQSVLGSMQQPNKFPVGMLGVHFDTSSSSLIAEIATAVKVFAYGVESYVSEPENIRYPLGT
RLSCSGAGAGEARWSTGERFYRHLRNVSVEGEAGRPSIEFTPDGELKAAELKIMNLRPAL
GEQLVWEEIGTWNSYPRERLIIKDIVWPGGLHTPPQGVPEKFHMRITFLEEPPYINLAPPDP
VSGRCSLDRGVICRVAPEVEVAGLEAGAAHGNSSLYQCCSGFCIDLLQQLAEQLGFTYELV
RVEDGRWGTLHHGKWNGLIADLVNKKTDMVLTSLIINSDREAVVDFSVPFMETGVAIVVA
KRTGIISPTAFLEPFDTASWMLVGAVAIQAATFSIFFFEWLSPSGFDCSTGNNSKRVPQNRFS
LCRTYWIVWAVLFQASVHVDSPRGFTARFMTNMWAMFAVVFLAIYTANLAAFMITREEY
HELSGLDDPRIARPLTQRPPLKFGTVPWSHTDATLAKYFTEPHAYMARYNRSTVSAGVTC
VLTGELDAFIYDGTVLDYLVSQDEDCRLLTVGAWYAMSGYGLAFTRNSKYVSMFNKRLL
DLRANGDLERLRRYWMTGTCKPNKQEHKSSDPLALEQFLSAFLLLMAGILLAALLLLLE
HVYFRYLRSHLAASSAGSCCALVSLSMGQSLTFHGAVVEAAARGFGERSHCRSAVCAAQ
VWRARHERDMAVARARQLA-
>IR1
MHPWLILTNIEDADNCTDYIQQSFQQLNLSVDADIAVASYNGGDNYTLTDVYNFGKIQGN
NLEVNHLGSWRPETGLDIKLKGYKYYNRWNFQNLTLRAISVIVDQPEMFYPEMLSEMTY
TAGVAAMTKITSQMLNTLKEQHNFRFNYSIAGRWIGSPKRNSTLAVTNALFWEEQDLSST
CARIFPKWLDWVDIIHPPTTNLQTKFYYLIPETGVGQYENRFLTPMSHGVWGCAFIAGIAC
TLVLTGAAWMESRPKPGLYAFFSVFAAVCQQGYEDGVQLLETYSSQGRRLTLLVIGLTSML
LYNYYTSSVVSWLLNAAAPSIGNLDGLINSDFELIFEDIGYTRGWLANPGFYYYSGFNNV
KEDELRDKKVTKAKRTVPVLQTVNTGVDLLRTGKYAFHTEPYTAAQVISKTYEDEELCNL
GALQMMLPAHVYIMAQKRSPYKEFFDWSLLRLLERGHVKAIRARFAGTMPACSGARPRA
LALGQAAPAFLMLLLCVLLSWIILAFEVLWSRVQLKKRGP-
>IR1.2
MAEDVLYFASQNMWLDSHHKWLLIDDDKAEIEGYNDTEIDNVLFEDRNTTLIDILSNLNIS
VDADIVVAEKGNSSYNLYEVYNYGKIQGGNLIVNEIGVWNHENGFNLNINLNGYKYYRR
WDFQNISMRMILVVQRASKNFDLESLTGPEPVPGVAMITQTPTDILYIVAKIHNIRYVSTITD
RWIGSYEKNSSKVVSTSLYFREQDVSPVIRGLSTVYERIDVINPPLTSIETRYYYRIPTMGPG
KFENQFLRPLSTTAWWSVIGVSTLCAGLLLLSALLEQRPSSVQYAVFSVVASLCQQFFQDID
DSGTKRISTARKVTILVTGLSCVLLYNYYTSSVVSWLLNGPPPSINSLKELLESPLELIYEDI
GYTRSWLQSPSYYFNKRNAPIEDELRQKKVFNKKKNAPLLEPLVQGIKMVQKGGYAYHT
EVNSANALISRTFSQSELCELGSLQSMEKTLLHPCLQKHSPYKEFMTWSLMRLSEQGIVSC
IQIRRSSFEVKCEGSSPRALALGGAAPAFILLLGGYM-
>IR2
MAYWYYLLNAVMTTMIGCSPMKNRSMIIIRSACLSGSILFLAVYQGHTSRVYTTLKHFERI
STLDDLYAFGAILYTTPGMRQFTRQLQRPGNKLEEDFFNRSRLILNERIGAGITLEIPRATTL
DRKSDAEMKILEHFSDREGRPLIDIVDECFMNYFLSYITRSGFPFFEEIQIFTQRLLEAGLPT
KYYKWTQQMLNIPTSLPETRSEPRPFSKIKLKDQRVAFFVLFVGSALSIIVFAVEIFKGPPVE
F-

>IR7d.1
SVIVKLGRFLPARRAAVPQMIIFGEDASEISSTIRWTVRAKYDSNGKFIIICAHLEQECDELK
IFQTLQSLYMFNAVVLKTSNKTKESLAYSYDFLSEGKCKNSIPYKVNLTTDCFNDNCFKNL
YPERLSNFRKCPLIMSTIEQPPFMYLHNLTSKPTGIDGDIMRLVADMLNATLHLKPPYDGA
DSGHFANNNWTGSLGDIYNNHSHASVCSAPITSGKYGNFQISFTYYSMDIVWATRLPAQQ
APWQKLLHPLNIYIRIILLLMFICIIFMN-
>IR7d.2
LTANMAISELNHFFDTNVTAGSQLGDMAAKVAIQNFDLRHATMLFFNSTLCYGVEVFLQF
YHYNIVINRAKVLARKTTRQFVLFASDTADIELLLDSIISFEMDNTGKFIIICESSTPKECDEQ
DIMVLCWNYRIVNMVFIRQEETEAVGFTYYPVADGICNNLKPIKLDSHNQYTKTTYGEIFR
KKFRNLNFCPIIASTFIQPPYMYIKNGIPTGIDGDLLRMLIHGMNASLKMMTPSRGTGWGF
REKNGTWMGSLADVYDDLANFSMTSAAITLTRFTDFQISSGYSTSKVVWVSESAQVQNV
ALKLLHP-
>IR7d.3
TLDLEPPFESIEEAVTKVDGYGGVEVVVDYYKDTPLERNYKVIPMNELKNYIRRIVEGENF
ILATDIALVKLLEPYVQILKKRISATGACFYMRPGWPAAKDVDDVIFSLVEAGFIENLLSDN
NNHRWIVNRMNADDVLQPKSLSVEKLSTCFYGLGIMWLICFIILLIEIVHHNKH-
>IR8a
MSFHYLFLLIFLINLGCVISELSLRFVFIIESQEQDLTHEISKALKLAESVRTDVKLDDAVVV
LDRETEDESYRMLCSSLSKGVSMIIDLSWTPWEMVEDLAAETGVPVVRTLLGSQQLVKAL
DEYLESRNATDAAIILESESDVDRTLYELLGASNIRVWVHAGLTRDSAKALKTMRPEPSFY
VIVGENAFIMDTYRRAVKEKLVRRDYRWNLVLTDYSTLELSQLVLPTVTLQADPGECCKL
MRREDCSCPNDFQRKQYILNALIQYIAEVYSKLDRDLPLVTSSISCEEPEAIMNSTRDRLYR
QFAEDAEISNETLFYWDMDRSGLFLRSRFILSTYKPTAGQQTIATWSADEEYKLLPGVELE
PLKMFFRIGTAPAVPWTLMKLDPETGEQMYDDDGQPLYEGYCIDLIARLSETMSFDYEIVS
PKSGGFGKKLPNGTWDGVVGDLMRGETDIAISALTMTAEREEVIDFVAPYFEQTGILIVIR
KPIRKTSLFKFMTVLRTEVWLSIVAALVLTGFMIWLLDKHSPYSARNNPHAYPYPCREFTL
KESFWFALTSFTPQGGGEAPKALSGRTLVAAYWLFVVLMLATFTANLAAFLTVERMQTPV
SSLEQLARQSRINYTVVEGSSVHQYFINMKFAEDTLYRVWKEITLNATSDQAQYRVWDYP
IREQYGHILLAINASEPVPDAKTGFQQVNEHTDADFAFIHDSAEIKYEVTRNCNLTEVGEV
FAEQPYAIAVQQGSRLQEELSRALLELQKERFLEQLAGKYWNESARQACPDADESEGITLE
SLGGVFIATLFGLGLAMITLAWEVFYYKRKEKNKVQTLNTKPEKVAFESKSTLETKVAESV
AKLKKRGKKGNLAKNVTFGDTFKPVAEKGVSYISVFPKDYRP-
>IR21a
MSFVHGYINDGGELVERKLLHASTDCQNYILFLTDIKASAKILGKQPKNKIIIVARSSQWAV
QEFLASVVSRMFVNLLVIGQSFKEGDDANLESPYILYTHKLYTDGLGASQPVVLNSWNHG
KFSRNVNLFPPKMTEGYAGHRFLVAAANQPPYVFRRIKADLDGGNPRVVWDGVEIRLVK
LLAERNNFSIEIIEPLELHLGSGDAVAKEITSGRADIGIAGMYLTTDRIRDLDVSQAHSQDCA
VFITLMSTALPRYRAILGPFHWHVWVALTFTYLFGMFPLAFSDKHTLRHLLHNSGEIENMF
WYVFGTFTNCFTFLGKNSWSKTDKITTRLLIGWYWIFTIIITSCYTGSIIAFVTLPVFPETVD
TIKQLLAGFYRVGTLDRGGWEKWFLNSSDPQTKKLLNKLEFVPNVEAGIRNTTKAFFWP
YAFLGSKAELEYIVQANFTATKSKRAALHISNECFAPFGITIGFPNNSVYSEKMSLDISRMIQ
SGIIDKIADEVRFEMQRSVTGKLLAAGSGTIKIPSAEEKGLTLEDTQGMFLLLGAGFIIAATA
LVSEWMGGFTRRCRFQRKVDTPISVNSREHLIPTPKTDIGSEIKIIGDTESRLHFDSRPSTAA

SRDTLEGQIINVTEDNIDVHNSFNVDRFDSRRSSSLDLDREVREIFEKDQKRRRIVSQDMES
VDEHGPTVSRVAFGDPIKHEK-
>IR25a
MSTVTVLLLFNLVHIAFGQTTQNINVLLINEESNALAEKAFEVAKEYVRRNPSLGLAVDPV
IVVGNRTDAKSFLENVCRKYNDMLLAKKTPHVVLDFTMTGVGSETIKSFTEALGLPTISGS
FGQVGDLRQWRTLNANQTRFLLQVMPPADILPEAIRAVVTKQDITNAAIIFDEFFVMDHK
YKSLLQNIPTRHVITPVKSFEANEIKTQLESLRNLDIVNFFIVGSLRTIKNVLDAADKNQYF
GRKTAWFALSLEKGDISCGCKNATIVHMRPTPDANSRDRLGKIKTTYSMNGEPEITSAFYF
DLSLRTFLSIKSLLDSGKWPNDMKYITCDDYDGKNTPNRTLDLKTAFQEIKETPTYAPFFIP
QDDPMNGRSYMEFSTDLLAITVKDGASISSHSLGSWKAGLSSNLTLTDPNNMSNYSAQLV
YRIVTVEQKPFIIRDDKAPKGFKGYCIDLIEEIRQIVKFDYEIILAPDGNFGTMDENGNWNG
IIKELVDKKADIGLASLSVMAERENVVDFTVPYYDLVGITIMMKLPRTPTSLFKFLTVLEND
VWLSILAAYFFTSFLMWVFDKWSPYSYQNNREKYKEDEEKREFTLKECLWFCMTSLTPQ
GGGEAPKNLSGRLLAATWWLFGFIIIASYTANLAAFLTVSRLDTPIESLDDLSKQYKIQYAP
LNGSAAMTYFQRMANIEEKFYEIWKEMSLNDSLKEVERAKLAVWDYPVSDKYSKMWQ
AMEEAVLPNTIEEAIQRVRDSKSSSEGFAWLGDATDVKYHVMTSCDLQSVGDEFSRKPYAI
AVQQGSPLKDQFNNAILQLLNKRKLEKLKEIWWNNNPETMKCEKQDDQSDGISIQNIGGV
FIVIFMGIGLACVTLGVEYWWYKWRKRPVVGDVTQVEPAKTTRNNVDKQGEGFTFRGR
NLGLTFKPKF-
>IR41a
MLVSTPALVPLEILLNTIINQYLQSAYCITVFSETPFTFILPTSFISLIPNETNLVEQIFNVSETG
CSDYIVRMRDPQIFMEAFERVVHIANVRRSDRKIIFLPYDEEYNEENDVNLPSLVFAMKGS
KYLANMLMIVNHASVNQDCKIFNLVTHQFVGRSEEATHLPKYLDSWDSCTQKFENEANL
FPHDLTNLYGKVVRVACFTYKPYALLDIDPAIEPLGRDGVEIRIVDELCRWINCTVEIVKED
VDQWGEIYANESGGIGVIGSVVEDRADIAITALYSWYEEYRVMDFSVAGVRTAITCIAPAPR
LLSSWEMPLMPFTWYMWLAVVFTYFYASTGLLTAQGCSTTTYPFFNVFGMMIGQSQYES
RASWRIRGVTGWLLIAGLILSCAYGAGLASTFTVPRYEPSIDTVQDIVDREMEWGATHDA
WIFSLTLSTEPLVKQLVSQFRIHSFDELKRKSYTRSMAYSIEKLPAGNFAIGEYITQEAVLDM
MLMLEDFYYEQCVVMMRKSSPYTEKVSQLVGRLHQSGLLLAWETQVALKHLNYKVQVE
VRLSRSKNDVGTTKPLNLDNVVGIFIVYAIGLTISFAIFLGEIYVHQRKKKNEVLHID-
>IR60a
KMKARYNFIDGYRGERENVGEWNGGLKKLASKSGHLLLGGIFPDFDVHEDFETSVTYLA
DAYTWVVPRAHKSAAWVALVIIFKSLVWYSVIAGFFLCGITWKIIAELSEDSDYNRSFRHC
FLNTWITVLGFVSYLHPVKESLRVFFVFLNIYCMLFSTAYQTKLFEVLTNPSYEYQIQTVEE
LVESGLKFGGFEELHDLFYNSTDPFDYRIGDQWTDITNITEAMIDVAVHRNFSLLCSRLELA
HISGITPELSDSVGNYKYYTFTDNVFSVPIETIALRGFPFMMEFSTTITIFKQSGLNEGLRQH
FAHFNERRRARQLRALLKEKSDVNPLSSEHLQGGFLALALGYVSGTLALIVEVILNCNYV
QNKFENFKRRVNPLS-
>IR64a
INRESTSTKLLIFITFMYAVTLYQYYNATIVSSLLLEAPRNIRTLKDLLDSDLKAGAHDIVYN
YDYFKRTTDPVAIELYHKKVVTATQHNYFPAEKCMDLVRRGGYAIHIDTSVAFPLIKATFNE
REICDTTLVQMYPLQRMGVVMRKNTQYREHVANAIRRFSEAGLPQRLRSDVDEPMPECA
HTPDSSVFCVGIREFSTPLLVLALGMLLSVMLLLCEIILHRVVQRAGLRDFVH-
>IR68a

GMLLVVSLPRLPMGWSIRLLTGWYWLYCILLVVSYRASMTAILANPAPRVTIDTLKELVDS
KVTCGGWGTQSKKFFEQSLDEYSQRIGDKFETIDDPMEAANKVAQGVYAYYDNSDFLKY
ISVVRKNSFMDPKQNNTANNTEVTGRKDTQRNLHIMTDCVVNIPISIGFHKNSPLKPLADI
YMWRVVEVGLVEKWLNDAMYQIRTLETSEDEVKALMNLKKLYGAFIALAIGYSLSAICLI
GELIHWHIIVKRDPNFDKYALHLYYLHKNKKH-
>IR75d
MGIGREQFVWRVGLPASAYRCVIMISSSISSFSSSSSGGCLAASAAMDVYGVSHNGSFDGL
VGRLQRNDAEVGLASIFIRPDRMQVADYISETCVLLCAFIFRQPARSAVSNVFLAPFSAGV
WAASAGVAAAAALLLVALRAVLQRTRQRDDLALFTLPETLTFALGTLCQQGFHTTPGVTS
VRLVMFSTLLASLFVFTAYSAKIVAILQTPSDALRTIDDLTRS-
>IR75p
MNGSVLVDSDLVLAERVGNHFKMVEMHRPGLNGSMITTPRGFYNGSFVDVRPHRELYRR
RRNMMGHPITMSNVIQDSNTTREHLPKEDRLELQYDSITKACWSAAKIGFEMINATAKYIF
SYRYGYKVDGQWSGMIADLYSNKADVGTNCVIFRDRFDVVTYTDLVAPMRMLFIFRQPP
LAYVANVFYLPFSTRVWVTIAVCTAIATVTLYLASKVELVLTKASTQQQLDGGICDALLLT
MSAVTQQGCYIEPRRAPGRMMVFVLFTALMALYAAYSANIVVLLQAPSDSIRSLPQLANA
KITLAANDVDYNHFVFNQSKEPLHTSIRDRVFPENGKARLYSLADGVERIRKGLFALHSVA
EPVYRQIEATFLESEKCDIATVDYLVTFDSFTPVRKGSPYLELIRVVHKQIRESGIQSAIRKR
FLVSKPHCTTKMSSFSSVGLMDMRPVLILMLYGVAVSVTIVIGEIAVYKLMNRYKRSSKVQ
LIKVMK-
>IR75p.1
EADLGTLTIFTQERMMAVDYIAMVGSTAVRFVFREPPLSYISNIFALPFSGAVWLAIFICVLG
CSIFLYIASKWEASMGMHPLQLDGSWADVLILMIGAVLQQGCTLEPRYAAGRCVTLILFLA
LTILYAAYSANIVVLLRAPSSSVRSLPDLLNSPLKLGASDFEYNRYFFKKLNDPIRKSIYEKK
IAPKGKKANYYSMKEGVERIRKGLFAFHMELNPGYRLIQETYQEDEKCDLVEIDYINEIDP
WVPGQKRSPFKDLFKINFLKIRESGVQANIHQRLTVPRPRCSGHVSTFSSVGITDMYPAML
MTLYGMLLAPAVLLMEIMYHRLMIARQQKRGTSDYDHIPFRH-
>IR75p.2
MRIILPAFVLVLTLFERKVFARDHTLVHFIKSYVENEEKPTILIMNNLCWDKKIVVSLANEIS
KIGSRSSTSMGVDSRYYYHDLLYLLDLDCPGAEEIIALATARNLFRSPYRWLVITAWSKNA
NIAALWNSPVLADSDLVLAAGSGGVLKLVELHKPSPNGTMISTLRGFYNGSLYDVRPHRE
LFRRRRDVMGHTITMSNVIQDSNTTVYHLPREDGMEPQYDSISKICWMNVKLAFQMLNA
TPGYVFSYRWGYKVNGKWSGMIDDIHSGRAELGTNCVVSDIERLDVVAYTDRLAPFRVR
FVFRQPPLPYVANIFSMPFSKNVWIAMSVCAVLSTATVYLAAKWEAKEGKGPTQLDSIGD
AMLLTFSAIGQQGCVMEPRRLSGRMMVFVLFTALMALYAAYSANIVVLLQAPSDSIRSLP
QLANAKITLAANDVDYNHFVFKLHKDPVREIVYKRIDPEKGKKHFYDLNEGVERIRQGLF
AFHSIVEPVYMRIEQTFLETEKCDLMEVDFLNSYDTFVPVRKESPYLELLRVVFKQIRESGI
QSALSKRLQVPKPHCTSKMSSFSSVGLMDMKPVLILMLYGVCLSVTIAAAEILVFKLSEHR
KNTRLSQRSHHHHKNTISASLFAVVLRSFYLVICDSILYFNKIKRMRLK-
>IR75q.2
LCSLRLCYGQDSQNMQVMLSDVIEASGRPSSVIAKLCWTPSKIIQLHTHLTSKYIQFSAND
VIKADNSEFYDEEQHIVFMADLDCPDIATYFEKNSARNVFRAPFRWILFGNTSTNDDDIVP
RAISNIDVLLDSEVLVLRSVDDVYEMHFIYKISPNNTWQTEFYGTWNAKHRFQKSPRFFEP
TSLRRLDIDGYEISICYVLTNNESVNHLSDGLDDHIDTITKVNFPTTNHLLDFLNAKRKYIF

ANTWGYRVNGSWNGMTGYLVRGEVEVGGSPMFFTFERVSIVDYIASPTPTRSKFVFQQPK
LSYENNLFLLPFNTTVWYSTIALVFIIYLVLLLVTKWEWKKTQDLLETREKDAGVLRANV
VDIIVLIFGAACQQGSPSELKGSLGRVVMLVLFLALMFLYTSYSANIVALLQSSSSHIKTLED
LLHSRIKFGVHDTVFNRYYFSTATEPVRKAIYEKKVAPPGTTPRFMTMDEGVIQMRKGLFA
FHMETGVGYKFVGKYFNEGEKCGLREIQYLQVIDPWLAVRKNTPFREMFKIGTKRIQEHG
LQYRENRLMYEKRPKCTGGGSNFVSVSMVDCYPAVLILSYGAIVALFLLALEILAHKKENI
LRKLNCRKDEMN-
>IR76b
MAGIELIISSICNATFCDVPYGGADKGSEVLTPEVINFKSLMQDVNGKNLKVTTYNNTPLS
WTEHHNGTVVGKGVAFTVMEILRKKFNFTYDVIEPKRNYELGGRVTDDSIIGLLNSSKVD
MAAAFMPTLIAYRKQVSFSIDLDEGVWVMMLKRPKESAAGSGLLAPFNDLVWYLVLAAV
LTFGPCITFFTRVRTKLIADGEGVLPLKPSFWFVYSAFLKQGTNLSPEANTTRVLFVTWWL
FMILLSAFYTANLTAFLTLSKFTLAIENPRDLYQKNFRWVASAGSSVEHIVKTEGEDLYYLS
AMINNGKARFLSVLSDKDFLDPVKRGAVLVKEQTVVDHLMYNDYTSKKDVEESDKCTY
VVAPNAFMKKQRAFAYPVGSKLKSLFDPVLTQIFQAGILDFLKRSDLPSTKICPLDLQSKD
RKLRNSDLIMTYLVMVAGSATAVAVFAAEIFIKRYLSVKVNKTKKTKDRNKKSKIGKKSTR
YDDSRPPPYDSLFGKNPRFNVETTRTKIINGREYYVFETGNGDRKLIPARAPSSFLYRSDK-
>IR87a
MVKDSFYPHANASHFPEKAKNYMLILEEKSELERNILQLNKLPTWNPLAKAIVFYQLKHN
ETAEETSIEFINELRDYKLFKTIVFIYDEYNDVVISYTWRPYSDTNCGGRCDSVYVLDRCT
NNTIYEFEKQHDMFPSDMKGCPLVAYAVIAEPYVMPPVGKITNSSFDDAYEFAKGGEINLV
KIISQFTNMSLITRTSDILENWGVVYQNGTATGAFEVLRNESADLVIGNVEVTRILRKWFHP
TVNYLQDEMTFCLPKAGQAPTWDNLVIIFQWTTWVATFFSLVIMGLVFHVFYYREHTNAT
KWPTNSLLMTFSMLLGWGASFEPKSPTFRILIFAWLCFSINMGISYESFLRSFLMHPRFEKQ
ISTEADLIQSRIPLGGREIYRSYFETNNASSFYLYREYNSTTFSEGVRRAALERNFAVVSSRR
QAVYQDQKLGKGAPLIYCFPESNNMYKYGVAILTRRWFPMLERFNNIIRSVSENGLIDKW
MNELLIHSVSSEEASTIMPLSIQNLLGAFMFIGFMYGASIVIFIGEVVIGFIEKETTKKVMNK
RLR-
>IR93a
NPDCKNGIMCFCQELMDAFISALDAATQDEFDVAAQVSDEEWEAIRPSKIQRRNMLLKH
MQQHLAAKSRCGNCSTWRALAADTWGATYRGLSDASDLSNVNTNGSSGVIDKIDLLKV
GFWRPIDAVRFDDVLFPHIHHGFRGKELPIITFHNPPWTILERNESGAIVKYSGLIFDIVNQL
AINKNFTLKIILASVLKKELANDTLADTMHGMDAKLTIAAISKGQGALAAASFTVLADPM
PGINYTMPVSIQPYAFMIARPRELSRALLFLLPFTTDTWLCLGLAVILMGPTLYIIHRMSPYY
EAMEITRQGGLATIHNCLWYIYGALLQQGGMYLPRADSGRLVVGTWWLVVLVVVTTYS
GNLVAFLTFPKQEVPVTTVAELLENRALYTWSITKGSYMEMELKNSDEPKYIALLKGAEM
VTTSVGMGGTMTSGSALLQRVRFHRHVIIDWKLRLSYLMRADRLESDNCDFALSAEEFFD
EKVAMIVPAGSPYLPVINKELDRMHKAGLITRWLEAYLPKKDRCWKASSMMQEVNNHT
VNLSDMQGSFFVLFMGFFSASTVLVLEFLYHRRKRRSELTVIKPYVE-
>HassIR93a
RPRELSRALLFLLPFTTDTWLCLGLAVILMGPTLYIIHRMSPYYEAMEITRQGGLATIHNCL
WYIYGALLQQGGMYLPRADSGRLVVGTWWLVVLVVVTTYSGNLVAFLTFPKQEVPVTTV
AELLENRALYTWSITKGSYMEMELKNSDEPKYIALLKGAEMVTTSVGMGGTMTSGSALL
QRVRFHRHVIIDWKLRLSYLMRADRLETDNCDFALSAEEFFDEKVAMIVPAGSPYLPVINK

ELDRMHKAGLITRWLEAYLPKKDRCWKASSMMQEVN-
>HassIR75d
ALLLVALRAVLQRTRQRDDLALFTLPETLTFALGTLCQQGFHTTPGVTSVRLVMFSTLLAS
LFVFTAYSAKIVAILQTPSDALRTIDDLTRSPITIGVQDTTYKKVYFLESPDESTQQLYRRKIL
PQGERAYHSVVDGIARVRTGLFAFQVESSSGYDIIRQTFTEREKCSLKEIEAFKLPLVAVPM
RKNSGYRELFATRLRWQREVGLMSRERR-
>HassIR87a
WGVVYQNGTATGAFEVLRNESADLVIGNVEVTRILRKWFHPTVNYLQDEMTFCLPKAGQ
APTWDNLVIIFQWTTWVATFLSLVVMGLVFHVFYYREHTNATKWPTNSLLMTFSMLLGW
GASFEPKSPT-
>HassIR76b
MAGIELIISSICNATFCDVPYGGADKGSEVLTPEVINFKSLMQDVNGKNLKVTTYNNTPLS
WTEHHNGTVVGKGVAFTVMEILRKKFNFTYDVIEPKRNYEWGGRAIEDSIIGLLNASKVD
MAAAFMPTLIAYRKQVSFSIDLDEGVWVMMLKRPKESAAGSGLLAPFNDLVWYLVLAAV
LTFGPCITFFTRVRTKLIADGEGVLPLKPSFWFVYSAFLKQGTNLSPEANTTRVLFVTWWL
FMILLSAFYTANLTAFLTLSKFTLAIENPRDLYQKNFRWVASAGSSVEHIVKTEGEDLYYLS
AMINNGKARFLSVLSDKDFLDPVKRGAVLVKEQTVVDHLMYNDYTSKKDVEESEKCTY
VVAPNAFMKKQRAFAYPVGSKLKNLFDPALTQIFQAGILDFLKRSDLPSTKICPLDLQSKD
RKLRNSDLIMTYLVMVAGSATAVAVFGAEIFIKRYLSVKVNKTKKPKDRNKKSKIGKKSTR
YDDSRPPPYDSLFGKNPRFNVETTRTKIINGREYYVFETVNGDRKLIPARAPSSFLYRSDK-
>HassIR75q.2
DLDCPDIDTYFEKNSARNVFRAPFRWIIFGNTSTNDDDIVPRAISNIDVLLDSEVLVLRSVD
DVYEMHYIYKISPNNTWQTEFYGTWDAKHRFQKSPRFFEPTSLRRLDIDGYEISICYVLTN
NESVNHLSDGLDDHIDTITKVNFPTTNHLLDFLNAKRKYIFANTWGYRVNGTWNGMTGY
LVRGEVEVGGSPMFFTFERVSIVDYIASPTPTRSKFVFQQPKLSYENNLFLLPFNTTVWYST
IALVFIIYLVLLLVTKWEWKKTQDLIETREKDAGVLRANVVDIIVLIFGAACQQGSPSELKG
SLGRVVMLVLFLALMFLYTSYSANIVALLQSSSSHIKTLEDLLHSRIKFGVHDTVFNR-
>HassIR75q.1
LCGILTFMSLERLKILEYFTSPTPIAARFVFRKPPLSYQNNLFLLPFTTGVWICLGAFVLILIV
VLYINTKWDIKKYEQFNEQNMDQTCLPPTWSDTTIFVLSAISQQGSSNELKGTLGRLVMFI
VFLAFLFLYTSYSANIVALLQSTSNQIRTLSDLLNSKLELGVEDVLYNRYYFSPAQSASDPIK
KAIYETKVAPRGKPNFLTLEEGVKAMQKRPFAFNMNTGTGYRIVSALFQEHEKCGLQEIE
YYQNAKGWLCSGKNSPFSEMFKVGYIRIQEHGLTDRENRLTYAKKPVCSVMGSSFDSVH
MVDFYPVCLMLLYGMILAFILLVIEILAHRHQMRKHNQELNITQLQ-
>HassIR75p.2
MRIILQAFVLILILLERKAFARDRTLVHFIKSYVENEEKPTILIMNNLCWEKKVVVSLANEIS
KIGSRWSTSMGVDSRYYFHDLLYLLDLD-
>HassIR75p.1
RNPVPNSRLELPGTATQRISEEPVPKVTVTKYGRIEDYRSSKVLSSRRHDLRGHTLTMSNVI
TDSNETRVHMNDRLNLHQDSITKMSYAVVKICFEMLNATERLMFTHTWGYKDKNGQWQ
GIVDQLLKKEADLGTLTIFTQERMMVVDYIAMVGSTAVRFVFREPPLSYISNIFTLPFSGAV
WLAIFICVLGCSIFLYIASKWEASMGMHPLQLDGSWADVLILMIGAVLQQGCTLEPRYAAG
RCVTLILFLALTILYAAYSANIVVLLRAPSSSVRSLPDLLNSPLKLGASDFEYNR-
>HassIR75p

NGKPRLYSLADGVERIRKGLFALHSVAEPVYRQIEATFLESEKCDIATVDYLVTFDSFTPVR
KGSPYLELIRVVHKQIRESGIQSAIRKRFLVSK-
>HassIR68a
KEGCNVYVILLANGLQASRLLRFGDRHRIIDTRAKFIMLHDFRLFHSELHYIWRRIVNIIFIK
HHNKMTGTAKGRPWFELSTVPFPNPIKGVFVPRRVDIWKNENFHYKR-
>HassIR60a
LKFGGFEELHDLFYNSTDPFDYRIGDQWTDITNITEAMIDVAVHRNFSLLCSRLELAHISGI
TPELSDSVGNYKYYTFTDNVFSVPIETIALRGFPFMMEFSTTITIFKQSGLNEGLRQQFGHF
NERRRARQLRALLKEKSDVNPLSSKHLQGGFLALALGYVSGTLALIVEVIVNCDYVQNKF
AQCKRRVNLLS-
>HassIR64a
VVMRKNTQYREHVAYALRRFSEVGLPQRLRSDVDEPMPECAHTPDSSVFCVGIREFSTPL
LVLALGMLVS-
>HassIR41a
VSQFRIHSFDELKRKSFTRSMAYSIEKLPAGNFAIGEYITQEAVLDMMLMLEDFYYEQCVV
MMRKSSPYTEKVSQLVGRLHQSGLLLAWETQVALKHLNYKVQVEVRLSRTKNDVGTTK
PLNLDNVVGIFIVYAIG-
>HassIR40a
TTNLDIATKENKQKYYEFLGKALDVSDQRTSLILCEPYECENILFELTENNLIHSMILYFFY
WSYGPVSDTFLMTMKEAMRVAVITNPRESVFRIYYNQGTPDRLNQLTLVNWWAGTLYKS
PVLPPTDKVYHNFRGRIFEVPVLHAPPWHFVKYNNDNTT-
>HassIR31a
FDVETTWDELEYLKKKKKTSKFYQELERGVELIQQGNTAFHSEYNQIYPHFKTFSDDHIC
KLQHVDTIPETLTWVTSTKNG-
>HassIR25a
MSTVTVLLLFHLVQIAFGQTTQNINVLLINEESNALAEKAFEVAKEYVRRNPSLGLAVDPV
IVVGNRTDAKSFLENVCRKYNDMLLAKKTPHVVLDFTMTGVGSETIKSFTEALGLPTISGS
FGQVGDLRQWRTLNANQTRFLLQVMPPADILPEAIRAVVTKQDITNAAIIFDEFFVMDHK
YKSLLQNIPTRHVITPVKSFEANEIKTQLESLRNLDIVNFFIVGSLRTIKNVLDAADKNQYF
GRKTAWFALSLEKGDISCGCKNATIVHMRPTPDANSRDRLGKIKTTYSMNGEPEITSAFYF
DLSLRTFLSIKSLLDSGKWPNDMKYITCDDYDGKNTPNRTLDLKTAFQEIKETPTYAPFFIP
QDDPMNGRSYMEFSTDLLAITVKDGASISSHSLGSWKAGLSSNLTLTDPNNMSNYSAQLV
YRIVTVEQKPFIIRDDKAPKGFKGYCIDLIEEIRQIVKFDYEIILAPDGNFGTMDENGNWNG
IIKELVDKKADIGLASLSVMAERENVVDFTVPYYDLVGITIMMKLPRTPTSLFKFLTVLEND
VWLSILAAYFFTSFLMWVFDKWSPYSYQNNREKYKEDEEKREFTLKECLWFCMTSLTPQ
GGGEAPKNLSGRLLAATWWLFGFIIIASYTANLAAFLTVSRLDTPIESLDDLSKQYKIQYAP
LNGSAAMTYFQRMANIEEKFYEIWKEMSLNDSLKEVERAKLAVWDYPVSDKYSKMWQ
AMEEAVLPNTIEEAIQRVRDSKSSSEGFAWLGDATDVKYHVMTSCDLQSVGDEFSRKPYAI
AVQQGSPLKDQFNNAILQLLNKRKLEKLKEIWWNNNPETMKCEKQDDQSDGISIQNIGGV
FIVIFMGIGLACVTLGVEYWWYKWRKRPVVGDVTQVEPAKSTRNNVDKQGEGFTFRGR
NLGLTFKPKF-
>HassIR21a
FTNCFTFLGKNSWSKTDKITTRLLIGWYWIFTIIITSCYTGSIIAFVTLPVFPETVDTIKQLLA
GFYRVGTLDRGGWEKWFLNSSDPQTMKLLNKLEFVPNVEAGIRNTTKAFFWPYAFLGSK

AELEYIVQANFTATKSKRAALHISNECFAPFGITIGFPNNSVYSEKMSLDISRMIQSGIIDKIA
NEVKFEMQRSVTGKLLAAGSGTIKIPSAEEKGLTLEDTQGMFLLLGAGFIIAATALVSEWM
GGFTRRCRFQRKNDTPISVNSREHLIPTPKTDIGSEIKIIGDTESKLHFDSRPSTAASRDTLEG
QIINV-
>HassIR8a
QEQDLTHEISKALKLAESGKSDVKLDDAIVVLDRETEEESYRMLCSSLSKGVSMIIDLSWT
PWEMAEDLAAETGVPLVRTLLGSEQLVKALDDYLESRNATDAAIILESESDVDRTLYEML
GASNIRVWVHAGLTRDSAKALKTMRPEPSFYVIVGENGFIMDTYRRAVKEKLVRRDFRW
NLILTDYSTLELSQLVLPTVILQADPGECCKLMKREDCSCPADFQRKQYILNALIQYIAEVY
SKLDRDLPMVTTSINCEEPEATMNSTRDRLFRQFAEDTEISNETLFYWDMDRSGLFLRSRFI
LSTYKPTAGQQTIATWSADEEYKLLPGVELEPLKMFFRIGTAPAVPWTLMKLDPETGEQM
YDDDGQPLYEGYCIDLIARLSETMS-
>HassIR7d.3
MIRVLRILVVVQLLLRNCYASSKLIETAVNISITTYNLHFTTTVIWKQNESECVTGFLQSYPG
SVVLSPWATYNDSKVRDINETIGFKQTIYFANNLFEYEIIMELINEVIRFPIRFILVLENPVKS
NSEISSFIEVTARNDQADLILISENDAGEVTLSTFFPYSEGVCGNYTPVFLKYGDELWPKKF
SNFYQCPIRTALLEYFPYVTVHFEKGKITSVGGYDGKILMIILKKLNASLEVTSAYNNVFGT
YVNGTATGSIGDLATEKADILIPADILTEKRYTVTLPSHTYHTVDIRWVGPKQREVYDWLK
FIVPDKTNFTYLHLLVYILFVIVAMLVRKCKPQMTSATNRILYQSFIILLGQSAKFVTKSWLL
NSLFVLWIWFCFFFRIDYQADLVDALQTLDLEPPFESIEEAVTKVEGYGGVEVVVDYYKD
TPLERNYKVIPMNELKNYIRRIVEGENFILATDIALVKQLEPYVQILKKRISATGACFYMRP
GWPAAKDVDDVIFSLVEAGFIENLLSDNNNHRWIVNRMNADDVLKPKSLSVEKLSTCFY
GLVIMWVICFIILLIEIVHHNKHHNE-
>HassIR7d.2
EIFRKKFRNLNFCPIIASTFIQPPYMYINNGVPRGIDGDLLRMLIYGMNASLKVMTPSRGTG
WGFREKNGTWMGSLADVYDDLANFSMTSAAITLTRFTDFQISSGYSTSKVVWVSESA-
>HassIR7d.1
EDPVSELGTLAAKVAFYNFEWRFMTVVMYNTVQAIGLNTFLMHYEKSVVVKLGRFLPAR
RSAVPQMIIFGEDASEISSTLRWTVRAKYDSNGKFIIICAHLEQECDELKIFQTL-
>HassIR2
MNLDDPDDYYIEDTVPYHANFIIISCQDYEELEELRQKLAASPYFHPLANILTYYHRREDK
QTMAKLFFSAWYYKAINSILVQYSDEERTLLVSEYTPYVNEDYKVQPENKFGCWTARNLG
MPVVGFDTGYVCVEKCHNVSIHTRLRANNLGTCIGFNTHVVSYNDFKHLRNLKLFEDRT
KDLHGFVLRAYAVEVKPFFLIKKHENGTYTLSARDGMIWNTMAELFNFGIDLSPSVDVM
MKPFNFEISIDQIFAFARRKGDLCLFPIYQFDVIVVGLDFTYPFKDSGICILSARAGFETSLFR
IKTLRDNVSTIILFLACFACTWATFTVYKAAEKRHLSFDQIGKDFMNTCRQILMINLYKPPS
HHFFRIFLAIALWCFFVINFTSQATIISFFTAVKRGKEVDTFEDVVAKGYPLEAMASPDLILP
DTEEKFRIINSRLVNEVDIYGCVDRLKTDPRRFCLLDCSVGRYIKRNKLNDRGEQYLHIAE
QDRIHSHYLAMVFSEHSPMTERFSKYMMILCEAGLIRKWEQYRYTDIKDDVTTKPLAFDD
LSGIFQVFCFMVGNTLIIFFLELVASRIKTCCQNIKIPHGLPKRNIE-
>HassIR1.2
GTKRISTARKVTILVTGLSCVLLYNYYTSSVVSWLLNGPPPSINSLKELLESPLELIYEDIGY
TRSWLQSPSYYYNKRNAAIEDELRQKKVFNKKKNAPLLEPLVQGIKMVKKGGYAYHTEV
DSANALISR-

>HassIR1.1
NLDGLINSDFELIFEDIGYTRGWLDNPGFFYYSGFKNVKEDELRDKKVTKAKRTVPVLQT
VNTGVELLRTGKYAFHTEPYTASQVISKTYEDEELC-
>HassIR8
RTSPAPPPPRAPSSITAALVVPHKAFGTRDYTKAEKAALSKLPRKLKLFSHVRLNITLSTQG
LTPSPMSILDSLCKEFLAVNVSAILYLMNHEQYGRSTASAQYFLQLAGYLGIPVIAWNADN
SGLEKRASHASLRLQLAPSIEHQTSAMLSILERYKWHQFSVVTSAIAGHDDFIQAVRERVT
ALQDRFKFTILNAIVVKRSSDLNELVTSEARVMLLYATREEAAEILSAAGDLHLTGENFVWI
VTQSVLGSMQQPNKFPVGMLGVHFDTSSSSLIAEIATAVKVFAYGVESYVSEPENIRYPLGT
RLSCSGAGAGEARWSTGERFYRHLRNVSVEGEAGRPSIEFTPDGELKAAELKIMNLRPAL
GEQLVWEEIGTWNSYPRERLIIKDIVWPGGLHTPPQGVPEKFHMRITFLEEPPYINLAPPDP
VSGRCSLDRGVICRVAPEVEVAGLEAGAAHGNSSLYQCCSGFCIDLLQQLAEQLGFTYELV
RVEDGRWGTLHHGKWNGLIADLVNKKTDMVLTSLIINSDREAVVDFSVPFMETGVAIVVA
KRTGIISPTAFLEPFDTASWMLVGAVAIQAATFSIFFFEWLSPSGFDCSTGNNSKRVPQNRFS
LCRTYWIVWAVLFQASVHVDSPRGFTARFMTNMWAMFAVVFLAIYTANLAAFMITREEY
HELSGLDDPRIARPLTQRPPLKFGTVPWSHTDATLAKYFTEPHAYMARYNRSTVSAGVTC
VLTGELDAFIYDGTVLDYLVSQDEDCRLLTVGAWYAMSGYGLAFTRNSKYVSMFNKRLL
DLRANGDLERLRRYWMTGTCKPNKQEHKSSDPLALEQFLSAFL-
>HassIR7
MCWYLLVLLLCVQKCTPQFVYTEMTEISYQIVGVFEKDSTIQMAAFNDSLNHVHVQEVTI
RPATLQPRRTDSYSVWRELCSNNAIQAVAVFGPQNPITDGAIRDQCAIANIPHIQATWQSMD
PDLELNEETTTQEEGEHEESEIPFKKISINFYPDSEEIALAYGKLLQYYKWGGFAALYEDNF
GLLRVQKILAEVSLQSQIHMYKLDPEGDNRKIFKALRKFQVSRFLLDCHSDHILHYLAEAD
NAKLVSEYQHFVLVSMDTSTVAESLIKMPSNITWLSITQYDKLKDGGHYLATRVGNWRSN
EESPSVVHFKLDSLIMDDVASHLVKALDVKDIAQPPVFGCTGDEEPWAHGAAYQQKILKT
QSYGVTGNVEFDQRGRRINYVLYINEIHIRDRQTIGRWESATGMINETRRLDSSAMAQQAS
KEFVVISRRAKPYFSFKEKCEKTECKDDDKFEGFSVDLVDNIFRILREEKYNYTYRFIHDY
DMEYGKVDPTTHTWTGLVGYLLDKKADLAVCDLTITEERKKVVDFSVPFMSLGISILYTQ
DRKVQPGMFSFLNPYTFEVWMHTATAYCVVSIILFICARISPADWENPEPCEKDPEELENIW
TFKNCAWLTMGSIMTQGCDILPKAIGTRWVCGMWWFFAVIVCQTYIAQLSASMTSALEN
EPINSVDDLAKQTKIRYGAIQGGSTLDFFKASKDKTYRHMYDTMIANPALLVTNNDEGEE
RVLKSKNMYAFFMESSTIEYKLKRNCKLKKVGGELDSKDYGIAMPANSPFRTDINRAILRL
KELTTLEQIKNKWWHEKYGAQKCEPTVDENDIEGDLEMENLMGAFVVLVVGLVFCLFIT
AIEFMNEVRNIVVREQVTHKEVFIKELKASLNFFQLQKPVLRNPSRAPSLASSSSDERREN
QAKAIENFMNLEKAV-
>HassIR6
MHVLISLLLVWVSQCVSAQEGPPIGGIFYKDSEDMKAALEVSAKSFNFTASIKEVSTRGEV
LEISKYVCQLAEEGVIGIIDGTGGRSSEIIQGLCDALELPHISIEHNDLYSDDWFVLNMYPSP
TAYNMVLQKLILHKEWKNFTLLYTKGHSLIRVSELLQMGNDTLVVSLRELSGSDYRDVLI
DAKHNGYRNFVVDTPSRYLEQVLLHAQQVGMMAEEHSYIFVSPDLFTLDMSRYKYGGV
NMTGFRLVELQDKDNEKLWNFTSTLNIETGKIFKPEQLKTQVLLIHDAVEVFAAASKKVK
VQPE-
>HassIR5
MWSYHVFLAVLYCWGQLVSADIDRRRFSNPTYYNVGGVLSSNESIAFFKDTISNLNFKDQ

YVPRGVTYHDYSMLMDPNPIKTALNVCKDLIAHRVYAVVVSHPLTGDLSPAAVSYTSGFY
HIPVIGISSRDSAFSDKNIHVSFLRTVPPYSHQADVWVDVLKHFNYMKVIFIHSSDTDGRAI
LGRFQTTSQSVDEDVDRKVVVEQVIEFEPGLDSFSDRLMDVKGAQARVFLMYASKTDAEI
IFRDATFLNMTTVGYVWMVTEQALDAANAPEGLLGLRLVNATNEHAHIQDSIYVLASAIR
DMNTSEEIHAPPSDCDNSGSIWTTGRLLFDYVRKQRLENGATGHVAFDDHGDRVHAEYD
MVNVRAQGEHVAVGKYFYSKETQKMRLELKEQEIIWMGRSTSKPEGFMIPTHLKVLTIEE
KPFVVYTRRIDDGSECTPEEIPCPHYNASEDTDQLYCCKGFCMDLLKHLSKAINFTYSLALS
PDGQFGNYIIRNFSQPGAKKEWTGLIGELVYERADMIVAPLTINPERAEFIEFSKPFKYQGIT
ILEKKPSRSSTLVSFLQPFSNTLWILVMVSVHVVALVLYLLDRFSPFGRFKLANIDGTEEDA
LNLSSAIWFAWGVLLNSGIGEGTPRSFSARVLGMVWAGFAMIIVASYTANLAAFLVLERPK
TKLTGINDARLRNTMENLTCATVKGSAVDMYFRRQVELSNMYRTMEANNYDNAEQAIED
VKNGKLMAFIWDSSRLEFEAAQDCELVTAGELFGRSGYGVGLQKGSPWADLVTLAILDF
HESGIMESLDNLWILRNNMLNCEENEKTPNTLGLKNMAGVFILVLAGIIGGIVLIVIEVVYK
RHQIRKQKRMEIARHAADRWRGAVEKRKTLRAAILPSQRRAKSNGVKEAGSISLAVERGA
RRRDEPRVPRYLPAYTPDVSHLVV-
>HassIR4
MRGANAIFLILFFGHLSALPDTIRIGGLFHPEDEKQEVAFRYAVERVNADRAVLPRAKLLAQ
VETISPQDSFHASKRVCHLLRSGVAAIFGPQSAPAAAHVQSICDTMELPHLETRWDYRTRR
ESCLVNLYPHPAALSRAYVDLVRAWGWRSFTIVYENSDGLVRLQELLKAHGPSELPVAVR
QLPDSHDYRPLLKQIKNSAESHIVLDCTTERIRDVLQQAQQIGMMSDYHSYLITSLDLHSV
DLEEFKYGGTNITALRLLDPERADVQRVVRDWVYDEARKGRKLQLGHTTAKENMTFIKT
ETALMYDAVHLFAKALHDLDTSQQIDVRPLSCEAEDTWPHGYSLINYMKISSPYCMRREA
SEKLTGNAQFEGYAIDLIHEISKILGFNYTFKLAPDGRYGSYNRETKEWDGMIRELLEQRA
DLAIADLTITYDREQVVDFTMPFMNLGISVLYRKPIKQPPNLFSFLSPLSLDVWIYMATAYL
GVSVLLFILARFSPYEWDSPRNCLDEPPVLENQFTLLNSLWFTIGSLMQQGSDIAPKAVSTR
MVAGMWWFFTLIMISSYTANLAAFLTVERMDSPIESAEDLAKQTKIKYGALKGGSTAAFF
RDSNFSTYQRMWSFMESARPSVFATSNKEGEERVVRGKGAYAYLMESTTIEYVVERNCDL
TQVGGMLDSKGYGIAMPPNSPYRTAISGAVLKLQEEGKLHILKTKWWKEKRGGGSCRDE
TSKSSSTANELGLANVGGVFVVLMGGMGVACVIAVCEFVWKSRKVAVDERKEEASLCSE
MASELRSALKCPGGGAGGGGGGPGGARDGAGSPYLHYGFSTKSQLH-
>HassIR3
FHYRLRLVPDNMYGARDPDTGHWNGIVRELMDRKADIAVASMTINYAREAVIDFTKPFM
NLGIGILFKVPTSQPTRLFSFLNPLAIEIWLYVLAAYILVSFTLFVMARFSPYEWSTSTHVCG
HETKLLTNQFSVCNSFWFITGTFLRQGSGLNPKATSTRIVGGIWWFFTLIILSSYTANLAAFL
TVERTVLPIQSAADLAAQHHIHYGTLNGGSTMSFFRDSNIDIYQKMWEHMSSASPPALVSS
YEEGVRRVLAGNYAFLMESTMLDHRVQRDCNLTQIGGLLDSKGYGIATWKGSPWRDKIS
LAILELQEKGVIQILYDKWWKNTGDVCNRDGKDSKANPLGVQNIGGVFVTLLCGLALA-
>HassIR2(2)
KIVQLPDGDDFRSQLKFIKKSGSVNYIINCKRQTLPLVLEQAQQVGIMSDEHSYLIMNPDF
QTIDIDPFKHGGSSITGIRMFDPSLESIQNFITSLNEKLTELSENEIENAIAENGLTLDLALVY
DAVTLFVSTLNAMSLEEGSNVTCDDAESWGFGSSIVNYARTMEVDGLTGIIKFDEDGFRSD
IEIDVLEIMSYGLDKVGTWTLEDGFVETKDNVAPAELEGSESMKGKHFVVLTALSAPYGM
LKESLKKLEGNDRYEGFGIELIDELAKINEFNYTFDIQEDGVYGSYDKVTGKWNGMMEKI
MDGRADFAITDLTITAARQKAVDFTSPFMNLGITILYKKPTKEPPDLFSFISPFSMGVWGWL

AGAFVGVSCLLFILGRLAPEEWQNPYPCIEEPETLDNQFTLANSFWFTLGSVLTQGSEIAPI
AVSTRMAGSMWWFFTLIMVSSYTANLAAFLTVESKFYAIKSVNDLASNPYGMTYGAKKG
GATFSFFKESDNLLYQKMYHYMEDHPELQTSTNDQGLDRVKSDSENYAFLMESTSIEYMV
ERNCDVAQVGGLLDSKGYGIAMKKNSPYRQPMSESILQLQEEGKLTRMKDKWWKEKRG
GGACADDDAGGGEAQPLVLANVGGVFIVLAAGSGMAVVCAFVEMVFDVWIISRKMKVS
FREELKAELKFILSFSGDTKPVRHRESTGSGSGGSKDADEKNADAESPVDDDRADPSPTPR
SERSGHSHHTLHSRRQSNAVQMAKMRKYSLRSAM-
>HassIR1
SFLEEPYLMQKPIEFGQKEELFGFCKDLMEVIAKKMGIKYKLKLSNDANYANDALPDIYT
GVVGEIMRKEADIAIAPFAVTPERERLVDFSEPFLTLDTPIAYTRTPRQLSDTFSFLRPLSKEI
WLCVLFSFFAVSIVLFLVSRFSPHEWKSVSISDTQLDHTMSS-
>MsexIR1
MLSAVVSGLPYDAFDLCLDYFLYRNVKLICVLDCEVNVGITKFTKTANSHRMLVTVADLG
QLNSSRVEQCLDQTLFPVGVVLNAGCPGTENTLLLVRILYELLTIINEATAGWALHPSKWR
SPLPQRNTSIASQKMLFNGNHLWLIIESNNEVDKNNITKSNDTLSPNVGLLETLNISVDADI
TVASKIDSSEYILYEVFNFGKIQGGDLIINRAGSWNLQLLMDVDINGYKYYRRWDFQQWQ
MRMIFVMSPIPKKLNPKVLTERTPTPGLTTITKTSAMSLHVIAEIHNFRFKYTMADRWIGTF
EANSTRVVANSLYFREQDVSPILRHQLYLYGKVDFINPPLTYIETKYYYRIPTKGPGKFQNQ
FLRPLSKGVWACLAAVSALCIVLLLMSSLLERRPSSAQYAVFSVIASICQQFFEDVDIGNST
RSSAARKLTILVTGLSCVLVHNYYTSSVVSWLLNGPPPSITSLQELMDSPLELLFEDVGYTR
SWLQTPNYYYNRRNAKIESELRAKKVFKRSSSSKLLVPLEEGIAMVKAGGYAYHTEPGRA
NEIISRMFTQRELCELGTLQSMEKTNLVPGVQTQSPYKEFLVWSTARLVERGVVSCVQRR
AASPSVQCDGSSPRALALGGAAPAFILLASGYGLATLIMFVERLIYSRLQNQINVTRCELFR
FK-
>MsexIR3
MSLRQDLLEFAYPIQPIYKWRFGFLLLLPEENNYFQLFYSKPFTRPLWNSLYIAAFIICIIFFL
LQLWEKRVVGKVQENGITYEMLVVMGSYCQHIPPLHSALSSRRTAYFVFLIFSYVIYSFYT
SNLLSHLVNDRKIVTDITTLSTNDFDFVIIDHAKFILEYYHPLPKGSLSALIKKLIGLESVTIL
DGLRKVKEGNCALLSDYITLNPYIRMAFNSDELCDLIQVDLFTDITKYFFTSKNFTYKEQI
KIGIQRINEVGILRRLLNENLAPVPNCHPTKHFQTKIIQIFVPLTTLLCAYGLSLLILLGECYY
FKRYRLFPYTD-
>MsexIR4
MIPGMCIITLYALFLIPGLIYGECPASMQATLPFMLEYAIWRGWHQAIVYTPHLEQDCKGSV
TRLVKCLNLNGIAVSENSLSSPRIVTKLGIIICPDLRRSTSEKFVKLIDKLQDLQMNTASYD
WLLTARFDEDFKYINSILNATSIRFDQNIAVTYPSRNVKSFSLGRELCDGNFLYKRNYVPSG
DPFSIQNRSRYCSYCGNGVKTFYDRFYYRTSERFKHLSNNRTYKHFGIVMPLYNSGLYYN
YSRDFNMTDIMKFSVNRFKPLKKFVNENYEQQGGIYILQYFKVRSNSTLYVSLLGIWSQE
ESMSKVEIPYPVEARSFLGEELIVGRCNSSFDGTTSVDEDVPTSPALLDDVLYFLTMRLNTT
PSLRYYNKLGFRTYEGSWTGLLGALIGNSVDVALEPVTAHSPRHQDMDFIFPVAETMCNIY
IRHQETSTVRDIFLAPFSTKLVGCVVGIAVIASLTAVVISKVSSKVRKNVHSLGYVESLIWST
GILCQQGSVWSPLNPAASILLIVCLFFAMVTYNAYAAFITSVLSVRVASVDTVAAVLHSPNL
KIGYIRNGADQMYLMSTKDTLLNAFYIRGYSNAENLVSSADEGLARAATQDYAFFAGQR
AARSTLRSLSQARGRCAVRELPVHTTRAHLAFPLAKNSPYAKPLLVSLLHLRTGGSLARLE
AALVPDMPYCATKAGFASARATDVRSAFILLGTGFVTALLLGFGEYCWKNKHQQKEFIKK

YYEQFLQVIEA-
>MsexIR7d
MLSKLFVLLKFVEFIYADTLSDTAAFLASSTYKLQYVTSVVWSSRRSEDATDFLKRFHGCV
VTNEIHNNNNLTTRLLNKAVGYKQTVLFVDSVEEFAYFINSVNLDLVIPIQLVLVLTTFVED
LSGVTRTAWQNDVADIIIVNQNFDGDISLTTFFPYGKGICGDYSPVVLKIDAEAFFPNKFDN
FYGCPIRTALMNFAPYLKVDMKNGTLNSIVGIDGRIFLYVMEALNATIDVVSIEDHDGMGS
FINGTPKGCFGDLVNKEADIIAPAAVMNYERYSRAQISHVYDTLDVHWCAPSRRIIFTWAK
VLLPLLDYITIYMFITFAVFVTVTAMATRYGMRRVRIKDSIIFQTYVILIGQIQKFETESYIVN
YLFVLWLWFCIVFRIAYQGDLIQGMHRTIQEPALGTLENALKYVDGYGGLEVFYNYYRDT
PLAANFQILEVHDIGPYIEAIAAGKKFLILTDILLVQEFDVKIQILDEPVSRAVTCIFMRPGWP
AAKEVDLLMQYIIEVGLTEKALKDYNYEWKRTRELRKEEYVHQSLDFLTLSACFYGLVL
MWMISSIIFIIEKTYYDDIKQKQSNDRSSRF-
>MsexIR8a
MKTGVRLSDSIVLLDRENEEESYRLLCSSVSKGVSMIVDISWFPWEKAEELSSVIGVPIVRT
LLGPQQLVTALDNYLESRNATDAAIIVESEIDVDKTLYELLGSSNVRVWVHAGLSRESAKA
LKTMRPEPSFYAIVGDSEFVFNTYKRAVKEKLVRRNYRWNLVITDYVESSYIEFSQLILPTM
FLQVDPAECCRVINQKDECSCPPMQKNQIILNSLIVYIVEVYSKLDDSTVTVRVDCEDLQA
ELNSTRDKLYKQFAEDTENNETIFYWIEDRSSLLLRSRFILYTYISDEGLTKVASWFAGENY
KLLPGVTLEPLKMFFRIGTALAVPWTLPKLHPDTGEQLVNEEGQPLYEGYCIDLIEKLSEA
MNFEYEIVTPKVGGFGKKLPNGTWDGVVGDLMVGETDIAVGALTMTAEREEVIDFVAPY
FEQTGILIVIRKPIRKTSLFKFMTVLRTEVWLSIVAALVLTGFMIWLLEKYSPYSARNNPDA
YPYPCREFTLKESFWFALTSFTPQGGGEAPKALSGRTLVAAYWLFVVLMLATFTANLAAFL
TVERMQTPVSSLEQLARQSRINYTVVESSSVHQYFINMKFAEDTLYRVWKEITLNATSDQA
QYRVWDYPIREQYGHILLAINASGPVPDAKTGFQQVNEHADADFAFIHDSAEIKYEVTRN
CNLTEVGEVFAEQPYAIAVQQGSRLQEDISRALLELQKERFLEQMASKYWNETARQACPD
ADESEGITLESLGGVFIATLFGLGLAMITLAWEVLYYKRKEKTKIHTLDVKPKPKPFAQTK
KNLEKLTGVVKLRKRDVAKTAKLGKDTVTIGDTFKPAKGVSYISVFPKNEYRP-
>MsexIR21a
MKCALVCILVFFQIVFNQEIEYYPSKASLGYKISKTISQTSKNNLITNLDDYKNAAGNKRK
KVWRNFNKDNENITEKVTVKRAVDPVFHGYPKTREELWNEHFLNESSAFDQTPSLIKLIH
NITLSYLKDCTPVLLYDSQVKSKESYLFQNLLKDFPVSFVHGYINDKNQLQEPKLLVPVRE
CVHFIIFLTDVKVSAKILGKQSDSKVVVIARSSQWAVQEFLSGPLSRRFVNLLVIGQSFKED
DDTTIEAPYILYTHKLYTDGLGASQPVVVTSWSHGKYSRNVDLFPPKMTEGYAGHRFVVA
AANQPPFVFRKVITDLDGGNPRVKWDGIEIRLLKLLAERNNFSIEIVEPQDLQLGPGDAVA
KEIAMGRADVGVAGMYMTIERAREMDVTFAHSQDCAVFITLMSTALPRYRAILGPFHWH
VWVALTFTYMFGMFPLAFSDKHTLRHLINNSGEIENMFWYVFGTFTNCFTFVGKNSWSRT
TKITTRLLIGWYWIFTIIITSCYTGSIIAFVTLPVFPETVDTIDQLLAGFYRIGTLDRGGWERW
FFNSSDPNTNKLMKKLELVPDVASGIRNTTKAFFFPYAFLGSQAELEYIVQANFSKTTSKR
AQLHISNECFVPFGVTMGFPNNSLYTAKLSHDIRAMFQSGIIDKIVDEVRWDMQRSSTGKL
LAASSGSLKIVSVEEKGLTLEDTQGMFLLLAAGFLIAASALISEWMGGFSRKCRFRKKDR
GTNSADSRELVSPDPDKAEINAVCDGTDSVLHFGTKSTSASSRDTLEGEIINVIEDSIIVHDN
MDTEQWGSRRSSSFDLDREVKEIFEKDSQRRGIPVDDVDIDNRLGTASHGAFGDHINSKY-
>MsexIR25a
MLSAKKTPHVVLDFTMTGVGSETIKSFTAALSLPTISGSFGQAGDLRQWRSLNSNQTKFLL

QVMPPADVMPESIRAIVTQQDITNAAILFDEFFVMDHKYKSLLQNIPTRHVITPVKSFNKD
EIKTQLRSLRDLDIVNFFIVGSLRTIKNVLDAADENQYFGRKTAWFALSLDKGDITCGCKD
ATIIYMRPTPDAKSRDRLGKIKTTYSMNGEPEITAAFYFDLSLRTFLAVKSLLDSGKWPND
MKYITCDDYDGKNTPNRTLDLKSAFQEVKETPTYAPFHIPEDDPINGRSYMEFSTDLSAVT
VKDGASIGSKALGTWKAGLANPLSLIDPENMSDYSAQLVYRVVTVEQQPFIIRDDAAPKG
FKGYCIDLIEEIRKIVKFDYEITLSPDGNFGTMDENGNWNGIIKELVEKRADIGLTSLSVMA
ERENVIDFTVPYYDLVGITIMMKLPRTPTSLFKFLTVLEHDVWLSILAAYFFTSFLMWVFD
KWSPYSYQNNREKYKDDEEKREFTLKECLWFCMTSLTPQGGGEAPKNLSGRFIIIASYTA
NLAAFLTVSRLDTPIESLDDLSKQYKIQYAPLNGSSAMTYFERMAAIEVRFYEIWKEMSLN
DSLSDVERAKLAVWDYPVSDKYSKMWQAMKEAGLPNSIEEALQRVRDSKSSNEGFAWL
ADATDVRYHVLTSCDLQMVGDEFSRKPYAIAVQQGSPLKDQFNNAILRLLNERKLENLKE
TWWNNNPNSMKCEKQDDQSDGISIQNIGGVFIVIFMGIGLACITLGVEYWWYKWRKRPL
VGDIMQVEPAKTTKDNVTKPESFNFRSRNLGLSNLKPKF-
>MsexIR31a
MLATITCDQSYLANIQQKIQGVLYINHYGDKLLSQVDRDYFSIQYKWLIVGEENVDLHNIR
YDADVTYVNRKLQISRLNKSEISQHEVIHFQDVYAHPDDGVSIHSWAYWMENKFVLTHD
RARTLRRLDLKGHPLRIATPTGLYTEETYNGTFSEYLADEKLLRMDSGIRSAHESSWLIIDR
LNATYVLLPTLLWSTEFHNNSMFVKLQNGDAELSGAILRVLLSRINRLDYIQPIWPFHVGF
SYLAERESSSNMFVEPFAVSVWWACLGMGFILVLAQKVTSRTRMEKDGAFFTVLATCLQQ
DASAVPEGLSGRWTFIVLSVSSMLVHAYYTSAIVSSLMSTGRSGPETLRDLADSRYAIASED
YDYMRYLFFDVKTNWDDLEYLKKRKMTSKLYQTTEFGVKLVQRGDTAFHGEFNQIYPYF
GTFTDDQICKLQYLDTLPESMTWVTTKLRGQWTNVFRSAGYWIQEVGLSKRLVSRLKAP
RPSCRAALLAERVKFGDVALLMMLTVFGAVLSLILLGLEILFAKWRKRNTPLVREGSSLSID
SVE-
>MsexIR40a
MYAREYLDFKTNAKVQEVRMRSEGDFLLTHDIHPTCGKSKHCKINNAAYSYININDALTH
NENPADVFYAPTVQATAADCSPARRSPWGISKNYISPHPYFRSSIEASQEHKASDNHSGAEE
FEMKQPLLITYCLILLLNINVVQLTTMFLTSILMFRIYYNQATPDRLNHLTLVNWWAGRLY
KSPVLPPADKVYQDFKGRIFDVPVLHAPPWHFVRYNNDSSVRVTGGRDDKLLALIAKKL
NFRYRYYDPPDRSQGSRISGNGTFKGTLGLIWKRQADFFLGDVTMTWERLQAVEFSFLTL
ADSGAFLTHAPAKLSETLAIIRPFRWEVWPLVCATLLVTGPALWVVIAAPSLWQRRQRDQL
GLLNNCCWFTTTFKEPSKTHKARLVSVLISLGATYVIGDMYSANLTSLLARPAREQPIGTLS
ALEEAMRDHGYELVVETHSSSLAILENGTGVYGRLAKLMRRQRVQRVRNVEMGVRLVLT
HKRVAVLGGRETLYYDTERFGSHNFHLSEKLYTRYSAIAFQIGCPYLETFNNVVMTLFEAG
ILSKMTTDEYKNLPEQARRSEPVTESDRESSDMTREPTATSQVQTESTKGLEPVSLRMLRG
AFCLLGIGHLLAGTALTLIIEIQLYRRAKKRREPVHVDTRKEALMKITTKMNRGICCAYRK
TKTVIFRYVDRALGPGAANGI-
>MsexIR41a
MTLTPAQFFSPLELLLHNLVVQYLSDSYCVSVVSETPLNFVFLPSFTYINPNDTDDNNLVEK
LLKVSEEGCSDYIVRFQNPQNFMNAFEKVNHLGKVRRSDRKLIFLSIPDVNYRNQALEML
SKVECSFVANILLVLTSDENQCGESFDFVTHKYVGPDSEVNEPLLLDHWNACTQQFDRKA
NLFPHDMTNMKGKTLKVSCFTYKPYVLLDLDPAVAPLGRDGMELRIVEEFCRWINCNVVI
INEDKDQWGEIYENQTGIGIIGSVVEDRADMGITALYSWYEEWQAMDFSISCIRTAITCLAP
APRILASWEMPLLPFSKLMWLAVVITFIYAAIGLLMAQKWSSNKALLIVFGILISQSQHKIG

DSWRVRSVIGWLLVAGMILVNAYGAGLASTFTVPKYESSIDTVQDIVDRNMEWGATHDA
WIFSLTLSPEPLVKKLVSQFRIHSFEELKKKSFTRSMAFSIEKLPAGYFAIGEYITKDAVLDM
QLMLEDFYYEQCVIMMRKSSPYTEKISQLVGRLHESGLMLAWETQVALKYLDYKVQVEV
KLSRSKNDVSVTEALNIKLVEGVFILYLIGVISAVILFIGEVLMEYYKSRVHAVIEI-
>MsexIR64a
MNVNFKLEFVLRHCLPDKIEIMVQKLVGTRLSKGILDHTAMNDWLNTCYFTKALTEELLQ
LTADGVPVCVVRPFTVKELSQHPIEIDSDTTIADKTENVYDLYEIYNTGFKKNGIFKTVHIG
DWRSSLNIEHPKRRNDLSGVVLSCPVVVLKKLEEETFEHYLMQATHVGFDSLHKLKYITL
LNYLKDMYNMTYDLQRTNSWGYIRNSSFDGMVGSLQRGQTDIGGSPIFFRADRNEIIDYT
AEVWHSRHSFIFRHPKHPSGLYTIYTRPLSTIVWYSVLALLLFSTVVLCVIFKLSWSQSSDS
SFSLALLFLWGALCQQGTSLVQQSTSMKILIFSTFVYSAILYQYYTASVVSTLLMEAPKNIK
TLKDLLESNLRIGVEEALYDRDYFKRTTDPVALEIYKRKVKQGASENFFLPEKGMALVKM
GGFAFHVDTIVSYGIVKKTFSEREVCDIQDVPMYAPHRLGAVMKKHSPYRDHLAYGIRKI
METGLMYRIKWAWDEPRPSCVKTPDSSIYSVSILEFSTPLIILAFGYWLSFVMLVLEIVLCCI
TQRNVQKRKGFEVSKIY-
>MsexIR68a
MCTMFGLCLCAVLLVGVPSSEATSPILRGVAHNKELRWVLIDILHSLRPRDITCIAVISDSVY
LNVFDGELFRIITNVPFVMIIVAEEEDLLSPNYITLKSLRQTKTDGCNVYIIFLASGLQVARL
LKFGDRHRILDTHAKYIMLHDIRLYHKDLHYLWKRIVNVVFIKYHRKIIGNAKSRPWFDL
LTVPFPNPIKSVLVPRRVDIWRNDKFHYNRSLFVDKTYNLYGQQLNAVYLDHVPSVVVTR
DNETSKIGGVEVEIFKTLSELMHFEVNMYQPAHAELNKWGQKQANGSFSGLLGEMASGR
ADAALGNLQYTPYHLELMDLSIPYTTQCWTFLTPEALTDNSWKTLILPFKLNMWIAVLLV
LLITGTIFYAMARYYMNLQDYRHDHDKRPFANALNQHPYRTECRLHIYFADAKPVGLYVF
GEIINSILYTYGMLLVVSLPRLPTGWSIRLLTGWYWLYCILLVVSYRASMTAILANPAPRVTI
DTLQELADSKVTCGGWGIETKKPEISDRFETINDPNDAANRVAQGVYAYYDNSNFLKYIS
VKRKSGLTMESDNATTNGSLVPPKPHEERDLHIMSDCVVNIPISIGFHKNSPFKPLADIYIRR
IVEVGLVEKWLNDAMEPIRALEVNDEEIKALMNLKKLYGAFVALAIGYFLSGICFLLEIIH
WNCMVKKDPMFDKYAIDVYYRNKNKK-
>MsexIR75d
MPYGTRAYHLSVTLGNMANPTACLAGIWLKPMPVKFLAAMLTVNLVPMGYLRDENRPH
SIYNYMPSPEDLVQLWRIAQQYGMRIDMRPDFQRLPDLLAYDFHREGVVLDVTCPGTELL
LRKASSNRAFIHRYSWLLLHNSSYDPTVIHSTLNTTSILPDSDVGWSSPDNLVDVYRVRPD
KPLVLTTLGLPRDSSCEDLSKLWREMPTAATRRKDLKNVYLTAATIITQPQFFKGWSDLRD
RQIDTYPKFTYPIMMLCAEDLNFRYNLKQVDFYGEDNNGSFNGLAGLLQRRDVEVGVTS
MFMREDRWRVLHYCSETVELVGAFMFRQPAQSAVSNVFALPFSRGVWAASGVVFLAAAL
LLATITYRLRREDHALQPLSLPECFTFAIGTVCQQGFYTFPSSVSVRVVMVSTLVAALFTFT
AYSAKVVAILQAPSDAVRTIADLARSPMALGVQETTYKKVYFAESKDPATQFLYRRKLLPL
GEGAYLSVVDGIARMRAGLFAFQVEQGSGYDIISKTFTEHEKCGLKEIQAFKLPMVAVPIV
KHCGYRELIASRLRWQREIGLMGRWRRRWLASRPQCDSAAGGFLSVGISDVLPAIEVLISY
TNN-
>MsexIR75p.1
MNTDQTSVLVKMLNEFGISTASSLQPNRSKYFLQYVLIVVDLSCPKIEEFLVEANAQGYFK
SPYRWLMINSGKASKILEKLDPLVDSDVVIASEVGYGFLFTEAYKIATNSNIIYNKRCFWTE
HNHDEYFTNGNVYTSNSTVITNNKELDSKTNKSKISMKIDKYGVTEDYRKSKVLSTRRSD

LKRNPLTMVNVITDSNETRKHLDDRLNLHQDSISKMSYAVVKICFEMLNATEKLMFTHTW
GYRDKNGNWQGVVDYLIKKEADLGTLTIFTQERMDVVDYIAMVGSTAVRFVFREPPLAYI
SNIFTLPFTGTVWMAILICVLGCSVFLYITSKWEATMGMHPLQLDGTWADVLILIIGAVLQQ
GCNLEPRYAAGRCVTLFLFVALTILYAAYSANIVVLLRAPSSSVRTLPDLLNSPIKLGASDFE
YNRYFFKKLNEPIRKAIYDKKIAPKGKKPNFYTMEEGVERIRQGLFAFHMELNPGYRLIQE
TYREDEKCDLVEIDYINEIDPWVPGQKRSPFNNLFKINFLKIRESGIQANIHHRLHVPKPRC
SGTVSTFSSVGITDMYPAMLATLYGMMLAPAVLLLEITYKKLMIVRQNKKVSTENLYITPF
VN-
>MsexIR75p.2
MIVLTLLFTGLVRASDDFNVQFITNFVINERKPTFLVYAGLCWKKHDEIKLTKALNKLGVR
TSSSLEVKSKFHENAVMFLTDLACDKSEEVIATATSRRLFQFSYRWLVLSNSTDALKMSLL
EASPVLATSDLVLAERRDEMYTMTELHKPANNSTMVSTLRGYYNGTFSDVRPRRELYWR
RRDLMWHPITMANVIQDSNSTKYYLPRDDRLELQNDAVAKVCWVFAKIAFDMLNATPQY
IFSYRWGYKVNGKWSGMIHDIGENRADLGTNCVLFKDRLDMITYTDLVAPMRMRFVFRQ
PPLAYYVTNIFALPFSSGVWLAIAVCSMVSAAALYLASIWEVKIEKSPTQLDGSIGDALLLTV
SAVAQQGCFIEPRRSPGRIMEWVFFTALMALYAAYSANIVVLLQAPSNSIKNLAQLADSKV
TLAANDVDYNRFVFNDNRDQLHISVHNTISPENGKPHFYGIFDGVERIRKGLFAFHSIVEP
VYRRIEQTFLESEKCDLTEVDFINSLDAFTPVKKDSPYLELLRVVYKQIRESGLQAALNKR
YQVPKPKCLNKIAAFSSVGLLDIQPVLILMLYGVALSVAIAVCEIIVFKIANCCSKKKKIKIV
HFKN-
>MsexIR75p.3
HKPSPNGSMVTTPRGFYNGSYVDIRPHRELFRRRGDVMGHPLTMSNVIQDSNSTQYHLPR
EDALELNYDVTSKICWMIARLAFEMVNATPRYIFSYRWGYQVDGQWSGMINDLKNGKA
DLGTNCIVTDTRRLSVVSYTDSVAPYRVRFIFRQPPLAYVANIFSLPFSSNVWIALVICCFAS
TAAVVLGFKWEANLDKDSQYLTGNIGDALLLTMSAVSQQGCVIEPRRAPGRIMLVVLFTT
LMALYAAYSANIVVLLQAPSNSIRNLAQLTASKITLAANDVDYNRFVFNLYKDPVHQSVN
KKINPDKGKGQFYTIDEGVEKIREGLFAFHSIVEGVYRRIEQTFLEMEKCDLTEVDFLSSFD
PFVPVKKDSPYIELLRVTFKQIRESGIRAALNKRYQVPKPPCHTKSAAFSSVGLLDLQPVLV
LMLYGIAISTAILAVEVAVFNM-
>MsexIR75q.1
MTGYLVRGEVEIGGSPMFFTIERIPYVDYISSPTPTRSKFVFQEPKLSYENNLFLLSFRTTVW
YSTLALIFILFLALFLVTYWEWKARRYIESRETDSAILRPNIGDVVILIFGATCQQGSPVELK
GSLGRIVLLILFLTLMFLYTSYSANIVALLQSSSSKIRTLDDLLHSRIKFGVHDTVFNRYYFS
TATEPVRKAIYEKKVAPPGSPAHFMTLEDGVKKIRKGLFAFHMETGVGYKFVGKYFEEGE
KCGLKEIQYLQVIDPWLAVKKDTPYKEMFKIGISSESEWYSEDYGIWSKPYGLNKSSEINF
SATIRRKDLRGLTIVTSLIISDNRTKYELYDFRNLLVDTVTKTTVRHLTPLYHFMNATKAFV
FTDNWGYYINGSWNGMIGDLARGDADLGGSLAFITKERLTVVEYLSHPTPITAKFVFREPP
LSYQHNLFLLPFKTTVWCCIGAFIVLVLLILYINAHWDIKKFQHYRQKTMDFTALRPNLSDI
TIFLISAMAQQGSHQELKGTLGRLVMFILFLTFVFLYTSYSASIVALLQSSSNQIRSLNDLLH
SKLELGVEDVPYNKYYFSTATEPIRKAIYETKVAPKGSRPNFMNIDDGVKKLRNEPFAFNM
NTGIGYKYVEKYFLEHQKCGLQEIEYIENNTPWLPCKKHSPFREIYKVGLFRVNEHGLGD
RENQLVYAKKPACTARGGNFGSVNMIDFYPVLLLLLYGMITAFLLLFLEIFIHQKQMALTLS
WNTAL-
>MsexIR76b

MAGIELIISSICNATFCNAVYDNPITDSQLTRKQMVYNDLAKQINGKNLKIATYNNYPLSW
VERSANGTLYGYGIAFDIVEILKKKFNFTYDVVVPEKNYEIGGTKPEESLLGLANSSQVDM
VAAFMPKLIVFQKMVTFSTDLDEGVWMMMLKRPQASAAGSGLLAPFNNLVWYLTLASV
LSYGPCITLLTSLRSKLVGDKERTLPLSPSFWFVYSAFIRQGTNLAPEANTTRILFATWWIFII
LLSAFYTANLTAFLTLSKFTLDIETTRDLYKKNYRWVSAEGSAVQYSVTNPNSDLYYLNKM
INSGRALFRNFRHNFEYLPMVKSGSVLVKEDLAIDHLMYNDYVQKTREGMSESDRCTYV
VAPTAFMKRQRAFVYPLGSILENLFNPVLHYILQAGIIDFLKATDLPLTRICPLDLQSKDRQL
RNTDLMMTYFIMAIGLAAALAVFIGELIIKRYSRVKIKTLKRKRPKRAKASKPDDSRPPPYE
SLFTKTQHSSRKSHKQIINGREYYVYQNGVDGTRLVPVRAPSAFLYR-
>MsexIR87a
MCGKMRAQFFLLFTIHVASAAGENPLLMATRNSDQIAKTAECVLKLSAKYFVEKXALSGS
IVIININSYYSTTQRLLLQTIHGVSEPYVMPPVRKIVNADYDDVYEFNKGAEINLVKIICQFT
NMTLVVRTSDREENWGTIYPDGTATGAYGVLRNGTTDLVIGNIEVTRTIRKWFHPTVSYTQ
DEMTWCIPKARQAPTWDNLVIIFQWQTWLATLISLLIMGLLFHYFYYKEHDNSTKWPTNS
ILMTFAMLLGWGANFDPKSMTFRILIFGWLCFSINMSISYESFLRTFLMHPRFEKQITTEAE
LIQSSLSIGGREIYQQHFKNNNASSFYFYRKYRTTTFIDGIRRAALERNFAIVSARRQAEFQ
DQKLGRGAPLIYCFPEENNLYKYGVVLLARKWFPMLERFNMIIRSVSENGLIDKWNEELFI
HTASGEGASAIVPLSIQHLLGAFMFIGFMCVISVFVFLCELLSEYYGRKRKVIKCRPYIP-
>MsexIR93a
MRLKLVGFLCLVCRVSGEEFPSLITANASIAVVLDRQYLGDKYQAVLDELKDYIKELARVD
LTHGGVVVHYYSWTSISLNKGFLAVFSVASCLDTWDLFSRTEEEELLLFALTEVDCPRLPL
RSAITVTYAEAGEELPQLLLDLRTSNAFKWKSAVILHDDTLNRDMVSRVVQSLTSQIDDES
ASPVSVSVFKMKHEINEYLRKKEMHRVLSKLPVKTVGENFIAIVTSDVMTTMADTARELL
MSHTMAQWLYVISDTNIHNSNLSGLIRALYEGENVAFIYNQTDNSPDCKNGIMCYCQEIM
NAFISALDAAIQDEFDVAAQVSDEEWEAIRPNKIQRRDMLLKHMQQHISTKSRCGNCTTW
RALAADTWGATYRHFTEDDILKENDNGTEATGVIEKVTLLDVGFWRPIDAMTFFDVLFPH
VQHGFRGKELPVITYHNPPWTILHTNESGAIVKYGGLMFDIVNQLAKNKNFTIKILLPGNV
KNEISNETDALHSRRAMLALAAIAKGQAALAAASFTILPNPTPGINYTIPVSTQPYAFLVAR
PRALSRAMLFFLPFTADTWLCLGLAVITMGPVLYIIHRMSPYYEAMKITRQGGLATIHNCL
WYIYGALLQQGGMYLPRADSGRLVVGTWWLVVLVVVTTYSGNLVAFLTFPKLEIPVTSIA
ELIENRALYTWSINKGSYLEMELKNSEEPKYKALLKGAELTKPTHSSETNAHAGVEDFMD
ERVAMIVPAGSPYLALLNKEINRMHKAGLITKWLSAYLPKRDRCYSMSSMAAEVNNHTV
NLNDMQGSFFVLLLGDFFFIVLNDETSLPFV-
>MsexIR143
MLQRIFLFCCITLVCSTLLDEKKSNTSELSFGSISKCISEFCKISLHFRSTAILEYGNNETVEK
HLFFNEIIKTLSSDGIQQIIIEDLTKIKNIEVAEADWMVVVYIYHCESLSKFNNAMMHDKIK
YLIIVPDHLETECKDDFIDVGNKVAYYDVTFIFNNNEEEDNTETYTISTFIREVDEATCKEIV
HGFINNNCQNGILESNIVFPNKNPTNIKNCPLNIGMGTLYPFSIIQDRDILKTFDKLNESQIR
GSDLELVKIAAKQFNATLNYNYVRKKEENPIGQYDFIPFIMNGSLDICAGGFYRIYGDVVS
YSGIYARQAVKWMYTADRKDKSWQNLVHKVNGLYIFIIYYVAYSCVWWLIRKFDKQSVS
ATTTLIFAWGALVGAGSLQDTRSLKQKIVNLMYLIMCLHLSAYIAAQLYSFYTISEPPERLE
TINDIMESGRPTYLVPVVKYFVIDENFRKYANQSKECDTFYDCFEKSLIYKGMTLLLHAFF
ANYQAKSAVGDEARILCTTQNVLTVYYEMLIRKNSPYVSKYQDIIVRLFEAGIPEKLYIEAI
GLTVVGRAESASKNIIANSYSCQTGCTVTIRQLDGAFYIWLVGCGVSFCVFIVEIRKKARR

MSLHLI-
>MS|comp100717_c0
PAVSSVRVVMFSTLLAALFVFTAYSAKIVAILQTPSDALRSIADLTRSPMVVGVQETTYKKV
YFQESPDEATQQLYRRKIEPQGERAYLSVVDGIARVRTGFFAFQVEQSAGYDIIKQTFTERE
KCSLKEIEAFKLPLVAVPMRKHSGYRELFASRLRWQREVGLMDRERRIWLVSRPRCEASSG
GFVSIGIIDVLPALQVLALGAFLSVVLLASEVG-
>MS|comp104026_c0
LLDVDTAIEPKGRDGVEIRIVEELCRWMNCTVEIVREDVEQWGDIYPNETGGIGVLGSVV
EDRADVAISE-
>MS|comp119947_c0
FPTTNHLLDFLNAKRKYVFADTWGYRVNGTWNGMTGYLLRGEVEIGGSPMFFTFERVSI
VDYISSPTPTRSKFVFQQPKLSYENNLFRSE-
>MS|comp1203294_c0
KSFTRSMAYSIEKLPAGEYITQEAMLDMMLMLEDFYYEQCVVMMRKSSPYTEKVSQLVG
RLHQSGLLLAWETQIALKHLNYKVQVEVR-
>MS|comp124776_c0
KKYEKYSEQTLDQTCLPPTWSDITIFLLSAISQQGSSNELKGTLGRLVMFIIFLAFVFLYTSY
SANIVVLLQSTSNQIRTLPDLLNSKLELGVEDVAYNRYYFSAAYTSKDPIKKAIFERKVAPK
GKPNYMSIEDGVKAIQK-
>MS|comp129582_c0
KEVMLGRADIGIAGMYLTSDRVRDMDVSMAHSHDCAIFITLMSTALPRYRAILGPFHWHV
WLALTFTYLFGMFPLAFSDKHTLRHLLHNSGEIENMFWYVFGTFTNCFTFLGKNSWSKTN
KITTRLLI-
>MS|comp131374_c0
INLNGYKYYRRWDFQNITMRMILVAQPAPKHLDLELLAGPSPVPGVAMITQTCATVLYEVA
VMHNIKYFPTITDRWIGTNERNSSKVVSNSLYFREQDLSPVIRFLKSVQENSDVLSPSLTAIE
TRYYYRIPTVGPGKFE-
>MS|comp135198_c0
FVGKYFNEGEKCGLREIQYLQVIDPWLAVRKNTPFREMFKIGTKRIQEHGLQYRENLLMY
EKRPKCS-
>MS|comp136712_c0
KRPFAFNMNTGTGYRIVSAFFREHEKCGLQEIDYIQNSKPWLCSQKYSPFGEMFKVGYIRI
QEHGLSDRENRLIYAKKPVCSVWEEASTQSIWWISILCVFYCCMERFWLLSCLALRFWCI
VCRREE-
>MS|comp138392_c0
WAAVAFTYFYASIGILTAQGFATTSYPFLKSFGMMIGQSQYQNVASNSWKIRSVTGWLLIA
GLILSSAYGAGLASTFTVPKYEPSIDTVQDIVDRDMEWGATHDAWIFSLTLSTEPLIKQLVN
QFRIYSFDKLKEKSFTRSMAYSIEKLPAGNFAIGEYITQEAML-
>MS|comp1582011_c0
SPYLELFRVVFKQLRESGIQSAVSKRLQVSKPHCSSKMSSFSSVGLMDMKPVLIFMLYGIC
LSVAIAVAEI-
>MS|comp159644_c0
MIIDLSWSPWRMAEDLASETGLPLVRTLLGSQQLVKALDDYLESRNATDAAIILESESDVD
RTLYELLGASSIRVWVHAGLTRDSAKALKTMRPEPSFYTIVGDSGFVMDTYRRAVKEKLV

RRSYRWNLILTDYSTPDVTQLVLPTVTLQADQVECCKLMKREDCTCPSDFQRKQYILNGL
IQYISETYSKLERDLPLTTTSVDCEDPQPVMNSTRDRLYRQFAEDSEISNETLFYWEAERSG
LFLRSRFILSTYSLEAGQQTIATWSADDKYKLLPGVELEPLKMFFRIGTAPAVPWTLMKVD
PNTGEQMFDEDGQPLYEGYCVDLIARLSETMSFDYEIVSPKSGDFGKKLPNGTWDGVVG
DLMRGETDIAISALTMTAEREEVIDFVAPYFEQTGILIVIRKPIRKTSLFKFMTVLRTEVWLS
IVAALVLTGFMIWLLDKYSPYSARNNPDAYPYPCREFTLKESFWFALTSFTPQGGGEAPKA
LSGRTLVAAYWLFVVLMLATFTANLAAFLTVERMQTPVSSLEQLARQSRINYTVVEGSSV
HQYFINMKFAEDTLYRVWKEITLNATSDQAQYRVWDYPIREQYGHILLAINASEPVPDAK
TGFQQVNEHTDADFAFIHDSAEIKYEVTRNCNLTEVGEVFAEQPYAIAVQQGSRLQEDLSR
ALLELQKERFLEQLASKYWNESARQACPDADESEGITLESLGGVFIATLFGLGLAMITLAW
EVFYYKRKEKNKVQAFNAKPEKVAFESKSTLESKVAESMAKLRKRAKVGKKGANIAKN
VTFGDSFKPVSEKGVSYISVFPKEYRP-
>MS|comp161214_c0
MAGIELIISSICNATFCEVPYNETYKGSGTDPMAEKDTNFMNLMKEVNGKVLKVTTYNNT
PLSWSEYHNGTMVGRGVAFTVMDILRKKFNFTYEVVLPKRNYEMGTKMTGDSVIGLLNS
SKVDMAAAFLPTLIAYRERVSFSIDLDEGIWVMMLKRPKESAAGSGLLAPFNELVWYLVL
AAVLTFGPCITFFTRVRSKLITDDEGVLPLKPSFWFVYSAFLKQGTNLAPEANTTRVLFVT
WWLFMILLSAFYTANLTAFLTLSKFTLAIEYPRDLYQKNYRWVASAGSSVEHVVKSEGEEL
YYLSAMISNNKAQFLSVISDTDFLGIVKKGAVLVKEQTVVDHLMYNDYTSKKDVEESEK
CTYVVAPNAFMKKQRAFAYPVGSKLKSLFDPVLTQIFQSGILDFLKRADLPSTKICPLDLQS
KDRKLRNSDLIMTYLVMVAGSATAVAVFAAEIFIKRYVSGKIITSKKLKKKKSKMSKKSTN
YDDSRPPPYDSLFGKNPRFNVETTRTKIINGREYYVFETASGEKKLIPARAPSSFLYRSDK-
>MS|comp165699_c0
RPTPDANSRDRLGKIKTTYSMNGEPEITSAFYFDLSLRTFLTIKSLLDSGKWPNDMKYITCD
DYDGKNTPNRTLDLKTAFQEIKETPTYAPFFIPQDDPMNGRSFMEFSTELLAITVKDGASIS
SHSLGSWKAGLSSNLTLTDPNNMSNYSAQLVYRIVTVEQRPFIVRDDKAPKGFKGYCIDLI
EEIRQIVKFDYEISLAPDGNFGIMDENGNWNGIIKELVDKKADIGLSSLSVMAERENVVDF
TVPYYDLVGITIMMKLPRTPTSLFKFLTVLENDVWLSILAAYFFTSFLMWVFDKWSPYSYQ
NNREKYKEDEEKREFTLKECLWFCMTSLTPQGGGEAPKNLSGRLLAATWWLFGFIIIASY
TANLAAFLTVSRLDTPIESLDDLSKQYKIQYAPLNGSSAMTYFQRMANIEEKFYEIWKEMS
LNDSLKEVERAKLAVWDYPVSDKYSKMWQAMEEAVLPNTIEEAIQRVRDSKSSSEGFAW
LGDATDVKYHVMTSCDLQSVGDEFSRKPYAIAVQQGSPLKDQFNNAILQLLNKRKLEKLK
EIWWNNNPETMKCEKQDDQSDGISIQNIGGVFIVIFMGIGLACVTLGVEYWWYKWRKRP
VVGDVVHVTQVEPAKSSRNNVDKQGEGFNFRGRNLGLSFKPKF-
>MS|comp1872421_c0
IDKITDEVRFEMQRSLTGHFLAAGSGTIKIPSAEEKGLTLEDTQGMFLLLAAGFIIAGTALVS
EWMGGFTRRCRFTRKVDTPISVNSG-
>MS|comp2100871_c0
SVNSGEHLIPTPKTDIDSEIRIIGDTESRLNFDSRPSTAVSTDTLDGQIINVSEDNFDVHNTFN
VSRFDS-
>MS|comp395436_c0
FAETWGYPINGTWNGMIGDIKRGKVDLCGIVTFITLERLPILEYLNQPTPVTAKFVFRQPPL
SYQNNLFILPFTSNVWM-
>MS|comp50464_c0

SFISLIPNSESLVMQIFNVSEMGCSDYIVRMQEPQKFMEAFETVVHKGNVRRSDRKIIFLPY
DEEYNEEYDVDLPSLVFSMKGSAYV-
>MS|comp634464_c0
KRRRFVSHDMESLDENRSTVSRAAFGDPIKHDK-
>MS|comp71302_c0
GGSNFVSVSMVDCYPALVILSYGAIVALFLLGLEILVYKREKILRKLSCVRHHNYE-
>MS|comp82009_c0
GRLQRQEAEVGLASFFIRPDRMRVADFISETCVLACTFIFRQPSRSAVSNVFLAPFSGGVWA
ACGGVA-
>MS|comp849777_c0
DTIKQLLAGFYRVGTLDRGGWEKWFLNSSDPDTAKLLKKLELVPNVEAGIRNTTKAFFWP
YAFLGSKAELEYIVQANFT-
>MS|comp88910_c0
EDHPDCKNGIMCYCQELMDAFVSALDAAIQDEFDVAAQVSDEEWESIRPNKIQRRDMLL
KHMQVKKGLVVHMINSTFFEVMTSKKS-
>MS|comp99629_c0
VTKWEWKKTSHLIESRKRDAGVLRANGVDVIVLIFGAACQQGSPSELKGSLGRVVMLVL
FLTLMFLYTSYSANIVALLQSSSSHIKTLDDLLHSRIKFGVHDTVFNRYYFSTATEPVRK-
>PxylIRd1
MAGIELLISSICYNATFCDAYYDNSGLDERLTKKQNEFLALRSEVNGKHLRIATYNNYPLS
YVEEINGTLVGQGVAFVIVDILRKRFNFTFDVILAEKNYESGGSKPEDSVIGLVNSSIADFA
AAFLPILYDYQTKVSFSHLLDEGVWLMMLKRPKESAAGSGLLAPFEYEVWYLILAAVLSY
GPCITLLTKLRNKLVTDEEPPIPISPSFWFVYGAFIKQGTTMSPEANTTRVLFATWWMFIILL
SAFYTANLTAFLTLSKFTLDIEYPQDLLRKNYRWMAQEGGTVQYIVRDPNEELHSLNVMV
KNGRAEFRAISNDFDYLPLVQGGAVLVREETAVLHLMYGDYLRKAREGVEEADRCTYVV
APNAFMTTMRAFAYPRNSTLRRLFDPVLNYVEQAGLIKHHLHRDLPSTKICPLDLQSKDR
RLRNSDLLMTYLIMLAGLAAATAAFMGEMIFKRYIRVKWKVKVHSGLIGQKKKSQSKKK
DTKKRWTDDTKPPPYESLFGRNSRYTGSENTEHKVINGREYWVVNTISGDRRLIPIRTPSA
FLYERK-
>PxylIRd1-like1
MNSEASANFAAASDALAQLYRNASVAGIRLVVTPFPRVAAGPRQPPLSPDGLDHLQGNVL
DLACPGADEVIRKASETRSFNQRNAWLLLHDAPYNITELEASMSSIAILPDADVILFTSDIT
VDFYRVTVHEPLQISELDISRNSSEQELAQFWLQRPSVVTRRRNLNNITVKAALVVTQPES
FVGWDDLTTKGIDTIPKVTYPLILLLAQDLNISFDFTQVQSYGDLVNESFHNSAIGLLEQSRI
EVAATSMLLRRDRMRMTHFVSESADLKFFRPAFIFRQPPQSAVANIFLLPLSRGVWIASFIV
FLLVAAFLSLFSRRLMGLDPSLEVITPGETLTFAVGAVCQQGFFMTPNLQSVRVMMFFTFIT
SLFLFTSYSAKIVALLQSPSNSILTVKDLAMSPLSCGVQDTSYKKTYYIVELSAGFDIISKTF
TESEKCGLNYVDPFRLQAVGIPIKKHSGLKELVSNRLRWFRDTGLMDRVRRIWSVQKPRC
DSASISGVVTVGLTDCLPAFQVLMCGAAGSVAILFMEVSVHK-
>PxylIRd1-like2
MQVWTAVVVSLCVTWRLVGAEDFPSLITANGSIAVVLDRQFLGEQYQLVLDQVKDWVRE
LARVELKHGGVVVHYYSWTTISLKKGFIAVFSIASCQDTWTLFSRTEEEELLLFALTEADCP
RLPSDAAITVSYMEPGHELPQILLDLRTTKAFHWKSAVILHDDTLNRDMVSRVVQSLTSQI
DDEDVPAISVTVFKMKHEINEYLRRKEMHRVLSKLPTRYIGNNFVAIVTSDVMSTMAETV

RDLGMSHTQAQWLYIVSDTNTHTGNLTSLINALHEGENLAYMYNITDDHPDCKNGLICYC
QEVLKAFVSALDAAVQEELEVAGQVSDEEWEAIKPSKLQRRNMLLKHMQQHISVNSRCG
NCSSWRALAADTWGATYRAFTEDTAAPGKKKDADNDTTSGVIEQIELLQVGTWRPVDG
VRYHDALFPHVEHGFRGKPLPIITYHNPPWTFLHTNESGTIVSYSGLIFDIIDQLSKIKNFTA
KILLPGNVKHDFSNDTVDSMQSESALATLSAVARGQAALAAAAFTILSDPMPGINYTVPVS
TQPYCFMIARPRELSRALLFMLPFTTDTWLCLGFAVILMGPTLYIIHRLSPYYEAMGVTPQ
GGLSTIHNCLWYIYGALLQQGGMYLPRADSGRLVVGTWWLVVLVVVTTYSGNLVAFLTF
PKQEQPVTTVAELVGQRGTFTWSIRKGSYLESELKNSDEQKYVTLLKGAELLTSSPEIGLA
GSGARVLHRVRTQRHALVDWKLRLSYLMREDHLRTDTCDFSLSAEEFMAEQVALIVPSGS
PYLSVINKEIMRMHKAGLISKWLSAYLPKKDRCWKSSAVAQEADNHTVNLSDMQGSFFV
LFLGFFIASSVLLLEWLYKRHKKKSEEIVIKPYVE-
>PxylIRd1-like3
MHIKSANGKKKKKKKKRSGKKGTGDTEVTQPDSFVGWDDLTTKGIDTIPKVTYPLILLLA
QDLNISFDFTQVQSYGDLVNGSFHNSAIGLLEQGRIEVAATSMLLRRDRMRMTHFVSESA
DLKFFRPAFIFRQPPQSAVANIFLLPLSRGVWIASFIVFLLVAAFLSLFSRRLMGLDPSLEVIT
PGETLTFAVGAVCQQGFFMTPNLQSIRVMMFFTFITSLFLFTSYSAKIVALLQSPSNSILTVK
DLAMSPLSCGVQDTSYKKTYYIDNPHPATKVLYERKLRAQGERAFSYTAEEGVARLRSGQ
FAFQVELSAGFDIISKTFTESEKCGLNYVDPFRLQAVGIPIKKHSGLKELVSNRLRWFRDTG
LMDRVRRIWSVQKPRCDSASISGVVTVGLADCLPAFQVLMCGAAGAVAILVMELAVFKM
FPVNEYRNEYSPSIGGLRKLLYICENFAHRNGMMYNVKKCEMMVFRFRGGPERVPPVYM
YGSAIRVVKQFKYLGHILTESLSDDADMERERRALAVRCNMLARRFSKCSRDVKIVLFKA
YCQSFYTCQLWTNYTRRAYIILRVQYNDALRILLRLPRYCSAKAMFADARVPDFFAVMRQ
RAAGCWDRVRSSSNELIAAINQDIYGNRFMIHWSCLHQSANKK-
>PxylIRd2-1
MKVTFDIIVISIALLFQVYVCEVVEYYPSLSKLSKPLQEKQKERQSIKYVPNKTANGHASD
KKPKNKPINKREVDTAFRGHPKTREELWNEHFLNKSSAFDQNPSLIVLLHNLTLRYLNDCT
PVILYDSEVASEKEHLFKDLFEDFPVTFVHGKIDTDDKLINPDLLKPVRECVHFIVFLTDLRT
TSKVIGMQASSKVVIVARTSQWAVQEYLVSSLSRKFVNLLVIGQSFKDEDDKLEAPYILYT
HELYIDGLGASRPVVLTSWSHGKYSRDVELFPHKMQQGYAGHRFIIAAADQPPFVVRRVK
SDLDGGNPRVIWDGIEIRILKLLGERNNFSIEIKEPQELHLGSSDAVAKEIAMGRADVGIAG
MYLTNQRIQEMDMTAAHSQDCAVFVTLLSTALPRYRAILGPFHWHVWVALTFTYLIGIFPL
AFSDKHTLRHLLNDSGEIENMFWYVFGTFTNCFTFVGKNSWSKTTKITTRLLIGWYWIFTI
IITSCYTGSIIAFVTLPVFPETIDSIDQLLRGFFRIGTLDHGGWERWFFNSSDPKTNKLLSRM
SFVPNVEAGIRNTTKAFFWNYAFLGSKAELEYIVQANFSLTKSKRATLHISNECFVPFGVSI
GFPNQSLYTAKLSGDIQRMSQSGLLNKIVDEVRWEIQRSTTGKLLAASSSGSIKIVSAEEKG
LTLEDTQGMFLLLAAGAIIAAAALISEWMGGCNRKCRPKKKVNSADRKILSVNSRENLIPT
PKSDVSSEIKFITDDDDVDSRVYFNQRPYSAGSRDTLDGHTIHVTDETIIVHQSVDTDRWE
YRRSSSMDLDKEVQEIFERDQRKRRFQSENILATNNITRHHTASKSAFGDPIISSTI-
>PxylIRd2-2
MADAAVDTKEVAPEEVTSTEAAKESPVKKAPVKETKEAESNGKEENGSGDAPADSPAEN
GDADESNDASENGDATEKKEAAVKRKSTADNGTAEKTTPEKKVPTKHRQKYNDMILAK
KSPHVVLDFTMTGIGSETVKSFTAALSLPTISGSFGQIGDLRQWRTLTANQTKFMLQVMPP
ADILPESIRAIVTKQDITNAAIIFDEYFVMDHKYKSLLQNIPTRHVITPVKSFNRDEIKNQLK
SLRQLDIVNFFVIGSLRTIKNVLDAADENQYFGRKTAWFALSLDKGDISCGCKDATIVYLR

PTPDAKSRDRLGKIKTTYSMNGEPEVTSAFYFDLSLRTFLAIKSLLDSGKWPNDMKYITCD
DYDGKNTPNRSLDLKAAFQEVKESPTYAPFFIPEDEPMNGRSYMEFNTEISAVTVKDGASI
GSRSLGSWKAGLANPLSLSDPENMSDYSAQLVYRIVTVEQQPFIIRDDDAPKGFKGYCIDL
IEEIRQIVKFDYEITLAPDNNFGTMDENGNWNGIIKELIEKRADIGLTSLSVMAERENVVDF
TVPYYDLVGITILMKLPRTPTSLFKFLTVLENDVWLSILAAYFFTSFLMWVFDKWSPYSFQ
NNREKYKDDEEKREFTLKECLWFCMTSLTPQGGGEAPKNLSGRLLAATWWLFGFIIIASY
TANLAAFLTVSRLDTPIESLDDLSKQYKIQYAPLNGSAAMTYFERMAHIEVRFYEIWKEMS
LNDSLSDVERAKLAVWDYPVSDKYSKMWQAMKEAGLPNSIEEAVQRVRDSKSSSEGFA
WLGDATDVRYHVLTSCELQMVGDEFSRKPYAIAVQQGSPLRDQFNNAVLLETVIRCILTM-
>PxylIRk1-like
MIFRMGEWAFFLILFFGHLSALPDTIRIGGLFHPEDDKQEVAFRYAVERVNADRAILPRAKL
LAQVETISAQDSFHASKRGGPLLKQIKNSAESHIVLDCATDRIRDVLQQAQQIGMMSDYH
SYLITSLDLHSVDLEEFKYGGTNITALRLLDPERTDVQRVIRDWVYDEARKGRKLQLAHT
SAKENMTFIKTETALMYDAVHLFAKALHDLDTSQQIDVRPLSCEAEDTWPHGYSLINYMK
IVEIKGLTGVIKFDHQGFRSDFSLDIIELTREGLQKAGTWNSSEGVNYTRSYGENQKQIVEI
LQNKTLIVTTILEVSVNDAAVSTRMVAGMWWFFTLIMISSYTANLAAFLTVERMDSPIESA
EDLAKQTKIKYGALKGGSTAAFFRDDTSKSSSTANELGLANVGGVFVVLMGGMGVACVI
AVCEFVWKSRKVAVDERKEEASLCSEMASELRSALKCPSSGGSNGGAGGARDGAGSPYL
HYGFNTKSQLH-
>PxylIRk2-1
TCDSDAPWPHGKELRDNINTVSAHGLTGPIEFKDGVRTSFKLQLMRLAGGEGGGSVVAG
HWSPGEGLTVTDPAAYMRDPPPNVTLTVVTVEEKPYVMVKEGWNLQGNARFEGFCIDLL
ARVAARAGFHYRLRLVPDNMYGVRDPVTGQWNGIVRELVDRKADIAVASMTINFARETVI
DFTKPFMNLGIGILFKVPTSQPTRLFSFLNPLAIEIWLYVLAAYILVSFTLFVMARFSPYEWS
TSTHVCGHETKLLTNQFSVCNSFWFITGTFLRQGSGLNPKATSTRIVGGIWWFFTLIILSSY
TANLAAFLTVERTVLPIQSAADLAAQNQVQYGTLNGGSTMTFFRDSNIDIYQKMWHHMS
TTSPPALVSSYEEGVRRVLGGNYAFLMESTMLDHRVQRDCNLTQIGGLLDSKGYGIATWK
GSPWRDKISLAILELQERGVVQILYDKWWKNTGDVCNRDGKDSKANALGVQNIGGLA-
>PxylIRk2-2
MIFRMGEWAFFLILFFGHLSALPDTIRIGGLFHPEDDKQEVAFRYAVERVNADRAILPRAKL
LAQVETISAQDSFHASKRVCHLLRSGVAAIFGPQSAPAAAHVQSICDTMELPHLETRWDYR
TRRESCLVNLYPHPAALSRAYVDLVRAWGWKSFTIVYENSDGLVRLQELLKAHGPSELPV
SVRQLPDSHDYRPLLKQIKNSAESHIVLDCATDRIRDVLQQAQQIGMMSDYHSYLITSLDL
HSVDLEEFKYGGTNITALRLLDPERTDVQRVIRDWVYDEARKGRKLQLAHTSAKENMTFI
KTETALMYDAVHLFAKALHDLDTSQQIDVRPLSCEAEDTWPHGYSLINYMKIVEIKGLTG
VIKFDHQGFRSDFSLDIIELTREGLQKAGTWNSSEGVNYTRSYGENQKQIVEILQNKTLIVT
TILSSPYCMRKEASEKLTGNAQFEGYAIDLIHEISKILGFNYTFKLAPDGRYGSYNRETREW
DGMIRELLEQRADLAIADLTITYDREQVVDFTMPFMNLGISVLYRKPIKQPPNLFSFLSPLS
LDVWIYMATAYLGVSVLLFILARFTPYEWHQTQSADGEKMVNIFSLANCLWFAIGSLMQQ
SCDFLPKAVSTRMVAGMWWFFTLIMISSYTANLAAFLTVERMDSPIESAEDLAKQTKIKY
GALKGGSTAAFFRDSNFSTYQRMWSFMESARPSVFTSSNKEGEERVMRGKGAYAYLMES
TTIEYVVERNCDLTQVGGMLDSKGYGIAMPPNSPYRTAISGAVLKLQEEGKLHILKTKWW
KEKRGGGSCRDDTSKSSSTANELGLANVGGVFVVLMGGMGVACVIAVCEFVWKSRKVA
VDERVYF-

>PxylIRk2-like1
MEIAFLFLLVFLINLGCIASELSLRFVFITEVQEPELAQQIARALKVSENIRPELRLTDFIVYL
DRENEEESYRKLCSAVSSDVSMIIDLSWSPWESATQIAEQTSIPLVRSLLGNQQLVSALDDY
LEVRNATDAALLLPSEIDVDKTLYHLLGSSNVRVWVHSGLTKDAARALKSMRPEPGFHAII
GDNTFVTDTYRRAVKEKLVRRDYRWNLVLTDYSAANLDLSMTVPTMILSADPTECCKLL
GQAEDCSCPADIQRKQHILNALIAYISEVYFKLDTSIPTVSVSADCENLRMSADMNVTRDR
LYRQFAEDAALSNDTIFYWDEDQMGLFLRTRFTLSSYAPSEGLNHVASWSADEEFKLLPG
VTLQPLRMFFRIGTTAAVPWTLPKLDPDTRQPMFTEEGQPMYEGYCIDLIQKISEVMEFDY
EIVTPQTGTFGRKLPNGSWDGVVGDLMRGETEFAVSALTMTAEREEVIDFVAPYFEQTGIL
IVIRKPTRKTSLFKFMTVLRTEVWLSIVAALILTGFMIWLLDKYSPYSARNNPEAYPYPCRE
FTLKESFWFALTSFTPQGGGEAPKALSGRTLVAAYWLFVVLMLATFTANLAAFLTVERMQ
TPVSSLEQLARQSRINYTVVEGSTIHEYFINMKFAEDTLYRVWKEITLNATSDQAQYRVWD
YPIREQYGHILLAINASGPVPDAKTGFEQVNEHTDADFAFIHDSAEIKYEVTRNCNLTEVG
EVFAEQPYAIAVQQGSRLQEDLSRALLDLQKERFLEQLASKYWNETAKQACPDADESEGI
TLESLGGVFIATLFGLGLAMLTLAWEVFYYKRKEKNKIKSIDATVEKPKEAFAPSKKSLLE
TKMAEGVARLRKRDKKGKGNLPKNVTIGDTFRPAADNANVSYIKVYPKDGFKL-
>PxylIRk2-like2
CTSKGLNPIAIFGPQSPISDGAVRDQCARAHIQATWQPNDPDMYTQPEPQPEEKGEEEEDE
DEDSPPGEEDETEEEQQEIEEVPTFKKISINFYPDDDELAMAFADVIRYYQWNSYAVLHED
DYGLLRVQKILARHSDKYSITVRRLDPNGDNQKVFKELKTSRSSRVLLDCHIDHVDKYMK
EAEILGMLDHYRHYILTSLDGSILSDTMTHYQSNITWLSITNFESLKNTQHTLSTRVGKWK
NDIPNYASTAVTTLQTEALLMDDVANFVLTALKDLPEEVRDNAGHPCEREGPWELGAPLQ
NQLLKTEMLGVTGLIKFNEYGKRVNYTLYVNEIHVNKRNTIAKWESATGELAENRSSSDD
EISQTSKHFVIISKIGKPYFYKKENQSLEDSDEEEYEGFSVDLVEAIFDVLKNKMNLNYSYS
FSKASYKDYGKINPETKKWEGLIGDLLDKKADLAVCDLTITEERKSVVDFSVPFMTLGISI
LYTKDHKVDPGTFSFLYPYSFDVWMLTATAYCAVSICLFVCSRISPADWENPQPCDKDPEE
LENIWNFKNCAWLTMGSIMTQGCDILPKAIGSRWVCGMWWFFAMIVCQTYIAQLAASMT
AASEQEPINSVEDLAQNNKILYGALNGGSTLSFFKNSKDKMYQRMYENMMSNPAVLVRD
NEEGERRVLSGKNKYAFFMESCTIEYKLKRNCELKKVGGELDSKDYGIAMPANSPFRSQI
NGAILRLKELTRLDEIKNHWWNDVNGAKNCSVDKDTEDVEGDLELKNLSGAFIVLVAGL
FCSLVITAAEFMNEVRNIVVREQVTHKEAFIQELKTSLNFFQLQKPVLRNPSRAPSVAASVA
SDRDARVNARLTSAANFLDLEKVTQ-
>PxylIRk2-like3
MNLGISILFRQPTAPEPKFFSFLLPFSTGVWVCLGLAYVGSSLVLYVVGRLSPAEWQNPFPC
VEEPEALHNQFTLANAFWFNLGAVLLQGSEIAPVAYSTRAAASAWWVFALVVTSSYTANL
ATLLARKSSDELISGVTDLAHNPYGIEYGAKAGGATYTFFEHSQNELYNEMFEHMKTKEM
PTTNEEGIKKVMTENFAFLMESTTIDYETQRNCLVIQVVETLKS-
>PxylIRk2-like4
MSQRSGAALLLLILVITGRVYETAAAFRNFETLKTTVTIGAIFPPNTVTEVAFASALARASME
SEHHHYVMKVVYAQYGDSFSASKAACQLTALGVIAIFGPTDPESATAVAAICRAAKIPHVQ
AVWQPPPIRGPERLSPPSINLYPEAVALSRAVALFIKDSDWNSYTLLYDDENGLIRLQEILKH
ADQEHRWYVRKLTPGEDNRPLLKSLKAMGETRVILDCPADRVKEYLRQANEVKFFEDYM
SYVLMSLDAHSIDLGDLRYGLSNVTCLRIFDHDDARTRSFLADWKARGSDDVKIPKESHE
ITVEIALASDAARLITDAVESAPEEFKLETQEIECGSDGQWEIGEMFTNHILTNPIVGITDQIA

LDNTTGERINFNVEIMELSNSGFNRIAKWNADTGFDYVRSASDVSGLLAEKWQNKTFKV
VSRLGAPYLVEKVPAEGEVLTGNDRYEGYSKDLIHEILKNTLNLNYELEIVPGNSYGTYNK
DTKKWNGLVGYLLDRKADLAICDLTITYERRSVVDFTTPFMTLGISILYSKATPPEPELFSF
LKPFSVDVWIYMAAAYLAVSLTLHVLARLAPNDWENPHPCDKSPEELENIWHIKNSCWLT
VGSIMTQGSDILPKGYSTRWVAGMWWFFALIMCSSYTANLAAFLTNAAMDDSIKSAEDL
MSQTKIKYGTLYGGSTYSFFKRSNVSTYQRMWTAMEASRPSVFVTSNDEGLERVLKSKR
KYAYFMESTAIEYQLERHCDLMQVGGLLDSKGYGIALPFFASYRTAVDNAVLKLAESGML
VELKNRWWKVPEGEGTCASSEDAAEESSGELGVDNVGGVFVVLGVGCGMAAGMGALE
FLWHVRDVAIEQKMSQMDAFWAELTFAISFWETEKPVAHSRPSSSASGSNNVSRASSVLRS
AADLFNVFNK-
>PxylIRk2-like5
MWWSISTFVGLLFLQQCTSDVKITTIEEKFYEIVGIFEPSAHVQRRAFNESMKFTSVGEMR
LGPRILEPPKTDSYAVWRKLCTSKGLNPIAIFGPQSPISDGAVRDQCARAQIPHIQATWQPN
DPDMYTQPEPQPEEKNEEDETEEEQQEIEEVPTFKKISINFYPDDDELAMAFADVIRYYQW
NSYAVLHEDDYGLLRVQKILARHSDKYSITVRRLDPNGDNQKVFKELKTSRSSRVLLDCHI
DHVDKYMKEAEILGMLDHYRHYILTSLDGSILSDTMTHYQSNITWLSITNFESLKNTQHTL
STRVGKWKNDIPNYASTAVTTLQTEALLMDDVANFVLTALKDLPEEVRDNAGHPCEREGP
WELGAPLQNQLLKTEMLGVTGLIKFNEYGKRVNYTLYVNEIHVNKRNTIAKWESATGEL
AENRSSSDDEISQTSKHFVIISKIGKPYFYKKENQSLEDSDEEEYEGFSVDLVEAIFDVLKN
KMNLNYSYSFSKASYKDYGKINPETKKWEGLIGDLLDKKADLAVCDLTITEERKSVVDFS
VPFMTLGISILYTKDHKVDPGTFSFLYPYSFDVWMLTATAYCAVSICLFVCSRISPADWENP
QPCDKDPEELENIWNFKNCAWLTMGSIMTQGCDILPKAIGSRWVCGMWWFFAMIVCQTY
IAQLAASMTAASEQEPINSVEDLAQNNKILYGALNGGSTLSFFKNSKDKMYQRMYENMM
SNPAVLVRDNEEGERRVLSGKNKYAFFMESCTIEYKLKRNCELKKVGGELDSKDYGIAMP
A-
>PxylIRk2-like7
MIQIIPIFILACANMGAVDAVSTTSFPIGGLFNAETKKDSMMAFQNIVTKCNSYHGEPLFSK
VMDAYSTALDVCRYASGRAAVTALLDARPTYGVCDATCLLANKLNITHLSVGWEPPSSLA
ENPFTFQYHPSPELISKAFAALIRDVLEWDKFTILYEDDDSFIRLQEVINTWPPEHEPILYRR
LDPKGDNRETFKYIFKVEHMSNHILDCHVDNVKKYFQEIIQVDNTTQYQSFILTALDAYM
VDLAEIPELVANVSTLHLTIDDPDRWNDIKMDGHYMSLETALTADALNHLEASIRKMSDT
DRDEYQGGPMKIEDPPSLCFMSSSEEYEREAWPPGERLRRAMIATQAYGFTGEIEFDEEGK
RRNFKIYYSKLDHDSKFQNAGFWNSTTDKITELNVVADRSTAQLPDSVIRVVSRVGSPYF
MINETNSTHVYGYAVDLIDAIFAHINSNDKFNTYKYEFYRVEGDSYGNPVEGTKNKKWTG
MIGDLIEHKAELGACDLTITAERARAVDFSVPFMSLGISMLFKEPDPEPANIFSFIQPLTLDV
WLYLATTYIIVSFILLICARMSQDDWVNPHPCNQNPDSYENIWTLYNCMWLTMGSIMTQG
CDILPRAASSRWITGTWWFFVLIFTASYTANMSMFLSNNRRSNDITNVKDLSEQNKISYGA
GDNTSTYKFFQYSNDSVYAKIWSVMSTAKPTVFTKNNDEGKDRVLRSKGKYVFFMESTAI
EYYTKRNCELKMVGSKLDSKDYGIAMPKRSYLKNKVDAAILHLQELGEMDNLKKKWW
EDENIKCDDPSKTEDDSGSLQMKNTSGIFLVLGFGGFIGLIVAIIDFMLHANKIAVKEKITFV
EAMSHEWKASLDPRVLHKAAGARSAAPSTRSSSRSRSRPARAVSVLSNIINFDDIY-
>PxylIRk2-like8
MQWTLKLNRLCVLIVAFCYCFVVGDKNIGAIFDDGTFLVQAAFKVALTDASKDQENPFVE
NVAQTPPGDVYEAEKGVCQLLENGLLGIFGPSNRASLTHVQSVADYLEVPHVIVEPMILQT

RNWSVINLYPHHDAYSQVFADIIVMKGWSEFTIIYEGAEILPFLTNVLTIQDLDSDDDILMN
VVQLPDNDDFRSFLQTIKKSGSVNYLVACTIAKLPTFLLQAQQVGIMSDDHSYLIMNPDFQ
TIDIEPFKHGGSNITGVRLFDPSLEEIQTFVKAINEYVTELSEGQIENAVTEATLNLNLALTY
DAVILYTAALTALGLEEGAAVSCTKDDSWSYGSSVINYIRTMELDSLTGLIKFDEDGFRSDF
EMEIVELMPHGLEKVGQWSEEDGYSQTRNIIPPADLEGSDAMKGKHFIVLISLTAPYGMLK
ESADKLEGNARYEGFGVELIDELSKMNEFNYTFEIQTDGVYGSLDKTTGKWNGMMEKV
MDGRVDFAITDLTITAARQKAVDFTSPFMNLGITILYKKPTKQPPDLFSFISPFSLEVWGWV
AGAYFGVSVLLFILGRFAADEWQNPYPCIEEPETLDNQFTMANSFWFTLGSLLTQGSEIAPI
AVSTRMAGSMWWFFTLIMVSSYTANLAAFLTVESKFYAIKSVTDLAANPYDITYGAKKG
GATFSFFKESDNLLYQKMYQYMEDHPEYQTASNEEGLERVKSDDENYAFLMESTSIEYMV
ERNCDVAQVGGLLDSKGYGIAMKKNSPYRQPMSESILQLQEQGTITRMKDKWWKEKRG
GGACADDDTGSGEAQPLVLANVGGVFIVLAGGSGMAAICAVFEMLFDVWATSRREKVPF
KDELIAELKFILSFSGDIKPVRHPRESTGSGSSKKEKEEEDAEEPDETEDHRDRMPTPARSE
KSTHSHHTLHSRRQSNSLHMARSRKFSRP-
>PxylIRk2-like9
MQWTLKLNRLCVLIVAFCYCFVVGDKNIGAIFDDGTFLVQAAFKVALTDASKDQENPFVE
NVAQTPPGDVYEAEKGVCQLLENGLLGIFGPSNRASLTHVQSVADYLEVPHVIVEPMILQT
RNWSVINLYPHHDAYSQVFADIIVMKGWSEFTIIYEGAEILPFLTNVLTIQDLDSDDDILMN
VVQLPDNDDFRSFLQTIKKSGSVNYLVACTIAKLPTFLLQAQQVGIMSDDHSYLIMNPDFQ
TIDIEPFKHGGSNITGVRLFDPSLEEIQTFVKAINEYVTELSEGQIENAVTEATLNLNLALTY
DAVILYTAALTALGLEEGAAVSCTKDDSWSYGSSVINYIRTMELDSLTGLIKFDEDGFRSDF
EMEIVELMPHGLEKVGQWSEEDGYSQTRNIIPPADLEGSDAMKGKHFIVLISLTAPYGMLK
ESADKLEGNARYEGFGVELIDELSKMNEFNYTFEIQTDGVYGSLDKTTGKWNGMMEKV
MDGRVDFAITDLTITAARQKAVDFTSPFMNLGITILYKKPTKQPPDLFSFISPFSLEVWGWV
AGAYFGVSVLLFILGRFAADEWQNPYPCIEEPETLDNQFTMANSFWFTLGSLLTQGSEIAPI
AVSTRMAGSMWWFFTLIMVSSYTANLAAFLTVESKFYAIKSVTDLAANPYDITYGAKKG
GATFSFFKESDNLLYQKMYQYMEDHPEYQTASNEEGLERVKSDDENYAFLMESTSIEYMV
ERNCDVAQVGGLLDSKGYGIAMKKNSPYRQPMSESILQLQEQGTITRMKDKWWKEKRG
GGACADDDTGSGEAQPLVLANVGGVFIVLAGGSGMAAICAVFEMLFDVWATSRREKVPF
KDELIAELKFILSFSGDIKPVRHPRESTGSGSSKKEKEEEDAEEPDETEDHRDRMPTPARSE
KSTHSHHTLHSRRQSNSLHMARSRKFSRP-
>PxylIRk3-like1
MVCLTGAMGTLLWIGLLMVSAGAGADMTLGGIFYEDDVDMQSAFNLSTRMYNFTSSMR
TVQRGQILEVSEHVCDLVKEGLIGIIDGTGGRSSEHIQAVCDMLEVPHVLVQHNDLFTANW
SILNLYPSGEAYNTALEKLIIHKGWQSFTVLYVKGHILGRVDNLFRLGNETSDMIVSVRELS
GEDYRDVLIEAKESEFTNFVVDCPSQNIELVLNHSQQVGLLADEHSYIFLSPDLFTLDLTQY
RYGGVNVTGFRLMDLKQNKKMWEYTADLNEETGQTREPEEIKTEVVLIHDAVKVFYEAL
KKVAVEPQELSCDNYDSWAYGSSLLNFMRTNKINGITRQLLFDGFGQRTNINLEIMELTPA
GNQSIGEFNNGLLDIQRPVLPVAQLTSESIMKNKTFIVLISTTAPYGYIKESLQELEGNDRYE
GFTVDLIEELSKLLEFSYEFREQNKYGTLYANGKWDGMVAEIMDEKADFGICDFTITSDRQ
RAIDFSVPFMTLGIGILYKEPSKQPPEMFSFMAVFSTEVWIWMLFAQIGLGVVMIFVGRISH
KEWQNPVPCIEDPDEFSNQFSFANSVWLIIGSVMQQGSEIAPIALAPRMITSVWWFFTMVM
VASYTGTLVAFLTVEKNVLPFTNVEELYRHKSISYGAKKDGSTINFFRDSKNEIYQKMYSK
MKSNPGWLVTHNDIAVSIAEEKNYAAFMESTSIEYYKERHCDLLQIGELLDTKSYGIGMK

KGSEYKKHIDDALLTLKERGDIQKLKDIWWKEKRGGGKCGVKRDEEQKQLTLRNMVGA
FVVLGVGCLLGLLISTLDMLWGVFKRSLKYQTTFQYELVEELKFALTFSGDVKPVRRRPT
EGSSEALADDPEAKDELKSLRSVRSSDTHRTHHSHSHSSRHSSRSQSVAFARMRSYD-
>PxylIRk3-like2
MGAALGRLTGELMHYCWPLGVAYGGHVQKILPGGYATVGAAAFTGAVTQTVSTMVIVL
EMTGQSSISLNPLLNQTLSGIFYEDDVDMQSAFNLSTRMYNFTSSMRTVQRGQILEVSEHV
CDLVKEGLIGIIDGTGGRSSEHIQAVCDMLEVPHVLVQHNDLFTANWSILNLYPSGEAYNT
ALEKLIIHKGWQSFTVLYVKGHILGRVDNLFRLGNETSDMIVSVRELSGEDYRDVLIEAKE
SEFTNFVVDCPSQNIELVLNHSQQVGLLADEHSYIFLSPDLFTLDLTQYRYGGVNVTGFRL
MDLKQNKKMWEYTADLNEETGQTREPEEIKTEVVLIHDAVKVFYEALKKVAVEPQELSC
DNYDSWAYGSSLLNFMRTNKINGITRQLLFDGFGQRTNINLEIMELTPAGNQSIGEFNNGLL
DIQRPVLPVAQLTSESIMKNKTFIVLISTTAPYGYIKESLQELEGNDRYEGFTVDLIEELSKL
LEFSYEFREQNKYGTLYANGKWDGMVAEIMDEKADFGICDFTITSDRQRAIDFSVPFMTL
GIGILYKEPSKQPPEMFSFMAVFSTEVWIWMLFAQIGLGVVMIFVGRISHKEWQNPVPCIE
DPDEFSNQFSFANSVWLIIGSVMQQGSEIAPIALAPRMITSVWWFFTMVMVASYTGTLVAF
LTVEKNVLPFTNVEELYRHKSISYGAKKDGSTINFFRDSKNEIYQKMYSKMKSNPGWLVT
HNDIAVSIAEEKNYAAFMESTSIEYYKRHCDLLQIGELLDTKSYGIGMKKGSEYKKHIDDA
LLTLKERGDIQKLKDIWWKEKRGGGKCGVKRDEEQKQLTLRNMVGAFVVLGVGCLLGL
LISTLDMLWGVFKRSLKYQTTFQYELVEELKFALTFSGDVKPVRRRPTEGHSSRSQSVAFA
RMRSYD-
>TcasIR40a
MRRDHGGDLVSASFDIVAGFLFEEICICFDKNTNINFLQHLLVRFVSNNIAIKLFNITTVEVQ
DKYFAFLNYQVTNHLGANTIFFSSHKFYEHVLLEINERDFIRRNLIYIFNWGRRPFSRYFVR
NIINVMKVFVITNPRNDTFRIFYNQAVPYKKHHLEMVNWWQHGVGLFNHPTLPAKYNNV
FKDFKENVFKIPVIHKPPWHFVQYGNDSIKVTGGRDDRILSLLSKKLNFRYDYFDPPERIQ
GSSASENGTFKGVLGLIWKRQAEFFIGDVALSHERANYVEFSFITLADSGAFITHAPSKLNE
ALALLRPFQWQVWPAIGVTFVVVGPVLYAIIALPNAWRPRFRVRSHARLFFDCTWFTTTV
LLKQTGKEPSSSHKARFFIIILSISSTYVINDMYSANLTSLLAKPGREKAINNLNQLEKAMAT
RGYDLYVERHSSSYSLFENGTGIYSRLWQMMNRRQTHFLLESVEEGVQLVRDSTNKAVIA
GRETLFFDIQRFGASNFHLSEKLNTAYSAIALQLGCPYIEEINKILMAIFEAGIITKMTENEY
EQLGKKKQTTSETEKELIPGVKKENRRVAKVSEDNEKLQPISIKMLQGTFYLLCIGNIFSGF
ILLAEILVYKHRKTYKHKKRRHRFVYLRKIRHSVASKFGAVVDAVRRVYRRAMHDAFVAT
LEYLE-
>TcasIR21a
MQRGLIVLKLCLTALALKSLDKRALQKSHEKSQLEKWEDKFLNRDPSFDQTASLVNLISK
VALDELSGCSATILYDKFTETSSDLLLEKLFRTFPIPYLHGQITDKYHMKVPKLQTSQDTCT
GYILFLKDVMRSKDVVGPQTNNKVVLVSRSSQWRVYEFLASEQSQSFMNLLVIAKSEKIV
SSSIARLICLALHLKFGTALAIYAPNGGKSAVYPSVIANVPKLGFRSAESVTSVITQNGANL
GIGGLYITDTRLKATDMSHIHSQDCAAFISLASTALPRYRAIMGPFHWTVWLSLTLVYLFAI
FPLAFSDKHTLRHLLDKPEEVENMFWYVFGTFTNAFSFFGKDSWSKTDKFATRLLIGFYW
IFTIIVTACYTGSIIAFVTLPVFPATVDTPEQLVRGKYTVGTLDKGGWQYWFENSTDPITQK
LLTRIDFVPDIESGLKNTTKAFFWPYAFLGSRAQLDYIVRTNFTTINKRSLLHISSECFVPFG
VSIIYNKNALYSKIIDQGVLQAVQSGIVDKIKNDVEWETMRSASGKLLAANSYGKSLKALT
VDDRALTLDDTQGMFLLLGIGFLLGGASLLSEWMGGCLHLCKGNRNQSATSIQSNYRSHE

VPTPREKLDSMQFNSFENHKIEEEIVEERNCIIHRQDDDDIEEHINRLFDFEGVFGEANPDS
RTGPEEELSFKNTTKAFFSLYAFLDSRAQLDYIVRTYFTSMNKRSLLHISSECFVPFGVSIIY
NKNALYSKIIDQGVLQAVQSGIVDKIKNDVEWETMRSASGKLLAANSYGKSLKALTVDD
RALTLDDTQGMFLLLGIGFLLGGASLLSEWMGGCLHLCKGKRNQSATSIQSNYRSHEVPT
PREKLDSMQFNSFENHKIEEEIVEERNCIIHRQDDDDIEEHINRLFDFEGVFGEANPDSRTG
PEEELSEENGKK-
>TcasIR76b
MGLFEIALAALCLNATCPGEEEPPEFPEVQYLAPDSNDRKTLFAQLTEQLKNENLIITTLKN
DRLSGTEKRNNTILGKGIAFDLLNILQDKFQFNYTLIEPKANVWGAEKFGVLDLLKDKKA
NLSAAFLPVLTQYSNHISYSPSLDTGEWVVLMKRPKESATGSGLLAPFNLPVWLLILLSLV
VVGPVIYFIIYLQAKLCKDDNNKVFPLPACIWFVYGALLKQGTTLNPMTDSSRLLFATWWI
FITILTAFYTANLTAFLTLSKFTLPITEPKDIGEKRYKWVTTKGNALEDTVTVNESLTELGKI
LGQPQRYLYVSDSDILRNYVHKRNWMFIREKPIVEYVMYDDYKEKTRNQIEEAKRCTYVI
TKFSVVSFSRAFAYSKDFKYKPLFDSTLVQIVKCHKCFSLLSRIQYLVESGIIKFKLREELPD
TEICPHNLGNKERQLRNSDLLMTYEIVGGGFIISAIVFIIEVIIRRQKKPKTKSLPLQNPNKH
TFEINLNNNYEKFGHFPYSSKFVTPPPPYHTLFNPPHKSDNMKKRNFNGREYWVYDSISG
ETKMIPMRTPSALLFQYTN-
>TcasIR93a
MLLELVLSSAFVCVIRGDSFPSLLTTNATLAVIIDREFLSNEYEVIKHAIESYLVFAKREILKH
GGVNVQYYSWTTINIKKDVTAIFSIASCPDTWRLFRQARDANLLHMAISESDCPRLPPDEA
ITVPLITRGEELPQLLLDLRTRQTYNWNSAFILYDDTLSRDQVTRVVKSITAQYSNLRVNAA
AISFVKLETRLPMDEIRRQVKEILSSVSIKTVGGNFLAIIGYELVELLMEYAKMFGLVNTRT
QWLYIISNTHFRHKDINRFRQLLSEGDNIAFLYNNTVNNDTCTGGIQCHCEEILSGFTRALD
EAILFEWETSSQVSDEEWEAIRPSKLDRRNSLLQGIKTFLLQRGQCDNCTSWLMKTGDTW
GREYQQNGTDSGGLISVGNWRPSDGPSMSDELFPHIVHGFRKRNLPIVTFHNPPWQIIRSN
ESGAVSEYAGVIFELIKELSKNLNFTYTVELAKIGQEFSANLTKNEAQVVTNFIPDSILDMIR
NKSVAFGACAFTVTEESKRLINFTSPISTQTYTFLVSRPRELSRALLFMSPFTGDTWLCLSA
SIVSMGPILYYIHKYSPVYEYKGLSKRGLSSVQNCIWYMYGALLQQGGMHLPQADSARII
VGAWWLVVLVLATTYCGNLVAFLTFPKIDIPITTIDELLAHSGTVTWSMPKGSYLERTLKY
TTEPRFRYLFDKKVEVGNFKNMIEDIENGKHVHIDWKIKLQYIMKQQYLDSDRCDLALGL
DEFLNEQLAMVVSQDTPYLEIINDEIKKLHQVGLIQKWLTDYLPKKDRCWKNNRHIVEVN
NHTVNMDDMQGSFFVLFLGFLLSFFITIGEKLWHKYVTKKKMKIIQPFTT-
>TcasIR64a.1
NKISLILVILSKTETYIIKSCLSNAIVDFAILANVAFSLRISCYKLFMHIKLIANVFYNQLDQV
LNRNHYHLAVIIDSGCIDYADFAIQDKKYFYETYHWLVPTTPQNLNNSLNFLQKSPLNINS
DVNVAILNGEGTKWSILDVYNPASSHHGQFTVTKLGLCDETNGYQAKIAGNKYWSRKN
MTGVQFKSAVVVPDPSIKLNDYLTSDKNRQLHSMHRFQSVTVNYCREMYNFSLEIQRTNS
WGYLTPNGHFDGLVGLLERRLVDFGSSPLIYKLDRMPVIDYSYGNWVLRSTFIYRRPKIIE
ASYKIFLRPLSRTVWICIVLMMVLLMLFLKVVFSREKRLLQKRNLVDSSWSFLFLFTLGAF
CQQGATCHPQLLSSRTLSIFVFLFCILTYQFYSASIVSYLLIDPPRKINNLKDLSDSNLRAGIE
DILIDRNYFVQTTDPVAIELFNKKIKFSNNNSGFYEPWDGLDLVKQGGFAFHVETSTAYPIIE
ETFTNEEICELEEVQMYRTQPMHTNLQKNSPFREMMNYCMLHLVENGLMYRLRKYWDA
RKPMCIESAKKFTFNVGLKEFSSGLIVLSYGILISLGLLLREVIVHKK-
>TcasIR64a.2

MSPPLPFMILLSVLTQTHALLDINLIENYFTEKSIKSATVFGCFRKTEQLNLVKIFSRGSSPIS
VLNLNQAGVYQSIKSNHQQIGVVLDGDCPESESFLITVSPGFTHIAPNVVFISVRSTETXFD
VKHHWLILSKSIQFLEKIKNAVVNINADIHVAVQSGTNWTIFDVYNPASEHGGSLKYTRVG
FYSRGRGYNAQTNEAKYWRRKDMTGVTFKTMVVLLVPFEGPLEDYLHNDDNRNINTFN
RFQNKLLRFCRDYYNYSMIVELGSSWGYPFPNGSFDGMVGAMEKKLIDFGSSPIFVREDR
ARVIDYGRNTWSWKAGFLFRSPKSRTSIEIFLKPLSTSIWLITGVLATASIVILKMVTTFERN
RYHSTSETSWSLSFLFTLGALCQQGSPWVPKMACGRITAISIFLLSLIIYQFYSASIVSHLLM
KPTNKIRNLKDLTDSSLKVGCEDIIYNKDLFAHTTDKVLKDLYAKKIYGKGNTSHFFPPEK
GLDLVRQGGYAFHIEVARAYPIIETTFPDNAICELREVKLFKNTDLYNTMQKGTPFRDMLE
SCFQRLAEQGILDREKKHWHPRKPECIQSSQAFVTFHVGLDEFYPALLVLLIGIVISLTVLV
VEKQIHIAREKMEREKGVVF-
>TcasIR64a.3
FQLRVLMERLFFLSVLAVIIYTTNCTDNHDIITSYIKEKSVKYATVFGCFTKKEKINLVKIISH
ICPISVFDINRLNIENRMESRHFHTGIILDGDCPSAEKFLINCGRSYLFDVKHHWLIVASSEKI
REKFNNVILNINADINVIIPEKPSNWSIIDVYNPASQHGGVLNFTRVGFYNKHDGYKIKYTG
VKYWNRKNLTGVTFKSMVVVTYSKTXKNSAYTIFQLPVPFEGTLQHYLDSDDNRDVNTF
NRFHSRLISFCRDYYNFSLDIEVSKSWGYTNEDGTFDGMVGALERKIIDFGSSPLFLREDR
ARVIDYGRNTWILRSLIKQQFRIISNWGFSAAFIFRNPKVRTSLEIFLRPLPSSVWLITGLLAI
VSIIILKLATSFERRRYVYDVETSWSISVIFTLGAFCQQGSPSTPKMACGRIATFFIFLLSVLIY
QFYSASLVSHLLNKPLTKIKNVRDLLLSPLKAGCEDILYDRDYFLHTTDKVAKELYAKKIL
GKSNSSNFHTPEAGLKLVAEGGYAFHVETATAYPIIESTFQDQAVCELREVPLFRTQPMHAN
FQKKSPFRDMFDTCFQRLAEHGLLVRERKHWHPRKPECIQSSKSIRFNVGLDDFYPALVIL
LVGIVASLLILVIEKEFRILTENPA-
>TcasIR75q.1
SFLGTILTVYKQLAEKKIVLNVLTNHWKINQTKLSQHTFLVGDTLCPQFNSLLSHVSKFFC
YQNSQQTLGQIITSSXKWLVFDQNSTVNTNDLLLDSNFAVASQISNGRFHLKLCYKRAPNE
TIKFNEIGVFSNGFEYYNHFIPTRNRSDLSGVNITVSYVVTKPDYPFDVEDYRFRHLEAFSK
LSYAMVYPMLEMLNCTKKFIQRSSWGYKGANETQFVGGMFGDIQNGTAEIGGTVSFYTV
DRMSVVDYLSVTTPSDLKFILRAPPLSYVNNLFTLPFDTKVWYCLYFIVGVTVLILYVIVR
CESTYENALERRNNIDNIKPKFFDVVMLQIEAITQQGSENEPKTMSGRIAVFIVFLVLMFLY
TSYSANIVVLLQSTSANINTLQDLLNSKITLGVEDVVYSHHYFETQTEFTRKSIYEKKVAPK
NQKSNFMTTEMGIEKMKDEFFAFHVETTAGYKQIMDTFQEHEKCGLIEIDYLNVLYPSITI
RKNSPYKEIVKVNFRKIYESGIRHRQLNRIYYKKPHCVGKGGSFKSVGIVDIYFSVEIFAIG
CFMALWLLLLEVLFKKKIKFLVQ-
>TcasIR75q.2
MKILIVFICLLINETTQNNFTDNLIVNTFNFIKILNVPVKISAHICWTRGKFDSLLMKLYXTV
LANTIHFIKSISDKYNTNLIKNVSPKYANPEHQLFIIDLKCNDSLSVLQQAEKFKLFKSPFK
WLLLGNSESLPNLYFGTDSQIFVTEPRSQLDDIKTIYKYSPMVPRFVQHSFDRFYTNTKRT
NLMGTTIKISYVITNLDSLNHLWDYRLQELKKKLYHFLICRNSHIDAINKLNYILVHNLMD
FLNASRQFTMQPTWGYKNSTTGLYSGMAGDLQKGLADLGGTPLFFTPDRIDIIDYIAATTP
TYMKFIFRAPPLSYVTNVFTLPFDSAVWHYCFVMVAVVVVCIYVIVVWEWKETKFEEKD
THSHIDTLRPNIFDVVMFEIGAITQQGTNAEPKSNSGRIITIFSFLTLMFLYTSYSANIVALLQ
STSDSIKNLEDLLNSRIKLGVEDIVYAHYYFENAQEPVRKAIYQQKVAPKGQKPNFMTAEE
GIRKVQQGFFAFHVELSTGYKIIGEVFQEGEKCGLKEIEYVNLIEPWLATQKKSPYKEVMK

IGMRKMHETGVQNREIRKIYTRKPQCHSGGSNFGSVGLIDCYSAFLTFGVGIAFAFLLFVM
ELIVRRYFIRREKERLK-

>TcasIR75s

IVLPMINDLIEHFNKTQIILAYLCDKNGTNLLLIRNNNNTNFRRLSGSEPLFXKKLYQVNVL
SPNSRDMPYPTPPAFLTYVLDAGCSNTKQLLLLVPVITHXLIFGNNILKASEQKQFATPFKW
IVYYNNPVELSFFIDEYFTKTNILVDSDVTLATINPTSGTFDLNKIYKRKINGSIIIENIGIWGR
GLGVTDTGYEKITYKRRRNLTKTVLKSCIVITNNDSLNHLTDKRDIHIDSIAKVNYVLVQH
LSDTINASLEYSVRGTWGYKDNKSQWSGMIGELTRNEADIGGTALFLTSDRIRVIDYIAMT
TPTRSKFIFRQPKLSYVANVFTLPFDASVWASVCGLLVIIAGLLYVVVVRWEWKKKDYVQV
VVFFAFWVDFPSSVFCRTNRTSRKFTILGSXVFITFGALCQQGSSSVPFSIPGRITLIFLLVSL
MFLYTSYSANIVALLQSSSSSIQTLQDILNSRLDVGVDNTVFNHFYFPNATEPIRRAIYQQK
VAPPGQKPKFYPIEEGIRKMRQGLFAFHVETGPGYKFVSEIFREDEKCGLQEIQYLQVPDP
WLAIQKNSSYKKMLKVGLRLLQENGIQEREVGLIYTKKPQCLARGSSFISVGLVDCYPAA
VVLAGGIGAALAVLILEIYVHQRFVGFLL-

>TcasIR8a

MVISENLDKTTANRLKAIRPIPNNFAIVATSSNMEELLQTALDENLVTLPERWNLVFLDFQY
QQFDKKRLKNMPINLLHMDEEICCRFLQSEKCECPHDFNLQENFLSLATNTLAKILKTLTM
ENLLRADLNCDDSRYSEATRTRFYELLQQEVDSNDLVFKENFGLHVNINGVIETGDEKVAE
YNYKTGVTVLDGKKVEPITPFFRIGITHALPWSYKETDSSGNTYWTGYCVDFTEELSKLM
GFGYEFVEPKSGTFGKKRDGVWDGVVGDLATGETDLAITALIMTADREEVIDYVAPYFEQ
TGITIVMRKPVRKTSLFKFMTVLKLEVWLSIVGALIVTGFMVWFLDKYSPYSARNNKKAY
PYPTREFTLKESFWFALTSFTPQGGGEAPKALSGRTLVAAYWLFVVLMLATFTANLAAFLT
VERMQTPVQSLEQLAKQSRINYTVVKDSDTHKYFINMKHAEDTLYRMWKELTLNASTDD
TQYRVWDYPIREQYGHILLAINDSNPVANASEGFRIVNEHTDADFAFIHDSSEIKYEISKNC
NLTEVGEVFAERPYAVAVQQGSHLQDEISKTILNLQKDRFFEQLQAKYWNHSGKGSCPTT
DDNEGITLESLGGVFIATLFGLALAMITLVGEVLYYRRKSKIQNSETKKPKTVQTSENWKT
DTLMPVSLINKDKQSVTIGTEFKPVNRNRDLSEFGHITLYPRARNRITQTSNE-

>TcasIR25a

MASSSAIIYRIAIYSRIATAHLNYSDFLNNVLTETHKMLKLVAFILYCTNLANGQTTQNINVL
FVNEEGNLVAEKAVDVATNYIKKNNKLGVNADPVKVVGNRTDASGLLDSLCSSYNEMIA
NSMNPHLVLDTTMTGLASETVKSFTAALGLPTISASFGQEGDLRQWRNIDENEKEYLVQIS
PPADVIPEIIRSLVLSKNVTNAAILFDDSFVMDHKYKSLLQNVATRHVIAPIKEADKIGDQL
RQLRKLDIVNFFILGSFENIKRVLDAADSVGFFNRKFSWHAITQDKGELKCNCRNATITLA
KPLIDAQYQDRLGLIKTSYQLNAEPEIAAAFYFDLALYSFLAVKEMIADGVWKRNNATNYI
TCDDFDGKNTPRRAGLNLKKYFSKEVSETPTYGPISIVSNGYSFMEFTMQISAVGVRESSS
DKSVPLGSWKAGYDNNLTLVDPQIMKNYTADVVYRVVTVEQKPFIIKDETAPKGYKGYCI
DLIQRISEILNFDYEITPVGDQKFGNMDENGKWNGVVRELMEKRADIGLGSMSVMAERE
NVIDFTVPYYDLVGITILMKLPKTPTSLFKFLTVLENEVWLCILAAYFFTSFLMWVFDRWS
PYSYQNNREKYKDDEEKREFNLKECLWFCMTSLTPQGGGEAPKNLSGRLVAATWWLFGF
IIIASYTANLAAFLTVSRLDTPIESLDDLSKQYKIQYAPLNGSSTMTYFERMANIEAKFYEIW
KDMSLNDSLSEVERAKLAVWDYPVSDKYTKMWQAMKEAGLPNTLDEAVKRVKDSRSSS
EGFAYLGDATDIRYLEITSCDLQMVGEEFSRKPYAIAVQQGSPLKDQFNTAILQLLNRRELE
RLKEKWWSKNPEAKKCDKQEDQSDGISIQNIGGVFIVIFVGIGLACITLAFEYWWYKYRK
GGKVVDVQAKHSDVATKINDGFHAKINKLYPRSRF-

>TcasIR144
MQVSKILLLSSLLLNRDETSKCLDAIFKQPVVVLRGVPKNLQNFDAWKPETYLILAPNATV
LEQMLEKWSTIESFNPRAKFWLLTHWHEIKPKTLTILAKFYIVNVAIVTRTGQVFTYYPYK
YENIAQPDTKPVLLGQCDNVPSFPDKLPKFWRNTTVQVLTKCLLPYVDCSDLDQGLETQI
FDLVQEFLKFKVRRIFDKSFKFGLAKINGSYSASFRFLQEREVDMAMGSFRSVGSTQFRDF
EFSTNHMEDKLVWVVPKARPMVHWVRLVKIFEPSFWGLLVVLTVAMARVFEKMARFTD
EPMGIYRKSGFRVAVLILIGSYLKKTPKRFEMRIIFIFWIYFCMVLNIVFNSNLTNVFFGTFN
TFQVNSFDDIIKSNLEMGLTDDVMHILSQEQNWPEITSTKVISSCAFGPACLNRTIFQRNLV
CCWGERSIKFRMAKFYTTQVHYVDDHLLFFYLLFYFVKGYPIVPQISKMIVQLKSAGFVQ
FIKSKVDKLEPRQGNELTTKILTLKRLEGPFYFLLVGWVGGIMIFGYEVVTYERKRRKKVR
QEVTKILKKKKMRQNEKVKILEI-
>TcasIR41a.1
TKMLFNNFCINILVNFIINNYHKNSRCLLIFTDGDFDYKGEIPTVRIKATNGSFNSYLIFNYH
GCQSVIIYTSNVTALLIKFETEIRLKMERFNERKFLIVPQNPSEDFDKFFNLKQLYFISDLLLV
LPTHNDTIFDLKTHKYVGVIDNNEPVLLDRWFSQNQSFLFGKNLYPNKLQNQLGRPLKM
ATFTYEPYSIIGNVFEQFFENDFILQGKSVGEHHGSELMSAVQFALKYNMTPVPVINEKDY
WGDIFPNWSGNGLLGNLVDDKADVGFSALYTWEFCYHFLELSKPLVRTGITCLVPAPKLS
ERWLTPLFSYSSYLWFCIILTLVIAIFVLSLVLFCYNHNKTLNLNYPLKRKTTYIHFLESAVTI
VLKPVFQQSLTLRELPIEIASKLLMGLVLLLALFLTSSYGSGLATVMTIPTYENAINTVEDFA
NSGLDWGATQDAWIMSIQNAEEQRYVKIVSKFHPISEEELFQFSKSGKFGFSIERLPFEDYA
IGDYIKEDVIDNFHLMKEDLYWEQCVIMLRKNSVLLPALDLFILKIFEAGLISHWQNEAVD
LYMNPKVQRAVKFYRQGQEHTVVKLQWSHVKGPFALLLIGLCISFIIFILELTLKKKRNQF-
>TcasIR41a.2
TLGCLTMTNLNVLLQILLKTYFLNTRCIFLFTDSTIDLQVETPIVYFKVSNTLNPSLIFQHHG
CQNILIHHENASDIFVQFENLIRLNNERFNERKYIVTGHNSLKILLTKQLEYVSDLLLVVPK
QTGHYELITHVYRHQNRSKINEPVLLDVWYSQNHSFRQENDLFPNKLTNQNQRVLKIGTL
SYEPYSVIGKLTVNXSPYYLNLGKDDYSFDGTETSLVYEFVHKYNLTPSFTIMGDDLWGD
VYANWTGIGLFGSVLNDEIDIGYAAVYTWEEYYKFMDYTKTLIRSGVTCLVPAPQLAAGW
VTPLRSFSLGMWIALVIVLLSNTIVLNLLFYRNQKYHXNQLFQILLFNAFSKRFFIDSLTTAI
KLYVQQPLTLTLKRGLLKYFIVTNMIMVLFISSSYSSGLSSVMTVPRYGKSIQTVKDLASSH
LNWTGTTDAWIFSLRQVEEANYENIKNRFVVKTQNDLVTASKQYNFGFSVERLPYGHYA
VGPYIQRDVICNYRIMQEDLYWGQCTFLLRKNSVLLPLLDKLILRVFEAGLEAYWENQVK
CFGRKNMNLRDFLGCLPIHGHVCPKRHYVLYTTYXEHDTIKLTWEHVEGAFAVLVLGYA
ASIFTFVIELILDKVRS-
>TcasIR68a
MIKNLLPYKCVVLISDDIYGGTFTKSWYRRFGPFITFVVIRVDEYEDLLSPFEETQACLDTA
KNEGCQMYLILLSNALQVSRLLRFGDKYRVINTRAKFVLLYDNRLFDKPLFYLWKRIINVI
FIRRYSGQKSDTKKNMPWYEITTVPFPTQITSILIPRRLDIWTKSKFRKGIDLFRDKTSDLRN
QTLKVAAFSHIPGTTKSLQEKTARTVIGNFSGTEVEILQTVSAAMNFHCELYEPVNVDVDL
WGGKQSSGKYTGLVGEMVSTNADIALGDLYYTPYILDLMDLSIPYNTECLTFLTPESLTDN
SWKTLILPFKYFRPAMWAAVLVCLLICGAVFHALARFHETISQNKSQVLEIHTKRKKIIILSI
CPEIEKLDSNLKYTKMREQYKPPRFEGQSIGLYQFSEPFNSVLYTYSMLLLVSLPKLPTGWS
LRMLTGWYWLYCLLLVVAYRASMTAILARPTPRVTIDTLQELVNSRLKCGGWGEINRQFF
KSSLDPITKLIGENFELVNDSNEAVDRVAQGVFAFYENSYYLKEALVKRQLRFQIARTTQN

QSEREMRDIAREDRNLHIMTDCVIKMPISIGLQKNSPIKPRVDKYIRRVLEAGLIKKWLQD
VMASILNAEVQSTQEEMKAIMNMKKFFGAIVALFIGYFISVVVLIVENVYFHFFVKRNPHY
NKYTRSIHHVKKAE-
>TcasIR100l
MPRKLFLWIFFLLVSCYGNLSETHLQFLKRYFVSANSVAISMLQTHHQEVKIRDLAEVISR
KLNSIGTPVVVHENHKSGSLNIIMIVWSLKILRQFLDSLVVPEEKGTYYIIILEQDCATVHSD
FAQILEQFWCEHNVLNVVVQNPCSGGTFYLFLPFEHRDNFWGSCKSWDFNEQMPNKLRN
LNQFPLKISLFLYNPTLIAKLPKGLKTNPRYHNLSASKGYGGLDGFLLRELVDYFNFDPVIV
ENLEEYGRVLPNGTAFGSLGDVVNQRVHFSINSRFLMDYGTKEIEYTFPYISDEICMLVPKS
LKVPTWKTLLKCFNTLSWVLIFVSCLCSTFAWYFVGPSKNLHKLIWQIYCFIVGIPQKIEPS
FSQFVFLLSCFFFNVTIFGIIQGSYFTEFATTSFYPDIDTLEELYESNLPVATHFWFLLDGDTS
DLMTKLKTHKIEATGDCLEQTARQRNIATLGRKSESDLIIRTKYTSRDGTPLVHIVEECHTS
LYLCGIVPKGSHFLAPFNQIITRLFEGGFTTKWYRDVFDGIISEEKPQLDETVSFNSLNMND
LQTAFHILTIGHLFSIMVLIGEVVIKGKHNKKLLT-
>TcasIR100
KVTIIILIMMCLSLPKIQTCPIKINHLKEHFKQVKSARIMILQNEIIVTDWLIMELIKDNKITV
TVQKAIRNFEPFNTSNLTRFEALEFNDTIPTLQTDSTCGHLIIVKNEERLYQYLKSDPGFLIL
NPRHFYAIVAMELFKTNVLREFWSLQVSNILLLDCDTSYTVLPFNGTTIRINAYTQRKLLR
NFHNYFLQVSMQPKPPTAIVKFPKPLRENPIYKDLVPFKDYAGLDGCLLKVLTQRLNMKY
VIVGNGQKYGTVLKNGTTTGTLAWIASNKVQISTNGRFLMTYGTNKLEFTVPYSSDQVC
AVVPKALKIPKIIMLAKSLTPSSWFMIFLIYVICVLIYTLMGSTGSTWTLYAIFHGFPVKIVPT
SRQSFFLTSCMLFSIIIMTIIEGSFFKTFTTTTYYKDINTLEELDESELPIAETFFSFTNDKSRIM
TSLKRKKLVINRDDILEQVARKRNIAKLERKRDIKVRLKTEFLDEEGESRLHVVEECFTTF
YIGFIVPKNSIFLPTFNNVIRRIFESGLTQKWYGDVEFSIFLEKIFKLENNIKHHSFSFDNIVSA
LCVLFIGLSLALLVFFWEVTKXKQITLIYVSLIYCIISRH-
>TcasIR100j
LTLVQVVICLLEVSHYDNEKFVNVYQHFTLVRYLTLTFLNDGVHRIDLNNLVVDLMSRLN
FSMMIKEKRLGKNSTTFQESDPFQGHIMVVYDVKVLLAFLEESTEVVPKARGSFAILFTSL
KCPTHYETNHALKQLWTNHGTANLIAFCDNIYVYHPFSKNDSTWGATLDYSPATETPNLF
RNFNGYLLRVSLFKRPPTALKQVPSYISNNPIYRDLKPGDFAGLDGTLLRFLSNYLNFTVVI
DESHPTHGRVLKNGTITGSLSDVVSHRVDFSANDWFLIDYQTPEIEPTVPFSYDQVCPVVS
KALKVPQWKAFFFIFDLTSWVLIFFMWLCCVFVWHVLNPFRDLSTIIWEICSVLFGNPVNV
VPLSNQHMFLGSCMVLNIIIMGIIQGSVFTDFTTTTFHKDINTLEELDEAGLKIASSAWYLD
FDTTDLIKRLKTKQIRNYIGSYKDTAFKRGMAVLGRKQDVEHMVKVEFVAEDGSPLLHVT
SECLQTFLLVSLFPKGSPFLPTFNNVITRLFEAGLTVKWYQDVTSTGTMLQQMKNFANRRP
TGLFSLNDAKLAFYALFVGYIASFVTFLTEILTKNHHNNVHNHVDVLKAQHHGQVQVDQ-
>TcasIR100n
DTFWIVYQTHFLLTDYLTLHILETEDHKFELRQFTQNILKRVNKYGYFLSVRITKSSLNKRN
KSYHFPSTAYAPSQNLAKLSDDQEFYKAKRLSTDSKHGFALIVWDLTTLHLFLDQDYRTIV
PEGRGTYAIQVVSKQCDVKNEIAFTLQRLWTEYQVINVVAQTPCSCDKTHIFIYHPFVKRE
GFWGLATSHTLDQIKGDSRLISNTLSDFNGFPLRISIFPRTPTAMQTLPKLLHYNPIYRNLTW
SKGFAGLDGLVLATLAEYFNFEVVLVGSLLEDDFGKVLPNGTTVGSLADITERRAVYNAN
ERLVAYFNLDQIDFTVPYTREDICLVVPKAAKIPKWKILFQSLDPQSWCFTLFAYVSCFMF
WYNIGPSRSLPKVSWQMFSFFLGIPTKSFARKLDQVLFLIPCMIFSVVMLGVVQGSFFTKL

TLFSFYQDVNTLEEMADLELPIGAFIWNLIRDDSDVIRRLKSKSVKPPDNIFDMIAAHRNIA
TIETRARAQLLIGSKYVDDDGFPLLHIVNECLTTFLNANIVPKGSALLTVFNAVLGKLFESG
LTRKWNNDVVDSLIAEKMISVNRKRVRTKSFSLYDAQGAFFVILVGYACSVFVFLCEIVLK
XDKICYLALIINKT-
>TcasIR100e
DDFWRVTKNHFLLVNSLTIQVLQTEEHQYDLNQYTVTLLKRLNSLNLLVALRMQEKFLSG
RNFPKHSVTNHTFSTTKPKFDPIGGEELTQLKRLSSDSSKGYFIVIWDVESLHNFLDEDFQV
VVPEARAXYMIHFAFTYSTEACKIVKLQVSSVLTRLWIDNNVFNIIAQTSCLCDLEVYYVHR
PFVKRGGFWGLTNSYQMSEIVENPRIIANPLINFNQFPLKIGIFPRPPTVIETLPKLLTDSPIY
KNLSFSKGFAGVDGLVLGTLAECLNFDTTVITSKPNSYGYIYKNGTATGAIADVIDRRMVF
SANSRFLLIYNTDQLEFTVPYTAEKMCLAVPKALKVYKWSSMFRCFNKLTWVSIICSFGIC
TIFWYLLKWQKLVTALATIAQFLLGVPANVRPNVPQMLFLNSCMGFNIVIMGIIQGFLFQS
FTTTSFYPDINTIEEMVDSELPLRSSIFYFLRIDNSSLIHKLKSRTMAAPPNVYDLVAFHRNIA
TTDIKSHVDFMVRSRYLDEDGWPLIHTVDECFETFLIANIVPKGSAFLTVFNNVITKLLEGG
LTQKWYEDVINSLILENWINLNRNKSKTHAFSLYDLQVAFYVIIMGCAVAILVFVAEIVHKR
RNXNNCCNNHHKNIIFAA-
>TcasIR100f
DDFWVIFSTHFLLATSLTFITVQTNSKQYDLRLLAQAIIQSMDKDQVMTTRHVILHNYAEN
INFNVVFKTGTKKNARDFVTDLLAKTKKLASDSREGFVIITWNVNVLQKFLAQHISEINPR
TRATYLFILISSSDSLRKIKHCLHFLWHKYDILNIVVHVLGCGTTTTLIYRPFCKTKNSWGEI
TAHQIEEIVQQPLLLTNSLQDLNQYPLQVSLFARDPTALTQLPKLLQNNPIYKNLASFYGLD
GSMLSTMAKILNFEVVIVENHDRLPFGRVWPNGTASGTLGDVVNRRVALSSNSRILADYN
TQEIEFTVAYNGDSICVAVPKSLKVPKWRVLFECFDAASWLLTLSVFIVCLCFWYCVALKN
FARILWDVYSFLMGIPTRIVPSRQYFFLSSCMVFNVIILQLLQGWLFTAFTKTVFYPDLDTL
EVLEKTNLPVATNMWFLFKDNSEVIQKLSSRGIGKTPNSLDLVAYSRNICVLDKRQDLELY
SQAKFVGPDGLSLLHIVNQCLTSVLLVNIVPKGSPFLPVFNDIMSRLFESGFTKKWYSDVV
TSRVTEKMVSLGRKERNFSFKIKDLQAAFYVMMAGCVFSLFVFVGELVTHXVFVMNKSS
QSKSHRFLLNCNYGV-
>TcasIR100g
TLFKIAEVTFFMVTMHEEFLSLLFGNYYHTNLYQTVKIQEKFARTNNKTGAWYENVALDQ
KLDPPIDQNWQRVKLRTSDSFEGFIIIVWDPQTLDQFLNQNFSLVVPRARATYFLLFVFSIY
ENCKLVNHILKRFWSEFSVLNIIAQTPYCCNKVYIHRPFVKTTNSWGVTQSYTLTEVTQNL
ALITNPLLDLNQFPLRIALFEKNPTAIRKLPKALQNNPIYRNLSRSKGFAGSDGFLLSAMVE
YLNFDPLIDETLEPMNFGHVLPNGTVCGVLAEVVHKRTDYAGNCRLMTYFGTDGYEFTA
PYSSEKIAMVVPKAGKVPRWRSLFNCFNALSWSLIFSIAIVSTVFWCFLRRSQHLKRASWE
MFAHFVGIPCRVVPSRGQFMFLTACMMFNIIILGIIQGSFFTDFTTTSYYPDLNTLEQVLDSN
LPIMAFAWRLLRTNSSPILQKLEQRSIPYEDNVYELVALYRNVAALDRRLDLELEIKTKYSG
RDGVSPLHIVDESLVTFLTTSLVPKGSPFLVVFNHVIRSMFEAGLTAKWYDDVVTSLIIEHK
HKTPSFGVKYRPFTLQDVQAAFYVIAFGYSCSVFVFWCEIIVKFSGKIKHFHYYFVLI-

7.SNMPs

>ApisSNMP1
MLNNKILESKSLNPRSNMRQMWSHPPLSADFKIYLFNVTNPIEAQKGEKVIIKEIGPYVYH
EWKEKENLIDDIDADTVEFSFKNTFVFDEMSTLPLTGDEIIVMPHLAMIGMVTMTKMMKP
AALGLVNKAIPYLYPDQTSAFMMGTANDIMWNGLDINCTSEEFASVAICSQIRQNSESLHK
ISKDHFKFSLFGVKNGTIESNRYTVKRGYTSPATEVGQVIRFNDKHKMDVGPATSATRYTG
QTPQYFNRSSRRTPTWPSFSGDICRSLTPDYVQETKYNGLNVFEYSAMLVKPEEKCYCLN
KKKCLKPGALDLTNCSGAPIIATLPHFYKSEDYLNNVDGLSPQVEKHKIQMYFEPMTGTPL
LGYKRLQFNIFLKRESKINVMKTLNEDEKLIPLFWVEEGIALNKTWTNQIKNKLYLPITIM
KYVKYIFVVFGIVFIILAVVVNYNSVKTMEITPKY-
>AaegSNMP1
MLIKNRKNLMLKPGTQMRGMFEKIPFPLDFKLYLFHVTNPDVVMKGGKPHVREIGPYFFE
EWKEKYDTVDNEEDDTLTFTLKNTWIFRPDLTKPLTGDEMITIPHPLILGALLMVQRDREA
MMPLVSKGMDIIMNPLTTGFLTTRVMDLLFDGILIDCSSQEFSAKALCSGLESEGAVMPFN
ETHFKFSMFGLKNGTDAGRWVVYRGVKNIMDLGRVVSFNDETEMDIYDGDECNRYIGT
DSTIFPPFLTTKDKLWAWSPEICRSIGAEYGGKSKYAGLPMSFFKLDFGDARNEPEHHCFCR
DPPDICPPKGTIDLAPCLGAPIIGSKPHFYDSDPKLLAAVDGLTPNEKDHDVYIHFQLLSGTP
VSAAKRLMFSMEIEPIRDHAVLGNLPTVILPLFWAEEGASLNKTWTNQLKYTLFLGLRFNT
AVKWLTIIIGTIGTIVGGFMHYKRTTKTVNVTPVQSVNGSSAKGNGAGMTVVGHQPDSKG
GSVTTPVIPSAKDLLQNSRNLPTVIEGLDRPQKVTVTEMQERY*-
>AaegSNMP2
MMVMNTELRQDTPQFKRWEAVPQPLDFKVYIFNVTNPYEVQMGRRPRVVEVGPYVYFQ
YCHKDNIRFSRDRSKVHFSQQQMYVFDAESSYPLTENDQLTVLNMHMNSILQIIDTQAKE
TITNFRSDVNNTLEKIPVVRVIKRIIEKTTPIQSILQLAEDETYDSLRLINAELNRIFGRPDSM
FLRTTPREFLFEGVPFCVNVIGIAKAICKEIEKRNTKTIRVQPDGSMKFSFFNHKNMTNDGT
YTINTGIKEPALTQMIEYWNGRNTLDRWINQSAGSSSKCNKIVGTDGSGYPPFREGVERMT
IFSSDICRTVDIKYVGPSSYEGIPALRFETDSHFLNEIGPEYGNDCYCVNRIPKAIVKNNGCL
YKGALDLSTCFDAPVVLTHPHMMGAAQEYTSLIDGLYPDPEKHQIFVDVEPLTGTPLNGG
KRVQFNMFLRRIDSIRLTDRLQTTLFPVLWIEEGIALNEDMVKLIDDSLMKVLTLLDIVQW
VMIGSGLLLAIIMPIVYFIKRKPSSGSITPTLTTTTSTVSISDGGGLGGNPQK*-
>AcorSNMP1
MQFHARLALASGIILTCITIFGWVAFPNLIKSKIKTGVTLKPGSQLREMYMKIPFPIPFYIYVF
NITNPNEIARGQTPIVQEVGPYEFEEWRSKDFVSENEDDDTMTFYSKETFIFKPGTLNGNE
VLTIPHPLIVAILLTVMRDKPSASNLINKAMNSIFRNPAHPFLTATFREIFFDGVIVNCSVTDF
AGKAVCTQLKTEGAKDFQFIEENVFKFSLLGTKNGTASNKFKVKRGLKDYRQTGMVVE
WNDLTELTTWTTKECNQIKGTEGTVFPGLIPHGSDIPSFAAMLCRSMAAKYLGDSKYDGIP
VSRYTLDFGDQSSNEEDKCYCPAPDECMKKGALDLRKCSGPVVATMPHFYNSDPSYVNG
VKGVNPDKAKHELYLLFNTFTGSPVAARNRLQFSMPFVPMPKVDVMKNVPNTLIPMFWV
EEALDLNKTLTKPLRTMYTTMKVVRIISWIILLGTIGGLGYAGFLYYKRSREIQITPVKKDK
KDNAISTVMSKSADDKGGVSNPAVSDNEIDKY-
>AgamSNMP1
MELKERNFKKIGLICVAVLLCGMVFSYGIFPSILRFMIKQNVLLKPGTQIRDMFEKIPFPLDF
KLHIFNVTNPDEIMRGGKPRVNDIGPLYFEEWKEKYDTVDNVEEDTLTFTLRNTWIFRPDL
SALTGEEIVTIPHPLIMGVLLMVQRDREAMMPLVKKGVNILFDPLESAFLKVRIMDLLFDG
IYVDCSSQDFAAKALCSGMDSEGAVMPHNETHYKFSFFGMRNHTEAGRWVVYRGVKNI

RDLGRVVSYNEETEMDIWDGDECNQYIGTDSTIFPPFLTAQDRLWAWSPEICRSLGAHYVH
KSKYAGLPMSYFELDFGDLKNEPHNHCFCRDAPDDCPPKGTMDLSPCLGGPIIGSKPHFY
GADPKLVEAVDGLAPNKAAHDVYIHFELASICWVSPVSAAKRLQFSMELGPIRDHELFGQ
LPDVILPMFWAEEGASLNKTWTNQLKYQLFLGLKFNATVKWLTIIIGTVGAVGSAYMYFR
KETKTTDVAPVDVSTPDTNPSSAKDGVVNVSLGRNLPPVIDGLDKPPKLRATELQQERY*-
>AgamSNMP2
ATELRQGTDQYKRWEALPQPLDFKVYIFNVTNPYEVMQGRRPKVVEVGPYVYFQYRQK
DNVRFSRDRSKVHFSQQQMYVFDAESSYPLTENDELTVLNMHMNSILQIIDNQAKETITNF
RSDVNNTLEKIPVVRVIKRIIERTTPIQSILQIAEDETYDSLRLINVELNRIFGRPDTMFLRTTP
KQFLFDGVPFCVNVIGIAKAICKEIEKRNTKTIRTMPDGSLRFSFFSHKNMTDDGMFTINTG
IKDPSRTQMIELWNGRTTLDVWNNRSSGLSSSCNKIHGTDGSGYPPFRTGVERMTIFSTDIC
RTVDIKLTGSSSYEGIPALRYEIDNNFLHEIGPEYGNDCYCVNKIPKSIVKSNGCLYKGALD
LSNCFDAPVVLTLPHMLGVAEEYTALIDGMDPEPERHQIFVDVEPYTGTPLNGGKRVQFN
MFLRRIDAIKLTDRLQPTLFPVIWIDEGIALNEDMVKLIDDSLMKVLSLLDVVQWVLIGVG
LLLAVLMPTVYFVKRCRGEGSRTVSPAVTATTSAASLSTVAGVTGDRSK-
>AmelSNMP1
MRFKKLIHDITTRFFHRGVHVILHSSFVKLQSFVTTGRKLHETLRVPVGRKCVTMKPKKL
GIIGGSLLAFGILICAIAFPPFLRSQVKKQIALKDGSEMRELWSNFPVPLDFKIYLFNVTNPM
EITAGEKPILEEVGPFFYDEYKQKVDLVDREEDDSLEYNLKATWFFNPSRSEGLTGEEELIV
PHVLILSMIKLTLEQQPAAMGILNKAVDNIFKKPESVFVRAKAREILFDGLPVDCTGKDFAS
SAICSVLKEKDDALIADGPGRYLFSLFGPKNGTVLPERIRVLRGIKNYKDVGKVTEVNGKT
KLDIWGEGDCNEFNGTDSTIFAPLLTEQDDIVSFAPDICRSMGARFDSYTKVKGINTYHYK
ADLGDMSSHPEEKCFCPSPDSCLTKNLMDLTKCVGAPLIASLPHLLGAEEKYLKMVDGLH
PNEEEHGIAMDFEPMTATPLSAHKRLQFNLYLHKVAKFKLMKNFPECLFPIFWVEEGILLG
DEFVKKLKTVFKTISIVGFMKWFTIVSGTCVSGAAAALFFKNKDKNKLDITKVTPQKGEE
KKWPNQMTISTIQSAAVPPNLDAD-
>AmelSNMP2
MWSYQVCAIICVIFGIYACITNLFSDGLFSIKNAILKNLPLIKGKDMYDEWILPVNLIFKCYF
FNVTNPDEVMEGNNPNLVEYGPFTYREVFEKQIVDVDEELDEIIYDVKSTFTFDKYASLNI
SKRDTVTILNPAYIGTISMLTTLPPSYIEKFGNNIPKLFPNRSSIFLKANPKEILFDGVKLTCN
ERKFPELSTICKTLKALRSPVLKEGEKEGVYYLSIFQRVNGTIRGRFSVNRGVNNISELGNI
GSYNGRRVQTIWRTEKCNTVRGSDTITWAPLINPMPSVLSFIPDLCRSIEADYDKEVSIYGL
IGSRFVMRERTWFLNQSQCYCLERNKVPNCLPQGLIDVSDCLKVPIIMSEPHFLHGDPQLL
MYALGLNPSEDLHETFIVIEPYTGTPLSGQKKIQLNLKLERQPVDLLSNISEGYFPLLWCAN
VRIFSKIIKLQY-
>BmorSNMP1
MQLAKPLKYAAISGIVAFVGLMFGWVIFPAILKSQLKKEMALSKKTDVRKMWEKIPFALD
FKIYLFNYTNAEDVQKGAVPIVKEVGPFYFEEWKEKVEVEENEGNDTINYKKIDVFLFKPE
LSGPGLTGEEVIVMPNIFMMAMALTVYREKPAMLNVAAKAINGIFDSPSDVFMRVKALDI
LFRGIIINCDRTEFAPKAACTTIKKEAPNGIVFEPNNQLRFSLFGVRNNSVDPHVVTVKRGV
QNVMDVGRVVAIDGKTKMNVWRDSCNEYQGTDGTVFPPFLTHKDRLQSFSGDLCRSFKP
WFQKKTSYNGIKTNRYVANIGDFANDPELQCYCDSPDKCPPKGLMDLYKCIKAPMFVSM
PHYLEGDPELLKNVKGLNPNAKEHGIEIDFEPISGTPMVAKQRIQFNIQLLKSEKMDLLKD
LPGTIVPLFWIEEGLSLNKTFVKMLKSQLFIPKRVVSVVCWCMISFGSLGVIAAVIFHFKGD

IMHLAVAGDNSVSKIKPENDENKEVGVMGQNQEPAKVM*-
>BmorSNMP2
MLAKYTKTIFSVSVAFLVVSIVLATWGFPKIIRKQIQKNVQISNTSKMYDKWVKLPMPLDF
KIYVFNVTNRDAINQGEKPNLKEIGPYVYKQYREKIILGYGDNDTIKYNLKKTFVFDPVAS
GDLREDDELTVINFSYMAAIISVQEMMPAAVGMINRALEQFFTNLTDPFQTVKVKDLFFDG
LFLNCEGDNTALGLICGKIRAEKPPTMRISKSANGFYFSMFSHMNRTVSGPYEMVRGTEN
LSDLGHVISYQGKRIMSAWDDQYCGQLNGTDSTIFPPLEDGNIPEKLYTFEPDICRSLFASL
VGKDTLFNISTYYYEISDMTLGSKSANPDNKCFCKrngsvkhdgcllmgvlnlapcqgAPAIASLPHF
YLGSDELADFFGDGIKPDKEKHNTYVHLDPITGVVIKGVKRLQFNIELRNVPSVPQLKEVP
SGLFPLLWIEEGAEIPEWLRKEIMDSHTMLWYVDAARWLVLAVAVVAVLVSATLVARSAAL
IPWPRNSNSISFILGNSVNTSKVHS*-
>CbowSNMP1
MRFPVKLAIGSISAFIFIILVGFVLFPRMITSKVKGMVNLAPGNEIRDMFIKVPFALSFKIYLF
NVTNPMEIQSGEKPIVKEVGPFCYEEWKEKMNIEDKEEDDTISYNQKDTYLKKWWPGCR
NGQEEVTIPHPLILGIVNTVARQKPGALSLINKAIKSIYSDPSSIFLTAKVDDILFDGVVINCN
VSDFAGKALCGQLRTAEALTKVGEVEKFSLFSSKNATLQKRIKAYRGKKNHRDVGRIVEY
NSSKMMDVWPTEECNSIEGTDGTIFPPLTKPGEGLFMFSPDLCRSLIAFFVRKSTYDGIPCG
EFTADLGDMSKNEKEKCYCSTPETCLKKGMMDLYKCSGIPIYASFPHFYNSDTSYLKGVG
GLSPNKTKHEIKILFESITGSPLYARKRLQFSMPLESTQKVELFKNFTGTVLPIFWIEEGVGL
NRTYTGQLKSLFTLTKVVKVSKWLILIGSLGGLAAAGYLFFKVDGRADITPVHEIRRHESK
SGSTVNGAGGHVLSGNGLEKY-
>CbowSNMP2
MKMFGASRFCNVKILFVTTVVATVVLIGVLLLSFVGMPLIVNDQLAKKLRLENNTEQWD
RFVELPVPLNLNVFVFNVTNSDEVTNNKATPILQEIGPYCYEERITRKILSANSTEDSITYEQ
SFNITFDEKRSGQWKESDKIVMVNPLFLILSQITNVIERFVVMGCIDKLFPPKYSTMFFEVD
IKTIMLEGIEFGVASDDIGPACNIVRNKLLEKTLPMKNVERIPSPTDPSVINSLKFAFLQYKIR
GPDGQYTTNRGIDDITQLGHIMRWDHSAEIDVWGRGESTNNATCKEVKGSDSTIYPPHVT
KSTKLDIFSTDICRTVQIRYKGTGTYQGDSGYYFGIDENTFRPATPSPENDCYCIQQTMAPD
GEPSCFLDGVVDVYPCFGAPILLSFPHFLYADESYLDGVIGIDPPNSSIHEIFLLIEPNTGTPL
QGMKRIQLNVVLRPVEFVEYTANLPSTVLPLIWIEEGVNLSQDLLDKLDKMYFNVIKAAD
AAKYAAIGVLTAFVLISGGFFVRKRYFK-
>CbowSNMP3
MKFYSVLFVVKDRANMLNKFNITVSGKIIVILGVFGLFCIFAGFYVGFKAVPDVITDKIWD
MKVLKENTEQWGMFMKTPFPFTFKVYLFDVQNPQEILQGAKPVLRETGPFVYKVYKWK
SEVEWDTPDDISYFSYMRFEFDRKASGIFSEDMKVTLFNTAYYGMLQKIDETQPEVLSTVE
GVLPSIFGENHGLFIKVKVKDYLFDGLKICENEGKDGGFVAGMVCKQMIARLPESKNLRL
EDNSILFSNMHYKNNTHQGRFTVKSGGQNRTETATLTLFNGKSYISSWTGEKSMCNKIRG
ATTVFPVNIEKNMTFEAYSEDICRTIPLEYSAEETVKDIVGYKFSAMNDSFSSTKKENFCYC
TNTTRTLDGEYGCLKDGVTDLKTCIGSSILVSFPHLLYGDEEYLDSVIGLNPEKSKHETTVI
LEPISGFPLSVTQRIQFNTFLRPIDNVISLENVSKSLFPLLWVEESLILDDQYTDMLKNELFR
TIKIVDIVKWVTIGSGAACVLIALILRMSSKTT-
>CbowSNMP4
MRLPLKLGVAGFLLILLSVIVGFIALNPIVRFGIRQQTALKRKSEIRNIYLKLPFPLDFRVYFF
NISNPMEVQKGATPILTEIGPYCYDEFKEKIDVLDNDAEDSLTYYPYDIYKFNAEKSGKLSD

TDYVTILHPALVGMVNQATRDSPALLSIVNKAIGPIFRDPESIYLTAKVKDILFDGVELNCK
VTEFAAKAVCTQIKSQIPGIKSDPEKSIFLFSLLGVKNATVGKSIKVSRGISNSRDLGKVLEF
DGKKVLKLWYEEQCNHFKGTDGWIIPPLLKPEEGLWSFSADLCRNVVAEYVEDSVTKGV
KTRRYEATLADMQNNEEDKCYCPTPKTCLRKGVFDLSKCMGVPILATLPHFLEADEIYLQ
QVKGLNPILDKHIIRIQLEPMTGTPIEARKRLQFNLPVSASEKITLMRNVSTSLHPIFWIEEG
VELDGALLEKVTEVFTFLGVFQVFRWLGLLIGFVSIAYAVYHHMKHSRSVHITPISGSSSSD
HVDINRSTNELVGKMKEVFQSDKGHTNPVMTGHEFDRYS-
>CquiSNMP1a
MVVGLTVSMAILPELVNLMLRQNLRLKPGSDLRKMYEKVPFGLDFKVHIFNITNPQEIMQ
GGRPRVKDIGPFYFEEWKEKYDIEDNDGEDTMTFDMKNTWIFRPDLTAPLTGNEMITVPY
LLVIGVLLAIQRDKEAMLPLISKGLDIIFEPLESAFVTVRVMDLLFDGIPVDCSSEEFAAKAL
CSGLDSEGAVAPLNDTHVKFSMFGLRNGTSIGRFKVYRGIKNVADLGRVITYNDETEMDF
YDGDECNKYVGTDSTIFPPFLTKKDRLWAWSPEICQSLGAVYAGKSSYQGFPTSFFTIDFGD
LRDDPVHQCYCRDPPDGCPPKGTIDLGPCVGAPILGSKPHFIGGDPKLLRDVDGLEPDPKE
HDIFIHYDLQTGTPFSAAKRLQFNLELEPIRGHEVFGKLPKMVLPMFWAEEGASLNKTWT
KQLKPLFMIRKFNATVKWLSIVLGTLGTIGAGFMHYKLHIKPVNVRPMEVQKTTVKEVEP
SVETNGTGKEPPEKIEPRVVESAHRNLPPLFDGGLAGKQKPVPSDQRER*-
>CquiSNMP1b
MKLEELNFKKIAIICACTLVGGLTFCYGIFPPILKFMLKQNVLLKPGTQMRGMFEKMPFPL
DFKIHLFNVSNPEEIMKGGKPKIKDVGPYYFEEWKEKFDTEDDLEEDTLSFTLRNTWKFR
PDLSSPLTGDEMITLPNMLLMGVFLMVQREREAMMPLIRKGAKIIFDPLESAFMTVRVMD
FLFDGLPVDCSSQDFASKALCSGMESEKVVLPLNDTHYQFSIFGGRNATDAGRWVVVYRG
VKNIKDLGRIVSFNGETEMDTYDGDECNQFVGTDSTIFPPFLTKEDRLWAWSPEICRSMGA
TYGGKSKYAGMPMSYFELDFGDLKNEPENHCFCRDPPEDCPPKGTMDLAPCLGAPLLGS
KPHFIDADPKLLEEVQGLEPNREDHDMFINFELISGTPVSAAKRLQFNLEMEPVRDHEVLG
NLPNVILPVFWVQEGVSLNKTWTNQLKYQLFLGLKFNATVKWLTIIIGTVGSIGAGIMHY
KRSTKSVNVTPVEAVSNGSGRIISVSSAGKDREAINNSKNLPAVLDGLGERIPKMAPVEQR
Y*-
>CquiSNMP1c
NVLLKPGTQMRWMFEKIPFPLDFKIHLFNVTNPDEVMKGGKPKIRDVGPYYFEEWKEKF
DTEDDLEEDTLSFTLRNTWIFRPDISAPLTGDEMITVPHLLVLGVFLSVQRDREEMMPLISS
GMKIIFDPLESAFMTVRVMDLLFDGIPVDCGSEEFAAKAVCSGMESEGAVAPLNETHVKFS
MFGMRNATDAGRWVVVYRGVKNIRDLGRIVSYNGEPEMDIYDGDECNQYIGTDSTIFPPFL
TKQDRLWAWAPEICRSLGAHYIGKSKYAGMPMSLFKLDFGDLKNEPENHCFCRDPPEDCP
PKGTMDLSMCIGVPILGSKPHLLDADPKLLEGVDGLEPNEAEHDVFIHFELLSGTPVSGAK
KLQLNLEVEPIRDHEVLGNLPTVVLPMIWVEEGVSLNKTWTNQLKYQLFLGLKFNATVK
WLTIIVGTLGSIGAGFMHYKRTSQVTQVEQVAAGAKSEGGGRFISVSAATTEEGKNGGGG
NLPAVLDGLDPSGISKRMSDPAQKERY*-
>CquiSNMP2
MKARNLNPPAPSQGGKLIKGKVLCSSPWVKAKAGCGAARLAKDNTELRQGTPQFKRFEA
LPQPLDFKVFIFNVTNPYEVQMGKRPRVVEVGPYIYFQYRQKDNIRFSRDRSKVHFSQQQ
LYVFDAESSYPLTENDPLMVLNMHMNSILQIIDNQAKETITNFRSDVNNTLEKIPIVRVIKRI
IEKTTPIQSILQIAEDETYDSLRLINVELNRIFGRPDSMFLRTTPKEFLFDGVPFCVNVIGIAK
AICKEIEKRNTKTIRVLPDGSMKFSFFNHKNMTEDGIYTINTGVKNALETQMIEFWNGKN

MLDKWSNSSRGSSMTCNKIEGTDGSGYPPFREGVQRMTIFSSDICRTVDIKYVGSSSYEGI
PAARYVTDDNFLNKIGPEHNNDCYCVNRIPKAIVKANGCLYEGALDLSTCFDAPVVLTLP
HMMGAAEEYTSLIDGMHPDPEKHQIFVDVEPLTGTPLNGGKRVQFNMFLRRIDSIRLTDR
LPTTLFPVLWIEEGIALNEDMVKLIDDSLMKILTILDIVQWTMIAIGLFLAISMPILYFTKRRP
SSGTITPTLTTTTSAASIPERGGLGGNPDK*
>DmelSNMP1
MQVPRVKLLMGSGAMFVFAIIYGWVIFPKILKFMISKQVTLKPGSDVRELWSNTPFPLHFY
IYVFNVTNPDEVSEGAKPRLQEVGPFVFDEWKDKYDLEDDVVEDTVSFTMRNTFIFNPKE
SLPLTGEEEIILPHPIMLPGGISVQREKAAMMELVSKGLSIVFPDAKAFLKAKFMDLFFRGI
NVDCSSEEFSAKALCTVFYTGEIKQAKQVNQTHFLFSFMGQANHSDSGRFTVCRGVKNN
KKLGKVVKFADEPEQDIWPDGECNTFVGTDSTVFAPGLKKEDGLWAFTPDLCRSLGAYY
QHKSSYHGMPSMRYTLDLGDIRADEKLHCFCEDPEDLDTCPPKGTMNLAACVGGPLMAS
MPHFYLGDPKLVADVDGLNPNEKDHAVYIDFELMSGTPFQAAKRLQFNLDMEPVEGIEP
MKNLPKLILPMFWVEEGVQLNKTYTNLVKYTLFLGLKINSVLRWSLITFSLVGLMFSAYLF
YHKSDSLDINSILKDNNKVDDVASTKEPLPSANPKQSSTVHPVQLPNTLIPGTNPATNPATH
HKMEHRERY-
>DmelSNMP2
MIHWSLIVSALGVCVAVLGGYCGWILFPNMVHKKVEQSVVIQDGSEQFKRFVNLPQPLNF
KVYIFNVTNSDRIQQGAIPIVEEIGPYVYKQFRQKKVKHFSRDGSKISYVQNVHFDFDAVA
SAPYTQDDRIVALNMHMNAFLQVFEREITDIFQGFANRLNSRLNQTPGVRVLKRLMERIR
GKRKSVLQISENDPGLALLLVHLNANLKAVFNDPRSMFVSTSVREYLFDGVRFCINPQGIA
KAICNQIKESGSKTIREKSDGSLAFSFFGHKNGSGHEVYEVHTGKGDPMRVLEIQKLDDSH
NLQVWLNASSEGETSVCNQINGTDASAYPPFRQRGDSMYIFSADICRSVQLFYQTDIQYQ
GIPGYRYSIGENFINDIGPEHDNECFCVDKLANVIKRKNGCLYAGALDLTTCLDAPVILTLP
HMLGASNEYRKMIRGLKPDAKKHQTFVDVQSLTGTPLQGGKRVQFNMFLKSINRIGITEN
LPTVLMPAIWVEEGIQLNGEMVAFFKKKLINTLKTLNIVHWATLCGGIGVAVACLIYYIYQR
GRVVEPPVK-
>HarmSNMP1
MQLPRELKYAAIAGGVALFGLIFGWVLFPTILKSQLKKEMALSKKTDVRKMWEKIPFALD
FKVYIFNFTNAEEVQKGATPILKEIGPYHFDEWKEKVEVEDHEEDDTITYKKRDVFYFNPE
MSAPGLTGEEIVVIPHIFMLGMALTVARDKPAMLNMVGKAMNGIFDDPPDIFLRVKALDIL
FRGMIINCARTEFAPKATCTALKKEGVSGLVLEPNNQFRFSIFGTRNNTIDPHVITVKRGITN
VMDVGQVVAVDGKTEQTIWRDTCNEFQGTDGTVFPPFVPETERIESFSTDLCRTFKPWYQ
KKTSYRGIKTNRYIANIGDFANDPELNCYCAKPDTCPPKGLMDLAPCMKAPMYASMPHFL
DSDPALLSKVKGLNPDVTQHGIEIDYEPITGTPMVAKQRIQFNIQLLKTDKLDLFKDLSGDI
VPLFWIDEGLALNKTFVNMLKHQLFIPKRVVGVLRWWMVSFGSLGAVIGIVFHFRDHIMR
LAVSGDTKVSKVTPEEEEQKDISVIGQAQEPAKVNI-
>HarmSNMP2
MLGKHSKIFFGVSMIFLVIAIVLASWGFQKVVNKQIQKNVQLSNDSKMFDRWVKLPIPLD
FKVYVFNVTNVEEVNRGEKPILKEIGPYVYKQYREKTILGYGPNDTIKYMLKKHFEFDPE
ASGSLTEDDELTVVHFSYLAALLTVHDMMPSLVAIVNKALEQLFPSLDDAFLRVKVRDLFF
DGIYLSCDGDNSALGLVCGKIRSEMPPTMRQAEGSNGFYFSMFSHMNRSESGPYEMIRGR
DNVYELGNIVSYKGQENMPMWGDKYCGQINGSDSSIFPPINENNVPKKIYTFEPDICRSVY
ADLVDKRELFNISTYYYEISETAFAAKSANPNNRCFCKKNWSANHDGCLLMGLLNLMPC

QGAPAIASLPHFYLGSEELLDYFQSGVQPDKEKHNTYVYMDPVTGVVLSGVKRLQFNIEL
RQIDTIPQLKTVPTGLFPMLWLEEGATIPESIQQELRDAHKLLSYVEVAKWFLLTIAIIAVIAS
AVAVARANALLSWPRNSNSVSFILGPSVTQVNKGN-
>HassSNMP1
MQLPRELKYAAIAGGVALFGLIFGWVLFPTILKSQLKKEMALSKKTDVRKMWEKIPFALD
FKVYIFYFTNAEEVQKGATPILKEIGPYHFDEWKEKVEVEDHEEDDTITYKKRDVFYFNPE
MSAPGLTGEEIVVIPHIFMLGMALTVARDKPAMLNMIGKAMNGIFDDPPDIFLRVKVLDIL
FRGMIINCARTEFAPKATCTALKKEGVSGLVLEPNNQFRFSIFGTRNNTIDPHVITVKRGITN
VMDVGQVVAVDGKTEQTIWRDTCNEFQGTDGTVFPPFVPETERIESFSTDLCRTFKPWYQ
KKTSYRGIKTNRYIANIGDFANDPELNCYCSKPDTCPPKGLMDLAPCMKAPMYVSLPHFL
DSDPALLTKVKGLNPDVTQHGIEIDYEPITGTPMVAKQRIQFNIQLLKTDKLDLFKDLSGDI
VPLFWIDEGLSLNKTFVNMLKHQLFIPKRVVGVLRWWMVSFGSLGAVIGIVFHFRDHIMR
LAVSGDTKVSKVIPEVEEQKDISVIGQAQEPAKVNI-
>MsexSNMP1
MRLARGIKYAVIGAGVALFGVLFGWVMFPAILKSQLKKEMALSKKTDVRKMWEKIPFAL
DFKIYLFNYTNPEEVQKGAAPIVKEVGPYYFEEWKEKVEIEDHEEDDTITYRKMDTFYFR
PELSGPGLTGEETIIMPHVFMMSMAITVYRDKPSMMNMLGKAINGIFDNPSDVFMRVNA
MDILFRGVIINCDRTEFAPKAACTAIKKEGAKSLIIEPNNQLRFSLFGLKNHTVDSRVVTVK
RGIKNVMDVGQVVAMDGAPQLEIWNDHCNEYQGTDGTIFPPFLTQKDRLQSYSADLCRS
FKPWFQKTTYYRGIKTNHYIANMGDFANDPELNCFCETPEKCPPKGLMDLTKCVKAPMY
ASMPHFLDADPQMLENVKGLNPDMNEHGIQIDFEPISGTPMMAKQRVQFNMELLRVEKIE
IMKELPGYIVPLLWIEGGLALNKTFVKMLKNQLFIPKRIVSVIRWWLLSFGMLAALGGVIF
HFKDDIMRIAIKGDSSVTKVNPEDGEQKDVSVIGQSHEPPKINM-
>MsexSNMP2
MLAKHSKLFFTGSVVFLIVAIVLASWGFPKIISTRIQKSIQLENSSMMYDKWVKLPIPLIFKV
YFFNVTNAEGINEGERPILQEIGPYVYKQYRERTVLGYGPNDTIKYMLKKNFVFDPEASN
GLTEDDDVTVINFPYMAALLTIQQMMPSAVAMVNRALEQFFSNLTDPFMRVKVKDLLFD
GVFLNCDGDSPALSLVCAKLKADSPPTMRPAEDGVNGYYFSMFSHLNRTETGPYEMVRG
TEDVFALGNIVSYKEKKSVSAWGDEYCNRINGSDASIFPPIDENNVPERLYTFEPEICRSLYA
SLAGKATLFNISTYYYEISSSALASKSANPDNKCYCKKDWSASHDGCLLMGVFNLMPCQ
GAPAIASLPHFYLASEELLEYFEDGVKPDKEKHNTYVYIDPVTGVVLKGVKRLQFNIELRN
MPRVPQLQAVPTGLFPMLWIEEGAVMTPDLQQELRDAHALLSYAQLARWIILAAAIILAIIA
TITVARSTSLISWPRNSNSVNFIIGPMVNDKMR-
>MS|comp162080_c0
MLGKHSKLIFAVSIGFLVVAIIMASWGFQKVVDKQIQKSVQLENDSLMFDKWLKLPMPLD
FKVYVFNVTNVEEVNKGEKPILEEIGPYVYKQYRERTVLGYGPNDTIKYMLRKRFEFDAE
ASGGLTEDDEVTVIHFSYLAALLTVHDMMPSLVGVINKALEQFFPSLEDAFLRVKVRDLFF
DGIYLSCDGDNAALGLVCGKIKGDLPPTMRMAEGSNGFYFSMFSHMNRSETGPYEMNRG
RDNIYELGNIVSYKGQEIMPMWGDKYCGQINGSDSSIFAPINEANVPQKLYTFEPDICRSLY
VDLVEKRELFNISAYYYEISESALAAKSANHDNKCFCRKNWSANHDGCLLMGLLNLMPC
QGAPAIASLPHFYLGSEELLEFFQSGIAPQREKHNSHVYIDPTTGVVLSGVKRLQFNIELRK
IDTIPQLSSVPTGLFPMLWLEEGATIPDSIQQELRDSHKLLGYVEVARWLLLTVAVIAVIASA
VAVARANALLSWPRNSNSVSFILGPSVT-
>MS|comp162251_c0

MLLPKELKYSAIAGGVAVFGLIFGWVLFPVILKGQLKKEMALSKKTDVRKMWETIPFALE
FKVYLFNYTNAEEVQKGAKPILKEIGPYHFDEWKEKVEIEDHEEDDTITYKKRDTFYFNP
ELSAPGLTGEEIVVMPHIFMLGMALTVNRDKPAMLNMVGKAMNGIFDNPPDIFMRVKAL
DILFRGIIINCARTEFAPKATCTALKKEGVSGLIIEPNNQFRFSIFGTRNNTIDPHIITVKRGIQ
NVMDVGQVVAVDGKPEQTIWKGACNEYQGTDGTVFPPFLTENDRIQSFSTDLCRSFKPW
YQKKTSYRGIKTNRYIANIGNFAEDPELQCFCPDPDKCPPKGLMDLAPCIKAPMYASMPH
YLESDPALLNNVKGLNPDINQHGIEIDFEPISGTPMVAKQRIQFNLQLLKTDKIDLFKDLSG
DIVPLFWIEEGLALNKTFVNMLKHQLFIPKRVVGVLRWWMVSFGSLGAVIGIVYHFRDHI
MRLAVSGDTKVSKVTPEEGQEQKDISVIGAQAQEPAKINI-
>PxylSNMP1
MKLPKHLKFAAGAGGAFLFGILFGWVMFPAILKGQLKKEMALSKKTDVRKMWETIPFAL
NFKVYLYNYTNPEEVQKGGVPIIKEVGPYHFDEWKEKVEIEDHEEDDTITYKKRDTFYFN
QEKSGPGLTGEEVITMPHVFMLAMATVVSREKPAMMNMIGKAINGIFDNPADVFIRVKAL
DIMFRGTMINCARTEFAPKAVCTALKKEAVNGLVMEPNNQFRFSLFGSRNGTIDPHVVTV
KRGIKNVMDVGQVVAIDGKPQQDVWRDHCNEYQGTDGTVFPPFLTEHDRLQSFSGDLCR
SFKPWYQKKSFYRGITTHRYIANIGDFANDPELNCFCDGPCPPKGLMDLMKCMKAPMYA
SMPHFLDSDPELLKNVKGLNPDVNEHGIEIDFEPISGTPMVANQRVQFNMQLLKHDKVEL
LNNLPDTIVPLFWIDEGLALNKTFVNMLKFQLFYPKKAVGVIKWLLVTFGGFGLIGCTIYH
YKDRIMSFASSPGSAAVTKVKPEEVEQKDVSVIGQPQEPAKINM-
>PxylSNMP1-like
MVISYPHFYTGEPARDRYVTGLQPDRDKHSSYVIVEPLTGTPFRSVARMQSNLRIHDLSGF
SSEYDKFSNLILPLFWAEYNQEGLPDYIRLTIYYMVVVLPPVSKALLSFFLLCGCYLIAKQI
YTHKLKSESLSATLPFKNKNVNNLARNKLFNYEKETFLKKPS-
>PxylSNMP2-like
MLGKHLRLGFAISLVVLVIAILMAAWGFPKIVQTQIKKNIQLADGSLMFDKWKKLPMPLSF
KVYVFNVTNPDAVDSGERPRLQEVGPFVYKEYREKTILGYGENDTIKYTLKKTFIFDQEAS
GSLTEDDVLTVLNFSYMGALLSVFEIMPGLIPMINQALGVMFQDLTDPFLRVKARDLFFDG
IYLNCVGESSALGIVCGKIRADAPPTMRPSEDGNGFYFSMFSHMNLSEAGPFEMVRGTED
VSQLGHIVSYKGKTSMNTWGDKYCGQLNGSDSSIFPPIDRIRVPERLYTFEPDICRGVVVLS
TVAYVGNQTLSNNKEFHSPHLSFQSSQKLECEPRRLHKNAIHHSSLSNCPRNWSANHDGC
LLMGLLTLMPCQGAPVIASLPHFYLASEELLEFFDGGVKPDKEKHNTYVYLDPVTGVILE
GVRRLQFNLELRNMKGVPQLENVPTGLFPLLWIEEGAILPQSVIDELQHSHTLLSYVEAVR
WIVLGIAILAFLGLAIAVIRTGNIPIFPKQANSVSFILRPGSFNPTDAHKAQ-
>PxylSNMP3
MKFGVRSSAAVLAVGAVVAVAVAIVGYAVVPGIIDETILQEVALENNTIALERFENVPFPLNF
TIHLFSVENGPEVLAGGIPRVRERGPYIYKLYQTRVIEGFNEDTISYRFLQTYEFDKEASFPN
TEDDRVTIANVAYHSVLQVAEQLAPTLLGALSLALDSVFGRELNSPLATVRVGDLVFDGIP
LCRGGGLLASVACAVIRDAAKDIPNMEVQPDGSLVFSLFAHKRDKLGELYKVDRGLQDPL
ALGSILQWNQRDFLPNWAGGPLSQCARLNGTDTGIFPPFIKRDTVLYGFNTDICRSVELRY
QYDTQYSGVPAYRFAANDWFLSNREGCFCLNVTAGVTAPDGCLLHGAQEMYSCIEPTLG
YLVCARKKDYIISGHSILTDDSYSISRVEALGGGWHTTSSPVAVKSNRGTALSPTEIAGSYLV
LTYPHFLFAHPTYANMVVGMTPDLERHRIFLDLEPVSTPYFLRKV-
>TcasSNMP2
MGCSCCTIKVLLVCVVISVALLIVSLALAFKVFPDLLESEVNKAVRLEDGTKQYDRFVELP

FPVDFKVYLFNVSNPQQVLDGTEKPKLEEIGPFVYKQYRKKTILGKNEEEDTISYTQKETF
EFDAEASKPLTEESVVTVLNPALMSIYQLAEDLHLAGAADTCIKQTFENNQGKVFIEANVR
KLLFDGFSFCKNTSPGICGLVNDLICAIAATKRNSDLVLPDYSLIFSYLNYKRKPDDGKYTV
KRGLTNIEKLGHIVAWNDSLYTKFWGEGTTCSEVKGTDSTLYPPRVTTDSAFYIYSTDICRF
VKINYKGEESYKGIDGYLFETSEDTLRSSAPEEDCYCSKLSRDMEGKKSCFLDGVIDMQT
CFGVPVLFSFPHFLWADNKYLSAVEGLNPVEEKHKTYLVVEPNTGTPLKGMKRIQLNGVI
RPIVGIKSMLQTKRALLPLLWIEEGVSLPQKYVDELKSSYFDKVQIVDGVRYALIVISAILV
GAFGIIILRKRSHAKHHV-
>TcasSNMP1a
MRLPVKIAIGCAIGLVVIIVFGFIAFPKMIKGKVKSMINLNKGSEIRQMFVKVPFALDFKIY
MFNVTNPMDVQKGALPVLKEVGPFCFEEWKEKVDLDDNDDEDVMFYNPKDTFYKANG
PGCLDGSQMITMAHPLILGMVNTVVRTKPGAISLISKAINSIYGNPDSIFMTASAMDILFDG
VVIKCGVKDFAGKAVCSQLKEAPDLRHVDENDLAFSFIGPKNATPGKRFKVLRGVKESHD
VGRILEYDNKKEMEVWPTKECNQYKGTDGTVFPPYLTKEEGLASYAPDLCRSLVAVYSG
DTKYDGIPVRIYTATLGDMSKNADEKCYCPTPDTCLKKGMMDLFKCAGVPVYVSLPHFY
ESDESYVKGVVGLNPNKKDHGIQILFESTTGGPVKAAKRLQFNMPLEPNPKLPIFANLPNT
VLPLFWVEEGVALNNTFTKPLKDLFKIMKIVKIAKWLIMLGCLGGLGAAGYLYFSKKGEA
NITPVHKVKPAENGVSTLGGEVNHAMSDNEIEKY-
>TcasSNMP1b
MVKWQRQLKPGNEVRDFYIKLPIPLDFRVYFFNISNPEEVKQGEKPILKQIGPYCYDAYKE
KINVEDDKDNDTLTYNPYDTYFFNQMRTGDLSQDDYVTILHPLTVGIVNAVATQKPQYLS
AVNKALPVIFKENSSIYLTAKVREILFDGVLINCNVKDFSANAVCSQFKGQPAMVEVEKNI
YSFSLLGSRNGSIPTRITIHRGVKNAADIGRVVTIDNKTDLDVWPEPECNAFRGTDGWVFP
SFLEKEDGIWTVASDLCRSFKAQYVEDLKFHGVVVRKYFADLGDMSSNPAEKCFCPAPEK
CLPKGVMDLTKCMKVPLYCTLPHFLRADEKLLQQVEGLSPELERHIIKIYFEPLTGTPMLG
QRRIQFNLQLMPIPKVAMMKTVPEALHPILWIEEGVELEGFLLKKVTSVFTLLKLMTFVRY
IMLGLSIQGILYGGYKLYQESKSKKVSPVQNGTTESKNHNQGKTGGIELPSMNKRNKENT
KNA-
>TcasSNMP1c
MSYKKITIISACCVVTIIGVAYIYAIRDISHRRNVRYKYIDRVNNVSNDVNGGVVSVGYCYD
YKRIDVDNADSTYTYDIYNRSGNSDDYVTIIHVVSVNYVSVKTHYNDAGKSITAKVRDID
GMINCTSRDTAMAVCTIRTKIGISKDYKYAGNGTTRITVRGIKSNGKVAVDNVTKSDWSNC
NYKGTDGWISGRKTIWMHATTCNIHADVGATSNGAVNKYYSDNICTNCSCGIDVTKCTAI
YISHRSDSIRGVKGNDTSHITRIGTSMAIRNVVKKITIMNVSVIHVWVMGVVNGWRMIKT
YTAVMKYISVASGTAYGGYHYKNKKYSKNIVSSK-
>TcasSNMP1
MTSTARRRNIMKKVYKIMDRVYNITNSVNGVVKVGYCYDAKKIDVNGDSTYTYTYNDK
SGRTADDYVTVHIVGIVNTVSRDSIVDRAIKSIKDNIYITTKVRDDGMTINCKVDSATAVCT
KAIGIIKNVYKSIGRNGTNRYKVRGMKKWHGRVVNHKSTVWSTKKCNRRGTDGWIIDK
VGWTYSSDCRNMHVVTSHGVAKYYADGDMSSNDKCYCKTCKGMMDTRCMGVIYATHR
VDKVRRTVRGKITDHIVRVIIGTAKRMNIVKKISMKTAHIWIAIVGKMIKVVVAKVDVVK
YCAVCAVAGSYCYKRKKKAVTVSKTAKA-
